# OMICfpp: a fuzzy approach for paired RNA-Seq counts

**DOI:** 10.1186/s12864-019-5496-5

**Published:** 2019-04-02

**Authors:** Alberto Berral-Gonzalez, Angela L. Riffo-Campos, Guillermo Ayala

**Affiliations:** 1 Grupo de Investigación Bioinformática y Genómica Funcional. Laboratorio 19. Centro de Investigación del Cáncer (CiC-IBMCC, Universidad de Salamanca-CSIC, Campus Universitario Miguel de Unamuno s/n, Salamanca, 37007 Spain; 2 Universidad de La Frontera. Centro De Excelencia de Modelación y Computación Científica, C/ Montevideo 740, Temuco, Chile; 3 Universidad de Valencia. Departamento de Estadística e Investigación Operativa, Avda. Vicent Andrés Estellés, 1, Burjasot, 46100 Spain

**Keywords:** Colorectal cancer, Ordered weight average, Randomization distribution

## Abstract

**Background:**

RNA sequencing is a widely used technology for differential expression analysis. However, the RNA-Seq do not provide accurate absolute measurements and the results can be different for each pipeline used. The major problem in statistical analysis of RNA-Seq and in the omics data in general, is the small sample size with respect to the large number of variables. In addition, experimental design must be taken into account and few tools consider it.

**Results:**

We propose OMICfpp, a method for the statistical analysis of RNA-Seq paired design data. First, we obtain a *p*-value for each case-control pair using a binomial test. These *p*-values are aggregated using an ordered weighted average (OWA) with a given orness previously chosen. The aggregated *p*-value from the original data is compared with the aggregated *p*-value obtained using the same method applied to random pairs. These new pairs are generated using between-pairs and complete randomization distributions. This randomization *p*-value is used as a raw *p*-value to test the differential expression of each gene. The OMICfpp method is evaluated using public data sets of 68 sample pairs from patients with colorectal cancer. We validate our results through bibliographic search of the reported genes and using simulated data set. Furthermore, we compared our results with those obtained by the methods edgeR and DESeq2 for paired samples. Finally, we propose new target genes to validate these as gene expression signatures in colorectal cancer. OMICfpp is available at http://www.uv.es/ayala/software/OMICfpp_0.2.tar.gz.

**Conclusions:**

Our study shows that OMICfpp is an accurate method for differential expression analysis in RNA-Seq data with paired design. In addition, we propose the use of randomized *p*-values pattern graphic as a powerful and robust method to select the target genes for experimental validation.

**Electronic supplementary material:**

The online version of this article (10.1186/s12864-019-5496-5) contains supplementary material, which is available to authorized users.

## Background

The sequencing technologies have provided major advances in the understanding of biological mechanisms. Particularly, within these sequencing technologies, the RNA-Seq has contributed to understanding gene expression, changing our view of the transcriptome [1, 2]. The identification of differentially expressed genes, new transcripts, expressed mutations, among others, has allowed a better understanding of human diseases. New biomarkers or therapeutic targets against diseases such as cancer have been proposed using this technology [3].

However, there is no standard pipeline for the analysis of RNA-Seq data. In fact, each step of the analysis admits many options. The reads can be aligned (or mapped) using different tools. Some widely used aligners are STAR [4], Tophat [5] or Bowtie [6]. Then, the matrix of counts is obtained, i.e the estimation of RNA abundance (cDNA) by the number of aligned read over a gene or isoform. These counts can be obtained using software like HTSeq [7] or featureCounts function of the Rsubread package [8]. The differential expression analysis can be done using the widely used edgeR [9], DESeq [10], among others. Besides, the RNA-Seq data results can be different for each pipeline and it is not established which is the best analysis protocol [11].

There are (and will be) many challenges to solve in mapping, read count and statistical analysis. In this sense, the major problem in statistical analysis of RNA-Seq, and in all omics data, is the small sample size with respect to the large number of variables (genes, isoforms, exon, …). It is not rare that just a few samples determine the results i.e. a great variation accounted by a few observations. Additionally, there exists important confounding variables in the differential expression analysis. They are the library size, the gene length and others [11, 12]. It is not rare that a first differential expression analysis provides several candidate genes that are not significant in a posterior experimental validation. Thereby, the RNA-Seq do not provide accurate absolute measurements [12]. In order to solved it, new methods for RNA-Seq data analysis have been developed [13, 14].

In this paper, we propose a new method for the differential expression RNA-Seq analysis with paired design. Our approach proposes to compare the counts within each pair by taking into account library sizes [15]. The *p*-values for all pairs corresponding to a given gene are aggregated using ordered weighted averages [16]. This aggregated value will quantify the phenotype-expression association from the gene expression profile. These values are used to test differential expression using randomization distributions. Our approach is compared with edgeR [9] and DESeq2 [17] methods for paired samples.

The methodology have been tested using a 68 pairs data set from patients with colorectal cancer. Of these, 50 are obtained from The Cancer Genome Atlas (TCGA) [18] and 18 from PRJNA218851 BioProject [19, 20].

Each pair is composed with a sample from solid tumor and adjacent normal tissue from the same individual. The new methodology has been implemented in the R package OMICfpp and is available at http://www.uv.es/ayala/software/OMICfpp_0.2.tar.gz.

## Methods

### Data

A colorectal cancer paired data set of 50 patients (tumor and normal adjacent tissue) were downloaded from TCGA [18] using **gdc-client** tool. In addition, a colorectal cancer data set of 18 pairs of samples were downloaded from SRA, PRJNA218851 BioProject [19] using the SRA toolkit [20]. The quality control of the PRJNA218851 raw dataset was checked using the FASTQC tool and low quality reads were discarded using fastx-toolkit (http://hannonlab.cshl.edu/fastx_toolkit/). Later, the reads were mapped using STAR with the GRCh38 human genome as the reference one. Then the SAM files were converted to sorted BAM files using Samtools [21]. Finally, the count matrix was generated using the summarizeOverlaps function of GenomicAlignments R package [22]. At this point, we have the counts of both data sets (PRJNA218851 and TCGA), so they are included in a single matrix using SummarizedExperiment R package [23]. A detailed description can be found in the Additional file 1: Methods.

### OMICfpp methodology

The major problem in statistical analysis of omics data is the small sample size with respect to the large number of variables (genes, exons, locii, …). From an statistical point of view we are dealing with counts and covariables describing the samples i.e. a count response model is the suitable approach. These models are part of the generalized linear models and should be the natural approach. However, the small sample sizes do make it more difficult to apply such kind of models. In this paper, we propose a method for RNA-Seq data in paired designs where we tackle the issue of small sample.

**Fig. 1:**
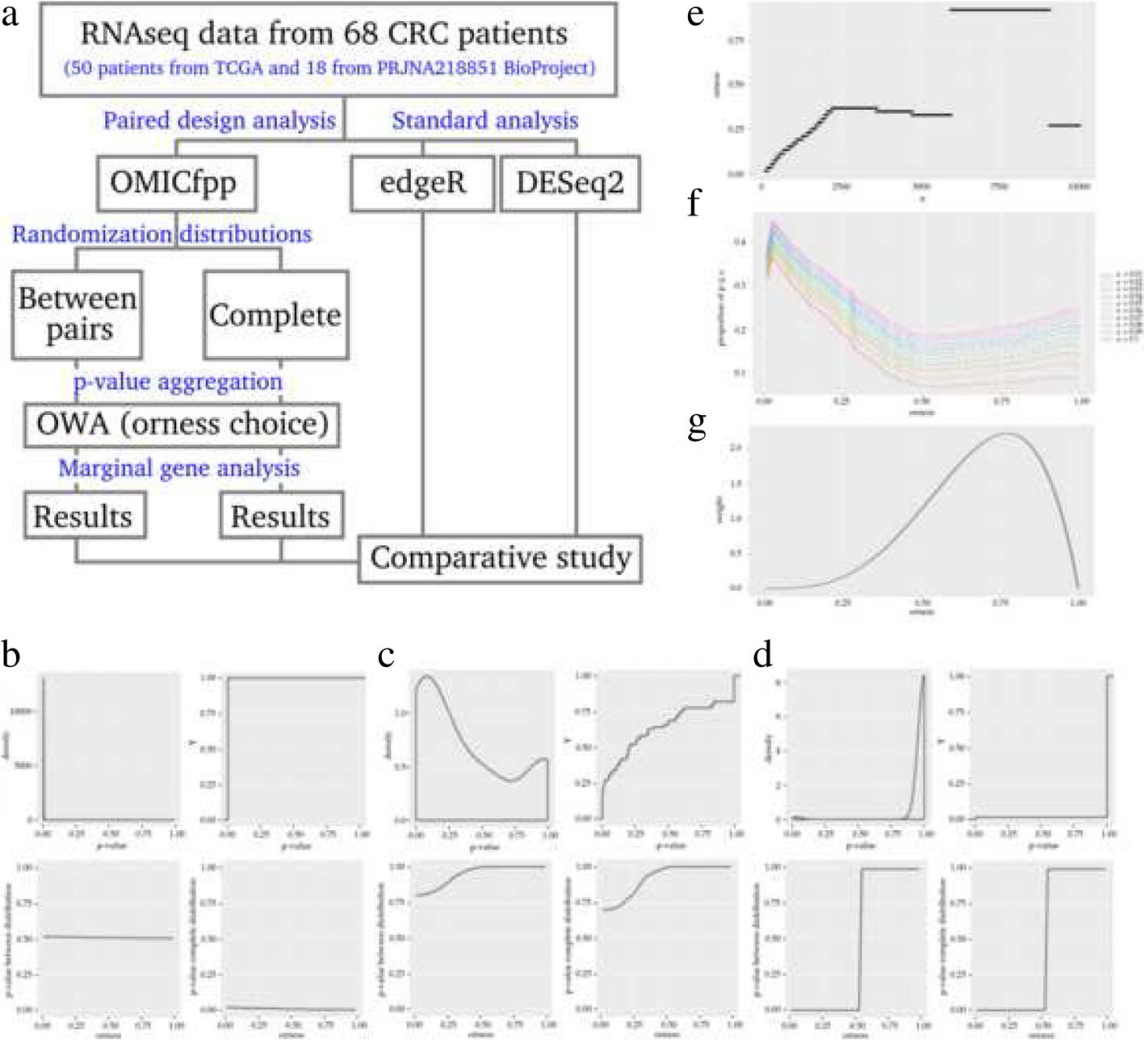
OMICfpp method. **a**) Workflow used by OMICfpp paired data analysis. The 68 paired RNA-Seq data from TCGA and PRJNA218851 BioProject were analyzed by our proposed method OMICfpp and by conventional methods edgeR and DESEq2. In the OMICfpp approach, original and randomized *p*-values are obtained for each paired data, applying different randomization distributions. The *p*-values must be aggregated using the OWA to obtain a single value per gene. The user decides, by choosing an orness, the weights assigned to the genes. Finally, a marginal gene analysis is performed and a list of genes ordered by importance according to the assigned weights is obtained. These results are compared with those obtained using edgeR. **b**) IL11, **c**) HIST2H3C and **d**) AC012414.3 are examples of genes with area under the cumulative distribution function, respectively. Top-left, the kernel density estimator corresponding to the original *p*-values of the binomial test; top-right: the corresponding cumulative distribution function of these original *p*-values; bottom-left: the between-pair *p*-values corresponding to all the values of orness used in the study; bottom-right, the complete *p*-values corresponding to all orness. **e**) Optimal orness by comparing *n*_0_ extreme genes. **f**) Proportion of significant genes for different *α* values obtained using the complete distribution. **g**) Density function used in “Results” section to calculate the score of Eq. 3

In our approach, a *p*-value for each case-control pair is obtained, using a binomial test. These *p*-values are aggregated using an ordered weighted average (OWA) with a given orness previously chosen by the user or using the **chooseOrness** function (from the package OMICfpp) for the automatic orness choice. The aggregated *p*-value from the original data is compared with the aggregated *p*-value obtained using the same method applied to random pairs. These new pairs are generated using a randomization distribution (“Randomization distributions” section). This randomization *p*-value is used as a raw *p*-value to test the differential expression of each gene (“Marginal gene analysis” section). Figure 1a displays the outline of our approach. A detailed software implementation is contained in Additional file 1: Methods.

#### Randomization distributions

The data are paired samples. It will be denoted as (*y*_*i*1_,*y*_*i*2_) the *i*-th pair of counts for a given gene. The whole expression profile would be (*y*_*i*1_,*y*_*i*2_) with *i*=1,…,*n* with 2*n* samples and *N* genes. We are going to consider different randomization distributions.

- **Between-pairs.** The first element of each pair is maintained as the original one. The second element of each pair is obtained permuting the second components of all pairs between them. We have (*y*_*i*,1_,*y*_*γ*(*i*),2_) for *i*=1,…,*n* where *γ* is now a permutation of (1,…,*n*). The number of possible permutations is *n*!.
- **Complete.** Let us choose *I*={*i*_1_,…,*i*_*n*_} a random subset of {1,…,2*n*}. The indices of {1,…,2*n*} not in {*i*_1_,…,*i*_*n*_} can be denoted *J*={*j*_1_,…,*j*_*n*_}. A random correspondence between *I* and *J* will produce the pairs. Cases can be considered controls and the pairs are randomly assigned too. The number of possible values is $\frac {(2n)!}{n!}$.

From now on, they will be named **between-pair** and **complete** distributions. Let (*y*_1_,*y*_2_) be a pair of counts to be compared and (*m*_1_,*m*_2_) the corresponding library sizes. A simple approach to compare the counts by taking into account the library sizes was proposed in [15]. In fact, assuming given the total number of counts per gene and the library sizes, we can test the null hypothesis *H*_*i*_:*p*_*i*1_=*m*_1_/(*m*_1_+*m*_2_) against *H*_*i*_:*p*_*i*1_≠*m*_1_/(*m*_1_+*m*_2_) where *p*_*i*1_ is the proportion of the *i*-th gene in the first sample. Under the null hypothesis, the statistic *Y*_*i*1_ follows a binomial distribution with *Y*_*i*1_+*Y*_*i*2_ trials and the success probability *m*_1_/(*m*_1_+*m*_2_). Note that the null distribution assume that the (random) value of *Y*_*i*1_+*Y*_*i*2_ is given.

Other testing procedures for this null hypothesis could be used and incorporated in our approach. For a given statistical test and for the *i*-th gene we will have $\phantom {\dot {i}\!}(t_{i1},\ldots,t_{i_{n}})$ where *t*_*ij*_ is the statistic or *p*-value obtained in the *j*-th test. It is well known that a few pairs could produce extreme values of these statistics. The simplest approach could be to aggregate the values $\phantom {\dot {i}\!}(t_{i1},\ldots,t_{i_{n}})$ using the mean or a median. In our opinion, a more general and really interesting point of view is to use ordered weighted averages (in short, OWA) [16].

Let us remember this aggregation operators. Let ***a***=(*a*_1_,…,*a*_*n*_) be the column vector of values aggregated and ***a***^′^ is the transpose of the column vector ***a***. Let $\phantom {\dot {i}\!}\boldsymbol {a}_{r} = (a_{r_{1}},\ldots,a_{r_{n}})'$ be the ordered version ***a*** i.e. $a_{r_{1}} \geq \ldots \geq a_{r_{n}}$. An ordered weighted average (OWA) operator of dimension *n* is a mapping $f: {\mathbb R}^{n} \rightarrow {\mathbb R}$ with an associated weighting vector ***w***=(*w*_1_,…,*w*_*n*_) such that ${\sum \nolimits }_{j=1}^{n} w_{j} =1$ and where $f(a_{1},\ldots,a_{n})={\sum \nolimits }_{j=1}^{n} w_{j} a_{r_{j}} = \boldsymbol {w}' \boldsymbol {a}_{r}$. The particular cases shown in Table 1 can better illustrate the idea underlying OWA operators.

**Table 1:** OWA aggregation values using ascending order

*w*	*f*(*a*_1_,…,*a*_*n*_)
(1,0,…,0)	min*ia*_*i*_
(0,0,…,1)	max*ia*_*i*_
$(\frac {1}{n},\frac {1}{n}, \ldots,\frac {1}{n})$	$\frac {1}{n} {\sum \nolimits }_{j=i}^{n} a_{i}$

In this paper we have used the weights proposed in [24]. The method uses, for an orness *δ*, the probability function of a binomial distribution with *n*−1 trials and success probability 1−*δ*: $w_{i} = \binom {n-1}{i-1} (1-\delta)^{i-1} \delta ^{n-i}$ for *i*=1,…,*n*. No weight is associated with any particular input. The relative magnitude of the input decides which weight corresponds to each input. We have chosen this approach with the following problem in mind. A major problem with paired RNA-Seq counts is that just a single pair of samples is responsible for the global observed difference or global effect. The whole pair or just an element of the pair could be an outlier or a real observation. The OWA operator permit us to control the influence of a particular pair. Each pair is marginally evaluated and the obtained statistics (*p*-values) are aggregated by taking into account their ordered values.

The OWA operators are bounded by the maximum and minimum operator. Yager [16] introduced a measure called **orness** to characterize the degree to which the aggregation is like an **or** (max) operation:

1 $$ \text{orness}(\boldsymbol{w}) = \frac{1}{n-1}\sum\limits_{i=1}^{n} (n-i)w_{i}.  $$

Note that orness((1,0,…,0))=1, orness((0,0,…,1))=0 and $\text {orness} \left ((\frac {1}{n},\frac {1}{n}, \ldots,\frac {1}{n}) \right)= 0.5$.

Up to now the OWA has been presented using the usual decreasing ordering. If the original values are increasingly ordered then the interpretation change. In our experiment we will aggregate *p*-values and these ***p***-**values will be increasingly ordered** per gene, from the most significant pair (lowest *p*-value) to the less significant pair (highest *p*-value). An orness near 1 corresponds to the minimum of the *p*-values and an orness near 0 corresponds with the maximum of the *p*-values. Thus, an orness close to one uses the most significant pairs and an orness close to zero will use the less significant pairs. So, when the orness goes from 0 to 1, we are going from the maximum to the minimum of the *p*-values.

#### Marginal gene analysis

The original pairs for a given gene are $\left (y_{i1}^{(0)},y_{i2}^{(0)}\right)$ for *i*=1,…,*n*. First, we choose a given orness *δ* and calculate the weights ***w***. Second, we choose a test to compare both counts, between-pair or complete. Third, we choose a randomization distribution and generates *B* realizations using it being $\left (y^{(b)}_{i1},y^{(b)}_{i2}\right)$ (with *i*=1,…,*n*) the *b*-th realization generated. The statistics observed (for the *n* comparisons) corresponding to the *b*-th realization generated will be $\boldsymbol {t}^{(b)} = \left (t_{1}^{(b)},\ldots,t_{n}^{(b)}\right)$ where *b*=0 corresponds with the original data. The corresponding *p*-values under the null hypothesis of no association with the phenotype would be $\boldsymbol {p}^{(b)} = \left (p_{1}^{(b)},\ldots,p_{n}^{(b)}\right)$. Fourth, we aggregate the generated *p*-values using an ordered weighted average. The *b*-th aggregated value will be $v_{b} = {\sum \nolimits }_{j=1}^{n} w_{j} p_{r_{j}}^{(b)} = \boldsymbol {w}' \boldsymbol {p}_{r}^{(b)}$.

Under the null distribution (any of them) the value *v*_0_ is like *v*_1_,…,*v*_*B*_ and any possible ordering of the vector (*v*_0_,*v*_1_,…,*v*_*B*_) has the same probability. If a one-tail test is used where low values correspond to the alternative hypothesis then the randomization *p*-value is given by

2 $$ p = \frac{|\{b: b=1,\ldots,B; v_{b} < v_{0} \}|}{B},  $$

where |·| denotes the number of elements. This *p*-value measures how extreme is *v*_0_ with respect to the others *v*_*b*_s and depends on the *δ*-orness used and the randomization distribution chosen. From now on, it will be denoted *p*_*b*_(*δ*) and *p*_*c*_(*δ*) for the between and complete distributions and a *δ* orness.

The between-pair *p*-values are evaluating the pair (or sample) factor i.e. we are looking for if there is a pair effect. Different orness will permit us to focus over a certain number of pairs from the lowest to the highest significant pairs. We are going to comment some genes in order to understand the utility of these *p*-values. We think that their interest is not just to declare a gene as significant or non significant. They shows a wider evaluation of the differential expression of the gene with respect to the pair effect (possibly outlier pairs) when the between-pair distribution is used and the condition effect (control vs cases) when the complete distribution is evaluated.

We have chosen three genes of the data used in section “Results” corresponding to extreme cases. Figures 1b, c and d shows a simple graphical description of the different *p*-values used in our approach. The top-left plot shows a kernel density estimator of the raw *p*-values corresponding to the original pairs. The top-right plot shows the empirical cumulative distribution function of these raw *p*-values. The bottom-left (respectively bottom-right) plot shows the between-pair (respectively complete) *p*-values for the different values of orness.

The first gene, Fig. 1b is a significant one with low *p*-values for all pairs. No outlier pair i.e. no pair with a clearly different *p*-value with respect to the other pairs. This can be seen in the plot bottom-left where the *p*_*b*_ is horizontal. The bottom-right shows the gene is considered as significant using any orness.

The second and third genes, Fig. 1c and d are non significant genes, for all orness in all samples Fig. 1c and for some orness Fig. 1d. The gene in Fig. 1d has the highest area under the cumulative distribution function of the original *p*-values. The mass probability is close to one. It is clear in the cumulative distribution function almost null along the whole unit interval.

### Differential expression using edgeR and DESeq2

In order to compare our results with the most used methodologies for differential expression analysis, we analyzed the data using the Bioconductor packages edgeR [9] and DESeq2 [17]. The Additional file 1: Methods contains the code and further details in order to reproduce these studies.

## Results

### Choosing an orness

Many possible methods could be proposed for the orness choice. We have implemented the following procedure where no prior knowledge of the user is assumed. It is a non supervised method. First, an small number of simulations is performed and the randomization *p*-values per gene corresponding to a set of orness values are calculated. For instance, we can take ten simulations and orness from 0.01 to 0.99 with a grid of 50 points. For each orness, we evaluate the mean of the largest *n*_0_*p*-values and the mean of the lowest *n*_0_*p*-values. We choose the orness corresponding to the largest difference between them i.e. the orness where the significant and non significant genes are more clearly distinguished. This is evaluated for different *n*_0_ values and the orness with the greater difference is chosen. The evaluated *n*_0_ values have to be chosen in such a way that the two gene set are clearly contained in the significant and non significant gene sets respectively. It is implemented in the function chooseOrness of the OMICfpp package. The simulation study will use this function. Note that the idea is to choose the orness comparing clearly significant and non significant gene sets. It is convenient to have a previous estimation about the fractions of both kind of genes. It can be estimated by using the procedure proposed in [25] and implemented in the R package [26]. We have used it to choose n_0. The details are in Additional file 1: Methods.

For our data set, values for *n*_0_ from 100 to 10000 with an increment of 10 were chosen. The number of estimated non significant genes (using the method in [25]) gives us a number around 11000 genes, thus we explore up to 10000. For each *n*_0_ the optimal orness is calculated (Fig. 1e). It is clear that there are two clearly defined intervals of *n*_0_ with the same orness within the interval. This figure suggests two possible orness values: 0.37 and 0.93.

The closer the orness to 1, the more stringent is the selection of differentially expressed genes (Fig. 1f and g). Thus, only genes that are significant in most or all samples are reported. It is not always the case that a gene is differentially expressed in all patients, especially when the sample size increases. So, choosing values of orness in the range [0.8, 1] could be a excluding selection. On the other hand, choosing the range [0,0.2] is too permissive. This is illustrated in Fig. 1f, where the proportion of genes with complete *p*-values lesser than a *α* value (from 0.1 to 0.01) in each *δ* orness value are evaluated.

However, the orness could be chosen according to an expert judgment based on previous knowledge. First, a small set of genes with differential expression experimentally verified and a set of housekeeping genes i.e. genes with no differential expression, can be proposed. In this case, we are concerned with colorectal cancer (CRC) data set. Thus, information from the TCGA project, through the web server for cancer and normal gene expression profiling (GEPIA) [27] can be used to select a set of genes with validated differential expression in CRC and other set of housekeeping genes. For instance, the genes *CDH3* [28], *IL11* [29] or *SLC11A1* [30] are experimentally validated as differentially expressed in CRC. Also, the genes *HIST2H3C*, *ACTB* or *RPS23* do not present differential expression in TCGA, have a constitutive function and are not previously described association with CRC, thus can be used as housekeeping. We can replace the data driven procedure with a supervised selection of significant and non significant gene sets.

Finally, the user could choose the orness according with a type of strategy. For instance, a greedy choice could be to use orness close to one i.e. looking for the most significant pairs. Also, a conservative strategies can be choose an orness of 0.5 and a inclusive strategy would use values close to zero i.e. close to the maximum of the *p*-values using the less significant pairs.

### OMICfpp results using an orness value

The between pairs distribution reports the difference between pairs allowing us to identify the influence of outlier pairs. On the other hand, the complete distribution allows us reporting the differences between the controls and cases i.e. the evaluation of the experimental condition. Our methodology allows the evaluation of both experimental factors, although the condition (colorectal cancer in our experimental study) will be evaluated using the complete distribution.

The randomization *p*-values have been estimated using 1000 realizations. Two thousand eight hundred ninety seven genes were differentially expressed using an orness value of 0.37 and 1564 with an orness of 0.93 (*p*-value < 0.001, see Additional file 1: Results). Of these, 501 genes were reported in common. We pretend to order the genes using the *p*-values. Obviously, if we have such a large number of null *p*-values, they can non ordered using only this *p*-value. Then, we have used a second ordering criteria using the score proposed.

The top 30 genes for both orness value are shown in Table 2 and a bibliographic search was conducted in order to determine if the genes of each list were previously reported and validated. It has been found that, using an orness value of 0.37, 46,67% and 70% of the genes were previously reported in colorectal cancer and in another type of cancer, respectively. In addition, the bold entries show that 56.6% of the first 30 genes were also reported by other methods.

**Table 2:** The top 30 genes with differential expression reported by the OMICfpp method using an 0.37 and 0.93 orness value, respectively, with the complete distribution

ENSEMBL ID	Gene symbol	Synonyms	CRC status	Other cancer
Results using an orness value of 0.37				
**ENSG00000001497**	**LAS1L**	**FLJ12525, WTS, Las1-like, dJ475B7.2**	**New**	**Known** [33]
ENSG00000002079	MYH16	MHC20, MYH16P, MYH5	New	New
**ENSG00000003147**	**ICA1**	**ICA69, ICAp69**	**New**	**Known** [34]
ENSG00000005844	ITGAL	CD11A; LFA-1; LFA1A	Known [40]	Known [41]
ENSG00000006071	ABCC8	HI, SUR, HHF1, MRP8, PHHI, SUR1, ABC36, HRINS, TNDM2, SUR1delta2	Known [42]	Known [43]
**ENSG00000006327**	**TNFRSF12A**	**FN14, CD266, TWEAKR**	**New**	**Known** [35]
**ENSG00000006704**	**GTF2IRD1**	**BEN, WBS, GTF3, RBAP2, CREAM1, MUSTRD1, WBSCR11, WBSCR12, hMusTRD1alpha1**	**New**	**known** [36]
**ENSG00000010539**	**ZNF200**	**-**	**New**	**New**
**ENSG00000011201**	**ANOS1**	**HH1, HHA, KAL, KMS, KAL1, ADMLX, WFDC19, KALIG-1**	**Known** [44]	**Known** [45]
ENSG00000013293	SLC7A14	PPP1R142	New	New
ENSG00000015285	WAS	THC, IMD2, SCNX, THC1, WASP, WASPA	Known [46]	Known [47]
ENSG00000015592	STMN4	RB3	Known [48]	Known [49]
**ENSG00000018236**	**CNTN1**	**F3, GP135, MYPCN**	**Known** [50]	**Known** [51]
**ENSG00000018280**	**SLC11A1**	**LSH, NRAMP, NRAMP1**	**Known** [30]	**Known** [52]
**ENSG00000029559**	**IBSP**	**BSP, BNSP, SP-II, BSP-II**	**Known** [53]	**Known** [53]
**ENSG00000030304**	**MUSK**	**CMS9, FADS**	**Known** [54]	**Known** [55]
ENSG00000033122	LRRC7	DENSIN	New	New
**ENSG00000034971**	**MYOC**	**GPOA, JOAG, TIGR, GLC1A, JOAG1**	**New**	**Known** [38]
**ENSG00000036672**	**USP2**	**USP9, UBP41**	**Known** [56]	**Known** [57]
ENSG00000040275	SPDL1	CCDC99, FLJ20364, hSpindly	New	Known [58]
ENSG00000040731	CDH10	-	New	Known [59]
ENSG00000043143	JADE2	PHF15, JADE-2	New	New
**ENSG00000044012**	**GUCA2B**	**-**	**Known** [60]	**New**
ENSG00000046774	MAGEC2	CT10, HCA587, MAGEE1	Known [31]	Known [31]
ENSG00000047617	ANO2	C12orf3, TMEM16B	New	New
**ENSG00000048462**	**TNFRSF17**	**BCM, BCMA, CD269, TNFRSF13A**	**Known** [61]	**Known** [62]
**ENSG00000050030**	**NEXMIF**	**XPN, MRX98, KIDLIA, KIAA2022**	**New**	**New**
ENSG00000053524	MCF2L2	ARHGEF22	New	New
**ENSG00000058600**	**POLR3E**	**SIN; RPC5**	**New**	**New**
**ENSG00000060718**	**COL11A1**	**STL2, COLL6, CO11A1**	**Known** [63]	**Known** [64]
Results using an orness value of 0.93				
ENSG00000001460	STPG1	MAPO2, C1orf201	New	New
**ENSG00000001497**	**LAS1L**	**FLJ12525, WTS, Las1-like, dJ475B7.2**	**New**	**Known** [33]
ENSG00000002822	MAD1L1	MAD1, PIG9, TP53I9, TXBP181	Known [65]	Known [66]
ENSG00000003096	KLHL13	BKLHD2	New	New
**ENSG00000003147**	**ICA1**	**ICA69, ICAp69**	**New**	**Known** [34]
ENSG00000003249	DBNDD1	-	New	New
ENSG00000004487	KDM1A	AOF2, BHC110, KDM1, KIAA0601, LSD1	Known [67]	Known [68]
ENSG00000004848	ARX	SSX, PRTS, CT121, EIEE1, MRX29, MRX32, MRX33, MRX36, MRX38, MRX43, MRX54, MRX76, MRX87, MRXS1	New	Known [69]
ENSG00000005001	PRSS22	BSSP-4, SP001LA, hBSSP-4	Known [70]	Known [71]
ENSG00000005249	PRKAR2B	PRKAR2, RII-BETA	Known [72]	Known [73]
ENSG00000005448	WDR54	-	Known [74]	New
ENSG00000006194	ZNF263	FPM315, ZSCAN44, ZKSCAN12	New	New
**ENSG00000006327**	**TNFRSF12A**	**FN14, CD266, TWEAKR**	**New**	**Known** [35]
**ENSG00000006704**	**GTF2IRD1**	**BEN, WBS, GTF3, RBAP2, CREAM1, MUSTRD1, WBSCR11, WBSCR12, hMusTRD1alpha1**	**New**	**known** [36]
ENSG00000007392	LUC7L	Luc7, SR+89, LUC7B1, hLuc7B1	New	Known [75]
ENSG00000008300	CELSR3	FMI1, EGFL1, HFMI1, MEGF2, ADGRC3, CDHF11, RESDA1	New	Known [76]
**ENSG00000010539**	**ZNF200**	**-**	**New**	**New**
ENSG00000010610	CD4	CD4mut	Known [77]	Known [78]
ENSG00000011143	MKS1	BBS13, FLJ20345, MKS, POC12	New	New
**ENSG00000011201**	**ANOS1**	**HH1, HHA, KAL, KMS, KAL1, ADMLX, WFDC19, KALIG-1**	**Known** [44]	**Known** [45]
ENSG00000011243	AKAP8L	HAP95, NAKAP95	Known [79]	Known [79]
ENSG00000011260	UTP18	WDR50, CGI-48	New	Known [80]
ENSG00000012211	PRICKLE3	Pk3, LMO6	New	New
ENSG00000013523	ANGEL1	Ccr4e, KIAA0759	New	New
**ENSG00000018236**	**CNTN1**	**F3, GP135, MYPCN**	**Known** [50]	**Known** [51]
**ENSG00000018280**	**SLC11A1**	**LSH, NRAMP, NRAMP1**	**Known** [30]	**Known** [52]
ENSG00000018625	ATP1A2	FHM2, MHP2	New	Known [81]
ENSG00000023839	ABCC2	DJS, MRP2, cMRP, ABC30, CMOAT	Known [82]	Known [83]
ENSG00000025772	TOMM34	TOM34, URCC3, HTOM34P	Known [84]	Known [85]
ENSG00000029153	ARNTL2	CLIF, MOP9, BMAL2, PASD9, bHLHe6	Known [86]	Known [87]

The term “known” is assigned if the gene has been previously reported as differentially expressed in colorectal cancer (CRC) or in other types of cancer, otherwise “New” is used. The genes reported in common by OMICfpp with an orness value of 0.37 and 0.93, edgeR and DESeq2 are in bold entries

**Fig. 2:**
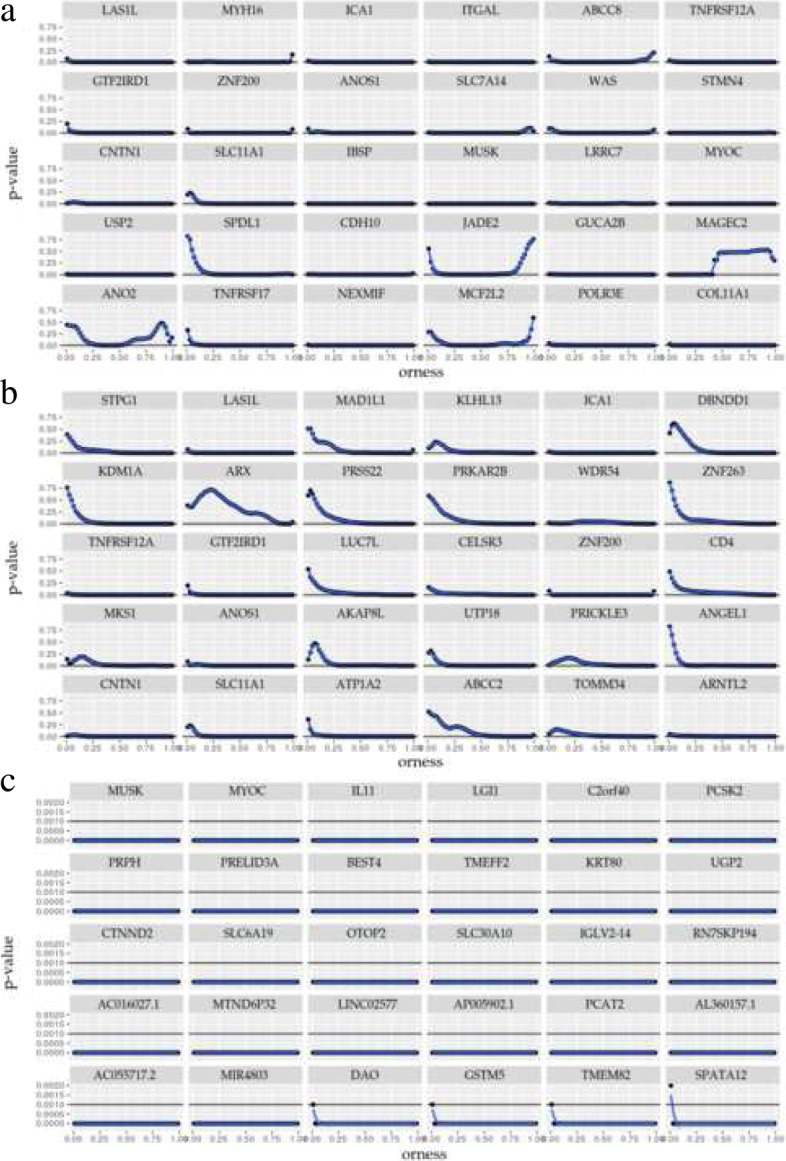
Randomization *p*-values for the 30 most significant genes using an orness value of (**a**) 0.37, (**b**) 0.93 and (**c**) the interval score

Figure 2a displays the randomization *p*-value observed for all the orness values. Although, not all genes have a consistent low *p*-value pattern in a wide range of orness values, all of them show a null *p*-value around the 0.37 orness. For instance, genes such as *ITGAL*, *IBSP* and *GUCA2B* have a consistent low *p*-value pattern, Fig. 2a, and its differential expression in colorectal cancer were verified in previous studies (Table 2). Moreover, some genes that have less clearly defined profiles as *MAGEC2*, Fig. 2a, have also been experimentally validated (Table 2). However, according to our results, it is not differentially expressed in all patients, which is confirmed in the bibliography [31]. This demonstrates the utility of randomized *p*-value profiles for target gene selection. Thus, we can suggest, that the same result can be occur by the *JADE2*, *ANO2* or *MCF2L2* genes, that have not been previously reported.

The results obtained using an orness of 0.93, show that 43.3% and 73.3% of the genes were previously reported in CRC and in another type of cancer, respectively (Table 2). In addition, the bold entries show that only 26.67% of the first 30 genes were also reported by other methods. The genes *ANOS1*, *CNTN1* or *ARNTL2*, with a well defined randomization *p*-values pattern and the genes *MAD1L1* or *ABCC2*, with less clearly defined profiles (Fig. 2b), have been experimentally validated in CRC (Table 2). Also, a considerable number of the first 30 genes (33.3%) reported using an orness of 0.93, were previously experimentally validated.

In view of all the above, we suggest that the genes reported as differentially expressed using OMICfpp, which have not been previously reported in the bibliography, have a high probability of being validated experimentally. Especially those genes that present a defined profile in the randomized *p*-values pattern graphic and, to a lesser extent, those in which the randomized *p*-values pattern is less defined.

### Ordering genes

We have selected in “Choosing an orness” section just two orness values according with an unsupervised method. However, it seems very interesting to explore the results using not just one or two orness values. Instead, we can use many orness values in order to sort the genes in the study. Note that for a given *δ*-orness the value *p*_*c*_(*δ*), randomization *p*-value using the complete distribution and a *δ*-orness, could be interpreted as the membership degree (in fuzzy set terminology) of this gene to be non significant i.e. to belong to the set of “non significant genes”. A high *p*_*c*_(*δ*) corresponds to non significant gene. The integral of this *p*-value with respect to the orness is a good quantification to be used to order the genes from most significant (lowest area) to lowest significant gene (highest area). This area is given by

3 $$ A = \int_{0}^{1} p_{c}(\delta) \psi(\delta) d\delta,  $$

where *ψ* is a density function over the unit interval [0,1]. This aggregated value is like a mean membership degree of the gene to “non significant genes”. This value is calculated for all genes and ordered in increasing order from the most significant to the lowest significant gene. The ordering obtained is consistent with the results in the next section and the whole list can be found in the file score_complete.html in Additional file 1: Results. Note that we use only the complete *p*-value because we are interested in the differential expression. The ordering using the between-pair distribution would order the genes according with the importance of particular pairs to the differential expression.

For our data set the density used for the orness is a beta distribution with parameters (4.310396,1.977092) shown in Fig. 1g. The automatic procedure suggested to use two possible orness values, 0.37 and 0.93. A common criteria is to use an orness close to 0.5 i.e. close to the average. Following this idea, we have chosen a beta distribution giving a probability 0.9 to the interval [.37,.93], a probability 0.05 to the interval [0,0.37] and a probability of 0.05 to the interval [0.93,1]. The probability mass is mainly concentrated in the central interval (0.9) y the two other intervals concentrate an small probability (0.05 each one).

We identify a total of 26 genes with *p*-value < 1.05e-12. The first 30 genes reported are shown in the Table 3. The results indicated that 83.3% of the first 30 genes reported are also reported using edgeR and DESeq2 methods. In addition, only a 30% of the genes have been reported in the CRC bibliography. The randomization *p*-values profiles for the genes of the Table 3, are shown in the Fig. 2c.

**Table 3:** The top 30 genes with differential expression reported by the OMICfpp method using the interval score evaluated using the complete distribution

ENSEMBL ID	Gene symbol	Synonyms	CRC status	Other cancer
**ENSG00000030304**	**MUSK**	**CMS9, FADS**	**Known** [54]	**Known** [55]
**ENSG00000034971**	**MYOC**	**GPOA, JOAG, TIGR, GLC1A, JOAG1**	**New**	**Known** [38]
**ENSG00000095752**	**IL11**	**AGIF, IL-11**	**Known** [29]	**Known** [29]
**ENSG00000108231**	**LGI1**	**EPT, ETL1, ADLTE, ADPAEF, ADPEAF, IB1099, EPITEMPIN**	**New**	**Known** [88]
**ENSG00000119147**	**C2orf40**	**ECRG4, augurin**	**Known** [89]	**Known** [90]
**ENSG00000125851**	**PCSK2**	**PC2, NEC2, SPC2, NEC 2, NEC-2**	**New**	**Known** [91]
**ENSG00000135406**	**PRPH**	**NEF4, PRPH1**	**Known** [92]	**Known** [93]
**ENSG00000141391**	**PRELID3A**	**C18orf43, FLJ31484, HFL-EDDG1, SLMO1**	**New**	**New**
**ENSG00000142959**	**BEST4**	**VMD2L2**	**New**	**New**
**ENSG00000144339**	**TMEFF2**	**TR, HPP1, TPEF, TR-2, TENB2, CT120.2**	**Known** [94]	**Known** [95]
**ENSG00000167767**	**KRT80**	**KB20**	**Known** [96]	**Known** [97]
**ENSG00000169764**	**UGP2**	**UDPG, UGP1, UDPGP, UGPP1, UGPP2, UDPGP2, pHC379**	**Known** [98]	**Known** [99]
**ENSG00000169862**	**CTNND2**	**GT24, NPRAP**	**New**	**Known** [100]
**ENSG00000174358**	**SLC6A19**	**HND, B0AT1**	**New**	**Known** [101]
**ENSG00000183034**	**OTOP2**	**-**	**New**	**New**
**ENSG00000196660**	**SLC30A10**	**ZNT8, ZRC1, HMDPC, ZNT10, ZnT-10, HMNDYT1, DKFZp547M236**	**Known** [102]	**Known** [103]
**ENSG00000211666**	**IGLV2-14**	**-**	**New**	**New**
ENSG00000223260	RN7SKP194	-	New	New
**ENSG00000225335**	**AC016027.1**	**-**	**New**	**New**
ENSG00000227649	MTND6P32	-	New	New
**ENSG00000228742**	**LINC02577**	**-**	**New**	**New**
ENSG00000253233	AP005902.1	-	New	New
**ENSG00000254166**	**PCAT2**	**PCA2, CARLO4, CARLo-4, TCONS00015167**	**New**	**New**
ENSG00000260574	AL360157.1	-	New	New
**ENSG00000261650**	**AC055717.2**	**-**	**New**	**New**
ENSG00000264099	MIR4803	hsa-mir-4803	New	Known [65]
**ENSG00000110887**	**DAO**	**DAAO, OXDA, DAMOX**	**New**	**Known** [104]
**ENSG00000134201**	**GSTM5**	**GTM5, GSTM5-5**	**Known** [105]	**Known** [106]
**ENSG00000162460**	**TMEM82**	**-**	**New**	**Known** [107]
**ENSG00000186451**	**SPATA12**	**SRG5**	**New**	**Known** [108]

The term “known” is assigned if the gene has been previously reported as differentially expressed in colorectal cancer (CRC) or in other types of cancer, otherwise “New” is used. The genes reported in common by OMICfpp, edgeR and DESeq2 are in bold entries

### OMICfpp in a small sample size context

**Fig. 3:**
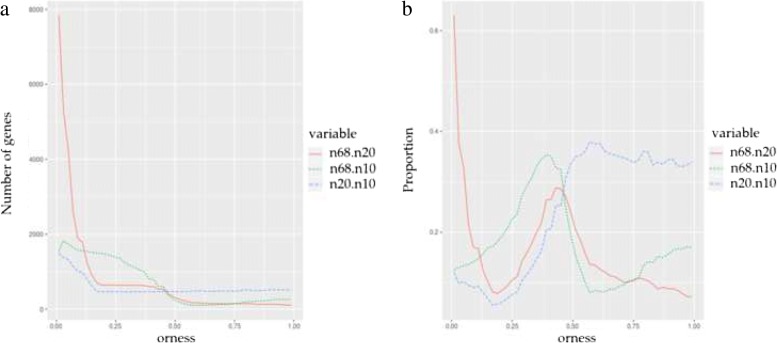
(**a**) Number of genes declared significant i.e. complete *p*-value lesser than *α*=0.001 using 68, 20 and 10 samples. Label n68.n20 corresponds with the proportion of genes declared significant using 68 and 20 for different orness values. Labels n68.n10 and n20.n10 corresponds with the proportion declared significant using 68 and 10 samples and 20 and 10 samples. (**b**) The corresponding proportions with respect to the number of significant genes using 68 pairs

This section contains a small study about the sample size. In our case the sample size refers to the number of pairs used in the study. The original data used in the paper has 68 pairs. We have reproduced the study with two random samples from the original pairs. Firstly we have used 10 pairs and, secondly, a total number of 20 pairs. The results obtained with 10, 20 and 68 pairs will be compared (Fig. 3). We have considered two *α* values and evaluated the number of genes with a *p*-value lesser than *α* for each orness value. In fact, we have plotted the fraction of common significant genes with respect to the total number of genes in the study. These *α* values show two typical behaviour in these plots. Figure 3a corresponds with *α*=0.001. Shows a great overlapping between the results for 68 and 20 pairs for small values of orness. The number of these genes decreases for higher values of orness. Similar comment can be applied to the comparison of 68 with 10 pairs.

As it could be expected when *α* is greater the number of common genes between the three studies is clearly greater. Figure 3b corresponds with *α*=0.001. The power of the study with 68 is much greater and only when we declare significant genes with a higher threshold the results are more similar.

### Comparing OMICfpp, edgeR and DESeq2

We have compared our results with those obtained using the methods edgeR and DESeq2. We had four different methods per gene and four *p*-values for them. The two first will be the complete randomization *p*-values corresponding to the orness values 0.37 and 0.93. The third *p*-value corresponds to the method implemented in the package edgeR [9] and the fourth corresponds to DESeq2 [17].

In order to compare the significant genes by taking into account the four criteria an *α* value of 0.001 have been chose. The significant genes for a given *p*-value is composed by those genes with the *p*-value < 0.001.

**Fig. 4:**
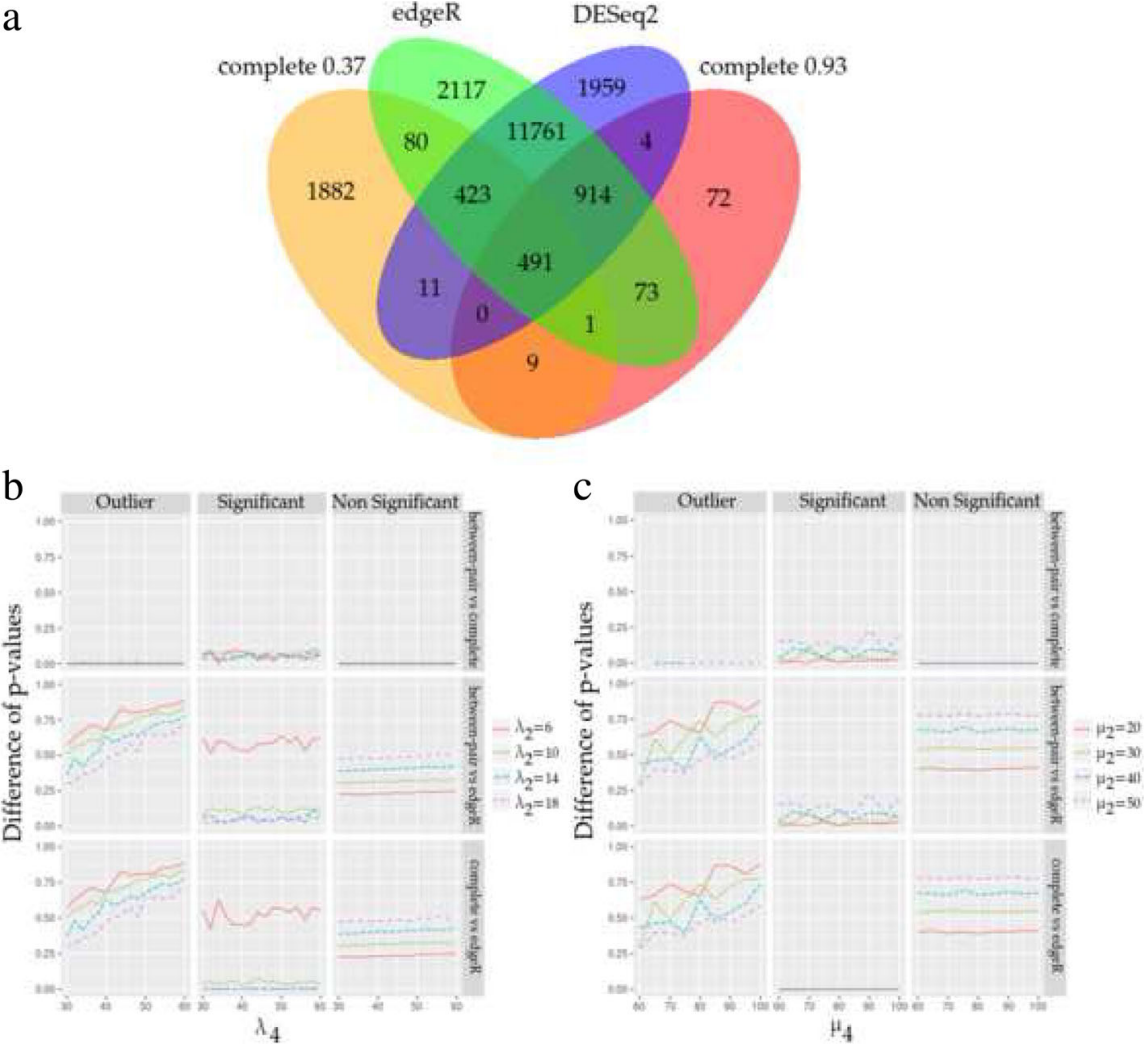
Comparison between OMICfpp, edgeR and DESeq2 results. **a**) Venn diagram comparing the genes with raw *p*-values less than 0.001, using a OMICfpp and the *p*-value obtained by edgeR and DESeq2. **b**) Simulated data set using Poisson distributions. Differences between *p*-values using different methods, different types of genes and all orness. Rows correspond to the comparisons between methods: **bc**, between-pair vs complete distributions; **be**, between-pair distribution vs edgeR method; **ce**, complete distribution vs edgeR methods. **c**) Simulated data set using Negative binomial distributions. Differences between *p*-values using different methods, different types of genes and all orness. Rows correspond to the comparisons between methods: **bc**, between-pair vs complete distributions; **be**, between-pair distribution vs edgeR method; **ce**, complete distribution vs edgeR methods

Under our analysis, edgeR reported 15860 significant genes and DESeq2 reported 15563 genes. Of these, 13589 genes are reported by both methods and 86.5% of these are not reported by OMICfpp (Fig. 4a). OMICfpp method reported 2897 and 1564 genes using an orness value of 0.37 and 0.93, respectively.

OMICfpp reports around 85% fewer genes if a *p*-value < 0.001 is considered. The same applies if an adjusted *p*-value < 0.001 for edgeR (14332 significant genes) and DESeq2 (14606 significant genes) is considered. Thus, our method is more restrictive than edgeR or DESeq2. Thus our method is more restrictive than edgeR or DESeq2. Furthermore, 914 genes are reported in common by edgeR, DESeq2 and OMICfpp using an orness of 0.93 i.e. 54% more that when an orness of 0.37 is used. Moreover, 95.4% of the genes reported using an orness of 0.93 were also reported by the other methods, while in the case of orness of 0.37 only 35% of the genes were reported by the other methods.

The first 30 genes reported by edgeR and DESeq2 are shown in the Tables 4 and 5, respectively. The results obtained using edgeR, show that 66.6% and 60% of the genes were previously reported in CRC and in another type of cancer, respectively. In addition, the bold entries show that 76.67% of the first 30 genes were also reported by the other methods (Table 4). For the DESeq2 results, 80% and 83.3% of the genes were previously reported in CRC and in another type of cancer, respectively, and 70% of the genes are also reported in the other methods (Table 5).

**Table 4:** The top 30 genes with differential expression reported using edgeR method

ENSEMBL ID	Gene symbol	Synonyms	CRC status	Other cancer
**ENSG00000167767**	**KRT80**	**KB20**	**Known** [96]	**Known** [97]
**ENSG00000251026**	**LINC02163**	**-**	**New**	**Known** [109]
**ENSG00000261650**	**AC055717.2**	**-**	**New**	**New**
**ENSG00000142959**	**BEST4**	**VMD2L2**	**New**	**New**
**ENSG00000182938**	**OTOP3**	**-**	**New**	**New**
**ENSG00000164283**	**ESM1**	**endocan**	**Known** [94]	**Known** [110]
**ENSG00000168748**	**CA7**	**CAVII, CA-VII**	**Known** [111]	**New**
**ENSG00000183034**	**OTOP2**	**-**	**New**	**New**
**ENSG00000105989**	**WNT2**	**IRP, INT1L1**	**Known** [112]	**Known** [113]
ENSG00000175832	ETV4	E1AF, PEA3, E1A-F, PEAS3	Known [114]	Known [115]
ENSG00000224269	AP000697.1	-	New	New
ENSG00000120254	MTHFD1L	DKFZP586G1517, FLJ21145, FTHFSDC1, MTC1THFS, dJ292B18.2	Known [116]	Known [117]
**ENSG00000230316**	**FEZF1-AS1**	**-**	**Known** [118]	**Known** [119]
**ENSG00000129474**	**AJUBA**	**JUB, MGC15563**	**Known** [120]	**Known** [121]
**ENSG00000103888**	**CEMIP**	**CCSP1, HYBID, TMEM2L, KIAA1199, IR2155535**	**Known** [122]	**Known** [122]
**ENSG00000163347**	**CLDN1**	**CLD1, SEMP1, ILVASC**	**Known** [123]	**Known** [124]
ENSG00000062038	CDH3	CDHP, HJMD, PCAD	Known [28]	Known [125]
**ENSG00000214039**	**LINC02418**	**-**	**New**	**New**
**ENSG00000174015**	**SPERT**	**CBY2, NURIT**	**Known** [126]	**New**
**ENSG00000060718**	**COL11A1**	**CO11A1, COLL6, STL2**	**Known** [63]	**Known** [64]
ENSG00000163815	CLEC3B	TN, TNA	Known [127]	Known [128]
**ENSG00000164379**	**FOXQ1**	**HFH1**	**Known** [129]	**Known** [130]
**ENSG00000122641**	**INHBA**	**EDF, FRP**	**Known** [131]	**Known** [132]
**ENSG00000172031**	**EPHX4**	**ABHD7, EPHXRP, FLJ90341, EH4**	**Known** [133]	**New**
**ENSG00000167755**	**KLK6**	**Bssp, Klk7, PRSS18, PRSS9, neurosin, SP59**	**Known** [134]	**Known** [135]
ENSG00000226320	LINC01811	-	New	New
**ENSG00000101255**	**TRIB3**	**NIPK, SINK, TRB3, SKIP3, C20orf97, dJ1103G7.3**	**Known** [136]	**Known** [137]
ENSG00000197905	TEAD4	TEF3, RTEF1, TEF-3, EFTR-2, TEFR-1, TCF13L1, hRTEF-1B	Known [138]	Known [139]
**ENSG00000231172**	**AC007099.1**	**LOC101927884**	**New**	**New**
**ENSG00000170373**	**CST1**	**-**	**Known** [140]	**Known** [141]

The term “known” is assigned if the gene has been previously reported as differentially expressed in colorectal cancer (CRC) or in other types of cancer, otherwise “New” is used.The genes reported in common by OMICfpp with an orness value of 0.37 and 0.93, edgeR and DESeq2 are in bold entries

**Table 5:** The top 30 genes with differential expression reported using DESeq2 method

ENSEMBL ID	Gene symbol	Synonyms	CRC status	Other cancer
**ENSG00000142959**	**BEST4**	**VMD2L2**	**New**	**New**
**ENSG00000183034**	**OTOP2**	**-**	**New**	**New**
**ENSG00000167767**	**KRT80**	**KB20**	**Known** [96]	**Known** [97]
**ENSG00000168748**	**CA7**	**CAVII, CA-VII**	**Known** [111]	**New**
ENSG00000062038	CDH3	CDHP, HJMD, PCAD	Known [28]	Known [125]
ENSG00000175832	ETV4	E1AF, PEA3, E1A-F, PEAS3	Known [114]	Known [115]
**ENSG00000164283**	**ESM1**	**endocan**	**Known** [94]	**Known** [110]
**ENSG00000103888**	**CEMIP**	**CCSP1, HYBID, TMEM2L, KIAA1199, IR2155535**	**Known** [122]	**Known** [122]
**ENSG00000060718**	**COL11A1**	**STL2, COLL6, CO11A1**	**Known** [63]	**Known** [64]
**ENSG00000164379**	**FOXQ1**	**HFH1**	**Known** [129]	**Known** [130]
**ENSG00000105989**	**WNT2**	**IRP, INT1L1**	**Known** [112]	**Known** [113]
**ENSG00000163347**	**CLDN1**	**CLD1, SEMP1, ILVASC**	**Known** [123]	**Known** [124]
**ENSG00000122641**	**INHBA**	**EDF, FRP**	**Known** [131]	**Known** [132]
ENSG00000133742	CA1	CAB, CA-I, Car1, HEL-S-11	Known [142]	Known [143]
**ENSG00000170373**	**CST1**	**-**	**Known** [140]	**Known** [141]
**ENSG00000269404**	**SPIB**	**SPI-B**	**Known** [102]	**Known** [144]
**ENSG00000105464**	**GRIN2D**	**EB11, NR2D, EIEE46, GluN2D, NMDAR2D**	**Known** [145]	**Known** [146]
**ENSG00000044012**	**GUCA2B**	**-**	**Known** [60]	**New**
ENSG00000163815	CLEC3B	TN, TNA	Known [127]	Known [128]
ENSG00000182271	TMIGD1	TMIGD, UNQ9372	New	Known [147]
ENSG00000103375	AQP8	AQP-8	Known [148]	Known [149]
**ENSG00000111846**	**GCNT2**	**II, CCAT, IGNT, ULG3, GCNT5, GCNT2C, NACGT1, NAGCT1, CTRCT13, bA421M1.1, bA360O19.2**	**Known** [150]	**Known** [151]
ENSG00000016602	CLCA4	CaCC, CaCC2	Known [152]	Known [153]
**ENSG00000178773**	**CPNE7**	**-**	**New**	**Known** [154]
**ENSG00000214039**	**LINC02418**	**-**	**New**	**New**
**ENSG00000123500**	**COL10A1**	**-**	**Known** [155]	**Known** [156]
**ENSG00000137673**	**MMP7**	**MMP-7, MPSL1, PUMP-1**	**Known** [157]	**Known** [158]
**ENSG00000129474**	**AJUBA**	**JUB, MGC15563**	**Known** [120]	**Known** [121]
ENSG00000135549	PKIB	PRKACN2	New	Known [159]
ENSG00000120254	MTHFD1L	DKFZP586G1517, FLJ21145, FTHFSDC1, MTC1THFS, dJ292B18.2	Known [116]	Known [117]

The term “known” is assigned if the gene has been previously reported as differentially expressed in colorectal cancer (CRC) or in other types of cancer, otherwise “New” is used. The genes reported in common by OMICfpp with an orness value of 0.37 and 0.93, edgeR and DESeq2 are in bold entries

### Simulation study

In order to obtain a more complete evaluation of the OMICfpp method, a simulation study was performed, using Poisson counts (Fig. 4b) and negative binomial distributions approach (Fig. 4c).

In the simulation study using Poisson counts, we consider three types of features (genes for instance): significant genes, non significant genes and outliers genes. We have to simulate random pairs of counts for the three types. We consider four Poisson random variables such that the *i*-th variable *X*_*i*_ follows a Poisson distribution with mean *λ*_*i*_ for *i*=1,…,4. If the gene is non significant then we simulate the random vector (*X*_1_,*X*_3_)=(*x*_1_,*x*_3_). The pair of counts for the pair are (*x*_1_,*x*_1_+*x*_3_). Note that the mean of *X*_3_, *λ*_3_, is small i.e. just an small increment of the count. If the gene is significant then we simulate (*X*_1_,*X*_2_)=(*x*_1_,*x*_2_) and the counts are (*x*_1_,*x*_1_+*x*_2_). Finally, if the gene is an outlier then we have two types of pairs. The first type of pair is as a pair of a non significant gene. The second type of genes is different. We consider a realization of (*X*_1_,*X*_4_)=(*x*_1_,*x*_4_) and the counts are (*x*_1_,*x*_1_+*x*_4_). The mean *λ*_4_ is much greater than *λ*_2_. The idea is to simulate genes with no differential expression for the most of the pairs except for a few ones. We call them outlier genes. This model is implemented in the function rPairedPoisson of the package OMICfpp. We have used 1000 genes with 50 significant, 50 outliers and 900 non significant genes. For each outlier gene we have used 1,2,…,10 outlier pairs for an outlier gene. The orness values used goes from 0.01 to 0.99 with a step of 0.02. The mean of the random variables will be *λ*_1_=10 and *λ*_3_=2. The mean *λ*_2_∈{6,10,14,18} and *λ*_4_ goes from 30 to 60 with an step of 2.

Figure 4b display a simple graphical description. We have three types of genes: outliers, significant and non significant genes. We pretend to compare three methods, between-pair and complete randomization *p*-values and edgeR. These *p*-values have been estimated for each simulated data set. The mean of the differences between each pair of methods has been calculated and displayed in this figure by taking into account the value of *λ*_4_ i.e. more extreme outliers and for different values of *λ*_2_ i.e. more clearly differentiated significant genes. Our *p*-values are sensible to the outliers (first column) and are similar to the results of edgeR when for *λ*_2_ equal to 10, 14 and 18. However, edgeR can detect a difference of *λ*_2_=6 and our methods can not detect it. The non-signficant genes are equally non-detected by all methods. Again, edgeR is more powerful but very sensitive to the outliers. Our methods are not so powerful but they detect the outliers and are not so sensible to them.

A similar model has been performed by replacing the Poisson distribution with the negative binomial distribution (Fig. 4c). Now, the means of the four negative distribution used are *μ*_1_=10, *μ*_3_=2 and *μ*_2_ takes the values 20, 30, 40 and 50. The values for *μ*_4_ goes from 60 to 100 with a step of 5. The dispersion parameter used for all negative distributions has been 1/10. We think the comments given using Poisson distributions can be applied to the study using negative binomial distributions.

It is important to note that the method DESeq2 can not be applied to this simulated data because it probably needs a greater over dispersion in the data. We have had problems with the estimation of the prior distributions. For this reason, we compare our methodology just with the method edgeR.

### Gene expression signatures for colorectal cancer

A total of 491 genes were reported in common for all methods (Fig. 4a), of these 65 genes are within the top 30 previously described (see Tables 2, 3, 4 and 5). These genes are studied in more detail, in order to propose a gene expression signatures for colorectal cancer. A total of 36 genes in common have been previously reported in CRC: *ANOS1*, *CNTN1*, *SLC11A1*, *IBSP*, *MUSK*, *USP2*, *GUCA2B*, *TNFRSF17*, *COL11A1*, *IL11*, *C2orf40*, *PRPH*, *TMEFF2*, *KRT80*, *UGP2*, *SLC30A10*, *GSTM5*, *ESM1*, *CA7*, *WNT2*, *FEZF1-AS1*, *AJUBA*, *CEMIP*, *CLDN1*, *SPERT*, *FOXQ1*, *INHBA*, *EPHX4*, *KLK6*, *TRIB3*, *CST1*, *SPIB*, *GRIN2D*, *GCNT2*, *COL10A1* and *MMP7*. Furthermore, a total of 29 genes in common have not been previously reported in CRC: *LAS1L*, *ICA1*, *TNFRSF12A*, *GTF2IRD1*, *ZNF200*, *MYOC*, *NEXMIF*, *POLR3E*, *LGI1*, *PCSK2*, *PRELID3A*, *BEST4*, *CTNND2*, *SLC6A19*, *OTOP2*, *IGLV2-14*, *AC016027.1*, *LINC02577*, *PCAT2*, *AC055717.2*, *DAO*, *TMEM82*, *SPATA12*, *LINC02163*, *OTOP3*, *LINC02414*, *AC07099.1*, *CPNE7* and *LINC02418* (see Tables 2, 3, 4 and 5). The randomization *p*-value profiles are shown in Fig. 2. In the case of validated genes that are shown in the Fig. 2, all have a defined randomization *p*-value profiles. The same happens with the profiles of the genes not reported in the literature, with the exception of *SPATA12*. Probably *SPATA12* corresponds to a false positive gene, despite being reported by all methods, since this gene is expressed primarily in testis and may play a role in testicular development and spermatogenesis. On the other hand, little or nothing is known about the *AC016027.1*, *AC055717.2* and *AC07099.1* genes, which makes their description difficult. This is the same situation with the coding genes of ncRNA, *LINC02577*, *LINC02163*, *LINC02414*, *LINC02418* and the prostate cancer associated transcript 2 (*PCAT2*) gene. The neurite extension and migration factor (*NEXMIF*) gene has not been previously reported in cancer, thus its function is unknown. The same goes for the RNA polymerase III subunit E (*POLR3E*) gene, the PRELI domain containing 3A (*PRELID3A*) gene, the otopetrin 2 (*OTOP2*) and 3 (*OTOP3*) genes, the immunoglobulin lambda variable 2-14 (*IGLV2-14*) gene and the bestrophin 4 (*BEST4*) gene that encodes a transmembrane proteins. The *LAS1L* gene is essential for cell proliferation and also for biogenesis of the 60S ribosomal subunit [32] and has been previously related with pancreatic cancer [33]. The *ICA1* gene encodes a protein with an arfaptin homology domain that is found both in the cytosol and as membrane-bound form on the Golgi complex and immature secretory granules. This protein binds to the small GTPase Rab2, thus it can be related to cancer [34]. The TNF receptor superfamily member 12A (*TNFRSF12A*) gene is well known in cancer, for example, it is linked to poor prognosis in breast cancer [35]. The *GTF2IRD1* gene encodes a transcription factor protein, that are related to tumor-promotion [36]. The zinc finger protein 200 (*ZNF200*) is a little known gene and in cancer, only variants associated to ovarian cancer have been previously reported [37]. The *MYOC* gene encodes the protein myocilin, which is believed to have a role in cytoskeletal function and it has been previously reported in glaucoma [38]. Thus, this gene has less likely to be validated experimentally in CRC. However, the rest of the genes (*LGI1*, *PCSK2*, *CTNND2*, *SLC6A19*, *DAO*, *TMEM82* and *CPNE7*) could be related to CRC and have been previously identified in other types of cancer. Thus, we propose these 20 genes as new candidate genes.

## Discussion

We develop OMICfpp as a method for statistical analysis of RNA-Seq data with a paired design and small sample size context. OMICfpp, through the orness election allows to the user assign weight to the results, based on each biological context. However, we also provide the alternative of automatic orness selection. Here we use colorectal cancer data, but OMICfpp can be applied to all kinds of biological problems that involve RNA-Seq analysis.

We use the **chooseOrness** function to select an orness value of 0.37 and 0.93. We also tested the possibility of using a probability distribution over the orness and use the score for CRC data analysis. The results suggest that the use of the score is a more robust method for gene selection, whereas a single orness selection is a reasonable method. Besides, a large number of genes reported in the top position using the score, are also reported within the results obtained by a single orness value.

On the other hand, we tested OMICfpp results using different sample sizes (“OMICfpp in a small sample size context” section). It is clear that a smaller sample size will affect more the highest values of orness. For low orness there is a great overlapping between significant genes using lower sample sizes (Fig. 3). These results confirm that the sample size is very important in obtaining results. We suggest to use *p*-values < 0.001 as the cut line for the results obtained using OMICfpp in smaller sample sizes.

The results obtained by OMICfpp method were validated through bibliographic review, and also by a simulation study. An important part of the results are in agreement with the cancer bibliography, validating the OMICfpp method. Also, we compare the results of OMICfpp with those obtained by edgeR [9] and DESeq2 [17]. We obtain a considerably smaller number of candidate genes than edgeR and DESeq2 (Fig. 4), indicating that our method is more accurate. In turn, the results using an orness of 0.93 were also supported by edgeR or DESeq2 by more than 90%. In addition, there is an important coincidence between the top 30 genes reported by OMICfpp, edgeR and DESeq2 methods.

Besides, the simulation study shows that edgeR is more powerful than our procedure. However, the outliers affects more the results of edgeR than ours. If there is a suspect than the differential expression is due to just one or two outlier pairs, then our approach could complement the study.

Moreover, we identify candidate genes not reported by edgeR and DESeq2 methods, which we suggest must be validated. Furthermore, 491 genes are reported by all compared methods (Fig. 4a). Of these, 65 genes are in the top result in all methods and 36 genes have been previously reported in the bibliography as differentially expressed in colorectal cancer (Tables 2, 3, 4 and 4. All of these with a well defined randomization *p*-value profile. Thus, we deepened in the study of the remaining 29 genes, using the biological data obtained in the bibliography and biological data bases, and our randomization *p*-value profiles (Fig. 2). Therefore, we propose the use of randomization *p*-values profiles as an accurate method to select the candidate genes for experimental validation.

Furthermore, in the last 20 years, it has been searched to identify “cancer signature” in terms of diagnosis, prognosis or prediction of therapeutic response [39]. Although the term refers to one or more genes, a biomarker panel with a growing number of genes is currently used. In this sense, we recommend the experimental validation of *LAS1L*, *ICA1*, *TNFRSF12A*, *GTF2IRD1*, *ZNF200*, *NEXMIF*, *POLR3E*, *LGI1*, *PCSK2*, *PRELID3A*, *BEST4*, *CTNND2*, *SLC6A19*, *OTOP2*, *IGLV2-14*, *PCAT2*, *DAO*, *TMEM82*, *OTOP3* and *CPNE7* genes as new targets for gene expression signature in colorectal cancer.

## Conclusions

RNA-Seq is a powerful method to study the complexity of the transcriptome, however there are many challenges to solve. On the one hand, the inclusion of the experimental design in the analysis of the results can contribute to the obtaining of more precise results. In this regard, OMICfpp is an accurate method for differential expression analysis in RNA-Seq data with paired design. On the other hand, a large number of genes identified as differentially expressed *in silico* are not experimentally validated. In this sense, we propose the use of randomized *p*-values profile graphic as a powerful and robust method to select the target genes for experimental validation.

## Additional file

Additional file 1 All procedures and data needed to reproduce the whole study have been included in the file SupplementaryMaterial.tar.gz. Once decompressed the file SupplementaryMaterialMethods.pdf contains a detailed description of the methods used and the results obtained. The whole paper can be reproduced reading this file. Other data files generated during the analysis are included in the folder Methods. The detailed html reports with the results can be found in the folder Results. (GZ 118,244 kb)

## Abbreviations

CRC: Colorectal cancer
OWA: Ordered Weighted Average
RNA-Seq: RNA sequencing TCGA: The Cancer Genome Atlas
