# Supplementary material for: OMICfpp: a fuzzy approach for paired RNA-Seq counts
Source: BMC Genomics. 2019 Apr 2;20:259. doi: 10.1186/s12864-019-5496-5 (PMC6444640; doi:10.1186/s12864-019-5496-5)
Supplement: Supplementary file 1 — All procedures and data needed to reproduce the whole study have been included in the file SupplementaryMaterial.tar.gz. Once decompressed the file SupplementaryMaterialMethods.pdf contains a detailed description of the methods used and the results obtained. The whole paper can be reproduced reading this file. Other data files generated during the analysis are included in the folder Methods. The detailed html reports with the results can be found in the folder Results. (GZ 118,244 kb) [file 12864_2019_5496_MOESM1_ESM.gz › SupplementaryMaterial/Results/edgeR.html]

edgeR


## edgeR

| gene | edgeR | edgeR\_adj |
| --- | --- | --- |
| gene | edgeR | edgeR\_adj |
| ENSG00000000005 | 2.90e-01 | 3.70e-01 |
| ENSG00000000419 | 3.11e-09 | 2.26e-08 |
| ENSG00000000457 | 2.31e-02 | 4.24e-02 |
| ENSG00000000460 | 6.27e-19 | 3.12e-17 |
| ENSG00000000938 | 1.07e-01 | 1.59e-01 |
| ENSG00000000971 | 1.42e-10 | 1.30e-09 |
| ENSG00000001036 | 1.41e-01 | 2.02e-01 |
| ENSG00000001084 | 4.21e-02 | 7.14e-02 |
| ENSG00000001167 | 3.67e-13 | 5.37e-12 |
| ENSG00000001460 | 1.49e-15 | 3.41e-14 |
| ENSG00000001461 | 1.58e-08 | 1.02e-07 |
| ENSG00000001497 | 4.83e-19 | 2.48e-17 |
| ENSG00000001561 | 4.18e-04 | 1.20e-03 |
| ENSG00000001617 | 1.92e-09 | 1.44e-08 |
| ENSG00000001626 | 1.09e-01 | 1.62e-01 |
| ENSG00000001629 | 1.77e-02 | 3.36e-02 |
| ENSG00000001630 | 3.54e-04 | 1.03e-03 |
| ENSG00000001631 | 7.10e-06 | 2.88e-05 |
| ENSG00000002016 | 6.43e-02 | 1.03e-01 |
| ENSG00000002079 | 2.67e-14 | 4.82e-13 |
| ENSG00000002330 | 1.98e-10 | 1.76e-09 |
| ENSG00000002549 | 4.77e-01 | 5.60e-01 |
| ENSG00000002586 | 2.68e-03 | 6.39e-03 |
| ENSG00000002587 | 7.66e-09 | 5.21e-08 |
| ENSG00000002726 | 3.84e-15 | 8.20e-14 |
| ENSG00000002745 | 6.27e-04 | 1.74e-03 |
| ENSG00000002746 | 2.74e-08 | 1.70e-07 |
| ENSG00000002822 | 5.89e-09 | 4.09e-08 |
| ENSG00000002834 | 2.40e-03 | 5.79e-03 |
| ENSG00000002919 | 8.48e-04 | 2.28e-03 |
| ENSG00000002933 | 8.76e-01 | 9.08e-01 |
| ENSG00000003056 | 1.31e-10 | 1.20e-09 |
| ENSG00000003096 | 2.99e-05 | 1.08e-04 |
| ENSG00000003137 | 3.51e-08 | 2.14e-07 |
| ENSG00000003147 | 7.00e-15 | 1.41e-13 |
| ENSG00000003249 | 5.10e-23 | 8.27e-21 |
| ENSG00000003393 | 9.51e-09 | 6.35e-08 |
| ENSG00000003400 | 1.99e-08 | 1.26e-07 |
| ENSG00000003402 | 1.71e-04 | 5.32e-04 |
| ENSG00000003436 | 7.05e-03 | 1.50e-02 |
| ENSG00000003509 | 3.04e-06 | 1.31e-05 |
| ENSG00000003756 | 3.16e-01 | 3.97e-01 |
| ENSG00000003987 | 6.11e-07 | 3.00e-06 |
| ENSG00000003989 | 7.23e-05 | 2.42e-04 |
| ENSG00000004059 | 6.89e-03 | 1.47e-02 |
| ENSG00000004139 | 6.67e-01 | 7.33e-01 |
| ENSG00000004142 | 5.68e-03 | 1.24e-02 |
| ENSG00000004399 | 3.51e-05 | 1.25e-04 |
| ENSG00000004455 | 5.68e-01 | 6.44e-01 |
| ENSG00000004468 | 1.20e-06 | 5.60e-06 |
| ENSG00000004478 | 7.49e-09 | 5.11e-08 |
| ENSG00000004487 | 2.82e-12 | 3.51e-11 |
| ENSG00000004534 | 1.61e-02 | 3.09e-02 |
| ENSG00000004660 | 6.86e-10 | 5.56e-09 |
| ENSG00000004700 | 2.21e-05 | 8.20e-05 |
| ENSG00000004766 | 8.91e-01 | 9.19e-01 |
| ENSG00000004776 | 5.77e-12 | 6.75e-11 |
| ENSG00000004777 | 2.23e-05 | 8.26e-05 |
| ENSG00000004779 | 8.89e-01 | 9.17e-01 |
| ENSG00000004799 | 3.62e-21 | 3.39e-19 |
| ENSG00000004809 | 9.40e-01 | 9.56e-01 |
| ENSG00000004838 | 1.85e-04 | 5.73e-04 |
| ENSG00000004846 | 1.09e-14 | 2.12e-13 |
| ENSG00000004848 | 2.37e-04 | 7.15e-04 |
| ENSG00000004864 | 9.37e-01 | 9.53e-01 |
| ENSG00000004866 | 6.59e-17 | 2.03e-15 |
| ENSG00000004897 | 3.11e-02 | 5.49e-02 |
| ENSG00000004939 | 3.45e-06 | 1.48e-05 |
| ENSG00000004948 | 9.86e-01 | 9.90e-01 |
| ENSG00000004961 | 7.61e-02 | 1.19e-01 |
| ENSG00000004975 | 2.41e-01 | 3.17e-01 |
| ENSG00000005001 | 1.37e-21 | 1.50e-19 |
| ENSG00000005007 | 9.76e-04 | 2.58e-03 |
| ENSG00000005020 | 5.19e-06 | 2.15e-05 |
| ENSG00000005022 | 6.43e-02 | 1.03e-01 |
| ENSG00000005059 | 3.91e-02 | 6.70e-02 |
| ENSG00000005073 | 2.92e-05 | 1.06e-04 |
| ENSG00000005075 | 1.34e-02 | 2.63e-02 |
| ENSG00000005100 | 7.02e-14 | 1.17e-12 |
| ENSG00000005102 | 3.50e-05 | 1.25e-04 |
| ENSG00000005108 | 7.73e-05 | 2.57e-04 |
| ENSG00000005156 | 2.67e-14 | 4.82e-13 |
| ENSG00000005175 | 5.87e-06 | 2.41e-05 |
| ENSG00000005187 | 4.30e-09 | 3.05e-08 |
| ENSG00000005189 | 1.16e-06 | 5.42e-06 |
| ENSG00000005194 | 8.17e-09 | 5.53e-08 |
| ENSG00000005206 | 2.39e-02 | 4.36e-02 |
| ENSG00000005238 | 6.46e-12 | 7.48e-11 |
| ENSG00000005243 | 4.56e-02 | 7.65e-02 |
| ENSG00000005249 | 4.26e-17 | 1.38e-15 |
| ENSG00000005302 | 1.30e-07 | 7.17e-07 |
| ENSG00000005339 | 1.74e-01 | 2.41e-01 |
| ENSG00000005379 | 3.12e-01 | 3.93e-01 |
| ENSG00000005381 | 2.19e-03 | 5.33e-03 |
| ENSG00000005421 | 9.76e-01 | 9.82e-01 |
| ENSG00000005436 | 1.17e-01 | 1.72e-01 |
| ENSG00000005448 | 1.65e-11 | 1.76e-10 |
| ENSG00000005469 | 2.43e-11 | 2.52e-10 |
| ENSG00000005471 | 8.80e-04 | 2.35e-03 |
| ENSG00000005483 | 1.84e-03 | 4.57e-03 |
| ENSG00000005486 | 1.33e-04 | 4.22e-04 |
| ENSG00000005513 | 2.36e-04 | 7.14e-04 |
| ENSG00000005700 | 5.76e-01 | 6.51e-01 |
| ENSG00000005801 | 6.60e-10 | 5.36e-09 |
| ENSG00000005810 | 6.59e-03 | 1.41e-02 |
| ENSG00000005812 | 5.82e-07 | 2.86e-06 |
| ENSG00000005844 | 1.18e-09 | 9.24e-09 |
| ENSG00000005882 | 4.16e-10 | 3.51e-09 |
| ENSG00000005884 | 1.05e-07 | 5.91e-07 |
| ENSG00000005889 | 8.16e-01 | 8.59e-01 |
| ENSG00000005893 | 7.88e-04 | 2.13e-03 |
| ENSG00000005961 | 4.28e-01 | 5.11e-01 |
| ENSG00000005981 | 6.68e-03 | 1.43e-02 |
| ENSG00000006007 | 7.65e-13 | 1.06e-11 |
| ENSG00000006015 | 1.00e-08 | 6.66e-08 |
| ENSG00000006016 | 1.12e-02 | 2.25e-02 |
| ENSG00000006025 | 7.03e-05 | 2.36e-04 |
| ENSG00000006042 | 2.60e-06 | 1.14e-05 |
| ENSG00000006047 | 2.26e-03 | 5.49e-03 |
| ENSG00000006062 | 2.66e-04 | 7.95e-04 |
| ENSG00000006071 | 4.50e-14 | 7.77e-13 |
| ENSG00000006118 | 1.16e-25 | 5.10e-23 |
| ENSG00000006125 | 3.46e-02 | 6.02e-02 |
| ENSG00000006128 | 7.81e-04 | 2.12e-03 |
| ENSG00000006194 | 2.49e-17 | 8.45e-16 |
| ENSG00000006210 | 1.02e-01 | 1.53e-01 |
| ENSG00000006282 | 1.28e-02 | 2.53e-02 |
| ENSG00000006283 | 1.91e-01 | 2.60e-01 |
| ENSG00000006327 | 2.08e-25 | 7.97e-23 |
| ENSG00000006377 | 4.05e-06 | 1.71e-05 |
| ENSG00000006432 | 7.15e-02 | 1.13e-01 |
| ENSG00000006451 | 5.05e-03 | 1.12e-02 |
| ENSG00000006453 | 3.51e-03 | 8.11e-03 |
| ENSG00000006459 | 9.41e-02 | 1.43e-01 |
| ENSG00000006468 | 5.98e-01 | 6.71e-01 |
| ENSG00000006530 | 1.16e-09 | 9.05e-09 |
| ENSG00000006534 | 3.36e-03 | 7.81e-03 |
| ENSG00000006555 | 4.80e-16 | 1.23e-14 |
| ENSG00000006576 | 3.94e-02 | 6.75e-02 |
| ENSG00000006606 | 8.08e-06 | 3.25e-05 |
| ENSG00000006607 | 4.12e-01 | 4.95e-01 |
| ENSG00000006611 | 5.19e-01 | 5.99e-01 |
| ENSG00000006625 | 7.38e-13 | 1.02e-11 |
| ENSG00000006634 | 9.25e-12 | 1.04e-10 |
| ENSG00000006638 | 1.42e-01 | 2.03e-01 |
| ENSG00000006652 | 3.16e-05 | 1.14e-04 |
| ENSG00000006659 | 8.46e-01 | 8.83e-01 |
| ENSG00000006695 | 6.12e-08 | 3.58e-07 |
| ENSG00000006704 | 3.33e-26 | 1.75e-23 |
| ENSG00000006712 | 5.40e-01 | 6.18e-01 |
| ENSG00000006715 | 3.06e-03 | 7.19e-03 |
| ENSG00000006740 | 1.58e-20 | 1.20e-18 |
| ENSG00000006744 | 1.81e-02 | 3.42e-02 |
| ENSG00000006747 | 4.39e-17 | 1.42e-15 |
| ENSG00000006756 | 9.42e-02 | 1.43e-01 |
| ENSG00000006757 | 3.57e-01 | 4.39e-01 |
| ENSG00000006788 | 2.86e-01 | 3.66e-01 |
| ENSG00000006831 | 1.10e-02 | 2.21e-02 |
| ENSG00000006837 | 2.98e-07 | 1.55e-06 |
| ENSG00000007001 | 2.30e-01 | 3.04e-01 |
| ENSG00000007038 | 3.52e-03 | 8.14e-03 |
| ENSG00000007047 | 8.75e-04 | 2.34e-03 |
| ENSG00000007062 | 2.42e-01 | 3.17e-01 |
| ENSG00000007080 | 9.33e-05 | 3.06e-04 |
| ENSG00000007129 | 2.14e-05 | 7.96e-05 |
| ENSG00000007168 | 2.57e-13 | 3.88e-12 |
| ENSG00000007171 | 3.49e-01 | 4.31e-01 |
| ENSG00000007174 | 1.22e-04 | 3.92e-04 |
| ENSG00000007202 | 5.70e-01 | 6.46e-01 |
| ENSG00000007216 | 4.32e-09 | 3.07e-08 |
| ENSG00000007237 | 2.40e-11 | 2.50e-10 |
| ENSG00000007255 | 5.82e-02 | 9.45e-02 |
| ENSG00000007264 | 1.29e-09 | 9.98e-09 |
| ENSG00000007306 | 2.39e-18 | 1.02e-16 |
| ENSG00000007312 | 4.92e-11 | 4.87e-10 |
| ENSG00000007314 | 6.17e-07 | 3.02e-06 |
| ENSG00000007341 | 9.10e-03 | 1.88e-02 |
| ENSG00000007350 | 7.70e-02 | 1.20e-01 |
| ENSG00000007372 | 2.55e-03 | 6.11e-03 |
| ENSG00000007376 | 1.18e-10 | 1.09e-09 |
| ENSG00000007384 | 5.47e-13 | 7.75e-12 |
| ENSG00000007392 | 2.35e-13 | 3.58e-12 |
| ENSG00000007402 | 1.06e-03 | 2.79e-03 |
| ENSG00000007516 | 1.68e-04 | 5.23e-04 |
| ENSG00000007520 | 2.16e-01 | 2.89e-01 |
| ENSG00000007541 | 2.45e-04 | 7.40e-04 |
| ENSG00000007545 | 2.32e-11 | 2.42e-10 |
| ENSG00000007866 | 2.50e-01 | 3.27e-01 |
| ENSG00000007908 | 6.37e-09 | 4.40e-08 |
| ENSG00000007923 | 4.07e-05 | 1.44e-04 |
| ENSG00000007933 | 1.50e-08 | 9.72e-08 |
| ENSG00000007944 | 9.34e-01 | 9.51e-01 |
| ENSG00000007952 | 2.24e-01 | 2.97e-01 |
| ENSG00000007968 | 1.22e-06 | 5.68e-06 |
| ENSG00000008018 | 2.44e-07 | 1.29e-06 |
| ENSG00000008056 | 5.08e-01 | 5.88e-01 |
| ENSG00000008083 | 1.56e-05 | 5.94e-05 |
| ENSG00000008086 | 2.61e-01 | 3.38e-01 |
| ENSG00000008118 | 4.75e-03 | 1.06e-02 |
| ENSG00000008128 | 5.74e-01 | 6.50e-01 |
| ENSG00000008130 | 2.11e-03 | 5.16e-03 |
| ENSG00000008196 | 2.68e-06 | 1.17e-05 |
| ENSG00000008226 | 7.65e-04 | 2.08e-03 |
| ENSG00000008256 | 1.70e-01 | 2.36e-01 |
| ENSG00000008277 | 3.80e-04 | 1.10e-03 |
| ENSG00000008282 | 4.85e-04 | 1.37e-03 |
| ENSG00000008283 | 1.96e-04 | 6.04e-04 |
| ENSG00000008294 | 1.02e-02 | 2.08e-02 |
| ENSG00000008300 | 9.34e-19 | 4.44e-17 |
| ENSG00000008311 | 3.90e-01 | 4.74e-01 |
| ENSG00000008323 | 6.92e-08 | 4.01e-07 |
| ENSG00000008324 | 8.98e-05 | 2.95e-04 |
| ENSG00000008382 | 1.28e-11 | 1.39e-10 |
| ENSG00000008394 | 4.60e-05 | 1.60e-04 |
| ENSG00000008405 | 9.72e-02 | 1.47e-01 |
| ENSG00000008438 | 5.87e-01 | 6.61e-01 |
| ENSG00000008441 | 6.42e-02 | 1.03e-01 |
| ENSG00000008513 | 1.53e-04 | 4.80e-04 |
| ENSG00000008516 | 1.04e-05 | 4.11e-05 |
| ENSG00000008517 | 1.22e-01 | 1.78e-01 |
| ENSG00000008710 | 9.52e-01 | 9.65e-01 |
| ENSG00000008735 | 1.07e-03 | 2.81e-03 |
| ENSG00000008838 | 1.65e-05 | 6.27e-05 |
| ENSG00000008853 | 8.77e-04 | 2.35e-03 |
| ENSG00000008869 | 3.14e-02 | 5.54e-02 |
| ENSG00000008952 | 4.92e-03 | 1.09e-02 |
| ENSG00000008988 | 1.75e-02 | 3.32e-02 |
| ENSG00000009307 | 4.57e-06 | 1.92e-05 |
| ENSG00000009335 | 5.97e-10 | 4.89e-09 |
| ENSG00000009413 | 2.67e-03 | 6.36e-03 |
| ENSG00000009694 | 5.62e-10 | 4.63e-09 |
| ENSG00000009709 | 7.88e-03 | 1.66e-02 |
| ENSG00000009724 | 5.54e-03 | 1.21e-02 |
| ENSG00000009765 | 9.98e-01 | 9.98e-01 |
| ENSG00000009780 | 7.01e-02 | 1.11e-01 |
| ENSG00000009790 | 3.15e-07 | 1.63e-06 |
| ENSG00000009830 | 2.79e-04 | 8.30e-04 |
| ENSG00000009844 | 8.53e-03 | 1.77e-02 |
| ENSG00000009950 | 3.70e-16 | 9.75e-15 |
| ENSG00000009954 | 1.16e-08 | 7.64e-08 |
| ENSG00000010017 | 5.04e-02 | 8.34e-02 |
| ENSG00000010030 | 4.34e-09 | 3.08e-08 |
| ENSG00000010072 | 1.97e-06 | 8.80e-06 |
| ENSG00000010165 | 1.73e-08 | 1.11e-07 |
| ENSG00000010219 | 3.57e-04 | 1.04e-03 |
| ENSG00000010244 | 1.39e-13 | 2.19e-12 |
| ENSG00000010256 | 8.88e-05 | 2.92e-04 |
| ENSG00000010270 | 1.49e-03 | 3.78e-03 |
| ENSG00000010278 | 6.00e-01 | 6.73e-01 |
| ENSG00000010282 | 1.47e-08 | 9.53e-08 |
| ENSG00000010292 | 8.44e-10 | 6.75e-09 |
| ENSG00000010295 | 1.50e-02 | 2.91e-02 |
| ENSG00000010310 | 7.35e-02 | 1.16e-01 |
| ENSG00000010318 | 8.50e-13 | 1.16e-11 |
| ENSG00000010319 | 2.67e-17 | 8.98e-16 |
| ENSG00000010322 | 2.28e-01 | 3.01e-01 |
| ENSG00000010327 | 2.78e-10 | 2.42e-09 |
| ENSG00000010361 | 8.05e-03 | 1.69e-02 |
| ENSG00000010379 | 1.75e-01 | 2.41e-01 |
| ENSG00000010404 | 4.59e-05 | 1.60e-04 |
| ENSG00000010438 | 3.42e-03 | 7.94e-03 |
| ENSG00000010539 | 6.25e-17 | 1.93e-15 |
| ENSG00000010610 | 2.24e-07 | 1.18e-06 |
| ENSG00000010626 | 3.07e-05 | 1.11e-04 |
| ENSG00000010671 | 2.44e-10 | 2.15e-09 |
| ENSG00000010704 | 8.17e-01 | 8.60e-01 |
| ENSG00000010803 | 1.88e-01 | 2.57e-01 |
| ENSG00000010810 | 1.74e-02 | 3.30e-02 |
| ENSG00000010818 | 4.05e-01 | 4.88e-01 |
| ENSG00000010932 | 1.16e-04 | 3.75e-04 |
| ENSG00000011007 | 6.03e-03 | 1.31e-02 |
| ENSG00000011009 | 4.62e-01 | 5.45e-01 |
| ENSG00000011021 | 1.16e-01 | 1.70e-01 |
| ENSG00000011028 | 2.58e-01 | 3.36e-01 |
| ENSG00000011052 | 1.87e-05 | 7.02e-05 |
| ENSG00000011083 | 6.57e-02 | 1.05e-01 |
| ENSG00000011105 | 1.97e-06 | 8.81e-06 |
| ENSG00000011114 | 1.01e-05 | 3.98e-05 |
| ENSG00000011132 | 1.20e-05 | 4.67e-05 |
| ENSG00000011143 | 3.95e-11 | 3.97e-10 |
| ENSG00000011198 | 7.62e-15 | 1.53e-13 |
| ENSG00000011201 | 2.82e-19 | 1.52e-17 |
| ENSG00000011243 | 6.48e-10 | 5.28e-09 |
| ENSG00000011258 | 1.00e+00 | 1.00e+00 |
| ENSG00000011260 | 8.72e-12 | 9.85e-11 |
| ENSG00000011275 | 3.87e-07 | 1.96e-06 |
| ENSG00000011295 | 2.91e-03 | 6.87e-03 |
| ENSG00000011304 | 1.06e-11 | 1.18e-10 |
| ENSG00000011332 | 7.19e-10 | 5.82e-09 |
| ENSG00000011347 | 1.29e-15 | 3.02e-14 |
| ENSG00000011376 | 3.81e-05 | 1.35e-04 |
| ENSG00000011405 | 2.19e-01 | 2.92e-01 |
| ENSG00000011422 | 2.99e-08 | 1.84e-07 |
| ENSG00000011426 | 1.86e-19 | 1.06e-17 |
| ENSG00000011451 | 1.85e-08 | 1.18e-07 |
| ENSG00000011454 | 2.11e-02 | 3.92e-02 |
| ENSG00000011465 | 1.50e-12 | 1.97e-11 |
| ENSG00000011478 | 4.52e-07 | 2.27e-06 |
| ENSG00000011485 | 1.26e-05 | 4.91e-05 |
| ENSG00000011523 | 4.79e-01 | 5.61e-01 |
| ENSG00000011566 | 1.77e-03 | 4.40e-03 |
| ENSG00000011590 | 5.86e-01 | 6.61e-01 |
| ENSG00000011600 | 6.01e-02 | 9.71e-02 |
| ENSG00000011638 | 1.21e-01 | 1.76e-01 |
| ENSG00000011677 | 2.74e-02 | 4.93e-02 |
| ENSG00000012048 | 2.88e-14 | 5.16e-13 |
| ENSG00000012061 | 4.00e-04 | 1.15e-03 |
| ENSG00000012124 | 1.66e-10 | 1.50e-09 |
| ENSG00000012171 | 1.39e-07 | 7.66e-07 |
| ENSG00000012174 | 1.03e-12 | 1.39e-11 |
| ENSG00000012211 | 2.82e-12 | 3.51e-11 |
| ENSG00000012223 | 7.97e-03 | 1.67e-02 |
| ENSG00000012232 | 1.05e-04 | 3.39e-04 |
| ENSG00000012504 | 4.61e-16 | 1.19e-14 |
| ENSG00000012660 | 3.53e-10 | 3.02e-09 |
| ENSG00000012779 | 5.54e-01 | 6.31e-01 |
| ENSG00000012817 | 2.06e-02 | 3.83e-02 |
| ENSG00000012822 | 1.55e-01 | 2.18e-01 |
| ENSG00000012963 | 1.64e-06 | 7.43e-06 |
| ENSG00000012983 | 7.44e-01 | 8.00e-01 |
| ENSG00000013016 | 5.82e-03 | 1.27e-02 |
| ENSG00000013275 | 3.31e-04 | 9.70e-04 |
| ENSG00000013288 | 2.40e-05 | 8.86e-05 |
| ENSG00000013293 | 3.99e-18 | 1.61e-16 |
| ENSG00000013297 | 4.99e-15 | 1.03e-13 |
| ENSG00000013306 | 3.73e-07 | 1.90e-06 |
| ENSG00000013364 | 1.25e-06 | 5.80e-06 |
| ENSG00000013374 | 6.37e-01 | 7.07e-01 |
| ENSG00000013375 | 4.05e-07 | 2.05e-06 |
| ENSG00000013392 | 1.17e-04 | 3.76e-04 |
| ENSG00000013441 | 1.41e-03 | 3.59e-03 |
| ENSG00000013503 | 2.09e-03 | 5.11e-03 |
| ENSG00000013523 | 1.95e-16 | 5.48e-15 |
| ENSG00000013561 | 1.25e-09 | 9.74e-09 |
| ENSG00000013563 | 4.76e-06 | 1.99e-05 |
| ENSG00000013573 | 1.20e-17 | 4.33e-16 |
| ENSG00000013583 | 1.22e-01 | 1.78e-01 |
| ENSG00000013588 | 6.49e-03 | 1.39e-02 |
| ENSG00000013619 | 7.79e-02 | 1.21e-01 |
| ENSG00000013725 | 2.50e-04 | 7.52e-04 |
| ENSG00000013810 | 5.72e-10 | 4.71e-09 |
| ENSG00000014123 | 8.95e-02 | 1.37e-01 |
| ENSG00000014138 | 7.17e-15 | 1.44e-13 |
| ENSG00000014164 | 1.51e-05 | 5.77e-05 |
| ENSG00000014216 | 6.15e-01 | 6.87e-01 |
| ENSG00000014257 | 3.59e-02 | 6.22e-02 |
| ENSG00000014641 | 5.56e-08 | 3.28e-07 |
| ENSG00000014824 | 1.95e-02 | 3.66e-02 |
| ENSG00000014914 | 5.38e-05 | 1.85e-04 |
| ENSG00000014919 | 1.37e-01 | 1.97e-01 |
| ENSG00000015133 | 2.18e-03 | 5.31e-03 |
| ENSG00000015153 | 5.54e-06 | 2.29e-05 |
| ENSG00000015171 | 1.13e-01 | 1.67e-01 |
| ENSG00000015285 | 1.44e-07 | 7.92e-07 |
| ENSG00000015413 | 3.11e-17 | 1.03e-15 |
| ENSG00000015475 | 2.26e-16 | 6.25e-15 |
| ENSG00000015479 | 9.40e-02 | 1.43e-01 |
| ENSG00000015520 | 2.86e-07 | 1.49e-06 |
| ENSG00000015532 | 7.43e-03 | 1.57e-02 |
| ENSG00000015568 | 8.34e-01 | 8.74e-01 |
| ENSG00000015592 | 1.50e-24 | 4.15e-22 |
| ENSG00000015676 | 5.99e-12 | 6.99e-11 |
| ENSG00000016082 | 1.25e-03 | 3.24e-03 |
| ENSG00000016391 | 6.02e-04 | 1.67e-03 |
| ENSG00000016402 | 1.18e-11 | 1.29e-10 |
| ENSG00000016490 | 1.87e-11 | 1.99e-10 |
| ENSG00000016602 | 2.34e-20 | 1.70e-18 |
| ENSG00000016864 | 1.51e-04 | 4.77e-04 |
| ENSG00000017260 | 3.07e-07 | 1.59e-06 |
| ENSG00000017427 | 1.13e-11 | 1.25e-10 |
| ENSG00000017483 | 1.17e-11 | 1.28e-10 |
| ENSG00000017797 | 7.27e-08 | 4.20e-07 |
| ENSG00000018189 | 6.00e-02 | 9.71e-02 |
| ENSG00000018236 | 1.03e-19 | 6.21e-18 |
| ENSG00000018280 | 9.63e-19 | 4.56e-17 |
| ENSG00000018408 | 9.91e-04 | 2.62e-03 |
| ENSG00000018510 | 3.09e-01 | 3.90e-01 |
| ENSG00000018607 | 3.58e-01 | 4.40e-01 |
| ENSG00000018610 | 7.02e-04 | 1.92e-03 |
| ENSG00000018625 | 6.77e-20 | 4.26e-18 |
| ENSG00000018699 | 6.16e-09 | 4.26e-08 |
| ENSG00000018869 | 5.69e-10 | 4.69e-09 |
| ENSG00000019102 | 3.91e-14 | 6.84e-13 |
| ENSG00000019144 | 5.41e-07 | 2.67e-06 |
| ENSG00000019169 | 3.05e-01 | 3.86e-01 |
| ENSG00000019186 | 1.03e-07 | 5.80e-07 |
| ENSG00000019485 | 7.94e-01 | 8.41e-01 |
| ENSG00000019505 | 5.82e-04 | 1.62e-03 |
| ENSG00000019549 | 9.20e-02 | 1.40e-01 |
| ENSG00000019582 | 2.28e-04 | 6.93e-04 |
| ENSG00000019991 | 2.79e-01 | 3.58e-01 |
| ENSG00000019995 | 1.77e-01 | 2.44e-01 |
| ENSG00000020129 | 1.86e-07 | 1.00e-06 |
| ENSG00000020181 | 4.06e-01 | 4.89e-01 |
| ENSG00000020219 | 4.65e-01 | 5.47e-01 |
| ENSG00000020256 | 5.47e-13 | 7.75e-12 |
| ENSG00000020426 | 5.38e-07 | 2.66e-06 |
| ENSG00000020577 | 4.39e-04 | 1.25e-03 |
| ENSG00000020633 | 9.65e-02 | 1.46e-01 |
| ENSG00000020922 | 8.93e-09 | 6.00e-08 |
| ENSG00000021300 | 2.29e-08 | 1.44e-07 |
| ENSG00000021355 | 7.14e-02 | 1.13e-01 |
| ENSG00000021461 | 7.96e-02 | 1.24e-01 |
| ENSG00000021488 | 5.88e-01 | 6.63e-01 |
| ENSG00000021574 | 2.20e-01 | 2.93e-01 |
| ENSG00000021645 | 7.47e-02 | 1.17e-01 |
| ENSG00000021762 | 5.62e-01 | 6.39e-01 |
| ENSG00000021776 | 5.05e-01 | 5.85e-01 |
| ENSG00000021826 | 3.14e-02 | 5.54e-02 |
| ENSG00000021852 | 5.92e-01 | 6.66e-01 |
| ENSG00000022267 | 4.76e-18 | 1.89e-16 |
| ENSG00000022277 | 4.17e-04 | 1.20e-03 |
| ENSG00000022355 | 4.79e-06 | 2.00e-05 |
| ENSG00000022556 | 2.15e-09 | 1.61e-08 |
| ENSG00000022567 | 3.47e-02 | 6.04e-02 |
| ENSG00000022840 | 2.65e-07 | 1.39e-06 |
| ENSG00000022976 | 3.63e-01 | 4.46e-01 |
| ENSG00000023041 | 5.71e-05 | 1.95e-04 |
| ENSG00000023171 | 5.56e-01 | 6.33e-01 |
| ENSG00000023191 | 4.38e-02 | 7.40e-02 |
| ENSG00000023228 | 1.61e-10 | 1.45e-09 |
| ENSG00000023287 | 6.59e-01 | 7.26e-01 |
| ENSG00000023318 | 7.83e-01 | 8.32e-01 |
| ENSG00000023330 | 5.66e-07 | 2.79e-06 |
| ENSG00000023445 | 9.48e-05 | 3.10e-04 |
| ENSG00000023516 | 7.31e-05 | 2.45e-04 |
| ENSG00000023572 | 2.03e-02 | 3.78e-02 |
| ENSG00000023608 | 3.04e-04 | 8.99e-04 |
| ENSG00000023697 | 3.78e-08 | 2.29e-07 |
| ENSG00000023734 | 1.01e-10 | 9.44e-10 |
| ENSG00000023839 | 1.25e-09 | 9.69e-09 |
| ENSG00000023892 | 1.54e-03 | 3.88e-03 |
| ENSG00000023902 | 1.85e-07 | 9.96e-07 |
| ENSG00000023909 | 6.61e-02 | 1.05e-01 |
| ENSG00000024048 | 1.83e-04 | 5.68e-04 |
| ENSG00000024422 | 8.53e-04 | 2.29e-03 |
| ENSG00000024526 | 9.83e-05 | 3.20e-04 |
| ENSG00000024862 | 3.86e-05 | 1.37e-04 |
| ENSG00000025039 | 2.32e-05 | 8.57e-05 |
| ENSG00000025156 | 8.18e-05 | 2.71e-04 |
| ENSG00000025293 | 9.24e-12 | 1.04e-10 |
| ENSG00000025423 | 2.34e-08 | 1.47e-07 |
| ENSG00000025434 | 6.03e-04 | 1.68e-03 |
| ENSG00000025708 | 1.27e-03 | 3.27e-03 |
| ENSG00000025770 | 4.69e-03 | 1.05e-02 |
| ENSG00000025772 | 8.78e-22 | 1.00e-19 |
| ENSG00000025796 | 8.42e-02 | 1.30e-01 |
| ENSG00000025800 | 1.56e-01 | 2.20e-01 |
| ENSG00000026025 | 1.44e-05 | 5.52e-05 |
| ENSG00000026036 | 2.81e-12 | 3.49e-11 |
| ENSG00000026103 | 9.39e-13 | 1.28e-11 |
| ENSG00000026297 | 3.06e-04 | 9.05e-04 |
| ENSG00000026508 | 1.41e-17 | 5.01e-16 |
| ENSG00000026559 | 8.35e-12 | 9.47e-11 |
| ENSG00000026652 | 1.44e-01 | 2.05e-01 |
| ENSG00000026751 | 8.52e-13 | 1.17e-11 |
| ENSG00000026950 | 6.26e-01 | 6.96e-01 |
| ENSG00000027001 | 1.54e-09 | 1.18e-08 |
| ENSG00000027075 | 2.15e-02 | 3.99e-02 |
| ENSG00000027644 | 7.72e-01 | 8.23e-01 |
| ENSG00000027697 | 1.15e-04 | 3.70e-04 |
| ENSG00000027847 | 6.37e-12 | 7.38e-11 |
| ENSG00000027869 | 3.18e-07 | 1.64e-06 |
| ENSG00000028116 | 6.69e-04 | 1.84e-03 |
| ENSG00000028137 | 1.17e-01 | 1.72e-01 |
| ENSG00000028203 | 8.71e-01 | 9.04e-01 |
| ENSG00000028277 | 9.25e-10 | 7.35e-09 |
| ENSG00000028310 | 2.89e-13 | 4.32e-12 |
| ENSG00000028528 | 1.01e-10 | 9.40e-10 |
| ENSG00000028839 | 2.72e-03 | 6.48e-03 |
| ENSG00000029153 | 2.25e-24 | 5.78e-22 |
| ENSG00000029363 | 2.79e-01 | 3.58e-01 |
| ENSG00000029364 | 9.16e-01 | 9.37e-01 |
| ENSG00000029534 | 4.90e-01 | 5.72e-01 |
| ENSG00000029559 | 2.55e-21 | 2.53e-19 |
| ENSG00000029639 | 8.85e-14 | 1.45e-12 |
| ENSG00000029725 | 2.32e-04 | 7.04e-04 |
| ENSG00000029993 | 3.91e-08 | 2.36e-07 |
| ENSG00000030066 | 2.13e-12 | 2.71e-11 |
| ENSG00000030110 | 3.08e-08 | 1.89e-07 |
| ENSG00000030304 | 5.08e-22 | 6.30e-20 |
| ENSG00000030419 | 3.67e-04 | 1.07e-03 |
| ENSG00000030582 | 2.12e-03 | 5.18e-03 |
| ENSG00000031003 | 6.32e-10 | 5.15e-09 |
| ENSG00000031081 | 1.61e-03 | 4.06e-03 |
| ENSG00000031691 | 2.05e-06 | 9.14e-06 |
| ENSG00000031698 | 4.31e-01 | 5.15e-01 |
| ENSG00000031823 | 7.36e-05 | 2.46e-04 |
| ENSG00000032219 | 3.08e-04 | 9.10e-04 |
| ENSG00000032389 | 2.98e-11 | 3.06e-10 |
| ENSG00000032444 | 7.18e-01 | 7.77e-01 |
| ENSG00000032742 | 2.03e-04 | 6.22e-04 |
| ENSG00000033011 | 5.93e-10 | 4.86e-09 |
| ENSG00000033030 | 9.02e-01 | 9.27e-01 |
| ENSG00000033050 | 3.19e-10 | 2.74e-09 |
| ENSG00000033100 | 4.65e-05 | 1.62e-04 |
| ENSG00000033122 | 1.23e-10 | 1.14e-09 |
| ENSG00000033170 | 2.34e-09 | 1.74e-08 |
| ENSG00000033178 | 1.50e-05 | 5.74e-05 |
| ENSG00000033327 | 2.08e-04 | 6.37e-04 |
| ENSG00000033627 | 9.03e-02 | 1.38e-01 |
| ENSG00000033800 | 1.52e-03 | 3.86e-03 |
| ENSG00000033867 | 8.27e-02 | 1.28e-01 |
| ENSG00000034053 | 5.53e-04 | 1.55e-03 |
| ENSG00000034152 | 2.80e-01 | 3.59e-01 |
| ENSG00000034239 | 2.91e-10 | 2.52e-09 |
| ENSG00000034510 | 8.91e-05 | 2.93e-04 |
| ENSG00000034533 | 1.04e-04 | 3.36e-04 |
| ENSG00000034677 | 3.23e-04 | 9.50e-04 |
| ENSG00000034693 | 1.19e-02 | 2.37e-02 |
| ENSG00000034713 | 7.04e-04 | 1.93e-03 |
| ENSG00000034971 | 3.67e-25 | 1.28e-22 |
| ENSG00000035115 | 1.71e-03 | 4.27e-03 |
| ENSG00000035141 | 5.34e-10 | 4.42e-09 |
| ENSG00000035403 | 1.36e-03 | 3.48e-03 |
| ENSG00000035499 | 5.23e-12 | 6.15e-11 |
| ENSG00000035664 | 1.65e-03 | 4.14e-03 |
| ENSG00000035681 | 2.47e-08 | 1.55e-07 |
| ENSG00000035687 | 8.71e-03 | 1.81e-02 |
| ENSG00000035720 | 1.26e-12 | 1.67e-11 |
| ENSG00000035862 | 1.91e-03 | 4.72e-03 |
| ENSG00000035928 | 2.98e-01 | 3.78e-01 |
| ENSG00000036054 | 4.34e-01 | 5.17e-01 |
| ENSG00000036257 | 8.69e-01 | 9.02e-01 |
| ENSG00000036448 | 6.19e-03 | 1.34e-02 |
| ENSG00000036473 | 1.79e-07 | 9.68e-07 |
| ENSG00000036530 | 7.69e-06 | 3.11e-05 |
| ENSG00000036549 | 4.20e-06 | 1.77e-05 |
| ENSG00000036565 | 1.77e-02 | 3.36e-02 |
| ENSG00000036672 | 2.09e-25 | 7.97e-23 |
| ENSG00000036828 | 1.92e-13 | 2.96e-12 |
| ENSG00000037042 | 3.42e-01 | 4.24e-01 |
| ENSG00000037241 | 1.72e-08 | 1.11e-07 |
| ENSG00000037280 | 6.87e-01 | 7.51e-01 |
| ENSG00000037474 | 9.66e-13 | 1.31e-11 |
| ENSG00000037637 | 4.93e-01 | 5.74e-01 |
| ENSG00000037749 | 2.96e-01 | 3.76e-01 |
| ENSG00000037757 | 5.48e-14 | 9.32e-13 |
| ENSG00000037897 | 8.20e-21 | 6.92e-19 |
| ENSG00000037965 | 1.92e-03 | 4.73e-03 |
| ENSG00000038002 | 3.06e-02 | 5.40e-02 |
| ENSG00000038210 | 9.79e-02 | 1.48e-01 |
| ENSG00000038219 | 1.00e-01 | 1.51e-01 |
| ENSG00000038274 | 2.04e-04 | 6.25e-04 |
| ENSG00000038295 | 6.31e-15 | 1.28e-13 |
| ENSG00000038358 | 7.75e-01 | 8.25e-01 |
| ENSG00000038382 | 5.65e-03 | 1.23e-02 |
| ENSG00000038427 | 2.85e-10 | 2.47e-09 |
| ENSG00000038532 | 6.06e-01 | 6.79e-01 |
| ENSG00000038945 | 7.81e-02 | 1.22e-01 |
| ENSG00000039068 | 1.75e-01 | 2.42e-01 |
| ENSG00000039123 | 1.34e-02 | 2.63e-02 |
| ENSG00000039139 | 2.66e-11 | 2.75e-10 |
| ENSG00000039319 | 9.63e-01 | 9.72e-01 |
| ENSG00000039523 | 7.38e-03 | 1.56e-02 |
| ENSG00000039537 | 2.80e-09 | 2.05e-08 |
| ENSG00000039560 | 3.18e-02 | 5.60e-02 |
| ENSG00000039600 | 1.35e-01 | 1.94e-01 |
| ENSG00000039650 | 9.21e-07 | 4.39e-06 |
| ENSG00000039987 | 1.61e-14 | 3.04e-13 |
| ENSG00000040199 | 2.08e-21 | 2.12e-19 |
| ENSG00000040275 | 4.47e-13 | 6.46e-12 |
| ENSG00000040341 | 6.79e-01 | 7.44e-01 |
| ENSG00000040487 | 1.99e-01 | 2.70e-01 |
| ENSG00000040531 | 1.20e-01 | 1.76e-01 |
| ENSG00000040608 | 3.59e-08 | 2.18e-07 |
| ENSG00000040633 | 4.55e-03 | 1.02e-02 |
| ENSG00000040731 | 7.70e-18 | 2.91e-16 |
| ENSG00000040933 | 2.65e-02 | 4.78e-02 |
| ENSG00000041353 | 2.70e-04 | 8.07e-04 |
| ENSG00000041357 | 9.40e-01 | 9.55e-01 |
| ENSG00000041515 | 3.39e-01 | 4.21e-01 |
| ENSG00000041802 | 5.85e-12 | 6.84e-11 |
| ENSG00000041880 | 5.34e-01 | 6.13e-01 |
| ENSG00000041982 | 6.79e-03 | 1.45e-02 |
| ENSG00000041988 | 6.24e-01 | 6.95e-01 |
| ENSG00000042062 | 2.41e-02 | 4.39e-02 |
| ENSG00000042088 | 5.37e-12 | 6.32e-11 |
| ENSG00000042286 | 4.66e-01 | 5.49e-01 |
| ENSG00000042304 | 1.28e-02 | 2.52e-02 |
| ENSG00000042317 | 2.24e-02 | 4.13e-02 |
| ENSG00000042429 | 6.39e-09 | 4.41e-08 |
| ENSG00000042445 | 7.39e-23 | 1.17e-20 |
| ENSG00000042493 | 2.31e-06 | 1.02e-05 |
| ENSG00000042753 | 1.68e-02 | 3.20e-02 |
| ENSG00000042781 | 4.93e-03 | 1.09e-02 |
| ENSG00000042813 | 2.43e-06 | 1.07e-05 |
| ENSG00000042832 | 1.22e-18 | 5.60e-17 |
| ENSG00000042980 | 2.71e-12 | 3.38e-11 |
| ENSG00000043039 | 1.07e-10 | 9.90e-10 |
| ENSG00000043093 | 3.90e-01 | 4.73e-01 |
| ENSG00000043143 | 1.01e-01 | 1.52e-01 |
| ENSG00000043355 | 7.19e-11 | 6.91e-10 |
| ENSG00000043462 | 3.01e-01 | 3.81e-01 |
| ENSG00000043514 | 5.48e-11 | 5.37e-10 |
| ENSG00000043591 | 3.25e-12 | 3.99e-11 |
| ENSG00000044012 | 1.85e-25 | 7.53e-23 |
| ENSG00000044090 | 5.80e-12 | 6.78e-11 |
| ENSG00000044115 | 1.08e-02 | 2.18e-02 |
| ENSG00000044446 | 3.57e-02 | 6.19e-02 |
| ENSG00000044459 | 1.04e-01 | 1.55e-01 |
| ENSG00000044524 | 1.68e-07 | 9.10e-07 |
| ENSG00000044574 | 3.46e-06 | 1.48e-05 |
| ENSG00000046604 | 2.55e-05 | 9.37e-05 |
| ENSG00000046647 | 5.32e-11 | 5.22e-10 |
| ENSG00000046651 | 4.76e-03 | 1.06e-02 |
| ENSG00000046653 | 5.78e-14 | 9.78e-13 |
| ENSG00000046774 | 1.47e-01 | 2.09e-01 |
| ENSG00000046889 | 3.74e-10 | 3.18e-09 |
| ENSG00000047056 | 6.90e-06 | 2.80e-05 |
| ENSG00000047188 | 1.09e-01 | 1.62e-01 |
| ENSG00000047230 | 1.87e-13 | 2.89e-12 |
| ENSG00000047249 | 1.18e-04 | 3.79e-04 |
| ENSG00000047315 | 1.34e-02 | 2.63e-02 |
| ENSG00000047346 | 5.22e-13 | 7.43e-12 |
| ENSG00000047365 | 5.49e-01 | 6.27e-01 |
| ENSG00000047410 | 1.31e-05 | 5.09e-05 |
| ENSG00000047457 | 1.09e-17 | 4.00e-16 |
| ENSG00000047578 | 9.61e-01 | 9.71e-01 |
| ENSG00000047579 | 6.09e-02 | 9.82e-02 |
| ENSG00000047597 | 5.85e-10 | 4.80e-09 |
| ENSG00000047617 | 8.90e-04 | 2.38e-03 |
| ENSG00000047621 | 7.03e-01 | 7.65e-01 |
| ENSG00000047634 | 2.95e-15 | 6.42e-14 |
| ENSG00000047644 | 5.40e-05 | 1.86e-04 |
| ENSG00000047648 | 4.47e-03 | 1.00e-02 |
| ENSG00000047662 | 8.25e-07 | 3.96e-06 |
| ENSG00000047849 | 2.42e-01 | 3.17e-01 |
| ENSG00000047932 | 9.60e-02 | 1.45e-01 |
| ENSG00000047936 | 3.86e-10 | 3.27e-09 |
| ENSG00000048028 | 1.97e-01 | 2.67e-01 |
| ENSG00000048052 | 2.41e-16 | 6.63e-15 |
| ENSG00000048140 | 2.40e-01 | 3.16e-01 |
| ENSG00000048162 | 8.44e-19 | 4.08e-17 |
| ENSG00000048342 | 3.32e-04 | 9.74e-04 |
| ENSG00000048392 | 2.46e-01 | 3.22e-01 |
| ENSG00000048405 | 3.06e-03 | 7.17e-03 |
| ENSG00000048462 | 8.12e-18 | 3.06e-16 |
| ENSG00000048471 | 2.14e-04 | 6.53e-04 |
| ENSG00000048540 | 4.84e-11 | 4.79e-10 |
| ENSG00000048544 | 2.88e-03 | 6.80e-03 |
| ENSG00000048545 | 1.71e-04 | 5.33e-04 |
| ENSG00000048649 | 8.57e-01 | 8.92e-01 |
| ENSG00000048707 | 3.59e-09 | 2.58e-08 |
| ENSG00000048740 | 1.86e-03 | 4.60e-03 |
| ENSG00000048828 | 5.66e-01 | 6.42e-01 |
| ENSG00000048991 | 2.54e-15 | 5.60e-14 |
| ENSG00000049089 | 1.08e-01 | 1.61e-01 |
| ENSG00000049130 | 1.16e-10 | 1.07e-09 |
| ENSG00000049167 | 8.74e-05 | 2.88e-04 |
| ENSG00000049192 | 1.76e-17 | 6.18e-16 |
| ENSG00000049239 | 7.85e-01 | 8.33e-01 |
| ENSG00000049245 | 1.19e-02 | 2.38e-02 |
| ENSG00000049246 | 2.03e-18 | 8.82e-17 |
| ENSG00000049247 | 1.29e-01 | 1.87e-01 |
| ENSG00000049249 | 1.53e-06 | 6.96e-06 |
| ENSG00000049283 | 1.59e-03 | 4.00e-03 |
| ENSG00000049323 | 1.43e-01 | 2.03e-01 |
| ENSG00000049449 | 1.79e-23 | 3.44e-21 |
| ENSG00000049540 | 1.89e-05 | 7.09e-05 |
| ENSG00000049541 | 3.03e-13 | 4.51e-12 |
| ENSG00000049618 | 2.07e-03 | 5.08e-03 |
| ENSG00000049656 | 3.65e-03 | 8.39e-03 |
| ENSG00000049759 | 4.37e-17 | 1.41e-15 |
| ENSG00000049768 | 2.60e-12 | 3.26e-11 |
| ENSG00000049769 | 5.95e-01 | 6.69e-01 |
| ENSG00000049860 | 4.81e-02 | 8.01e-02 |
| ENSG00000049883 | 4.41e-11 | 4.40e-10 |
| ENSG00000050030 | 5.14e-18 | 2.02e-16 |
| ENSG00000050130 | 6.90e-03 | 1.47e-02 |
| ENSG00000050165 | 6.97e-03 | 1.48e-02 |
| ENSG00000050327 | 9.74e-02 | 1.47e-01 |
| ENSG00000050344 | 9.91e-26 | 4.46e-23 |
| ENSG00000050393 | 9.35e-02 | 1.42e-01 |
| ENSG00000050405 | 9.75e-17 | 2.91e-15 |
| ENSG00000050426 | 1.67e-06 | 7.56e-06 |
| ENSG00000050438 | 4.38e-07 | 2.20e-06 |
| ENSG00000050555 | 2.79e-05 | 1.02e-04 |
| ENSG00000050628 | 5.02e-04 | 1.42e-03 |
| ENSG00000050730 | 2.47e-01 | 3.24e-01 |
| ENSG00000050748 | 3.08e-01 | 3.88e-01 |
| ENSG00000050767 | 3.31e-05 | 1.19e-04 |
| ENSG00000050820 | 1.49e-02 | 2.88e-02 |
| ENSG00000051009 | 5.67e-05 | 1.94e-04 |
| ENSG00000051108 | 1.75e-10 | 1.58e-09 |
| ENSG00000051128 | 2.13e-01 | 2.85e-01 |
| ENSG00000051180 | 2.60e-07 | 1.37e-06 |
| ENSG00000051341 | 1.14e-11 | 1.25e-10 |
| ENSG00000051382 | 1.99e-02 | 3.73e-02 |
| ENSG00000051523 | 1.61e-01 | 2.25e-01 |
| ENSG00000051596 | 7.43e-13 | 1.03e-11 |
| ENSG00000051620 | 2.26e-02 | 4.16e-02 |
| ENSG00000051825 | 1.86e-03 | 4.61e-03 |
| ENSG00000052126 | 1.99e-02 | 3.72e-02 |
| ENSG00000052344 | 2.10e-06 | 9.33e-06 |
| ENSG00000052723 | 6.80e-01 | 7.45e-01 |
| ENSG00000052749 | 2.74e-20 | 1.95e-18 |
| ENSG00000052795 | 7.24e-13 | 1.00e-11 |
| ENSG00000052802 | 1.49e-03 | 3.79e-03 |
| ENSG00000052841 | 1.36e-04 | 4.32e-04 |
| ENSG00000052850 | 1.41e-01 | 2.01e-01 |
| ENSG00000053108 | 5.19e-01 | 5.98e-01 |
| ENSG00000053254 | 2.52e-14 | 4.58e-13 |
| ENSG00000053328 | 4.05e-11 | 4.06e-10 |
| ENSG00000053371 | 4.12e-04 | 1.18e-03 |
| ENSG00000053372 | 4.73e-14 | 8.14e-13 |
| ENSG00000053438 | 3.29e-01 | 4.11e-01 |
| ENSG00000053501 | 1.00e-02 | 2.04e-02 |
| ENSG00000053524 | 3.92e-09 | 2.80e-08 |
| ENSG00000053702 | 6.55e-04 | 1.81e-03 |
| ENSG00000053747 | 2.72e-04 | 8.11e-04 |
| ENSG00000053770 | 2.08e-11 | 2.19e-10 |
| ENSG00000053900 | 1.63e-01 | 2.27e-01 |
| ENSG00000053918 | 1.39e-04 | 4.40e-04 |
| ENSG00000054116 | 3.12e-01 | 3.93e-01 |
| ENSG00000054118 | 9.34e-01 | 9.51e-01 |
| ENSG00000054148 | 5.09e-01 | 5.89e-01 |
| ENSG00000054179 | 5.52e-03 | 1.21e-02 |
| ENSG00000054219 | 3.29e-02 | 5.76e-02 |
| ENSG00000054267 | 5.00e-01 | 5.80e-01 |
| ENSG00000054277 | 8.90e-15 | 1.76e-13 |
| ENSG00000054282 | 4.77e-05 | 1.66e-04 |
| ENSG00000054356 | 6.49e-09 | 4.47e-08 |
| ENSG00000054392 | 1.14e-08 | 7.50e-08 |
| ENSG00000054523 | 8.35e-05 | 2.76e-04 |
| ENSG00000054598 | 1.84e-04 | 5.69e-04 |
| ENSG00000054611 | 3.07e-06 | 1.33e-05 |
| ENSG00000054654 | 8.30e-05 | 2.75e-04 |
| ENSG00000054690 | 1.46e-01 | 2.08e-01 |
| ENSG00000054793 | 2.98e-02 | 5.28e-02 |
| ENSG00000054803 | 5.91e-02 | 9.58e-02 |
| ENSG00000054938 | 8.61e-03 | 1.79e-02 |
| ENSG00000054965 | 1.15e-01 | 1.70e-01 |
| ENSG00000054967 | 3.13e-14 | 5.57e-13 |
| ENSG00000054983 | 6.47e-04 | 1.79e-03 |
| ENSG00000055044 | 8.42e-18 | 3.16e-16 |
| ENSG00000055070 | 7.33e-02 | 1.15e-01 |
| ENSG00000055118 | 6.47e-01 | 7.16e-01 |
| ENSG00000055130 | 4.03e-07 | 2.04e-06 |
| ENSG00000055147 | 1.26e-02 | 2.50e-02 |
| ENSG00000055163 | 8.28e-01 | 8.69e-01 |
| ENSG00000055208 | 3.84e-08 | 2.32e-07 |
| ENSG00000055211 | 1.67e-13 | 2.60e-12 |
| ENSG00000055332 | 1.89e-04 | 5.82e-04 |
| ENSG00000055483 | 1.33e-15 | 3.09e-14 |
| ENSG00000055609 | 5.53e-01 | 6.30e-01 |
| ENSG00000055732 | 3.07e-01 | 3.88e-01 |
| ENSG00000055813 | 1.95e-08 | 1.24e-07 |
| ENSG00000055917 | 3.38e-01 | 4.20e-01 |
| ENSG00000055950 | 2.09e-01 | 2.81e-01 |
| ENSG00000055955 | 3.33e-02 | 5.82e-02 |
| ENSG00000055957 | 1.52e-01 | 2.15e-01 |
| ENSG00000056050 | 4.43e-02 | 7.46e-02 |
| ENSG00000056097 | 2.85e-06 | 1.24e-05 |
| ENSG00000056277 | 5.19e-16 | 1.33e-14 |
| ENSG00000056291 | 4.35e-02 | 7.34e-02 |
| ENSG00000056487 | 4.56e-07 | 2.29e-06 |
| ENSG00000056558 | 1.02e-05 | 4.04e-05 |
| ENSG00000056586 | 6.45e-03 | 1.39e-02 |
| ENSG00000056736 | 1.34e-04 | 4.25e-04 |
| ENSG00000056972 | 3.22e-13 | 4.76e-12 |
| ENSG00000056998 | 6.10e-03 | 1.32e-02 |
| ENSG00000057019 | 1.88e-02 | 3.54e-02 |
| ENSG00000057149 | 7.20e-06 | 2.92e-05 |
| ENSG00000057252 | 6.74e-04 | 1.85e-03 |
| ENSG00000057294 | 1.16e-07 | 6.45e-07 |
| ENSG00000057468 | 4.23e-03 | 9.56e-03 |
| ENSG00000057593 | 1.27e-08 | 8.29e-08 |
| ENSG00000057608 | 2.35e-01 | 3.10e-01 |
| ENSG00000057657 | 2.18e-03 | 5.32e-03 |
| ENSG00000057663 | 3.23e-04 | 9.49e-04 |
| ENSG00000057704 | 1.03e-23 | 2.22e-21 |
| ENSG00000057757 | 1.44e-02 | 2.81e-02 |
| ENSG00000057935 | 3.47e-01 | 4.30e-01 |
| ENSG00000058056 | 2.00e-02 | 3.74e-02 |
| ENSG00000058063 | 1.71e-02 | 3.26e-02 |
| ENSG00000058085 | 8.39e-07 | 4.03e-06 |
| ENSG00000058091 | 2.44e-03 | 5.88e-03 |
| ENSG00000058262 | 5.62e-08 | 3.31e-07 |
| ENSG00000058272 | 2.74e-03 | 6.52e-03 |
| ENSG00000058335 | 2.35e-01 | 3.10e-01 |
| ENSG00000058404 | 2.65e-08 | 1.65e-07 |
| ENSG00000058453 | 5.15e-07 | 2.56e-06 |
| ENSG00000058600 | 4.91e-18 | 1.94e-16 |
| ENSG00000058668 | 1.07e-06 | 5.04e-06 |
| ENSG00000058673 | 4.28e-02 | 7.25e-02 |
| ENSG00000058729 | 1.93e-01 | 2.63e-01 |
| ENSG00000058799 | 3.45e-04 | 1.01e-03 |
| ENSG00000058804 | 4.55e-06 | 1.91e-05 |
| ENSG00000058866 | 1.38e-01 | 1.98e-01 |
| ENSG00000059122 | 8.33e-04 | 2.24e-03 |
| ENSG00000059145 | 6.99e-09 | 4.79e-08 |
| ENSG00000059377 | 1.06e-05 | 4.19e-05 |
| ENSG00000059378 | 6.09e-03 | 1.32e-02 |
| ENSG00000059573 | 5.17e-01 | 5.97e-01 |
| ENSG00000059588 | 2.12e-17 | 7.35e-16 |
| ENSG00000059691 | 9.98e-04 | 2.64e-03 |
| ENSG00000059728 | 2.70e-16 | 7.36e-15 |
| ENSG00000059758 | 4.25e-01 | 5.08e-01 |
| ENSG00000059769 | 9.18e-02 | 1.40e-01 |
| ENSG00000059804 | 1.34e-04 | 4.26e-04 |
| ENSG00000059915 | 1.53e-08 | 9.90e-08 |
| ENSG00000060069 | 1.99e-05 | 7.47e-05 |
| ENSG00000060138 | 8.87e-12 | 1.00e-10 |
| ENSG00000060140 | 2.65e-13 | 3.98e-12 |
| ENSG00000060237 | 5.47e-03 | 1.20e-02 |
| ENSG00000060303 | 8.06e-02 | 1.25e-01 |
| ENSG00000060339 | 4.35e-06 | 1.83e-05 |
| ENSG00000060491 | 1.28e-09 | 9.94e-09 |
| ENSG00000060558 | 2.91e-07 | 1.51e-06 |
| ENSG00000060566 | 1.24e-13 | 1.97e-12 |
| ENSG00000060642 | 9.38e-04 | 2.49e-03 |
| ENSG00000060656 | 4.09e-05 | 1.44e-04 |
| ENSG00000060688 | 5.39e-09 | 3.77e-08 |
| ENSG00000060709 | 3.04e-03 | 7.15e-03 |
| ENSG00000060718 | 1.66e-30 | 3.47e-27 |
| ENSG00000060749 | 9.66e-03 | 1.98e-02 |
| ENSG00000060762 | 9.09e-18 | 3.39e-16 |
| ENSG00000060971 | 1.11e-13 | 1.79e-12 |
| ENSG00000060982 | 8.28e-01 | 8.69e-01 |
| ENSG00000061273 | 1.16e-11 | 1.28e-10 |
| ENSG00000061337 | 2.14e-17 | 7.41e-16 |
| ENSG00000061455 | 4.08e-07 | 2.06e-06 |
| ENSG00000061656 | 2.71e-03 | 6.46e-03 |
| ENSG00000061676 | 1.61e-01 | 2.25e-01 |
| ENSG00000061794 | 8.90e-06 | 3.55e-05 |
| ENSG00000061918 | 9.40e-01 | 9.56e-01 |
| ENSG00000061936 | 4.63e-09 | 3.27e-08 |
| ENSG00000061938 | 4.78e-07 | 2.39e-06 |
| ENSG00000061987 | 1.33e-04 | 4.24e-04 |
| ENSG00000062038 | 1.31e-31 | 3.22e-28 |
| ENSG00000062096 | 7.43e-02 | 1.17e-01 |
| ENSG00000062194 | 8.83e-01 | 9.12e-01 |
| ENSG00000062282 | 3.38e-21 | 3.18e-19 |
| ENSG00000062370 | 5.13e-01 | 5.92e-01 |
| ENSG00000062485 | 4.61e-02 | 7.72e-02 |
| ENSG00000062524 | 8.23e-10 | 6.59e-09 |
| ENSG00000062582 | 8.80e-02 | 1.35e-01 |
| ENSG00000062598 | 1.05e-08 | 6.99e-08 |
| ENSG00000062650 | 3.45e-02 | 6.02e-02 |
| ENSG00000062716 | 4.13e-03 | 9.35e-03 |
| ENSG00000062725 | 2.02e-03 | 4.96e-03 |
| ENSG00000062822 | 1.60e-09 | 1.22e-08 |
| ENSG00000063015 | 7.46e-10 | 6.01e-09 |
| ENSG00000063046 | 1.85e-04 | 5.73e-04 |
| ENSG00000063127 | 1.62e-08 | 1.04e-07 |
| ENSG00000063169 | 8.95e-01 | 9.22e-01 |
| ENSG00000063176 | 3.90e-01 | 4.74e-01 |
| ENSG00000063177 | 1.05e-06 | 4.95e-06 |
| ENSG00000063180 | 1.92e-05 | 7.19e-05 |
| ENSG00000063241 | 2.27e-01 | 3.01e-01 |
| ENSG00000063244 | 4.09e-08 | 2.47e-07 |
| ENSG00000063245 | 1.44e-02 | 2.80e-02 |
| ENSG00000063322 | 3.06e-01 | 3.87e-01 |
| ENSG00000063438 | 1.66e-15 | 3.77e-14 |
| ENSG00000063587 | 1.24e-09 | 9.63e-09 |
| ENSG00000063601 | 1.55e-11 | 1.67e-10 |
| ENSG00000063660 | 3.17e-05 | 1.14e-04 |
| ENSG00000063761 | 1.68e-02 | 3.20e-02 |
| ENSG00000063854 | 3.60e-01 | 4.43e-01 |
| ENSG00000063978 | 1.01e-01 | 1.52e-01 |
| ENSG00000064012 | 3.07e-02 | 5.42e-02 |
| ENSG00000064042 | 7.55e-02 | 1.18e-01 |
| ENSG00000064102 | 9.34e-17 | 2.80e-15 |
| ENSG00000064115 | 8.38e-02 | 1.29e-01 |
| ENSG00000064195 | 4.52e-02 | 7.59e-02 |
| ENSG00000064199 | 2.52e-04 | 7.57e-04 |
| ENSG00000064201 | 1.06e-05 | 4.19e-05 |
| ENSG00000064205 | 9.40e-20 | 5.72e-18 |
| ENSG00000064218 | 3.14e-10 | 2.70e-09 |
| ENSG00000064225 | 6.03e-04 | 1.68e-03 |
| ENSG00000064270 | 2.59e-01 | 3.36e-01 |
| ENSG00000064300 | 5.60e-12 | 6.57e-11 |
| ENSG00000064309 | 1.48e-01 | 2.10e-01 |
| ENSG00000064313 | 2.92e-08 | 1.80e-07 |
| ENSG00000064393 | 2.91e-02 | 5.17e-02 |
| ENSG00000064419 | 7.75e-09 | 5.27e-08 |
| ENSG00000064489 | 5.82e-02 | 9.44e-02 |
| ENSG00000064490 | 3.46e-03 | 8.01e-03 |
| ENSG00000064545 | 1.66e-15 | 3.77e-14 |
| ENSG00000064547 | 1.68e-08 | 1.08e-07 |
| ENSG00000064601 | 2.11e-07 | 1.12e-06 |
| ENSG00000064607 | 7.32e-10 | 5.91e-09 |
| ENSG00000064651 | 6.27e-14 | 1.05e-12 |
| ENSG00000064652 | 9.97e-17 | 2.96e-15 |
| ENSG00000064655 | 9.06e-16 | 2.18e-14 |
| ENSG00000064666 | 9.99e-23 | 1.52e-20 |
| ENSG00000064687 | 1.79e-02 | 3.40e-02 |
| ENSG00000064692 | 1.94e-01 | 2.64e-01 |
| ENSG00000064703 | 2.31e-06 | 1.02e-05 |
| ENSG00000064726 | 1.38e-01 | 1.97e-01 |
| ENSG00000064763 | 9.86e-06 | 3.90e-05 |
| ENSG00000064787 | 7.39e-17 | 2.25e-15 |
| ENSG00000064835 | 1.22e-01 | 1.78e-01 |
| ENSG00000064886 | 7.10e-05 | 2.38e-04 |
| ENSG00000064932 | 4.30e-11 | 4.30e-10 |
| ENSG00000064933 | 3.30e-10 | 2.83e-09 |
| ENSG00000064961 | 1.21e-01 | 1.77e-01 |
| ENSG00000064989 | 9.90e-01 | 9.93e-01 |
| ENSG00000064995 | 5.17e-01 | 5.97e-01 |
| ENSG00000064999 | 1.45e-01 | 2.06e-01 |
| ENSG00000065000 | 4.51e-05 | 1.58e-04 |
| ENSG00000065029 | 5.18e-02 | 8.55e-02 |
| ENSG00000065054 | 3.81e-02 | 6.55e-02 |
| ENSG00000065057 | 7.40e-11 | 7.09e-10 |
| ENSG00000065060 | 8.48e-09 | 5.72e-08 |
| ENSG00000065135 | 6.00e-01 | 6.73e-01 |
| ENSG00000065150 | 1.29e-17 | 4.61e-16 |
| ENSG00000065154 | 3.32e-02 | 5.81e-02 |
| ENSG00000065183 | 6.00e-17 | 1.87e-15 |
| ENSG00000065243 | 3.33e-03 | 7.75e-03 |
| ENSG00000065268 | 1.12e-07 | 6.24e-07 |
| ENSG00000065308 | 6.26e-01 | 6.97e-01 |
| ENSG00000065320 | 4.44e-22 | 5.55e-20 |
| ENSG00000065325 | 7.54e-28 | 7.44e-25 |
| ENSG00000065328 | 5.55e-19 | 2.81e-17 |
| ENSG00000065357 | 5.29e-10 | 4.37e-09 |
| ENSG00000065361 | 1.94e-01 | 2.64e-01 |
| ENSG00000065371 | 3.48e-14 | 6.13e-13 |
| ENSG00000065413 | 1.38e-07 | 7.60e-07 |
| ENSG00000065427 | 5.78e-11 | 5.64e-10 |
| ENSG00000065457 | 5.44e-08 | 3.21e-07 |
| ENSG00000065485 | 1.19e-04 | 3.81e-04 |
| ENSG00000065491 | 2.13e-04 | 6.50e-04 |
| ENSG00000065518 | 9.35e-03 | 1.92e-02 |
| ENSG00000065526 | 7.51e-01 | 8.05e-01 |
| ENSG00000065534 | 2.30e-13 | 3.49e-12 |
| ENSG00000065548 | 1.33e-07 | 7.33e-07 |
| ENSG00000065559 | 1.56e-10 | 1.41e-09 |
| ENSG00000065600 | 3.32e-27 | 2.62e-24 |
| ENSG00000065609 | 2.22e-15 | 4.92e-14 |
| ENSG00000065613 | 7.46e-03 | 1.58e-02 |
| ENSG00000065615 | 1.01e-02 | 2.06e-02 |
| ENSG00000065618 | 2.71e-08 | 1.68e-07 |
| ENSG00000065621 | 2.24e-03 | 5.44e-03 |
| ENSG00000065665 | 1.69e-07 | 9.13e-07 |
| ENSG00000065675 | 3.59e-01 | 4.42e-01 |
| ENSG00000065717 | 6.74e-01 | 7.39e-01 |
| ENSG00000065802 | 4.24e-02 | 7.19e-02 |
| ENSG00000065809 | 1.77e-13 | 2.74e-12 |
| ENSG00000065833 | 1.01e-05 | 3.98e-05 |
| ENSG00000065882 | 3.71e-07 | 1.90e-06 |
| ENSG00000065883 | 3.39e-04 | 9.91e-04 |
| ENSG00000065911 | 3.05e-19 | 1.63e-17 |
| ENSG00000065923 | 1.21e-07 | 6.76e-07 |
| ENSG00000065970 | 8.98e-01 | 9.24e-01 |
| ENSG00000065978 | 2.27e-02 | 4.17e-02 |
| ENSG00000065989 | 2.11e-02 | 3.92e-02 |
| ENSG00000066027 | 3.45e-05 | 1.24e-04 |
| ENSG00000066032 | 9.55e-08 | 5.40e-07 |
| ENSG00000066044 | 7.19e-18 | 2.74e-16 |
| ENSG00000066056 | 8.62e-01 | 8.96e-01 |
| ENSG00000066084 | 1.17e-01 | 1.72e-01 |
| ENSG00000066117 | 1.17e-10 | 1.08e-09 |
| ENSG00000066135 | 7.73e-08 | 4.44e-07 |
| ENSG00000066136 | 1.38e-03 | 3.54e-03 |
| ENSG00000066185 | 1.03e-06 | 4.86e-06 |
| ENSG00000066230 | 9.04e-10 | 7.19e-09 |
| ENSG00000066248 | 1.89e-03 | 4.68e-03 |
| ENSG00000066279 | 1.66e-09 | 1.27e-08 |
| ENSG00000066294 | 3.94e-02 | 6.75e-02 |
| ENSG00000066322 | 5.60e-02 | 9.13e-02 |
| ENSG00000066336 | 2.73e-02 | 4.90e-02 |
| ENSG00000066379 | 3.62e-11 | 3.66e-10 |
| ENSG00000066382 | 8.48e-08 | 4.84e-07 |
| ENSG00000066405 | 8.86e-04 | 2.37e-03 |
| ENSG00000066422 | 5.21e-03 | 1.15e-02 |
| ENSG00000066427 | 3.33e-01 | 4.15e-01 |
| ENSG00000066455 | 3.37e-02 | 5.89e-02 |
| ENSG00000066468 | 1.09e-18 | 5.09e-17 |
| ENSG00000066557 | 4.72e-04 | 1.34e-03 |
| ENSG00000066583 | 2.20e-01 | 2.93e-01 |
| ENSG00000066629 | 7.25e-09 | 4.96e-08 |
| ENSG00000066651 | 3.45e-16 | 9.14e-15 |
| ENSG00000066654 | 8.76e-01 | 9.07e-01 |
| ENSG00000066697 | 1.89e-11 | 2.00e-10 |
| ENSG00000066735 | 4.13e-05 | 1.45e-04 |
| ENSG00000066739 | 3.38e-02 | 5.90e-02 |
| ENSG00000066777 | 9.27e-01 | 9.46e-01 |
| ENSG00000066813 | 1.09e-04 | 3.53e-04 |
| ENSG00000066827 | 7.92e-01 | 8.39e-01 |
| ENSG00000066855 | 5.78e-09 | 4.02e-08 |
| ENSG00000066923 | 6.56e-02 | 1.05e-01 |
| ENSG00000066926 | 3.25e-14 | 5.76e-13 |
| ENSG00000066933 | 1.75e-05 | 6.61e-05 |
| ENSG00000067048 | 4.03e-01 | 4.86e-01 |
| ENSG00000067057 | 6.59e-02 | 1.05e-01 |
| ENSG00000067064 | 5.23e-01 | 6.02e-01 |
| ENSG00000067066 | 3.28e-02 | 5.76e-02 |
| ENSG00000067082 | 1.93e-10 | 1.72e-09 |
| ENSG00000067113 | 2.86e-24 | 7.08e-22 |
| ENSG00000067141 | 6.42e-15 | 1.30e-13 |
| ENSG00000067167 | 4.10e-01 | 4.93e-01 |
| ENSG00000067177 | 1.15e-11 | 1.26e-10 |
| ENSG00000067182 | 2.39e-12 | 3.02e-11 |
| ENSG00000067191 | 3.24e-14 | 5.75e-13 |
| ENSG00000067208 | 4.65e-01 | 5.47e-01 |
| ENSG00000067221 | 1.16e-01 | 1.71e-01 |
| ENSG00000067225 | 3.66e-11 | 3.70e-10 |
| ENSG00000067248 | 6.37e-01 | 7.07e-01 |
| ENSG00000067334 | 6.14e-03 | 1.33e-02 |
| ENSG00000067365 | 8.23e-12 | 9.34e-11 |
| ENSG00000067369 | 3.14e-06 | 1.35e-05 |
| ENSG00000067445 | 4.01e-01 | 4.84e-01 |
| ENSG00000067533 | 1.74e-11 | 1.85e-10 |
| ENSG00000067560 | 2.90e-01 | 3.70e-01 |
| ENSG00000067596 | 1.12e-05 | 4.38e-05 |
| ENSG00000067601 | 3.61e-04 | 1.05e-03 |
| ENSG00000067606 | 7.20e-02 | 1.13e-01 |
| ENSG00000067646 | 4.00e-02 | 6.83e-02 |
| ENSG00000067704 | 7.87e-01 | 8.35e-01 |
| ENSG00000067715 | 6.55e-03 | 1.41e-02 |
| ENSG00000067798 | 1.63e-03 | 4.10e-03 |
| ENSG00000067829 | 1.33e-02 | 2.62e-02 |
| ENSG00000067836 | 2.87e-07 | 1.50e-06 |
| ENSG00000067840 | 3.55e-22 | 4.55e-20 |
| ENSG00000067842 | 1.88e-12 | 2.42e-11 |
| ENSG00000067900 | 2.51e-04 | 7.54e-04 |
| ENSG00000067955 | 1.00e-19 | 6.06e-18 |
| ENSG00000067992 | 2.60e-04 | 7.79e-04 |
| ENSG00000068001 | 4.04e-11 | 4.06e-10 |
| ENSG00000068024 | 9.46e-02 | 1.43e-01 |
| ENSG00000068028 | 3.64e-12 | 4.43e-11 |
| ENSG00000068078 | 2.76e-04 | 8.22e-04 |
| ENSG00000068079 | 6.03e-02 | 9.75e-02 |
| ENSG00000068097 | 6.04e-03 | 1.31e-02 |
| ENSG00000068120 | 1.76e-03 | 4.39e-03 |
| ENSG00000068137 | 2.14e-04 | 6.53e-04 |
| ENSG00000068305 | 1.49e-06 | 6.80e-06 |
| ENSG00000068308 | 4.37e-03 | 9.83e-03 |
| ENSG00000068323 | 8.87e-02 | 1.36e-01 |
| ENSG00000068354 | 1.07e-01 | 1.60e-01 |
| ENSG00000068366 | 1.91e-09 | 1.44e-08 |
| ENSG00000068383 | 5.80e-13 | 8.16e-12 |
| ENSG00000068394 | 1.90e-01 | 2.59e-01 |
| ENSG00000068400 | 1.14e-04 | 3.68e-04 |
| ENSG00000068438 | 2.43e-23 | 4.44e-21 |
| ENSG00000068489 | 2.77e-16 | 7.49e-15 |
| ENSG00000068615 | 7.40e-05 | 2.48e-04 |
| ENSG00000068650 | 1.30e-23 | 2.68e-21 |
| ENSG00000068654 | 3.71e-15 | 7.95e-14 |
| ENSG00000068697 | 1.85e-02 | 3.49e-02 |
| ENSG00000068724 | 1.02e-07 | 5.76e-07 |
| ENSG00000068745 | 9.09e-01 | 9.33e-01 |
| ENSG00000068781 | 1.38e-04 | 4.37e-04 |
| ENSG00000068784 | 6.99e-01 | 7.61e-01 |
| ENSG00000068796 | 1.28e-03 | 3.30e-03 |
| ENSG00000068831 | 1.09e-11 | 1.21e-10 |
| ENSG00000068878 | 3.10e-08 | 1.90e-07 |
| ENSG00000068885 | 8.99e-03 | 1.86e-02 |
| ENSG00000068903 | 1.68e-01 | 2.33e-01 |
| ENSG00000068912 | 7.26e-01 | 7.84e-01 |
| ENSG00000068971 | 1.10e-04 | 3.55e-04 |
| ENSG00000068976 | 1.08e-15 | 2.58e-14 |
| ENSG00000068985 | 1.20e-02 | 2.40e-02 |
| ENSG00000069011 | 1.90e-11 | 2.01e-10 |
| ENSG00000069018 | 5.11e-12 | 6.03e-11 |
| ENSG00000069020 | 3.72e-03 | 8.53e-03 |
| ENSG00000069122 | 1.59e-03 | 4.01e-03 |
| ENSG00000069188 | 2.69e-04 | 8.03e-04 |
| ENSG00000069248 | 9.02e-08 | 5.13e-07 |
| ENSG00000069275 | 8.44e-02 | 1.30e-01 |
| ENSG00000069329 | 2.16e-02 | 4.00e-02 |
| ENSG00000069345 | 1.78e-01 | 2.45e-01 |
| ENSG00000069399 | 9.85e-02 | 1.48e-01 |
| ENSG00000069424 | 3.43e-06 | 1.47e-05 |
| ENSG00000069431 | 7.25e-01 | 7.83e-01 |
| ENSG00000069482 | 2.45e-02 | 4.45e-02 |
| ENSG00000069493 | 4.57e-05 | 1.60e-04 |
| ENSG00000069509 | 6.18e-06 | 2.53e-05 |
| ENSG00000069535 | 1.52e-13 | 2.39e-12 |
| ENSG00000069667 | 1.26e-04 | 4.03e-04 |
| ENSG00000069696 | 1.34e-04 | 4.26e-04 |
| ENSG00000069702 | 2.74e-06 | 1.19e-05 |
| ENSG00000069712 | 3.30e-01 | 4.12e-01 |
| ENSG00000069764 | 8.51e-11 | 8.08e-10 |
| ENSG00000069812 | 2.04e-03 | 5.01e-03 |
| ENSG00000069849 | 1.04e-10 | 9.68e-10 |
| ENSG00000069869 | 2.85e-05 | 1.04e-04 |
| ENSG00000069943 | 2.94e-02 | 5.22e-02 |
| ENSG00000069956 | 9.27e-09 | 6.21e-08 |
| ENSG00000069966 | 3.82e-01 | 4.65e-01 |
| ENSG00000069974 | 5.50e-14 | 9.35e-13 |
| ENSG00000069998 | 8.37e-06 | 3.36e-05 |
| ENSG00000070010 | 1.36e-05 | 5.27e-05 |
| ENSG00000070018 | 3.68e-02 | 6.36e-02 |
| ENSG00000070019 | 1.89e-02 | 3.56e-02 |
| ENSG00000070031 | 3.39e-03 | 7.87e-03 |
| ENSG00000070047 | 3.47e-03 | 8.04e-03 |
| ENSG00000070061 | 3.53e-11 | 3.58e-10 |
| ENSG00000070081 | 2.38e-03 | 5.74e-03 |
| ENSG00000070087 | 3.93e-01 | 4.76e-01 |
| ENSG00000070159 | 1.22e-01 | 1.78e-01 |
| ENSG00000070182 | 9.14e-01 | 9.36e-01 |
| ENSG00000070190 | 9.94e-01 | 9.96e-01 |
| ENSG00000070193 | 1.69e-08 | 1.09e-07 |
| ENSG00000070214 | 1.24e-09 | 9.67e-09 |
| ENSG00000070269 | 2.32e-02 | 4.26e-02 |
| ENSG00000070366 | 3.87e-07 | 1.96e-06 |
| ENSG00000070367 | 2.99e-01 | 3.80e-01 |
| ENSG00000070371 | 3.86e-03 | 8.81e-03 |
| ENSG00000070388 | 5.88e-01 | 6.62e-01 |
| ENSG00000070404 | 4.21e-11 | 4.22e-10 |
| ENSG00000070413 | 1.96e-06 | 8.75e-06 |
| ENSG00000070423 | 9.16e-07 | 4.37e-06 |
| ENSG00000070444 | 3.35e-05 | 1.20e-04 |
| ENSG00000070476 | 1.31e-09 | 1.01e-08 |
| ENSG00000070495 | 4.75e-06 | 1.99e-05 |
| ENSG00000070501 | 1.39e-11 | 1.50e-10 |
| ENSG00000070526 | 4.68e-09 | 3.30e-08 |
| ENSG00000070540 | 5.06e-03 | 1.12e-02 |
| ENSG00000070601 | 2.05e-02 | 3.83e-02 |
| ENSG00000070610 | 9.64e-09 | 6.43e-08 |
| ENSG00000070614 | 2.43e-05 | 8.94e-05 |
| ENSG00000070669 | 9.26e-19 | 4.41e-17 |
| ENSG00000070718 | 2.69e-16 | 7.33e-15 |
| ENSG00000070729 | 1.55e-23 | 3.11e-21 |
| ENSG00000070731 | 4.43e-03 | 9.95e-03 |
| ENSG00000070748 | 2.76e-25 | 1.01e-22 |
| ENSG00000070756 | 1.34e-20 | 1.05e-18 |
| ENSG00000070759 | 7.35e-20 | 4.59e-18 |
| ENSG00000070761 | 5.64e-13 | 7.96e-12 |
| ENSG00000070770 | 7.57e-19 | 3.70e-17 |
| ENSG00000070778 | 4.37e-21 | 3.93e-19 |
| ENSG00000070785 | 1.65e-08 | 1.06e-07 |
| ENSG00000070808 | 2.78e-11 | 2.87e-10 |
| ENSG00000070814 | 5.77e-16 | 1.45e-14 |
| ENSG00000070831 | 2.87e-08 | 1.77e-07 |
| ENSG00000070882 | 3.97e-20 | 2.68e-18 |
| ENSG00000070886 | 9.35e-06 | 3.72e-05 |
| ENSG00000070915 | 1.44e-02 | 2.81e-02 |
| ENSG00000070950 | 1.01e-14 | 1.98e-13 |
| ENSG00000070961 | 4.22e-10 | 3.56e-09 |
| ENSG00000070985 | 7.48e-05 | 2.50e-04 |
| ENSG00000071051 | 3.14e-01 | 3.95e-01 |
| ENSG00000071054 | 1.12e-11 | 1.24e-10 |
| ENSG00000071073 | 4.07e-13 | 5.91e-12 |
| ENSG00000071082 | 1.53e-06 | 6.98e-06 |
| ENSG00000071127 | 6.13e-06 | 2.51e-05 |
| ENSG00000071189 | 1.65e-03 | 4.15e-03 |
| ENSG00000071203 | 2.19e-19 | 1.22e-17 |
| ENSG00000071205 | 4.14e-05 | 1.46e-04 |
| ENSG00000071242 | 4.23e-02 | 7.18e-02 |
| ENSG00000071243 | 7.72e-01 | 8.23e-01 |
| ENSG00000071246 | 6.97e-02 | 1.10e-01 |
| ENSG00000071282 | 3.17e-12 | 3.90e-11 |
| ENSG00000071462 | 8.01e-18 | 3.02e-16 |
| ENSG00000071537 | 4.02e-08 | 2.43e-07 |
| ENSG00000071539 | 7.63e-26 | 3.59e-23 |
| ENSG00000071553 | 1.35e-05 | 5.21e-05 |
| ENSG00000071564 | 2.39e-14 | 4.37e-13 |
| ENSG00000071575 | 1.70e-02 | 3.24e-02 |
| ENSG00000071626 | 1.51e-12 | 1.97e-11 |
| ENSG00000071655 | 7.65e-02 | 1.20e-01 |
| ENSG00000071794 | 9.63e-01 | 9.72e-01 |
| ENSG00000071859 | 1.01e-06 | 4.77e-06 |
| ENSG00000071889 | 4.62e-01 | 5.45e-01 |
| ENSG00000071894 | 1.28e-09 | 9.93e-09 |
| ENSG00000071909 | 1.59e-05 | 6.04e-05 |
| ENSG00000071967 | 4.07e-12 | 4.90e-11 |
| ENSG00000071991 | 1.34e-17 | 4.80e-16 |
| ENSG00000071994 | 8.55e-13 | 1.17e-11 |
| ENSG00000072041 | 5.39e-18 | 2.11e-16 |
| ENSG00000072042 | 7.90e-10 | 6.34e-09 |
| ENSG00000072062 | 2.90e-10 | 2.51e-09 |
| ENSG00000072071 | 1.37e-03 | 3.51e-03 |
| ENSG00000072080 | 6.90e-01 | 7.54e-01 |
| ENSG00000072110 | 1.09e-03 | 2.85e-03 |
| ENSG00000072121 | 1.70e-02 | 3.24e-02 |
| ENSG00000072133 | 4.43e-08 | 2.66e-07 |
| ENSG00000072134 | 4.38e-02 | 7.40e-02 |
| ENSG00000072135 | 2.18e-05 | 8.09e-05 |
| ENSG00000072163 | 3.38e-08 | 2.06e-07 |
| ENSG00000072182 | 9.77e-03 | 2.00e-02 |
| ENSG00000072195 | 6.78e-10 | 5.50e-09 |
| ENSG00000072201 | 3.64e-01 | 4.47e-01 |
| ENSG00000072210 | 5.44e-15 | 1.12e-13 |
| ENSG00000072274 | 5.11e-06 | 2.12e-05 |
| ENSG00000072310 | 2.39e-02 | 4.36e-02 |
| ENSG00000072315 | 1.54e-01 | 2.17e-01 |
| ENSG00000072364 | 1.05e-02 | 2.13e-02 |
| ENSG00000072401 | 3.14e-01 | 3.95e-01 |
| ENSG00000072415 | 6.63e-11 | 6.41e-10 |
| ENSG00000072422 | 1.43e-06 | 6.57e-06 |
| ENSG00000072501 | 2.05e-07 | 1.09e-06 |
| ENSG00000072506 | 7.30e-08 | 4.21e-07 |
| ENSG00000072518 | 1.56e-05 | 5.95e-05 |
| ENSG00000072571 | 9.42e-10 | 7.47e-09 |
| ENSG00000072609 | 1.86e-07 | 1.00e-06 |
| ENSG00000072657 | 3.44e-16 | 9.12e-15 |
| ENSG00000072682 | 3.01e-01 | 3.81e-01 |
| ENSG00000072694 | 3.08e-07 | 1.60e-06 |
| ENSG00000072736 | 2.31e-07 | 1.22e-06 |
| ENSG00000072756 | 4.87e-08 | 2.90e-07 |
| ENSG00000072778 | 1.71e-11 | 1.82e-10 |
| ENSG00000072786 | 4.49e-01 | 5.32e-01 |
| ENSG00000072803 | 5.52e-01 | 6.30e-01 |
| ENSG00000072818 | 5.47e-06 | 2.26e-05 |
| ENSG00000072832 | 1.87e-01 | 2.55e-01 |
| ENSG00000072840 | 5.84e-04 | 1.63e-03 |
| ENSG00000072849 | 2.49e-05 | 9.16e-05 |
| ENSG00000072858 | 2.38e-06 | 1.05e-05 |
| ENSG00000072864 | 5.67e-19 | 2.86e-17 |
| ENSG00000072952 | 2.94e-06 | 1.27e-05 |
| ENSG00000072954 | 9.21e-04 | 2.45e-03 |
| ENSG00000072958 | 6.37e-03 | 1.37e-02 |
| ENSG00000073008 | 1.47e-18 | 6.59e-17 |
| ENSG00000073050 | 1.52e-08 | 9.86e-08 |
| ENSG00000073060 | 5.67e-09 | 3.95e-08 |
| ENSG00000073067 | 8.57e-09 | 5.78e-08 |
| ENSG00000073111 | 1.08e-15 | 2.57e-14 |
| ENSG00000073146 | 1.24e-05 | 4.81e-05 |
| ENSG00000073150 | 5.97e-06 | 2.45e-05 |
| ENSG00000073169 | 1.28e-02 | 2.53e-02 |
| ENSG00000073282 | 1.99e-03 | 4.89e-03 |
| ENSG00000073331 | 9.12e-01 | 9.35e-01 |
| ENSG00000073350 | 1.71e-01 | 2.37e-01 |
| ENSG00000073417 | 5.00e-16 | 1.28e-14 |
| ENSG00000073464 | 9.04e-11 | 8.54e-10 |
| ENSG00000073536 | 4.57e-18 | 1.82e-16 |
| ENSG00000073578 | 1.12e-11 | 1.24e-10 |
| ENSG00000073584 | 3.16e-01 | 3.97e-01 |
| ENSG00000073598 | 4.74e-01 | 5.57e-01 |
| ENSG00000073605 | 6.58e-04 | 1.81e-03 |
| ENSG00000073614 | 1.93e-01 | 2.63e-01 |
| ENSG00000073670 | 2.77e-03 | 6.58e-03 |
| ENSG00000073711 | 7.06e-22 | 8.35e-20 |
| ENSG00000073712 | 1.86e-04 | 5.76e-04 |
| ENSG00000073734 | 2.98e-20 | 2.08e-18 |
| ENSG00000073737 | 2.47e-18 | 1.05e-16 |
| ENSG00000073754 | 7.57e-03 | 1.60e-02 |
| ENSG00000073756 | 5.67e-02 | 9.24e-02 |
| ENSG00000073792 | 1.04e-02 | 2.10e-02 |
| ENSG00000073803 | 2.76e-02 | 4.95e-02 |
| ENSG00000073849 | 3.30e-01 | 4.12e-01 |
| ENSG00000073861 | 6.04e-08 | 3.54e-07 |
| ENSG00000073905 | 7.88e-08 | 4.52e-07 |
| ENSG00000073910 | 3.05e-01 | 3.86e-01 |
| ENSG00000073921 | 5.47e-01 | 6.24e-01 |
| ENSG00000073969 | 2.36e-02 | 4.32e-02 |
| ENSG00000074047 | 9.36e-01 | 9.52e-01 |
| ENSG00000074054 | 8.76e-04 | 2.34e-03 |
| ENSG00000074071 | 1.31e-06 | 6.04e-06 |
| ENSG00000074181 | 3.08e-15 | 6.68e-14 |
| ENSG00000074201 | 1.87e-17 | 6.56e-16 |
| ENSG00000074211 | 2.40e-01 | 3.16e-01 |
| ENSG00000074219 | 3.09e-03 | 7.25e-03 |
| ENSG00000074266 | 1.43e-05 | 5.51e-05 |
| ENSG00000074276 | 1.10e-11 | 1.21e-10 |
| ENSG00000074317 | 3.38e-08 | 2.06e-07 |
| ENSG00000074319 | 8.77e-01 | 9.08e-01 |
| ENSG00000074356 | 1.21e-03 | 3.13e-03 |
| ENSG00000074370 | 2.94e-12 | 3.64e-11 |
| ENSG00000074410 | 1.12e-17 | 4.09e-16 |
| ENSG00000074416 | 5.24e-15 | 1.08e-13 |
| ENSG00000074527 | 2.11e-10 | 1.88e-09 |
| ENSG00000074582 | 1.16e-11 | 1.27e-10 |
| ENSG00000074590 | 2.88e-04 | 8.55e-04 |
| ENSG00000074603 | 1.61e-05 | 6.13e-05 |
| ENSG00000074621 | 5.17e-07 | 2.57e-06 |
| ENSG00000074657 | 4.82e-01 | 5.64e-01 |
| ENSG00000074660 | 9.10e-02 | 1.39e-01 |
| ENSG00000074695 | 4.88e-01 | 5.69e-01 |
| ENSG00000074696 | 2.07e-18 | 8.95e-17 |
| ENSG00000074706 | 9.56e-07 | 4.54e-06 |
| ENSG00000074755 | 7.13e-22 | 8.42e-20 |
| ENSG00000074771 | 3.06e-01 | 3.87e-01 |
| ENSG00000074800 | 2.06e-09 | 1.54e-08 |
| ENSG00000074803 | 6.93e-02 | 1.10e-01 |
| ENSG00000074842 | 1.51e-05 | 5.76e-05 |
| ENSG00000074855 | 4.48e-08 | 2.69e-07 |
| ENSG00000074935 | 2.28e-05 | 8.45e-05 |
| ENSG00000074964 | 3.52e-01 | 4.34e-01 |
| ENSG00000074966 | 1.37e-04 | 4.33e-04 |
| ENSG00000075035 | 5.00e-13 | 7.14e-12 |
| ENSG00000075043 | 9.83e-03 | 2.01e-02 |
| ENSG00000075073 | 7.77e-08 | 4.46e-07 |
| ENSG00000075089 | 2.23e-01 | 2.97e-01 |
| ENSG00000075131 | 3.64e-09 | 2.61e-08 |
| ENSG00000075142 | 1.22e-16 | 3.55e-15 |
| ENSG00000075151 | 3.73e-04 | 1.08e-03 |
| ENSG00000075188 | 5.58e-14 | 9.46e-13 |
| ENSG00000075213 | 2.74e-01 | 3.53e-01 |
| ENSG00000075218 | 2.16e-16 | 6.00e-15 |
| ENSG00000075223 | 6.26e-04 | 1.73e-03 |
| ENSG00000075234 | 3.61e-09 | 2.59e-08 |
| ENSG00000075239 | 9.97e-23 | 1.52e-20 |
| ENSG00000075240 | 2.15e-10 | 1.90e-09 |
| ENSG00000075275 | 2.28e-20 | 1.65e-18 |
| ENSG00000075290 | 1.14e-10 | 1.06e-09 |
| ENSG00000075292 | 9.63e-01 | 9.72e-01 |
| ENSG00000075303 | 8.72e-01 | 9.04e-01 |
| ENSG00000075336 | 1.14e-06 | 5.31e-06 |
| ENSG00000075340 | 9.22e-10 | 7.33e-09 |
| ENSG00000075388 | 1.62e-03 | 4.06e-03 |
| ENSG00000075391 | 9.49e-09 | 6.34e-08 |
| ENSG00000075399 | 3.00e-03 | 7.06e-03 |
| ENSG00000075407 | 1.37e-03 | 3.51e-03 |
| ENSG00000075413 | 2.87e-03 | 6.79e-03 |
| ENSG00000075415 | 7.08e-04 | 1.94e-03 |
| ENSG00000075420 | 1.43e-03 | 3.64e-03 |
| ENSG00000075426 | 1.36e-05 | 5.27e-05 |
| ENSG00000075429 | 7.00e-08 | 4.05e-07 |
| ENSG00000075461 | 6.06e-03 | 1.31e-02 |
| ENSG00000075539 | 4.22e-07 | 2.13e-06 |
| ENSG00000075568 | 2.22e-11 | 2.33e-10 |
| ENSG00000075618 | 4.30e-10 | 3.62e-09 |
| ENSG00000075624 | 3.81e-01 | 4.64e-01 |
| ENSG00000075643 | 6.11e-07 | 3.00e-06 |
| ENSG00000075651 | 7.49e-09 | 5.11e-08 |
| ENSG00000075673 | 1.44e-03 | 3.68e-03 |
| ENSG00000075702 | 1.84e-20 | 1.37e-18 |
| ENSG00000075711 | 1.37e-02 | 2.68e-02 |
| ENSG00000075785 | 8.02e-01 | 8.48e-01 |
| ENSG00000075790 | 2.82e-04 | 8.39e-04 |
| ENSG00000075826 | 1.83e-02 | 3.46e-02 |
| ENSG00000075856 | 1.99e-11 | 2.10e-10 |
| ENSG00000075884 | 1.36e-11 | 1.48e-10 |
| ENSG00000075886 | 7.80e-03 | 1.64e-02 |
| ENSG00000075891 | 5.63e-02 | 9.17e-02 |
| ENSG00000075914 | 1.02e-19 | 6.18e-18 |
| ENSG00000075945 | 1.01e-05 | 3.98e-05 |
| ENSG00000075975 | 1.63e-06 | 7.38e-06 |
| ENSG00000076003 | 3.64e-16 | 9.61e-15 |
| ENSG00000076043 | 6.92e-15 | 1.40e-13 |
| ENSG00000076053 | 4.57e-01 | 5.40e-01 |
| ENSG00000076067 | 4.75e-01 | 5.58e-01 |
| ENSG00000076108 | 5.97e-01 | 6.71e-01 |
| ENSG00000076201 | 1.57e-04 | 4.93e-04 |
| ENSG00000076242 | 1.13e-01 | 1.67e-01 |
| ENSG00000076248 | 1.49e-03 | 3.78e-03 |
| ENSG00000076258 | 6.65e-17 | 2.04e-15 |
| ENSG00000076321 | 1.31e-02 | 2.59e-02 |
| ENSG00000076344 | 2.72e-02 | 4.89e-02 |
| ENSG00000076351 | 3.31e-06 | 1.42e-05 |
| ENSG00000076356 | 1.08e-09 | 8.50e-09 |
| ENSG00000076382 | 8.83e-09 | 5.94e-08 |
| ENSG00000076513 | 3.39e-13 | 4.99e-12 |
| ENSG00000076554 | 3.58e-03 | 8.25e-03 |
| ENSG00000076555 | 5.03e-20 | 3.26e-18 |
| ENSG00000076604 | 3.34e-03 | 7.77e-03 |
| ENSG00000076641 | 6.27e-15 | 1.28e-13 |
| ENSG00000076650 | 6.57e-01 | 7.25e-01 |
| ENSG00000076662 | 2.33e-09 | 1.73e-08 |
| ENSG00000076685 | 8.67e-02 | 1.33e-01 |
| ENSG00000076706 | 4.53e-01 | 5.36e-01 |
| ENSG00000076716 | 1.72e-05 | 6.50e-05 |
| ENSG00000076770 | 4.04e-05 | 1.42e-04 |
| ENSG00000076826 | 7.57e-02 | 1.18e-01 |
| ENSG00000076864 | 6.13e-02 | 9.88e-02 |
| ENSG00000076924 | 2.45e-05 | 9.03e-05 |
| ENSG00000076928 | 3.30e-07 | 1.70e-06 |
| ENSG00000076944 | 5.42e-01 | 6.20e-01 |
| ENSG00000076984 | 3.56e-01 | 4.38e-01 |
| ENSG00000077009 | 1.41e-10 | 1.29e-09 |
| ENSG00000077044 | 2.11e-01 | 2.84e-01 |
| ENSG00000077063 | 8.70e-03 | 1.80e-02 |
| ENSG00000077080 | 2.44e-12 | 3.08e-11 |
| ENSG00000077092 | 6.72e-01 | 7.37e-01 |
| ENSG00000077097 | 4.59e-01 | 5.42e-01 |
| ENSG00000077147 | 5.10e-01 | 5.90e-01 |
| ENSG00000077150 | 8.50e-06 | 3.41e-05 |
| ENSG00000077152 | 2.15e-17 | 7.44e-16 |
| ENSG00000077157 | 1.50e-11 | 1.61e-10 |
| ENSG00000077232 | 2.01e-09 | 1.51e-08 |
| ENSG00000077235 | 3.53e-02 | 6.13e-02 |
| ENSG00000077238 | 2.72e-07 | 1.43e-06 |
| ENSG00000077254 | 3.44e-03 | 7.97e-03 |
| ENSG00000077264 | 1.19e-10 | 1.10e-09 |
| ENSG00000077274 | 1.15e-01 | 1.70e-01 |
| ENSG00000077279 | 2.72e-04 | 8.11e-04 |
| ENSG00000077312 | 1.45e-09 | 1.11e-08 |
| ENSG00000077327 | 8.02e-06 | 3.23e-05 |
| ENSG00000077348 | 1.02e-15 | 2.44e-14 |
| ENSG00000077380 | 7.69e-01 | 8.20e-01 |
| ENSG00000077420 | 7.16e-10 | 5.79e-09 |
| ENSG00000077454 | 5.80e-02 | 9.42e-02 |
| ENSG00000077458 | 3.33e-02 | 5.82e-02 |
| ENSG00000077463 | 8.17e-09 | 5.53e-08 |
| ENSG00000077498 | 6.88e-02 | 1.09e-01 |
| ENSG00000077514 | 3.63e-01 | 4.46e-01 |
| ENSG00000077522 | 1.64e-03 | 4.11e-03 |
| ENSG00000077549 | 3.22e-08 | 1.97e-07 |
| ENSG00000077585 | 5.03e-05 | 1.74e-04 |
| ENSG00000077616 | 9.87e-13 | 1.33e-11 |
| ENSG00000077684 | 4.87e-01 | 5.68e-01 |
| ENSG00000077713 | 8.43e-01 | 8.81e-01 |
| ENSG00000077721 | 2.76e-01 | 3.54e-01 |
| ENSG00000077782 | 8.20e-08 | 4.69e-07 |
| ENSG00000077800 | 6.56e-01 | 7.24e-01 |
| ENSG00000077935 | 1.54e-02 | 2.98e-02 |
| ENSG00000077942 | 3.27e-11 | 3.33e-10 |
| ENSG00000077943 | 6.78e-19 | 3.36e-17 |
| ENSG00000077984 | 2.42e-02 | 4.42e-02 |
| ENSG00000078018 | 2.82e-12 | 3.51e-11 |
| ENSG00000078043 | 3.72e-03 | 8.54e-03 |
| ENSG00000078053 | 1.07e-04 | 3.46e-04 |
| ENSG00000078061 | 2.81e-02 | 5.03e-02 |
| ENSG00000078070 | 1.23e-02 | 2.45e-02 |
| ENSG00000078081 | 2.01e-04 | 6.17e-04 |
| ENSG00000078098 | 1.35e-18 | 6.16e-17 |
| ENSG00000078114 | 2.42e-20 | 1.74e-18 |
| ENSG00000078124 | 2.20e-06 | 9.76e-06 |
| ENSG00000078140 | 4.01e-02 | 6.85e-02 |
| ENSG00000078142 | 9.33e-06 | 3.71e-05 |
| ENSG00000078177 | 5.43e-02 | 8.90e-02 |
| ENSG00000078237 | 4.42e-05 | 1.55e-04 |
| ENSG00000078246 | 7.97e-15 | 1.59e-13 |
| ENSG00000078269 | 4.49e-03 | 1.01e-02 |
| ENSG00000078295 | 2.27e-07 | 1.20e-06 |
| ENSG00000078304 | 6.15e-01 | 6.87e-01 |
| ENSG00000078319 | 2.19e-08 | 1.38e-07 |
| ENSG00000078328 | 1.93e-08 | 1.23e-07 |
| ENSG00000078369 | 8.07e-01 | 8.52e-01 |
| ENSG00000078399 | 1.54e-03 | 3.89e-03 |
| ENSG00000078401 | 2.51e-02 | 4.55e-02 |
| ENSG00000078403 | 4.09e-05 | 1.44e-04 |
| ENSG00000078487 | 4.57e-02 | 7.67e-02 |
| ENSG00000078549 | 1.71e-18 | 7.56e-17 |
| ENSG00000078579 | 7.06e-12 | 8.11e-11 |
| ENSG00000078589 | 1.68e-09 | 1.28e-08 |
| ENSG00000078596 | 2.84e-21 | 2.74e-19 |
| ENSG00000078618 | 7.77e-04 | 2.11e-03 |
| ENSG00000078668 | 6.41e-01 | 7.10e-01 |
| ENSG00000078674 | 9.15e-02 | 1.39e-01 |
| ENSG00000078687 | 7.00e-01 | 7.62e-01 |
| ENSG00000078699 | 2.57e-04 | 7.72e-04 |
| ENSG00000078725 | 1.49e-01 | 2.11e-01 |
| ENSG00000078747 | 9.26e-01 | 9.45e-01 |
| ENSG00000078795 | 9.55e-02 | 1.45e-01 |
| ENSG00000078804 | 6.35e-28 | 6.65e-25 |
| ENSG00000078808 | 3.47e-02 | 6.03e-02 |
| ENSG00000078814 | 6.67e-04 | 1.84e-03 |
| ENSG00000078898 | 2.70e-05 | 9.87e-05 |
| ENSG00000078900 | 3.97e-15 | 8.45e-14 |
| ENSG00000078902 | 9.17e-01 | 9.38e-01 |
| ENSG00000078967 | 9.52e-06 | 3.78e-05 |
| ENSG00000079101 | 8.01e-01 | 8.47e-01 |
| ENSG00000079102 | 7.15e-07 | 3.47e-06 |
| ENSG00000079112 | 3.04e-05 | 1.10e-04 |
| ENSG00000079134 | 1.80e-01 | 2.48e-01 |
| ENSG00000079150 | 1.87e-02 | 3.53e-02 |
| ENSG00000079156 | 7.34e-05 | 2.46e-04 |
| ENSG00000079215 | 5.48e-04 | 1.54e-03 |
| ENSG00000079246 | 1.69e-06 | 7.65e-06 |
| ENSG00000079257 | 3.04e-10 | 2.62e-09 |
| ENSG00000079263 | 9.27e-07 | 4.42e-06 |
| ENSG00000079277 | 3.94e-05 | 1.39e-04 |
| ENSG00000079308 | 9.94e-09 | 6.62e-08 |
| ENSG00000079313 | 6.07e-02 | 9.80e-02 |
| ENSG00000079332 | 9.70e-04 | 2.57e-03 |
| ENSG00000079335 | 3.66e-17 | 1.20e-15 |
| ENSG00000079337 | 2.94e-01 | 3.75e-01 |
| ENSG00000079385 | 1.18e-15 | 2.79e-14 |
| ENSG00000079387 | 7.85e-07 | 3.78e-06 |
| ENSG00000079393 | 1.00e-02 | 2.05e-02 |
| ENSG00000079432 | 8.20e-01 | 8.63e-01 |
| ENSG00000079435 | 2.03e-03 | 4.98e-03 |
| ENSG00000079459 | 6.14e-03 | 1.33e-02 |
| ENSG00000079462 | 1.15e-15 | 2.71e-14 |
| ENSG00000079482 | 4.27e-01 | 5.10e-01 |
| ENSG00000079557 | 2.84e-01 | 3.64e-01 |
| ENSG00000079616 | 9.07e-03 | 1.87e-02 |
| ENSG00000079689 | 1.03e-24 | 2.97e-22 |
| ENSG00000079691 | 8.28e-02 | 1.28e-01 |
| ENSG00000079739 | 9.19e-22 | 1.05e-19 |
| ENSG00000079785 | 2.70e-02 | 4.87e-02 |
| ENSG00000079805 | 6.29e-06 | 2.57e-05 |
| ENSG00000079819 | 3.04e-08 | 1.87e-07 |
| ENSG00000079841 | 7.24e-03 | 1.53e-02 |
| ENSG00000079931 | 6.58e-01 | 7.25e-01 |
| ENSG00000079950 | 1.12e-02 | 2.25e-02 |
| ENSG00000079974 | 8.04e-03 | 1.69e-02 |
| ENSG00000079999 | 3.46e-04 | 1.01e-03 |
| ENSG00000080007 | 1.21e-01 | 1.77e-01 |
| ENSG00000080031 | 5.74e-20 | 3.66e-18 |
| ENSG00000080166 | 8.59e-03 | 1.79e-02 |
| ENSG00000080189 | 1.92e-13 | 2.96e-12 |
| ENSG00000080200 | 7.77e-06 | 3.13e-05 |
| ENSG00000080224 | 6.34e-10 | 5.16e-09 |
| ENSG00000080293 | 2.14e-12 | 2.73e-11 |
| ENSG00000080298 | 6.81e-05 | 2.29e-04 |
| ENSG00000080345 | 5.96e-04 | 1.66e-03 |
| ENSG00000080371 | 7.66e-01 | 8.18e-01 |
| ENSG00000080493 | 3.38e-23 | 5.90e-21 |
| ENSG00000080503 | 1.41e-04 | 4.45e-04 |
| ENSG00000080511 | 6.66e-02 | 1.06e-01 |
| ENSG00000080546 | 5.84e-02 | 9.47e-02 |
| ENSG00000080561 | 9.58e-01 | 9.69e-01 |
| ENSG00000080572 | 8.63e-01 | 8.96e-01 |
| ENSG00000080573 | 4.67e-09 | 3.30e-08 |
| ENSG00000080603 | 1.43e-04 | 4.51e-04 |
| ENSG00000080608 | 3.41e-23 | 5.93e-21 |
| ENSG00000080618 | 6.55e-04 | 1.81e-03 |
| ENSG00000080644 | 9.95e-11 | 9.31e-10 |
| ENSG00000080709 | 8.52e-01 | 8.88e-01 |
| ENSG00000080802 | 2.40e-01 | 3.16e-01 |
| ENSG00000080815 | 4.77e-11 | 4.73e-10 |
| ENSG00000080819 | 4.68e-17 | 1.50e-15 |
| ENSG00000080822 | 5.28e-01 | 6.07e-01 |
| ENSG00000080823 | 2.61e-07 | 1.37e-06 |
| ENSG00000080824 | 9.74e-10 | 7.71e-09 |
| ENSG00000080839 | 4.99e-14 | 8.56e-13 |
| ENSG00000080845 | 2.08e-16 | 5.80e-15 |
| ENSG00000080854 | 5.94e-01 | 6.68e-01 |
| ENSG00000080947 | 2.68e-02 | 4.84e-02 |
| ENSG00000080986 | 2.33e-05 | 8.59e-05 |
| ENSG00000081014 | 3.16e-04 | 9.31e-04 |
| ENSG00000081019 | 8.29e-05 | 2.74e-04 |
| ENSG00000081026 | 4.86e-12 | 5.76e-11 |
| ENSG00000081041 | 2.15e-13 | 3.28e-12 |
| ENSG00000081051 | 3.62e-09 | 2.60e-08 |
| ENSG00000081052 | 1.29e-05 | 5.02e-05 |
| ENSG00000081059 | 2.92e-20 | 2.04e-18 |
| ENSG00000081087 | 1.95e-06 | 8.72e-06 |
| ENSG00000081138 | 1.56e-01 | 2.20e-01 |
| ENSG00000081148 | 7.55e-01 | 8.09e-01 |
| ENSG00000081154 | 8.27e-01 | 8.68e-01 |
| ENSG00000081177 | 1.86e-02 | 3.52e-02 |
| ENSG00000081181 | 1.29e-08 | 8.46e-08 |
| ENSG00000081189 | 1.05e-14 | 2.05e-13 |
| ENSG00000081237 | 2.63e-06 | 1.15e-05 |
| ENSG00000081248 | 7.45e-01 | 8.00e-01 |
| ENSG00000081277 | 3.22e-21 | 3.05e-19 |
| ENSG00000081307 | 9.84e-02 | 1.48e-01 |
| ENSG00000081320 | 1.45e-08 | 9.43e-08 |
| ENSG00000081377 | 2.54e-01 | 3.31e-01 |
| ENSG00000081386 | 5.98e-02 | 9.68e-02 |
| ENSG00000081479 | 2.02e-05 | 7.54e-05 |
| ENSG00000081665 | 3.36e-01 | 4.17e-01 |
| ENSG00000081692 | 1.89e-08 | 1.20e-07 |
| ENSG00000081721 | 4.01e-09 | 2.87e-08 |
| ENSG00000081760 | 1.30e-02 | 2.57e-02 |
| ENSG00000081791 | 6.80e-03 | 1.45e-02 |
| ENSG00000081800 | 8.72e-16 | 2.10e-14 |
| ENSG00000081803 | 4.75e-04 | 1.35e-03 |
| ENSG00000081818 | 5.26e-07 | 2.61e-06 |
| ENSG00000081842 | 4.60e-03 | 1.03e-02 |
| ENSG00000081853 | 4.18e-01 | 5.01e-01 |
| ENSG00000081870 | 6.45e-01 | 7.14e-01 |
| ENSG00000081913 | 1.30e-05 | 5.04e-05 |
| ENSG00000081923 | 1.25e-19 | 7.36e-18 |
| ENSG00000081985 | 3.03e-02 | 5.36e-02 |
| ENSG00000082014 | 2.88e-05 | 1.05e-04 |
| ENSG00000082068 | 7.78e-03 | 1.64e-02 |
| ENSG00000082074 | 5.07e-06 | 2.11e-05 |
| ENSG00000082126 | 5.62e-10 | 4.64e-09 |
| ENSG00000082146 | 5.17e-11 | 5.08e-10 |
| ENSG00000082153 | 5.53e-01 | 6.30e-01 |
| ENSG00000082175 | 2.77e-14 | 4.99e-13 |
| ENSG00000082196 | 7.50e-08 | 4.32e-07 |
| ENSG00000082212 | 4.76e-12 | 5.65e-11 |
| ENSG00000082213 | 3.08e-01 | 3.89e-01 |
| ENSG00000082258 | 7.40e-01 | 7.96e-01 |
| ENSG00000082269 | 7.45e-03 | 1.57e-02 |
| ENSG00000082293 | 3.24e-14 | 5.74e-13 |
| ENSG00000082397 | 8.66e-25 | 2.55e-22 |
| ENSG00000082438 | 5.46e-02 | 8.94e-02 |
| ENSG00000082458 | 6.51e-01 | 7.19e-01 |
| ENSG00000082482 | 2.06e-10 | 1.83e-09 |
| ENSG00000082497 | 2.89e-12 | 3.59e-11 |
| ENSG00000082512 | 3.18e-19 | 1.70e-17 |
| ENSG00000082515 | 4.37e-04 | 1.25e-03 |
| ENSG00000082516 | 3.48e-14 | 6.12e-13 |
| ENSG00000082556 | 7.69e-01 | 8.21e-01 |
| ENSG00000082641 | 8.48e-15 | 1.68e-13 |
| ENSG00000082684 | 3.73e-09 | 2.68e-08 |
| ENSG00000082701 | 1.08e-04 | 3.51e-04 |
| ENSG00000082781 | 3.92e-04 | 1.13e-03 |
| ENSG00000082805 | 5.55e-01 | 6.32e-01 |
| ENSG00000082898 | 2.65e-06 | 1.16e-05 |
| ENSG00000082929 | 1.26e-01 | 1.83e-01 |
| ENSG00000082996 | 3.28e-01 | 4.10e-01 |
| ENSG00000083067 | 6.37e-02 | 1.02e-01 |
| ENSG00000083093 | 2.48e-18 | 1.05e-16 |
| ENSG00000083097 | 1.03e-01 | 1.54e-01 |
| ENSG00000083099 | 5.00e-04 | 1.41e-03 |
| ENSG00000083123 | 4.25e-03 | 9.60e-03 |
| ENSG00000083168 | 1.80e-01 | 2.48e-01 |
| ENSG00000083223 | 5.23e-01 | 6.03e-01 |
| ENSG00000083290 | 4.46e-03 | 1.00e-02 |
| ENSG00000083307 | 6.98e-07 | 3.40e-06 |
| ENSG00000083312 | 2.13e-03 | 5.19e-03 |
| ENSG00000083444 | 1.66e-10 | 1.50e-09 |
| ENSG00000083454 | 5.10e-03 | 1.13e-02 |
| ENSG00000083457 | 1.35e-02 | 2.66e-02 |
| ENSG00000083520 | 4.87e-10 | 4.05e-09 |
| ENSG00000083535 | 1.79e-07 | 9.64e-07 |
| ENSG00000083544 | 9.42e-01 | 9.57e-01 |
| ENSG00000083622 | 1.16e-03 | 3.01e-03 |
| ENSG00000083635 | 9.15e-20 | 5.60e-18 |
| ENSG00000083642 | 8.71e-03 | 1.81e-02 |
| ENSG00000083720 | 3.16e-03 | 7.38e-03 |
| ENSG00000083750 | 1.51e-02 | 2.92e-02 |
| ENSG00000083782 | 2.21e-21 | 2.23e-19 |
| ENSG00000083799 | 1.19e-07 | 6.65e-07 |
| ENSG00000083807 | 2.02e-11 | 2.13e-10 |
| ENSG00000083812 | 9.88e-01 | 9.91e-01 |
| ENSG00000083814 | 2.64e-11 | 2.73e-10 |
| ENSG00000083817 | 9.39e-02 | 1.43e-01 |
| ENSG00000083828 | 4.08e-01 | 4.91e-01 |
| ENSG00000083838 | 2.07e-01 | 2.79e-01 |
| ENSG00000083844 | 7.42e-04 | 2.02e-03 |
| ENSG00000083845 | 4.59e-06 | 1.92e-05 |
| ENSG00000083857 | 2.01e-03 | 4.93e-03 |
| ENSG00000083896 | 2.65e-01 | 3.43e-01 |
| ENSG00000083937 | 1.49e-07 | 8.14e-07 |
| ENSG00000084070 | 5.36e-05 | 1.84e-04 |
| ENSG00000084072 | 4.26e-05 | 1.50e-04 |
| ENSG00000084073 | 7.97e-02 | 1.24e-01 |
| ENSG00000084090 | 6.22e-03 | 1.34e-02 |
| ENSG00000084092 | 5.19e-03 | 1.15e-02 |
| ENSG00000084093 | 2.09e-01 | 2.81e-01 |
| ENSG00000084110 | 4.57e-01 | 5.39e-01 |
| ENSG00000084112 | 5.87e-01 | 6.61e-01 |
| ENSG00000084207 | 8.06e-11 | 7.69e-10 |
| ENSG00000084234 | 6.65e-09 | 4.58e-08 |
| ENSG00000084444 | 8.29e-01 | 8.70e-01 |
| ENSG00000084453 | 2.02e-06 | 9.00e-06 |
| ENSG00000084463 | 3.32e-05 | 1.19e-04 |
| ENSG00000084623 | 2.68e-07 | 1.40e-06 |
| ENSG00000084628 | 5.01e-03 | 1.11e-02 |
| ENSG00000084636 | 1.94e-04 | 5.96e-04 |
| ENSG00000084652 | 2.41e-06 | 1.06e-05 |
| ENSG00000084674 | 1.92e-04 | 5.93e-04 |
| ENSG00000084676 | 6.43e-13 | 8.98e-12 |
| ENSG00000084693 | 1.27e-02 | 2.51e-02 |
| ENSG00000084710 | 1.30e-02 | 2.56e-02 |
| ENSG00000084731 | 2.86e-04 | 8.49e-04 |
| ENSG00000084733 | 9.81e-01 | 9.87e-01 |
| ENSG00000084734 | 2.18e-06 | 9.66e-06 |
| ENSG00000084754 | 1.23e-15 | 2.89e-14 |
| ENSG00000084764 | 9.27e-07 | 4.42e-06 |
| ENSG00000084774 | 4.42e-21 | 3.95e-19 |
| ENSG00000085063 | 1.41e-03 | 3.59e-03 |
| ENSG00000085117 | 2.04e-09 | 1.53e-08 |
| ENSG00000085185 | 1.07e-03 | 2.80e-03 |
| ENSG00000085224 | 1.44e-02 | 2.80e-02 |
| ENSG00000085231 | 6.18e-01 | 6.89e-01 |
| ENSG00000085265 | 5.13e-03 | 1.13e-02 |
| ENSG00000085274 | 2.47e-01 | 3.24e-01 |
| ENSG00000085276 | 7.43e-01 | 7.99e-01 |
| ENSG00000085365 | 9.43e-01 | 9.58e-01 |
| ENSG00000085377 | 1.28e-04 | 4.08e-04 |
| ENSG00000085382 | 9.93e-01 | 9.95e-01 |
| ENSG00000085415 | 5.47e-07 | 2.70e-06 |
| ENSG00000085433 | 5.10e-02 | 8.44e-02 |
| ENSG00000085449 | 3.25e-02 | 5.71e-02 |
| ENSG00000085465 | 4.19e-22 | 5.32e-20 |
| ENSG00000085491 | 1.48e-08 | 9.61e-08 |
| ENSG00000085511 | 5.22e-04 | 1.47e-03 |
| ENSG00000085514 | 4.98e-01 | 5.79e-01 |
| ENSG00000085552 | 2.24e-12 | 2.84e-11 |
| ENSG00000085563 | 4.65e-11 | 4.62e-10 |
| ENSG00000085644 | 3.40e-01 | 4.22e-01 |
| ENSG00000085662 | 9.63e-08 | 5.44e-07 |
| ENSG00000085719 | 3.65e-02 | 6.31e-02 |
| ENSG00000085721 | 1.14e-04 | 3.68e-04 |
| ENSG00000085733 | 9.95e-02 | 1.50e-01 |
| ENSG00000085741 | 2.33e-03 | 5.63e-03 |
| ENSG00000085760 | 3.76e-01 | 4.59e-01 |
| ENSG00000085788 | 1.54e-01 | 2.17e-01 |
| ENSG00000085831 | 2.04e-01 | 2.75e-01 |
| ENSG00000085832 | 7.50e-03 | 1.58e-02 |
| ENSG00000085840 | 1.52e-10 | 1.38e-09 |
| ENSG00000085871 | 4.58e-03 | 1.03e-02 |
| ENSG00000085872 | 1.11e-06 | 5.21e-06 |
| ENSG00000085978 | 6.67e-09 | 4.58e-08 |
| ENSG00000085982 | 3.09e-10 | 2.66e-09 |
| ENSG00000085998 | 1.68e-09 | 1.28e-08 |
| ENSG00000085999 | 4.51e-16 | 1.17e-14 |
| ENSG00000086015 | 2.60e-10 | 2.28e-09 |
| ENSG00000086061 | 5.55e-11 | 5.43e-10 |
| ENSG00000086062 | 2.41e-02 | 4.39e-02 |
| ENSG00000086065 | 9.72e-04 | 2.57e-03 |
| ENSG00000086102 | 7.98e-05 | 2.65e-04 |
| ENSG00000086159 | 8.34e-08 | 4.76e-07 |
| ENSG00000086189 | 3.73e-10 | 3.17e-09 |
| ENSG00000086200 | 5.27e-11 | 5.19e-10 |
| ENSG00000086205 | 2.09e-05 | 7.78e-05 |
| ENSG00000086232 | 1.46e-08 | 9.46e-08 |
| ENSG00000086288 | 7.65e-01 | 8.17e-01 |
| ENSG00000086289 | 5.79e-05 | 1.98e-04 |
| ENSG00000086300 | 3.60e-06 | 1.54e-05 |
| ENSG00000086475 | 1.61e-12 | 2.10e-11 |
| ENSG00000086504 | 2.41e-01 | 3.16e-01 |
| ENSG00000086506 | 1.45e-04 | 4.59e-04 |
| ENSG00000086544 | 4.94e-01 | 5.75e-01 |
| ENSG00000086548 | 1.61e-04 | 5.04e-04 |
| ENSG00000086570 | 2.03e-03 | 4.98e-03 |
| ENSG00000086589 | 4.37e-02 | 7.37e-02 |
| ENSG00000086598 | 9.29e-03 | 1.91e-02 |
| ENSG00000086619 | 5.05e-05 | 1.75e-04 |
| ENSG00000086666 | 4.03e-05 | 1.42e-04 |
| ENSG00000086696 | 1.95e-21 | 2.01e-19 |
| ENSG00000086712 | 5.32e-13 | 7.56e-12 |
| ENSG00000086717 | 4.49e-13 | 6.49e-12 |
| ENSG00000086730 | 1.40e-03 | 3.58e-03 |
| ENSG00000086758 | 6.09e-08 | 3.56e-07 |
| ENSG00000086827 | 1.79e-06 | 8.06e-06 |
| ENSG00000086848 | 2.34e-01 | 3.09e-01 |
| ENSG00000086967 | 2.82e-07 | 1.47e-06 |
| ENSG00000086991 | 9.58e-18 | 3.55e-16 |
| ENSG00000087008 | 2.95e-06 | 1.28e-05 |
| ENSG00000087053 | 6.24e-01 | 6.95e-01 |
| ENSG00000087074 | 8.73e-01 | 9.05e-01 |
| ENSG00000087076 | 1.25e-03 | 3.24e-03 |
| ENSG00000087077 | 2.22e-11 | 2.33e-10 |
| ENSG00000087085 | 1.37e-09 | 1.06e-08 |
| ENSG00000087086 | 3.81e-01 | 4.64e-01 |
| ENSG00000087087 | 1.24e-09 | 9.67e-09 |
| ENSG00000087088 | 4.50e-03 | 1.01e-02 |
| ENSG00000087095 | 7.06e-01 | 7.67e-01 |
| ENSG00000087111 | 2.13e-11 | 2.24e-10 |
| ENSG00000087116 | 2.39e-19 | 1.31e-17 |
| ENSG00000087128 | 9.49e-01 | 9.63e-01 |
| ENSG00000087152 | 1.62e-01 | 2.27e-01 |
| ENSG00000087157 | 5.65e-06 | 2.33e-05 |
| ENSG00000087191 | 2.03e-01 | 2.74e-01 |
| ENSG00000087206 | 1.76e-03 | 4.38e-03 |
| ENSG00000087237 | 1.41e-05 | 5.43e-05 |
| ENSG00000087245 | 5.72e-02 | 9.31e-02 |
| ENSG00000087250 | 4.92e-08 | 2.93e-07 |
| ENSG00000087253 | 1.26e-06 | 5.85e-06 |
| ENSG00000087258 | 4.42e-16 | 1.15e-14 |
| ENSG00000087263 | 6.26e-17 | 1.94e-15 |
| ENSG00000087266 | 3.50e-02 | 6.08e-02 |
| ENSG00000087269 | 1.11e-14 | 2.16e-13 |
| ENSG00000087274 | 1.51e-16 | 4.31e-15 |
| ENSG00000087299 | 3.20e-03 | 7.47e-03 |
| ENSG00000087301 | 3.51e-03 | 8.12e-03 |
| ENSG00000087302 | 1.31e-01 | 1.90e-01 |
| ENSG00000087303 | 1.15e-09 | 8.98e-09 |
| ENSG00000087338 | 3.09e-01 | 3.89e-01 |
| ENSG00000087365 | 1.97e-01 | 2.68e-01 |
| ENSG00000087448 | 1.10e-01 | 1.64e-01 |
| ENSG00000087470 | 1.40e-02 | 2.74e-02 |
| ENSG00000087494 | 2.01e-01 | 2.72e-01 |
| ENSG00000087495 | 3.64e-02 | 6.29e-02 |
| ENSG00000087502 | 4.86e-04 | 1.38e-03 |
| ENSG00000087510 | 2.89e-04 | 8.57e-04 |
| ENSG00000087586 | 4.12e-16 | 1.07e-14 |
| ENSG00000087589 | 2.16e-03 | 5.26e-03 |
| ENSG00000087842 | 2.13e-02 | 3.94e-02 |
| ENSG00000087884 | 1.09e-05 | 4.29e-05 |
| ENSG00000087903 | 3.61e-02 | 6.25e-02 |
| ENSG00000087995 | 3.11e-04 | 9.16e-04 |
| ENSG00000088002 | 8.73e-22 | 1.00e-19 |
| ENSG00000088035 | 8.92e-01 | 9.20e-01 |
| ENSG00000088038 | 1.60e-04 | 5.01e-04 |
| ENSG00000088053 | 2.87e-01 | 3.67e-01 |
| ENSG00000088179 | 2.88e-02 | 5.14e-02 |
| ENSG00000088205 | 2.20e-12 | 2.80e-11 |
| ENSG00000088247 | 1.07e-11 | 1.19e-10 |
| ENSG00000088256 | 1.70e-20 | 1.28e-18 |
| ENSG00000088280 | 1.24e-18 | 5.70e-17 |
| ENSG00000088298 | 3.99e-05 | 1.41e-04 |
| ENSG00000088305 | 9.07e-12 | 1.02e-10 |
| ENSG00000088320 | 1.06e-05 | 4.17e-05 |
| ENSG00000088325 | 3.06e-18 | 1.28e-16 |
| ENSG00000088340 | 2.60e-10 | 2.27e-09 |
| ENSG00000088356 | 1.10e-14 | 2.14e-13 |
| ENSG00000088367 | 3.33e-01 | 4.14e-01 |
| ENSG00000088386 | 2.86e-09 | 2.09e-08 |
| ENSG00000088387 | 1.17e-02 | 2.33e-02 |
| ENSG00000088448 | 1.76e-12 | 2.28e-11 |
| ENSG00000088451 | 1.91e-15 | 4.29e-14 |
| ENSG00000088538 | 5.82e-05 | 1.99e-04 |
| ENSG00000088543 | 1.34e-07 | 7.39e-07 |
| ENSG00000088682 | 3.75e-07 | 1.91e-06 |
| ENSG00000088726 | 2.48e-11 | 2.58e-10 |
| ENSG00000088727 | 2.46e-14 | 4.49e-13 |
| ENSG00000088756 | 5.33e-11 | 5.23e-10 |
| ENSG00000088766 | 3.51e-09 | 2.52e-08 |
| ENSG00000088808 | 3.26e-06 | 1.40e-05 |
| ENSG00000088812 | 3.84e-02 | 6.60e-02 |
| ENSG00000088826 | 5.67e-25 | 1.83e-22 |
| ENSG00000088827 | 3.40e-11 | 3.45e-10 |
| ENSG00000088832 | 1.62e-03 | 4.06e-03 |
| ENSG00000088833 | 1.00e+00 | 1.00e+00 |
| ENSG00000088836 | 9.93e-22 | 1.11e-19 |
| ENSG00000088854 | 2.60e-11 | 2.69e-10 |
| ENSG00000088876 | 1.47e-13 | 2.32e-12 |
| ENSG00000088881 | 3.68e-05 | 1.31e-04 |
| ENSG00000088882 | 1.22e-11 | 1.34e-10 |
| ENSG00000088888 | 7.71e-04 | 2.09e-03 |
| ENSG00000088899 | 4.33e-19 | 2.24e-17 |
| ENSG00000088926 | 8.24e-01 | 8.66e-01 |
| ENSG00000088930 | 3.69e-12 | 4.48e-11 |
| ENSG00000088970 | 4.97e-01 | 5.77e-01 |
| ENSG00000088986 | 5.03e-01 | 5.83e-01 |
| ENSG00000088992 | 1.09e-23 | 2.31e-21 |
| ENSG00000089006 | 1.89e-03 | 4.67e-03 |
| ENSG00000089009 | 4.20e-01 | 5.03e-01 |
| ENSG00000089012 | 7.62e-07 | 3.68e-06 |
| ENSG00000089022 | 1.83e-07 | 9.84e-07 |
| ENSG00000089041 | 5.99e-06 | 2.46e-05 |
| ENSG00000089048 | 1.89e-04 | 5.83e-04 |
| ENSG00000089050 | 6.84e-04 | 1.88e-03 |
| ENSG00000089053 | 1.35e-11 | 1.47e-10 |
| ENSG00000089057 | 8.65e-02 | 1.33e-01 |
| ENSG00000089060 | 1.01e-05 | 3.98e-05 |
| ENSG00000089063 | 1.57e-01 | 2.21e-01 |
| ENSG00000089091 | 4.63e-04 | 1.32e-03 |
| ENSG00000089094 | 3.69e-09 | 2.65e-08 |
| ENSG00000089101 | 2.10e-02 | 3.90e-02 |
| ENSG00000089116 | 3.17e-11 | 3.24e-10 |
| ENSG00000089123 | 1.16e-11 | 1.27e-10 |
| ENSG00000089127 | 5.09e-02 | 8.42e-02 |
| ENSG00000089154 | 2.32e-09 | 1.72e-08 |
| ENSG00000089157 | 2.10e-05 | 7.83e-05 |
| ENSG00000089159 | 1.22e-03 | 3.17e-03 |
| ENSG00000089163 | 5.94e-06 | 2.44e-05 |
| ENSG00000089169 | 7.66e-16 | 1.88e-14 |
| ENSG00000089177 | 2.32e-04 | 7.03e-04 |
| ENSG00000089195 | 2.66e-16 | 7.26e-15 |
| ENSG00000089199 | 1.16e-14 | 2.24e-13 |
| ENSG00000089220 | 3.06e-01 | 3.86e-01 |
| ENSG00000089225 | 9.92e-06 | 3.93e-05 |
| ENSG00000089234 | 1.40e-02 | 2.74e-02 |
| ENSG00000089248 | 2.48e-02 | 4.50e-02 |
| ENSG00000089250 | 9.67e-08 | 5.46e-07 |
| ENSG00000089280 | 6.32e-16 | 1.58e-14 |
| ENSG00000089289 | 1.49e-03 | 3.78e-03 |
| ENSG00000089327 | 8.21e-23 | 1.28e-20 |
| ENSG00000089335 | 2.09e-01 | 2.80e-01 |
| ENSG00000089351 | 6.68e-22 | 7.97e-20 |
| ENSG00000089356 | 1.46e-13 | 2.31e-12 |
| ENSG00000089472 | 1.86e-06 | 8.37e-06 |
| ENSG00000089486 | 1.12e-10 | 1.04e-09 |
| ENSG00000089505 | 8.95e-08 | 5.09e-07 |
| ENSG00000089558 | 4.48e-11 | 4.47e-10 |
| ENSG00000089597 | 1.42e-04 | 4.50e-04 |
| ENSG00000089639 | 1.28e-01 | 1.86e-01 |
| ENSG00000089682 | 1.17e-02 | 2.34e-02 |
| ENSG00000089685 | 5.15e-15 | 1.07e-13 |
| ENSG00000089692 | 2.33e-09 | 1.73e-08 |
| ENSG00000089693 | 1.11e-06 | 5.22e-06 |
| ENSG00000089723 | 2.75e-16 | 7.47e-15 |
| ENSG00000089737 | 6.07e-01 | 6.79e-01 |
| ENSG00000089775 | 1.56e-05 | 5.94e-05 |
| ENSG00000089818 | 1.61e-02 | 3.08e-02 |
| ENSG00000089820 | 1.17e-01 | 1.71e-01 |
| ENSG00000089847 | 5.65e-04 | 1.58e-03 |
| ENSG00000089876 | 1.99e-03 | 4.89e-03 |
| ENSG00000089902 | 3.46e-02 | 6.03e-02 |
| ENSG00000089916 | 1.54e-01 | 2.17e-01 |
| ENSG00000090006 | 6.46e-16 | 1.62e-14 |
| ENSG00000090013 | 5.51e-02 | 9.00e-02 |
| ENSG00000090020 | 8.62e-15 | 1.71e-13 |
| ENSG00000090054 | 7.89e-01 | 8.37e-01 |
| ENSG00000090060 | 6.84e-01 | 7.48e-01 |
| ENSG00000090061 | 2.47e-01 | 3.24e-01 |
| ENSG00000090097 | 3.17e-09 | 2.30e-08 |
| ENSG00000090104 | 2.54e-04 | 7.62e-04 |
| ENSG00000090238 | 2.25e-02 | 4.15e-02 |
| ENSG00000090263 | 1.83e-01 | 2.51e-01 |
| ENSG00000090266 | 2.77e-03 | 6.58e-03 |
| ENSG00000090273 | 2.66e-08 | 1.65e-07 |
| ENSG00000090316 | 1.79e-01 | 2.47e-01 |
| ENSG00000090339 | 4.29e-07 | 2.16e-06 |
| ENSG00000090372 | 2.14e-04 | 6.54e-04 |
| ENSG00000090376 | 2.09e-02 | 3.89e-02 |
| ENSG00000090382 | 4.92e-01 | 5.73e-01 |
| ENSG00000090402 | 1.13e-09 | 8.85e-09 |
| ENSG00000090432 | 1.92e-14 | 3.59e-13 |
| ENSG00000090447 | 4.33e-25 | 1.45e-22 |
| ENSG00000090470 | 4.78e-05 | 1.66e-04 |
| ENSG00000090487 | 1.85e-10 | 1.66e-09 |
| ENSG00000090512 | 3.31e-07 | 1.70e-06 |
| ENSG00000090520 | 8.68e-04 | 2.33e-03 |
| ENSG00000090530 | 4.31e-15 | 9.06e-14 |
| ENSG00000090534 | 6.74e-01 | 7.39e-01 |
| ENSG00000090539 | 7.92e-01 | 8.39e-01 |
| ENSG00000090554 | 7.55e-10 | 6.09e-09 |
| ENSG00000090565 | 2.54e-08 | 1.58e-07 |
| ENSG00000090581 | 4.48e-01 | 5.31e-01 |
| ENSG00000090612 | 3.84e-01 | 4.67e-01 |
| ENSG00000090615 | 2.87e-01 | 3.67e-01 |
| ENSG00000090621 | 6.76e-10 | 5.48e-09 |
| ENSG00000090659 | 4.07e-16 | 1.06e-14 |
| ENSG00000090661 | 6.50e-06 | 2.65e-05 |
| ENSG00000090674 | 5.89e-04 | 1.64e-03 |
| ENSG00000090686 | 4.12e-01 | 4.95e-01 |
| ENSG00000090776 | 3.93e-02 | 6.72e-02 |
| ENSG00000090857 | 9.91e-01 | 9.93e-01 |
| ENSG00000090861 | 8.36e-15 | 1.66e-13 |
| ENSG00000090863 | 3.38e-06 | 1.45e-05 |
| ENSG00000090889 | 1.02e-16 | 3.02e-15 |
| ENSG00000090905 | 4.06e-01 | 4.89e-01 |
| ENSG00000090924 | 2.35e-05 | 8.68e-05 |
| ENSG00000090932 | 2.48e-03 | 5.95e-03 |
| ENSG00000090971 | 1.90e-04 | 5.86e-04 |
| ENSG00000090975 | 2.74e-02 | 4.92e-02 |
| ENSG00000090989 | 9.39e-01 | 9.55e-01 |
| ENSG00000091009 | 8.19e-01 | 8.62e-01 |
| ENSG00000091010 | 3.32e-01 | 4.13e-01 |
| ENSG00000091039 | 1.66e-02 | 3.18e-02 |
| ENSG00000091073 | 1.76e-08 | 1.13e-07 |
| ENSG00000091106 | 7.71e-02 | 1.20e-01 |
| ENSG00000091127 | 2.61e-24 | 6.53e-22 |
| ENSG00000091128 | 5.86e-01 | 6.61e-01 |
| ENSG00000091129 | 5.43e-02 | 8.90e-02 |
| ENSG00000091136 | 5.66e-06 | 2.33e-05 |
| ENSG00000091137 | 1.37e-05 | 5.30e-05 |
| ENSG00000091138 | 2.43e-17 | 8.27e-16 |
| ENSG00000091140 | 1.15e-06 | 5.37e-06 |
| ENSG00000091157 | 4.81e-17 | 1.54e-15 |
| ENSG00000091164 | 1.49e-02 | 2.88e-02 |
| ENSG00000091181 | 7.72e-07 | 3.73e-06 |
| ENSG00000091262 | 2.06e-03 | 5.04e-03 |
| ENSG00000091317 | 6.90e-02 | 1.09e-01 |
| ENSG00000091409 | 1.15e-07 | 6.43e-07 |
| ENSG00000091428 | 1.57e-06 | 7.15e-06 |
| ENSG00000091436 | 2.21e-04 | 6.73e-04 |
| ENSG00000091482 | 2.88e-01 | 3.68e-01 |
| ENSG00000091483 | 4.69e-05 | 1.63e-04 |
| ENSG00000091490 | 2.55e-02 | 4.63e-02 |
| ENSG00000091513 | 4.94e-08 | 2.94e-07 |
| ENSG00000091527 | 2.47e-02 | 4.50e-02 |
| ENSG00000091536 | 2.07e-01 | 2.79e-01 |
| ENSG00000091542 | 2.23e-06 | 9.87e-06 |
| ENSG00000091583 | 6.76e-04 | 1.86e-03 |
| ENSG00000091592 | 3.21e-04 | 9.44e-04 |
| ENSG00000091622 | 1.80e-13 | 2.79e-12 |
| ENSG00000091640 | 1.18e-07 | 6.57e-07 |
| ENSG00000091651 | 1.47e-24 | 4.11e-22 |
| ENSG00000091656 | 2.20e-01 | 2.93e-01 |
| ENSG00000091704 | 3.15e-01 | 3.96e-01 |
| ENSG00000091732 | 7.22e-08 | 4.17e-07 |
| ENSG00000091831 | 2.64e-09 | 1.94e-08 |
| ENSG00000091844 | 7.87e-02 | 1.22e-01 |
| ENSG00000091879 | 2.38e-14 | 4.37e-13 |
| ENSG00000091947 | 6.50e-02 | 1.04e-01 |
| ENSG00000091972 | 6.24e-01 | 6.95e-01 |
| ENSG00000091986 | 9.77e-12 | 1.09e-10 |
| ENSG00000092009 | 3.87e-20 | 2.62e-18 |
| ENSG00000092010 | 9.17e-01 | 9.38e-01 |
| ENSG00000092020 | 8.12e-05 | 2.69e-04 |
| ENSG00000092036 | 2.98e-01 | 3.78e-01 |
| ENSG00000092051 | 2.32e-19 | 1.28e-17 |
| ENSG00000092054 | 4.27e-02 | 7.23e-02 |
| ENSG00000092067 | 1.53e-01 | 2.16e-01 |
| ENSG00000092068 | 2.76e-02 | 4.96e-02 |
| ENSG00000092094 | 7.70e-04 | 2.09e-03 |
| ENSG00000092096 | 9.82e-14 | 1.60e-12 |
| ENSG00000092098 | 7.83e-01 | 8.32e-01 |
| ENSG00000092108 | 7.68e-02 | 1.20e-01 |
| ENSG00000092140 | 1.07e-03 | 2.80e-03 |
| ENSG00000092148 | 6.38e-01 | 7.08e-01 |
| ENSG00000092199 | 8.95e-05 | 2.94e-04 |
| ENSG00000092200 | 2.02e-04 | 6.20e-04 |
| ENSG00000092201 | 3.91e-06 | 1.66e-05 |
| ENSG00000092203 | 3.58e-06 | 1.53e-05 |
| ENSG00000092208 | 4.09e-04 | 1.18e-03 |
| ENSG00000092295 | 2.98e-04 | 8.82e-04 |
| ENSG00000092330 | 2.99e-06 | 1.29e-05 |
| ENSG00000092345 | 1.78e-01 | 2.45e-01 |
| ENSG00000092377 | 9.28e-05 | 3.04e-04 |
| ENSG00000092421 | 1.17e-21 | 1.29e-19 |
| ENSG00000092439 | 6.41e-03 | 1.38e-02 |
| ENSG00000092445 | 3.91e-14 | 6.84e-13 |
| ENSG00000092470 | 1.74e-06 | 7.85e-06 |
| ENSG00000092529 | 8.92e-01 | 9.19e-01 |
| ENSG00000092531 | 4.23e-05 | 1.49e-04 |
| ENSG00000092607 | 3.01e-17 | 1.00e-15 |
| ENSG00000092621 | 1.96e-06 | 8.77e-06 |
| ENSG00000092758 | 2.03e-06 | 9.06e-06 |
| ENSG00000092820 | 1.18e-03 | 3.06e-03 |
| ENSG00000092841 | 6.86e-08 | 3.98e-07 |
| ENSG00000092847 | 8.44e-02 | 1.30e-01 |
| ENSG00000092850 | 5.04e-04 | 1.42e-03 |
| ENSG00000092853 | 2.28e-11 | 2.38e-10 |
| ENSG00000092871 | 6.86e-01 | 7.50e-01 |
| ENSG00000092929 | 2.88e-02 | 5.14e-02 |
| ENSG00000092931 | 7.35e-14 | 1.22e-12 |
| ENSG00000092964 | 9.46e-04 | 2.51e-03 |
| ENSG00000092969 | 5.89e-04 | 1.64e-03 |
| ENSG00000092978 | 3.47e-09 | 2.50e-08 |
| ENSG00000093000 | 3.16e-01 | 3.97e-01 |
| ENSG00000093009 | 9.48e-18 | 3.52e-16 |
| ENSG00000093010 | 6.30e-11 | 6.11e-10 |
| ENSG00000093072 | 2.20e-12 | 2.79e-11 |
| ENSG00000093100 | 2.53e-01 | 3.30e-01 |
| ENSG00000093134 | 2.67e-05 | 9.78e-05 |
| ENSG00000093144 | 3.21e-06 | 1.38e-05 |
| ENSG00000093167 | 1.71e-11 | 1.82e-10 |
| ENSG00000093183 | 3.38e-01 | 4.20e-01 |
| ENSG00000093217 | 1.32e-03 | 3.41e-03 |
| ENSG00000094631 | 1.16e-01 | 1.70e-01 |
| ENSG00000094661 | 1.31e-02 | 2.58e-02 |
| ENSG00000094755 | 4.82e-06 | 2.02e-05 |
| ENSG00000094796 | 4.06e-18 | 1.63e-16 |
| ENSG00000094804 | 6.85e-18 | 2.62e-16 |
| ENSG00000094841 | 4.77e-01 | 5.59e-01 |
| ENSG00000094880 | 5.59e-04 | 1.57e-03 |
| ENSG00000094914 | 3.02e-10 | 2.61e-09 |
| ENSG00000094916 | 2.28e-04 | 6.93e-04 |
| ENSG00000094963 | 2.33e-10 | 2.06e-09 |
| ENSG00000094975 | 6.93e-05 | 2.33e-04 |
| ENSG00000095002 | 8.95e-13 | 1.22e-11 |
| ENSG00000095015 | 9.66e-01 | 9.75e-01 |
| ENSG00000095059 | 1.29e-04 | 4.13e-04 |
| ENSG00000095066 | 9.71e-04 | 2.57e-03 |
| ENSG00000095110 | 3.97e-14 | 6.94e-13 |
| ENSG00000095139 | 6.71e-01 | 7.37e-01 |
| ENSG00000095203 | 5.85e-14 | 9.89e-13 |
| ENSG00000095209 | 1.02e-01 | 1.53e-01 |
| ENSG00000095261 | 3.13e-04 | 9.22e-04 |
| ENSG00000095303 | 2.05e-15 | 4.57e-14 |
| ENSG00000095319 | 7.06e-16 | 1.74e-14 |
| ENSG00000095321 | 6.16e-09 | 4.26e-08 |
| ENSG00000095370 | 5.94e-02 | 9.61e-02 |
| ENSG00000095380 | 3.22e-09 | 2.33e-08 |
| ENSG00000095383 | 4.93e-01 | 5.74e-01 |
| ENSG00000095397 | 5.04e-07 | 2.51e-06 |
| ENSG00000095464 | 4.80e-01 | 5.63e-01 |
| ENSG00000095485 | 4.55e-09 | 3.22e-08 |
| ENSG00000095539 | 6.94e-08 | 4.02e-07 |
| ENSG00000095564 | 3.94e-04 | 1.14e-03 |
| ENSG00000095574 | 8.84e-01 | 9.13e-01 |
| ENSG00000095585 | 4.90e-07 | 2.45e-06 |
| ENSG00000095587 | 5.80e-01 | 6.55e-01 |
| ENSG00000095596 | 6.53e-06 | 2.67e-05 |
| ENSG00000095627 | 6.25e-01 | 6.96e-01 |
| ENSG00000095637 | 3.92e-07 | 1.99e-06 |
| ENSG00000095713 | 6.87e-08 | 3.99e-07 |
| ENSG00000095739 | 1.39e-06 | 6.38e-06 |
| ENSG00000095752 | 1.58e-24 | 4.29e-22 |
| ENSG00000095777 | 1.00e-09 | 7.90e-09 |
| ENSG00000095787 | 6.15e-01 | 6.87e-01 |
| ENSG00000095794 | 2.61e-01 | 3.38e-01 |
| ENSG00000095906 | 4.03e-07 | 2.04e-06 |
| ENSG00000095917 | 1.30e-06 | 5.99e-06 |
| ENSG00000095932 | 9.12e-01 | 9.35e-01 |
| ENSG00000095951 | 5.27e-01 | 6.06e-01 |
| ENSG00000095970 | 1.79e-05 | 6.75e-05 |
| ENSG00000095981 | 2.48e-01 | 3.24e-01 |
| ENSG00000096006 | 4.81e-01 | 5.63e-01 |
| ENSG00000096060 | 7.70e-09 | 5.24e-08 |
| ENSG00000096063 | 1.61e-09 | 1.23e-08 |
| ENSG00000096070 | 9.70e-12 | 1.09e-10 |
| ENSG00000096080 | 3.82e-01 | 4.65e-01 |
| ENSG00000096088 | 3.24e-12 | 3.97e-11 |
| ENSG00000096092 | 6.70e-03 | 1.43e-02 |
| ENSG00000096093 | 2.67e-06 | 1.16e-05 |
| ENSG00000096264 | 1.76e-09 | 1.33e-08 |
| ENSG00000096384 | 9.15e-19 | 4.36e-17 |
| ENSG00000096395 | 5.24e-01 | 6.03e-01 |
| ENSG00000096401 | 6.57e-03 | 1.41e-02 |
| ENSG00000096433 | 1.13e-02 | 2.28e-02 |
| ENSG00000096654 | 1.55e-04 | 4.86e-04 |
| ENSG00000096696 | 8.53e-02 | 1.31e-01 |
| ENSG00000096717 | 3.80e-03 | 8.71e-03 |
| ENSG00000096746 | 8.74e-03 | 1.81e-02 |
| ENSG00000096872 | 4.85e-02 | 8.07e-02 |
| ENSG00000096968 | 1.87e-03 | 4.63e-03 |
| ENSG00000096996 | 2.06e-02 | 3.84e-02 |
| ENSG00000097007 | 2.68e-05 | 9.81e-05 |
| ENSG00000097021 | 2.48e-02 | 4.52e-02 |
| ENSG00000097033 | 2.12e-08 | 1.34e-07 |
| ENSG00000097046 | 2.70e-10 | 2.36e-09 |
| ENSG00000097096 | 2.04e-01 | 2.75e-01 |
| ENSG00000099139 | 1.48e-22 | 2.06e-20 |
| ENSG00000099194 | 1.02e-18 | 4.78e-17 |
| ENSG00000099203 | 1.78e-04 | 5.53e-04 |
| ENSG00000099204 | 7.74e-01 | 8.24e-01 |
| ENSG00000099219 | 7.55e-01 | 8.09e-01 |
| ENSG00000099246 | 1.96e-05 | 7.36e-05 |
| ENSG00000099250 | 6.10e-02 | 9.84e-02 |
| ENSG00000099251 | 9.02e-10 | 7.18e-09 |
| ENSG00000099256 | 1.16e-02 | 2.33e-02 |
| ENSG00000099260 | 5.40e-03 | 1.19e-02 |
| ENSG00000099282 | 4.12e-01 | 4.94e-01 |
| ENSG00000099284 | 5.86e-01 | 6.61e-01 |
| ENSG00000099290 | 2.08e-10 | 1.85e-09 |
| ENSG00000099308 | 6.71e-09 | 4.61e-08 |
| ENSG00000099326 | 1.91e-06 | 8.57e-06 |
| ENSG00000099330 | 4.51e-12 | 5.38e-11 |
| ENSG00000099331 | 9.91e-01 | 9.93e-01 |
| ENSG00000099337 | 1.72e-04 | 5.35e-04 |
| ENSG00000099338 | 2.02e-08 | 1.28e-07 |
| ENSG00000099341 | 1.67e-04 | 5.21e-04 |
| ENSG00000099364 | 1.28e-06 | 5.93e-06 |
| ENSG00000099365 | 4.46e-01 | 5.29e-01 |
| ENSG00000099377 | 1.17e-05 | 4.56e-05 |
| ENSG00000099381 | 2.05e-07 | 1.09e-06 |
| ENSG00000099385 | 1.47e-02 | 2.85e-02 |
| ENSG00000099399 | 1.10e-01 | 1.64e-01 |
| ENSG00000099617 | 3.72e-04 | 1.08e-03 |
| ENSG00000099622 | 2.74e-05 | 1.00e-04 |
| ENSG00000099624 | 3.42e-01 | 4.24e-01 |
| ENSG00000099625 | 4.51e-14 | 7.79e-13 |
| ENSG00000099715 | 1.34e-13 | 2.13e-12 |
| ENSG00000099725 | 9.13e-01 | 9.36e-01 |
| ENSG00000099769 | 2.54e-01 | 3.31e-01 |
| ENSG00000099783 | 1.13e-06 | 5.30e-06 |
| ENSG00000099785 | 1.83e-07 | 9.86e-07 |
| ENSG00000099795 | 1.85e-01 | 2.54e-01 |
| ENSG00000099797 | 1.22e-05 | 4.74e-05 |
| ENSG00000099800 | 3.84e-05 | 1.36e-04 |
| ENSG00000099804 | 3.78e-07 | 1.92e-06 |
| ENSG00000099810 | 1.05e-11 | 1.17e-10 |
| ENSG00000099812 | 1.24e-07 | 6.90e-07 |
| ENSG00000099814 | 2.00e-04 | 6.14e-04 |
| ENSG00000099817 | 4.53e-03 | 1.02e-02 |
| ENSG00000099821 | 1.51e-04 | 4.76e-04 |
| ENSG00000099822 | 8.53e-01 | 8.89e-01 |
| ENSG00000099834 | 4.93e-16 | 1.27e-14 |
| ENSG00000099840 | 1.99e-03 | 4.90e-03 |
| ENSG00000099849 | 3.17e-01 | 3.98e-01 |
| ENSG00000099860 | 7.56e-02 | 1.18e-01 |
| ENSG00000099864 | 1.80e-14 | 3.38e-13 |
| ENSG00000099866 | 1.04e-16 | 3.08e-15 |
| ENSG00000099869 | 1.56e-04 | 4.88e-04 |
| ENSG00000099875 | 1.05e-08 | 6.94e-08 |
| ENSG00000099889 | 7.07e-11 | 6.81e-10 |
| ENSG00000099899 | 8.77e-08 | 4.99e-07 |
| ENSG00000099901 | 2.11e-18 | 9.10e-17 |
| ENSG00000099904 | 1.00e-10 | 9.37e-10 |
| ENSG00000099910 | 3.52e-01 | 4.35e-01 |
| ENSG00000099917 | 7.65e-02 | 1.20e-01 |
| ENSG00000099937 | 1.15e-08 | 7.57e-08 |
| ENSG00000099940 | 1.91e-12 | 2.46e-11 |
| ENSG00000099942 | 4.87e-02 | 8.10e-02 |
| ENSG00000099949 | 2.20e-02 | 4.06e-02 |
| ENSG00000099953 | 1.36e-21 | 1.49e-19 |
| ENSG00000099954 | 9.77e-01 | 9.83e-01 |
| ENSG00000099956 | 2.29e-02 | 4.21e-02 |
| ENSG00000099957 | 3.81e-07 | 1.94e-06 |
| ENSG00000099958 | 1.49e-09 | 1.14e-08 |
| ENSG00000099960 | 9.97e-01 | 9.98e-01 |
| ENSG00000099968 | 3.79e-07 | 1.93e-06 |
| ENSG00000099974 | 7.16e-01 | 7.75e-01 |
| ENSG00000099977 | 2.00e-09 | 1.50e-08 |
| ENSG00000099984 | 3.88e-01 | 4.71e-01 |
| ENSG00000099985 | 1.12e-13 | 1.80e-12 |
| ENSG00000099991 | 2.45e-01 | 3.21e-01 |
| ENSG00000099992 | 1.71e-12 | 2.22e-11 |
| ENSG00000099994 | 2.11e-01 | 2.83e-01 |
| ENSG00000099995 | 9.52e-01 | 9.65e-01 |
| ENSG00000099998 | 1.73e-01 | 2.40e-01 |
| ENSG00000099999 | 3.92e-09 | 2.80e-08 |
| ENSG00000100003 | 9.24e-20 | 5.64e-18 |
| ENSG00000100012 | 4.11e-01 | 4.94e-01 |
| ENSG00000100014 | 1.37e-18 | 6.23e-17 |
| ENSG00000100023 | 1.69e-01 | 2.35e-01 |
| ENSG00000100024 | 2.22e-01 | 2.95e-01 |
| ENSG00000100027 | 2.98e-02 | 5.29e-02 |
| ENSG00000100028 | 8.73e-07 | 4.18e-06 |
| ENSG00000100029 | 4.03e-12 | 4.86e-11 |
| ENSG00000100030 | 2.15e-04 | 6.55e-04 |
| ENSG00000100031 | 6.42e-04 | 1.77e-03 |
| ENSG00000100033 | 5.12e-01 | 5.92e-01 |
| ENSG00000100034 | 5.46e-04 | 1.53e-03 |
| ENSG00000100036 | 4.05e-11 | 4.07e-10 |
| ENSG00000100038 | 5.57e-01 | 6.34e-01 |
| ENSG00000100053 | 9.53e-01 | 9.65e-01 |
| ENSG00000100055 | 1.84e-06 | 8.29e-06 |
| ENSG00000100056 | 3.45e-01 | 4.27e-01 |
| ENSG00000100058 | 1.56e-06 | 7.10e-06 |
| ENSG00000100060 | 5.09e-10 | 4.23e-09 |
| ENSG00000100065 | 6.19e-01 | 6.91e-01 |
| ENSG00000100068 | 7.60e-07 | 3.67e-06 |
| ENSG00000100075 | 1.17e-01 | 1.72e-01 |
| ENSG00000100077 | 3.04e-02 | 5.38e-02 |
| ENSG00000100078 | 1.91e-10 | 1.71e-09 |
| ENSG00000100079 | 1.54e-13 | 2.42e-12 |
| ENSG00000100083 | 4.08e-01 | 4.91e-01 |
| ENSG00000100084 | 5.53e-05 | 1.90e-04 |
| ENSG00000100092 | 5.49e-10 | 4.54e-09 |
| ENSG00000100095 | 1.72e-14 | 3.24e-13 |
| ENSG00000100097 | 4.38e-01 | 5.21e-01 |
| ENSG00000100099 | 2.02e-12 | 2.58e-11 |
| ENSG00000100100 | 9.25e-01 | 9.45e-01 |
| ENSG00000100101 | 1.42e-02 | 2.77e-02 |
| ENSG00000100104 | 1.20e-02 | 2.39e-02 |
| ENSG00000100105 | 1.56e-12 | 2.04e-11 |
| ENSG00000100106 | 3.00e-10 | 2.59e-09 |
| ENSG00000100109 | 9.15e-02 | 1.39e-01 |
| ENSG00000100116 | 2.69e-04 | 8.03e-04 |
| ENSG00000100121 | 1.76e-01 | 2.43e-01 |
| ENSG00000100122 | 3.17e-05 | 1.14e-04 |
| ENSG00000100124 | 7.87e-01 | 8.35e-01 |
| ENSG00000100129 | 8.39e-02 | 1.29e-01 |
| ENSG00000100138 | 2.55e-09 | 1.88e-08 |
| ENSG00000100139 | 1.09e-03 | 2.84e-03 |
| ENSG00000100142 | 3.73e-05 | 1.32e-04 |
| ENSG00000100147 | 6.51e-05 | 2.20e-04 |
| ENSG00000100150 | 8.04e-07 | 3.87e-06 |
| ENSG00000100151 | 1.30e-01 | 1.88e-01 |
| ENSG00000100154 | 7.22e-11 | 6.94e-10 |
| ENSG00000100156 | 4.88e-04 | 1.38e-03 |
| ENSG00000100162 | 5.63e-05 | 1.93e-04 |
| ENSG00000100167 | 6.72e-01 | 7.38e-01 |
| ENSG00000100170 | 1.33e-04 | 4.22e-04 |
| ENSG00000100181 | 6.17e-05 | 2.09e-04 |
| ENSG00000100191 | 1.14e-01 | 1.69e-01 |
| ENSG00000100196 | 8.65e-10 | 6.90e-09 |
| ENSG00000100197 | 3.35e-01 | 4.17e-01 |
| ENSG00000100201 | 5.49e-01 | 6.26e-01 |
| ENSG00000100206 | 4.58e-02 | 7.67e-02 |
| ENSG00000100207 | 8.55e-08 | 4.88e-07 |
| ENSG00000100209 | 1.13e-01 | 1.67e-01 |
| ENSG00000100211 | 2.20e-02 | 4.07e-02 |
| ENSG00000100216 | 2.26e-01 | 3.00e-01 |
| ENSG00000100218 | 6.25e-12 | 7.25e-11 |
| ENSG00000100219 | 9.93e-01 | 9.95e-01 |
| ENSG00000100220 | 8.71e-03 | 1.81e-02 |
| ENSG00000100221 | 1.52e-13 | 2.39e-12 |
| ENSG00000100225 | 1.94e-01 | 2.64e-01 |
| ENSG00000100226 | 1.68e-02 | 3.21e-02 |
| ENSG00000100227 | 1.38e-01 | 1.98e-01 |
| ENSG00000100228 | 4.82e-17 | 1.54e-15 |
| ENSG00000100234 | 1.40e-01 | 2.00e-01 |
| ENSG00000100239 | 8.06e-01 | 8.51e-01 |
| ENSG00000100241 | 1.49e-03 | 3.78e-03 |
| ENSG00000100242 | 4.02e-12 | 4.85e-11 |
| ENSG00000100243 | 3.60e-02 | 6.23e-02 |
| ENSG00000100246 | 1.87e-01 | 2.56e-01 |
| ENSG00000100249 | 1.83e-01 | 2.51e-01 |
| ENSG00000100253 | 3.55e-08 | 2.16e-07 |
| ENSG00000100258 | 8.78e-01 | 9.09e-01 |
| ENSG00000100263 | 4.71e-07 | 2.36e-06 |
| ENSG00000100266 | 9.01e-21 | 7.47e-19 |
| ENSG00000100271 | 8.65e-01 | 8.99e-01 |
| ENSG00000100276 | 7.09e-02 | 1.12e-01 |
| ENSG00000100280 | 3.57e-05 | 1.27e-04 |
| ENSG00000100281 | 2.03e-04 | 6.23e-04 |
| ENSG00000100284 | 7.01e-02 | 1.11e-01 |
| ENSG00000100285 | 8.00e-11 | 7.64e-10 |
| ENSG00000100288 | 7.58e-02 | 1.19e-01 |
| ENSG00000100290 | 6.34e-03 | 1.36e-02 |
| ENSG00000100292 | 1.03e-11 | 1.15e-10 |
| ENSG00000100294 | 1.69e-04 | 5.27e-04 |
| ENSG00000100296 | 8.67e-06 | 3.47e-05 |
| ENSG00000100297 | 2.19e-08 | 1.38e-07 |
| ENSG00000100298 | 3.60e-01 | 4.42e-01 |
| ENSG00000100299 | 1.43e-09 | 1.10e-08 |
| ENSG00000100300 | 9.19e-01 | 9.40e-01 |
| ENSG00000100302 | 2.97e-14 | 5.31e-13 |
| ENSG00000100304 | 1.55e-01 | 2.18e-01 |
| ENSG00000100307 | 5.94e-21 | 5.11e-19 |
| ENSG00000100311 | 4.91e-07 | 2.45e-06 |
| ENSG00000100312 | 4.22e-02 | 7.15e-02 |
| ENSG00000100314 | 4.04e-01 | 4.87e-01 |
| ENSG00000100316 | 1.95e-02 | 3.66e-02 |
| ENSG00000100319 | 3.24e-01 | 4.06e-01 |
| ENSG00000100320 | 9.32e-01 | 9.49e-01 |
| ENSG00000100321 | 4.47e-16 | 1.16e-14 |
| ENSG00000100324 | 6.13e-01 | 6.85e-01 |
| ENSG00000100325 | 1.58e-05 | 6.02e-05 |
| ENSG00000100330 | 1.06e-09 | 8.36e-09 |
| ENSG00000100335 | 1.14e-01 | 1.68e-01 |
| ENSG00000100336 | 2.60e-02 | 4.71e-02 |
| ENSG00000100341 | 6.64e-01 | 7.30e-01 |
| ENSG00000100342 | 1.42e-03 | 3.61e-03 |
| ENSG00000100344 | 2.04e-12 | 2.61e-11 |
| ENSG00000100345 | 6.94e-02 | 1.10e-01 |
| ENSG00000100346 | 1.77e-04 | 5.48e-04 |
| ENSG00000100347 | 8.41e-02 | 1.30e-01 |
| ENSG00000100348 | 4.22e-03 | 9.53e-03 |
| ENSG00000100350 | 4.92e-13 | 7.04e-12 |
| ENSG00000100351 | 4.89e-08 | 2.92e-07 |
| ENSG00000100353 | 3.08e-07 | 1.59e-06 |
| ENSG00000100354 | 3.14e-02 | 5.54e-02 |
| ENSG00000100359 | 2.09e-03 | 5.12e-03 |
| ENSG00000100360 | 4.05e-03 | 9.20e-03 |
| ENSG00000100362 | 5.38e-04 | 1.51e-03 |
| ENSG00000100364 | 2.30e-09 | 1.71e-08 |
| ENSG00000100365 | 1.18e-05 | 4.61e-05 |
| ENSG00000100368 | 1.66e-07 | 9.02e-07 |
| ENSG00000100372 | 3.17e-09 | 2.30e-08 |
| ENSG00000100373 | 9.69e-03 | 1.98e-02 |
| ENSG00000100376 | 9.47e-08 | 5.36e-07 |
| ENSG00000100379 | 4.90e-01 | 5.71e-01 |
| ENSG00000100380 | 2.23e-01 | 2.97e-01 |
| ENSG00000100385 | 8.72e-05 | 2.88e-04 |
| ENSG00000100387 | 1.80e-01 | 2.48e-01 |
| ENSG00000100393 | 6.09e-02 | 9.82e-02 |
| ENSG00000100395 | 1.11e-02 | 2.24e-02 |
| ENSG00000100399 | 2.77e-04 | 8.24e-04 |
| ENSG00000100401 | 2.72e-16 | 7.40e-15 |
| ENSG00000100403 | 1.33e-02 | 2.62e-02 |
| ENSG00000100410 | 1.66e-05 | 6.30e-05 |
| ENSG00000100412 | 1.81e-13 | 2.80e-12 |
| ENSG00000100413 | 1.35e-07 | 7.42e-07 |
| ENSG00000100416 | 7.98e-13 | 1.10e-11 |
| ENSG00000100417 | 1.22e-06 | 5.68e-06 |
| ENSG00000100418 | 6.36e-17 | 1.96e-15 |
| ENSG00000100422 | 3.46e-10 | 2.96e-09 |
| ENSG00000100425 | 3.19e-01 | 4.00e-01 |
| ENSG00000100426 | 4.70e-06 | 1.97e-05 |
| ENSG00000100427 | 4.91e-02 | 8.16e-02 |
| ENSG00000100429 | 7.76e-09 | 5.27e-08 |
| ENSG00000100433 | 9.75e-13 | 1.32e-11 |
| ENSG00000100439 | 9.13e-06 | 3.64e-05 |
| ENSG00000100441 | 2.75e-02 | 4.94e-02 |
| ENSG00000100442 | 2.49e-01 | 3.25e-01 |
| ENSG00000100445 | 3.01e-01 | 3.81e-01 |
| ENSG00000100448 | 2.42e-17 | 8.24e-16 |
| ENSG00000100450 | 3.41e-01 | 4.23e-01 |
| ENSG00000100453 | 5.28e-11 | 5.19e-10 |
| ENSG00000100461 | 2.51e-02 | 4.57e-02 |
| ENSG00000100462 | 1.96e-10 | 1.75e-09 |
| ENSG00000100473 | 6.49e-02 | 1.04e-01 |
| ENSG00000100478 | 2.01e-05 | 7.53e-05 |
| ENSG00000100479 | 1.82e-12 | 2.35e-11 |
| ENSG00000100483 | 2.46e-01 | 3.22e-01 |
| ENSG00000100485 | 2.44e-12 | 3.07e-11 |
| ENSG00000100490 | 2.77e-20 | 1.95e-18 |
| ENSG00000100503 | 1.34e-02 | 2.63e-02 |
| ENSG00000100504 | 3.85e-02 | 6.61e-02 |
| ENSG00000100505 | 1.26e-14 | 2.43e-13 |
| ENSG00000100519 | 3.08e-01 | 3.89e-01 |
| ENSG00000100522 | 2.11e-08 | 1.33e-07 |
| ENSG00000100523 | 2.79e-02 | 4.99e-02 |
| ENSG00000100526 | 4.64e-13 | 6.68e-12 |
| ENSG00000100528 | 9.15e-01 | 9.37e-01 |
| ENSG00000100532 | 1.06e-12 | 1.42e-11 |
| ENSG00000100554 | 2.44e-12 | 3.07e-11 |
| ENSG00000100557 | 1.09e-11 | 1.21e-10 |
| ENSG00000100558 | 8.93e-08 | 5.08e-07 |
| ENSG00000100564 | 5.61e-01 | 6.38e-01 |
| ENSG00000100565 | 4.69e-05 | 1.63e-04 |
| ENSG00000100567 | 3.08e-06 | 1.33e-05 |
| ENSG00000100568 | 1.40e-11 | 1.51e-10 |
| ENSG00000100575 | 8.22e-07 | 3.95e-06 |
| ENSG00000100577 | 4.61e-08 | 2.76e-07 |
| ENSG00000100578 | 8.81e-03 | 1.83e-02 |
| ENSG00000100580 | 6.55e-01 | 7.23e-01 |
| ENSG00000100583 | 2.01e-07 | 1.07e-06 |
| ENSG00000100591 | 3.56e-08 | 2.16e-07 |
| ENSG00000100592 | 2.21e-05 | 8.19e-05 |
| ENSG00000100593 | 3.80e-07 | 1.93e-06 |
| ENSG00000100596 | 1.35e-02 | 2.65e-02 |
| ENSG00000100599 | 3.67e-04 | 1.07e-03 |
| ENSG00000100600 | 4.64e-06 | 1.94e-05 |
| ENSG00000100601 | 2.89e-01 | 3.69e-01 |
| ENSG00000100603 | 4.15e-01 | 4.98e-01 |
| ENSG00000100604 | 1.91e-18 | 8.37e-17 |
| ENSG00000100605 | 8.42e-05 | 2.78e-04 |
| ENSG00000100612 | 1.40e-03 | 3.58e-03 |
| ENSG00000100614 | 1.16e-14 | 2.24e-13 |
| ENSG00000100625 | 7.52e-15 | 1.51e-13 |
| ENSG00000100626 | 3.95e-16 | 1.03e-14 |
| ENSG00000100628 | 4.97e-18 | 1.96e-16 |
| ENSG00000100629 | 6.23e-02 | 1.00e-01 |
| ENSG00000100632 | 2.60e-09 | 1.91e-08 |
| ENSG00000100644 | 8.77e-01 | 9.08e-01 |
| ENSG00000100647 | 6.65e-07 | 3.25e-06 |
| ENSG00000100650 | 2.43e-11 | 2.53e-10 |
| ENSG00000100652 | 1.44e-05 | 5.54e-05 |
| ENSG00000100664 | 8.19e-04 | 2.21e-03 |
| ENSG00000100665 | 7.51e-19 | 3.67e-17 |
| ENSG00000100678 | 8.54e-16 | 2.06e-14 |
| ENSG00000100697 | 5.40e-03 | 1.19e-02 |
| ENSG00000100711 | 1.32e-03 | 3.39e-03 |
| ENSG00000100714 | 1.20e-12 | 1.60e-11 |
| ENSG00000100721 | 3.34e-08 | 2.05e-07 |
| ENSG00000100722 | 2.01e-03 | 4.93e-03 |
| ENSG00000100726 | 7.75e-11 | 7.41e-10 |
| ENSG00000100731 | 5.48e-01 | 6.26e-01 |
| ENSG00000100739 | 5.15e-13 | 7.34e-12 |
| ENSG00000100744 | 3.52e-10 | 3.01e-09 |
| ENSG00000100749 | 2.03e-07 | 1.09e-06 |
| ENSG00000100764 | 4.10e-01 | 4.93e-01 |
| ENSG00000100767 | 2.67e-03 | 6.37e-03 |
| ENSG00000100784 | 1.09e-11 | 1.20e-10 |
| ENSG00000100796 | 2.30e-03 | 5.57e-03 |
| ENSG00000100802 | 8.60e-01 | 8.95e-01 |
| ENSG00000100804 | 3.26e-07 | 1.68e-06 |
| ENSG00000100811 | 1.40e-01 | 2.00e-01 |
| ENSG00000100813 | 9.55e-02 | 1.45e-01 |
| ENSG00000100814 | 4.24e-18 | 1.70e-16 |
| ENSG00000100815 | 5.40e-03 | 1.19e-02 |
| ENSG00000100823 | 1.05e-10 | 9.76e-10 |
| ENSG00000100836 | 6.50e-03 | 1.39e-02 |
| ENSG00000100842 | 5.54e-08 | 3.27e-07 |
| ENSG00000100852 | 2.23e-01 | 2.96e-01 |
| ENSG00000100865 | 2.84e-02 | 5.07e-02 |
| ENSG00000100867 | 4.77e-18 | 1.89e-16 |
| ENSG00000100883 | 3.27e-01 | 4.09e-01 |
| ENSG00000100884 | 3.71e-01 | 4.54e-01 |
| ENSG00000100888 | 8.56e-01 | 8.92e-01 |
| ENSG00000100889 | 1.28e-06 | 5.93e-06 |
| ENSG00000100890 | 6.74e-01 | 7.39e-01 |
| ENSG00000100897 | 3.06e-11 | 3.14e-10 |
| ENSG00000100902 | 6.58e-01 | 7.26e-01 |
| ENSG00000100906 | 1.52e-06 | 6.95e-06 |
| ENSG00000100908 | 2.39e-01 | 3.14e-01 |
| ENSG00000100911 | 1.23e-01 | 1.79e-01 |
| ENSG00000100916 | 1.03e-01 | 1.54e-01 |
| ENSG00000100918 | 1.33e-02 | 2.61e-02 |
| ENSG00000100926 | 1.48e-01 | 2.10e-01 |
| ENSG00000100934 | 8.13e-07 | 3.91e-06 |
| ENSG00000100938 | 5.30e-02 | 8.72e-02 |
| ENSG00000100941 | 1.73e-09 | 1.31e-08 |
| ENSG00000100949 | 4.11e-01 | 4.94e-01 |
| ENSG00000100968 | 3.15e-01 | 3.96e-01 |
| ENSG00000100979 | 4.32e-01 | 5.15e-01 |
| ENSG00000100982 | 3.90e-04 | 1.13e-03 |
| ENSG00000100983 | 2.15e-04 | 6.56e-04 |
| ENSG00000100985 | 1.52e-06 | 6.94e-06 |
| ENSG00000100987 | 8.98e-05 | 2.95e-04 |
| ENSG00000100991 | 2.48e-10 | 2.18e-09 |
| ENSG00000100994 | 4.42e-01 | 5.25e-01 |
| ENSG00000100997 | 8.21e-05 | 2.72e-04 |
| ENSG00000101000 | 1.58e-08 | 1.02e-07 |
| ENSG00000101003 | 1.59e-19 | 9.18e-18 |
| ENSG00000101004 | 4.81e-01 | 5.63e-01 |
| ENSG00000101017 | 2.04e-01 | 2.75e-01 |
| ENSG00000101019 | 1.56e-08 | 1.01e-07 |
| ENSG00000101040 | 1.67e-08 | 1.07e-07 |
| ENSG00000101049 | 1.52e-12 | 1.99e-11 |
| ENSG00000101052 | 5.12e-07 | 2.54e-06 |
| ENSG00000101057 | 2.89e-18 | 1.21e-16 |
| ENSG00000101074 | 2.49e-07 | 1.31e-06 |
| ENSG00000101076 | 4.25e-01 | 5.08e-01 |
| ENSG00000101079 | 2.09e-01 | 2.81e-01 |
| ENSG00000101082 | 5.48e-09 | 3.83e-08 |
| ENSG00000101084 | 2.08e-06 | 9.26e-06 |
| ENSG00000101096 | 1.11e-02 | 2.24e-02 |
| ENSG00000101098 | 2.95e-19 | 1.58e-17 |
| ENSG00000101104 | 8.57e-17 | 2.58e-15 |
| ENSG00000101109 | 1.46e-05 | 5.62e-05 |
| ENSG00000101115 | 1.25e-28 | 1.59e-25 |
| ENSG00000101126 | 1.44e-14 | 2.76e-13 |
| ENSG00000101132 | 2.51e-07 | 1.32e-06 |
| ENSG00000101134 | 4.99e-02 | 8.28e-02 |
| ENSG00000101138 | 3.47e-21 | 3.25e-19 |
| ENSG00000101144 | 9.65e-12 | 1.08e-10 |
| ENSG00000101146 | 6.16e-20 | 3.91e-18 |
| ENSG00000101150 | 3.80e-19 | 2.00e-17 |
| ENSG00000101152 | 2.37e-02 | 4.34e-02 |
| ENSG00000101158 | 1.63e-21 | 1.72e-19 |
| ENSG00000101160 | 1.51e-03 | 3.82e-03 |
| ENSG00000101161 | 6.68e-09 | 4.59e-08 |
| ENSG00000101162 | 5.30e-02 | 8.72e-02 |
| ENSG00000101166 | 6.20e-04 | 1.72e-03 |
| ENSG00000101180 | 5.33e-01 | 6.12e-01 |
| ENSG00000101181 | 1.01e-12 | 1.37e-11 |
| ENSG00000101182 | 2.18e-14 | 4.03e-13 |
| ENSG00000101187 | 4.57e-28 | 5.17e-25 |
| ENSG00000101188 | 3.34e-01 | 4.16e-01 |
| ENSG00000101189 | 7.32e-25 | 2.24e-22 |
| ENSG00000101190 | 2.24e-17 | 7.72e-16 |
| ENSG00000101191 | 6.52e-05 | 2.20e-04 |
| ENSG00000101193 | 6.85e-14 | 1.14e-12 |
| ENSG00000101194 | 3.30e-16 | 8.80e-15 |
| ENSG00000101197 | 8.23e-07 | 3.96e-06 |
| ENSG00000101198 | 2.92e-02 | 5.20e-02 |
| ENSG00000101199 | 2.10e-16 | 5.86e-15 |
| ENSG00000101203 | 7.16e-01 | 7.76e-01 |
| ENSG00000101204 | 9.83e-06 | 3.89e-05 |
| ENSG00000101210 | 2.07e-10 | 1.84e-09 |
| ENSG00000101213 | 1.24e-03 | 3.21e-03 |
| ENSG00000101216 | 1.70e-08 | 1.10e-07 |
| ENSG00000101220 | 6.68e-08 | 3.88e-07 |
| ENSG00000101222 | 4.45e-05 | 1.56e-04 |
| ENSG00000101224 | 4.50e-25 | 1.48e-22 |
| ENSG00000101230 | 2.90e-05 | 1.05e-04 |
| ENSG00000101236 | 1.09e-12 | 1.46e-11 |
| ENSG00000101246 | 6.97e-05 | 2.34e-04 |
| ENSG00000101247 | 7.14e-03 | 1.52e-02 |
| ENSG00000101251 | 3.32e-01 | 4.13e-01 |
| ENSG00000101255 | 2.32e-29 | 3.61e-26 |
| ENSG00000101265 | 1.18e-08 | 7.76e-08 |
| ENSG00000101266 | 8.34e-05 | 2.76e-04 |
| ENSG00000101276 | 1.56e-01 | 2.19e-01 |
| ENSG00000101278 | 1.91e-09 | 1.44e-08 |
| ENSG00000101280 | 2.44e-01 | 3.20e-01 |
| ENSG00000101282 | 4.98e-01 | 5.79e-01 |
| ENSG00000101290 | 8.17e-01 | 8.60e-01 |
| ENSG00000101292 | 7.56e-01 | 8.10e-01 |
| ENSG00000101294 | 2.50e-20 | 1.79e-18 |
| ENSG00000101298 | 1.08e-09 | 8.49e-09 |
| ENSG00000101306 | 6.77e-07 | 3.30e-06 |
| ENSG00000101307 | 3.45e-01 | 4.27e-01 |
| ENSG00000101310 | 1.98e-10 | 1.77e-09 |
| ENSG00000101311 | 1.79e-12 | 2.31e-11 |
| ENSG00000101323 | 9.56e-01 | 9.68e-01 |
| ENSG00000101327 | 1.09e-01 | 1.62e-01 |
| ENSG00000101331 | 2.92e-08 | 1.80e-07 |
| ENSG00000101333 | 3.14e-05 | 1.13e-04 |
| ENSG00000101335 | 1.41e-06 | 6.48e-06 |
| ENSG00000101336 | 6.92e-05 | 2.33e-04 |
| ENSG00000101337 | 4.93e-10 | 4.10e-09 |
| ENSG00000101342 | 5.33e-01 | 6.12e-01 |
| ENSG00000101343 | 1.94e-04 | 5.98e-04 |
| ENSG00000101346 | 1.13e-06 | 5.30e-06 |
| ENSG00000101347 | 7.01e-02 | 1.11e-01 |
| ENSG00000101349 | 2.28e-05 | 8.44e-05 |
| ENSG00000101350 | 6.05e-01 | 6.77e-01 |
| ENSG00000101353 | 2.56e-04 | 7.67e-04 |
| ENSG00000101361 | 5.22e-22 | 6.41e-20 |
| ENSG00000101363 | 1.50e-07 | 8.22e-07 |
| ENSG00000101365 | 7.80e-01 | 8.29e-01 |
| ENSG00000101367 | 5.82e-08 | 3.42e-07 |
| ENSG00000101384 | 2.48e-01 | 3.25e-01 |
| ENSG00000101391 | 2.96e-18 | 1.24e-16 |
| ENSG00000101400 | 2.22e-02 | 4.09e-02 |
| ENSG00000101405 | 1.18e-01 | 1.73e-01 |
| ENSG00000101407 | 2.24e-19 | 1.24e-17 |
| ENSG00000101412 | 1.73e-20 | 1.29e-18 |
| ENSG00000101413 | 4.19e-12 | 5.02e-11 |
| ENSG00000101417 | 4.29e-01 | 5.12e-01 |
| ENSG00000101421 | 1.16e-01 | 1.71e-01 |
| ENSG00000101425 | 4.95e-03 | 1.10e-02 |
| ENSG00000101438 | 9.70e-07 | 4.60e-06 |
| ENSG00000101439 | 5.01e-01 | 5.81e-01 |
| ENSG00000101440 | 4.71e-05 | 1.64e-04 |
| ENSG00000101441 | 4.35e-23 | 7.31e-21 |
| ENSG00000101442 | 4.37e-15 | 9.17e-14 |
| ENSG00000101443 | 6.76e-05 | 2.28e-04 |
| ENSG00000101444 | 7.41e-18 | 2.81e-16 |
| ENSG00000101445 | 1.94e-14 | 3.62e-13 |
| ENSG00000101447 | 1.13e-10 | 1.05e-09 |
| ENSG00000101448 | 9.73e-03 | 1.99e-02 |
| ENSG00000101452 | 3.16e-09 | 2.30e-08 |
| ENSG00000101457 | 4.05e-15 | 8.57e-14 |
| ENSG00000101460 | 5.20e-01 | 5.99e-01 |
| ENSG00000101463 | 1.68e-03 | 4.21e-03 |
| ENSG00000101464 | 1.36e-10 | 1.25e-09 |
| ENSG00000101470 | 1.47e-01 | 2.09e-01 |
| ENSG00000101473 | 1.87e-01 | 2.55e-01 |
| ENSG00000101474 | 3.54e-08 | 2.15e-07 |
| ENSG00000101489 | 1.11e-16 | 3.26e-15 |
| ENSG00000101493 | 2.00e-01 | 2.71e-01 |
| ENSG00000101542 | 4.42e-02 | 7.45e-02 |
| ENSG00000101544 | 8.96e-02 | 1.37e-01 |
| ENSG00000101546 | 4.73e-03 | 1.06e-02 |
| ENSG00000101557 | 1.31e-04 | 4.18e-04 |
| ENSG00000101558 | 5.10e-16 | 1.31e-14 |
| ENSG00000101574 | 4.07e-02 | 6.93e-02 |
| ENSG00000101577 | 7.99e-06 | 3.22e-05 |
| ENSG00000101596 | 6.24e-10 | 5.09e-09 |
| ENSG00000101605 | 9.74e-21 | 8.01e-19 |
| ENSG00000101608 | 3.20e-06 | 1.38e-05 |
| ENSG00000101624 | 9.98e-04 | 2.64e-03 |
| ENSG00000101638 | 4.37e-04 | 1.25e-03 |
| ENSG00000101639 | 9.95e-02 | 1.50e-01 |
| ENSG00000101654 | 1.36e-03 | 3.49e-03 |
| ENSG00000101665 | 3.12e-13 | 4.63e-12 |
| ENSG00000101670 | 8.14e-18 | 3.07e-16 |
| ENSG00000101680 | 1.55e-17 | 5.48e-16 |
| ENSG00000101695 | 1.47e-12 | 1.93e-11 |
| ENSG00000101745 | 4.15e-09 | 2.95e-08 |
| ENSG00000101746 | 6.86e-10 | 5.56e-09 |
| ENSG00000101751 | 5.95e-01 | 6.69e-01 |
| ENSG00000101752 | 2.80e-01 | 3.59e-01 |
| ENSG00000101773 | 6.91e-01 | 7.55e-01 |
| ENSG00000101782 | 1.51e-19 | 8.77e-18 |
| ENSG00000101811 | 1.67e-07 | 9.07e-07 |
| ENSG00000101812 | 1.81e-02 | 3.42e-02 |
| ENSG00000101825 | 7.26e-06 | 2.94e-05 |
| ENSG00000101842 | 8.22e-03 | 1.72e-02 |
| ENSG00000101843 | 2.32e-07 | 1.22e-06 |
| ENSG00000101844 | 4.26e-13 | 6.18e-12 |
| ENSG00000101846 | 6.19e-05 | 2.10e-04 |
| ENSG00000101849 | 8.93e-01 | 9.20e-01 |
| ENSG00000101850 | 1.24e-07 | 6.91e-07 |
| ENSG00000101856 | 9.81e-01 | 9.86e-01 |
| ENSG00000101868 | 2.49e-12 | 3.13e-11 |
| ENSG00000101871 | 1.49e-01 | 2.11e-01 |
| ENSG00000101882 | 1.88e-05 | 7.06e-05 |
| ENSG00000101883 | 9.67e-01 | 9.75e-01 |
| ENSG00000101888 | 1.55e-03 | 3.91e-03 |
| ENSG00000101892 | 4.51e-01 | 5.34e-01 |
| ENSG00000101898 | 8.16e-15 | 1.62e-13 |
| ENSG00000101901 | 9.78e-03 | 2.00e-02 |
| ENSG00000101911 | 1.25e-11 | 1.36e-10 |
| ENSG00000101916 | 5.80e-05 | 1.98e-04 |
| ENSG00000101928 | 3.57e-07 | 1.83e-06 |
| ENSG00000101935 | 6.62e-12 | 7.65e-11 |
| ENSG00000101938 | 1.37e-18 | 6.23e-17 |
| ENSG00000101940 | 3.32e-01 | 4.13e-01 |
| ENSG00000101945 | 1.34e-17 | 4.80e-16 |
| ENSG00000101951 | 4.11e-03 | 9.33e-03 |
| ENSG00000101955 | 5.32e-18 | 2.09e-16 |
| ENSG00000101958 | 2.26e-05 | 8.37e-05 |
| ENSG00000101966 | 6.67e-01 | 7.33e-01 |
| ENSG00000101972 | 5.30e-02 | 8.71e-02 |
| ENSG00000101974 | 2.24e-02 | 4.13e-02 |
| ENSG00000101977 | 8.25e-01 | 8.67e-01 |
| ENSG00000101981 | 5.41e-01 | 6.19e-01 |
| ENSG00000101986 | 4.47e-01 | 5.30e-01 |
| ENSG00000101997 | 1.19e-04 | 3.83e-04 |
| ENSG00000102001 | 6.24e-01 | 6.95e-01 |
| ENSG00000102003 | 6.69e-20 | 4.22e-18 |
| ENSG00000102007 | 3.89e-11 | 3.92e-10 |
| ENSG00000102010 | 1.42e-22 | 2.01e-20 |
| ENSG00000102024 | 4.17e-11 | 4.18e-10 |
| ENSG00000102030 | 4.57e-15 | 9.55e-14 |
| ENSG00000102032 | 4.06e-03 | 9.22e-03 |
| ENSG00000102034 | 1.68e-05 | 6.37e-05 |
| ENSG00000102038 | 1.39e-03 | 3.56e-03 |
| ENSG00000102043 | 4.54e-01 | 5.37e-01 |
| ENSG00000102048 | 3.97e-15 | 8.44e-14 |
| ENSG00000102053 | 1.16e-01 | 1.71e-01 |
| ENSG00000102054 | 1.85e-09 | 1.39e-08 |
| ENSG00000102057 | 6.57e-02 | 1.05e-01 |
| ENSG00000102078 | 5.25e-13 | 7.47e-12 |
| ENSG00000102081 | 7.42e-03 | 1.57e-02 |
| ENSG00000102096 | 8.02e-02 | 1.24e-01 |
| ENSG00000102098 | 1.01e-02 | 2.06e-02 |
| ENSG00000102100 | 9.99e-07 | 4.72e-06 |
| ENSG00000102103 | 9.18e-05 | 3.01e-04 |
| ENSG00000102104 | 9.34e-01 | 9.51e-01 |
| ENSG00000102109 | 2.12e-10 | 1.88e-09 |
| ENSG00000102119 | 1.39e-13 | 2.19e-12 |
| ENSG00000102125 | 1.07e-11 | 1.19e-10 |
| ENSG00000102128 | 6.95e-03 | 1.48e-02 |
| ENSG00000102144 | 1.39e-13 | 2.19e-12 |
| ENSG00000102145 | 2.02e-02 | 3.78e-02 |
| ENSG00000102158 | 3.17e-03 | 7.41e-03 |
| ENSG00000102172 | 4.09e-10 | 3.46e-09 |
| ENSG00000102174 | 4.80e-01 | 5.62e-01 |
| ENSG00000102178 | 1.91e-07 | 1.03e-06 |
| ENSG00000102181 | 2.78e-02 | 4.98e-02 |
| ENSG00000102189 | 8.87e-01 | 9.16e-01 |
| ENSG00000102195 | 1.53e-01 | 2.16e-01 |
| ENSG00000102218 | 3.04e-01 | 3.85e-01 |
| ENSG00000102221 | 1.38e-18 | 6.25e-17 |
| ENSG00000102225 | 6.23e-07 | 3.05e-06 |
| ENSG00000102226 | 2.41e-05 | 8.87e-05 |
| ENSG00000102230 | 1.15e-04 | 3.70e-04 |
| ENSG00000102239 | 8.47e-01 | 8.85e-01 |
| ENSG00000102241 | 7.49e-03 | 1.58e-02 |
| ENSG00000102243 | 1.11e-11 | 1.23e-10 |
| ENSG00000102245 | 4.40e-10 | 3.69e-09 |
| ENSG00000102265 | 2.93e-21 | 2.82e-19 |
| ENSG00000102271 | 5.95e-05 | 2.03e-04 |
| ENSG00000102287 | 2.62e-13 | 3.94e-12 |
| ENSG00000102290 | 1.03e-10 | 9.62e-10 |
| ENSG00000102302 | 3.90e-02 | 6.69e-02 |
| ENSG00000102309 | 6.69e-07 | 3.26e-06 |
| ENSG00000102312 | 3.21e-10 | 2.76e-09 |
| ENSG00000102313 | 2.84e-05 | 1.03e-04 |
| ENSG00000102316 | 2.62e-08 | 1.63e-07 |
| ENSG00000102317 | 1.62e-07 | 8.82e-07 |
| ENSG00000102349 | 9.10e-10 | 7.24e-09 |
| ENSG00000102359 | 9.31e-18 | 3.46e-16 |
| ENSG00000102362 | 4.75e-05 | 1.65e-04 |
| ENSG00000102383 | 1.14e-10 | 1.06e-09 |
| ENSG00000102384 | 1.87e-15 | 4.21e-14 |
| ENSG00000102385 | 3.13e-01 | 3.94e-01 |
| ENSG00000102387 | 4.24e-03 | 9.58e-03 |
| ENSG00000102390 | 9.96e-04 | 2.63e-03 |
| ENSG00000102393 | 6.46e-14 | 1.08e-12 |
| ENSG00000102401 | 2.84e-05 | 1.03e-04 |
| ENSG00000102409 | 1.21e-15 | 2.85e-14 |
| ENSG00000102445 | 4.89e-06 | 2.04e-05 |
| ENSG00000102452 | 6.48e-01 | 7.16e-01 |
| ENSG00000102466 | 3.49e-07 | 1.79e-06 |
| ENSG00000102468 | 6.34e-04 | 1.75e-03 |
| ENSG00000102471 | 9.91e-01 | 9.93e-01 |
| ENSG00000102524 | 8.66e-02 | 1.33e-01 |
| ENSG00000102531 | 1.32e-01 | 1.91e-01 |
| ENSG00000102539 | 4.63e-01 | 5.46e-01 |
| ENSG00000102543 | 1.29e-02 | 2.55e-02 |
| ENSG00000102547 | 2.83e-01 | 3.62e-01 |
| ENSG00000102554 | 4.27e-02 | 7.23e-02 |
| ENSG00000102572 | 4.89e-01 | 5.71e-01 |
| ENSG00000102575 | 9.14e-03 | 1.89e-02 |
| ENSG00000102580 | 4.71e-01 | 5.54e-01 |
| ENSG00000102595 | 6.38e-17 | 1.97e-15 |
| ENSG00000102606 | 1.86e-05 | 7.00e-05 |
| ENSG00000102678 | 2.36e-11 | 2.46e-10 |
| ENSG00000102683 | 5.42e-22 | 6.64e-20 |
| ENSG00000102699 | 4.47e-01 | 5.30e-01 |
| ENSG00000102710 | 1.79e-09 | 1.35e-08 |
| ENSG00000102738 | 8.34e-04 | 2.25e-03 |
| ENSG00000102743 | 2.01e-16 | 5.62e-15 |
| ENSG00000102753 | 3.39e-05 | 1.21e-04 |
| ENSG00000102755 | 5.13e-05 | 1.77e-04 |
| ENSG00000102760 | 5.08e-06 | 2.12e-05 |
| ENSG00000102763 | 4.44e-03 | 9.98e-03 |
| ENSG00000102780 | 6.47e-04 | 1.79e-03 |
| ENSG00000102781 | 9.05e-03 | 1.87e-02 |
| ENSG00000102786 | 1.34e-04 | 4.25e-04 |
| ENSG00000102794 | 2.18e-05 | 8.09e-05 |
| ENSG00000102796 | 4.06e-01 | 4.89e-01 |
| ENSG00000102802 | 6.82e-02 | 1.08e-01 |
| ENSG00000102804 | 3.92e-03 | 8.94e-03 |
| ENSG00000102805 | 3.64e-02 | 6.30e-02 |
| ENSG00000102837 | 7.01e-02 | 1.11e-01 |
| ENSG00000102854 | 5.06e-05 | 1.75e-04 |
| ENSG00000102858 | 1.95e-06 | 8.73e-06 |
| ENSG00000102870 | 3.42e-03 | 7.93e-03 |
| ENSG00000102871 | 5.24e-02 | 8.63e-02 |
| ENSG00000102878 | 1.84e-13 | 2.84e-12 |
| ENSG00000102879 | 3.60e-02 | 6.24e-02 |
| ENSG00000102882 | 1.04e-09 | 8.23e-09 |
| ENSG00000102886 | 7.08e-17 | 2.16e-15 |
| ENSG00000102890 | 4.80e-01 | 5.62e-01 |
| ENSG00000102891 | 1.13e-06 | 5.28e-06 |
| ENSG00000102893 | 1.11e-04 | 3.57e-04 |
| ENSG00000102897 | 6.53e-08 | 3.80e-07 |
| ENSG00000102898 | 2.43e-18 | 1.04e-16 |
| ENSG00000102900 | 1.36e-15 | 3.15e-14 |
| ENSG00000102901 | 1.80e-04 | 5.58e-04 |
| ENSG00000102904 | 1.12e-01 | 1.66e-01 |
| ENSG00000102908 | 3.74e-05 | 1.33e-04 |
| ENSG00000102910 | 2.02e-04 | 6.18e-04 |
| ENSG00000102921 | 2.17e-02 | 4.02e-02 |
| ENSG00000102924 | 9.03e-01 | 9.28e-01 |
| ENSG00000102931 | 3.19e-01 | 4.00e-01 |
| ENSG00000102934 | 6.57e-01 | 7.25e-01 |
| ENSG00000102935 | 2.81e-04 | 8.36e-04 |
| ENSG00000102962 | 7.46e-02 | 1.17e-01 |
| ENSG00000102967 | 3.44e-15 | 7.40e-14 |
| ENSG00000102970 | 4.02e-01 | 4.85e-01 |
| ENSG00000102974 | 5.63e-02 | 9.18e-02 |
| ENSG00000102977 | 6.05e-14 | 1.02e-12 |
| ENSG00000102978 | 5.06e-09 | 3.55e-08 |
| ENSG00000102981 | 9.30e-06 | 3.70e-05 |
| ENSG00000102984 | 2.48e-11 | 2.58e-10 |
| ENSG00000102996 | 1.37e-06 | 6.28e-06 |
| ENSG00000103005 | 3.77e-01 | 4.60e-01 |
| ENSG00000103018 | 2.11e-10 | 1.87e-09 |
| ENSG00000103021 | 1.92e-20 | 1.42e-18 |
| ENSG00000103023 | 2.63e-02 | 4.75e-02 |
| ENSG00000103024 | 3.64e-01 | 4.46e-01 |
| ENSG00000103034 | 2.41e-08 | 1.51e-07 |
| ENSG00000103035 | 1.84e-09 | 1.39e-08 |
| ENSG00000103037 | 3.22e-20 | 2.22e-18 |
| ENSG00000103042 | 1.48e-15 | 3.41e-14 |
| ENSG00000103043 | 6.08e-11 | 5.91e-10 |
| ENSG00000103044 | 3.28e-01 | 4.10e-01 |
| ENSG00000103047 | 1.56e-11 | 1.67e-10 |
| ENSG00000103051 | 8.88e-03 | 1.84e-02 |
| ENSG00000103056 | 8.81e-14 | 1.44e-12 |
| ENSG00000103061 | 1.65e-07 | 8.93e-07 |
| ENSG00000103064 | 4.69e-17 | 1.50e-15 |
| ENSG00000103066 | 2.25e-03 | 5.45e-03 |
| ENSG00000103067 | 3.85e-01 | 4.68e-01 |
| ENSG00000103089 | 4.82e-03 | 1.07e-02 |
| ENSG00000103091 | 2.41e-13 | 3.65e-12 |
| ENSG00000103111 | 7.49e-01 | 8.04e-01 |
| ENSG00000103121 | 1.36e-06 | 6.26e-06 |
| ENSG00000103126 | 2.66e-05 | 9.72e-05 |
| ENSG00000103145 | 9.10e-02 | 1.39e-01 |
| ENSG00000103148 | 9.60e-20 | 5.82e-18 |
| ENSG00000103150 | 7.79e-02 | 1.21e-01 |
| ENSG00000103152 | 5.41e-08 | 3.20e-07 |
| ENSG00000103154 | 7.80e-11 | 7.45e-10 |
| ENSG00000103160 | 9.86e-07 | 4.66e-06 |
| ENSG00000103168 | 4.90e-10 | 4.08e-09 |
| ENSG00000103174 | 4.20e-01 | 5.03e-01 |
| ENSG00000103175 | 1.35e-05 | 5.22e-05 |
| ENSG00000103184 | 3.11e-18 | 1.29e-16 |
| ENSG00000103187 | 1.07e-01 | 1.59e-01 |
| ENSG00000103194 | 5.28e-11 | 5.19e-10 |
| ENSG00000103196 | 4.11e-03 | 9.32e-03 |
| ENSG00000103197 | 4.83e-03 | 1.08e-02 |
| ENSG00000103199 | 1.43e-04 | 4.51e-04 |
| ENSG00000103200 | 8.32e-01 | 8.72e-01 |
| ENSG00000103202 | 6.95e-06 | 2.82e-05 |
| ENSG00000103222 | 5.64e-17 | 1.77e-15 |
| ENSG00000103226 | 2.65e-07 | 1.39e-06 |
| ENSG00000103227 | 3.49e-03 | 8.07e-03 |
| ENSG00000103241 | 6.88e-09 | 4.72e-08 |
| ENSG00000103245 | 7.37e-02 | 1.16e-01 |
| ENSG00000103248 | 9.62e-05 | 3.14e-04 |
| ENSG00000103249 | 6.61e-09 | 4.55e-08 |
| ENSG00000103253 | 3.33e-19 | 1.76e-17 |
| ENSG00000103254 | 5.05e-04 | 1.42e-03 |
| ENSG00000103257 | 1.29e-24 | 3.63e-22 |
| ENSG00000103260 | 6.88e-01 | 7.52e-01 |
| ENSG00000103264 | 6.09e-01 | 6.81e-01 |
| ENSG00000103266 | 7.82e-02 | 1.22e-01 |
| ENSG00000103269 | 1.57e-07 | 8.55e-07 |
| ENSG00000103274 | 5.01e-01 | 5.82e-01 |
| ENSG00000103275 | 2.24e-15 | 4.96e-14 |
| ENSG00000103310 | 3.56e-01 | 4.38e-01 |
| ENSG00000103313 | 4.10e-01 | 4.93e-01 |
| ENSG00000103316 | 7.69e-03 | 1.62e-02 |
| ENSG00000103319 | 2.76e-05 | 1.01e-04 |
| ENSG00000103326 | 4.57e-03 | 1.02e-02 |
| ENSG00000103335 | 1.32e-12 | 1.75e-11 |
| ENSG00000103342 | 3.33e-08 | 2.04e-07 |
| ENSG00000103343 | 3.78e-03 | 8.66e-03 |
| ENSG00000103351 | 7.76e-05 | 2.58e-04 |
| ENSG00000103353 | 5.28e-20 | 3.41e-18 |
| ENSG00000103355 | 5.15e-10 | 4.27e-09 |
| ENSG00000103356 | 4.78e-16 | 1.23e-14 |
| ENSG00000103363 | 4.96e-04 | 1.40e-03 |
| ENSG00000103365 | 1.64e-05 | 6.23e-05 |
| ENSG00000103375 | 3.79e-21 | 3.52e-19 |
| ENSG00000103381 | 4.55e-01 | 5.38e-01 |
| ENSG00000103404 | 3.83e-15 | 8.19e-14 |
| ENSG00000103415 | 1.34e-03 | 3.44e-03 |
| ENSG00000103423 | 2.19e-13 | 3.35e-12 |
| ENSG00000103426 | 5.62e-01 | 6.39e-01 |
| ENSG00000103429 | 1.32e-08 | 8.66e-08 |
| ENSG00000103449 | 3.17e-06 | 1.37e-05 |
| ENSG00000103460 | 5.67e-03 | 1.24e-02 |
| ENSG00000103472 | 7.71e-01 | 8.22e-01 |
| ENSG00000103479 | 5.04e-02 | 8.35e-02 |
| ENSG00000103485 | 4.39e-02 | 7.40e-02 |
| ENSG00000103489 | 4.66e-05 | 1.63e-04 |
| ENSG00000103490 | 2.88e-01 | 3.68e-01 |
| ENSG00000103494 | 1.76e-21 | 1.84e-19 |
| ENSG00000103495 | 6.72e-01 | 7.37e-01 |
| ENSG00000103496 | 1.88e-02 | 3.54e-02 |
| ENSG00000103502 | 4.23e-01 | 5.06e-01 |
| ENSG00000103507 | 2.12e-01 | 2.84e-01 |
| ENSG00000103510 | 5.07e-03 | 1.12e-02 |
| ENSG00000103512 | 1.34e-07 | 7.41e-07 |
| ENSG00000103522 | 8.29e-01 | 8.70e-01 |
| ENSG00000103528 | 1.44e-02 | 2.81e-02 |
| ENSG00000103534 | 8.35e-02 | 1.29e-01 |
| ENSG00000103540 | 8.77e-04 | 2.35e-03 |
| ENSG00000103544 | 1.60e-14 | 3.02e-13 |
| ENSG00000103546 | 1.23e-10 | 1.13e-09 |
| ENSG00000103549 | 2.84e-01 | 3.64e-01 |
| ENSG00000103550 | 1.07e-10 | 9.96e-10 |
| ENSG00000103569 | 8.76e-11 | 8.29e-10 |
| ENSG00000103591 | 2.16e-01 | 2.89e-01 |
| ENSG00000103599 | 7.62e-09 | 5.19e-08 |
| ENSG00000103642 | 7.92e-04 | 2.14e-03 |
| ENSG00000103647 | 2.06e-18 | 8.90e-17 |
| ENSG00000103653 | 7.93e-01 | 8.40e-01 |
| ENSG00000103657 | 7.75e-05 | 2.58e-04 |
| ENSG00000103671 | 1.29e-09 | 9.98e-09 |
| ENSG00000103707 | 2.40e-01 | 3.16e-01 |
| ENSG00000103710 | 1.34e-05 | 5.19e-05 |
| ENSG00000103723 | 1.12e-10 | 1.04e-09 |
| ENSG00000103740 | 2.51e-05 | 9.22e-05 |
| ENSG00000103742 | 9.14e-02 | 1.39e-01 |
| ENSG00000103769 | 8.33e-04 | 2.24e-03 |
| ENSG00000103811 | 1.20e-10 | 1.11e-09 |
| ENSG00000103832 | 9.39e-02 | 1.43e-01 |
| ENSG00000103852 | 5.36e-06 | 2.22e-05 |
| ENSG00000103855 | 1.88e-17 | 6.56e-16 |
| ENSG00000103876 | 3.52e-01 | 4.34e-01 |
| ENSG00000103888 | 1.10e-31 | 3.06e-28 |
| ENSG00000103932 | 1.86e-06 | 8.35e-06 |
| ENSG00000103942 | 7.83e-10 | 6.30e-09 |
| ENSG00000103966 | 1.68e-02 | 3.21e-02 |
| ENSG00000103978 | 6.02e-01 | 6.74e-01 |
| ENSG00000103994 | 2.63e-04 | 7.87e-04 |
| ENSG00000103995 | 1.15e-04 | 3.70e-04 |
| ENSG00000104043 | 9.32e-06 | 3.71e-05 |
| ENSG00000104044 | 2.22e-02 | 4.10e-02 |
| ENSG00000104047 | 5.66e-01 | 6.43e-01 |
| ENSG00000104055 | 9.37e-02 | 1.42e-01 |
| ENSG00000104059 | 6.42e-13 | 8.97e-12 |
| ENSG00000104064 | 1.72e-04 | 5.34e-04 |
| ENSG00000104067 | 1.29e-01 | 1.87e-01 |
| ENSG00000104081 | 2.38e-07 | 1.25e-06 |
| ENSG00000104093 | 1.40e-05 | 5.41e-05 |
| ENSG00000104112 | 1.72e-20 | 1.29e-18 |
| ENSG00000104129 | 4.57e-01 | 5.40e-01 |
| ENSG00000104131 | 2.81e-06 | 1.22e-05 |
| ENSG00000104133 | 1.63e-03 | 4.10e-03 |
| ENSG00000104140 | 6.09e-01 | 6.81e-01 |
| ENSG00000104142 | 1.21e-01 | 1.77e-01 |
| ENSG00000104147 | 2.25e-08 | 1.41e-07 |
| ENSG00000104154 | 3.97e-12 | 4.78e-11 |
| ENSG00000104164 | 1.85e-01 | 2.54e-01 |
| ENSG00000104177 | 8.46e-02 | 1.30e-01 |
| ENSG00000104205 | 1.46e-05 | 5.60e-05 |
| ENSG00000104213 | 1.27e-01 | 1.85e-01 |
| ENSG00000104218 | 6.63e-10 | 5.38e-09 |
| ENSG00000104219 | 5.06e-07 | 2.52e-06 |
| ENSG00000104221 | 7.93e-02 | 1.23e-01 |
| ENSG00000104228 | 5.95e-04 | 1.66e-03 |
| ENSG00000104231 | 6.23e-12 | 7.23e-11 |
| ENSG00000104237 | 1.95e-17 | 6.80e-16 |
| ENSG00000104267 | 1.21e-23 | 2.51e-21 |
| ENSG00000104290 | 2.25e-06 | 9.97e-06 |
| ENSG00000104299 | 3.51e-01 | 4.33e-01 |
| ENSG00000104312 | 6.72e-24 | 1.53e-21 |
| ENSG00000104313 | 1.31e-01 | 1.89e-01 |
| ENSG00000104320 | 3.02e-04 | 8.92e-04 |
| ENSG00000104321 | 1.01e-05 | 4.00e-05 |
| ENSG00000104324 | 5.28e-11 | 5.19e-10 |
| ENSG00000104325 | 2.58e-08 | 1.61e-07 |
| ENSG00000104327 | 2.72e-01 | 3.50e-01 |
| ENSG00000104331 | 1.27e-02 | 2.52e-02 |
| ENSG00000104332 | 2.19e-20 | 1.61e-18 |
| ENSG00000104341 | 9.69e-13 | 1.31e-11 |
| ENSG00000104343 | 2.61e-02 | 4.71e-02 |
| ENSG00000104356 | 1.40e-19 | 8.18e-18 |
| ENSG00000104361 | 1.20e-02 | 2.38e-02 |
| ENSG00000104365 | 6.44e-07 | 3.15e-06 |
| ENSG00000104368 | 2.76e-06 | 1.20e-05 |
| ENSG00000104369 | 2.56e-19 | 1.39e-17 |
| ENSG00000104371 | 4.16e-21 | 3.78e-19 |
| ENSG00000104375 | 1.40e-10 | 1.28e-09 |
| ENSG00000104381 | 1.08e-03 | 2.83e-03 |
| ENSG00000104388 | 9.47e-01 | 9.61e-01 |
| ENSG00000104408 | 9.75e-09 | 6.50e-08 |
| ENSG00000104412 | 1.77e-01 | 2.44e-01 |
| ENSG00000104413 | 3.16e-01 | 3.97e-01 |
| ENSG00000104415 | 1.27e-20 | 1.02e-18 |
| ENSG00000104419 | 5.26e-08 | 3.12e-07 |
| ENSG00000104427 | 3.60e-01 | 4.43e-01 |
| ENSG00000104432 | 1.38e-02 | 2.69e-02 |
| ENSG00000104435 | 2.25e-24 | 5.78e-22 |
| ENSG00000104442 | 7.85e-02 | 1.22e-01 |
| ENSG00000104447 | 8.90e-03 | 1.84e-02 |
| ENSG00000104450 | 8.86e-01 | 9.15e-01 |
| ENSG00000104472 | 2.66e-04 | 7.96e-04 |
| ENSG00000104490 | 6.71e-01 | 7.36e-01 |
| ENSG00000104497 | 3.84e-01 | 4.67e-01 |
| ENSG00000104517 | 5.32e-03 | 1.17e-02 |
| ENSG00000104518 | 2.58e-01 | 3.36e-01 |
| ENSG00000104522 | 1.21e-11 | 1.32e-10 |
| ENSG00000104524 | 7.57e-17 | 2.30e-15 |
| ENSG00000104529 | 1.07e-04 | 3.46e-04 |
| ENSG00000104537 | 2.95e-01 | 3.76e-01 |
| ENSG00000104549 | 1.05e-13 | 1.70e-12 |
| ENSG00000104611 | 3.29e-01 | 4.11e-01 |
| ENSG00000104613 | 8.36e-04 | 2.25e-03 |
| ENSG00000104626 | 4.23e-05 | 1.49e-04 |
| ENSG00000104635 | 2.05e-01 | 2.76e-01 |
| ENSG00000104643 | 7.81e-07 | 3.77e-06 |
| ENSG00000104660 | 1.97e-03 | 4.84e-03 |
| ENSG00000104671 | 4.46e-01 | 5.29e-01 |
| ENSG00000104679 | 7.19e-04 | 1.96e-03 |
| ENSG00000104687 | 5.46e-06 | 2.25e-05 |
| ENSG00000104689 | 2.65e-15 | 5.81e-14 |
| ENSG00000104691 | 7.25e-01 | 7.83e-01 |
| ENSG00000104695 | 8.53e-12 | 9.66e-11 |
| ENSG00000104714 | 5.91e-05 | 2.02e-04 |
| ENSG00000104722 | 2.05e-18 | 8.89e-17 |
| ENSG00000104723 | 5.65e-06 | 2.33e-05 |
| ENSG00000104728 | 2.55e-04 | 7.66e-04 |
| ENSG00000104731 | 6.13e-12 | 7.13e-11 |
| ENSG00000104738 | 3.83e-15 | 8.18e-14 |
| ENSG00000104755 | 1.46e-02 | 2.84e-02 |
| ENSG00000104756 | 4.34e-15 | 9.11e-14 |
| ENSG00000104760 | 8.84e-01 | 9.13e-01 |
| ENSG00000104763 | 6.74e-05 | 2.27e-04 |
| ENSG00000104765 | 3.79e-01 | 4.62e-01 |
| ENSG00000104774 | 2.84e-05 | 1.03e-04 |
| ENSG00000104783 | 5.17e-08 | 3.07e-07 |
| ENSG00000104804 | 2.00e-01 | 2.71e-01 |
| ENSG00000104805 | 6.94e-03 | 1.48e-02 |
| ENSG00000104808 | 5.13e-04 | 1.45e-03 |
| ENSG00000104812 | 2.17e-01 | 2.90e-01 |
| ENSG00000104814 | 3.10e-10 | 2.67e-09 |
| ENSG00000104818 | 7.18e-02 | 1.13e-01 |
| ENSG00000104823 | 1.32e-09 | 1.02e-08 |
| ENSG00000104824 | 5.64e-07 | 2.78e-06 |
| ENSG00000104825 | 5.44e-01 | 6.22e-01 |
| ENSG00000104826 | 1.23e-03 | 3.18e-03 |
| ENSG00000104827 | 2.76e-05 | 1.01e-04 |
| ENSG00000104833 | 1.59e-19 | 9.20e-18 |
| ENSG00000104835 | 2.19e-05 | 8.12e-05 |
| ENSG00000104848 | 1.23e-01 | 1.80e-01 |
| ENSG00000104852 | 1.26e-09 | 9.83e-09 |
| ENSG00000104853 | 1.10e-01 | 1.63e-01 |
| ENSG00000104856 | 8.38e-01 | 8.77e-01 |
| ENSG00000104859 | 9.33e-06 | 3.71e-05 |
| ENSG00000104863 | 1.01e-01 | 1.52e-01 |
| ENSG00000104866 | 1.36e-03 | 3.49e-03 |
| ENSG00000104870 | 2.10e-07 | 1.12e-06 |
| ENSG00000104872 | 1.16e-01 | 1.70e-01 |
| ENSG00000104879 | 2.76e-01 | 3.54e-01 |
| ENSG00000104880 | 6.61e-09 | 4.55e-08 |
| ENSG00000104881 | 1.15e-14 | 2.24e-13 |
| ENSG00000104883 | 4.79e-03 | 1.07e-02 |
| ENSG00000104884 | 2.65e-09 | 1.94e-08 |
| ENSG00000104885 | 2.83e-02 | 5.06e-02 |
| ENSG00000104886 | 5.00e-02 | 8.30e-02 |
| ENSG00000104888 | 1.82e-23 | 3.45e-21 |
| ENSG00000104889 | 1.36e-16 | 3.93e-15 |
| ENSG00000104892 | 4.37e-23 | 7.32e-21 |
| ENSG00000104894 | 2.40e-07 | 1.27e-06 |
| ENSG00000104897 | 2.82e-09 | 2.07e-08 |
| ENSG00000104899 | 6.53e-12 | 7.55e-11 |
| ENSG00000104901 | 5.42e-01 | 6.20e-01 |
| ENSG00000104903 | 2.71e-06 | 1.18e-05 |
| ENSG00000104904 | 4.39e-02 | 7.41e-02 |
| ENSG00000104907 | 1.37e-16 | 3.95e-15 |
| ENSG00000104915 | 1.03e-06 | 4.88e-06 |
| ENSG00000104918 | 6.04e-06 | 2.48e-05 |
| ENSG00000104921 | 1.36e-12 | 1.79e-11 |
| ENSG00000104936 | 8.10e-03 | 1.70e-02 |
| ENSG00000104938 | 4.55e-13 | 6.57e-12 |
| ENSG00000104941 | 1.60e-01 | 2.23e-01 |
| ENSG00000104946 | 1.26e-01 | 1.83e-01 |
| ENSG00000104951 | 9.77e-06 | 3.87e-05 |
| ENSG00000104953 | 5.63e-01 | 6.40e-01 |
| ENSG00000104957 | 5.67e-04 | 1.59e-03 |
| ENSG00000104960 | 1.96e-02 | 3.68e-02 |
| ENSG00000104964 | 2.41e-03 | 5.80e-03 |
| ENSG00000104967 | 6.21e-01 | 6.93e-01 |
| ENSG00000104969 | 5.75e-05 | 1.97e-04 |
| ENSG00000104970 | 8.73e-04 | 2.34e-03 |
| ENSG00000104972 | 8.89e-04 | 2.38e-03 |
| ENSG00000104973 | 1.45e-04 | 4.58e-04 |
| ENSG00000104974 | 9.22e-02 | 1.40e-01 |
| ENSG00000104976 | 7.17e-01 | 7.76e-01 |
| ENSG00000104979 | 1.39e-11 | 1.50e-10 |
| ENSG00000104980 | 1.47e-05 | 5.63e-05 |
| ENSG00000104983 | 4.57e-02 | 7.67e-02 |
| ENSG00000104998 | 2.23e-07 | 1.18e-06 |
| ENSG00000105011 | 1.72e-12 | 2.24e-11 |
| ENSG00000105048 | 2.80e-05 | 1.02e-04 |
| ENSG00000105053 | 1.34e-04 | 4.25e-04 |
| ENSG00000105058 | 2.83e-02 | 5.05e-02 |
| ENSG00000105063 | 1.54e-03 | 3.90e-03 |
| ENSG00000105072 | 1.86e-06 | 8.33e-06 |
| ENSG00000105085 | 5.04e-07 | 2.51e-06 |
| ENSG00000105088 | 4.48e-03 | 1.01e-02 |
| ENSG00000105122 | 1.23e-04 | 3.93e-04 |
| ENSG00000105127 | 9.48e-09 | 6.34e-08 |
| ENSG00000105131 | 7.78e-10 | 6.26e-09 |
| ENSG00000105135 | 6.89e-01 | 7.53e-01 |
| ENSG00000105136 | 9.12e-01 | 9.34e-01 |
| ENSG00000105137 | 8.86e-01 | 9.15e-01 |
| ENSG00000105141 | 8.70e-03 | 1.81e-02 |
| ENSG00000105143 | 4.99e-01 | 5.80e-01 |
| ENSG00000105146 | 1.11e-03 | 2.91e-03 |
| ENSG00000105171 | 3.41e-01 | 4.23e-01 |
| ENSG00000105173 | 2.45e-18 | 1.04e-16 |
| ENSG00000105176 | 8.14e-14 | 1.34e-12 |
| ENSG00000105185 | 2.21e-20 | 1.62e-18 |
| ENSG00000105186 | 9.63e-07 | 4.56e-06 |
| ENSG00000105193 | 1.01e-06 | 4.75e-06 |
| ENSG00000105197 | 2.19e-14 | 4.05e-13 |
| ENSG00000105202 | 3.31e-13 | 4.88e-12 |
| ENSG00000105204 | 5.74e-01 | 6.50e-01 |
| ENSG00000105205 | 1.39e-01 | 1.99e-01 |
| ENSG00000105219 | 1.73e-24 | 4.64e-22 |
| ENSG00000105220 | 9.98e-11 | 9.34e-10 |
| ENSG00000105221 | 1.31e-06 | 6.05e-06 |
| ENSG00000105223 | 1.58e-01 | 2.22e-01 |
| ENSG00000105227 | 6.01e-11 | 5.86e-10 |
| ENSG00000105229 | 2.22e-03 | 5.40e-03 |
| ENSG00000105245 | 3.87e-02 | 6.64e-02 |
| ENSG00000105246 | 4.18e-08 | 2.52e-07 |
| ENSG00000105248 | 7.15e-01 | 7.75e-01 |
| ENSG00000105251 | 4.84e-07 | 2.42e-06 |
| ENSG00000105254 | 9.18e-06 | 3.66e-05 |
| ENSG00000105255 | 1.61e-02 | 3.09e-02 |
| ENSG00000105258 | 1.47e-05 | 5.62e-05 |
| ENSG00000105261 | 2.61e-04 | 7.81e-04 |
| ENSG00000105270 | 2.90e-08 | 1.79e-07 |
| ENSG00000105278 | 1.24e-08 | 8.12e-08 |
| ENSG00000105281 | 1.47e-12 | 1.93e-11 |
| ENSG00000105287 | 4.05e-01 | 4.88e-01 |
| ENSG00000105289 | 1.54e-08 | 9.96e-08 |
| ENSG00000105290 | 2.41e-13 | 3.65e-12 |
| ENSG00000105298 | 2.16e-04 | 6.59e-04 |
| ENSG00000105321 | 5.08e-01 | 5.88e-01 |
| ENSG00000105323 | 1.62e-03 | 4.07e-03 |
| ENSG00000105325 | 1.39e-05 | 5.36e-05 |
| ENSG00000105327 | 3.92e-09 | 2.80e-08 |
| ENSG00000105329 | 3.84e-02 | 6.60e-02 |
| ENSG00000105339 | 3.99e-04 | 1.15e-03 |
| ENSG00000105341 | 6.61e-01 | 7.28e-01 |
| ENSG00000105352 | 2.99e-03 | 7.03e-03 |
| ENSG00000105355 | 1.92e-02 | 3.61e-02 |
| ENSG00000105357 | 3.55e-02 | 6.16e-02 |
| ENSG00000105364 | 5.60e-09 | 3.90e-08 |
| ENSG00000105366 | 2.03e-15 | 4.54e-14 |
| ENSG00000105369 | 5.65e-13 | 7.97e-12 |
| ENSG00000105372 | 2.11e-07 | 1.12e-06 |
| ENSG00000105373 | 6.70e-01 | 7.36e-01 |
| ENSG00000105374 | 1.21e-05 | 4.70e-05 |
| ENSG00000105376 | 1.02e-03 | 2.69e-03 |
| ENSG00000105379 | 4.77e-03 | 1.06e-02 |
| ENSG00000105383 | 1.75e-07 | 9.45e-07 |
| ENSG00000105388 | 5.83e-01 | 6.58e-01 |
| ENSG00000105392 | 1.42e-01 | 2.03e-01 |
| ENSG00000105393 | 4.95e-02 | 8.22e-02 |
| ENSG00000105397 | 5.93e-06 | 2.44e-05 |
| ENSG00000105398 | 6.16e-01 | 6.88e-01 |
| ENSG00000105401 | 2.08e-06 | 9.24e-06 |
| ENSG00000105402 | 2.24e-02 | 4.12e-02 |
| ENSG00000105404 | 1.50e-01 | 2.13e-01 |
| ENSG00000105409 | 1.68e-07 | 9.10e-07 |
| ENSG00000105419 | 9.29e-04 | 2.47e-03 |
| ENSG00000105426 | 2.32e-06 | 1.02e-05 |
| ENSG00000105427 | 2.95e-01 | 3.76e-01 |
| ENSG00000105429 | 6.61e-06 | 2.69e-05 |
| ENSG00000105438 | 9.38e-03 | 1.93e-02 |
| ENSG00000105443 | 7.68e-04 | 2.08e-03 |
| ENSG00000105447 | 1.75e-13 | 2.71e-12 |
| ENSG00000105464 | 6.29e-29 | 8.50e-26 |
| ENSG00000105467 | 1.48e-02 | 2.87e-02 |
| ENSG00000105472 | 1.19e-04 | 3.83e-04 |
| ENSG00000105479 | 1.34e-07 | 7.41e-07 |
| ENSG00000105483 | 1.21e-02 | 2.41e-02 |
| ENSG00000105486 | 1.30e-12 | 1.72e-11 |
| ENSG00000105492 | 3.32e-15 | 7.16e-14 |
| ENSG00000105497 | 1.36e-06 | 6.24e-06 |
| ENSG00000105499 | 2.23e-01 | 2.96e-01 |
| ENSG00000105501 | 1.56e-02 | 3.01e-02 |
| ENSG00000105509 | 9.59e-01 | 9.70e-01 |
| ENSG00000105514 | 2.29e-12 | 2.90e-11 |
| ENSG00000105516 | 3.13e-03 | 7.33e-03 |
| ENSG00000105518 | 1.22e-04 | 3.90e-04 |
| ENSG00000105519 | 1.09e-02 | 2.20e-02 |
| ENSG00000105520 | 1.19e-02 | 2.38e-02 |
| ENSG00000105523 | 9.09e-08 | 5.17e-07 |
| ENSG00000105538 | 5.66e-01 | 6.43e-01 |
| ENSG00000105549 | 2.47e-01 | 3.23e-01 |
| ENSG00000105550 | 7.42e-03 | 1.57e-02 |
| ENSG00000105552 | 7.67e-05 | 2.56e-04 |
| ENSG00000105556 | 8.73e-12 | 9.87e-11 |
| ENSG00000105559 | 5.81e-03 | 1.26e-02 |
| ENSG00000105568 | 3.49e-01 | 4.31e-01 |
| ENSG00000105576 | 5.64e-16 | 1.43e-14 |
| ENSG00000105583 | 4.55e-01 | 5.38e-01 |
| ENSG00000105605 | 2.97e-13 | 4.43e-12 |
| ENSG00000105607 | 5.32e-03 | 1.17e-02 |
| ENSG00000105609 | 1.03e-19 | 6.21e-18 |
| ENSG00000105610 | 9.36e-05 | 3.07e-04 |
| ENSG00000105612 | 7.76e-03 | 1.63e-02 |
| ENSG00000105613 | 5.28e-01 | 6.07e-01 |
| ENSG00000105617 | 4.92e-02 | 8.17e-02 |
| ENSG00000105618 | 1.38e-05 | 5.32e-05 |
| ENSG00000105619 | 1.09e-01 | 1.62e-01 |
| ENSG00000105639 | 3.27e-02 | 5.73e-02 |
| ENSG00000105640 | 1.51e-05 | 5.79e-05 |
| ENSG00000105641 | 2.98e-01 | 3.78e-01 |
| ENSG00000105642 | 1.69e-03 | 4.22e-03 |
| ENSG00000105643 | 6.93e-01 | 7.56e-01 |
| ENSG00000105647 | 5.07e-06 | 2.11e-05 |
| ENSG00000105649 | 2.54e-03 | 6.09e-03 |
| ENSG00000105650 | 1.28e-10 | 1.18e-09 |
| ENSG00000105655 | 1.92e-03 | 4.73e-03 |
| ENSG00000105656 | 4.08e-02 | 6.95e-02 |
| ENSG00000105662 | 5.63e-01 | 6.40e-01 |
| ENSG00000105664 | 1.20e-22 | 1.73e-20 |
| ENSG00000105668 | 9.27e-06 | 3.69e-05 |
| ENSG00000105669 | 9.41e-02 | 1.43e-01 |
| ENSG00000105671 | 5.94e-06 | 2.44e-05 |
| ENSG00000105672 | 9.87e-01 | 9.90e-01 |
| ENSG00000105675 | 8.54e-01 | 8.90e-01 |
| ENSG00000105676 | 3.04e-08 | 1.87e-07 |
| ENSG00000105677 | 8.34e-11 | 7.94e-10 |
| ENSG00000105679 | 2.02e-01 | 2.73e-01 |
| ENSG00000105694 | 8.88e-04 | 2.37e-03 |
| ENSG00000105695 | 5.09e-06 | 2.12e-05 |
| ENSG00000105696 | 9.06e-19 | 4.33e-17 |
| ENSG00000105697 | 3.22e-07 | 1.66e-06 |
| ENSG00000105698 | 6.85e-02 | 1.09e-01 |
| ENSG00000105699 | 5.63e-01 | 6.39e-01 |
| ENSG00000105700 | 2.21e-06 | 9.77e-06 |
| ENSG00000105701 | 5.28e-01 | 6.07e-01 |
| ENSG00000105705 | 1.02e-03 | 2.69e-03 |
| ENSG00000105707 | 1.71e-05 | 6.48e-05 |
| ENSG00000105708 | 2.55e-01 | 3.32e-01 |
| ENSG00000105711 | 6.49e-08 | 3.78e-07 |
| ENSG00000105717 | 9.69e-14 | 1.58e-12 |
| ENSG00000105722 | 9.12e-12 | 1.03e-10 |
| ENSG00000105723 | 7.08e-01 | 7.69e-01 |
| ENSG00000105726 | 2.21e-06 | 9.78e-06 |
| ENSG00000105732 | 1.44e-05 | 5.54e-05 |
| ENSG00000105737 | 5.69e-13 | 8.02e-12 |
| ENSG00000105738 | 8.23e-06 | 3.31e-05 |
| ENSG00000105750 | 2.63e-03 | 6.28e-03 |
| ENSG00000105755 | 3.67e-17 | 1.20e-15 |
| ENSG00000105767 | 9.43e-01 | 9.58e-01 |
| ENSG00000105771 | 6.40e-14 | 1.07e-12 |
| ENSG00000105778 | 6.76e-05 | 2.28e-04 |
| ENSG00000105784 | 2.58e-20 | 1.84e-18 |
| ENSG00000105792 | 2.23e-05 | 8.28e-05 |
| ENSG00000105793 | 1.40e-08 | 9.12e-08 |
| ENSG00000105808 | 1.15e-01 | 1.70e-01 |
| ENSG00000105810 | 2.32e-06 | 1.02e-05 |
| ENSG00000105819 | 1.47e-03 | 3.73e-03 |
| ENSG00000105821 | 7.95e-15 | 1.59e-13 |
| ENSG00000105825 | 4.16e-03 | 9.42e-03 |
| ENSG00000105829 | 1.47e-03 | 3.74e-03 |
| ENSG00000105835 | 6.19e-03 | 1.34e-02 |
| ENSG00000105849 | 5.04e-13 | 7.20e-12 |
| ENSG00000105851 | 1.37e-12 | 1.81e-11 |
| ENSG00000105852 | 7.15e-01 | 7.74e-01 |
| ENSG00000105854 | 5.37e-02 | 8.81e-02 |
| ENSG00000105855 | 3.14e-07 | 1.62e-06 |
| ENSG00000105856 | 1.46e-02 | 2.83e-02 |
| ENSG00000105865 | 2.25e-16 | 6.23e-15 |
| ENSG00000105866 | 3.32e-01 | 4.14e-01 |
| ENSG00000105875 | 7.99e-01 | 8.45e-01 |
| ENSG00000105877 | 5.84e-01 | 6.59e-01 |
| ENSG00000105879 | 9.31e-09 | 6.24e-08 |
| ENSG00000105880 | 2.74e-04 | 8.16e-04 |
| ENSG00000105887 | 1.10e-01 | 1.63e-01 |
| ENSG00000105889 | 5.43e-01 | 6.21e-01 |
| ENSG00000105894 | 7.60e-26 | 3.59e-23 |
| ENSG00000105926 | 5.61e-12 | 6.58e-11 |
| ENSG00000105928 | 4.21e-01 | 5.04e-01 |
| ENSG00000105929 | 9.45e-09 | 6.32e-08 |
| ENSG00000105939 | 1.33e-01 | 1.92e-01 |
| ENSG00000105948 | 2.35e-18 | 1.01e-16 |
| ENSG00000105953 | 1.06e-05 | 4.17e-05 |
| ENSG00000105954 | 1.03e-03 | 2.72e-03 |
| ENSG00000105963 | 1.56e-02 | 3.01e-02 |
| ENSG00000105967 | 1.44e-02 | 2.80e-02 |
| ENSG00000105968 | 5.51e-04 | 1.54e-03 |
| ENSG00000105971 | 6.01e-02 | 9.72e-02 |
| ENSG00000105974 | 5.84e-09 | 4.06e-08 |
| ENSG00000105976 | 4.83e-21 | 4.26e-19 |
| ENSG00000105982 | 4.21e-15 | 8.87e-14 |
| ENSG00000105983 | 1.03e-15 | 2.45e-14 |
| ENSG00000105988 | 6.79e-09 | 4.66e-08 |
| ENSG00000105989 | 3.03e-33 | 1.27e-29 |
| ENSG00000105991 | 1.22e-02 | 2.42e-02 |
| ENSG00000105993 | 5.39e-06 | 2.23e-05 |
| ENSG00000105996 | 1.95e-02 | 3.67e-02 |
| ENSG00000105997 | 3.56e-02 | 6.18e-02 |
| ENSG00000106003 | 4.77e-11 | 4.73e-10 |
| ENSG00000106004 | 2.89e-04 | 8.58e-04 |
| ENSG00000106006 | 1.49e-03 | 3.78e-03 |
| ENSG00000106009 | 2.84e-13 | 4.25e-12 |
| ENSG00000106012 | 1.89e-02 | 3.56e-02 |
| ENSG00000106013 | 1.32e-04 | 4.21e-04 |
| ENSG00000106018 | 2.17e-05 | 8.06e-05 |
| ENSG00000106025 | 6.78e-01 | 7.43e-01 |
| ENSG00000106028 | 5.64e-15 | 1.15e-13 |
| ENSG00000106031 | 1.12e-02 | 2.25e-02 |
| ENSG00000106034 | 2.62e-13 | 3.94e-12 |
| ENSG00000106038 | 1.11e-04 | 3.59e-04 |
| ENSG00000106049 | 2.08e-06 | 9.26e-06 |
| ENSG00000106052 | 5.84e-01 | 6.58e-01 |
| ENSG00000106066 | 2.09e-06 | 9.30e-06 |
| ENSG00000106069 | 2.56e-01 | 3.33e-01 |
| ENSG00000106070 | 1.99e-12 | 2.55e-11 |
| ENSG00000106077 | 3.17e-04 | 9.33e-04 |
| ENSG00000106078 | 7.70e-05 | 2.56e-04 |
| ENSG00000106080 | 4.90e-12 | 5.81e-11 |
| ENSG00000106086 | 1.57e-10 | 1.42e-09 |
| ENSG00000106089 | 4.27e-20 | 2.86e-18 |
| ENSG00000106100 | 7.70e-09 | 5.24e-08 |
| ENSG00000106105 | 1.38e-22 | 1.97e-20 |
| ENSG00000106113 | 3.16e-01 | 3.97e-01 |
| ENSG00000106123 | 4.79e-10 | 3.99e-09 |
| ENSG00000106125 | 7.17e-10 | 5.80e-09 |
| ENSG00000106128 | 5.77e-01 | 6.52e-01 |
| ENSG00000106133 | 3.05e-11 | 3.13e-10 |
| ENSG00000106144 | 3.46e-13 | 5.07e-12 |
| ENSG00000106153 | 4.56e-08 | 2.73e-07 |
| ENSG00000106178 | 9.73e-08 | 5.49e-07 |
| ENSG00000106211 | 7.25e-01 | 7.83e-01 |
| ENSG00000106236 | 3.19e-03 | 7.45e-03 |
| ENSG00000106244 | 1.31e-09 | 1.01e-08 |
| ENSG00000106245 | 2.48e-02 | 4.51e-02 |
| ENSG00000106246 | 5.65e-10 | 4.66e-09 |
| ENSG00000106258 | 2.07e-01 | 2.78e-01 |
| ENSG00000106261 | 3.00e-04 | 8.87e-04 |
| ENSG00000106263 | 3.23e-18 | 1.34e-16 |
| ENSG00000106266 | 3.10e-07 | 1.60e-06 |
| ENSG00000106268 | 2.99e-12 | 3.70e-11 |
| ENSG00000106278 | 1.32e-13 | 2.10e-12 |
| ENSG00000106290 | 1.89e-05 | 7.10e-05 |
| ENSG00000106299 | 1.47e-13 | 2.31e-12 |
| ENSG00000106302 | 6.03e-02 | 9.74e-02 |
| ENSG00000106304 | 8.31e-02 | 1.28e-01 |
| ENSG00000106305 | 2.54e-04 | 7.64e-04 |
| ENSG00000106327 | 1.49e-19 | 8.70e-18 |
| ENSG00000106328 | 9.86e-01 | 9.90e-01 |
| ENSG00000106330 | 7.42e-06 | 3.00e-05 |
| ENSG00000106331 | 1.47e-04 | 4.64e-04 |
| ENSG00000106333 | 9.89e-02 | 1.49e-01 |
| ENSG00000106336 | 1.53e-01 | 2.15e-01 |
| ENSG00000106341 | 3.38e-01 | 4.20e-01 |
| ENSG00000106344 | 1.83e-19 | 1.04e-17 |
| ENSG00000106346 | 1.35e-02 | 2.64e-02 |
| ENSG00000106348 | 2.38e-18 | 1.02e-16 |
| ENSG00000106351 | 5.09e-12 | 6.01e-11 |
| ENSG00000106355 | 6.80e-10 | 5.51e-09 |
| ENSG00000106366 | 2.51e-17 | 8.50e-16 |
| ENSG00000106367 | 3.31e-11 | 3.37e-10 |
| ENSG00000106384 | 1.09e-02 | 2.20e-02 |
| ENSG00000106392 | 6.13e-04 | 1.70e-03 |
| ENSG00000106397 | 8.56e-16 | 2.07e-14 |
| ENSG00000106399 | 2.16e-09 | 1.61e-08 |
| ENSG00000106400 | 1.31e-01 | 1.90e-01 |
| ENSG00000106404 | 9.91e-01 | 9.94e-01 |
| ENSG00000106415 | 5.50e-03 | 1.21e-02 |
| ENSG00000106436 | 4.21e-02 | 7.15e-02 |
| ENSG00000106443 | 1.21e-13 | 1.93e-12 |
| ENSG00000106459 | 2.22e-04 | 6.77e-04 |
| ENSG00000106460 | 3.53e-01 | 4.36e-01 |
| ENSG00000106462 | 2.20e-18 | 9.46e-17 |
| ENSG00000106477 | 2.79e-10 | 2.42e-09 |
| ENSG00000106479 | 9.72e-01 | 9.79e-01 |
| ENSG00000106483 | 2.74e-13 | 4.11e-12 |
| ENSG00000106484 | 2.66e-17 | 8.95e-16 |
| ENSG00000106511 | 7.06e-07 | 3.43e-06 |
| ENSG00000106524 | 3.23e-07 | 1.66e-06 |
| ENSG00000106526 | 9.18e-02 | 1.40e-01 |
| ENSG00000106536 | 6.63e-03 | 1.42e-02 |
| ENSG00000106537 | 2.92e-01 | 3.72e-01 |
| ENSG00000106538 | 2.31e-08 | 1.45e-07 |
| ENSG00000106541 | 1.10e-01 | 1.63e-01 |
| ENSG00000106546 | 8.27e-05 | 2.74e-04 |
| ENSG00000106554 | 1.45e-02 | 2.82e-02 |
| ENSG00000106560 | 9.32e-01 | 9.49e-01 |
| ENSG00000106565 | 3.85e-03 | 8.80e-03 |
| ENSG00000106571 | 1.78e-07 | 9.58e-07 |
| ENSG00000106588 | 3.27e-02 | 5.73e-02 |
| ENSG00000106591 | 7.61e-05 | 2.54e-04 |
| ENSG00000106603 | 6.27e-16 | 1.57e-14 |
| ENSG00000106605 | 1.39e-01 | 1.99e-01 |
| ENSG00000106608 | 1.30e-05 | 5.03e-05 |
| ENSG00000106609 | 8.76e-05 | 2.89e-04 |
| ENSG00000106610 | 8.68e-02 | 1.33e-01 |
| ENSG00000106615 | 9.46e-10 | 7.50e-09 |
| ENSG00000106617 | 3.07e-10 | 2.64e-09 |
| ENSG00000106624 | 3.43e-03 | 7.95e-03 |
| ENSG00000106628 | 6.14e-18 | 2.37e-16 |
| ENSG00000106631 | 5.37e-03 | 1.18e-02 |
| ENSG00000106633 | 2.34e-06 | 1.03e-05 |
| ENSG00000106635 | 1.32e-03 | 3.40e-03 |
| ENSG00000106636 | 3.26e-02 | 5.72e-02 |
| ENSG00000106638 | 6.68e-16 | 1.66e-14 |
| ENSG00000106665 | 8.75e-03 | 1.82e-02 |
| ENSG00000106682 | 1.34e-01 | 1.93e-01 |
| ENSG00000106683 | 3.83e-16 | 1.01e-14 |
| ENSG00000106686 | 2.85e-09 | 2.08e-08 |
| ENSG00000106688 | 5.48e-13 | 7.77e-12 |
| ENSG00000106689 | 3.27e-01 | 4.09e-01 |
| ENSG00000106692 | 5.02e-05 | 1.74e-04 |
| ENSG00000106701 | 6.13e-03 | 1.32e-02 |
| ENSG00000106714 | 9.96e-13 | 1.35e-11 |
| ENSG00000106723 | 8.64e-01 | 8.98e-01 |
| ENSG00000106733 | 1.20e-04 | 3.85e-04 |
| ENSG00000106771 | 6.34e-01 | 7.04e-01 |
| ENSG00000106772 | 9.01e-03 | 1.86e-02 |
| ENSG00000106780 | 1.41e-04 | 4.45e-04 |
| ENSG00000106785 | 5.45e-02 | 8.93e-02 |
| ENSG00000106789 | 6.20e-08 | 3.62e-07 |
| ENSG00000106799 | 1.19e-03 | 3.10e-03 |
| ENSG00000106803 | 2.30e-03 | 5.56e-03 |
| ENSG00000106804 | 7.38e-02 | 1.16e-01 |
| ENSG00000106809 | 2.07e-20 | 1.52e-18 |
| ENSG00000106819 | 4.23e-01 | 5.06e-01 |
| ENSG00000106823 | 2.95e-04 | 8.72e-04 |
| ENSG00000106829 | 4.00e-11 | 4.02e-10 |
| ENSG00000106852 | 7.50e-01 | 8.05e-01 |
| ENSG00000106853 | 2.26e-08 | 1.42e-07 |
| ENSG00000106868 | 9.46e-05 | 3.09e-04 |
| ENSG00000106927 | 7.62e-05 | 2.54e-04 |
| ENSG00000106948 | 6.24e-04 | 1.73e-03 |
| ENSG00000106952 | 2.07e-07 | 1.10e-06 |
| ENSG00000106976 | 2.29e-06 | 1.01e-05 |
| ENSG00000106991 | 4.62e-02 | 7.73e-02 |
| ENSG00000106992 | 4.59e-05 | 1.60e-04 |
| ENSG00000106993 | 4.73e-01 | 5.56e-01 |
| ENSG00000107014 | 2.04e-01 | 2.76e-01 |
| ENSG00000107018 | 3.47e-01 | 4.29e-01 |
| ENSG00000107020 | 9.44e-08 | 5.34e-07 |
| ENSG00000107021 | 7.29e-02 | 1.15e-01 |
| ENSG00000107036 | 1.41e-01 | 2.02e-01 |
| ENSG00000107077 | 7.81e-01 | 8.30e-01 |
| ENSG00000107099 | 1.99e-04 | 6.11e-04 |
| ENSG00000107104 | 9.96e-05 | 3.24e-04 |
| ENSG00000107105 | 5.41e-01 | 6.20e-01 |
| ENSG00000107130 | 9.84e-01 | 9.89e-01 |
| ENSG00000107140 | 8.52e-01 | 8.89e-01 |
| ENSG00000107147 | 3.16e-02 | 5.56e-02 |
| ENSG00000107159 | 3.15e-18 | 1.31e-16 |
| ENSG00000107164 | 3.80e-06 | 1.62e-05 |
| ENSG00000107165 | 2.31e-04 | 7.00e-04 |
| ENSG00000107175 | 7.18e-01 | 7.77e-01 |
| ENSG00000107185 | 1.57e-09 | 1.20e-08 |
| ENSG00000107186 | 4.20e-06 | 1.77e-05 |
| ENSG00000107187 | 1.07e-04 | 3.46e-04 |
| ENSG00000107201 | 6.14e-04 | 1.70e-03 |
| ENSG00000107223 | 2.86e-02 | 5.10e-02 |
| ENSG00000107242 | 2.63e-11 | 2.72e-10 |
| ENSG00000107249 | 1.32e-02 | 2.59e-02 |
| ENSG00000107262 | 4.43e-03 | 9.95e-03 |
| ENSG00000107263 | 2.78e-04 | 8.27e-04 |
| ENSG00000107281 | 9.27e-04 | 2.47e-03 |
| ENSG00000107282 | 9.38e-11 | 8.84e-10 |
| ENSG00000107290 | 1.96e-01 | 2.67e-01 |
| ENSG00000107295 | 3.87e-17 | 1.26e-15 |
| ENSG00000107317 | 1.71e-06 | 7.71e-06 |
| ENSG00000107331 | 6.75e-03 | 1.44e-02 |
| ENSG00000107338 | 2.56e-19 | 1.39e-17 |
| ENSG00000107341 | 6.58e-10 | 5.35e-09 |
| ENSG00000107362 | 2.13e-02 | 3.95e-02 |
| ENSG00000107371 | 1.02e-20 | 8.34e-19 |
| ENSG00000107372 | 1.98e-01 | 2.69e-01 |
| ENSG00000107404 | 6.04e-07 | 2.97e-06 |
| ENSG00000107438 | 1.29e-06 | 5.96e-06 |
| ENSG00000107443 | 1.39e-17 | 4.97e-16 |
| ENSG00000107447 | 3.41e-01 | 4.23e-01 |
| ENSG00000107485 | 3.96e-02 | 6.78e-02 |
| ENSG00000107518 | 5.94e-11 | 5.79e-10 |
| ENSG00000107521 | 8.28e-07 | 3.98e-06 |
| ENSG00000107537 | 5.74e-15 | 1.17e-13 |
| ENSG00000107551 | 8.27e-05 | 2.74e-04 |
| ENSG00000107554 | 8.80e-01 | 9.10e-01 |
| ENSG00000107560 | 3.09e-01 | 3.89e-01 |
| ENSG00000107562 | 1.19e-22 | 1.72e-20 |
| ENSG00000107566 | 3.17e-01 | 3.99e-01 |
| ENSG00000107581 | 9.86e-03 | 2.02e-02 |
| ENSG00000107593 | 8.85e-04 | 2.37e-03 |
| ENSG00000107611 | 1.81e-02 | 3.42e-02 |
| ENSG00000107614 | 9.29e-01 | 9.47e-01 |
| ENSG00000107625 | 1.45e-03 | 3.70e-03 |
| ENSG00000107643 | 9.50e-01 | 9.63e-01 |
| ENSG00000107651 | 2.21e-02 | 4.08e-02 |
| ENSG00000107669 | 4.01e-04 | 1.16e-03 |
| ENSG00000107672 | 2.61e-02 | 4.72e-02 |
| ENSG00000107679 | 2.78e-02 | 4.98e-02 |
| ENSG00000107719 | 4.50e-17 | 1.45e-15 |
| ENSG00000107731 | 2.65e-05 | 9.71e-05 |
| ENSG00000107736 | 5.98e-10 | 4.90e-09 |
| ENSG00000107738 | 7.97e-19 | 3.87e-17 |
| ENSG00000107742 | 1.78e-06 | 8.03e-06 |
| ENSG00000107745 | 3.56e-03 | 8.21e-03 |
| ENSG00000107758 | 1.06e-05 | 4.17e-05 |
| ENSG00000107771 | 6.11e-01 | 6.83e-01 |
| ENSG00000107779 | 1.64e-04 | 5.12e-04 |
| ENSG00000107789 | 6.75e-01 | 7.40e-01 |
| ENSG00000107796 | 3.90e-04 | 1.13e-03 |
| ENSG00000107798 | 2.10e-02 | 3.90e-02 |
| ENSG00000107807 | 3.74e-20 | 2.55e-18 |
| ENSG00000107815 | 2.73e-22 | 3.56e-20 |
| ENSG00000107816 | 2.39e-02 | 4.37e-02 |
| ENSG00000107819 | 2.04e-21 | 2.09e-19 |
| ENSG00000107821 | 8.70e-01 | 9.03e-01 |
| ENSG00000107829 | 1.88e-04 | 5.80e-04 |
| ENSG00000107831 | 2.89e-04 | 8.57e-04 |
| ENSG00000107833 | 2.26e-14 | 4.16e-13 |
| ENSG00000107854 | 5.14e-01 | 5.93e-01 |
| ENSG00000107859 | 3.97e-05 | 1.40e-04 |
| ENSG00000107862 | 5.69e-05 | 1.95e-04 |
| ENSG00000107863 | 1.52e-03 | 3.84e-03 |
| ENSG00000107864 | 5.61e-21 | 4.89e-19 |
| ENSG00000107872 | 3.54e-01 | 4.36e-01 |
| ENSG00000107874 | 4.11e-02 | 6.99e-02 |
| ENSG00000107882 | 4.22e-01 | 5.05e-01 |
| ENSG00000107890 | 3.21e-01 | 4.02e-01 |
| ENSG00000107897 | 7.73e-01 | 8.24e-01 |
| ENSG00000107902 | 3.35e-05 | 1.20e-04 |
| ENSG00000107929 | 4.53e-06 | 1.90e-05 |
| ENSG00000107937 | 2.73e-23 | 4.90e-21 |
| ENSG00000107938 | 2.39e-04 | 7.21e-04 |
| ENSG00000107949 | 6.85e-08 | 3.97e-07 |
| ENSG00000107951 | 2.53e-09 | 1.86e-08 |
| ENSG00000107954 | 2.27e-09 | 1.69e-08 |
| ENSG00000107957 | 1.78e-01 | 2.45e-01 |
| ENSG00000107959 | 1.57e-08 | 1.02e-07 |
| ENSG00000107960 | 1.56e-01 | 2.19e-01 |
| ENSG00000107968 | 7.45e-05 | 2.49e-04 |
| ENSG00000107984 | 6.84e-05 | 2.30e-04 |
| ENSG00000108001 | 1.92e-01 | 2.62e-01 |
| ENSG00000108010 | 1.23e-14 | 2.38e-13 |
| ENSG00000108018 | 6.34e-18 | 2.44e-16 |
| ENSG00000108021 | 4.32e-05 | 1.52e-04 |
| ENSG00000108039 | 2.95e-01 | 3.76e-01 |
| ENSG00000108055 | 3.27e-02 | 5.73e-02 |
| ENSG00000108061 | 2.57e-07 | 1.35e-06 |
| ENSG00000108064 | 5.88e-02 | 9.53e-02 |
| ENSG00000108091 | 4.22e-02 | 7.15e-02 |
| ENSG00000108094 | 1.19e-03 | 3.09e-03 |
| ENSG00000108100 | 6.22e-01 | 6.93e-01 |
| ENSG00000108106 | 1.62e-18 | 7.22e-17 |
| ENSG00000108107 | 2.87e-08 | 1.77e-07 |
| ENSG00000108175 | 7.47e-08 | 4.30e-07 |
| ENSG00000108176 | 7.96e-02 | 1.24e-01 |
| ENSG00000108179 | 1.43e-05 | 5.49e-05 |
| ENSG00000108187 | 4.40e-18 | 1.76e-16 |
| ENSG00000108219 | 4.32e-01 | 5.15e-01 |
| ENSG00000108231 | 1.90e-25 | 7.61e-23 |
| ENSG00000108239 | 1.55e-08 | 1.00e-07 |
| ENSG00000108242 | 9.23e-08 | 5.24e-07 |
| ENSG00000108244 | 6.47e-19 | 3.22e-17 |
| ENSG00000108255 | 1.54e-01 | 2.17e-01 |
| ENSG00000108256 | 3.56e-03 | 8.22e-03 |
| ENSG00000108262 | 1.33e-09 | 1.03e-08 |
| ENSG00000108298 | 4.77e-04 | 1.35e-03 |
| ENSG00000108306 | 4.95e-01 | 5.76e-01 |
| ENSG00000108309 | 6.08e-10 | 4.97e-09 |
| ENSG00000108312 | 2.73e-01 | 3.51e-01 |
| ENSG00000108342 | 3.64e-10 | 3.10e-09 |
| ENSG00000108344 | 2.62e-05 | 9.60e-05 |
| ENSG00000108349 | 1.42e-04 | 4.48e-04 |
| ENSG00000108352 | 3.46e-08 | 2.11e-07 |
| ENSG00000108370 | 1.09e-20 | 8.87e-19 |
| ENSG00000108375 | 1.03e-15 | 2.45e-14 |
| ENSG00000108379 | 3.04e-19 | 1.62e-17 |
| ENSG00000108381 | 1.62e-23 | 3.20e-21 |
| ENSG00000108384 | 8.75e-09 | 5.89e-08 |
| ENSG00000108387 | 5.32e-01 | 6.11e-01 |
| ENSG00000108389 | 4.98e-01 | 5.79e-01 |
| ENSG00000108395 | 7.52e-03 | 1.59e-02 |
| ENSG00000108405 | 2.01e-18 | 8.74e-17 |
| ENSG00000108406 | 6.85e-04 | 1.88e-03 |
| ENSG00000108417 | 1.60e-13 | 2.49e-12 |
| ENSG00000108423 | 5.78e-03 | 1.26e-02 |
| ENSG00000108424 | 8.39e-07 | 4.03e-06 |
| ENSG00000108433 | 4.72e-04 | 1.34e-03 |
| ENSG00000108439 | 1.46e-12 | 1.92e-11 |
| ENSG00000108443 | 1.34e-01 | 1.93e-01 |
| ENSG00000108448 | 1.70e-01 | 2.37e-01 |
| ENSG00000108452 | 1.39e-02 | 2.72e-02 |
| ENSG00000108465 | 1.11e-07 | 6.21e-07 |
| ENSG00000108468 | 1.07e-02 | 2.17e-02 |
| ENSG00000108469 | 6.89e-01 | 7.53e-01 |
| ENSG00000108474 | 1.56e-07 | 8.50e-07 |
| ENSG00000108479 | 6.09e-13 | 8.54e-12 |
| ENSG00000108506 | 1.46e-08 | 9.46e-08 |
| ENSG00000108509 | 2.26e-15 | 5.01e-14 |
| ENSG00000108510 | 5.64e-01 | 6.40e-01 |
| ENSG00000108511 | 3.54e-05 | 1.27e-04 |
| ENSG00000108515 | 4.09e-05 | 1.44e-04 |
| ENSG00000108518 | 3.71e-02 | 6.41e-02 |
| ENSG00000108523 | 1.08e-06 | 5.08e-06 |
| ENSG00000108528 | 4.12e-05 | 1.45e-04 |
| ENSG00000108551 | 7.52e-04 | 2.05e-03 |
| ENSG00000108556 | 3.65e-01 | 4.47e-01 |
| ENSG00000108557 | 1.04e-09 | 8.19e-09 |
| ENSG00000108559 | 1.16e-05 | 4.55e-05 |
| ENSG00000108561 | 6.81e-04 | 1.87e-03 |
| ENSG00000108576 | 2.47e-01 | 3.23e-01 |
| ENSG00000108578 | 4.00e-03 | 9.10e-03 |
| ENSG00000108582 | 6.32e-03 | 1.36e-02 |
| ENSG00000108587 | 8.05e-01 | 8.50e-01 |
| ENSG00000108588 | 2.73e-04 | 8.15e-04 |
| ENSG00000108590 | 8.31e-01 | 8.71e-01 |
| ENSG00000108591 | 3.69e-02 | 6.38e-02 |
| ENSG00000108592 | 6.40e-05 | 2.17e-04 |
| ENSG00000108599 | 1.78e-03 | 4.42e-03 |
| ENSG00000108602 | 9.70e-03 | 1.99e-02 |
| ENSG00000108604 | 1.91e-09 | 1.43e-08 |
| ENSG00000108622 | 1.54e-02 | 2.98e-02 |
| ENSG00000108639 | 5.50e-05 | 1.89e-04 |
| ENSG00000108641 | 3.42e-10 | 2.93e-09 |
| ENSG00000108651 | 3.17e-11 | 3.24e-10 |
| ENSG00000108654 | 8.18e-03 | 1.71e-02 |
| ENSG00000108666 | 3.36e-09 | 2.42e-08 |
| ENSG00000108669 | 5.04e-08 | 3.00e-07 |
| ENSG00000108671 | 2.88e-04 | 8.54e-04 |
| ENSG00000108679 | 7.36e-02 | 1.16e-01 |
| ENSG00000108684 | 8.85e-02 | 1.36e-01 |
| ENSG00000108688 | 1.55e-02 | 2.99e-02 |
| ENSG00000108691 | 2.11e-01 | 2.83e-01 |
| ENSG00000108700 | 1.29e-09 | 1.00e-08 |
| ENSG00000108702 | 8.67e-02 | 1.33e-01 |
| ENSG00000108733 | 3.97e-02 | 6.78e-02 |
| ENSG00000108759 | 1.32e-07 | 7.31e-07 |
| ENSG00000108771 | 3.11e-02 | 5.48e-02 |
| ENSG00000108773 | 1.86e-20 | 1.38e-18 |
| ENSG00000108774 | 5.89e-02 | 9.54e-02 |
| ENSG00000108784 | 7.53e-02 | 1.18e-01 |
| ENSG00000108785 | 1.74e-08 | 1.11e-07 |
| ENSG00000108786 | 9.39e-02 | 1.43e-01 |
| ENSG00000108788 | 1.62e-05 | 6.14e-05 |
| ENSG00000108797 | 1.40e-01 | 2.00e-01 |
| ENSG00000108798 | 1.16e-06 | 5.43e-06 |
| ENSG00000108799 | 1.84e-03 | 4.56e-03 |
| ENSG00000108813 | 4.19e-14 | 7.30e-13 |
| ENSG00000108819 | 6.55e-05 | 2.21e-04 |
| ENSG00000108821 | 2.44e-17 | 8.30e-16 |
| ENSG00000108823 | 5.89e-14 | 9.94e-13 |
| ENSG00000108825 | 9.17e-02 | 1.40e-01 |
| ENSG00000108826 | 3.34e-01 | 4.16e-01 |
| ENSG00000108828 | 9.61e-01 | 9.71e-01 |
| ENSG00000108829 | 8.24e-04 | 2.22e-03 |
| ENSG00000108830 | 4.02e-11 | 4.04e-10 |
| ENSG00000108839 | 2.13e-02 | 3.96e-02 |
| ENSG00000108840 | 3.77e-02 | 6.50e-02 |
| ENSG00000108846 | 2.03e-05 | 7.58e-05 |
| ENSG00000108848 | 3.43e-04 | 1.00e-03 |
| ENSG00000108849 | 2.59e-13 | 3.90e-12 |
| ENSG00000108852 | 7.01e-16 | 1.73e-14 |
| ENSG00000108854 | 7.63e-03 | 1.61e-02 |
| ENSG00000108861 | 1.85e-02 | 3.49e-02 |
| ENSG00000108878 | 6.61e-01 | 7.28e-01 |
| ENSG00000108883 | 9.16e-14 | 1.50e-12 |
| ENSG00000108924 | 1.92e-14 | 3.58e-13 |
| ENSG00000108932 | 5.12e-02 | 8.46e-02 |
| ENSG00000108946 | 1.23e-01 | 1.80e-01 |
| ENSG00000108947 | 7.56e-04 | 2.06e-03 |
| ENSG00000108950 | 4.95e-02 | 8.22e-02 |
| ENSG00000108953 | 1.61e-01 | 2.26e-01 |
| ENSG00000108958 | 2.59e-01 | 3.36e-01 |
| ENSG00000108960 | 1.67e-01 | 2.33e-01 |
| ENSG00000108961 | 7.58e-01 | 8.12e-01 |
| ENSG00000108963 | 1.50e-01 | 2.12e-01 |
| ENSG00000108984 | 5.10e-08 | 3.03e-07 |
| ENSG00000109016 | 1.40e-03 | 3.57e-03 |
| ENSG00000109046 | 8.27e-01 | 8.69e-01 |
| ENSG00000109047 | 8.56e-05 | 2.82e-04 |
| ENSG00000109061 | 3.63e-01 | 4.46e-01 |
| ENSG00000109062 | 7.86e-10 | 6.31e-09 |
| ENSG00000109063 | 2.16e-08 | 1.36e-07 |
| ENSG00000109065 | 2.23e-16 | 6.19e-15 |
| ENSG00000109066 | 2.20e-03 | 5.35e-03 |
| ENSG00000109072 | 9.72e-01 | 9.79e-01 |
| ENSG00000109079 | 3.41e-10 | 2.92e-09 |
| ENSG00000109083 | 1.73e-02 | 3.29e-02 |
| ENSG00000109084 | 1.52e-20 | 1.16e-18 |
| ENSG00000109089 | 1.23e-13 | 1.96e-12 |
| ENSG00000109099 | 3.64e-06 | 1.55e-05 |
| ENSG00000109101 | 2.98e-05 | 1.08e-04 |
| ENSG00000109103 | 6.95e-07 | 3.38e-06 |
| ENSG00000109107 | 4.39e-03 | 9.87e-03 |
| ENSG00000109111 | 5.10e-03 | 1.13e-02 |
| ENSG00000109113 | 6.08e-03 | 1.32e-02 |
| ENSG00000109118 | 1.88e-01 | 2.56e-01 |
| ENSG00000109132 | 8.78e-21 | 7.32e-19 |
| ENSG00000109133 | 7.66e-04 | 2.08e-03 |
| ENSG00000109158 | 4.90e-02 | 8.16e-02 |
| ENSG00000109163 | 8.62e-01 | 8.96e-01 |
| ENSG00000109171 | 7.66e-06 | 3.10e-05 |
| ENSG00000109180 | 9.34e-04 | 2.49e-03 |
| ENSG00000109181 | 1.06e-05 | 4.18e-05 |
| ENSG00000109182 | 9.00e-21 | 7.47e-19 |
| ENSG00000109184 | 2.30e-01 | 3.04e-01 |
| ENSG00000109189 | 1.68e-01 | 2.34e-01 |
| ENSG00000109193 | 6.50e-01 | 7.18e-01 |
| ENSG00000109205 | 7.57e-08 | 4.36e-07 |
| ENSG00000109220 | 4.30e-01 | 5.13e-01 |
| ENSG00000109255 | 3.10e-07 | 1.60e-06 |
| ENSG00000109265 | 4.11e-17 | 1.33e-15 |
| ENSG00000109270 | 3.69e-08 | 2.24e-07 |
| ENSG00000109272 | 2.98e-01 | 3.79e-01 |
| ENSG00000109320 | 3.15e-01 | 3.97e-01 |
| ENSG00000109321 | 3.22e-09 | 2.33e-08 |
| ENSG00000109323 | 6.85e-03 | 1.46e-02 |
| ENSG00000109332 | 1.61e-04 | 5.04e-04 |
| ENSG00000109339 | 5.64e-16 | 1.43e-14 |
| ENSG00000109381 | 8.77e-01 | 9.08e-01 |
| ENSG00000109390 | 4.34e-09 | 3.08e-08 |
| ENSG00000109424 | 5.26e-08 | 3.12e-07 |
| ENSG00000109436 | 1.22e-11 | 1.33e-10 |
| ENSG00000109445 | 4.01e-03 | 9.13e-03 |
| ENSG00000109452 | 4.94e-01 | 5.75e-01 |
| ENSG00000109458 | 7.06e-15 | 1.42e-13 |
| ENSG00000109466 | 5.30e-01 | 6.09e-01 |
| ENSG00000109471 | 2.52e-09 | 1.86e-08 |
| ENSG00000109472 | 2.24e-04 | 6.82e-04 |
| ENSG00000109475 | 2.49e-01 | 3.25e-01 |
| ENSG00000109501 | 1.26e-05 | 4.90e-05 |
| ENSG00000109511 | 9.87e-02 | 1.49e-01 |
| ENSG00000109519 | 9.78e-06 | 3.88e-05 |
| ENSG00000109534 | 1.34e-10 | 1.23e-09 |
| ENSG00000109536 | 2.78e-03 | 6.60e-03 |
| ENSG00000109572 | 1.26e-02 | 2.50e-02 |
| ENSG00000109576 | 2.44e-08 | 1.53e-07 |
| ENSG00000109586 | 1.19e-06 | 5.54e-06 |
| ENSG00000109606 | 1.02e-08 | 6.81e-08 |
| ENSG00000109610 | 1.49e-01 | 2.12e-01 |
| ENSG00000109618 | 4.00e-03 | 9.10e-03 |
| ENSG00000109625 | 2.75e-05 | 1.00e-04 |
| ENSG00000109654 | 1.85e-03 | 4.58e-03 |
| ENSG00000109667 | 5.31e-02 | 8.73e-02 |
| ENSG00000109670 | 7.16e-01 | 7.75e-01 |
| ENSG00000109674 | 9.72e-12 | 1.09e-10 |
| ENSG00000109680 | 1.19e-01 | 1.75e-01 |
| ENSG00000109684 | 4.06e-03 | 9.22e-03 |
| ENSG00000109685 | 4.67e-03 | 1.04e-02 |
| ENSG00000109686 | 1.80e-04 | 5.57e-04 |
| ENSG00000109689 | 3.90e-03 | 8.91e-03 |
| ENSG00000109705 | 3.33e-05 | 1.20e-04 |
| ENSG00000109736 | 1.38e-07 | 7.58e-07 |
| ENSG00000109738 | 2.88e-01 | 3.68e-01 |
| ENSG00000109743 | 5.96e-10 | 4.89e-09 |
| ENSG00000109756 | 8.98e-03 | 1.86e-02 |
| ENSG00000109758 | 1.02e-02 | 2.07e-02 |
| ENSG00000109762 | 1.11e-02 | 2.24e-02 |
| ENSG00000109771 | 7.59e-03 | 1.60e-02 |
| ENSG00000109775 | 3.38e-04 | 9.90e-04 |
| ENSG00000109787 | 5.00e-08 | 2.98e-07 |
| ENSG00000109790 | 4.82e-01 | 5.65e-01 |
| ENSG00000109794 | 9.46e-09 | 6.32e-08 |
| ENSG00000109805 | 9.80e-11 | 9.19e-10 |
| ENSG00000109814 | 9.61e-24 | 2.10e-21 |
| ENSG00000109819 | 2.24e-16 | 6.23e-15 |
| ENSG00000109832 | 3.01e-10 | 2.60e-09 |
| ENSG00000109846 | 2.11e-10 | 1.87e-09 |
| ENSG00000109851 | 3.98e-02 | 6.81e-02 |
| ENSG00000109854 | 2.13e-04 | 6.50e-04 |
| ENSG00000109861 | 4.86e-01 | 5.68e-01 |
| ENSG00000109881 | 1.51e-11 | 1.63e-10 |
| ENSG00000109906 | 2.38e-18 | 1.02e-16 |
| ENSG00000109911 | 2.55e-01 | 3.31e-01 |
| ENSG00000109917 | 3.70e-21 | 3.45e-19 |
| ENSG00000109919 | 9.81e-01 | 9.86e-01 |
| ENSG00000109920 | 2.44e-09 | 1.81e-08 |
| ENSG00000109927 | 1.53e-02 | 2.96e-02 |
| ENSG00000109929 | 4.17e-01 | 5.00e-01 |
| ENSG00000109943 | 4.17e-07 | 2.10e-06 |
| ENSG00000109944 | 5.04e-06 | 2.10e-05 |
| ENSG00000109956 | 9.49e-03 | 1.95e-02 |
| ENSG00000109971 | 1.88e-10 | 1.68e-09 |
| ENSG00000109991 | 3.30e-01 | 4.11e-01 |
| ENSG00000110002 | 1.22e-15 | 2.87e-14 |
| ENSG00000110011 | 7.67e-06 | 3.10e-05 |
| ENSG00000110013 | 2.80e-17 | 9.36e-16 |
| ENSG00000110025 | 2.73e-01 | 3.52e-01 |
| ENSG00000110031 | 4.22e-03 | 9.54e-03 |
| ENSG00000110042 | 1.67e-06 | 7.55e-06 |
| ENSG00000110046 | 3.08e-01 | 3.88e-01 |
| ENSG00000110047 | 1.46e-04 | 4.61e-04 |
| ENSG00000110048 | 9.24e-01 | 9.43e-01 |
| ENSG00000110057 | 3.39e-04 | 9.93e-04 |
| ENSG00000110060 | 1.75e-06 | 7.87e-06 |
| ENSG00000110063 | 1.45e-08 | 9.45e-08 |
| ENSG00000110066 | 3.74e-05 | 1.33e-04 |
| ENSG00000110074 | 3.85e-04 | 1.11e-03 |
| ENSG00000110075 | 3.98e-01 | 4.82e-01 |
| ENSG00000110076 | 7.01e-07 | 3.41e-06 |
| ENSG00000110077 | 4.31e-09 | 3.06e-08 |
| ENSG00000110079 | 3.23e-09 | 2.34e-08 |
| ENSG00000110080 | 4.29e-05 | 1.51e-04 |
| ENSG00000110090 | 5.82e-17 | 1.82e-15 |
| ENSG00000110092 | 7.92e-26 | 3.69e-23 |
| ENSG00000110104 | 5.99e-18 | 2.32e-16 |
| ENSG00000110107 | 2.72e-10 | 2.37e-09 |
| ENSG00000110108 | 2.01e-06 | 8.96e-06 |
| ENSG00000110148 | 2.64e-02 | 4.76e-02 |
| ENSG00000110169 | 5.01e-01 | 5.81e-01 |
| ENSG00000110171 | 5.13e-05 | 1.77e-04 |
| ENSG00000110172 | 1.20e-07 | 6.70e-07 |
| ENSG00000110195 | 7.82e-08 | 4.49e-07 |
| ENSG00000110200 | 1.22e-02 | 2.42e-02 |
| ENSG00000110203 | 3.85e-01 | 4.68e-01 |
| ENSG00000110218 | 2.92e-09 | 2.13e-08 |
| ENSG00000110237 | 6.99e-01 | 7.61e-01 |
| ENSG00000110243 | 8.35e-01 | 8.75e-01 |
| ENSG00000110244 | 2.47e-01 | 3.23e-01 |
| ENSG00000110245 | 2.41e-01 | 3.17e-01 |
| ENSG00000110274 | 1.31e-13 | 2.08e-12 |
| ENSG00000110315 | 7.01e-01 | 7.63e-01 |
| ENSG00000110318 | 3.61e-03 | 8.31e-03 |
| ENSG00000110321 | 7.27e-01 | 7.85e-01 |
| ENSG00000110324 | 1.10e-11 | 1.22e-10 |
| ENSG00000110328 | 8.08e-03 | 1.69e-02 |
| ENSG00000110330 | 9.63e-05 | 3.14e-04 |
| ENSG00000110344 | 1.62e-02 | 3.11e-02 |
| ENSG00000110367 | 9.54e-02 | 1.44e-01 |
| ENSG00000110375 | 2.27e-09 | 1.69e-08 |
| ENSG00000110395 | 5.25e-02 | 8.65e-02 |
| ENSG00000110400 | 3.45e-08 | 2.10e-07 |
| ENSG00000110422 | 1.03e-03 | 2.72e-03 |
| ENSG00000110427 | 3.61e-06 | 1.54e-05 |
| ENSG00000110429 | 1.37e-05 | 5.28e-05 |
| ENSG00000110435 | 5.36e-01 | 6.15e-01 |
| ENSG00000110436 | 3.10e-04 | 9.14e-04 |
| ENSG00000110442 | 4.47e-02 | 7.52e-02 |
| ENSG00000110446 | 4.37e-01 | 5.19e-01 |
| ENSG00000110448 | 8.92e-01 | 9.20e-01 |
| ENSG00000110455 | 1.23e-02 | 2.44e-02 |
| ENSG00000110484 | 5.16e-01 | 5.96e-01 |
| ENSG00000110492 | 6.06e-04 | 1.68e-03 |
| ENSG00000110497 | 3.00e-01 | 3.80e-01 |
| ENSG00000110514 | 2.98e-01 | 3.78e-01 |
| ENSG00000110536 | 1.27e-05 | 4.92e-05 |
| ENSG00000110583 | 1.10e-01 | 1.64e-01 |
| ENSG00000110619 | 1.98e-05 | 7.40e-05 |
| ENSG00000110628 | 1.42e-04 | 4.50e-04 |
| ENSG00000110651 | 5.23e-05 | 1.80e-04 |
| ENSG00000110660 | 7.43e-02 | 1.17e-01 |
| ENSG00000110665 | 3.30e-07 | 1.70e-06 |
| ENSG00000110675 | 4.54e-06 | 1.90e-05 |
| ENSG00000110680 | 3.39e-08 | 2.07e-07 |
| ENSG00000110693 | 2.86e-05 | 1.04e-04 |
| ENSG00000110696 | 3.22e-06 | 1.39e-05 |
| ENSG00000110697 | 1.08e-01 | 1.61e-01 |
| ENSG00000110700 | 6.10e-06 | 2.50e-05 |
| ENSG00000110711 | 2.88e-01 | 3.68e-01 |
| ENSG00000110713 | 2.37e-08 | 1.49e-07 |
| ENSG00000110717 | 3.08e-01 | 3.89e-01 |
| ENSG00000110719 | 3.85e-02 | 6.61e-02 |
| ENSG00000110721 | 2.06e-05 | 7.68e-05 |
| ENSG00000110723 | 2.38e-02 | 4.34e-02 |
| ENSG00000110756 | 5.68e-01 | 6.45e-01 |
| ENSG00000110768 | 1.43e-03 | 3.64e-03 |
| ENSG00000110777 | 9.98e-11 | 9.34e-10 |
| ENSG00000110786 | 1.06e-01 | 1.58e-01 |
| ENSG00000110799 | 5.94e-01 | 6.68e-01 |
| ENSG00000110801 | 4.99e-01 | 5.79e-01 |
| ENSG00000110811 | 3.48e-03 | 8.06e-03 |
| ENSG00000110841 | 8.14e-05 | 2.70e-04 |
| ENSG00000110844 | 8.92e-10 | 7.11e-09 |
| ENSG00000110848 | 9.11e-08 | 5.18e-07 |
| ENSG00000110851 | 1.18e-01 | 1.73e-01 |
| ENSG00000110852 | 7.34e-06 | 2.97e-05 |
| ENSG00000110871 | 1.55e-03 | 3.93e-03 |
| ENSG00000110876 | 6.57e-04 | 1.81e-03 |
| ENSG00000110880 | 2.56e-01 | 3.32e-01 |
| ENSG00000110881 | 6.13e-16 | 1.54e-14 |
| ENSG00000110887 | 9.91e-27 | 6.59e-24 |
| ENSG00000110888 | 7.76e-03 | 1.63e-02 |
| ENSG00000110900 | 3.75e-11 | 3.78e-10 |
| ENSG00000110906 | 7.69e-04 | 2.09e-03 |
| ENSG00000110911 | 6.20e-18 | 2.39e-16 |
| ENSG00000110917 | 6.34e-03 | 1.36e-02 |
| ENSG00000110921 | 1.86e-03 | 4.60e-03 |
| ENSG00000110925 | 4.77e-06 | 2.00e-05 |
| ENSG00000110931 | 1.41e-02 | 2.74e-02 |
| ENSG00000110934 | 1.01e-10 | 9.42e-10 |
| ENSG00000110944 | 4.06e-18 | 1.63e-16 |
| ENSG00000110955 | 3.37e-08 | 2.06e-07 |
| ENSG00000110958 | 4.14e-05 | 1.46e-04 |
| ENSG00000110975 | 6.82e-18 | 2.61e-16 |
| ENSG00000110987 | 1.06e-10 | 9.89e-10 |
| ENSG00000111011 | 3.60e-01 | 4.43e-01 |
| ENSG00000111012 | 7.67e-23 | 1.21e-20 |
| ENSG00000111046 | 1.32e-01 | 1.90e-01 |
| ENSG00000111052 | 8.32e-01 | 8.72e-01 |
| ENSG00000111057 | 1.07e-05 | 4.21e-05 |
| ENSG00000111058 | 3.95e-04 | 1.14e-03 |
| ENSG00000111077 | 1.45e-10 | 1.32e-09 |
| ENSG00000111087 | 2.95e-01 | 3.76e-01 |
| ENSG00000111110 | 4.72e-25 | 1.53e-22 |
| ENSG00000111142 | 7.15e-07 | 3.47e-06 |
| ENSG00000111144 | 4.16e-03 | 9.42e-03 |
| ENSG00000111145 | 2.56e-02 | 4.65e-02 |
| ENSG00000111181 | 3.78e-03 | 8.66e-03 |
| ENSG00000111186 | 5.10e-06 | 2.12e-05 |
| ENSG00000111196 | 6.79e-14 | 1.13e-12 |
| ENSG00000111199 | 4.58e-13 | 6.60e-12 |
| ENSG00000111203 | 1.19e-03 | 3.09e-03 |
| ENSG00000111206 | 1.38e-15 | 3.20e-14 |
| ENSG00000111215 | 1.55e-04 | 4.85e-04 |
| ENSG00000111218 | 1.40e-03 | 3.58e-03 |
| ENSG00000111224 | 2.90e-01 | 3.69e-01 |
| ENSG00000111229 | 1.27e-01 | 1.85e-01 |
| ENSG00000111231 | 1.39e-10 | 1.27e-09 |
| ENSG00000111237 | 4.19e-01 | 5.02e-01 |
| ENSG00000111241 | 7.00e-01 | 7.62e-01 |
| ENSG00000111245 | 3.39e-05 | 1.22e-04 |
| ENSG00000111247 | 5.10e-12 | 6.02e-11 |
| ENSG00000111249 | 4.39e-14 | 7.61e-13 |
| ENSG00000111252 | 8.31e-05 | 2.75e-04 |
| ENSG00000111254 | 1.26e-04 | 4.04e-04 |
| ENSG00000111261 | 5.08e-02 | 8.41e-02 |
| ENSG00000111262 | 2.63e-09 | 1.93e-08 |
| ENSG00000111266 | 7.72e-02 | 1.20e-01 |
| ENSG00000111269 | 8.45e-06 | 3.39e-05 |
| ENSG00000111271 | 9.78e-05 | 3.19e-04 |
| ENSG00000111275 | 2.67e-06 | 1.16e-05 |
| ENSG00000111276 | 1.19e-01 | 1.74e-01 |
| ENSG00000111291 | 1.75e-02 | 3.33e-02 |
| ENSG00000111300 | 2.10e-12 | 2.68e-11 |
| ENSG00000111305 | 7.07e-03 | 1.50e-02 |
| ENSG00000111319 | 8.33e-05 | 2.76e-04 |
| ENSG00000111321 | 2.48e-04 | 7.48e-04 |
| ENSG00000111325 | 5.30e-02 | 8.72e-02 |
| ENSG00000111328 | 3.90e-07 | 1.98e-06 |
| ENSG00000111331 | 1.09e-08 | 7.21e-08 |
| ENSG00000111335 | 2.38e-03 | 5.74e-03 |
| ENSG00000111339 | 8.56e-07 | 4.10e-06 |
| ENSG00000111341 | 3.79e-10 | 3.22e-09 |
| ENSG00000111344 | 2.07e-02 | 3.85e-02 |
| ENSG00000111348 | 3.59e-01 | 4.42e-01 |
| ENSG00000111358 | 4.21e-06 | 1.78e-05 |
| ENSG00000111361 | 2.88e-04 | 8.56e-04 |
| ENSG00000111364 | 2.36e-20 | 1.71e-18 |
| ENSG00000111371 | 5.27e-01 | 6.06e-01 |
| ENSG00000111404 | 1.95e-22 | 2.63e-20 |
| ENSG00000111405 | 1.06e-04 | 3.43e-04 |
| ENSG00000111412 | 2.90e-01 | 3.70e-01 |
| ENSG00000111424 | 1.33e-09 | 1.03e-08 |
| ENSG00000111432 | 4.55e-02 | 7.64e-02 |
| ENSG00000111445 | 3.22e-10 | 2.77e-09 |
| ENSG00000111450 | 4.72e-05 | 1.64e-04 |
| ENSG00000111452 | 2.07e-07 | 1.11e-06 |
| ENSG00000111481 | 6.23e-10 | 5.09e-09 |
| ENSG00000111490 | 4.57e-18 | 1.83e-16 |
| ENSG00000111530 | 3.89e-03 | 8.88e-03 |
| ENSG00000111536 | 8.56e-01 | 8.91e-01 |
| ENSG00000111537 | 7.58e-04 | 2.06e-03 |
| ENSG00000111540 | 7.98e-11 | 7.61e-10 |
| ENSG00000111554 | 8.57e-03 | 1.78e-02 |
| ENSG00000111581 | 4.37e-16 | 1.13e-14 |
| ENSG00000111596 | 1.27e-02 | 2.51e-02 |
| ENSG00000111602 | 9.39e-15 | 1.85e-13 |
| ENSG00000111605 | 6.07e-12 | 7.06e-11 |
| ENSG00000111615 | 8.88e-04 | 2.37e-03 |
| ENSG00000111639 | 1.62e-02 | 3.11e-02 |
| ENSG00000111640 | 1.41e-07 | 7.73e-07 |
| ENSG00000111641 | 3.26e-22 | 4.22e-20 |
| ENSG00000111642 | 2.56e-06 | 1.12e-05 |
| ENSG00000111644 | 1.63e-01 | 2.27e-01 |
| ENSG00000111647 | 3.80e-06 | 1.61e-05 |
| ENSG00000111652 | 2.85e-05 | 1.04e-04 |
| ENSG00000111653 | 6.17e-05 | 2.09e-04 |
| ENSG00000111664 | 4.22e-02 | 7.16e-02 |
| ENSG00000111665 | 1.33e-20 | 1.04e-18 |
| ENSG00000111666 | 4.24e-05 | 1.49e-04 |
| ENSG00000111667 | 2.83e-05 | 1.03e-04 |
| ENSG00000111669 | 1.15e-02 | 2.31e-02 |
| ENSG00000111670 | 2.70e-11 | 2.79e-10 |
| ENSG00000111671 | 2.18e-03 | 5.30e-03 |
| ENSG00000111674 | 2.10e-01 | 2.82e-01 |
| ENSG00000111676 | 8.62e-01 | 8.96e-01 |
| ENSG00000111678 | 2.70e-02 | 4.85e-02 |
| ENSG00000111679 | 6.00e-01 | 6.73e-01 |
| ENSG00000111684 | 1.03e-10 | 9.62e-10 |
| ENSG00000111696 | 4.61e-01 | 5.43e-01 |
| ENSG00000111700 | 9.24e-14 | 1.51e-12 |
| ENSG00000111701 | 3.70e-03 | 8.50e-03 |
| ENSG00000111704 | 3.46e-01 | 4.29e-01 |
| ENSG00000111707 | 9.05e-01 | 9.29e-01 |
| ENSG00000111711 | 2.18e-04 | 6.65e-04 |
| ENSG00000111713 | 1.34e-01 | 1.92e-01 |
| ENSG00000111716 | 1.26e-04 | 4.03e-04 |
| ENSG00000111725 | 3.23e-01 | 4.05e-01 |
| ENSG00000111726 | 4.47e-06 | 1.88e-05 |
| ENSG00000111727 | 4.17e-11 | 4.18e-10 |
| ENSG00000111728 | 9.18e-11 | 8.66e-10 |
| ENSG00000111729 | 9.58e-01 | 9.69e-01 |
| ENSG00000111731 | 7.79e-03 | 1.64e-02 |
| ENSG00000111732 | 4.46e-10 | 3.74e-09 |
| ENSG00000111737 | 4.04e-01 | 4.87e-01 |
| ENSG00000111752 | 7.74e-01 | 8.24e-01 |
| ENSG00000111775 | 2.01e-04 | 6.17e-04 |
| ENSG00000111780 | 8.55e-01 | 8.91e-01 |
| ENSG00000111783 | 1.59e-03 | 4.01e-03 |
| ENSG00000111785 | 1.99e-02 | 3.72e-02 |
| ENSG00000111786 | 6.06e-06 | 2.48e-05 |
| ENSG00000111788 | 1.82e-10 | 1.63e-09 |
| ENSG00000111790 | 1.56e-01 | 2.19e-01 |
| ENSG00000111796 | 7.07e-16 | 1.74e-14 |
| ENSG00000111799 | 9.84e-14 | 1.60e-12 |
| ENSG00000111801 | 2.12e-01 | 2.84e-01 |
| ENSG00000111802 | 2.80e-21 | 2.72e-19 |
| ENSG00000111816 | 5.21e-03 | 1.15e-02 |
| ENSG00000111817 | 2.42e-01 | 3.18e-01 |
| ENSG00000111832 | 3.29e-02 | 5.76e-02 |
| ENSG00000111834 | 5.03e-01 | 5.83e-01 |
| ENSG00000111837 | 1.36e-04 | 4.31e-04 |
| ENSG00000111843 | 4.84e-01 | 5.66e-01 |
| ENSG00000111845 | 2.10e-14 | 3.89e-13 |
| ENSG00000111846 | 1.46e-28 | 1.74e-25 |
| ENSG00000111850 | 7.01e-04 | 1.92e-03 |
| ENSG00000111859 | 8.78e-09 | 5.91e-08 |
| ENSG00000111860 | 2.38e-08 | 1.49e-07 |
| ENSG00000111863 | 1.06e-19 | 6.37e-18 |
| ENSG00000111875 | 5.91e-01 | 6.65e-01 |
| ENSG00000111877 | 7.49e-01 | 8.04e-01 |
| ENSG00000111879 | 1.71e-07 | 9.26e-07 |
| ENSG00000111880 | 1.03e-02 | 2.10e-02 |
| ENSG00000111885 | 4.47e-12 | 5.33e-11 |
| ENSG00000111886 | 7.88e-01 | 8.36e-01 |
| ENSG00000111897 | 1.02e-12 | 1.37e-11 |
| ENSG00000111906 | 2.59e-01 | 3.36e-01 |
| ENSG00000111907 | 1.93e-10 | 1.72e-09 |
| ENSG00000111911 | 1.56e-01 | 2.19e-01 |
| ENSG00000111912 | 7.31e-09 | 4.99e-08 |
| ENSG00000111913 | 1.99e-06 | 8.87e-06 |
| ENSG00000111961 | 7.28e-07 | 3.53e-06 |
| ENSG00000111962 | 5.47e-11 | 5.36e-10 |
| ENSG00000111981 | 1.16e-18 | 5.37e-17 |
| ENSG00000112029 | 1.59e-09 | 1.22e-08 |
| ENSG00000112031 | 3.97e-03 | 9.05e-03 |
| ENSG00000112033 | 4.34e-10 | 3.65e-09 |
| ENSG00000112038 | 1.49e-03 | 3.79e-03 |
| ENSG00000112039 | 1.85e-08 | 1.18e-07 |
| ENSG00000112041 | 2.28e-01 | 3.01e-01 |
| ENSG00000112053 | 2.33e-01 | 3.08e-01 |
| ENSG00000112062 | 1.15e-02 | 2.30e-02 |
| ENSG00000112077 | 3.12e-12 | 3.84e-11 |
| ENSG00000112078 | 1.24e-07 | 6.89e-07 |
| ENSG00000112079 | 1.88e-02 | 3.55e-02 |
| ENSG00000112081 | 7.91e-05 | 2.63e-04 |
| ENSG00000112096 | 6.62e-02 | 1.05e-01 |
| ENSG00000112110 | 1.76e-03 | 4.38e-03 |
| ENSG00000112115 | 6.63e-06 | 2.70e-05 |
| ENSG00000112116 | 1.19e-05 | 4.65e-05 |
| ENSG00000112118 | 3.93e-19 | 2.06e-17 |
| ENSG00000112130 | 1.84e-15 | 4.15e-14 |
| ENSG00000112137 | 9.38e-03 | 1.93e-02 |
| ENSG00000112139 | 4.91e-09 | 3.45e-08 |
| ENSG00000112144 | 3.38e-01 | 4.20e-01 |
| ENSG00000112146 | 4.37e-01 | 5.20e-01 |
| ENSG00000112149 | 1.18e-01 | 1.74e-01 |
| ENSG00000112159 | 1.67e-03 | 4.19e-03 |
| ENSG00000112164 | 3.70e-02 | 6.38e-02 |
| ENSG00000112167 | 1.26e-02 | 2.50e-02 |
| ENSG00000112175 | 1.37e-16 | 3.96e-15 |
| ENSG00000112182 | 9.17e-07 | 4.38e-06 |
| ENSG00000112183 | 2.72e-15 | 5.96e-14 |
| ENSG00000112186 | 4.00e-08 | 2.41e-07 |
| ENSG00000112195 | 9.07e-01 | 9.31e-01 |
| ENSG00000112200 | 5.58e-03 | 1.22e-02 |
| ENSG00000112208 | 4.28e-01 | 5.12e-01 |
| ENSG00000112210 | 6.19e-01 | 6.90e-01 |
| ENSG00000112212 | 7.87e-08 | 4.51e-07 |
| ENSG00000112214 | 3.36e-08 | 2.05e-07 |
| ENSG00000112218 | 3.99e-01 | 4.82e-01 |
| ENSG00000112232 | 4.32e-20 | 2.89e-18 |
| ENSG00000112234 | 7.07e-03 | 1.50e-02 |
| ENSG00000112237 | 2.00e-03 | 4.91e-03 |
| ENSG00000112238 | 5.95e-08 | 3.50e-07 |
| ENSG00000112242 | 9.75e-17 | 2.91e-15 |
| ENSG00000112245 | 3.49e-11 | 3.54e-10 |
| ENSG00000112246 | 6.85e-02 | 1.09e-01 |
| ENSG00000112249 | 3.55e-11 | 3.59e-10 |
| ENSG00000112276 | 2.93e-09 | 2.14e-08 |
| ENSG00000112280 | 1.15e-03 | 3.00e-03 |
| ENSG00000112282 | 1.95e-01 | 2.65e-01 |
| ENSG00000112290 | 4.32e-01 | 5.15e-01 |
| ENSG00000112293 | 2.99e-06 | 1.29e-05 |
| ENSG00000112294 | 2.63e-01 | 3.40e-01 |
| ENSG00000112297 | 1.66e-05 | 6.28e-05 |
| ENSG00000112299 | 8.60e-02 | 1.32e-01 |
| ENSG00000112303 | 1.29e-04 | 4.11e-04 |
| ENSG00000112304 | 1.06e-08 | 7.06e-08 |
| ENSG00000112305 | 4.08e-09 | 2.91e-08 |
| ENSG00000112306 | 1.58e-03 | 3.99e-03 |
| ENSG00000112308 | 3.58e-01 | 4.40e-01 |
| ENSG00000112309 | 2.08e-01 | 2.79e-01 |
| ENSG00000112312 | 6.68e-01 | 7.34e-01 |
| ENSG00000112319 | 2.88e-01 | 3.68e-01 |
| ENSG00000112320 | 1.33e-03 | 3.42e-03 |
| ENSG00000112333 | 2.42e-01 | 3.18e-01 |
| ENSG00000112335 | 5.74e-02 | 9.33e-02 |
| ENSG00000112337 | 5.82e-01 | 6.57e-01 |
| ENSG00000112339 | 1.26e-03 | 3.25e-03 |
| ENSG00000112343 | 2.43e-02 | 4.42e-02 |
| ENSG00000112357 | 3.22e-04 | 9.46e-04 |
| ENSG00000112365 | 1.74e-04 | 5.41e-04 |
| ENSG00000112367 | 7.26e-07 | 3.52e-06 |
| ENSG00000112378 | 2.65e-16 | 7.24e-15 |
| ENSG00000112379 | 2.59e-08 | 1.61e-07 |
| ENSG00000112394 | 2.97e-01 | 3.78e-01 |
| ENSG00000112406 | 2.38e-04 | 7.20e-04 |
| ENSG00000112414 | 6.39e-02 | 1.02e-01 |
| ENSG00000112419 | 8.14e-02 | 1.26e-01 |
| ENSG00000112425 | 2.65e-07 | 1.39e-06 |
| ENSG00000112473 | 1.15e-01 | 1.69e-01 |
| ENSG00000112486 | 2.74e-02 | 4.93e-02 |
| ENSG00000112494 | 2.87e-15 | 6.26e-14 |
| ENSG00000112499 | 2.87e-05 | 1.04e-04 |
| ENSG00000112511 | 1.55e-05 | 5.91e-05 |
| ENSG00000112514 | 8.68e-04 | 2.33e-03 |
| ENSG00000112530 | 2.53e-04 | 7.61e-04 |
| ENSG00000112531 | 1.22e-04 | 3.91e-04 |
| ENSG00000112539 | 4.26e-01 | 5.09e-01 |
| ENSG00000112541 | 2.62e-06 | 1.15e-05 |
| ENSG00000112559 | 1.21e-26 | 7.73e-24 |
| ENSG00000112561 | 2.46e-06 | 1.08e-05 |
| ENSG00000112562 | 3.97e-01 | 4.80e-01 |
| ENSG00000112576 | 1.02e-03 | 2.70e-03 |
| ENSG00000112578 | 2.40e-24 | 6.14e-22 |
| ENSG00000112584 | 1.22e-05 | 4.74e-05 |
| ENSG00000112592 | 5.20e-07 | 2.58e-06 |
| ENSG00000112599 | 5.10e-01 | 5.90e-01 |
| ENSG00000112619 | 2.47e-06 | 1.08e-05 |
| ENSG00000112624 | 1.61e-02 | 3.10e-02 |
| ENSG00000112640 | 8.99e-01 | 9.25e-01 |
| ENSG00000112651 | 1.21e-02 | 2.40e-02 |
| ENSG00000112655 | 4.98e-15 | 1.03e-13 |
| ENSG00000112658 | 4.05e-01 | 4.88e-01 |
| ENSG00000112659 | 5.25e-02 | 8.64e-02 |
| ENSG00000112667 | 5.11e-05 | 1.76e-04 |
| ENSG00000112679 | 1.71e-01 | 2.38e-01 |
| ENSG00000112685 | 2.29e-04 | 6.96e-04 |
| ENSG00000112695 | 4.52e-05 | 1.58e-04 |
| ENSG00000112697 | 4.48e-02 | 7.54e-02 |
| ENSG00000112699 | 1.22e-03 | 3.16e-03 |
| ENSG00000112701 | 6.65e-01 | 7.31e-01 |
| ENSG00000112706 | 9.28e-05 | 3.04e-04 |
| ENSG00000112715 | 1.32e-19 | 7.74e-18 |
| ENSG00000112739 | 4.78e-03 | 1.07e-02 |
| ENSG00000112742 | 1.74e-10 | 1.57e-09 |
| ENSG00000112759 | 4.11e-15 | 8.70e-14 |
| ENSG00000112761 | 9.57e-07 | 4.54e-06 |
| ENSG00000112763 | 5.40e-02 | 8.86e-02 |
| ENSG00000112769 | 6.42e-01 | 7.11e-01 |
| ENSG00000112773 | 2.33e-12 | 2.95e-11 |
| ENSG00000112782 | 7.02e-16 | 1.73e-14 |
| ENSG00000112787 | 1.59e-10 | 1.44e-09 |
| ENSG00000112796 | 3.21e-03 | 7.49e-03 |
| ENSG00000112799 | 1.37e-07 | 7.54e-07 |
| ENSG00000112812 | 6.85e-02 | 1.09e-01 |
| ENSG00000112818 | 1.66e-13 | 2.58e-12 |
| ENSG00000112837 | 3.26e-02 | 5.72e-02 |
| ENSG00000112851 | 5.97e-07 | 2.93e-06 |
| ENSG00000112852 | 2.75e-04 | 8.19e-04 |
| ENSG00000112855 | 1.18e-01 | 1.73e-01 |
| ENSG00000112874 | 3.50e-08 | 2.13e-07 |
| ENSG00000112877 | 3.73e-23 | 6.30e-21 |
| ENSG00000112893 | 2.06e-05 | 7.69e-05 |
| ENSG00000112902 | 1.09e-02 | 2.19e-02 |
| ENSG00000112936 | 1.45e-20 | 1.12e-18 |
| ENSG00000112941 | 2.78e-14 | 5.00e-13 |
| ENSG00000112964 | 2.42e-18 | 1.03e-16 |
| ENSG00000112972 | 4.12e-01 | 4.95e-01 |
| ENSG00000112977 | 9.38e-05 | 3.07e-04 |
| ENSG00000112981 | 2.31e-05 | 8.52e-05 |
| ENSG00000112983 | 2.79e-06 | 1.22e-05 |
| ENSG00000112984 | 2.79e-17 | 9.32e-16 |
| ENSG00000112992 | 9.04e-07 | 4.32e-06 |
| ENSG00000112996 | 1.60e-08 | 1.03e-07 |
| ENSG00000113013 | 5.27e-06 | 2.18e-05 |
| ENSG00000113048 | 6.00e-06 | 2.46e-05 |
| ENSG00000113068 | 2.29e-02 | 4.21e-02 |
| ENSG00000113070 | 4.98e-01 | 5.79e-01 |
| ENSG00000113073 | 7.11e-02 | 1.12e-01 |
| ENSG00000113083 | 6.36e-06 | 2.60e-05 |
| ENSG00000113088 | 2.99e-07 | 1.55e-06 |
| ENSG00000113100 | 3.96e-02 | 6.77e-02 |
| ENSG00000113108 | 2.30e-02 | 4.23e-02 |
| ENSG00000113119 | 1.23e-08 | 8.05e-08 |
| ENSG00000113140 | 5.51e-14 | 9.36e-13 |
| ENSG00000113141 | 1.06e-02 | 2.14e-02 |
| ENSG00000113161 | 3.96e-03 | 9.03e-03 |
| ENSG00000113163 | 2.20e-06 | 9.74e-06 |
| ENSG00000113194 | 5.48e-03 | 1.20e-02 |
| ENSG00000113196 | 2.64e-05 | 9.65e-05 |
| ENSG00000113205 | 4.34e-04 | 1.24e-03 |
| ENSG00000113209 | 1.61e-06 | 7.31e-06 |
| ENSG00000113211 | 4.89e-04 | 1.38e-03 |
| ENSG00000113212 | 4.73e-02 | 7.89e-02 |
| ENSG00000113231 | 1.94e-01 | 2.64e-01 |
| ENSG00000113240 | 4.78e-01 | 5.61e-01 |
| ENSG00000113248 | 2.05e-01 | 2.76e-01 |
| ENSG00000113249 | 8.39e-05 | 2.77e-04 |
| ENSG00000113262 | 3.90e-02 | 6.69e-02 |
| ENSG00000113263 | 3.18e-06 | 1.37e-05 |
| ENSG00000113269 | 7.08e-01 | 7.69e-01 |
| ENSG00000113272 | 4.52e-02 | 7.59e-02 |
| ENSG00000113273 | 9.27e-01 | 9.46e-01 |
| ENSG00000113282 | 3.83e-04 | 1.11e-03 |
| ENSG00000113296 | 1.61e-03 | 4.05e-03 |
| ENSG00000113300 | 3.02e-02 | 5.36e-02 |
| ENSG00000113302 | 7.49e-01 | 8.04e-01 |
| ENSG00000113303 | 3.03e-19 | 1.62e-17 |
| ENSG00000113312 | 3.62e-01 | 4.45e-01 |
| ENSG00000113318 | 9.02e-01 | 9.27e-01 |
| ENSG00000113319 | 7.07e-03 | 1.50e-02 |
| ENSG00000113327 | 4.73e-17 | 1.51e-15 |
| ENSG00000113328 | 8.80e-04 | 2.35e-03 |
| ENSG00000113356 | 3.36e-12 | 4.11e-11 |
| ENSG00000113360 | 3.21e-06 | 1.38e-05 |
| ENSG00000113361 | 9.03e-10 | 7.19e-09 |
| ENSG00000113368 | 3.73e-07 | 1.90e-06 |
| ENSG00000113369 | 4.38e-01 | 5.21e-01 |
| ENSG00000113384 | 1.55e-01 | 2.18e-01 |
| ENSG00000113387 | 3.03e-01 | 3.83e-01 |
| ENSG00000113389 | 1.31e-04 | 4.19e-04 |
| ENSG00000113391 | 6.61e-03 | 1.42e-02 |
| ENSG00000113396 | 2.11e-22 | 2.80e-20 |
| ENSG00000113407 | 1.49e-07 | 8.16e-07 |
| ENSG00000113430 | 3.44e-02 | 5.99e-02 |
| ENSG00000113441 | 1.43e-01 | 2.04e-01 |
| ENSG00000113448 | 4.75e-09 | 3.35e-08 |
| ENSG00000113456 | 2.37e-13 | 3.60e-12 |
| ENSG00000113460 | 1.21e-12 | 1.61e-11 |
| ENSG00000113492 | 1.99e-02 | 3.73e-02 |
| ENSG00000113494 | 5.32e-01 | 6.11e-01 |
| ENSG00000113504 | 2.45e-03 | 5.89e-03 |
| ENSG00000113520 | 5.49e-01 | 6.27e-01 |
| ENSG00000113522 | 9.43e-05 | 3.09e-04 |
| ENSG00000113525 | 8.71e-04 | 2.33e-03 |
| ENSG00000113532 | 1.35e-06 | 6.23e-06 |
| ENSG00000113552 | 1.03e-17 | 3.79e-16 |
| ENSG00000113555 | 1.58e-04 | 4.94e-04 |
| ENSG00000113558 | 1.03e-02 | 2.10e-02 |
| ENSG00000113569 | 1.38e-15 | 3.20e-14 |
| ENSG00000113575 | 6.44e-01 | 7.13e-01 |
| ENSG00000113578 | 2.57e-01 | 3.34e-01 |
| ENSG00000113580 | 1.79e-18 | 7.85e-17 |
| ENSG00000113583 | 1.77e-01 | 2.44e-01 |
| ENSG00000113593 | 1.42e-04 | 4.48e-04 |
| ENSG00000113594 | 1.72e-24 | 4.64e-22 |
| ENSG00000113595 | 9.40e-03 | 1.93e-02 |
| ENSG00000113597 | 9.64e-01 | 9.73e-01 |
| ENSG00000113600 | 4.41e-01 | 5.24e-01 |
| ENSG00000113615 | 2.89e-01 | 3.69e-01 |
| ENSG00000113621 | 1.08e-03 | 2.84e-03 |
| ENSG00000113638 | 6.05e-05 | 2.06e-04 |
| ENSG00000113643 | 5.32e-06 | 2.21e-05 |
| ENSG00000113645 | 1.50e-07 | 8.20e-07 |
| ENSG00000113648 | 3.05e-11 | 3.12e-10 |
| ENSG00000113649 | 2.43e-10 | 2.14e-09 |
| ENSG00000113657 | 8.97e-02 | 1.37e-01 |
| ENSG00000113658 | 3.40e-04 | 9.95e-04 |
| ENSG00000113712 | 1.83e-01 | 2.51e-01 |
| ENSG00000113716 | 3.67e-12 | 4.46e-11 |
| ENSG00000113719 | 1.15e-02 | 2.30e-02 |
| ENSG00000113721 | 4.67e-10 | 3.90e-09 |
| ENSG00000113722 | 6.76e-04 | 1.86e-03 |
| ENSG00000113732 | 3.21e-01 | 4.03e-01 |
| ENSG00000113734 | 1.22e-01 | 1.79e-01 |
| ENSG00000113739 | 6.02e-21 | 5.17e-19 |
| ENSG00000113742 | 2.11e-06 | 9.36e-06 |
| ENSG00000113749 | 6.16e-01 | 6.88e-01 |
| ENSG00000113758 | 1.24e-02 | 2.47e-02 |
| ENSG00000113761 | 4.47e-05 | 1.56e-04 |
| ENSG00000113763 | 1.62e-05 | 6.17e-05 |
| ENSG00000113790 | 2.15e-12 | 2.73e-11 |
| ENSG00000113805 | 4.15e-17 | 1.34e-15 |
| ENSG00000113810 | 3.28e-05 | 1.18e-04 |
| ENSG00000113811 | 2.44e-02 | 4.44e-02 |
| ENSG00000113812 | 1.10e-03 | 2.87e-03 |
| ENSG00000113838 | 8.99e-04 | 2.40e-03 |
| ENSG00000113845 | 1.65e-01 | 2.30e-01 |
| ENSG00000113851 | 1.83e-03 | 4.54e-03 |
| ENSG00000113889 | 2.17e-09 | 1.62e-08 |
| ENSG00000113905 | 1.08e-01 | 1.61e-01 |
| ENSG00000113916 | 5.17e-01 | 5.97e-01 |
| ENSG00000113924 | 1.78e-10 | 1.60e-09 |
| ENSG00000113946 | 3.00e-18 | 1.26e-16 |
| ENSG00000113966 | 1.05e-01 | 1.57e-01 |
| ENSG00000113971 | 5.34e-02 | 8.77e-02 |
| ENSG00000114013 | 7.39e-02 | 1.16e-01 |
| ENSG00000114019 | 1.41e-10 | 1.29e-09 |
| ENSG00000114021 | 8.23e-18 | 3.10e-16 |
| ENSG00000114023 | 1.17e-18 | 5.40e-17 |
| ENSG00000114026 | 5.43e-03 | 1.19e-02 |
| ENSG00000114030 | 2.55e-02 | 4.62e-02 |
| ENSG00000114054 | 1.19e-01 | 1.75e-01 |
| ENSG00000114062 | 3.58e-01 | 4.41e-01 |
| ENSG00000114098 | 6.86e-05 | 2.31e-04 |
| ENSG00000114107 | 1.62e-09 | 1.24e-08 |
| ENSG00000114113 | 4.21e-02 | 7.14e-02 |
| ENSG00000114115 | 1.56e-02 | 3.00e-02 |
| ENSG00000114120 | 5.84e-03 | 1.27e-02 |
| ENSG00000114124 | 2.97e-03 | 6.98e-03 |
| ENSG00000114125 | 2.72e-06 | 1.19e-05 |
| ENSG00000114126 | 9.85e-01 | 9.89e-01 |
| ENSG00000114127 | 8.51e-03 | 1.77e-02 |
| ENSG00000114166 | 1.46e-16 | 4.19e-15 |
| ENSG00000114200 | 1.48e-22 | 2.06e-20 |
| ENSG00000114204 | 3.93e-02 | 6.73e-02 |
| ENSG00000114209 | 5.97e-02 | 9.65e-02 |
| ENSG00000114248 | 4.36e-09 | 3.09e-08 |
| ENSG00000114251 | 5.44e-15 | 1.12e-13 |
| ENSG00000114268 | 1.04e-06 | 4.92e-06 |
| ENSG00000114270 | 1.72e-20 | 1.29e-18 |
| ENSG00000114279 | 1.96e-04 | 6.02e-04 |
| ENSG00000114302 | 1.54e-01 | 2.17e-01 |
| ENSG00000114315 | 1.70e-01 | 2.36e-01 |
| ENSG00000114316 | 5.58e-01 | 6.35e-01 |
| ENSG00000114331 | 9.73e-05 | 3.17e-04 |
| ENSG00000114346 | 4.36e-18 | 1.75e-16 |
| ENSG00000114349 | 6.61e-03 | 1.42e-02 |
| ENSG00000114353 | 1.79e-01 | 2.47e-01 |
| ENSG00000114354 | 5.37e-04 | 1.51e-03 |
| ENSG00000114374 | 2.12e-02 | 3.94e-02 |
| ENSG00000114378 | 2.00e-01 | 2.71e-01 |
| ENSG00000114383 | 4.88e-01 | 5.70e-01 |
| ENSG00000114388 | 3.56e-04 | 1.04e-03 |
| ENSG00000114391 | 1.39e-02 | 2.71e-02 |
| ENSG00000114395 | 9.81e-01 | 9.86e-01 |
| ENSG00000114405 | 1.15e-01 | 1.70e-01 |
| ENSG00000114416 | 9.54e-08 | 5.39e-07 |
| ENSG00000114423 | 2.60e-01 | 3.38e-01 |
| ENSG00000114439 | 9.78e-01 | 9.84e-01 |
| ENSG00000114446 | 4.33e-05 | 1.52e-04 |
| ENSG00000114450 | 2.48e-02 | 4.52e-02 |
| ENSG00000114455 | 6.26e-20 | 3.96e-18 |
| ENSG00000114473 | 2.59e-01 | 3.36e-01 |
| ENSG00000114480 | 6.19e-01 | 6.90e-01 |
| ENSG00000114487 | 3.55e-08 | 2.16e-07 |
| ENSG00000114491 | 7.11e-15 | 1.43e-13 |
| ENSG00000114503 | 3.02e-14 | 5.38e-13 |
| ENSG00000114520 | 9.45e-07 | 4.49e-06 |
| ENSG00000114529 | 2.67e-01 | 3.45e-01 |
| ENSG00000114541 | 9.80e-11 | 9.19e-10 |
| ENSG00000114544 | 7.66e-10 | 6.17e-09 |
| ENSG00000114547 | 7.69e-02 | 1.20e-01 |
| ENSG00000114554 | 6.24e-22 | 7.49e-20 |
| ENSG00000114573 | 2.17e-01 | 2.89e-01 |
| ENSG00000114626 | 3.36e-01 | 4.18e-01 |
| ENSG00000114631 | 2.49e-10 | 2.18e-09 |
| ENSG00000114638 | 4.30e-01 | 5.13e-01 |
| ENSG00000114646 | 8.06e-01 | 8.51e-01 |
| ENSG00000114648 | 3.67e-09 | 2.64e-08 |
| ENSG00000114650 | 9.94e-01 | 9.95e-01 |
| ENSG00000114654 | 3.09e-02 | 5.46e-02 |
| ENSG00000114656 | 1.34e-27 | 1.17e-24 |
| ENSG00000114670 | 9.69e-02 | 1.46e-01 |
| ENSG00000114686 | 1.27e-15 | 2.96e-14 |
| ENSG00000114698 | 3.55e-16 | 9.39e-15 |
| ENSG00000114735 | 9.35e-04 | 2.49e-03 |
| ENSG00000114737 | 1.95e-02 | 3.67e-02 |
| ENSG00000114738 | 1.71e-08 | 1.10e-07 |
| ENSG00000114739 | 1.66e-10 | 1.50e-09 |
| ENSG00000114742 | 8.77e-01 | 9.08e-01 |
| ENSG00000114744 | 3.25e-02 | 5.71e-02 |
| ENSG00000114745 | 1.17e-02 | 2.34e-02 |
| ENSG00000114757 | 2.68e-02 | 4.83e-02 |
| ENSG00000114767 | 7.57e-17 | 2.30e-15 |
| ENSG00000114770 | 9.60e-01 | 9.70e-01 |
| ENSG00000114771 | 6.06e-09 | 4.20e-08 |
| ENSG00000114779 | 2.83e-02 | 5.06e-02 |
| ENSG00000114784 | 2.99e-03 | 7.04e-03 |
| ENSG00000114786 | 1.56e-06 | 7.10e-06 |
| ENSG00000114790 | 5.43e-06 | 2.25e-05 |
| ENSG00000114796 | 6.75e-01 | 7.40e-01 |
| ENSG00000114805 | 3.30e-02 | 5.79e-02 |
| ENSG00000114812 | 5.85e-12 | 6.84e-11 |
| ENSG00000114841 | 1.32e-01 | 1.90e-01 |
| ENSG00000114850 | 7.25e-03 | 1.54e-02 |
| ENSG00000114853 | 3.62e-03 | 8.34e-03 |
| ENSG00000114854 | 7.52e-02 | 1.18e-01 |
| ENSG00000114857 | 1.07e-02 | 2.17e-02 |
| ENSG00000114859 | 5.22e-12 | 6.14e-11 |
| ENSG00000114861 | 2.67e-02 | 4.81e-02 |
| ENSG00000114867 | 1.04e-05 | 4.11e-05 |
| ENSG00000114902 | 9.74e-01 | 9.81e-01 |
| ENSG00000114904 | 4.43e-04 | 1.26e-03 |
| ENSG00000114923 | 4.47e-01 | 5.30e-01 |
| ENSG00000114933 | 5.42e-01 | 6.20e-01 |
| ENSG00000114942 | 1.83e-04 | 5.67e-04 |
| ENSG00000114948 | 2.19e-04 | 6.67e-04 |
| ENSG00000114956 | 3.04e-04 | 8.98e-04 |
| ENSG00000114978 | 1.02e-01 | 1.53e-01 |
| ENSG00000114982 | 5.50e-03 | 1.20e-02 |
| ENSG00000114988 | 4.68e-02 | 7.83e-02 |
| ENSG00000114993 | 4.55e-20 | 3.01e-18 |
| ENSG00000114999 | 2.73e-10 | 2.37e-09 |
| ENSG00000115008 | 2.41e-10 | 2.12e-09 |
| ENSG00000115009 | 1.17e-04 | 3.78e-04 |
| ENSG00000115020 | 8.15e-01 | 8.59e-01 |
| ENSG00000115041 | 4.10e-09 | 2.93e-08 |
| ENSG00000115042 | 1.57e-01 | 2.20e-01 |
| ENSG00000115053 | 4.20e-14 | 7.30e-13 |
| ENSG00000115073 | 1.57e-01 | 2.20e-01 |
| ENSG00000115084 | 9.98e-03 | 2.04e-02 |
| ENSG00000115085 | 3.72e-03 | 8.55e-03 |
| ENSG00000115091 | 1.23e-01 | 1.79e-01 |
| ENSG00000115107 | 1.69e-01 | 2.35e-01 |
| ENSG00000115109 | 2.41e-03 | 5.81e-03 |
| ENSG00000115112 | 3.98e-06 | 1.69e-05 |
| ENSG00000115128 | 3.03e-04 | 8.94e-04 |
| ENSG00000115129 | 2.73e-16 | 7.42e-15 |
| ENSG00000115137 | 1.50e-03 | 3.80e-03 |
| ENSG00000115138 | 1.24e-05 | 4.81e-05 |
| ENSG00000115145 | 1.38e-06 | 6.36e-06 |
| ENSG00000115155 | 7.76e-01 | 8.26e-01 |
| ENSG00000115159 | 1.27e-04 | 4.07e-04 |
| ENSG00000115163 | 4.63e-16 | 1.19e-14 |
| ENSG00000115165 | 6.00e-05 | 2.04e-04 |
| ENSG00000115170 | 2.58e-08 | 1.61e-07 |
| ENSG00000115183 | 5.94e-01 | 6.68e-01 |
| ENSG00000115194 | 3.85e-01 | 4.68e-01 |
| ENSG00000115204 | 2.72e-15 | 5.94e-14 |
| ENSG00000115207 | 1.76e-03 | 4.39e-03 |
| ENSG00000115211 | 1.25e-05 | 4.86e-05 |
| ENSG00000115216 | 9.21e-01 | 9.41e-01 |
| ENSG00000115221 | 1.02e-01 | 1.53e-01 |
| ENSG00000115226 | 3.65e-02 | 6.31e-02 |
| ENSG00000115232 | 8.28e-06 | 3.33e-05 |
| ENSG00000115233 | 9.89e-17 | 2.94e-15 |
| ENSG00000115234 | 9.96e-02 | 1.50e-01 |
| ENSG00000115239 | 1.21e-02 | 2.42e-02 |
| ENSG00000115241 | 1.18e-11 | 1.30e-10 |
| ENSG00000115252 | 2.95e-09 | 2.15e-08 |
| ENSG00000115255 | 2.60e-10 | 2.27e-09 |
| ENSG00000115257 | 2.47e-06 | 1.09e-05 |
| ENSG00000115263 | 8.12e-21 | 6.87e-19 |
| ENSG00000115266 | 1.90e-04 | 5.87e-04 |
| ENSG00000115267 | 4.87e-01 | 5.69e-01 |
| ENSG00000115268 | 2.91e-09 | 2.12e-08 |
| ENSG00000115271 | 1.39e-02 | 2.72e-02 |
| ENSG00000115274 | 2.09e-06 | 9.29e-06 |
| ENSG00000115275 | 3.55e-09 | 2.55e-08 |
| ENSG00000115282 | 9.88e-01 | 9.92e-01 |
| ENSG00000115286 | 2.68e-02 | 4.83e-02 |
| ENSG00000115289 | 4.72e-08 | 2.82e-07 |
| ENSG00000115290 | 3.23e-02 | 5.68e-02 |
| ENSG00000115295 | 1.97e-13 | 3.02e-12 |
| ENSG00000115297 | 3.89e-10 | 3.30e-09 |
| ENSG00000115306 | 5.52e-01 | 6.30e-01 |
| ENSG00000115307 | 9.62e-02 | 1.45e-01 |
| ENSG00000115310 | 2.52e-06 | 1.10e-05 |
| ENSG00000115317 | 1.99e-01 | 2.70e-01 |
| ENSG00000115318 | 4.95e-02 | 8.22e-02 |
| ENSG00000115325 | 5.26e-02 | 8.66e-02 |
| ENSG00000115339 | 1.48e-01 | 2.09e-01 |
| ENSG00000115350 | 4.89e-01 | 5.71e-01 |
| ENSG00000115353 | 4.60e-09 | 3.25e-08 |
| ENSG00000115355 | 3.52e-03 | 8.14e-03 |
| ENSG00000115361 | 6.01e-09 | 4.17e-08 |
| ENSG00000115363 | 9.30e-22 | 1.05e-19 |
| ENSG00000115364 | 2.27e-03 | 5.51e-03 |
| ENSG00000115365 | 6.79e-01 | 7.43e-01 |
| ENSG00000115368 | 1.52e-18 | 6.82e-17 |
| ENSG00000115380 | 9.44e-08 | 5.34e-07 |
| ENSG00000115386 | 2.15e-09 | 1.61e-08 |
| ENSG00000115392 | 5.88e-10 | 4.83e-09 |
| ENSG00000115414 | 9.85e-04 | 2.61e-03 |
| ENSG00000115415 | 5.82e-04 | 1.62e-03 |
| ENSG00000115419 | 1.52e-02 | 2.94e-02 |
| ENSG00000115421 | 6.35e-02 | 1.02e-01 |
| ENSG00000115423 | 5.68e-05 | 1.94e-04 |
| ENSG00000115425 | 5.01e-11 | 4.95e-10 |
| ENSG00000115446 | 3.85e-02 | 6.61e-02 |
| ENSG00000115457 | 5.46e-03 | 1.20e-02 |
| ENSG00000115459 | 7.61e-01 | 8.14e-01 |
| ENSG00000115461 | 1.93e-01 | 2.63e-01 |
| ENSG00000115464 | 4.82e-02 | 8.03e-02 |
| ENSG00000115468 | 2.88e-09 | 2.10e-08 |
| ENSG00000115474 | 8.36e-01 | 8.76e-01 |
| ENSG00000115484 | 4.86e-14 | 8.35e-13 |
| ENSG00000115486 | 6.12e-03 | 1.32e-02 |
| ENSG00000115488 | 5.32e-02 | 8.75e-02 |
| ENSG00000115504 | 2.51e-01 | 3.27e-01 |
| ENSG00000115507 | 7.53e-25 | 2.28e-22 |
| ENSG00000115514 | 4.68e-01 | 5.50e-01 |
| ENSG00000115520 | 2.52e-04 | 7.57e-04 |
| ENSG00000115523 | 8.42e-01 | 8.80e-01 |
| ENSG00000115524 | 1.61e-04 | 5.04e-04 |
| ENSG00000115525 | 8.75e-05 | 2.88e-04 |
| ENSG00000115526 | 3.00e-02 | 5.32e-02 |
| ENSG00000115539 | 8.29e-03 | 1.73e-02 |
| ENSG00000115540 | 1.61e-02 | 3.08e-02 |
| ENSG00000115541 | 1.65e-12 | 2.15e-11 |
| ENSG00000115548 | 1.53e-05 | 5.84e-05 |
| ENSG00000115556 | 2.65e-07 | 1.39e-06 |
| ENSG00000115561 | 3.16e-07 | 1.63e-06 |
| ENSG00000115568 | 3.48e-11 | 3.53e-10 |
| ENSG00000115590 | 6.80e-17 | 2.09e-15 |
| ENSG00000115592 | 8.80e-01 | 9.10e-01 |
| ENSG00000115593 | 1.35e-03 | 3.46e-03 |
| ENSG00000115594 | 2.88e-06 | 1.25e-05 |
| ENSG00000115596 | 7.68e-05 | 2.56e-04 |
| ENSG00000115598 | 5.31e-04 | 1.49e-03 |
| ENSG00000115602 | 6.08e-03 | 1.32e-02 |
| ENSG00000115604 | 1.93e-04 | 5.95e-04 |
| ENSG00000115607 | 4.65e-06 | 1.95e-05 |
| ENSG00000115616 | 8.01e-19 | 3.88e-17 |
| ENSG00000115641 | 4.91e-07 | 2.45e-06 |
| ENSG00000115648 | 3.78e-01 | 4.61e-01 |
| ENSG00000115649 | 1.74e-07 | 9.38e-07 |
| ENSG00000115652 | 1.53e-11 | 1.65e-10 |
| ENSG00000115657 | 8.96e-12 | 1.01e-10 |
| ENSG00000115661 | 6.05e-01 | 6.78e-01 |
| ENSG00000115665 | 3.60e-16 | 9.50e-15 |
| ENSG00000115677 | 2.88e-04 | 8.55e-04 |
| ENSG00000115685 | 9.27e-01 | 9.45e-01 |
| ENSG00000115687 | 1.39e-13 | 2.19e-12 |
| ENSG00000115694 | 2.31e-06 | 1.02e-05 |
| ENSG00000115705 | 1.26e-07 | 6.98e-07 |
| ENSG00000115718 | 1.74e-01 | 2.41e-01 |
| ENSG00000115738 | 9.95e-10 | 7.87e-09 |
| ENSG00000115750 | 2.66e-06 | 1.16e-05 |
| ENSG00000115756 | 4.03e-04 | 1.16e-03 |
| ENSG00000115758 | 1.86e-14 | 3.48e-13 |
| ENSG00000115760 | 9.44e-01 | 9.59e-01 |
| ENSG00000115761 | 5.92e-10 | 4.86e-09 |
| ENSG00000115762 | 1.94e-08 | 1.23e-07 |
| ENSG00000115806 | 1.05e-11 | 1.17e-10 |
| ENSG00000115808 | 6.81e-01 | 7.45e-01 |
| ENSG00000115816 | 1.20e-03 | 3.12e-03 |
| ENSG00000115825 | 5.02e-01 | 5.82e-01 |
| ENSG00000115827 | 4.10e-04 | 1.18e-03 |
| ENSG00000115828 | 9.22e-03 | 1.90e-02 |
| ENSG00000115839 | 5.69e-03 | 1.24e-02 |
| ENSG00000115840 | 5.41e-08 | 3.20e-07 |
| ENSG00000115841 | 4.12e-21 | 3.75e-19 |
| ENSG00000115844 | 1.60e-01 | 2.24e-01 |
| ENSG00000115850 | 8.43e-02 | 1.30e-01 |
| ENSG00000115866 | 3.99e-18 | 1.61e-16 |
| ENSG00000115875 | 7.76e-13 | 1.07e-11 |
| ENSG00000115884 | 3.62e-01 | 4.45e-01 |
| ENSG00000115896 | 2.09e-06 | 9.29e-06 |
| ENSG00000115902 | 2.14e-10 | 1.90e-09 |
| ENSG00000115904 | 2.79e-02 | 5.00e-02 |
| ENSG00000115919 | 2.75e-03 | 6.54e-03 |
| ENSG00000115935 | 2.16e-05 | 8.02e-05 |
| ENSG00000115942 | 1.47e-06 | 6.73e-06 |
| ENSG00000115944 | 7.74e-01 | 8.24e-01 |
| ENSG00000115946 | 2.74e-20 | 1.95e-18 |
| ENSG00000115947 | 2.93e-02 | 5.21e-02 |
| ENSG00000115956 | 5.46e-02 | 8.94e-02 |
| ENSG00000115963 | 4.04e-04 | 1.16e-03 |
| ENSG00000115966 | 7.15e-01 | 7.75e-01 |
| ENSG00000115970 | 8.09e-09 | 5.47e-08 |
| ENSG00000115977 | 1.08e-02 | 2.18e-02 |
| ENSG00000115993 | 1.17e-17 | 4.22e-16 |
| ENSG00000115998 | 3.60e-01 | 4.43e-01 |
| ENSG00000116001 | 1.49e-07 | 8.13e-07 |
| ENSG00000116005 | 3.34e-05 | 1.20e-04 |
| ENSG00000116014 | 2.07e-05 | 7.71e-05 |
| ENSG00000116016 | 1.19e-12 | 1.58e-11 |
| ENSG00000116017 | 3.84e-18 | 1.56e-16 |
| ENSG00000116030 | 3.77e-01 | 4.60e-01 |
| ENSG00000116031 | 2.15e-10 | 1.90e-09 |
| ENSG00000116032 | 5.36e-06 | 2.22e-05 |
| ENSG00000116035 | 7.31e-01 | 7.88e-01 |
| ENSG00000116039 | 2.25e-05 | 8.34e-05 |
| ENSG00000116044 | 4.93e-03 | 1.09e-02 |
| ENSG00000116062 | 2.45e-10 | 2.15e-09 |
| ENSG00000116095 | 3.31e-01 | 4.12e-01 |
| ENSG00000116096 | 7.12e-01 | 7.72e-01 |
| ENSG00000116106 | 6.88e-09 | 4.71e-08 |
| ENSG00000116117 | 7.49e-05 | 2.50e-04 |
| ENSG00000116120 | 3.22e-12 | 3.95e-11 |
| ENSG00000116127 | 1.98e-01 | 2.69e-01 |
| ENSG00000116128 | 3.01e-06 | 1.30e-05 |
| ENSG00000116132 | 3.68e-08 | 2.23e-07 |
| ENSG00000116133 | 2.79e-01 | 3.58e-01 |
| ENSG00000116138 | 4.84e-01 | 5.66e-01 |
| ENSG00000116141 | 5.17e-04 | 1.46e-03 |
| ENSG00000116147 | 3.30e-07 | 1.70e-06 |
| ENSG00000116151 | 1.77e-02 | 3.36e-02 |
| ENSG00000116157 | 2.00e-03 | 4.92e-03 |
| ENSG00000116161 | 6.36e-09 | 4.39e-08 |
| ENSG00000116171 | 4.92e-16 | 1.26e-14 |
| ENSG00000116176 | 2.32e-11 | 2.42e-10 |
| ENSG00000116183 | 1.15e-08 | 7.60e-08 |
| ENSG00000116191 | 9.75e-04 | 2.58e-03 |
| ENSG00000116194 | 7.42e-18 | 2.82e-16 |
| ENSG00000116198 | 2.03e-02 | 3.79e-02 |
| ENSG00000116199 | 3.69e-02 | 6.38e-02 |
| ENSG00000116205 | 9.17e-01 | 9.38e-01 |
| ENSG00000116209 | 4.49e-15 | 9.41e-14 |
| ENSG00000116212 | 1.73e-04 | 5.38e-04 |
| ENSG00000116213 | 7.59e-11 | 7.27e-10 |
| ENSG00000116218 | 6.22e-04 | 1.73e-03 |
| ENSG00000116221 | 7.41e-08 | 4.27e-07 |
| ENSG00000116237 | 8.91e-01 | 9.18e-01 |
| ENSG00000116251 | 2.38e-01 | 3.14e-01 |
| ENSG00000116254 | 2.71e-05 | 9.91e-05 |
| ENSG00000116260 | 4.55e-02 | 7.64e-02 |
| ENSG00000116266 | 4.67e-04 | 1.33e-03 |
| ENSG00000116273 | 6.61e-04 | 1.82e-03 |
| ENSG00000116285 | 6.65e-01 | 7.31e-01 |
| ENSG00000116288 | 4.50e-03 | 1.01e-02 |
| ENSG00000116299 | 8.89e-02 | 1.36e-01 |
| ENSG00000116329 | 1.97e-02 | 3.70e-02 |
| ENSG00000116337 | 2.95e-19 | 1.58e-17 |
| ENSG00000116350 | 4.90e-01 | 5.71e-01 |
| ENSG00000116353 | 6.03e-06 | 2.48e-05 |
| ENSG00000116396 | 1.46e-04 | 4.59e-04 |
| ENSG00000116406 | 5.06e-04 | 1.43e-03 |
| ENSG00000116455 | 4.51e-23 | 7.50e-21 |
| ENSG00000116459 | 2.15e-11 | 2.26e-10 |
| ENSG00000116473 | 3.17e-15 | 6.87e-14 |
| ENSG00000116478 | 4.81e-01 | 5.63e-01 |
| ENSG00000116489 | 8.29e-01 | 8.70e-01 |
| ENSG00000116497 | 5.92e-03 | 1.29e-02 |
| ENSG00000116514 | 2.15e-05 | 8.00e-05 |
| ENSG00000116521 | 1.77e-06 | 7.95e-06 |
| ENSG00000116525 | 1.77e-01 | 2.44e-01 |
| ENSG00000116539 | 2.24e-02 | 4.12e-02 |
| ENSG00000116544 | 7.00e-06 | 2.84e-05 |
| ENSG00000116560 | 1.79e-11 | 1.91e-10 |
| ENSG00000116574 | 6.77e-18 | 2.60e-16 |
| ENSG00000116580 | 4.62e-02 | 7.74e-02 |
| ENSG00000116584 | 2.83e-02 | 5.05e-02 |
| ENSG00000116586 | 1.18e-01 | 1.73e-01 |
| ENSG00000116604 | 3.84e-10 | 3.26e-09 |
| ENSG00000116641 | 8.14e-01 | 8.57e-01 |
| ENSG00000116649 | 2.95e-16 | 7.95e-15 |
| ENSG00000116652 | 2.72e-01 | 3.50e-01 |
| ENSG00000116661 | 2.32e-03 | 5.62e-03 |
| ENSG00000116663 | 1.32e-02 | 2.59e-02 |
| ENSG00000116667 | 1.15e-11 | 1.26e-10 |
| ENSG00000116668 | 1.44e-02 | 2.81e-02 |
| ENSG00000116670 | 1.93e-14 | 3.60e-13 |
| ENSG00000116675 | 2.71e-01 | 3.49e-01 |
| ENSG00000116678 | 4.91e-11 | 4.86e-10 |
| ENSG00000116679 | 1.43e-02 | 2.78e-02 |
| ENSG00000116685 | 1.27e-02 | 2.51e-02 |
| ENSG00000116688 | 5.92e-13 | 8.32e-12 |
| ENSG00000116690 | 8.52e-01 | 8.89e-01 |
| ENSG00000116691 | 3.03e-09 | 2.20e-08 |
| ENSG00000116698 | 4.09e-05 | 1.44e-04 |
| ENSG00000116701 | 2.73e-01 | 3.51e-01 |
| ENSG00000116703 | 1.62e-01 | 2.26e-01 |
| ENSG00000116704 | 2.39e-15 | 5.28e-14 |
| ENSG00000116711 | 9.45e-03 | 1.94e-02 |
| ENSG00000116717 | 8.17e-01 | 8.61e-01 |
| ENSG00000116729 | 5.79e-15 | 1.18e-13 |
| ENSG00000116731 | 1.63e-01 | 2.27e-01 |
| ENSG00000116741 | 2.84e-10 | 2.47e-09 |
| ENSG00000116745 | 8.54e-01 | 8.90e-01 |
| ENSG00000116747 | 3.28e-01 | 4.10e-01 |
| ENSG00000116748 | 1.24e-19 | 7.34e-18 |
| ENSG00000116750 | 1.28e-04 | 4.09e-04 |
| ENSG00000116752 | 4.21e-02 | 7.15e-02 |
| ENSG00000116754 | 7.87e-05 | 2.62e-04 |
| ENSG00000116761 | 3.69e-05 | 1.31e-04 |
| ENSG00000116771 | 2.07e-04 | 6.32e-04 |
| ENSG00000116774 | 9.65e-08 | 5.45e-07 |
| ENSG00000116783 | 2.42e-04 | 7.30e-04 |
| ENSG00000116785 | 9.36e-02 | 1.42e-01 |
| ENSG00000116786 | 6.46e-01 | 7.15e-01 |
| ENSG00000116791 | 1.73e-02 | 3.29e-02 |
| ENSG00000116793 | 1.72e-03 | 4.30e-03 |
| ENSG00000116809 | 8.07e-01 | 8.52e-01 |
| ENSG00000116815 | 5.77e-03 | 1.26e-02 |
| ENSG00000116819 | 5.74e-01 | 6.50e-01 |
| ENSG00000116824 | 6.08e-06 | 2.49e-05 |
| ENSG00000116830 | 4.86e-11 | 4.81e-10 |
| ENSG00000116833 | 7.11e-20 | 4.45e-18 |
| ENSG00000116852 | 2.45e-10 | 2.15e-09 |
| ENSG00000116857 | 1.76e-12 | 2.28e-11 |
| ENSG00000116863 | 2.00e-01 | 2.70e-01 |
| ENSG00000116871 | 5.86e-03 | 1.27e-02 |
| ENSG00000116874 | 1.43e-03 | 3.65e-03 |
| ENSG00000116882 | 2.01e-01 | 2.71e-01 |
| ENSG00000116883 | 5.35e-07 | 2.65e-06 |
| ENSG00000116885 | 3.83e-02 | 6.59e-02 |
| ENSG00000116898 | 1.57e-02 | 3.02e-02 |
| ENSG00000116903 | 5.34e-01 | 6.13e-01 |
| ENSG00000116906 | 8.16e-02 | 1.26e-01 |
| ENSG00000116918 | 6.45e-01 | 7.14e-01 |
| ENSG00000116922 | 9.25e-22 | 1.05e-19 |
| ENSG00000116954 | 2.74e-08 | 1.70e-07 |
| ENSG00000116957 | 2.29e-08 | 1.44e-07 |
| ENSG00000116962 | 2.64e-01 | 3.42e-01 |
| ENSG00000116977 | 2.13e-01 | 2.86e-01 |
| ENSG00000116981 | 2.04e-01 | 2.75e-01 |
| ENSG00000116983 | 1.16e-11 | 1.28e-10 |
| ENSG00000116984 | 2.50e-05 | 9.18e-05 |
| ENSG00000116985 | 6.22e-07 | 3.04e-06 |
| ENSG00000116990 | 2.57e-01 | 3.34e-01 |
| ENSG00000116991 | 1.91e-18 | 8.37e-17 |
| ENSG00000117000 | 8.46e-01 | 8.84e-01 |
| ENSG00000117009 | 1.08e-02 | 2.18e-02 |
| ENSG00000117010 | 7.57e-13 | 1.05e-11 |
| ENSG00000117013 | 1.02e-03 | 2.70e-03 |
| ENSG00000117016 | 7.67e-11 | 7.34e-10 |
| ENSG00000117020 | 5.37e-04 | 1.51e-03 |
| ENSG00000117036 | 3.21e-01 | 4.02e-01 |
| ENSG00000117054 | 1.44e-16 | 4.13e-15 |
| ENSG00000117069 | 8.51e-04 | 2.28e-03 |
| ENSG00000117090 | 2.16e-09 | 1.61e-08 |
| ENSG00000117091 | 2.42e-12 | 3.05e-11 |
| ENSG00000117114 | 1.81e-07 | 9.76e-07 |
| ENSG00000117115 | 8.14e-22 | 9.47e-20 |
| ENSG00000117118 | 2.32e-09 | 1.72e-08 |
| ENSG00000117122 | 1.48e-18 | 6.65e-17 |
| ENSG00000117133 | 9.53e-01 | 9.65e-01 |
| ENSG00000117139 | 2.54e-01 | 3.30e-01 |
| ENSG00000117143 | 3.90e-10 | 3.30e-09 |
| ENSG00000117148 | 2.17e-01 | 2.90e-01 |
| ENSG00000117151 | 8.27e-05 | 2.74e-04 |
| ENSG00000117152 | 6.73e-01 | 7.38e-01 |
| ENSG00000117153 | 2.03e-02 | 3.80e-02 |
| ENSG00000117154 | 9.34e-01 | 9.51e-01 |
| ENSG00000117155 | 9.74e-07 | 4.62e-06 |
| ENSG00000117174 | 6.72e-10 | 5.45e-09 |
| ENSG00000117215 | 3.73e-07 | 1.90e-06 |
| ENSG00000117222 | 1.39e-02 | 2.72e-02 |
| ENSG00000117226 | 1.32e-10 | 1.21e-09 |
| ENSG00000117228 | 1.38e-01 | 1.98e-01 |
| ENSG00000117242 | 4.34e-01 | 5.17e-01 |
| ENSG00000117245 | 7.91e-05 | 2.63e-04 |
| ENSG00000117262 | 2.10e-14 | 3.89e-13 |
| ENSG00000117266 | 7.41e-03 | 1.57e-02 |
| ENSG00000117280 | 2.31e-04 | 7.01e-04 |
| ENSG00000117281 | 1.61e-12 | 2.10e-11 |
| ENSG00000117298 | 1.67e-10 | 1.51e-09 |
| ENSG00000117305 | 4.02e-15 | 8.52e-14 |
| ENSG00000117308 | 9.59e-01 | 9.70e-01 |
| ENSG00000117318 | 8.48e-07 | 4.07e-06 |
| ENSG00000117322 | 1.32e-09 | 1.02e-08 |
| ENSG00000117335 | 6.75e-14 | 1.13e-12 |
| ENSG00000117360 | 4.15e-12 | 4.98e-11 |
| ENSG00000117362 | 1.87e-01 | 2.56e-01 |
| ENSG00000117385 | 8.75e-17 | 2.63e-15 |
| ENSG00000117394 | 5.64e-14 | 9.57e-13 |
| ENSG00000117395 | 2.83e-10 | 2.46e-09 |
| ENSG00000117399 | 2.72e-10 | 2.37e-09 |
| ENSG00000117400 | 2.29e-03 | 5.56e-03 |
| ENSG00000117407 | 7.43e-02 | 1.17e-01 |
| ENSG00000117408 | 4.62e-01 | 5.45e-01 |
| ENSG00000117410 | 9.84e-02 | 1.48e-01 |
| ENSG00000117411 | 2.28e-07 | 1.21e-06 |
| ENSG00000117419 | 2.15e-05 | 7.99e-05 |
| ENSG00000117425 | 4.93e-02 | 8.20e-02 |
| ENSG00000117448 | 1.90e-01 | 2.60e-01 |
| ENSG00000117450 | 7.86e-04 | 2.13e-03 |
| ENSG00000117461 | 1.37e-02 | 2.68e-02 |
| ENSG00000117472 | 6.00e-17 | 1.87e-15 |
| ENSG00000117475 | 2.21e-01 | 2.94e-01 |
| ENSG00000117477 | 2.27e-05 | 8.41e-05 |
| ENSG00000117479 | 1.30e-16 | 3.79e-15 |
| ENSG00000117480 | 9.53e-01 | 9.65e-01 |
| ENSG00000117481 | 2.25e-11 | 2.36e-10 |
| ENSG00000117500 | 1.81e-02 | 3.42e-02 |
| ENSG00000117501 | 6.66e-06 | 2.71e-05 |
| ENSG00000117505 | 6.67e-01 | 7.33e-01 |
| ENSG00000117507 | 6.54e-03 | 1.40e-02 |
| ENSG00000117519 | 8.25e-01 | 8.67e-01 |
| ENSG00000117523 | 5.53e-04 | 1.55e-03 |
| ENSG00000117525 | 2.50e-01 | 3.26e-01 |
| ENSG00000117528 | 3.07e-16 | 8.24e-15 |
| ENSG00000117533 | 7.69e-02 | 1.20e-01 |
| ENSG00000117543 | 5.82e-05 | 1.99e-04 |
| ENSG00000117560 | 1.10e-04 | 3.56e-04 |
| ENSG00000117569 | 1.94e-03 | 4.78e-03 |
| ENSG00000117586 | 2.55e-09 | 1.88e-08 |
| ENSG00000117592 | 7.46e-24 | 1.68e-21 |
| ENSG00000117593 | 4.06e-02 | 6.91e-02 |
| ENSG00000117594 | 6.21e-01 | 6.92e-01 |
| ENSG00000117595 | 6.91e-03 | 1.47e-02 |
| ENSG00000117597 | 4.00e-19 | 2.08e-17 |
| ENSG00000117598 | 7.58e-01 | 8.11e-01 |
| ENSG00000117600 | 1.82e-03 | 4.52e-03 |
| ENSG00000117601 | 7.24e-01 | 7.82e-01 |
| ENSG00000117602 | 9.02e-03 | 1.86e-02 |
| ENSG00000117614 | 4.97e-05 | 1.72e-04 |
| ENSG00000117616 | 1.97e-03 | 4.86e-03 |
| ENSG00000117620 | 3.10e-09 | 2.25e-08 |
| ENSG00000117625 | 7.80e-01 | 8.30e-01 |
| ENSG00000117632 | 2.72e-07 | 1.42e-06 |
| ENSG00000117640 | 4.50e-11 | 4.48e-10 |
| ENSG00000117643 | 7.95e-09 | 5.39e-08 |
| ENSG00000117650 | 6.92e-17 | 2.12e-15 |
| ENSG00000117676 | 4.13e-10 | 3.49e-09 |
| ENSG00000117682 | 8.10e-19 | 3.92e-17 |
| ENSG00000117691 | 5.05e-05 | 1.75e-04 |
| ENSG00000117697 | 1.83e-01 | 2.52e-01 |
| ENSG00000117707 | 3.59e-10 | 3.06e-09 |
| ENSG00000117713 | 9.01e-01 | 9.26e-01 |
| ENSG00000117724 | 6.00e-10 | 4.91e-09 |
| ENSG00000117748 | 2.71e-04 | 8.08e-04 |
| ENSG00000117751 | 1.09e-02 | 2.20e-02 |
| ENSG00000117758 | 3.48e-13 | 5.11e-12 |
| ENSG00000117791 | 3.49e-16 | 9.25e-15 |
| ENSG00000117834 | 1.89e-01 | 2.59e-01 |
| ENSG00000117859 | 2.87e-02 | 5.12e-02 |
| ENSG00000117862 | 1.14e-05 | 4.46e-05 |
| ENSG00000117868 | 1.63e-03 | 4.09e-03 |
| ENSG00000117877 | 1.95e-24 | 5.16e-22 |
| ENSG00000117899 | 8.94e-03 | 1.85e-02 |
| ENSG00000117906 | 3.10e-07 | 1.60e-06 |
| ENSG00000117971 | 3.10e-04 | 9.14e-04 |
| ENSG00000117983 | 6.19e-02 | 9.96e-02 |
| ENSG00000117984 | 3.12e-07 | 1.61e-06 |
| ENSG00000118004 | 2.11e-03 | 5.16e-03 |
| ENSG00000118007 | 9.20e-03 | 1.90e-02 |
| ENSG00000118017 | 2.89e-01 | 3.69e-01 |
| ENSG00000118046 | 1.30e-02 | 2.57e-02 |
| ENSG00000118058 | 8.58e-01 | 8.93e-01 |
| ENSG00000118094 | 4.10e-06 | 1.73e-05 |
| ENSG00000118096 | 3.51e-02 | 6.11e-02 |
| ENSG00000118113 | 2.42e-16 | 6.65e-15 |
| ENSG00000118137 | 4.10e-01 | 4.93e-01 |
| ENSG00000118156 | 3.25e-01 | 4.06e-01 |
| ENSG00000118160 | 1.13e-04 | 3.64e-04 |
| ENSG00000118162 | 7.87e-02 | 1.22e-01 |
| ENSG00000118181 | 4.10e-01 | 4.93e-01 |
| ENSG00000118193 | 7.77e-15 | 1.55e-13 |
| ENSG00000118194 | 2.90e-11 | 2.99e-10 |
| ENSG00000118197 | 7.59e-01 | 8.12e-01 |
| ENSG00000118200 | 1.03e-02 | 2.10e-02 |
| ENSG00000118217 | 1.44e-02 | 2.79e-02 |
| ENSG00000118242 | 6.74e-04 | 1.85e-03 |
| ENSG00000118246 | 2.39e-10 | 2.10e-09 |
| ENSG00000118257 | 3.48e-01 | 4.31e-01 |
| ENSG00000118260 | 3.42e-01 | 4.24e-01 |
| ENSG00000118263 | 4.59e-07 | 2.30e-06 |
| ENSG00000118271 | 3.95e-07 | 2.00e-06 |
| ENSG00000118276 | 1.23e-02 | 2.45e-02 |
| ENSG00000118292 | 4.71e-03 | 1.05e-02 |
| ENSG00000118298 | 2.93e-09 | 2.14e-08 |
| ENSG00000118307 | 1.07e-01 | 1.60e-01 |
| ENSG00000118308 | 1.21e-16 | 3.52e-15 |
| ENSG00000118322 | 3.26e-03 | 7.60e-03 |
| ENSG00000118363 | 5.08e-01 | 5.88e-01 |
| ENSG00000118369 | 2.94e-08 | 1.81e-07 |
| ENSG00000118402 | 6.50e-10 | 5.29e-09 |
| ENSG00000118407 | 2.95e-09 | 2.15e-08 |
| ENSG00000118412 | 8.15e-01 | 8.58e-01 |
| ENSG00000118418 | 5.43e-02 | 8.90e-02 |
| ENSG00000118420 | 2.56e-17 | 8.65e-16 |
| ENSG00000118432 | 2.65e-18 | 1.12e-16 |
| ENSG00000118454 | 8.87e-05 | 2.92e-04 |
| ENSG00000118473 | 4.83e-08 | 2.88e-07 |
| ENSG00000118482 | 6.20e-02 | 9.98e-02 |
| ENSG00000118491 | 1.16e-01 | 1.71e-01 |
| ENSG00000118492 | 2.90e-05 | 1.05e-04 |
| ENSG00000118495 | 9.10e-02 | 1.39e-01 |
| ENSG00000118496 | 4.43e-02 | 7.46e-02 |
| ENSG00000118503 | 1.85e-01 | 2.53e-01 |
| ENSG00000118507 | 5.13e-14 | 8.76e-13 |
| ENSG00000118508 | 5.41e-05 | 1.86e-04 |
| ENSG00000118513 | 7.29e-02 | 1.15e-01 |
| ENSG00000118514 | 4.11e-01 | 4.94e-01 |
| ENSG00000118515 | 1.87e-13 | 2.88e-12 |
| ENSG00000118518 | 1.20e-02 | 2.39e-02 |
| ENSG00000118520 | 2.03e-02 | 3.78e-02 |
| ENSG00000118523 | 2.22e-04 | 6.76e-04 |
| ENSG00000118526 | 2.95e-21 | 2.83e-19 |
| ENSG00000118557 | 2.30e-08 | 1.44e-07 |
| ENSG00000118564 | 3.42e-09 | 2.47e-08 |
| ENSG00000118579 | 2.54e-04 | 7.62e-04 |
| ENSG00000118596 | 2.48e-07 | 1.31e-06 |
| ENSG00000118600 | 2.04e-11 | 2.15e-10 |
| ENSG00000118620 | 9.12e-02 | 1.39e-01 |
| ENSG00000118640 | 2.54e-08 | 1.58e-07 |
| ENSG00000118655 | 2.90e-10 | 2.51e-09 |
| ENSG00000118680 | 1.14e-08 | 7.50e-08 |
| ENSG00000118689 | 3.51e-01 | 4.33e-01 |
| ENSG00000118690 | 5.26e-08 | 3.12e-07 |
| ENSG00000118702 | 7.18e-01 | 7.77e-01 |
| ENSG00000118705 | 4.96e-16 | 1.27e-14 |
| ENSG00000118707 | 1.86e-22 | 2.53e-20 |
| ENSG00000118729 | 2.66e-14 | 4.81e-13 |
| ENSG00000118733 | 8.83e-18 | 3.30e-16 |
| ENSG00000118762 | 2.94e-01 | 3.74e-01 |
| ENSG00000118777 | 2.13e-25 | 8.04e-23 |
| ENSG00000118785 | 7.41e-14 | 1.23e-12 |
| ENSG00000118804 | 8.49e-19 | 4.10e-17 |
| ENSG00000118816 | 9.93e-01 | 9.95e-01 |
| ENSG00000118849 | 1.01e-06 | 4.76e-06 |
| ENSG00000118855 | 2.75e-05 | 1.00e-04 |
| ENSG00000118873 | 3.53e-02 | 6.14e-02 |
| ENSG00000118894 | 1.51e-11 | 1.62e-10 |
| ENSG00000118898 | 1.27e-02 | 2.51e-02 |
| ENSG00000118900 | 6.85e-01 | 7.50e-01 |
| ENSG00000118903 | 3.46e-01 | 4.29e-01 |
| ENSG00000118922 | 1.67e-06 | 7.55e-06 |
| ENSG00000118939 | 4.18e-11 | 4.18e-10 |
| ENSG00000118946 | 3.32e-11 | 3.38e-10 |
| ENSG00000118960 | 4.32e-01 | 5.15e-01 |
| ENSG00000118961 | 9.30e-05 | 3.05e-04 |
| ENSG00000118965 | 8.25e-08 | 4.71e-07 |
| ENSG00000118971 | 9.03e-04 | 2.41e-03 |
| ENSG00000118972 | 3.69e-04 | 1.07e-03 |
| ENSG00000118976 | 1.23e-02 | 2.45e-02 |
| ENSG00000118985 | 5.45e-03 | 1.20e-02 |
| ENSG00000118990 | 6.01e-01 | 6.74e-01 |
| ENSG00000118997 | 4.86e-02 | 8.08e-02 |
| ENSG00000119004 | 7.09e-01 | 7.70e-01 |
| ENSG00000119013 | 8.93e-07 | 4.27e-06 |
| ENSG00000119041 | 6.43e-11 | 6.23e-10 |
| ENSG00000119042 | 2.48e-06 | 1.09e-05 |
| ENSG00000119048 | 2.32e-01 | 3.07e-01 |
| ENSG00000119121 | 4.21e-20 | 2.83e-18 |
| ENSG00000119125 | 6.13e-02 | 9.88e-02 |
| ENSG00000119138 | 6.52e-20 | 4.12e-18 |
| ENSG00000119139 | 1.06e-03 | 2.78e-03 |
| ENSG00000119147 | 4.07e-25 | 1.39e-22 |
| ENSG00000119185 | 1.78e-05 | 6.71e-05 |
| ENSG00000119203 | 3.92e-15 | 8.35e-14 |
| ENSG00000119227 | 3.26e-16 | 8.70e-15 |
| ENSG00000119231 | 4.91e-08 | 2.93e-07 |
| ENSG00000119242 | 5.66e-01 | 6.43e-01 |
| ENSG00000119280 | 4.45e-17 | 1.44e-15 |
| ENSG00000119283 | 1.99e-02 | 3.72e-02 |
| ENSG00000119285 | 1.19e-17 | 4.31e-16 |
| ENSG00000119314 | 1.86e-03 | 4.61e-03 |
| ENSG00000119318 | 7.47e-07 | 3.61e-06 |
| ENSG00000119321 | 5.80e-01 | 6.55e-01 |
| ENSG00000119326 | 1.06e-02 | 2.16e-02 |
| ENSG00000119328 | 1.55e-08 | 1.00e-07 |
| ENSG00000119333 | 9.74e-09 | 6.49e-08 |
| ENSG00000119335 | 4.55e-14 | 7.85e-13 |
| ENSG00000119383 | 2.05e-02 | 3.82e-02 |
| ENSG00000119392 | 5.93e-02 | 9.61e-02 |
| ENSG00000119396 | 8.38e-04 | 2.25e-03 |
| ENSG00000119397 | 9.22e-03 | 1.90e-02 |
| ENSG00000119401 | 1.78e-09 | 1.35e-08 |
| ENSG00000119402 | 1.09e-02 | 2.19e-02 |
| ENSG00000119403 | 1.68e-15 | 3.81e-14 |
| ENSG00000119408 | 8.15e-16 | 1.98e-14 |
| ENSG00000119411 | 1.85e-05 | 6.97e-05 |
| ENSG00000119414 | 1.60e-03 | 4.02e-03 |
| ENSG00000119421 | 6.60e-01 | 7.27e-01 |
| ENSG00000119431 | 1.64e-02 | 3.14e-02 |
| ENSG00000119440 | 1.57e-02 | 3.02e-02 |
| ENSG00000119446 | 3.88e-02 | 6.65e-02 |
| ENSG00000119457 | 4.71e-01 | 5.53e-01 |
| ENSG00000119471 | 1.24e-01 | 1.80e-01 |
| ENSG00000119487 | 8.34e-02 | 1.29e-01 |
| ENSG00000119508 | 9.02e-01 | 9.27e-01 |
| ENSG00000119509 | 6.68e-07 | 3.26e-06 |
| ENSG00000119514 | 1.24e-14 | 2.39e-13 |
| ENSG00000119522 | 8.23e-01 | 8.65e-01 |
| ENSG00000119523 | 7.06e-07 | 3.43e-06 |
| ENSG00000119535 | 1.70e-03 | 4.24e-03 |
| ENSG00000119537 | 8.67e-01 | 9.00e-01 |
| ENSG00000119541 | 3.42e-15 | 7.35e-14 |
| ENSG00000119547 | 1.12e-13 | 1.81e-12 |
| ENSG00000119559 | 9.75e-02 | 1.47e-01 |
| ENSG00000119574 | 3.68e-04 | 1.07e-03 |
| ENSG00000119596 | 2.01e-01 | 2.71e-01 |
| ENSG00000119599 | 8.31e-04 | 2.24e-03 |
| ENSG00000119608 | 2.51e-01 | 3.28e-01 |
| ENSG00000119614 | 1.74e-01 | 2.41e-01 |
| ENSG00000119616 | 8.02e-02 | 1.24e-01 |
| ENSG00000119630 | 1.24e-10 | 1.14e-09 |
| ENSG00000119632 | 2.33e-01 | 3.08e-01 |
| ENSG00000119636 | 1.86e-07 | 9.97e-07 |
| ENSG00000119638 | 5.51e-01 | 6.28e-01 |
| ENSG00000119640 | 2.59e-10 | 2.27e-09 |
| ENSG00000119650 | 2.92e-01 | 3.72e-01 |
| ENSG00000119655 | 3.99e-04 | 1.15e-03 |
| ENSG00000119661 | 2.57e-01 | 3.34e-01 |
| ENSG00000119669 | 6.72e-02 | 1.07e-01 |
| ENSG00000119673 | 3.12e-09 | 2.26e-08 |
| ENSG00000119681 | 1.08e-07 | 6.08e-07 |
| ENSG00000119682 | 6.97e-03 | 1.48e-02 |
| ENSG00000119684 | 2.82e-06 | 1.23e-05 |
| ENSG00000119685 | 4.62e-06 | 1.94e-05 |
| ENSG00000119686 | 6.24e-15 | 1.27e-13 |
| ENSG00000119688 | 7.11e-05 | 2.39e-04 |
| ENSG00000119689 | 1.96e-10 | 1.75e-09 |
| ENSG00000119698 | 2.62e-01 | 3.40e-01 |
| ENSG00000119699 | 2.87e-01 | 3.67e-01 |
| ENSG00000119703 | 8.94e-03 | 1.85e-02 |
| ENSG00000119705 | 2.18e-01 | 2.91e-01 |
| ENSG00000119707 | 2.24e-01 | 2.98e-01 |
| ENSG00000119711 | 1.21e-12 | 1.61e-11 |
| ENSG00000119714 | 1.85e-02 | 3.48e-02 |
| ENSG00000119715 | 7.68e-01 | 8.20e-01 |
| ENSG00000119718 | 5.46e-05 | 1.87e-04 |
| ENSG00000119720 | 5.00e-02 | 8.29e-02 |
| ENSG00000119723 | 5.21e-06 | 2.16e-05 |
| ENSG00000119725 | 5.20e-02 | 8.58e-02 |
| ENSG00000119729 | 8.12e-01 | 8.56e-01 |
| ENSG00000119737 | 1.63e-04 | 5.11e-04 |
| ENSG00000119760 | 2.43e-06 | 1.07e-05 |
| ENSG00000119771 | 2.07e-12 | 2.65e-11 |
| ENSG00000119772 | 4.07e-04 | 1.17e-03 |
| ENSG00000119777 | 3.78e-04 | 1.10e-03 |
| ENSG00000119778 | 7.42e-01 | 7.98e-01 |
| ENSG00000119782 | 1.63e-13 | 2.54e-12 |
| ENSG00000119787 | 2.61e-01 | 3.38e-01 |
| ENSG00000119801 | 1.34e-09 | 1.03e-08 |
| ENSG00000119812 | 8.30e-18 | 3.12e-16 |
| ENSG00000119820 | 5.67e-02 | 9.23e-02 |
| ENSG00000119844 | 6.63e-04 | 1.83e-03 |
| ENSG00000119862 | 3.91e-12 | 4.72e-11 |
| ENSG00000119865 | 1.93e-12 | 2.48e-11 |
| ENSG00000119866 | 1.52e-05 | 5.81e-05 |
| ENSG00000119878 | 5.07e-02 | 8.40e-02 |
| ENSG00000119888 | 5.53e-03 | 1.21e-02 |
| ENSG00000119899 | 1.05e-09 | 8.24e-09 |
| ENSG00000119900 | 1.50e-02 | 2.90e-02 |
| ENSG00000119906 | 9.10e-01 | 9.34e-01 |
| ENSG00000119912 | 2.14e-03 | 5.21e-03 |
| ENSG00000119913 | 1.90e-01 | 2.60e-01 |
| ENSG00000119915 | 1.74e-01 | 2.41e-01 |
| ENSG00000119917 | 3.45e-02 | 6.01e-02 |
| ENSG00000119919 | 3.61e-23 | 6.17e-21 |
| ENSG00000119922 | 2.56e-04 | 7.67e-04 |
| ENSG00000119927 | 3.36e-01 | 4.18e-01 |
| ENSG00000119929 | 7.04e-02 | 1.11e-01 |
| ENSG00000119938 | 5.15e-09 | 3.62e-08 |
| ENSG00000119943 | 4.74e-04 | 1.35e-03 |
| ENSG00000119946 | 2.40e-11 | 2.50e-10 |
| ENSG00000119950 | 2.75e-20 | 1.95e-18 |
| ENSG00000119953 | 7.47e-04 | 2.03e-03 |
| ENSG00000119965 | 4.32e-03 | 9.75e-03 |
| ENSG00000119969 | 2.47e-14 | 4.50e-13 |
| ENSG00000119973 | 3.75e-02 | 6.47e-02 |
| ENSG00000119977 | 7.41e-01 | 7.97e-01 |
| ENSG00000119979 | 1.11e-05 | 4.37e-05 |
| ENSG00000119986 | 6.53e-08 | 3.80e-07 |
| ENSG00000120008 | 6.07e-01 | 6.79e-01 |
| ENSG00000120029 | 5.71e-11 | 5.58e-10 |
| ENSG00000120049 | 1.33e-01 | 1.92e-01 |
| ENSG00000120051 | 3.35e-01 | 4.17e-01 |
| ENSG00000120053 | 4.15e-11 | 4.16e-10 |
| ENSG00000120054 | 1.19e-04 | 3.83e-04 |
| ENSG00000120055 | 1.53e-03 | 3.86e-03 |
| ENSG00000120057 | 3.65e-16 | 9.63e-15 |
| ENSG00000120063 | 1.16e-13 | 1.85e-12 |
| ENSG00000120068 | 6.97e-12 | 8.01e-11 |
| ENSG00000120071 | 3.85e-02 | 6.61e-02 |
| ENSG00000120075 | 1.09e-01 | 1.62e-01 |
| ENSG00000120088 | 5.81e-01 | 6.56e-01 |
| ENSG00000120093 | 3.65e-05 | 1.30e-04 |
| ENSG00000120094 | 8.44e-01 | 8.82e-01 |
| ENSG00000120129 | 1.35e-10 | 1.24e-09 |
| ENSG00000120137 | 8.41e-11 | 8.00e-10 |
| ENSG00000120149 | 3.08e-21 | 2.95e-19 |
| ENSG00000120156 | 2.79e-09 | 2.04e-08 |
| ENSG00000120158 | 1.55e-22 | 2.15e-20 |
| ENSG00000120159 | 5.91e-02 | 9.57e-02 |
| ENSG00000120160 | 1.04e-01 | 1.56e-01 |
| ENSG00000120162 | 5.33e-19 | 2.70e-17 |
| ENSG00000120210 | 6.55e-02 | 1.05e-01 |
| ENSG00000120211 | 2.04e-02 | 3.81e-02 |
| ENSG00000120215 | 4.02e-01 | 4.85e-01 |
| ENSG00000120217 | 5.92e-01 | 6.66e-01 |
| ENSG00000120251 | 6.46e-08 | 3.77e-07 |
| ENSG00000120253 | 9.40e-19 | 4.46e-17 |
| ENSG00000120254 | 3.53e-32 | 1.23e-28 |
| ENSG00000120256 | 1.73e-18 | 7.61e-17 |
| ENSG00000120262 | 6.83e-04 | 1.88e-03 |
| ENSG00000120265 | 2.03e-02 | 3.79e-02 |
| ENSG00000120278 | 3.30e-01 | 4.12e-01 |
| ENSG00000120279 | 3.60e-08 | 2.19e-07 |
| ENSG00000120280 | 8.27e-09 | 5.59e-08 |
| ENSG00000120306 | 2.55e-08 | 1.59e-07 |
| ENSG00000120314 | 6.85e-01 | 7.49e-01 |
| ENSG00000120318 | 1.26e-06 | 5.84e-06 |
| ENSG00000120322 | 1.45e-01 | 2.06e-01 |
| ENSG00000120324 | 1.43e-04 | 4.51e-04 |
| ENSG00000120327 | 7.54e-11 | 7.22e-10 |
| ENSG00000120328 | 1.16e-01 | 1.71e-01 |
| ENSG00000120329 | 1.07e-04 | 3.47e-04 |
| ENSG00000120332 | 1.40e-01 | 2.01e-01 |
| ENSG00000120333 | 8.09e-02 | 1.25e-01 |
| ENSG00000120334 | 1.10e-12 | 1.47e-11 |
| ENSG00000120337 | 1.03e-02 | 2.10e-02 |
| ENSG00000120341 | 7.94e-07 | 3.82e-06 |
| ENSG00000120370 | 1.84e-01 | 2.52e-01 |
| ENSG00000120436 | 1.68e-06 | 7.61e-06 |
| ENSG00000120437 | 3.44e-01 | 4.26e-01 |
| ENSG00000120438 | 9.72e-06 | 3.86e-05 |
| ENSG00000120440 | 7.45e-05 | 2.49e-04 |
| ENSG00000120451 | 6.76e-04 | 1.86e-03 |
| ENSG00000120457 | 8.39e-05 | 2.77e-04 |
| ENSG00000120458 | 1.06e-02 | 2.15e-02 |
| ENSG00000120471 | 3.69e-01 | 4.52e-01 |
| ENSG00000120498 | 7.30e-22 | 8.57e-20 |
| ENSG00000120500 | 6.46e-01 | 7.15e-01 |
| ENSG00000120509 | 7.03e-03 | 1.50e-02 |
| ENSG00000120519 | 9.98e-01 | 9.99e-01 |
| ENSG00000120526 | 5.95e-21 | 5.11e-19 |
| ENSG00000120533 | 5.12e-06 | 2.13e-05 |
| ENSG00000120539 | 4.85e-13 | 6.96e-12 |
| ENSG00000120549 | 7.89e-02 | 1.23e-01 |
| ENSG00000120555 | 3.90e-02 | 6.68e-02 |
| ENSG00000120594 | 2.99e-02 | 5.31e-02 |
| ENSG00000120616 | 1.01e-03 | 2.65e-03 |
| ENSG00000120645 | 2.42e-04 | 7.29e-04 |
| ENSG00000120647 | 3.68e-08 | 2.23e-07 |
| ENSG00000120656 | 4.29e-04 | 1.23e-03 |
| ENSG00000120658 | 4.52e-03 | 1.01e-02 |
| ENSG00000120659 | 1.13e-07 | 6.31e-07 |
| ENSG00000120662 | 2.84e-08 | 1.75e-07 |
| ENSG00000120664 | 5.58e-01 | 6.35e-01 |
| ENSG00000120669 | 4.55e-13 | 6.56e-12 |
| ENSG00000120675 | 2.50e-03 | 6.00e-03 |
| ENSG00000120685 | 2.37e-14 | 4.34e-13 |
| ENSG00000120686 | 1.96e-06 | 8.76e-06 |
| ENSG00000120688 | 4.87e-04 | 1.38e-03 |
| ENSG00000120690 | 3.70e-01 | 4.53e-01 |
| ENSG00000120693 | 1.60e-03 | 4.02e-03 |
| ENSG00000120694 | 7.88e-14 | 1.30e-12 |
| ENSG00000120696 | 1.01e-01 | 1.52e-01 |
| ENSG00000120697 | 1.24e-07 | 6.89e-07 |
| ENSG00000120699 | 4.82e-17 | 1.54e-15 |
| ENSG00000120705 | 9.18e-03 | 1.89e-02 |
| ENSG00000120708 | 2.02e-25 | 7.91e-23 |
| ENSG00000120709 | 7.27e-02 | 1.14e-01 |
| ENSG00000120725 | 1.02e-05 | 4.04e-05 |
| ENSG00000120727 | 8.71e-03 | 1.81e-02 |
| ENSG00000120729 | 5.22e-16 | 1.33e-14 |
| ENSG00000120733 | 2.63e-01 | 3.40e-01 |
| ENSG00000120738 | 1.59e-01 | 2.23e-01 |
| ENSG00000120742 | 3.05e-03 | 7.16e-03 |
| ENSG00000120756 | 4.02e-11 | 4.04e-10 |
| ENSG00000120784 | 4.23e-02 | 7.17e-02 |
| ENSG00000120798 | 1.07e-07 | 6.01e-07 |
| ENSG00000120800 | 9.63e-12 | 1.08e-10 |
| ENSG00000120802 | 5.66e-06 | 2.33e-05 |
| ENSG00000120805 | 7.88e-01 | 8.36e-01 |
| ENSG00000120820 | 8.29e-02 | 1.28e-01 |
| ENSG00000120832 | 6.54e-01 | 7.21e-01 |
| ENSG00000120833 | 2.91e-13 | 4.35e-12 |
| ENSG00000120837 | 1.87e-04 | 5.78e-04 |
| ENSG00000120860 | 3.63e-02 | 6.29e-02 |
| ENSG00000120868 | 1.60e-02 | 3.07e-02 |
| ENSG00000120875 | 4.46e-14 | 7.72e-13 |
| ENSG00000120885 | 2.68e-17 | 8.99e-16 |
| ENSG00000120889 | 1.10e-17 | 4.02e-16 |
| ENSG00000120896 | 5.33e-11 | 5.23e-10 |
| ENSG00000120899 | 5.44e-15 | 1.12e-13 |
| ENSG00000120903 | 2.34e-01 | 3.09e-01 |
| ENSG00000120907 | 1.71e-15 | 3.87e-14 |
| ENSG00000120910 | 7.25e-04 | 1.98e-03 |
| ENSG00000120913 | 8.85e-14 | 1.45e-12 |
| ENSG00000120915 | 3.20e-18 | 1.33e-16 |
| ENSG00000120925 | 1.89e-03 | 4.67e-03 |
| ENSG00000120937 | 9.34e-02 | 1.42e-01 |
| ENSG00000120942 | 2.65e-05 | 9.68e-05 |
| ENSG00000120948 | 2.28e-03 | 5.54e-03 |
| ENSG00000120949 | 3.44e-01 | 4.26e-01 |
| ENSG00000120963 | 1.12e-08 | 7.43e-08 |
| ENSG00000120992 | 6.19e-05 | 2.10e-04 |
| ENSG00000121005 | 4.61e-03 | 1.03e-02 |
| ENSG00000121022 | 5.45e-04 | 1.53e-03 |
| ENSG00000121039 | 3.12e-11 | 3.19e-10 |
| ENSG00000121053 | 9.91e-01 | 9.93e-01 |
| ENSG00000121057 | 1.40e-01 | 2.00e-01 |
| ENSG00000121058 | 7.19e-03 | 1.53e-02 |
| ENSG00000121060 | 2.69e-03 | 6.41e-03 |
| ENSG00000121064 | 7.77e-05 | 2.58e-04 |
| ENSG00000121067 | 3.34e-05 | 1.20e-04 |
| ENSG00000121068 | 6.94e-01 | 7.57e-01 |
| ENSG00000121073 | 2.58e-04 | 7.74e-04 |
| ENSG00000121075 | 1.70e-04 | 5.30e-04 |
| ENSG00000121089 | 3.13e-11 | 3.21e-10 |
| ENSG00000121101 | 4.66e-01 | 5.48e-01 |
| ENSG00000121104 | 2.41e-17 | 8.22e-16 |
| ENSG00000121152 | 7.34e-14 | 1.22e-12 |
| ENSG00000121207 | 6.81e-10 | 5.52e-09 |
| ENSG00000121210 | 2.27e-02 | 4.17e-02 |
| ENSG00000121211 | 1.00e-11 | 1.12e-10 |
| ENSG00000121236 | 2.13e-02 | 3.94e-02 |
| ENSG00000121270 | 2.38e-03 | 5.74e-03 |
| ENSG00000121274 | 1.29e-02 | 2.55e-02 |
| ENSG00000121281 | 3.27e-01 | 4.09e-01 |
| ENSG00000121289 | 1.67e-11 | 1.79e-10 |
| ENSG00000121297 | 1.29e-05 | 5.02e-05 |
| ENSG00000121310 | 4.02e-05 | 1.42e-04 |
| ENSG00000121314 | 6.20e-01 | 6.92e-01 |
| ENSG00000121316 | 7.75e-01 | 8.25e-01 |
| ENSG00000121318 | 5.45e-02 | 8.93e-02 |
| ENSG00000121335 | 2.25e-04 | 6.84e-04 |
| ENSG00000121350 | 1.81e-07 | 9.77e-07 |
| ENSG00000121361 | 8.66e-03 | 1.80e-02 |
| ENSG00000121380 | 1.40e-01 | 2.00e-01 |
| ENSG00000121390 | 1.66e-09 | 1.26e-08 |
| ENSG00000121406 | 2.92e-09 | 2.13e-08 |
| ENSG00000121410 | 1.06e-02 | 2.16e-02 |
| ENSG00000121413 | 6.73e-11 | 6.51e-10 |
| ENSG00000121417 | 7.14e-07 | 3.46e-06 |
| ENSG00000121440 | 2.16e-08 | 1.36e-07 |
| ENSG00000121446 | 7.60e-01 | 8.13e-01 |
| ENSG00000121454 | 5.83e-03 | 1.27e-02 |
| ENSG00000121481 | 3.30e-02 | 5.78e-02 |
| ENSG00000121486 | 1.29e-01 | 1.87e-01 |
| ENSG00000121542 | 2.49e-01 | 3.26e-01 |
| ENSG00000121552 | 7.14e-01 | 7.74e-01 |
| ENSG00000121570 | 5.27e-02 | 8.67e-02 |
| ENSG00000121577 | 1.87e-05 | 7.02e-05 |
| ENSG00000121578 | 1.16e-03 | 3.01e-03 |
| ENSG00000121579 | 1.15e-05 | 4.51e-05 |
| ENSG00000121594 | 7.00e-05 | 2.35e-04 |
| ENSG00000121621 | 1.16e-13 | 1.85e-12 |
| ENSG00000121644 | 1.02e-05 | 4.04e-05 |
| ENSG00000121653 | 7.91e-01 | 8.39e-01 |
| ENSG00000121671 | 1.13e-11 | 1.24e-10 |
| ENSG00000121680 | 5.69e-03 | 1.24e-02 |
| ENSG00000121690 | 7.23e-01 | 7.81e-01 |
| ENSG00000121691 | 2.55e-12 | 3.20e-11 |
| ENSG00000121716 | 2.00e-13 | 3.07e-12 |
| ENSG00000121741 | 9.88e-06 | 3.92e-05 |
| ENSG00000121742 | 2.62e-02 | 4.73e-02 |
| ENSG00000121743 | 5.91e-14 | 9.97e-13 |
| ENSG00000121749 | 1.59e-01 | 2.23e-01 |
| ENSG00000121753 | 7.55e-01 | 8.09e-01 |
| ENSG00000121764 | 7.70e-01 | 8.21e-01 |
| ENSG00000121766 | 2.48e-01 | 3.24e-01 |
| ENSG00000121769 | 4.58e-03 | 1.03e-02 |
| ENSG00000121774 | 3.35e-05 | 1.20e-04 |
| ENSG00000121775 | 4.85e-03 | 1.08e-02 |
| ENSG00000121797 | 4.79e-04 | 1.36e-03 |
| ENSG00000121807 | 6.09e-14 | 1.02e-12 |
| ENSG00000121851 | 2.64e-07 | 1.39e-06 |
| ENSG00000121853 | 8.84e-01 | 9.13e-01 |
| ENSG00000121858 | 1.10e-14 | 2.14e-13 |
| ENSG00000121864 | 3.37e-02 | 5.88e-02 |
| ENSG00000121871 | 1.86e-19 | 1.06e-17 |
| ENSG00000121879 | 4.37e-01 | 5.20e-01 |
| ENSG00000121892 | 4.11e-01 | 4.94e-01 |
| ENSG00000121895 | 3.62e-11 | 3.66e-10 |
| ENSG00000121897 | 3.64e-05 | 1.30e-04 |
| ENSG00000121898 | 3.38e-08 | 2.07e-07 |
| ENSG00000121900 | 1.84e-11 | 1.96e-10 |
| ENSG00000121903 | 3.08e-01 | 3.89e-01 |
| ENSG00000121904 | 6.76e-11 | 6.53e-10 |
| ENSG00000121905 | 1.87e-07 | 1.01e-06 |
| ENSG00000121931 | 1.10e-01 | 1.64e-01 |
| ENSG00000121933 | 2.01e-14 | 3.74e-13 |
| ENSG00000121940 | 1.29e-01 | 1.87e-01 |
| ENSG00000121957 | 4.18e-12 | 5.02e-11 |
| ENSG00000121964 | 1.76e-02 | 3.34e-02 |
| ENSG00000121966 | 4.88e-01 | 5.69e-01 |
| ENSG00000121988 | 4.12e-12 | 4.95e-11 |
| ENSG00000121989 | 2.92e-05 | 1.06e-04 |
| ENSG00000122008 | 9.98e-01 | 9.99e-01 |
| ENSG00000122012 | 2.34e-04 | 7.08e-04 |
| ENSG00000122025 | 4.66e-12 | 5.54e-11 |
| ENSG00000122026 | 7.29e-03 | 1.55e-02 |
| ENSG00000122033 | 1.52e-02 | 2.93e-02 |
| ENSG00000122034 | 1.59e-21 | 1.70e-19 |
| ENSG00000122035 | 1.86e-04 | 5.74e-04 |
| ENSG00000122042 | 2.71e-09 | 1.99e-08 |
| ENSG00000122043 | 7.56e-01 | 8.10e-01 |
| ENSG00000122068 | 8.67e-01 | 9.00e-01 |
| ENSG00000122085 | 6.13e-01 | 6.85e-01 |
| ENSG00000122121 | 2.94e-05 | 1.07e-04 |
| ENSG00000122122 | 4.73e-08 | 2.82e-07 |
| ENSG00000122126 | 6.16e-01 | 6.87e-01 |
| ENSG00000122133 | 1.76e-17 | 6.20e-16 |
| ENSG00000122136 | 2.08e-06 | 9.24e-06 |
| ENSG00000122140 | 2.81e-07 | 1.47e-06 |
| ENSG00000122176 | 6.57e-01 | 7.24e-01 |
| ENSG00000122180 | 8.98e-01 | 9.24e-01 |
| ENSG00000122188 | 7.08e-10 | 5.73e-09 |
| ENSG00000122194 | 1.85e-01 | 2.54e-01 |
| ENSG00000122203 | 9.80e-03 | 2.00e-02 |
| ENSG00000122218 | 3.11e-03 | 7.28e-03 |
| ENSG00000122223 | 1.14e-07 | 6.38e-07 |
| ENSG00000122224 | 1.02e-15 | 2.44e-14 |
| ENSG00000122254 | 5.89e-01 | 6.63e-01 |
| ENSG00000122257 | 2.81e-02 | 5.03e-02 |
| ENSG00000122299 | 7.43e-01 | 7.99e-01 |
| ENSG00000122335 | 3.96e-01 | 4.79e-01 |
| ENSG00000122359 | 2.42e-05 | 8.92e-05 |
| ENSG00000122367 | 3.10e-15 | 6.73e-14 |
| ENSG00000122375 | 7.55e-08 | 4.34e-07 |
| ENSG00000122376 | 3.75e-01 | 4.57e-01 |
| ENSG00000122378 | 2.86e-07 | 1.49e-06 |
| ENSG00000122386 | 5.82e-08 | 3.43e-07 |
| ENSG00000122390 | 1.28e-05 | 4.98e-05 |
| ENSG00000122406 | 1.05e-01 | 1.56e-01 |
| ENSG00000122417 | 1.99e-01 | 2.70e-01 |
| ENSG00000122420 | 8.94e-06 | 3.57e-05 |
| ENSG00000122432 | 7.60e-01 | 8.13e-01 |
| ENSG00000122435 | 5.94e-02 | 9.62e-02 |
| ENSG00000122477 | 5.21e-02 | 8.59e-02 |
| ENSG00000122481 | 6.76e-02 | 1.07e-01 |
| ENSG00000122482 | 2.73e-01 | 3.52e-01 |
| ENSG00000122483 | 4.80e-04 | 1.36e-03 |
| ENSG00000122484 | 6.52e-03 | 1.40e-02 |
| ENSG00000122490 | 3.89e-12 | 4.70e-11 |
| ENSG00000122507 | 5.22e-01 | 6.02e-01 |
| ENSG00000122512 | 2.90e-07 | 1.51e-06 |
| ENSG00000122515 | 1.98e-15 | 4.43e-14 |
| ENSG00000122543 | 4.74e-01 | 5.56e-01 |
| ENSG00000122545 | 5.64e-01 | 6.41e-01 |
| ENSG00000122547 | 2.25e-10 | 1.99e-09 |
| ENSG00000122548 | 3.14e-01 | 3.95e-01 |
| ENSG00000122550 | 3.12e-01 | 3.93e-01 |
| ENSG00000122557 | 5.77e-01 | 6.53e-01 |
| ENSG00000122565 | 1.11e-14 | 2.17e-13 |
| ENSG00000122566 | 2.02e-07 | 1.08e-06 |
| ENSG00000122574 | 6.58e-07 | 3.21e-06 |
| ENSG00000122584 | 4.04e-15 | 8.57e-14 |
| ENSG00000122585 | 1.31e-20 | 1.03e-18 |
| ENSG00000122591 | 1.08e-02 | 2.17e-02 |
| ENSG00000122592 | 1.73e-01 | 2.40e-01 |
| ENSG00000122641 | 9.94e-30 | 1.81e-26 |
| ENSG00000122642 | 2.45e-16 | 6.74e-15 |
| ENSG00000122643 | 2.25e-02 | 4.15e-02 |
| ENSG00000122644 | 5.38e-06 | 2.23e-05 |
| ENSG00000122674 | 3.10e-03 | 7.26e-03 |
| ENSG00000122678 | 5.88e-17 | 1.84e-15 |
| ENSG00000122679 | 9.83e-01 | 9.87e-01 |
| ENSG00000122687 | 1.15e-12 | 1.54e-11 |
| ENSG00000122691 | 2.34e-12 | 2.96e-11 |
| ENSG00000122692 | 3.20e-04 | 9.41e-04 |
| ENSG00000122694 | 2.54e-21 | 2.52e-19 |
| ENSG00000122696 | 1.93e-04 | 5.94e-04 |
| ENSG00000122705 | 1.91e-05 | 7.15e-05 |
| ENSG00000122707 | 2.82e-14 | 5.06e-13 |
| ENSG00000122711 | 2.99e-01 | 3.79e-01 |
| ENSG00000122728 | 3.78e-04 | 1.10e-03 |
| ENSG00000122729 | 8.29e-02 | 1.28e-01 |
| ENSG00000122733 | 4.55e-12 | 5.42e-11 |
| ENSG00000122735 | 2.27e-01 | 3.01e-01 |
| ENSG00000122741 | 2.12e-02 | 3.94e-02 |
| ENSG00000122756 | 8.39e-20 | 5.19e-18 |
| ENSG00000122778 | 8.19e-28 | 7.80e-25 |
| ENSG00000122779 | 6.06e-09 | 4.20e-08 |
| ENSG00000122783 | 1.53e-01 | 2.16e-01 |
| ENSG00000122786 | 2.19e-02 | 4.04e-02 |
| ENSG00000122787 | 6.34e-02 | 1.02e-01 |
| ENSG00000122824 | 6.33e-10 | 5.16e-09 |
| ENSG00000122852 | 2.19e-09 | 1.63e-08 |
| ENSG00000122859 | 9.42e-07 | 4.48e-06 |
| ENSG00000122861 | 5.10e-19 | 2.60e-17 |
| ENSG00000122862 | 4.74e-03 | 1.06e-02 |
| ENSG00000122863 | 2.17e-02 | 4.02e-02 |
| ENSG00000122870 | 2.20e-02 | 4.07e-02 |
| ENSG00000122872 | 5.86e-02 | 9.50e-02 |
| ENSG00000122873 | 7.69e-01 | 8.20e-01 |
| ENSG00000122877 | 8.20e-01 | 8.63e-01 |
| ENSG00000122882 | 3.29e-04 | 9.66e-04 |
| ENSG00000122884 | 9.90e-09 | 6.59e-08 |
| ENSG00000122912 | 7.82e-04 | 2.12e-03 |
| ENSG00000122952 | 1.36e-08 | 8.86e-08 |
| ENSG00000122958 | 2.31e-01 | 3.06e-01 |
| ENSG00000122965 | 5.95e-14 | 1.00e-12 |
| ENSG00000122966 | 2.44e-13 | 3.70e-12 |
| ENSG00000122970 | 1.38e-07 | 7.59e-07 |
| ENSG00000122971 | 2.47e-21 | 2.47e-19 |
| ENSG00000122986 | 1.06e-09 | 8.33e-09 |
| ENSG00000123009 | 3.98e-10 | 3.37e-09 |
| ENSG00000123064 | 5.25e-07 | 2.61e-06 |
| ENSG00000123066 | 3.77e-02 | 6.50e-02 |
| ENSG00000123080 | 1.09e-04 | 3.51e-04 |
| ENSG00000123091 | 5.02e-12 | 5.94e-11 |
| ENSG00000123094 | 6.50e-03 | 1.40e-02 |
| ENSG00000123095 | 9.97e-08 | 5.62e-07 |
| ENSG00000123096 | 1.98e-12 | 2.54e-11 |
| ENSG00000123104 | 2.97e-01 | 3.77e-01 |
| ENSG00000123106 | 4.15e-02 | 7.05e-02 |
| ENSG00000123119 | 2.30e-11 | 2.40e-10 |
| ENSG00000123124 | 1.43e-01 | 2.04e-01 |
| ENSG00000123130 | 4.07e-11 | 4.08e-10 |
| ENSG00000123131 | 8.05e-17 | 2.44e-15 |
| ENSG00000123136 | 1.39e-13 | 2.19e-12 |
| ENSG00000123143 | 2.70e-04 | 8.07e-04 |
| ENSG00000123144 | 2.96e-05 | 1.07e-04 |
| ENSG00000123146 | 6.13e-05 | 2.08e-04 |
| ENSG00000123154 | 9.61e-05 | 3.14e-04 |
| ENSG00000123159 | 5.23e-08 | 3.10e-07 |
| ENSG00000123171 | 9.73e-06 | 3.86e-05 |
| ENSG00000123178 | 1.95e-01 | 2.65e-01 |
| ENSG00000123179 | 3.97e-18 | 1.61e-16 |
| ENSG00000123191 | 7.34e-10 | 5.93e-09 |
| ENSG00000123200 | 2.49e-03 | 5.99e-03 |
| ENSG00000123201 | 1.74e-08 | 1.12e-07 |
| ENSG00000123213 | 1.82e-05 | 6.85e-05 |
| ENSG00000123219 | 5.16e-09 | 3.62e-08 |
| ENSG00000123240 | 1.10e-11 | 1.21e-10 |
| ENSG00000123243 | 2.29e-13 | 3.48e-12 |
| ENSG00000123268 | 3.95e-01 | 4.78e-01 |
| ENSG00000123297 | 5.48e-04 | 1.54e-03 |
| ENSG00000123329 | 6.08e-07 | 2.98e-06 |
| ENSG00000123338 | 4.88e-10 | 4.06e-09 |
| ENSG00000123342 | 5.87e-02 | 9.51e-02 |
| ENSG00000123349 | 4.94e-01 | 5.74e-01 |
| ENSG00000123352 | 7.51e-12 | 8.57e-11 |
| ENSG00000123353 | 2.81e-01 | 3.60e-01 |
| ENSG00000123358 | 4.24e-02 | 7.18e-02 |
| ENSG00000123360 | 5.70e-10 | 4.69e-09 |
| ENSG00000123364 | 1.83e-03 | 4.54e-03 |
| ENSG00000123374 | 7.14e-19 | 3.51e-17 |
| ENSG00000123384 | 2.30e-01 | 3.04e-01 |
| ENSG00000123388 | 2.87e-09 | 2.10e-08 |
| ENSG00000123395 | 8.37e-06 | 3.36e-05 |
| ENSG00000123405 | 5.41e-07 | 2.68e-06 |
| ENSG00000123407 | 6.39e-04 | 1.77e-03 |
| ENSG00000123411 | 9.08e-03 | 1.88e-02 |
| ENSG00000123415 | 2.01e-01 | 2.72e-01 |
| ENSG00000123416 | 7.19e-03 | 1.52e-02 |
| ENSG00000123427 | 1.65e-09 | 1.25e-08 |
| ENSG00000123444 | 2.00e-01 | 2.70e-01 |
| ENSG00000123453 | 1.15e-02 | 2.30e-02 |
| ENSG00000123454 | 6.84e-02 | 1.09e-01 |
| ENSG00000123472 | 1.38e-08 | 9.00e-08 |
| ENSG00000123473 | 6.85e-14 | 1.14e-12 |
| ENSG00000123485 | 2.58e-19 | 1.40e-17 |
| ENSG00000123496 | 2.91e-06 | 1.26e-05 |
| ENSG00000123500 | 5.29e-27 | 4.10e-24 |
| ENSG00000123505 | 2.15e-01 | 2.88e-01 |
| ENSG00000123545 | 2.17e-01 | 2.89e-01 |
| ENSG00000123552 | 2.62e-01 | 3.40e-01 |
| ENSG00000123560 | 1.51e-24 | 4.15e-22 |
| ENSG00000123561 | 2.42e-04 | 7.30e-04 |
| ENSG00000123562 | 9.46e-11 | 8.90e-10 |
| ENSG00000123569 | 7.07e-05 | 2.37e-04 |
| ENSG00000123570 | 4.81e-11 | 4.76e-10 |
| ENSG00000123572 | 1.00e-02 | 2.05e-02 |
| ENSG00000123575 | 1.85e-03 | 4.59e-03 |
| ENSG00000123584 | 1.33e-02 | 2.61e-02 |
| ENSG00000123595 | 1.29e-09 | 1.00e-08 |
| ENSG00000123600 | 6.62e-04 | 1.82e-03 |
| ENSG00000123607 | 1.72e-01 | 2.39e-01 |
| ENSG00000123609 | 2.75e-02 | 4.93e-02 |
| ENSG00000123610 | 1.63e-07 | 8.86e-07 |
| ENSG00000123612 | 1.45e-11 | 1.57e-10 |
| ENSG00000123636 | 6.34e-01 | 7.04e-01 |
| ENSG00000123643 | 1.07e-19 | 6.40e-18 |
| ENSG00000123684 | 4.76e-06 | 1.99e-05 |
| ENSG00000123685 | 1.49e-04 | 4.70e-04 |
| ENSG00000123689 | 5.29e-05 | 1.82e-04 |
| ENSG00000123700 | 4.83e-04 | 1.37e-03 |
| ENSG00000123728 | 3.52e-02 | 6.12e-02 |
| ENSG00000123737 | 5.15e-06 | 2.14e-05 |
| ENSG00000123739 | 1.34e-01 | 1.93e-01 |
| ENSG00000123810 | 8.01e-01 | 8.47e-01 |
| ENSG00000123815 | 3.65e-03 | 8.39e-03 |
| ENSG00000123836 | 2.09e-06 | 9.29e-06 |
| ENSG00000123838 | 2.00e-01 | 2.70e-01 |
| ENSG00000123843 | 1.89e-06 | 8.48e-06 |
| ENSG00000123870 | 4.67e-01 | 5.50e-01 |
| ENSG00000123892 | 2.68e-03 | 6.39e-03 |
| ENSG00000123901 | 1.30e-02 | 2.57e-02 |
| ENSG00000123908 | 5.57e-13 | 7.88e-12 |
| ENSG00000123933 | 1.93e-04 | 5.94e-04 |
| ENSG00000123965 | 1.01e-01 | 1.52e-01 |
| ENSG00000123975 | 2.13e-19 | 1.19e-17 |
| ENSG00000123977 | 2.32e-01 | 3.06e-01 |
| ENSG00000123983 | 1.29e-01 | 1.87e-01 |
| ENSG00000123989 | 1.05e-21 | 1.17e-19 |
| ENSG00000123992 | 5.79e-05 | 1.98e-04 |
| ENSG00000123999 | 3.74e-05 | 1.33e-04 |
| ENSG00000124003 | 1.44e-01 | 2.05e-01 |
| ENSG00000124006 | 9.60e-01 | 9.70e-01 |
| ENSG00000124019 | 4.79e-02 | 7.98e-02 |
| ENSG00000124067 | 2.27e-03 | 5.51e-03 |
| ENSG00000124074 | 6.95e-08 | 4.03e-07 |
| ENSG00000124089 | 3.16e-05 | 1.14e-04 |
| ENSG00000124091 | 1.09e-03 | 2.86e-03 |
| ENSG00000124092 | 1.45e-01 | 2.06e-01 |
| ENSG00000124097 | 1.46e-01 | 2.08e-01 |
| ENSG00000124098 | 3.41e-03 | 7.90e-03 |
| ENSG00000124102 | 5.29e-01 | 6.08e-01 |
| ENSG00000124103 | 8.56e-01 | 8.91e-01 |
| ENSG00000124104 | 3.10e-02 | 5.47e-02 |
| ENSG00000124107 | 2.08e-01 | 2.80e-01 |
| ENSG00000124116 | 4.95e-06 | 2.06e-05 |
| ENSG00000124120 | 2.54e-12 | 3.19e-11 |
| ENSG00000124126 | 7.24e-03 | 1.53e-02 |
| ENSG00000124134 | 6.34e-02 | 1.02e-01 |
| ENSG00000124140 | 4.84e-01 | 5.66e-01 |
| ENSG00000124143 | 5.12e-12 | 6.05e-11 |
| ENSG00000124145 | 2.48e-08 | 1.55e-07 |
| ENSG00000124151 | 9.92e-02 | 1.49e-01 |
| ENSG00000124155 | 5.32e-04 | 1.49e-03 |
| ENSG00000124157 | 8.74e-07 | 4.18e-06 |
| ENSG00000124159 | 2.44e-04 | 7.37e-04 |
| ENSG00000124160 | 1.15e-06 | 5.37e-06 |
| ENSG00000124164 | 4.06e-06 | 1.72e-05 |
| ENSG00000124171 | 8.41e-06 | 3.38e-05 |
| ENSG00000124172 | 3.32e-02 | 5.81e-02 |
| ENSG00000124177 | 2.06e-07 | 1.10e-06 |
| ENSG00000124181 | 8.63e-14 | 1.42e-12 |
| ENSG00000124191 | 3.69e-10 | 3.14e-09 |
| ENSG00000124193 | 7.80e-10 | 6.27e-09 |
| ENSG00000124194 | 4.08e-01 | 4.91e-01 |
| ENSG00000124196 | 5.89e-01 | 6.63e-01 |
| ENSG00000124198 | 4.90e-03 | 1.09e-02 |
| ENSG00000124201 | 6.34e-02 | 1.02e-01 |
| ENSG00000124203 | 8.39e-10 | 6.72e-09 |
| ENSG00000124205 | 2.77e-20 | 1.95e-18 |
| ENSG00000124207 | 8.46e-22 | 9.76e-20 |
| ENSG00000124208 | 5.76e-01 | 6.52e-01 |
| ENSG00000124209 | 4.26e-08 | 2.56e-07 |
| ENSG00000124212 | 1.40e-05 | 5.38e-05 |
| ENSG00000124214 | 1.19e-08 | 7.84e-08 |
| ENSG00000124215 | 3.07e-01 | 3.88e-01 |
| ENSG00000124216 | 1.90e-19 | 1.08e-17 |
| ENSG00000124217 | 1.87e-13 | 2.88e-12 |
| ENSG00000124222 | 6.37e-12 | 7.38e-11 |
| ENSG00000124224 | 4.80e-05 | 1.67e-04 |
| ENSG00000124225 | 1.40e-14 | 2.68e-13 |
| ENSG00000124226 | 3.18e-16 | 8.50e-15 |
| ENSG00000124228 | 7.76e-12 | 8.84e-11 |
| ENSG00000124232 | 1.54e-01 | 2.17e-01 |
| ENSG00000124233 | 2.06e-01 | 2.78e-01 |
| ENSG00000124237 | 1.54e-03 | 3.89e-03 |
| ENSG00000124243 | 1.94e-04 | 5.97e-04 |
| ENSG00000124249 | 1.34e-02 | 2.63e-02 |
| ENSG00000124251 | 1.53e-02 | 2.96e-02 |
| ENSG00000124253 | 4.12e-16 | 1.07e-14 |
| ENSG00000124256 | 7.46e-02 | 1.17e-01 |
| ENSG00000124257 | 3.70e-04 | 1.07e-03 |
| ENSG00000124260 | 3.57e-04 | 1.04e-03 |
| ENSG00000124275 | 6.09e-02 | 9.82e-02 |
| ENSG00000124279 | 8.37e-01 | 8.77e-01 |
| ENSG00000124299 | 2.19e-01 | 2.92e-01 |
| ENSG00000124302 | 3.54e-10 | 3.02e-09 |
| ENSG00000124313 | 6.29e-10 | 5.13e-09 |
| ENSG00000124333 | 6.27e-01 | 6.98e-01 |
| ENSG00000124334 | 7.39e-04 | 2.01e-03 |
| ENSG00000124343 | 8.53e-01 | 8.89e-01 |
| ENSG00000124356 | 3.39e-01 | 4.21e-01 |
| ENSG00000124357 | 4.35e-02 | 7.36e-02 |
| ENSG00000124370 | 8.05e-02 | 1.25e-01 |
| ENSG00000124374 | 9.88e-08 | 5.57e-07 |
| ENSG00000124380 | 7.27e-01 | 7.85e-01 |
| ENSG00000124383 | 9.51e-07 | 4.52e-06 |
| ENSG00000124391 | 1.64e-07 | 8.90e-07 |
| ENSG00000124399 | 1.06e-01 | 1.58e-01 |
| ENSG00000124406 | 5.20e-12 | 6.13e-11 |
| ENSG00000124422 | 3.28e-01 | 4.09e-01 |
| ENSG00000124429 | 2.49e-01 | 3.25e-01 |
| ENSG00000124440 | 1.87e-09 | 1.41e-08 |
| ENSG00000124444 | 2.79e-02 | 5.00e-02 |
| ENSG00000124449 | 2.49e-01 | 3.25e-01 |
| ENSG00000124459 | 2.69e-03 | 6.41e-03 |
| ENSG00000124466 | 1.34e-07 | 7.38e-07 |
| ENSG00000124467 | 2.57e-01 | 3.34e-01 |
| ENSG00000124469 | 1.19e-02 | 2.38e-02 |
| ENSG00000124479 | 5.11e-02 | 8.44e-02 |
| ENSG00000124486 | 2.31e-01 | 3.05e-01 |
| ENSG00000124490 | 6.20e-01 | 6.91e-01 |
| ENSG00000124491 | 7.20e-16 | 1.77e-14 |
| ENSG00000124493 | 3.59e-01 | 4.41e-01 |
| ENSG00000124496 | 1.09e-02 | 2.19e-02 |
| ENSG00000124507 | 3.13e-15 | 6.79e-14 |
| ENSG00000124508 | 1.36e-01 | 1.95e-01 |
| ENSG00000124523 | 1.95e-01 | 2.65e-01 |
| ENSG00000124532 | 1.33e-03 | 3.42e-03 |
| ENSG00000124535 | 1.23e-10 | 1.13e-09 |
| ENSG00000124541 | 2.63e-05 | 9.63e-05 |
| ENSG00000124549 | 2.18e-09 | 1.62e-08 |
| ENSG00000124557 | 9.33e-05 | 3.06e-04 |
| ENSG00000124562 | 1.06e-08 | 7.00e-08 |
| ENSG00000124564 | 4.26e-06 | 1.80e-05 |
| ENSG00000124568 | 1.39e-11 | 1.50e-10 |
| ENSG00000124570 | 2.15e-01 | 2.87e-01 |
| ENSG00000124571 | 8.05e-22 | 9.39e-20 |
| ENSG00000124574 | 1.32e-12 | 1.75e-11 |
| ENSG00000124575 | 5.89e-01 | 6.63e-01 |
| ENSG00000124587 | 3.70e-01 | 4.53e-01 |
| ENSG00000124588 | 5.88e-14 | 9.93e-13 |
| ENSG00000124593 | 2.20e-01 | 2.93e-01 |
| ENSG00000124596 | 6.43e-02 | 1.03e-01 |
| ENSG00000124602 | 1.35e-21 | 1.49e-19 |
| ENSG00000124608 | 1.64e-03 | 4.11e-03 |
| ENSG00000124610 | 2.32e-01 | 3.07e-01 |
| ENSG00000124613 | 4.63e-04 | 1.32e-03 |
| ENSG00000124614 | 1.15e-01 | 1.69e-01 |
| ENSG00000124615 | 1.68e-14 | 3.16e-13 |
| ENSG00000124635 | 1.43e-03 | 3.65e-03 |
| ENSG00000124641 | 1.17e-03 | 3.04e-03 |
| ENSG00000124657 | 7.61e-03 | 1.60e-02 |
| ENSG00000124659 | 9.59e-04 | 2.54e-03 |
| ENSG00000124664 | 4.55e-07 | 2.28e-06 |
| ENSG00000124678 | 4.60e-07 | 2.30e-06 |
| ENSG00000124688 | 3.35e-02 | 5.86e-02 |
| ENSG00000124701 | 9.28e-03 | 1.91e-02 |
| ENSG00000124702 | 6.33e-01 | 7.03e-01 |
| ENSG00000124713 | 8.15e-06 | 3.28e-05 |
| ENSG00000124721 | 4.32e-01 | 5.15e-01 |
| ENSG00000124731 | 3.41e-14 | 6.02e-13 |
| ENSG00000124733 | 7.31e-04 | 1.99e-03 |
| ENSG00000124743 | 8.18e-16 | 1.99e-14 |
| ENSG00000124749 | 4.21e-08 | 2.54e-07 |
| ENSG00000124762 | 3.91e-13 | 5.70e-12 |
| ENSG00000124766 | 2.47e-21 | 2.47e-19 |
| ENSG00000124767 | 2.28e-08 | 1.43e-07 |
| ENSG00000124772 | 8.98e-16 | 2.16e-14 |
| ENSG00000124780 | 6.97e-02 | 1.10e-01 |
| ENSG00000124782 | 1.57e-02 | 3.02e-02 |
| ENSG00000124783 | 5.77e-05 | 1.97e-04 |
| ENSG00000124784 | 1.28e-16 | 3.71e-15 |
| ENSG00000124785 | 8.07e-01 | 8.52e-01 |
| ENSG00000124786 | 1.33e-07 | 7.37e-07 |
| ENSG00000124787 | 2.78e-16 | 7.53e-15 |
| ENSG00000124788 | 3.33e-02 | 5.82e-02 |
| ENSG00000124789 | 2.05e-04 | 6.28e-04 |
| ENSG00000124795 | 2.43e-05 | 8.94e-05 |
| ENSG00000124802 | 4.64e-15 | 9.68e-14 |
| ENSG00000124813 | 2.32e-01 | 3.06e-01 |
| ENSG00000124818 | 2.29e-03 | 5.55e-03 |
| ENSG00000124827 | 2.19e-03 | 5.32e-03 |
| ENSG00000124831 | 1.10e-01 | 1.63e-01 |
| ENSG00000124835 | 1.36e-02 | 2.66e-02 |
| ENSG00000124839 | 2.49e-01 | 3.25e-01 |
| ENSG00000124875 | 5.33e-11 | 5.23e-10 |
| ENSG00000124882 | 1.42e-06 | 6.50e-06 |
| ENSG00000124915 | 1.01e-07 | 5.67e-07 |
| ENSG00000124920 | 1.12e-01 | 1.66e-01 |
| ENSG00000124935 | 3.95e-08 | 2.39e-07 |
| ENSG00000124939 | 2.39e-12 | 3.02e-11 |
| ENSG00000124942 | 1.67e-14 | 3.15e-13 |
| ENSG00000125037 | 8.47e-06 | 3.40e-05 |
| ENSG00000125046 | 2.59e-01 | 3.36e-01 |
| ENSG00000125084 | 2.44e-04 | 7.35e-04 |
| ENSG00000125089 | 2.13e-06 | 9.46e-06 |
| ENSG00000125107 | 3.22e-02 | 5.66e-02 |
| ENSG00000125122 | 2.18e-01 | 2.90e-01 |
| ENSG00000125124 | 2.20e-02 | 4.06e-02 |
| ENSG00000125144 | 3.74e-20 | 2.55e-18 |
| ENSG00000125148 | 1.11e-18 | 5.18e-17 |
| ENSG00000125149 | 1.14e-10 | 1.05e-09 |
| ENSG00000125166 | 2.54e-03 | 6.08e-03 |
| ENSG00000125170 | 2.15e-08 | 1.36e-07 |
| ENSG00000125207 | 9.97e-17 | 2.96e-15 |
| ENSG00000125245 | 4.47e-08 | 2.68e-07 |
| ENSG00000125246 | 7.69e-12 | 8.77e-11 |
| ENSG00000125247 | 2.13e-15 | 4.73e-14 |
| ENSG00000125249 | 2.08e-08 | 1.32e-07 |
| ENSG00000125255 | 7.07e-17 | 2.16e-15 |
| ENSG00000125257 | 6.88e-01 | 7.52e-01 |
| ENSG00000125266 | 5.25e-03 | 1.16e-02 |
| ENSG00000125285 | 9.14e-02 | 1.39e-01 |
| ENSG00000125304 | 8.04e-01 | 8.49e-01 |
| ENSG00000125319 | 8.51e-16 | 2.06e-14 |
| ENSG00000125337 | 3.21e-03 | 7.48e-03 |
| ENSG00000125347 | 4.85e-01 | 5.66e-01 |
| ENSG00000125351 | 1.80e-10 | 1.62e-09 |
| ENSG00000125352 | 2.35e-07 | 1.24e-06 |
| ENSG00000125354 | 3.22e-09 | 2.33e-08 |
| ENSG00000125355 | 2.52e-16 | 6.91e-15 |
| ENSG00000125356 | 5.13e-08 | 3.05e-07 |
| ENSG00000125363 | 2.02e-13 | 3.10e-12 |
| ENSG00000125375 | 1.74e-09 | 1.32e-08 |
| ENSG00000125378 | 1.94e-12 | 2.49e-11 |
| ENSG00000125384 | 7.06e-08 | 4.08e-07 |
| ENSG00000125385 | 9.08e-02 | 1.38e-01 |
| ENSG00000125386 | 2.13e-02 | 3.94e-02 |
| ENSG00000125388 | 2.95e-02 | 5.24e-02 |
| ENSG00000125398 | 1.88e-21 | 1.94e-19 |
| ENSG00000125409 | 2.73e-03 | 6.48e-03 |
| ENSG00000125414 | 1.00e-04 | 3.27e-04 |
| ENSG00000125430 | 1.49e-01 | 2.12e-01 |
| ENSG00000125434 | 3.02e-09 | 2.20e-08 |
| ENSG00000125445 | 4.94e-01 | 5.75e-01 |
| ENSG00000125447 | 2.67e-03 | 6.36e-03 |
| ENSG00000125449 | 6.72e-07 | 3.28e-06 |
| ENSG00000125450 | 5.99e-16 | 1.51e-14 |
| ENSG00000125454 | 1.06e-07 | 5.95e-07 |
| ENSG00000125457 | 9.40e-03 | 1.93e-02 |
| ENSG00000125458 | 4.32e-01 | 5.15e-01 |
| ENSG00000125459 | 5.63e-13 | 7.95e-12 |
| ENSG00000125462 | 9.08e-03 | 1.87e-02 |
| ENSG00000125482 | 3.81e-03 | 8.71e-03 |
| ENSG00000125484 | 4.08e-09 | 2.91e-08 |
| ENSG00000125485 | 7.77e-25 | 2.32e-22 |
| ENSG00000125492 | 1.68e-01 | 2.33e-01 |
| ENSG00000125498 | 9.17e-01 | 9.38e-01 |
| ENSG00000125503 | 8.69e-09 | 5.85e-08 |
| ENSG00000125505 | 1.85e-01 | 2.53e-01 |
| ENSG00000125508 | 3.52e-09 | 2.53e-08 |
| ENSG00000125510 | 2.87e-06 | 1.25e-05 |
| ENSG00000125514 | 6.26e-01 | 6.96e-01 |
| ENSG00000125520 | 1.51e-02 | 2.92e-02 |
| ENSG00000125531 | 4.33e-13 | 6.27e-12 |
| ENSG00000125534 | 4.76e-01 | 5.58e-01 |
| ENSG00000125538 | 2.51e-08 | 1.57e-07 |
| ENSG00000125551 | 6.56e-01 | 7.24e-01 |
| ENSG00000125571 | 6.54e-01 | 7.22e-01 |
| ENSG00000125611 | 2.26e-02 | 4.16e-02 |
| ENSG00000125618 | 8.40e-01 | 8.79e-01 |
| ENSG00000125629 | 1.46e-02 | 2.83e-02 |
| ENSG00000125630 | 1.86e-21 | 1.93e-19 |
| ENSG00000125631 | 1.47e-02 | 2.86e-02 |
| ENSG00000125633 | 2.62e-01 | 3.40e-01 |
| ENSG00000125637 | 3.14e-01 | 3.95e-01 |
| ENSG00000125648 | 1.51e-25 | 6.40e-23 |
| ENSG00000125650 | 8.53e-02 | 1.31e-01 |
| ENSG00000125651 | 2.79e-02 | 4.99e-02 |
| ENSG00000125652 | 3.38e-02 | 5.90e-02 |
| ENSG00000125656 | 2.88e-06 | 1.25e-05 |
| ENSG00000125657 | 6.56e-14 | 1.10e-12 |
| ENSG00000125675 | 7.58e-16 | 1.86e-14 |
| ENSG00000125676 | 9.43e-07 | 4.48e-06 |
| ENSG00000125686 | 3.69e-03 | 8.48e-03 |
| ENSG00000125691 | 6.99e-07 | 3.40e-06 |
| ENSG00000125703 | 4.91e-03 | 1.09e-02 |
| ENSG00000125726 | 1.94e-05 | 7.25e-05 |
| ENSG00000125730 | 1.93e-02 | 3.62e-02 |
| ENSG00000125731 | 2.44e-02 | 4.44e-02 |
| ENSG00000125733 | 9.23e-01 | 9.43e-01 |
| ENSG00000125734 | 1.41e-05 | 5.44e-05 |
| ENSG00000125735 | 4.14e-02 | 7.03e-02 |
| ENSG00000125740 | 5.02e-05 | 1.74e-04 |
| ENSG00000125741 | 2.59e-01 | 3.37e-01 |
| ENSG00000125743 | 1.03e-09 | 8.15e-09 |
| ENSG00000125744 | 2.20e-01 | 2.93e-01 |
| ENSG00000125746 | 1.62e-01 | 2.26e-01 |
| ENSG00000125753 | 1.35e-06 | 6.21e-06 |
| ENSG00000125755 | 9.44e-09 | 6.31e-08 |
| ENSG00000125772 | 9.88e-12 | 1.11e-10 |
| ENSG00000125775 | 4.39e-22 | 5.50e-20 |
| ENSG00000125779 | 2.70e-02 | 4.85e-02 |
| ENSG00000125780 | 2.77e-02 | 4.97e-02 |
| ENSG00000125787 | 8.81e-03 | 1.83e-02 |
| ENSG00000125788 | 4.28e-01 | 5.11e-01 |
| ENSG00000125798 | 1.26e-11 | 1.37e-10 |
| ENSG00000125804 | 8.47e-03 | 1.76e-02 |
| ENSG00000125810 | 1.67e-01 | 2.33e-01 |
| ENSG00000125812 | 1.30e-11 | 1.41e-10 |
| ENSG00000125813 | 9.02e-01 | 9.27e-01 |
| ENSG00000125814 | 1.66e-01 | 2.32e-01 |
| ENSG00000125817 | 1.89e-02 | 3.56e-02 |
| ENSG00000125818 | 2.55e-05 | 9.36e-05 |
| ENSG00000125820 | 1.26e-06 | 5.84e-06 |
| ENSG00000125821 | 1.13e-13 | 1.81e-12 |
| ENSG00000125823 | 1.05e-11 | 1.17e-10 |
| ENSG00000125826 | 1.41e-13 | 2.22e-12 |
| ENSG00000125827 | 9.96e-01 | 9.98e-01 |
| ENSG00000125831 | 5.27e-02 | 8.67e-02 |
| ENSG00000125834 | 1.04e-06 | 4.90e-06 |
| ENSG00000125835 | 6.93e-12 | 7.98e-11 |
| ENSG00000125841 | 2.04e-01 | 2.75e-01 |
| ENSG00000125843 | 2.90e-01 | 3.70e-01 |
| ENSG00000125844 | 6.28e-01 | 6.99e-01 |
| ENSG00000125845 | 3.46e-18 | 1.42e-16 |
| ENSG00000125846 | 2.12e-08 | 1.34e-07 |
| ENSG00000125848 | 3.59e-02 | 6.23e-02 |
| ENSG00000125850 | 3.67e-02 | 6.34e-02 |
| ENSG00000125851 | 6.68e-25 | 2.07e-22 |
| ENSG00000125863 | 4.87e-01 | 5.68e-01 |
| ENSG00000125864 | 1.35e-18 | 6.16e-17 |
| ENSG00000125868 | 1.28e-03 | 3.29e-03 |
| ENSG00000125869 | 8.27e-02 | 1.28e-01 |
| ENSG00000125870 | 3.42e-06 | 1.47e-05 |
| ENSG00000125871 | 9.89e-12 | 1.11e-10 |
| ENSG00000125872 | 6.43e-11 | 6.23e-10 |
| ENSG00000125875 | 1.47e-01 | 2.09e-01 |
| ENSG00000125877 | 2.99e-08 | 1.84e-07 |
| ENSG00000125878 | 4.26e-01 | 5.09e-01 |
| ENSG00000125879 | 5.76e-01 | 6.51e-01 |
| ENSG00000125885 | 1.81e-13 | 2.80e-12 |
| ENSG00000125888 | 3.85e-02 | 6.61e-02 |
| ENSG00000125895 | 1.60e-16 | 4.56e-15 |
| ENSG00000125898 | 9.58e-08 | 5.41e-07 |
| ENSG00000125900 | 3.17e-01 | 3.99e-01 |
| ENSG00000125901 | 1.92e-06 | 8.59e-06 |
| ENSG00000125910 | 1.28e-03 | 3.31e-03 |
| ENSG00000125912 | 2.60e-06 | 1.14e-05 |
| ENSG00000125931 | 5.85e-09 | 4.06e-08 |
| ENSG00000125944 | 2.53e-05 | 9.30e-05 |
| ENSG00000125945 | 2.74e-03 | 6.52e-03 |
| ENSG00000125952 | 2.57e-04 | 7.72e-04 |
| ENSG00000125954 | 1.97e-01 | 2.67e-01 |
| ENSG00000125962 | 2.41e-08 | 1.51e-07 |
| ENSG00000125965 | 1.74e-02 | 3.30e-02 |
| ENSG00000125966 | 7.37e-02 | 1.16e-01 |
| ENSG00000125967 | 1.16e-02 | 2.32e-02 |
| ENSG00000125968 | 4.24e-02 | 7.19e-02 |
| ENSG00000125970 | 1.84e-03 | 4.56e-03 |
| ENSG00000125971 | 2.13e-08 | 1.35e-07 |
| ENSG00000125975 | 3.92e-01 | 4.75e-01 |
| ENSG00000125977 | 1.95e-18 | 8.49e-17 |
| ENSG00000125991 | 6.38e-08 | 3.72e-07 |
| ENSG00000125995 | 4.51e-04 | 1.28e-03 |
| ENSG00000125997 | 1.55e-03 | 3.92e-03 |
| ENSG00000125998 | 6.37e-01 | 7.07e-01 |
| ENSG00000125999 | 7.85e-02 | 1.22e-01 |
| ENSG00000126001 | 1.93e-12 | 2.48e-11 |
| ENSG00000126003 | 6.54e-12 | 7.55e-11 |
| ENSG00000126005 | 8.15e-02 | 1.26e-01 |
| ENSG00000126010 | 9.12e-07 | 4.35e-06 |
| ENSG00000126012 | 1.39e-07 | 7.66e-07 |
| ENSG00000126016 | 6.18e-08 | 3.61e-07 |
| ENSG00000126062 | 5.19e-04 | 1.46e-03 |
| ENSG00000126067 | 1.08e-12 | 1.45e-11 |
| ENSG00000126070 | 2.16e-01 | 2.89e-01 |
| ENSG00000126088 | 9.51e-02 | 1.44e-01 |
| ENSG00000126091 | 4.34e-06 | 1.83e-05 |
| ENSG00000126106 | 1.48e-05 | 5.66e-05 |
| ENSG00000126107 | 1.08e-11 | 1.20e-10 |
| ENSG00000126214 | 3.08e-04 | 9.09e-04 |
| ENSG00000126215 | 2.30e-09 | 1.71e-08 |
| ENSG00000126216 | 6.01e-15 | 1.23e-13 |
| ENSG00000126217 | 5.54e-06 | 2.29e-05 |
| ENSG00000126218 | 7.79e-04 | 2.11e-03 |
| ENSG00000126226 | 2.97e-19 | 1.59e-17 |
| ENSG00000126231 | 7.67e-08 | 4.41e-07 |
| ENSG00000126233 | 7.21e-04 | 1.97e-03 |
| ENSG00000126243 | 1.09e-01 | 1.62e-01 |
| ENSG00000126246 | 2.43e-02 | 4.42e-02 |
| ENSG00000126247 | 9.24e-01 | 9.43e-01 |
| ENSG00000126249 | 6.63e-22 | 7.93e-20 |
| ENSG00000126251 | 8.85e-01 | 9.15e-01 |
| ENSG00000126254 | 1.80e-02 | 3.41e-02 |
| ENSG00000126259 | 1.93e-01 | 2.63e-01 |
| ENSG00000126261 | 3.98e-15 | 8.46e-14 |
| ENSG00000126262 | 7.91e-03 | 1.66e-02 |
| ENSG00000126264 | 9.35e-05 | 3.06e-04 |
| ENSG00000126266 | 1.38e-06 | 6.34e-06 |
| ENSG00000126267 | 1.70e-02 | 3.24e-02 |
| ENSG00000126337 | 9.99e-03 | 2.04e-02 |
| ENSG00000126351 | 3.97e-01 | 4.81e-01 |
| ENSG00000126353 | 2.36e-05 | 8.70e-05 |
| ENSG00000126368 | 4.03e-08 | 2.43e-07 |
| ENSG00000126391 | 7.26e-01 | 7.84e-01 |
| ENSG00000126432 | 7.13e-01 | 7.73e-01 |
| ENSG00000126453 | 2.33e-13 | 3.54e-12 |
| ENSG00000126456 | 3.20e-05 | 1.15e-04 |
| ENSG00000126457 | 1.41e-10 | 1.28e-09 |
| ENSG00000126458 | 1.80e-01 | 2.47e-01 |
| ENSG00000126460 | 1.03e-05 | 4.07e-05 |
| ENSG00000126461 | 5.80e-02 | 9.41e-02 |
| ENSG00000126464 | 2.92e-03 | 6.88e-03 |
| ENSG00000126467 | 3.29e-07 | 1.69e-06 |
| ENSG00000126500 | 4.00e-03 | 9.10e-03 |
| ENSG00000126522 | 8.28e-02 | 1.28e-01 |
| ENSG00000126524 | 5.64e-04 | 1.58e-03 |
| ENSG00000126545 | 8.57e-03 | 1.78e-02 |
| ENSG00000126550 | 3.83e-04 | 1.11e-03 |
| ENSG00000126561 | 7.19e-01 | 7.78e-01 |
| ENSG00000126562 | 3.45e-01 | 4.27e-01 |
| ENSG00000126581 | 2.83e-03 | 6.69e-03 |
| ENSG00000126583 | 3.18e-08 | 1.95e-07 |
| ENSG00000126602 | 1.45e-18 | 6.52e-17 |
| ENSG00000126603 | 4.39e-03 | 9.88e-03 |
| ENSG00000126653 | 1.92e-01 | 2.62e-01 |
| ENSG00000126698 | 3.83e-01 | 4.67e-01 |
| ENSG00000126705 | 7.43e-02 | 1.17e-01 |
| ENSG00000126709 | 1.59e-09 | 1.21e-08 |
| ENSG00000126733 | 2.26e-06 | 1.00e-05 |
| ENSG00000126746 | 1.76e-03 | 4.39e-03 |
| ENSG00000126749 | 1.14e-11 | 1.26e-10 |
| ENSG00000126756 | 1.48e-03 | 3.76e-03 |
| ENSG00000126759 | 1.03e-10 | 9.60e-10 |
| ENSG00000126767 | 1.77e-13 | 2.75e-12 |
| ENSG00000126768 | 1.07e-09 | 8.38e-09 |
| ENSG00000126773 | 2.93e-01 | 3.73e-01 |
| ENSG00000126775 | 9.64e-02 | 1.46e-01 |
| ENSG00000126777 | 3.72e-09 | 2.67e-08 |
| ENSG00000126778 | 1.07e-17 | 3.90e-16 |
| ENSG00000126785 | 2.36e-04 | 7.14e-04 |
| ENSG00000126787 | 1.41e-09 | 1.09e-08 |
| ENSG00000126790 | 2.43e-10 | 2.14e-09 |
| ENSG00000126803 | 4.42e-11 | 4.41e-10 |
| ENSG00000126804 | 2.90e-02 | 5.17e-02 |
| ENSG00000126814 | 1.59e-06 | 7.21e-06 |
| ENSG00000126821 | 4.95e-09 | 3.48e-08 |
| ENSG00000126822 | 2.68e-01 | 3.46e-01 |
| ENSG00000126838 | 4.41e-03 | 9.91e-03 |
| ENSG00000126856 | 1.63e-02 | 3.13e-02 |
| ENSG00000126858 | 1.06e-02 | 2.15e-02 |
| ENSG00000126860 | 1.70e-08 | 1.09e-07 |
| ENSG00000126861 | 1.84e-05 | 6.94e-05 |
| ENSG00000126870 | 4.30e-07 | 2.17e-06 |
| ENSG00000126878 | 3.31e-05 | 1.19e-04 |
| ENSG00000126882 | 9.11e-07 | 4.35e-06 |
| ENSG00000126883 | 7.75e-03 | 1.63e-02 |
| ENSG00000126890 | 3.38e-01 | 4.20e-01 |
| ENSG00000126895 | 7.44e-06 | 3.01e-05 |
| ENSG00000126903 | 8.36e-04 | 2.25e-03 |
| ENSG00000126934 | 2.92e-03 | 6.88e-03 |
| ENSG00000126945 | 1.80e-03 | 4.48e-03 |
| ENSG00000126947 | 5.35e-13 | 7.60e-12 |
| ENSG00000126950 | 4.14e-15 | 8.75e-14 |
| ENSG00000126952 | 1.06e-01 | 1.58e-01 |
| ENSG00000126953 | 1.32e-13 | 2.09e-12 |
| ENSG00000126970 | 3.21e-01 | 4.02e-01 |
| ENSG00000127022 | 1.07e-05 | 4.22e-05 |
| ENSG00000127054 | 8.30e-04 | 2.24e-03 |
| ENSG00000127074 | 6.53e-16 | 1.63e-14 |
| ENSG00000127080 | 2.22e-04 | 6.76e-04 |
| ENSG00000127081 | 8.85e-02 | 1.36e-01 |
| ENSG00000127083 | 1.94e-12 | 2.49e-11 |
| ENSG00000127084 | 6.09e-01 | 6.81e-01 |
| ENSG00000127124 | 4.02e-01 | 4.86e-01 |
| ENSG00000127125 | 7.04e-07 | 3.42e-06 |
| ENSG00000127129 | 1.38e-11 | 1.49e-10 |
| ENSG00000127152 | 2.03e-04 | 6.21e-04 |
| ENSG00000127184 | 2.31e-10 | 2.04e-09 |
| ENSG00000127191 | 1.26e-10 | 1.16e-09 |
| ENSG00000127220 | 5.64e-05 | 1.93e-04 |
| ENSG00000127241 | 2.86e-10 | 2.48e-09 |
| ENSG00000127249 | 3.15e-13 | 4.67e-12 |
| ENSG00000127252 | 3.33e-01 | 4.14e-01 |
| ENSG00000127311 | 3.24e-07 | 1.67e-06 |
| ENSG00000127314 | 1.24e-03 | 3.20e-03 |
| ENSG00000127318 | 8.00e-02 | 1.24e-01 |
| ENSG00000127324 | 5.04e-06 | 2.10e-05 |
| ENSG00000127325 | 3.90e-08 | 2.36e-07 |
| ENSG00000127328 | 3.87e-02 | 6.64e-02 |
| ENSG00000127329 | 4.45e-02 | 7.49e-02 |
| ENSG00000127334 | 9.50e-10 | 7.53e-09 |
| ENSG00000127337 | 3.35e-09 | 2.42e-08 |
| ENSG00000127362 | 1.99e-02 | 3.72e-02 |
| ENSG00000127364 | 3.36e-03 | 7.80e-03 |
| ENSG00000127366 | 2.48e-07 | 1.31e-06 |
| ENSG00000127377 | 3.86e-01 | 4.69e-01 |
| ENSG00000127399 | 8.97e-13 | 1.22e-11 |
| ENSG00000127412 | 5.31e-02 | 8.72e-02 |
| ENSG00000127415 | 8.56e-04 | 2.30e-03 |
| ENSG00000127418 | 6.88e-16 | 1.71e-14 |
| ENSG00000127419 | 6.66e-02 | 1.06e-01 |
| ENSG00000127423 | 5.45e-20 | 3.49e-18 |
| ENSG00000127445 | 9.65e-01 | 9.74e-01 |
| ENSG00000127452 | 6.85e-01 | 7.49e-01 |
| ENSG00000127463 | 5.43e-07 | 2.68e-06 |
| ENSG00000127472 | 8.94e-08 | 5.09e-07 |
| ENSG00000127481 | 8.71e-01 | 9.04e-01 |
| ENSG00000127483 | 6.70e-03 | 1.43e-02 |
| ENSG00000127507 | 2.00e-07 | 1.07e-06 |
| ENSG00000127511 | 3.98e-07 | 2.01e-06 |
| ENSG00000127526 | 2.70e-03 | 6.44e-03 |
| ENSG00000127527 | 1.47e-02 | 2.86e-02 |
| ENSG00000127528 | 1.49e-05 | 5.70e-05 |
| ENSG00000127530 | 6.02e-02 | 9.73e-02 |
| ENSG00000127533 | 9.17e-01 | 9.39e-01 |
| ENSG00000127540 | 3.41e-02 | 5.96e-02 |
| ENSG00000127554 | 4.95e-03 | 1.10e-02 |
| ENSG00000127561 | 3.90e-01 | 4.73e-01 |
| ENSG00000127564 | 1.33e-18 | 6.10e-17 |
| ENSG00000127578 | 1.91e-01 | 2.61e-01 |
| ENSG00000127580 | 8.45e-09 | 5.70e-08 |
| ENSG00000127585 | 1.13e-02 | 2.26e-02 |
| ENSG00000127586 | 7.67e-17 | 2.32e-15 |
| ENSG00000127588 | 5.66e-16 | 1.43e-14 |
| ENSG00000127589 | 1.00e-07 | 5.65e-07 |
| ENSG00000127603 | 6.72e-01 | 7.38e-01 |
| ENSG00000127616 | 9.63e-12 | 1.08e-10 |
| ENSG00000127663 | 7.67e-01 | 8.19e-01 |
| ENSG00000127666 | 3.17e-02 | 5.58e-02 |
| ENSG00000127720 | 8.51e-07 | 4.08e-06 |
| ENSG00000127743 | 1.70e-04 | 5.31e-04 |
| ENSG00000127774 | 7.05e-01 | 7.67e-01 |
| ENSG00000127804 | 2.59e-04 | 7.77e-04 |
| ENSG00000127824 | 1.96e-07 | 1.05e-06 |
| ENSG00000127831 | 4.17e-01 | 5.00e-01 |
| ENSG00000127837 | 2.26e-01 | 3.00e-01 |
| ENSG00000127838 | 8.01e-01 | 8.47e-01 |
| ENSG00000127863 | 5.13e-01 | 5.93e-01 |
| ENSG00000127870 | 2.91e-04 | 8.62e-04 |
| ENSG00000127884 | 1.61e-05 | 6.11e-05 |
| ENSG00000127903 | 6.46e-13 | 9.01e-12 |
| ENSG00000127914 | 1.50e-08 | 9.74e-08 |
| ENSG00000127920 | 1.19e-06 | 5.55e-06 |
| ENSG00000127922 | 5.94e-13 | 8.34e-12 |
| ENSG00000127928 | 4.19e-11 | 4.20e-10 |
| ENSG00000127946 | 7.04e-06 | 2.86e-05 |
| ENSG00000127947 | 1.92e-10 | 1.71e-09 |
| ENSG00000127948 | 6.38e-03 | 1.37e-02 |
| ENSG00000127951 | 3.60e-23 | 6.17e-21 |
| ENSG00000127952 | 4.31e-03 | 9.72e-03 |
| ENSG00000127954 | 5.30e-05 | 1.82e-04 |
| ENSG00000127955 | 1.22e-13 | 1.95e-12 |
| ENSG00000127957 | 2.48e-13 | 3.75e-12 |
| ENSG00000127980 | 3.35e-02 | 5.86e-02 |
| ENSG00000127989 | 4.98e-03 | 1.10e-02 |
| ENSG00000127990 | 3.04e-12 | 3.76e-11 |
| ENSG00000127993 | 2.92e-04 | 8.65e-04 |
| ENSG00000127995 | 7.31e-13 | 1.01e-11 |
| ENSG00000128000 | 5.85e-01 | 6.60e-01 |
| ENSG00000128011 | 6.95e-09 | 4.76e-08 |
| ENSG00000128016 | 1.06e-03 | 2.79e-03 |
| ENSG00000128039 | 5.24e-10 | 4.34e-09 |
| ENSG00000128040 | 2.94e-19 | 1.58e-17 |
| ENSG00000128045 | 6.29e-04 | 1.74e-03 |
| ENSG00000128050 | 1.63e-18 | 7.22e-17 |
| ENSG00000128052 | 5.53e-03 | 1.21e-02 |
| ENSG00000128059 | 9.84e-21 | 8.08e-19 |
| ENSG00000128159 | 2.33e-03 | 5.64e-03 |
| ENSG00000128165 | 1.32e-04 | 4.20e-04 |
| ENSG00000128185 | 1.07e-02 | 2.17e-02 |
| ENSG00000128191 | 1.95e-02 | 3.65e-02 |
| ENSG00000128203 | 8.39e-01 | 8.79e-01 |
| ENSG00000128218 | 8.21e-10 | 6.58e-09 |
| ENSG00000128228 | 1.09e-02 | 2.20e-02 |
| ENSG00000128242 | 5.65e-04 | 1.58e-03 |
| ENSG00000128245 | 9.09e-01 | 9.32e-01 |
| ENSG00000128253 | 6.39e-01 | 7.09e-01 |
| ENSG00000128254 | 3.83e-01 | 4.67e-01 |
| ENSG00000128262 | 5.13e-02 | 8.47e-02 |
| ENSG00000128266 | 2.70e-03 | 6.43e-03 |
| ENSG00000128268 | 3.03e-03 | 7.12e-03 |
| ENSG00000128271 | 6.13e-05 | 2.08e-04 |
| ENSG00000128272 | 2.24e-03 | 5.44e-03 |
| ENSG00000128274 | 1.87e-02 | 3.53e-02 |
| ENSG00000128276 | 5.72e-01 | 6.48e-01 |
| ENSG00000128283 | 2.52e-07 | 1.32e-06 |
| ENSG00000128284 | 3.69e-03 | 8.49e-03 |
| ENSG00000128285 | 7.87e-01 | 8.35e-01 |
| ENSG00000128294 | 5.93e-07 | 2.91e-06 |
| ENSG00000128298 | 6.61e-02 | 1.05e-01 |
| ENSG00000128309 | 8.37e-02 | 1.29e-01 |
| ENSG00000128310 | 9.66e-02 | 1.46e-01 |
| ENSG00000128311 | 3.66e-14 | 6.42e-13 |
| ENSG00000128313 | 1.36e-01 | 1.96e-01 |
| ENSG00000128322 | 6.07e-06 | 2.49e-05 |
| ENSG00000128335 | 5.70e-01 | 6.46e-01 |
| ENSG00000128340 | 1.52e-04 | 4.77e-04 |
| ENSG00000128342 | 1.33e-15 | 3.09e-14 |
| ENSG00000128346 | 5.81e-04 | 1.62e-03 |
| ENSG00000128383 | 2.30e-05 | 8.51e-05 |
| ENSG00000128394 | 1.24e-03 | 3.22e-03 |
| ENSG00000128408 | 6.63e-06 | 2.70e-05 |
| ENSG00000128422 | 4.04e-18 | 1.63e-16 |
| ENSG00000128438 | 8.46e-08 | 4.83e-07 |
| ENSG00000128463 | 8.90e-02 | 1.36e-01 |
| ENSG00000128482 | 2.58e-21 | 2.55e-19 |
| ENSG00000128487 | 6.89e-15 | 1.39e-13 |
| ENSG00000128510 | 3.24e-15 | 7.02e-14 |
| ENSG00000128512 | 1.14e-01 | 1.68e-01 |
| ENSG00000128513 | 2.52e-10 | 2.21e-09 |
| ENSG00000128524 | 4.59e-14 | 7.91e-13 |
| ENSG00000128534 | 1.47e-14 | 2.81e-13 |
| ENSG00000128536 | 3.66e-02 | 6.33e-02 |
| ENSG00000128563 | 7.62e-12 | 8.70e-11 |
| ENSG00000128564 | 6.52e-09 | 4.49e-08 |
| ENSG00000128567 | 1.07e-13 | 1.73e-12 |
| ENSG00000128578 | 1.46e-20 | 1.12e-18 |
| ENSG00000128581 | 4.75e-11 | 4.72e-10 |
| ENSG00000128585 | 1.11e-02 | 2.23e-02 |
| ENSG00000128590 | 3.26e-01 | 4.08e-01 |
| ENSG00000128591 | 3.77e-06 | 1.60e-05 |
| ENSG00000128594 | 3.80e-02 | 6.54e-02 |
| ENSG00000128595 | 2.82e-13 | 4.22e-12 |
| ENSG00000128596 | 3.26e-03 | 7.60e-03 |
| ENSG00000128602 | 1.33e-01 | 1.92e-01 |
| ENSG00000128604 | 6.14e-01 | 6.86e-01 |
| ENSG00000128606 | 1.29e-05 | 5.02e-05 |
| ENSG00000128607 | 2.51e-03 | 6.02e-03 |
| ENSG00000128609 | 2.90e-08 | 1.79e-07 |
| ENSG00000128610 | 1.99e-25 | 7.86e-23 |
| ENSG00000128617 | 2.90e-01 | 3.70e-01 |
| ENSG00000128626 | 1.45e-07 | 7.95e-07 |
| ENSG00000128641 | 5.60e-05 | 1.92e-04 |
| ENSG00000128645 | 4.32e-16 | 1.12e-14 |
| ENSG00000128652 | 8.15e-08 | 4.66e-07 |
| ENSG00000128654 | 4.39e-01 | 5.22e-01 |
| ENSG00000128655 | 1.21e-02 | 2.41e-02 |
| ENSG00000128656 | 2.59e-05 | 9.48e-05 |
| ENSG00000128683 | 8.74e-21 | 7.32e-19 |
| ENSG00000128692 | 1.94e-14 | 3.61e-13 |
| ENSG00000128694 | 2.48e-05 | 9.11e-05 |
| ENSG00000128699 | 3.65e-05 | 1.30e-04 |
| ENSG00000128708 | 1.62e-03 | 4.08e-03 |
| ENSG00000128709 | 7.12e-01 | 7.72e-01 |
| ENSG00000128710 | 4.11e-05 | 1.45e-04 |
| ENSG00000128713 | 6.04e-03 | 1.31e-02 |
| ENSG00000128714 | 5.94e-01 | 6.68e-01 |
| ENSG00000128731 | 3.05e-01 | 3.86e-01 |
| ENSG00000128739 | 2.23e-07 | 1.18e-06 |
| ENSG00000128789 | 4.31e-02 | 7.29e-02 |
| ENSG00000128791 | 8.61e-01 | 8.96e-01 |
| ENSG00000128805 | 6.84e-01 | 7.49e-01 |
| ENSG00000128815 | 6.17e-06 | 2.53e-05 |
| ENSG00000128829 | 1.53e-03 | 3.88e-03 |
| ENSG00000128833 | 1.14e-01 | 1.68e-01 |
| ENSG00000128849 | 5.59e-03 | 1.22e-02 |
| ENSG00000128872 | 7.24e-08 | 4.18e-07 |
| ENSG00000128881 | 1.34e-04 | 4.26e-04 |
| ENSG00000128886 | 4.93e-02 | 8.20e-02 |
| ENSG00000128891 | 2.55e-05 | 9.35e-05 |
| ENSG00000128908 | 3.72e-01 | 4.54e-01 |
| ENSG00000128915 | 1.57e-03 | 3.96e-03 |
| ENSG00000128917 | 3.04e-07 | 1.58e-06 |
| ENSG00000128918 | 9.30e-02 | 1.41e-01 |
| ENSG00000128923 | 1.40e-02 | 2.74e-02 |
| ENSG00000128928 | 1.06e-03 | 2.79e-03 |
| ENSG00000128944 | 2.25e-13 | 3.43e-12 |
| ENSG00000128951 | 1.09e-01 | 1.63e-01 |
| ENSG00000128965 | 1.27e-13 | 2.02e-12 |
| ENSG00000128973 | 2.99e-09 | 2.18e-08 |
| ENSG00000128989 | 7.08e-01 | 7.69e-01 |
| ENSG00000129003 | 5.48e-01 | 6.26e-01 |
| ENSG00000129007 | 2.54e-09 | 1.87e-08 |
| ENSG00000129009 | 3.09e-03 | 7.23e-03 |
| ENSG00000129028 | 8.62e-05 | 2.84e-04 |
| ENSG00000129038 | 1.54e-06 | 7.02e-06 |
| ENSG00000129048 | 1.37e-06 | 6.28e-06 |
| ENSG00000129055 | 7.44e-08 | 4.28e-07 |
| ENSG00000129071 | 7.36e-01 | 7.93e-01 |
| ENSG00000129083 | 2.01e-01 | 2.72e-01 |
| ENSG00000129084 | 4.79e-07 | 2.39e-06 |
| ENSG00000129103 | 9.12e-07 | 4.35e-06 |
| ENSG00000129116 | 8.58e-04 | 2.30e-03 |
| ENSG00000129128 | 5.82e-01 | 6.57e-01 |
| ENSG00000129151 | 8.20e-03 | 1.72e-02 |
| ENSG00000129152 | 2.11e-01 | 2.83e-01 |
| ENSG00000129158 | 1.76e-03 | 4.39e-03 |
| ENSG00000129159 | 2.05e-10 | 1.83e-09 |
| ENSG00000129167 | 4.00e-17 | 1.30e-15 |
| ENSG00000129170 | 5.13e-01 | 5.92e-01 |
| ENSG00000129173 | 2.29e-07 | 1.21e-06 |
| ENSG00000129187 | 4.78e-18 | 1.89e-16 |
| ENSG00000129194 | 2.25e-07 | 1.19e-06 |
| ENSG00000129195 | 3.76e-10 | 3.20e-09 |
| ENSG00000129197 | 8.32e-06 | 3.34e-05 |
| ENSG00000129204 | 4.58e-01 | 5.41e-01 |
| ENSG00000129214 | 7.08e-01 | 7.69e-01 |
| ENSG00000129219 | 2.03e-01 | 2.74e-01 |
| ENSG00000129221 | 8.35e-01 | 8.75e-01 |
| ENSG00000129226 | 1.65e-02 | 3.15e-02 |
| ENSG00000129235 | 1.78e-02 | 3.38e-02 |
| ENSG00000129244 | 5.73e-21 | 4.99e-19 |
| ENSG00000129245 | 7.59e-03 | 1.60e-02 |
| ENSG00000129250 | 2.64e-16 | 7.21e-15 |
| ENSG00000129255 | 9.04e-08 | 5.14e-07 |
| ENSG00000129292 | 1.84e-05 | 6.93e-05 |
| ENSG00000129295 | 1.40e-08 | 9.11e-08 |
| ENSG00000129315 | 6.85e-05 | 2.31e-04 |
| ENSG00000129317 | 1.17e-07 | 6.52e-07 |
| ENSG00000129347 | 2.59e-10 | 2.26e-09 |
| ENSG00000129351 | 1.59e-16 | 4.53e-15 |
| ENSG00000129353 | 6.78e-08 | 3.94e-07 |
| ENSG00000129354 | 3.76e-01 | 4.59e-01 |
| ENSG00000129355 | 9.38e-01 | 9.54e-01 |
| ENSG00000129422 | 1.58e-09 | 1.20e-08 |
| ENSG00000129437 | 8.40e-01 | 8.79e-01 |
| ENSG00000129450 | 2.82e-01 | 3.62e-01 |
| ENSG00000129451 | 7.29e-13 | 1.01e-11 |
| ENSG00000129455 | 7.64e-28 | 7.44e-25 |
| ENSG00000129460 | 2.26e-04 | 6.87e-04 |
| ENSG00000129465 | 6.54e-09 | 4.50e-08 |
| ENSG00000129467 | 2.13e-02 | 3.95e-02 |
| ENSG00000129472 | 5.53e-01 | 6.31e-01 |
| ENSG00000129473 | 4.40e-10 | 3.70e-09 |
| ENSG00000129474 | 8.90e-32 | 2.66e-28 |
| ENSG00000129480 | 6.53e-04 | 1.80e-03 |
| ENSG00000129484 | 2.00e-07 | 1.07e-06 |
| ENSG00000129493 | 7.18e-11 | 6.90e-10 |
| ENSG00000129514 | 5.06e-07 | 2.52e-06 |
| ENSG00000129515 | 2.94e-07 | 1.53e-06 |
| ENSG00000129518 | 1.01e-11 | 1.13e-10 |
| ENSG00000129521 | 3.03e-07 | 1.57e-06 |
| ENSG00000129534 | 5.27e-02 | 8.67e-02 |
| ENSG00000129535 | 1.32e-01 | 1.90e-01 |
| ENSG00000129538 | 3.38e-11 | 3.44e-10 |
| ENSG00000129559 | 1.61e-01 | 2.25e-01 |
| ENSG00000129562 | 7.42e-01 | 7.98e-01 |
| ENSG00000129566 | 1.72e-10 | 1.55e-09 |
| ENSG00000129595 | 1.87e-13 | 2.88e-12 |
| ENSG00000129596 | 8.35e-12 | 9.47e-11 |
| ENSG00000129625 | 2.42e-02 | 4.41e-02 |
| ENSG00000129636 | 6.27e-07 | 3.07e-06 |
| ENSG00000129646 | 7.96e-04 | 2.15e-03 |
| ENSG00000129654 | 8.38e-01 | 8.77e-01 |
| ENSG00000129657 | 8.22e-01 | 8.64e-01 |
| ENSG00000129667 | 5.91e-17 | 1.84e-15 |
| ENSG00000129673 | 6.86e-06 | 2.79e-05 |
| ENSG00000129675 | 1.03e-10 | 9.63e-10 |
| ENSG00000129680 | 5.65e-02 | 9.20e-02 |
| ENSG00000129682 | 1.11e-06 | 5.19e-06 |
| ENSG00000129691 | 1.37e-02 | 2.68e-02 |
| ENSG00000129696 | 5.61e-02 | 9.15e-02 |
| ENSG00000129744 | 2.18e-03 | 5.31e-03 |
| ENSG00000129749 | 2.34e-02 | 4.29e-02 |
| ENSG00000129757 | 6.39e-01 | 7.08e-01 |
| ENSG00000129810 | 7.17e-12 | 8.22e-11 |
| ENSG00000129824 | 9.31e-01 | 9.49e-01 |
| ENSG00000129910 | 2.53e-05 | 9.30e-05 |
| ENSG00000129911 | 7.02e-07 | 3.41e-06 |
| ENSG00000129925 | 9.67e-01 | 9.76e-01 |
| ENSG00000129932 | 7.72e-05 | 2.57e-04 |
| ENSG00000129933 | 1.50e-03 | 3.80e-03 |
| ENSG00000129946 | 3.88e-03 | 8.87e-03 |
| ENSG00000129951 | 2.89e-01 | 3.69e-01 |
| ENSG00000129968 | 1.15e-01 | 1.70e-01 |
| ENSG00000129988 | 1.33e-05 | 5.16e-05 |
| ENSG00000129990 | 2.02e-14 | 3.75e-13 |
| ENSG00000129991 | 4.05e-14 | 7.07e-13 |
| ENSG00000129993 | 6.52e-08 | 3.80e-07 |
| ENSG00000130005 | 7.74e-07 | 3.73e-06 |
| ENSG00000130021 | 8.75e-01 | 9.07e-01 |
| ENSG00000130023 | 3.75e-08 | 2.27e-07 |
| ENSG00000130024 | 5.32e-02 | 8.75e-02 |
| ENSG00000130032 | 1.37e-07 | 7.56e-07 |
| ENSG00000130035 | 4.06e-05 | 1.43e-04 |
| ENSG00000130037 | 1.07e-08 | 7.09e-08 |
| ENSG00000130038 | 9.71e-02 | 1.47e-01 |
| ENSG00000130045 | 7.30e-01 | 7.88e-01 |
| ENSG00000130052 | 1.87e-04 | 5.77e-04 |
| ENSG00000130054 | 5.78e-02 | 9.39e-02 |
| ENSG00000130055 | 7.06e-22 | 8.35e-20 |
| ENSG00000130066 | 8.93e-02 | 1.37e-01 |
| ENSG00000130119 | 1.65e-16 | 4.68e-15 |
| ENSG00000130147 | 3.96e-13 | 5.77e-12 |
| ENSG00000130150 | 2.60e-03 | 6.22e-03 |
| ENSG00000130158 | 1.45e-16 | 4.15e-15 |
| ENSG00000130159 | 2.66e-03 | 6.34e-03 |
| ENSG00000130164 | 1.54e-07 | 8.40e-07 |
| ENSG00000130165 | 2.58e-03 | 6.18e-03 |
| ENSG00000130167 | 3.86e-02 | 6.63e-02 |
| ENSG00000130173 | 1.19e-02 | 2.38e-02 |
| ENSG00000130175 | 4.41e-02 | 7.43e-02 |
| ENSG00000130176 | 8.49e-08 | 4.84e-07 |
| ENSG00000130177 | 7.20e-14 | 1.20e-12 |
| ENSG00000130182 | 1.84e-02 | 3.48e-02 |
| ENSG00000130193 | 1.74e-12 | 2.26e-11 |
| ENSG00000130202 | 1.63e-03 | 4.09e-03 |
| ENSG00000130203 | 4.62e-02 | 7.74e-02 |
| ENSG00000130204 | 2.57e-10 | 2.26e-09 |
| ENSG00000130208 | 1.64e-01 | 2.29e-01 |
| ENSG00000130222 | 1.52e-02 | 2.93e-02 |
| ENSG00000130224 | 3.03e-10 | 2.62e-09 |
| ENSG00000130226 | 4.27e-15 | 8.97e-14 |
| ENSG00000130227 | 1.73e-02 | 3.28e-02 |
| ENSG00000130234 | 2.71e-02 | 4.87e-02 |
| ENSG00000130244 | 4.84e-01 | 5.66e-01 |
| ENSG00000130254 | 1.91e-02 | 3.59e-02 |
| ENSG00000130255 | 3.46e-09 | 2.50e-08 |
| ENSG00000130270 | 5.53e-03 | 1.21e-02 |
| ENSG00000130287 | 4.69e-11 | 4.66e-10 |
| ENSG00000130294 | 3.70e-19 | 1.95e-17 |
| ENSG00000130299 | 2.45e-17 | 8.33e-16 |
| ENSG00000130300 | 2.08e-01 | 2.79e-01 |
| ENSG00000130303 | 4.74e-01 | 5.57e-01 |
| ENSG00000130304 | 1.07e-05 | 4.22e-05 |
| ENSG00000130305 | 1.26e-14 | 2.42e-13 |
| ENSG00000130307 | 2.60e-03 | 6.22e-03 |
| ENSG00000130309 | 3.57e-17 | 1.17e-15 |
| ENSG00000130311 | 3.38e-01 | 4.20e-01 |
| ENSG00000130312 | 3.08e-04 | 9.08e-04 |
| ENSG00000130313 | 8.38e-03 | 1.75e-02 |
| ENSG00000130332 | 1.63e-11 | 1.74e-10 |
| ENSG00000130338 | 1.68e-08 | 1.08e-07 |
| ENSG00000130340 | 2.70e-07 | 1.41e-06 |
| ENSG00000130347 | 1.35e-02 | 2.64e-02 |
| ENSG00000130348 | 1.62e-02 | 3.10e-02 |
| ENSG00000130349 | 2.02e-06 | 8.99e-06 |
| ENSG00000130363 | 6.38e-06 | 2.61e-05 |
| ENSG00000130368 | 4.27e-05 | 1.50e-04 |
| ENSG00000130377 | 2.57e-02 | 4.65e-02 |
| ENSG00000130382 | 6.28e-10 | 5.12e-09 |
| ENSG00000130385 | 2.79e-01 | 3.58e-01 |
| ENSG00000130396 | 7.91e-01 | 8.39e-01 |
| ENSG00000130402 | 4.28e-01 | 5.11e-01 |
| ENSG00000130413 | 1.49e-06 | 6.82e-06 |
| ENSG00000130414 | 2.72e-05 | 9.92e-05 |
| ENSG00000130427 | 4.76e-01 | 5.58e-01 |
| ENSG00000130429 | 1.55e-05 | 5.90e-05 |
| ENSG00000130433 | 5.93e-08 | 3.48e-07 |
| ENSG00000130449 | 4.75e-13 | 6.83e-12 |
| ENSG00000130475 | 5.88e-10 | 4.83e-09 |
| ENSG00000130477 | 1.54e-02 | 2.97e-02 |
| ENSG00000130479 | 5.29e-05 | 1.82e-04 |
| ENSG00000130487 | 7.77e-01 | 8.27e-01 |
| ENSG00000130489 | 1.99e-01 | 2.70e-01 |
| ENSG00000130508 | 1.85e-05 | 6.97e-05 |
| ENSG00000130511 | 8.36e-12 | 9.48e-11 |
| ENSG00000130513 | 8.63e-16 | 2.08e-14 |
| ENSG00000130517 | 1.72e-05 | 6.50e-05 |
| ENSG00000130518 | 2.19e-15 | 4.86e-14 |
| ENSG00000130520 | 4.02e-09 | 2.87e-08 |
| ENSG00000130522 | 3.44e-04 | 1.01e-03 |
| ENSG00000130528 | 2.94e-05 | 1.07e-04 |
| ENSG00000130529 | 1.14e-09 | 8.91e-09 |
| ENSG00000130540 | 2.02e-08 | 1.28e-07 |
| ENSG00000130544 | 5.31e-04 | 1.49e-03 |
| ENSG00000130545 | 1.20e-01 | 1.75e-01 |
| ENSG00000130558 | 1.16e-13 | 1.86e-12 |
| ENSG00000130559 | 9.45e-14 | 1.54e-12 |
| ENSG00000130560 | 3.65e-05 | 1.30e-04 |
| ENSG00000130561 | 1.29e-03 | 3.34e-03 |
| ENSG00000130584 | 5.24e-01 | 6.04e-01 |
| ENSG00000130589 | 4.90e-13 | 7.01e-12 |
| ENSG00000130590 | 1.57e-17 | 5.54e-16 |
| ENSG00000130592 | 7.70e-14 | 1.27e-12 |
| ENSG00000130595 | 2.30e-17 | 7.88e-16 |
| ENSG00000130598 | 4.10e-02 | 6.97e-02 |
| ENSG00000130600 | 9.33e-10 | 7.41e-09 |
| ENSG00000130612 | 2.73e-01 | 3.51e-01 |
| ENSG00000130635 | 1.26e-13 | 2.00e-12 |
| ENSG00000130638 | 6.41e-03 | 1.38e-02 |
| ENSG00000130640 | 5.29e-01 | 6.08e-01 |
| ENSG00000130643 | 1.33e-12 | 1.76e-11 |
| ENSG00000130649 | 1.27e-02 | 2.51e-02 |
| ENSG00000130653 | 6.07e-07 | 2.98e-06 |
| ENSG00000130669 | 1.81e-01 | 2.49e-01 |
| ENSG00000130675 | 4.06e-10 | 3.43e-09 |
| ENSG00000130684 | 9.33e-07 | 4.44e-06 |
| ENSG00000130695 | 2.43e-09 | 1.80e-08 |
| ENSG00000130699 | 2.27e-11 | 2.38e-10 |
| ENSG00000130700 | 4.58e-01 | 5.40e-01 |
| ENSG00000130701 | 4.16e-08 | 2.51e-07 |
| ENSG00000130702 | 3.22e-03 | 7.51e-03 |
| ENSG00000130703 | 1.86e-04 | 5.74e-04 |
| ENSG00000130706 | 3.17e-11 | 3.24e-10 |
| ENSG00000130707 | 2.27e-01 | 3.01e-01 |
| ENSG00000130711 | 2.55e-17 | 8.61e-16 |
| ENSG00000130713 | 2.86e-23 | 5.12e-21 |
| ENSG00000130714 | 3.56e-02 | 6.18e-02 |
| ENSG00000130717 | 8.11e-02 | 1.26e-01 |
| ENSG00000130720 | 5.17e-03 | 1.14e-02 |
| ENSG00000130723 | 1.05e-06 | 4.95e-06 |
| ENSG00000130724 | 3.26e-05 | 1.17e-04 |
| ENSG00000130725 | 3.90e-04 | 1.13e-03 |
| ENSG00000130726 | 5.56e-12 | 6.53e-11 |
| ENSG00000130731 | 1.25e-15 | 2.93e-14 |
| ENSG00000130733 | 3.42e-05 | 1.23e-04 |
| ENSG00000130734 | 8.04e-08 | 4.60e-07 |
| ENSG00000130741 | 3.29e-16 | 8.76e-15 |
| ENSG00000130748 | 1.88e-04 | 5.82e-04 |
| ENSG00000130749 | 7.15e-03 | 1.52e-02 |
| ENSG00000130751 | 6.13e-01 | 6.85e-01 |
| ENSG00000130755 | 4.54e-07 | 2.28e-06 |
| ENSG00000130758 | 4.40e-09 | 3.12e-08 |
| ENSG00000130762 | 4.28e-02 | 7.25e-02 |
| ENSG00000130764 | 5.29e-03 | 1.16e-02 |
| ENSG00000130766 | 2.27e-13 | 3.46e-12 |
| ENSG00000130768 | 5.72e-07 | 2.82e-06 |
| ENSG00000130770 | 3.39e-05 | 1.21e-04 |
| ENSG00000130772 | 3.27e-05 | 1.18e-04 |
| ENSG00000130775 | 5.26e-02 | 8.66e-02 |
| ENSG00000130779 | 8.57e-02 | 1.32e-01 |
| ENSG00000130783 | 7.93e-01 | 8.40e-01 |
| ENSG00000130787 | 2.10e-02 | 3.90e-02 |
| ENSG00000130803 | 5.63e-11 | 5.51e-10 |
| ENSG00000130810 | 6.22e-08 | 3.63e-07 |
| ENSG00000130811 | 4.09e-07 | 2.07e-06 |
| ENSG00000130812 | 8.23e-04 | 2.22e-03 |
| ENSG00000130813 | 4.12e-07 | 2.08e-06 |
| ENSG00000130816 | 2.00e-16 | 5.60e-15 |
| ENSG00000130818 | 3.18e-01 | 3.99e-01 |
| ENSG00000130821 | 1.17e-09 | 9.11e-09 |
| ENSG00000130822 | 1.29e-04 | 4.10e-04 |
| ENSG00000130826 | 2.78e-25 | 1.01e-22 |
| ENSG00000130827 | 8.11e-15 | 1.61e-13 |
| ENSG00000130829 | 2.90e-14 | 5.20e-13 |
| ENSG00000130830 | 9.04e-01 | 9.29e-01 |
| ENSG00000130844 | 1.17e-04 | 3.78e-04 |
| ENSG00000130856 | 5.74e-02 | 9.34e-02 |
| ENSG00000130876 | 7.11e-01 | 7.71e-01 |
| ENSG00000130881 | 7.23e-03 | 1.53e-02 |
| ENSG00000130921 | 1.65e-02 | 3.16e-02 |
| ENSG00000130935 | 7.04e-07 | 3.42e-06 |
| ENSG00000130939 | 3.26e-01 | 4.07e-01 |
| ENSG00000130940 | 3.45e-10 | 2.95e-09 |
| ENSG00000130943 | 5.61e-04 | 1.57e-03 |
| ENSG00000130948 | 4.36e-01 | 5.19e-01 |
| ENSG00000130950 | 8.34e-02 | 1.29e-01 |
| ENSG00000130956 | 1.19e-03 | 3.10e-03 |
| ENSG00000130957 | 3.42e-05 | 1.22e-04 |
| ENSG00000130958 | 5.78e-07 | 2.84e-06 |
| ENSG00000130962 | 3.34e-04 | 9.78e-04 |
| ENSG00000130985 | 6.52e-05 | 2.20e-04 |
| ENSG00000130988 | 9.06e-04 | 2.42e-03 |
| ENSG00000130997 | 5.17e-03 | 1.14e-02 |
| ENSG00000131002 | 5.38e-01 | 6.17e-01 |
| ENSG00000131013 | 3.96e-01 | 4.79e-01 |
| ENSG00000131015 | 2.45e-23 | 4.44e-21 |
| ENSG00000131016 | 8.32e-08 | 4.75e-07 |
| ENSG00000131018 | 1.83e-11 | 1.94e-10 |
| ENSG00000131019 | 2.18e-16 | 6.06e-15 |
| ENSG00000131023 | 6.54e-01 | 7.22e-01 |
| ENSG00000131037 | 1.19e-03 | 3.10e-03 |
| ENSG00000131042 | 4.86e-01 | 5.67e-01 |
| ENSG00000131043 | 9.23e-07 | 4.40e-06 |
| ENSG00000131044 | 3.68e-08 | 2.24e-07 |
| ENSG00000131050 | 4.23e-01 | 5.06e-01 |
| ENSG00000131051 | 3.89e-12 | 4.70e-11 |
| ENSG00000131055 | 6.12e-01 | 6.84e-01 |
| ENSG00000131061 | 9.46e-01 | 9.60e-01 |
| ENSG00000131067 | 6.42e-01 | 7.11e-01 |
| ENSG00000131069 | 1.31e-10 | 1.20e-09 |
| ENSG00000131080 | 9.42e-05 | 3.08e-04 |
| ENSG00000131089 | 1.15e-13 | 1.85e-12 |
| ENSG00000131094 | 8.23e-09 | 5.56e-08 |
| ENSG00000131095 | 3.87e-01 | 4.70e-01 |
| ENSG00000131096 | 2.53e-21 | 2.52e-19 |
| ENSG00000131097 | 3.43e-03 | 7.95e-03 |
| ENSG00000131100 | 1.37e-03 | 3.52e-03 |
| ENSG00000131115 | 3.06e-01 | 3.86e-01 |
| ENSG00000131116 | 1.30e-02 | 2.57e-02 |
| ENSG00000131126 | 1.40e-05 | 5.41e-05 |
| ENSG00000131127 | 4.11e-02 | 6.99e-02 |
| ENSG00000131142 | 8.03e-04 | 2.17e-03 |
| ENSG00000131143 | 1.96e-03 | 4.83e-03 |
| ENSG00000131148 | 4.21e-14 | 7.32e-13 |
| ENSG00000131149 | 3.49e-07 | 1.79e-06 |
| ENSG00000131152 | 3.68e-01 | 4.51e-01 |
| ENSG00000131153 | 1.62e-12 | 2.11e-11 |
| ENSG00000131165 | 3.72e-02 | 6.42e-02 |
| ENSG00000131171 | 1.29e-08 | 8.43e-08 |
| ENSG00000131174 | 1.29e-02 | 2.55e-02 |
| ENSG00000131183 | 1.19e-03 | 3.09e-03 |
| ENSG00000131187 | 6.33e-12 | 7.34e-11 |
| ENSG00000131188 | 1.92e-26 | 1.15e-23 |
| ENSG00000131196 | 8.39e-05 | 2.77e-04 |
| ENSG00000131203 | 2.79e-02 | 5.00e-02 |
| ENSG00000131233 | 7.62e-01 | 8.14e-01 |
| ENSG00000131236 | 5.24e-08 | 3.11e-07 |
| ENSG00000131238 | 2.31e-05 | 8.54e-05 |
| ENSG00000131242 | 7.34e-01 | 7.91e-01 |
| ENSG00000131263 | 2.73e-11 | 2.82e-10 |
| ENSG00000131269 | 3.00e-03 | 7.05e-03 |
| ENSG00000131323 | 1.13e-01 | 1.67e-01 |
| ENSG00000131351 | 3.61e-02 | 6.25e-02 |
| ENSG00000131355 | 1.87e-01 | 2.55e-01 |
| ENSG00000131368 | 2.86e-02 | 5.11e-02 |
| ENSG00000131370 | 2.53e-04 | 7.61e-04 |
| ENSG00000131373 | 3.66e-05 | 1.30e-04 |
| ENSG00000131374 | 6.82e-04 | 1.87e-03 |
| ENSG00000131375 | 5.50e-01 | 6.27e-01 |
| ENSG00000131378 | 4.61e-01 | 5.43e-01 |
| ENSG00000131379 | 3.37e-02 | 5.89e-02 |
| ENSG00000131381 | 2.71e-02 | 4.88e-02 |
| ENSG00000131386 | 6.03e-10 | 4.93e-09 |
| ENSG00000131389 | 6.98e-27 | 5.13e-24 |
| ENSG00000131398 | 4.58e-03 | 1.03e-02 |
| ENSG00000131400 | 7.26e-01 | 7.84e-01 |
| ENSG00000131401 | 8.31e-10 | 6.65e-09 |
| ENSG00000131408 | 1.49e-04 | 4.68e-04 |
| ENSG00000131409 | 6.56e-10 | 5.33e-09 |
| ENSG00000131435 | 4.06e-01 | 4.89e-01 |
| ENSG00000131437 | 1.15e-07 | 6.44e-07 |
| ENSG00000131446 | 3.58e-03 | 8.26e-03 |
| ENSG00000131459 | 7.32e-01 | 7.89e-01 |
| ENSG00000131462 | 3.96e-12 | 4.78e-11 |
| ENSG00000131467 | 2.85e-11 | 2.94e-10 |
| ENSG00000131469 | 7.54e-06 | 3.05e-05 |
| ENSG00000131470 | 2.36e-13 | 3.59e-12 |
| ENSG00000131471 | 2.10e-13 | 3.21e-12 |
| ENSG00000131473 | 5.02e-17 | 1.59e-15 |
| ENSG00000131475 | 1.01e-01 | 1.52e-01 |
| ENSG00000131477 | 4.10e-01 | 4.93e-01 |
| ENSG00000131480 | 5.28e-05 | 1.82e-04 |
| ENSG00000131482 | 4.70e-13 | 6.76e-12 |
| ENSG00000131484 | 3.35e-01 | 4.17e-01 |
| ENSG00000131495 | 8.44e-04 | 2.27e-03 |
| ENSG00000131503 | 7.21e-01 | 7.80e-01 |
| ENSG00000131504 | 1.42e-05 | 5.48e-05 |
| ENSG00000131507 | 6.74e-02 | 1.07e-01 |
| ENSG00000131508 | 2.02e-01 | 2.73e-01 |
| ENSG00000131558 | 1.84e-09 | 1.39e-08 |
| ENSG00000131584 | 6.46e-07 | 3.16e-06 |
| ENSG00000131591 | 1.66e-08 | 1.07e-07 |
| ENSG00000131620 | 1.19e-05 | 4.65e-05 |
| ENSG00000131626 | 6.65e-01 | 7.31e-01 |
| ENSG00000131634 | 4.92e-03 | 1.09e-02 |
| ENSG00000131650 | 3.35e-13 | 4.94e-12 |
| ENSG00000131652 | 3.74e-09 | 2.68e-08 |
| ENSG00000131653 | 9.60e-05 | 3.14e-04 |
| ENSG00000131668 | 7.33e-01 | 7.90e-01 |
| ENSG00000131669 | 3.00e-02 | 5.32e-02 |
| ENSG00000131686 | 6.98e-03 | 1.49e-02 |
| ENSG00000131697 | 3.34e-15 | 7.21e-14 |
| ENSG00000131711 | 1.03e-05 | 4.06e-05 |
| ENSG00000131721 | 8.04e-01 | 8.50e-01 |
| ENSG00000131724 | 6.21e-02 | 9.99e-02 |
| ENSG00000131725 | 2.69e-01 | 3.47e-01 |
| ENSG00000131730 | 1.70e-05 | 6.42e-05 |
| ENSG00000131732 | 1.44e-04 | 4.54e-04 |
| ENSG00000131737 | 3.58e-06 | 1.53e-05 |
| ENSG00000131738 | 3.36e-01 | 4.18e-01 |
| ENSG00000131746 | 1.15e-15 | 2.71e-14 |
| ENSG00000131747 | 1.47e-12 | 1.93e-11 |
| ENSG00000131748 | 3.14e-02 | 5.54e-02 |
| ENSG00000131759 | 4.67e-02 | 7.81e-02 |
| ENSG00000131771 | 4.36e-01 | 5.19e-01 |
| ENSG00000131773 | 2.71e-03 | 6.45e-03 |
| ENSG00000131778 | 1.10e-03 | 2.88e-03 |
| ENSG00000131779 | 7.95e-03 | 1.67e-02 |
| ENSG00000131781 | 3.99e-18 | 1.61e-16 |
| ENSG00000131788 | 6.57e-06 | 2.68e-05 |
| ENSG00000131791 | 1.29e-02 | 2.55e-02 |
| ENSG00000131797 | 4.29e-10 | 3.61e-09 |
| ENSG00000131828 | 3.95e-01 | 4.79e-01 |
| ENSG00000131831 | 9.21e-10 | 7.32e-09 |
| ENSG00000131844 | 3.21e-04 | 9.43e-04 |
| ENSG00000131845 | 1.22e-11 | 1.33e-10 |
| ENSG00000131848 | 2.22e-01 | 2.95e-01 |
| ENSG00000131849 | 8.76e-13 | 1.20e-11 |
| ENSG00000131871 | 2.29e-07 | 1.21e-06 |
| ENSG00000131873 | 2.84e-07 | 1.48e-06 |
| ENSG00000131876 | 7.18e-14 | 1.19e-12 |
| ENSG00000131885 | 4.49e-01 | 5.32e-01 |
| ENSG00000131899 | 3.86e-04 | 1.12e-03 |
| ENSG00000131910 | 1.71e-06 | 7.73e-06 |
| ENSG00000131914 | 1.62e-01 | 2.27e-01 |
| ENSG00000131931 | 6.87e-01 | 7.51e-01 |
| ENSG00000131941 | 3.78e-01 | 4.61e-01 |
| ENSG00000131943 | 4.45e-01 | 5.28e-01 |
| ENSG00000131944 | 5.50e-07 | 2.72e-06 |
| ENSG00000131951 | 2.43e-07 | 1.28e-06 |
| ENSG00000131966 | 7.01e-05 | 2.35e-04 |
| ENSG00000131969 | 5.04e-02 | 8.35e-02 |
| ENSG00000131979 | 1.41e-01 | 2.01e-01 |
| ENSG00000131981 | 1.66e-14 | 3.14e-13 |
| ENSG00000131982 | 1.65e-01 | 2.30e-01 |
| ENSG00000132000 | 6.46e-15 | 1.31e-13 |
| ENSG00000132002 | 1.19e-07 | 6.64e-07 |
| ENSG00000132003 | 5.84e-25 | 1.87e-22 |
| ENSG00000132004 | 3.79e-07 | 1.93e-06 |
| ENSG00000132005 | 1.43e-04 | 4.52e-04 |
| ENSG00000132010 | 7.72e-01 | 8.23e-01 |
| ENSG00000132016 | 7.62e-01 | 8.14e-01 |
| ENSG00000132017 | 3.44e-07 | 1.76e-06 |
| ENSG00000132024 | 2.80e-02 | 5.01e-02 |
| ENSG00000132026 | 9.87e-07 | 4.67e-06 |
| ENSG00000132031 | 2.02e-14 | 3.75e-13 |
| ENSG00000132109 | 9.67e-01 | 9.76e-01 |
| ENSG00000132122 | 1.03e-03 | 2.70e-03 |
| ENSG00000132128 | 3.49e-03 | 8.07e-03 |
| ENSG00000132141 | 3.19e-07 | 1.65e-06 |
| ENSG00000132153 | 2.57e-09 | 1.89e-08 |
| ENSG00000132155 | 1.28e-06 | 5.93e-06 |
| ENSG00000132164 | 1.45e-03 | 3.68e-03 |
| ENSG00000132170 | 1.29e-07 | 7.17e-07 |
| ENSG00000132182 | 1.38e-12 | 1.82e-11 |
| ENSG00000132185 | 2.54e-11 | 2.63e-10 |
| ENSG00000132196 | 1.90e-16 | 5.36e-15 |
| ENSG00000132199 | 4.64e-02 | 7.77e-02 |
| ENSG00000132204 | 2.52e-02 | 4.58e-02 |
| ENSG00000132205 | 1.01e-02 | 2.07e-02 |
| ENSG00000132207 | 1.59e-03 | 4.01e-03 |
| ENSG00000132254 | 1.47e-01 | 2.08e-01 |
| ENSG00000132256 | 6.11e-05 | 2.08e-04 |
| ENSG00000132259 | 2.87e-01 | 3.67e-01 |
| ENSG00000132274 | 5.28e-02 | 8.68e-02 |
| ENSG00000132275 | 1.02e-06 | 4.83e-06 |
| ENSG00000132286 | 9.77e-04 | 2.59e-03 |
| ENSG00000132294 | 4.62e-01 | 5.44e-01 |
| ENSG00000132297 | 5.65e-01 | 6.42e-01 |
| ENSG00000132300 | 9.44e-13 | 1.28e-11 |
| ENSG00000132305 | 6.02e-04 | 1.67e-03 |
| ENSG00000132313 | 3.33e-14 | 5.88e-13 |
| ENSG00000132321 | 1.77e-04 | 5.49e-04 |
| ENSG00000132323 | 8.00e-12 | 9.10e-11 |
| ENSG00000132326 | 7.99e-01 | 8.45e-01 |
| ENSG00000132329 | 7.46e-01 | 8.02e-01 |
| ENSG00000132330 | 9.13e-20 | 5.60e-18 |
| ENSG00000132334 | 1.75e-01 | 2.41e-01 |
| ENSG00000132341 | 4.61e-19 | 2.38e-17 |
| ENSG00000132356 | 1.88e-02 | 3.54e-02 |
| ENSG00000132357 | 8.92e-02 | 1.36e-01 |
| ENSG00000132359 | 6.63e-01 | 7.29e-01 |
| ENSG00000132361 | 7.69e-01 | 8.21e-01 |
| ENSG00000132376 | 2.34e-09 | 1.74e-08 |
| ENSG00000132382 | 5.74e-13 | 8.08e-12 |
| ENSG00000132383 | 7.83e-01 | 8.32e-01 |
| ENSG00000132386 | 1.53e-02 | 2.95e-02 |
| ENSG00000132388 | 1.45e-06 | 6.66e-06 |
| ENSG00000132394 | 3.36e-09 | 2.43e-08 |
| ENSG00000132405 | 2.81e-03 | 6.65e-03 |
| ENSG00000132406 | 4.43e-03 | 9.95e-03 |
| ENSG00000132423 | 1.21e-01 | 1.77e-01 |
| ENSG00000132424 | 2.37e-01 | 3.13e-01 |
| ENSG00000132429 | 1.65e-04 | 5.14e-04 |
| ENSG00000132432 | 7.24e-04 | 1.98e-03 |
| ENSG00000132434 | 1.39e-02 | 2.72e-02 |
| ENSG00000132436 | 2.76e-15 | 6.03e-14 |
| ENSG00000132437 | 6.96e-02 | 1.10e-01 |
| ENSG00000132463 | 4.74e-01 | 5.56e-01 |
| ENSG00000132464 | 1.53e-16 | 4.36e-15 |
| ENSG00000132465 | 1.36e-18 | 6.21e-17 |
| ENSG00000132466 | 6.43e-02 | 1.03e-01 |
| ENSG00000132467 | 5.91e-03 | 1.28e-02 |
| ENSG00000132470 | 6.49e-03 | 1.39e-02 |
| ENSG00000132471 | 2.49e-04 | 7.51e-04 |
| ENSG00000132475 | 1.09e-01 | 1.62e-01 |
| ENSG00000132478 | 1.31e-03 | 3.38e-03 |
| ENSG00000132481 | 5.34e-02 | 8.76e-02 |
| ENSG00000132485 | 6.51e-01 | 7.19e-01 |
| ENSG00000132507 | 5.51e-04 | 1.54e-03 |
| ENSG00000132510 | 5.57e-01 | 6.35e-01 |
| ENSG00000132514 | 6.88e-19 | 3.39e-17 |
| ENSG00000132517 | 1.29e-09 | 9.99e-09 |
| ENSG00000132518 | 2.79e-01 | 3.58e-01 |
| ENSG00000132522 | 2.69e-01 | 3.47e-01 |
| ENSG00000132530 | 3.37e-02 | 5.88e-02 |
| ENSG00000132535 | 6.38e-02 | 1.02e-01 |
| ENSG00000132541 | 2.72e-01 | 3.51e-01 |
| ENSG00000132549 | 5.12e-01 | 5.92e-01 |
| ENSG00000132554 | 4.54e-11 | 4.52e-10 |
| ENSG00000132561 | 8.51e-15 | 1.69e-13 |
| ENSG00000132563 | 2.89e-13 | 4.31e-12 |
| ENSG00000132570 | 9.87e-04 | 2.61e-03 |
| ENSG00000132581 | 4.49e-01 | 5.32e-01 |
| ENSG00000132589 | 4.58e-01 | 5.41e-01 |
| ENSG00000132591 | 5.62e-04 | 1.57e-03 |
| ENSG00000132600 | 3.97e-09 | 2.84e-08 |
| ENSG00000132603 | 5.16e-15 | 1.07e-13 |
| ENSG00000132604 | 6.33e-04 | 1.75e-03 |
| ENSG00000132612 | 9.40e-01 | 9.55e-01 |
| ENSG00000132613 | 2.54e-02 | 4.61e-02 |
| ENSG00000132622 | 1.97e-02 | 3.68e-02 |
| ENSG00000132623 | 2.76e-09 | 2.02e-08 |
| ENSG00000132635 | 4.29e-09 | 3.05e-08 |
| ENSG00000132639 | 3.85e-16 | 1.01e-14 |
| ENSG00000132640 | 5.63e-15 | 1.15e-13 |
| ENSG00000132646 | 1.04e-13 | 1.68e-12 |
| ENSG00000132661 | 7.03e-18 | 2.68e-16 |
| ENSG00000132664 | 6.52e-08 | 3.80e-07 |
| ENSG00000132669 | 9.71e-03 | 1.99e-02 |
| ENSG00000132670 | 2.26e-01 | 3.00e-01 |
| ENSG00000132671 | 6.67e-03 | 1.43e-02 |
| ENSG00000132676 | 3.46e-13 | 5.07e-12 |
| ENSG00000132677 | 1.02e-10 | 9.54e-10 |
| ENSG00000132680 | 5.38e-14 | 9.16e-13 |
| ENSG00000132681 | 1.33e-02 | 2.61e-02 |
| ENSG00000132688 | 1.25e-02 | 2.49e-02 |
| ENSG00000132692 | 5.53e-07 | 2.73e-06 |
| ENSG00000132693 | 4.49e-01 | 5.32e-01 |
| ENSG00000132694 | 1.16e-05 | 4.53e-05 |
| ENSG00000132698 | 6.66e-02 | 1.06e-01 |
| ENSG00000132702 | 9.89e-01 | 9.92e-01 |
| ENSG00000132703 | 5.98e-01 | 6.71e-01 |
| ENSG00000132704 | 6.30e-12 | 7.31e-11 |
| ENSG00000132716 | 5.29e-01 | 6.08e-01 |
| ENSG00000132718 | 1.62e-05 | 6.17e-05 |
| ENSG00000132740 | 5.85e-02 | 9.49e-02 |
| ENSG00000132744 | 3.42e-01 | 4.24e-01 |
| ENSG00000132746 | 8.26e-13 | 1.13e-11 |
| ENSG00000132749 | 2.03e-07 | 1.09e-06 |
| ENSG00000132763 | 1.81e-04 | 5.62e-04 |
| ENSG00000132768 | 2.78e-16 | 7.52e-15 |
| ENSG00000132773 | 7.66e-01 | 8.18e-01 |
| ENSG00000132780 | 3.47e-08 | 2.11e-07 |
| ENSG00000132781 | 2.44e-05 | 8.98e-05 |
| ENSG00000132792 | 9.25e-10 | 7.35e-09 |
| ENSG00000132793 | 1.85e-03 | 4.59e-03 |
| ENSG00000132801 | 1.20e-02 | 2.40e-02 |
| ENSG00000132819 | 1.27e-01 | 1.84e-01 |
| ENSG00000132821 | 5.15e-01 | 5.95e-01 |
| ENSG00000132823 | 4.42e-10 | 3.71e-09 |
| ENSG00000132824 | 2.87e-07 | 1.50e-06 |
| ENSG00000132825 | 1.72e-05 | 6.52e-05 |
| ENSG00000132832 | 2.28e-01 | 3.02e-01 |
| ENSG00000132837 | 6.53e-08 | 3.80e-07 |
| ENSG00000132840 | 2.54e-09 | 1.87e-08 |
| ENSG00000132842 | 2.93e-01 | 3.73e-01 |
| ENSG00000132846 | 7.13e-06 | 2.89e-05 |
| ENSG00000132849 | 8.01e-01 | 8.47e-01 |
| ENSG00000132854 | 3.53e-12 | 4.31e-11 |
| ENSG00000132855 | 6.54e-02 | 1.04e-01 |
| ENSG00000132872 | 7.55e-18 | 2.86e-16 |
| ENSG00000132874 | 7.56e-04 | 2.06e-03 |
| ENSG00000132879 | 5.61e-05 | 1.92e-04 |
| ENSG00000132881 | 1.07e-01 | 1.59e-01 |
| ENSG00000132906 | 7.13e-06 | 2.89e-05 |
| ENSG00000132911 | 8.92e-04 | 2.38e-03 |
| ENSG00000132912 | 4.00e-05 | 1.41e-04 |
| ENSG00000132915 | 9.78e-22 | 1.10e-19 |
| ENSG00000132932 | 5.81e-10 | 4.78e-09 |
| ENSG00000132938 | 4.25e-03 | 9.59e-03 |
| ENSG00000132950 | 4.14e-05 | 1.46e-04 |
| ENSG00000132952 | 2.68e-15 | 5.89e-14 |
| ENSG00000132953 | 1.01e-12 | 1.36e-11 |
| ENSG00000132958 | 1.66e-02 | 3.17e-02 |
| ENSG00000132963 | 4.87e-10 | 4.06e-09 |
| ENSG00000132964 | 2.01e-13 | 3.08e-12 |
| ENSG00000132965 | 5.53e-01 | 6.30e-01 |
| ENSG00000132967 | 1.38e-01 | 1.98e-01 |
| ENSG00000132970 | 1.65e-04 | 5.16e-04 |
| ENSG00000132972 | 5.90e-01 | 6.65e-01 |
| ENSG00000132975 | 2.64e-14 | 4.78e-13 |
| ENSG00000133019 | 1.95e-02 | 3.65e-02 |
| ENSG00000133020 | 3.75e-01 | 4.58e-01 |
| ENSG00000133026 | 4.77e-02 | 7.95e-02 |
| ENSG00000133027 | 1.49e-08 | 9.65e-08 |
| ENSG00000133028 | 2.39e-11 | 2.49e-10 |
| ENSG00000133030 | 5.96e-01 | 6.70e-01 |
| ENSG00000133048 | 2.37e-17 | 8.11e-16 |
| ENSG00000133055 | 8.81e-05 | 2.90e-04 |
| ENSG00000133056 | 4.84e-01 | 5.66e-01 |
| ENSG00000133059 | 9.35e-02 | 1.42e-01 |
| ENSG00000133063 | 3.22e-02 | 5.66e-02 |
| ENSG00000133065 | 2.44e-09 | 1.80e-08 |
| ENSG00000133067 | 1.04e-02 | 2.11e-02 |
| ENSG00000133069 | 1.85e-04 | 5.73e-04 |
| ENSG00000133083 | 1.13e-18 | 5.23e-17 |
| ENSG00000133101 | 3.94e-06 | 1.67e-05 |
| ENSG00000133103 | 5.00e-05 | 1.73e-04 |
| ENSG00000133104 | 2.40e-10 | 2.11e-09 |
| ENSG00000133105 | 5.18e-01 | 5.98e-01 |
| ENSG00000133106 | 3.63e-06 | 1.55e-05 |
| ENSG00000133107 | 8.89e-01 | 9.17e-01 |
| ENSG00000133110 | 8.98e-04 | 2.40e-03 |
| ENSG00000133111 | 3.68e-12 | 4.47e-11 |
| ENSG00000133112 | 1.64e-01 | 2.29e-01 |
| ENSG00000133114 | 9.78e-11 | 9.19e-10 |
| ENSG00000133115 | 3.53e-02 | 6.13e-02 |
| ENSG00000133116 | 4.51e-14 | 7.79e-13 |
| ENSG00000133119 | 2.21e-21 | 2.23e-19 |
| ENSG00000133121 | 6.25e-03 | 1.35e-02 |
| ENSG00000133124 | 2.95e-01 | 3.75e-01 |
| ENSG00000133131 | 3.12e-16 | 8.37e-15 |
| ENSG00000133134 | 8.06e-05 | 2.68e-04 |
| ENSG00000133135 | 1.01e-02 | 2.05e-02 |
| ENSG00000133136 | 3.16e-01 | 3.97e-01 |
| ENSG00000133138 | 3.50e-02 | 6.08e-02 |
| ENSG00000133142 | 7.91e-01 | 8.38e-01 |
| ENSG00000133169 | 5.17e-15 | 1.07e-13 |
| ENSG00000133193 | 4.16e-04 | 1.19e-03 |
| ENSG00000133195 | 1.01e-02 | 2.05e-02 |
| ENSG00000133216 | 3.36e-06 | 1.44e-05 |
| ENSG00000133226 | 2.52e-01 | 3.29e-01 |
| ENSG00000133243 | 3.20e-03 | 7.46e-03 |
| ENSG00000133246 | 1.33e-01 | 1.91e-01 |
| ENSG00000133247 | 2.59e-10 | 2.27e-09 |
| ENSG00000133250 | 1.37e-02 | 2.68e-02 |
| ENSG00000133256 | 1.10e-08 | 7.28e-08 |
| ENSG00000133265 | 5.46e-06 | 2.25e-05 |
| ENSG00000133275 | 7.11e-11 | 6.85e-10 |
| ENSG00000133302 | 4.65e-02 | 7.78e-02 |
| ENSG00000133313 | 8.81e-06 | 3.52e-05 |
| ENSG00000133315 | 1.07e-08 | 7.09e-08 |
| ENSG00000133316 | 1.89e-19 | 1.07e-17 |
| ENSG00000133317 | 7.78e-03 | 1.64e-02 |
| ENSG00000133318 | 3.89e-04 | 1.12e-03 |
| ENSG00000133321 | 8.44e-08 | 4.82e-07 |
| ENSG00000133328 | 8.84e-19 | 4.24e-17 |
| ENSG00000133392 | 2.81e-14 | 5.05e-13 |
| ENSG00000133393 | 1.47e-06 | 6.75e-06 |
| ENSG00000133398 | 2.20e-09 | 1.64e-08 |
| ENSG00000133401 | 6.21e-01 | 6.92e-01 |
| ENSG00000133422 | 2.74e-19 | 1.48e-17 |
| ENSG00000133424 | 9.95e-18 | 3.66e-16 |
| ENSG00000133433 | 9.21e-01 | 9.41e-01 |
| ENSG00000133454 | 8.76e-04 | 2.34e-03 |
| ENSG00000133460 | 6.66e-02 | 1.06e-01 |
| ENSG00000133466 | 5.87e-08 | 3.45e-07 |
| ENSG00000133475 | 2.52e-02 | 4.57e-02 |
| ENSG00000133477 | 4.46e-01 | 5.30e-01 |
| ENSG00000133488 | 3.15e-07 | 1.63e-06 |
| ENSG00000133519 | 1.76e-03 | 4.39e-03 |
| ENSG00000133561 | 1.87e-14 | 3.50e-13 |
| ENSG00000133574 | 7.24e-11 | 6.95e-10 |
| ENSG00000133597 | 8.01e-02 | 1.24e-01 |
| ENSG00000133606 | 3.16e-05 | 1.14e-04 |
| ENSG00000133612 | 1.57e-18 | 6.98e-17 |
| ENSG00000133619 | 3.30e-10 | 2.83e-09 |
| ENSG00000133624 | 5.41e-07 | 2.67e-06 |
| ENSG00000133627 | 9.48e-24 | 2.08e-21 |
| ENSG00000133636 | 1.53e-03 | 3.86e-03 |
| ENSG00000133639 | 9.78e-11 | 9.19e-10 |
| ENSG00000133640 | 1.15e-01 | 1.69e-01 |
| ENSG00000133641 | 3.77e-09 | 2.70e-08 |
| ENSG00000133657 | 3.97e-04 | 1.15e-03 |
| ENSG00000133661 | 4.41e-09 | 3.12e-08 |
| ENSG00000133665 | 6.34e-01 | 7.04e-01 |
| ENSG00000133678 | 6.18e-09 | 4.28e-08 |
| ENSG00000133687 | 2.10e-10 | 1.87e-09 |
| ENSG00000133703 | 4.51e-07 | 2.27e-06 |
| ENSG00000133704 | 2.62e-02 | 4.74e-02 |
| ENSG00000133706 | 1.60e-08 | 1.03e-07 |
| ENSG00000133710 | 2.38e-09 | 1.76e-08 |
| ENSG00000133731 | 2.14e-12 | 2.72e-11 |
| ENSG00000133739 | 6.55e-01 | 7.22e-01 |
| ENSG00000133740 | 6.04e-18 | 2.33e-16 |
| ENSG00000133742 | 1.36e-21 | 1.49e-19 |
| ENSG00000133773 | 8.42e-11 | 8.01e-10 |
| ENSG00000133789 | 4.99e-01 | 5.80e-01 |
| ENSG00000133794 | 8.01e-06 | 3.23e-05 |
| ENSG00000133800 | 4.48e-23 | 7.48e-21 |
| ENSG00000133805 | 5.07e-05 | 1.75e-04 |
| ENSG00000133808 | 3.81e-14 | 6.67e-13 |
| ENSG00000133812 | 3.84e-01 | 4.67e-01 |
| ENSG00000133816 | 7.99e-09 | 5.42e-08 |
| ENSG00000133818 | 8.10e-06 | 3.26e-05 |
| ENSG00000133835 | 4.39e-01 | 5.22e-01 |
| ENSG00000133858 | 2.52e-06 | 1.10e-05 |
| ENSG00000133863 | 4.66e-03 | 1.04e-02 |
| ENSG00000133872 | 3.13e-09 | 2.27e-08 |
| ENSG00000133874 | 7.85e-01 | 8.34e-01 |
| ENSG00000133878 | 5.61e-19 | 2.83e-17 |
| ENSG00000133884 | 2.85e-06 | 1.24e-05 |
| ENSG00000133895 | 1.02e-07 | 5.76e-07 |
| ENSG00000133935 | 3.53e-03 | 8.15e-03 |
| ENSG00000133937 | 3.93e-06 | 1.67e-05 |
| ENSG00000133943 | 6.66e-12 | 7.68e-11 |
| ENSG00000133958 | 1.14e-13 | 1.83e-12 |
| ENSG00000133961 | 5.17e-09 | 3.63e-08 |
| ENSG00000133962 | 2.69e-01 | 3.47e-01 |
| ENSG00000133980 | 1.89e-01 | 2.58e-01 |
| ENSG00000133983 | 4.05e-04 | 1.17e-03 |
| ENSG00000133985 | 1.45e-07 | 7.93e-07 |
| ENSG00000133997 | 1.12e-02 | 2.25e-02 |
| ENSG00000134001 | 1.26e-06 | 5.85e-06 |
| ENSG00000134007 | 7.36e-02 | 1.16e-01 |
| ENSG00000134013 | 3.28e-18 | 1.35e-16 |
| ENSG00000134014 | 9.42e-01 | 9.57e-01 |
| ENSG00000134020 | 9.75e-07 | 4.62e-06 |
| ENSG00000134028 | 5.60e-23 | 8.92e-21 |
| ENSG00000134030 | 9.87e-01 | 9.91e-01 |
| ENSG00000134042 | 4.21e-01 | 5.04e-01 |
| ENSG00000134046 | 2.46e-05 | 9.07e-05 |
| ENSG00000134049 | 5.50e-05 | 1.89e-04 |
| ENSG00000134056 | 2.61e-09 | 1.92e-08 |
| ENSG00000134057 | 2.37e-15 | 5.24e-14 |
| ENSG00000134058 | 1.73e-09 | 1.31e-08 |
| ENSG00000134061 | 2.37e-09 | 1.76e-08 |
| ENSG00000134070 | 1.75e-16 | 4.96e-15 |
| ENSG00000134072 | 7.07e-03 | 1.50e-02 |
| ENSG00000134077 | 1.60e-06 | 7.25e-06 |
| ENSG00000134086 | 1.03e-05 | 4.06e-05 |
| ENSG00000134107 | 1.98e-18 | 8.61e-17 |
| ENSG00000134108 | 7.96e-02 | 1.24e-01 |
| ENSG00000134109 | 1.03e-01 | 1.54e-01 |
| ENSG00000134115 | 1.52e-04 | 4.77e-04 |
| ENSG00000134121 | 1.93e-19 | 1.09e-17 |
| ENSG00000134138 | 6.42e-08 | 3.74e-07 |
| ENSG00000134146 | 5.96e-11 | 5.80e-10 |
| ENSG00000134152 | 3.79e-02 | 6.53e-02 |
| ENSG00000134153 | 3.51e-01 | 4.33e-01 |
| ENSG00000134160 | 6.50e-02 | 1.04e-01 |
| ENSG00000134184 | 4.83e-05 | 1.68e-04 |
| ENSG00000134186 | 2.78e-06 | 1.21e-05 |
| ENSG00000134193 | 4.09e-03 | 9.29e-03 |
| ENSG00000134198 | 6.20e-05 | 2.10e-04 |
| ENSG00000134200 | 9.71e-05 | 3.17e-04 |
| ENSG00000134201 | 8.15e-27 | 5.59e-24 |
| ENSG00000134202 | 5.80e-07 | 2.85e-06 |
| ENSG00000134207 | 5.27e-09 | 3.69e-08 |
| ENSG00000134215 | 1.35e-03 | 3.45e-03 |
| ENSG00000134216 | 5.22e-02 | 8.61e-02 |
| ENSG00000134222 | 3.82e-15 | 8.18e-14 |
| ENSG00000134240 | 1.55e-13 | 2.43e-12 |
| ENSG00000134242 | 1.61e-04 | 5.04e-04 |
| ENSG00000134243 | 2.02e-05 | 7.56e-05 |
| ENSG00000134245 | 5.69e-10 | 4.69e-09 |
| ENSG00000134247 | 6.22e-01 | 6.93e-01 |
| ENSG00000134248 | 1.85e-04 | 5.73e-04 |
| ENSG00000134250 | 2.43e-02 | 4.42e-02 |
| ENSG00000134253 | 3.90e-03 | 8.90e-03 |
| ENSG00000134255 | 3.40e-01 | 4.22e-01 |
| ENSG00000134256 | 1.30e-09 | 1.01e-08 |
| ENSG00000134258 | 1.03e-06 | 4.86e-06 |
| ENSG00000134259 | 6.85e-05 | 2.31e-04 |
| ENSG00000134262 | 8.06e-02 | 1.25e-01 |
| ENSG00000134265 | 4.41e-03 | 9.92e-03 |
| ENSG00000134278 | 2.25e-03 | 5.47e-03 |
| ENSG00000134283 | 4.18e-07 | 2.11e-06 |
| ENSG00000134285 | 1.45e-03 | 3.68e-03 |
| ENSG00000134287 | 3.97e-07 | 2.01e-06 |
| ENSG00000134291 | 8.22e-01 | 8.64e-01 |
| ENSG00000134294 | 1.67e-03 | 4.19e-03 |
| ENSG00000134297 | 2.29e-21 | 2.30e-19 |
| ENSG00000134308 | 2.22e-02 | 4.09e-02 |
| ENSG00000134313 | 1.13e-03 | 2.95e-03 |
| ENSG00000134317 | 2.17e-19 | 1.21e-17 |
| ENSG00000134318 | 8.72e-01 | 9.04e-01 |
| ENSG00000134321 | 5.73e-08 | 3.37e-07 |
| ENSG00000134323 | 2.80e-07 | 1.46e-06 |
| ENSG00000134324 | 9.75e-07 | 4.62e-06 |
| ENSG00000134326 | 8.21e-02 | 1.27e-01 |
| ENSG00000134330 | 2.81e-04 | 8.35e-04 |
| ENSG00000134333 | 2.94e-07 | 1.53e-06 |
| ENSG00000134339 | 8.12e-07 | 3.91e-06 |
| ENSG00000134343 | 2.96e-08 | 1.83e-07 |
| ENSG00000134352 | 1.17e-09 | 9.15e-09 |
| ENSG00000134363 | 2.02e-03 | 4.95e-03 |
| ENSG00000134365 | 1.02e-01 | 1.52e-01 |
| ENSG00000134369 | 3.04e-01 | 3.85e-01 |
| ENSG00000134371 | 1.75e-01 | 2.41e-01 |
| ENSG00000134375 | 7.61e-03 | 1.60e-02 |
| ENSG00000134376 | 5.55e-08 | 3.28e-07 |
| ENSG00000134389 | 5.42e-01 | 6.20e-01 |
| ENSG00000134398 | 2.06e-03 | 5.04e-03 |
| ENSG00000134419 | 2.63e-04 | 7.88e-04 |
| ENSG00000134438 | 2.47e-04 | 7.44e-04 |
| ENSG00000134440 | 1.33e-05 | 5.15e-05 |
| ENSG00000134443 | 6.10e-06 | 2.50e-05 |
| ENSG00000134444 | 1.23e-06 | 5.73e-06 |
| ENSG00000134452 | 1.09e-01 | 1.61e-01 |
| ENSG00000134453 | 5.73e-06 | 2.36e-05 |
| ENSG00000134460 | 4.90e-02 | 8.15e-02 |
| ENSG00000134461 | 1.10e-12 | 1.47e-11 |
| ENSG00000134463 | 5.10e-09 | 3.58e-08 |
| ENSG00000134470 | 1.95e-06 | 8.73e-06 |
| ENSG00000134480 | 8.53e-06 | 3.42e-05 |
| ENSG00000134489 | 9.51e-01 | 9.64e-01 |
| ENSG00000134490 | 3.57e-17 | 1.17e-15 |
| ENSG00000134504 | 6.93e-07 | 3.37e-06 |
| ENSG00000134508 | 5.81e-01 | 6.56e-01 |
| ENSG00000134516 | 1.59e-08 | 1.03e-07 |
| ENSG00000134531 | 4.33e-14 | 7.52e-13 |
| ENSG00000134532 | 3.17e-10 | 2.73e-09 |
| ENSG00000134533 | 1.43e-10 | 1.30e-09 |
| ENSG00000134538 | 7.45e-04 | 2.03e-03 |
| ENSG00000134539 | 1.33e-03 | 3.43e-03 |
| ENSG00000134545 | 3.16e-07 | 1.63e-06 |
| ENSG00000134548 | 7.78e-09 | 5.29e-08 |
| ENSG00000134551 | 1.95e-02 | 3.66e-02 |
| ENSG00000134569 | 9.09e-02 | 1.39e-01 |
| ENSG00000134571 | 3.98e-08 | 2.40e-07 |
| ENSG00000134574 | 1.60e-02 | 3.07e-02 |
| ENSG00000134575 | 8.53e-08 | 4.87e-07 |
| ENSG00000134588 | 3.98e-15 | 8.46e-14 |
| ENSG00000134590 | 1.31e-02 | 2.58e-02 |
| ENSG00000134594 | 3.41e-04 | 9.96e-04 |
| ENSG00000134597 | 1.83e-12 | 2.36e-11 |
| ENSG00000134602 | 2.95e-04 | 8.72e-04 |
| ENSG00000134612 | 3.00e-02 | 5.32e-02 |
| ENSG00000134627 | 5.83e-09 | 4.05e-08 |
| ENSG00000134640 | 4.85e-01 | 5.67e-01 |
| ENSG00000134644 | 3.27e-06 | 1.41e-05 |
| ENSG00000134668 | 6.90e-16 | 1.71e-14 |
| ENSG00000134684 | 2.54e-17 | 8.59e-16 |
| ENSG00000134686 | 2.00e-09 | 1.50e-08 |
| ENSG00000134690 | 2.63e-14 | 4.77e-13 |
| ENSG00000134697 | 1.83e-08 | 1.17e-07 |
| ENSG00000134698 | 8.91e-01 | 9.19e-01 |
| ENSG00000134709 | 2.60e-01 | 3.38e-01 |
| ENSG00000134716 | 1.37e-05 | 5.31e-05 |
| ENSG00000134717 | 2.28e-03 | 5.54e-03 |
| ENSG00000134744 | 4.10e-03 | 9.30e-03 |
| ENSG00000134748 | 4.77e-01 | 5.59e-01 |
| ENSG00000134755 | 3.13e-16 | 8.39e-15 |
| ENSG00000134757 | 1.80e-12 | 2.32e-11 |
| ENSG00000134758 | 9.50e-13 | 1.29e-11 |
| ENSG00000134759 | 2.14e-02 | 3.97e-02 |
| ENSG00000134760 | 9.44e-14 | 1.54e-12 |
| ENSG00000134762 | 4.16e-07 | 2.10e-06 |
| ENSG00000134765 | 6.48e-01 | 7.17e-01 |
| ENSG00000134769 | 7.14e-07 | 3.46e-06 |
| ENSG00000134775 | 4.94e-02 | 8.20e-02 |
| ENSG00000134779 | 2.46e-02 | 4.47e-02 |
| ENSG00000134780 | 3.00e-01 | 3.80e-01 |
| ENSG00000134802 | 1.31e-01 | 1.89e-01 |
| ENSG00000134809 | 2.71e-06 | 1.18e-05 |
| ENSG00000134812 | 1.18e-12 | 1.58e-11 |
| ENSG00000134815 | 1.25e-17 | 4.48e-16 |
| ENSG00000134817 | 8.77e-03 | 1.82e-02 |
| ENSG00000134824 | 2.48e-10 | 2.18e-09 |
| ENSG00000134825 | 9.93e-06 | 3.93e-05 |
| ENSG00000134827 | 1.16e-17 | 4.19e-16 |
| ENSG00000134830 | 9.55e-01 | 9.67e-01 |
| ENSG00000134851 | 4.95e-14 | 8.48e-13 |
| ENSG00000134852 | 3.73e-01 | 4.56e-01 |
| ENSG00000134853 | 3.65e-10 | 3.10e-09 |
| ENSG00000134864 | 3.62e-03 | 8.33e-03 |
| ENSG00000134871 | 1.34e-07 | 7.41e-07 |
| ENSG00000134873 | 5.11e-01 | 5.91e-01 |
| ENSG00000134874 | 5.31e-07 | 2.63e-06 |
| ENSG00000134882 | 3.07e-12 | 3.80e-11 |
| ENSG00000134884 | 1.25e-07 | 6.94e-07 |
| ENSG00000134897 | 1.83e-04 | 5.68e-04 |
| ENSG00000134899 | 2.46e-01 | 3.22e-01 |
| ENSG00000134900 | 4.68e-07 | 2.35e-06 |
| ENSG00000134901 | 7.50e-13 | 1.04e-11 |
| ENSG00000134905 | 1.67e-14 | 3.15e-13 |
| ENSG00000134909 | 4.56e-01 | 5.39e-01 |
| ENSG00000134910 | 6.07e-11 | 5.91e-10 |
| ENSG00000134917 | 7.30e-05 | 2.44e-04 |
| ENSG00000134940 | 1.42e-05 | 5.46e-05 |
| ENSG00000134954 | 1.65e-03 | 4.13e-03 |
| ENSG00000134955 | 8.35e-07 | 4.01e-06 |
| ENSG00000134962 | 3.05e-15 | 6.63e-14 |
| ENSG00000134970 | 2.42e-02 | 4.41e-02 |
| ENSG00000134982 | 5.99e-08 | 3.51e-07 |
| ENSG00000134986 | 4.29e-02 | 7.26e-02 |
| ENSG00000134987 | 1.62e-06 | 7.35e-06 |
| ENSG00000134996 | 9.69e-03 | 1.98e-02 |
| ENSG00000135002 | 6.02e-06 | 2.47e-05 |
| ENSG00000135018 | 2.09e-05 | 7.80e-05 |
| ENSG00000135040 | 7.25e-01 | 7.83e-01 |
| ENSG00000135045 | 2.19e-02 | 4.04e-02 |
| ENSG00000135046 | 8.62e-01 | 8.96e-01 |
| ENSG00000135047 | 1.78e-01 | 2.45e-01 |
| ENSG00000135048 | 1.12e-02 | 2.25e-02 |
| ENSG00000135049 | 7.57e-11 | 7.25e-10 |
| ENSG00000135052 | 1.64e-15 | 3.73e-14 |
| ENSG00000135063 | 9.72e-18 | 3.59e-16 |
| ENSG00000135069 | 8.96e-23 | 1.37e-20 |
| ENSG00000135070 | 2.68e-03 | 6.39e-03 |
| ENSG00000135074 | 2.71e-07 | 1.42e-06 |
| ENSG00000135077 | 7.17e-01 | 7.76e-01 |
| ENSG00000135083 | 6.52e-12 | 7.53e-11 |
| ENSG00000135090 | 2.84e-07 | 1.48e-06 |
| ENSG00000135093 | 4.49e-10 | 3.76e-09 |
| ENSG00000135094 | 1.05e-01 | 1.57e-01 |
| ENSG00000135097 | 9.58e-01 | 9.69e-01 |
| ENSG00000135100 | 4.10e-05 | 1.44e-04 |
| ENSG00000135108 | 5.59e-09 | 3.90e-08 |
| ENSG00000135111 | 1.34e-07 | 7.42e-07 |
| ENSG00000135114 | 6.70e-05 | 2.26e-04 |
| ENSG00000135116 | 5.23e-15 | 1.08e-13 |
| ENSG00000135119 | 2.08e-17 | 7.24e-16 |
| ENSG00000135124 | 1.37e-14 | 2.63e-13 |
| ENSG00000135127 | 7.47e-12 | 8.54e-11 |
| ENSG00000135144 | 2.44e-05 | 8.99e-05 |
| ENSG00000135148 | 2.67e-03 | 6.37e-03 |
| ENSG00000135164 | 1.67e-03 | 4.18e-03 |
| ENSG00000135185 | 3.21e-13 | 4.75e-12 |
| ENSG00000135205 | 2.75e-01 | 3.53e-01 |
| ENSG00000135211 | 6.02e-01 | 6.74e-01 |
| ENSG00000135218 | 1.52e-19 | 8.85e-18 |
| ENSG00000135220 | 1.39e-15 | 3.23e-14 |
| ENSG00000135222 | 8.92e-01 | 9.19e-01 |
| ENSG00000135226 | 3.34e-01 | 4.16e-01 |
| ENSG00000135241 | 3.92e-01 | 4.75e-01 |
| ENSG00000135245 | 1.47e-27 | 1.26e-24 |
| ENSG00000135248 | 6.00e-01 | 6.73e-01 |
| ENSG00000135249 | 5.11e-10 | 4.24e-09 |
| ENSG00000135250 | 1.01e-03 | 2.66e-03 |
| ENSG00000135253 | 8.46e-15 | 1.68e-13 |
| ENSG00000135269 | 3.62e-02 | 6.28e-02 |
| ENSG00000135272 | 7.67e-07 | 3.70e-06 |
| ENSG00000135297 | 7.11e-02 | 1.12e-01 |
| ENSG00000135298 | 5.35e-19 | 2.71e-17 |
| ENSG00000135299 | 1.40e-07 | 7.67e-07 |
| ENSG00000135312 | 8.38e-05 | 2.77e-04 |
| ENSG00000135314 | 2.06e-02 | 3.84e-02 |
| ENSG00000135315 | 7.10e-01 | 7.71e-01 |
| ENSG00000135316 | 1.74e-08 | 1.12e-07 |
| ENSG00000135317 | 9.67e-02 | 1.46e-01 |
| ENSG00000135318 | 1.94e-02 | 3.65e-02 |
| ENSG00000135324 | 8.87e-03 | 1.84e-02 |
| ENSG00000135333 | 8.28e-16 | 2.01e-14 |
| ENSG00000135334 | 6.28e-11 | 6.10e-10 |
| ENSG00000135336 | 5.04e-09 | 3.54e-08 |
| ENSG00000135338 | 4.02e-01 | 4.85e-01 |
| ENSG00000135341 | 3.82e-03 | 8.74e-03 |
| ENSG00000135346 | 8.99e-03 | 1.86e-02 |
| ENSG00000135362 | 2.53e-11 | 2.62e-10 |
| ENSG00000135363 | 2.27e-12 | 2.88e-11 |
| ENSG00000135365 | 2.92e-01 | 3.72e-01 |
| ENSG00000135372 | 2.02e-19 | 1.14e-17 |
| ENSG00000135373 | 3.19e-01 | 4.01e-01 |
| ENSG00000135374 | 3.55e-11 | 3.60e-10 |
| ENSG00000135378 | 7.29e-06 | 2.95e-05 |
| ENSG00000135387 | 2.44e-10 | 2.14e-09 |
| ENSG00000135390 | 6.15e-01 | 6.86e-01 |
| ENSG00000135392 | 9.78e-01 | 9.84e-01 |
| ENSG00000135404 | 2.34e-03 | 5.65e-03 |
| ENSG00000135406 | 3.46e-23 | 5.99e-21 |
| ENSG00000135407 | 1.89e-06 | 8.50e-06 |
| ENSG00000135409 | 2.75e-08 | 1.70e-07 |
| ENSG00000135414 | 5.26e-08 | 3.12e-07 |
| ENSG00000135423 | 4.87e-19 | 2.50e-17 |
| ENSG00000135424 | 4.21e-07 | 2.12e-06 |
| ENSG00000135426 | 6.48e-12 | 7.50e-11 |
| ENSG00000135436 | 2.39e-10 | 2.10e-09 |
| ENSG00000135437 | 8.55e-24 | 1.90e-21 |
| ENSG00000135439 | 2.19e-01 | 2.91e-01 |
| ENSG00000135441 | 1.34e-07 | 7.39e-07 |
| ENSG00000135443 | 2.66e-04 | 7.96e-04 |
| ENSG00000135446 | 1.25e-17 | 4.48e-16 |
| ENSG00000135447 | 1.38e-11 | 1.50e-10 |
| ENSG00000135451 | 1.38e-18 | 6.25e-17 |
| ENSG00000135452 | 1.59e-03 | 4.01e-03 |
| ENSG00000135454 | 9.71e-04 | 2.57e-03 |
| ENSG00000135457 | 6.27e-04 | 1.74e-03 |
| ENSG00000135469 | 2.37e-02 | 4.33e-02 |
| ENSG00000135472 | 5.80e-01 | 6.55e-01 |
| ENSG00000135473 | 1.49e-08 | 9.66e-08 |
| ENSG00000135476 | 9.92e-13 | 1.34e-11 |
| ENSG00000135477 | 7.73e-10 | 6.22e-09 |
| ENSG00000135480 | 1.65e-17 | 5.82e-16 |
| ENSG00000135482 | 1.36e-01 | 1.96e-01 |
| ENSG00000135486 | 2.28e-16 | 6.32e-15 |
| ENSG00000135502 | 7.18e-01 | 7.77e-01 |
| ENSG00000135503 | 5.09e-02 | 8.41e-02 |
| ENSG00000135506 | 8.83e-05 | 2.91e-04 |
| ENSG00000135517 | 1.16e-02 | 2.32e-02 |
| ENSG00000135519 | 7.90e-01 | 8.38e-01 |
| ENSG00000135521 | 1.44e-07 | 7.87e-07 |
| ENSG00000135525 | 1.99e-03 | 4.89e-03 |
| ENSG00000135535 | 9.15e-08 | 5.20e-07 |
| ENSG00000135537 | 1.40e-06 | 6.43e-06 |
| ENSG00000135540 | 1.22e-09 | 9.53e-09 |
| ENSG00000135541 | 2.04e-05 | 7.63e-05 |
| ENSG00000135547 | 7.39e-01 | 7.95e-01 |
| ENSG00000135549 | 2.14e-26 | 1.26e-23 |
| ENSG00000135577 | 1.37e-04 | 4.35e-04 |
| ENSG00000135587 | 1.81e-03 | 4.50e-03 |
| ENSG00000135596 | 1.08e-01 | 1.60e-01 |
| ENSG00000135597 | 1.75e-08 | 1.12e-07 |
| ENSG00000135604 | 2.15e-04 | 6.57e-04 |
| ENSG00000135605 | 4.64e-03 | 1.04e-02 |
| ENSG00000135617 | 2.03e-08 | 1.28e-07 |
| ENSG00000135622 | 2.90e-06 | 1.26e-05 |
| ENSG00000135624 | 3.42e-12 | 4.18e-11 |
| ENSG00000135625 | 2.69e-07 | 1.41e-06 |
| ENSG00000135631 | 3.36e-01 | 4.18e-01 |
| ENSG00000135632 | 6.81e-19 | 3.38e-17 |
| ENSG00000135636 | 2.93e-01 | 3.73e-01 |
| ENSG00000135637 | 1.99e-01 | 2.69e-01 |
| ENSG00000135638 | 4.90e-04 | 1.39e-03 |
| ENSG00000135643 | 5.58e-01 | 6.35e-01 |
| ENSG00000135655 | 2.72e-01 | 3.51e-01 |
| ENSG00000135677 | 2.14e-01 | 2.86e-01 |
| ENSG00000135678 | 1.11e-23 | 2.33e-21 |
| ENSG00000135679 | 8.52e-02 | 1.31e-01 |
| ENSG00000135686 | 1.34e-01 | 1.93e-01 |
| ENSG00000135697 | 2.67e-04 | 7.98e-04 |
| ENSG00000135698 | 2.34e-04 | 7.08e-04 |
| ENSG00000135702 | 8.05e-13 | 1.11e-11 |
| ENSG00000135709 | 1.58e-20 | 1.20e-18 |
| ENSG00000135720 | 8.56e-06 | 3.43e-05 |
| ENSG00000135722 | 3.66e-09 | 2.63e-08 |
| ENSG00000135723 | 6.64e-06 | 2.71e-05 |
| ENSG00000135736 | 3.80e-01 | 4.63e-01 |
| ENSG00000135740 | 4.79e-10 | 3.99e-09 |
| ENSG00000135744 | 1.63e-12 | 2.12e-11 |
| ENSG00000135747 | 3.56e-11 | 3.61e-10 |
| ENSG00000135749 | 2.62e-13 | 3.94e-12 |
| ENSG00000135750 | 2.13e-01 | 2.85e-01 |
| ENSG00000135763 | 2.30e-23 | 4.23e-21 |
| ENSG00000135766 | 1.25e-17 | 4.48e-16 |
| ENSG00000135773 | 1.11e-09 | 8.72e-09 |
| ENSG00000135775 | 1.17e-01 | 1.71e-01 |
| ENSG00000135776 | 7.46e-01 | 8.01e-01 |
| ENSG00000135778 | 1.91e-02 | 3.60e-02 |
| ENSG00000135801 | 1.70e-03 | 4.24e-03 |
| ENSG00000135821 | 8.74e-01 | 9.06e-01 |
| ENSG00000135823 | 2.82e-13 | 4.22e-12 |
| ENSG00000135824 | 2.24e-02 | 4.13e-02 |
| ENSG00000135828 | 1.86e-16 | 5.24e-15 |
| ENSG00000135829 | 9.83e-10 | 7.78e-09 |
| ENSG00000135835 | 3.95e-04 | 1.14e-03 |
| ENSG00000135837 | 2.85e-01 | 3.65e-01 |
| ENSG00000135838 | 6.89e-01 | 7.52e-01 |
| ENSG00000135842 | 1.03e-15 | 2.45e-14 |
| ENSG00000135845 | 6.40e-05 | 2.17e-04 |
| ENSG00000135862 | 1.38e-02 | 2.69e-02 |
| ENSG00000135870 | 5.57e-02 | 9.09e-02 |
| ENSG00000135898 | 7.19e-11 | 6.91e-10 |
| ENSG00000135899 | 3.91e-03 | 8.93e-03 |
| ENSG00000135900 | 2.23e-01 | 2.96e-01 |
| ENSG00000135902 | 9.25e-05 | 3.04e-04 |
| ENSG00000135903 | 9.02e-01 | 9.27e-01 |
| ENSG00000135905 | 7.48e-10 | 6.03e-09 |
| ENSG00000135912 | 1.11e-19 | 6.68e-18 |
| ENSG00000135913 | 1.08e-03 | 2.83e-03 |
| ENSG00000135914 | 1.16e-04 | 3.74e-04 |
| ENSG00000135916 | 4.95e-20 | 3.23e-18 |
| ENSG00000135917 | 6.80e-03 | 1.45e-02 |
| ENSG00000135919 | 2.09e-12 | 2.67e-11 |
| ENSG00000135924 | 1.07e-02 | 2.17e-02 |
| ENSG00000135925 | 5.77e-03 | 1.26e-02 |
| ENSG00000135926 | 1.65e-06 | 7.47e-06 |
| ENSG00000135929 | 8.29e-05 | 2.75e-04 |
| ENSG00000135930 | 1.45e-01 | 2.06e-01 |
| ENSG00000135931 | 8.40e-05 | 2.78e-04 |
| ENSG00000135932 | 2.97e-12 | 3.68e-11 |
| ENSG00000135940 | 9.72e-03 | 1.99e-02 |
| ENSG00000135945 | 7.47e-02 | 1.17e-01 |
| ENSG00000135951 | 4.52e-04 | 1.29e-03 |
| ENSG00000135953 | 1.80e-04 | 5.58e-04 |
| ENSG00000135956 | 6.52e-15 | 1.32e-13 |
| ENSG00000135960 | 1.27e-04 | 4.05e-04 |
| ENSG00000135966 | 2.97e-11 | 3.05e-10 |
| ENSG00000135968 | 4.82e-03 | 1.07e-02 |
| ENSG00000135972 | 1.91e-01 | 2.61e-01 |
| ENSG00000135973 | 5.92e-01 | 6.66e-01 |
| ENSG00000135974 | 5.88e-06 | 2.42e-05 |
| ENSG00000135976 | 3.15e-03 | 7.36e-03 |
| ENSG00000135999 | 4.47e-01 | 5.31e-01 |
| ENSG00000136002 | 7.53e-01 | 8.07e-01 |
| ENSG00000136003 | 1.53e-04 | 4.80e-04 |
| ENSG00000136010 | 4.49e-01 | 5.32e-01 |
| ENSG00000136011 | 9.19e-17 | 2.76e-15 |
| ENSG00000136014 | 2.36e-06 | 1.04e-05 |
| ENSG00000136021 | 3.15e-04 | 9.28e-04 |
| ENSG00000136026 | 1.27e-01 | 1.85e-01 |
| ENSG00000136040 | 1.77e-03 | 4.40e-03 |
| ENSG00000136044 | 3.79e-20 | 2.57e-18 |
| ENSG00000136045 | 4.64e-15 | 9.68e-14 |
| ENSG00000136048 | 3.08e-11 | 3.16e-10 |
| ENSG00000136051 | 2.88e-01 | 3.68e-01 |
| ENSG00000136052 | 4.06e-15 | 8.59e-14 |
| ENSG00000136059 | 6.74e-14 | 1.13e-12 |
| ENSG00000136068 | 1.12e-08 | 7.40e-08 |
| ENSG00000136098 | 4.47e-06 | 1.88e-05 |
| ENSG00000136099 | 5.37e-06 | 2.23e-05 |
| ENSG00000136100 | 5.73e-01 | 6.48e-01 |
| ENSG00000136104 | 1.55e-10 | 1.41e-09 |
| ENSG00000136108 | 7.44e-18 | 2.82e-16 |
| ENSG00000136110 | 1.87e-02 | 3.52e-02 |
| ENSG00000136111 | 5.09e-12 | 6.02e-11 |
| ENSG00000136114 | 6.50e-02 | 1.04e-01 |
| ENSG00000136122 | 2.19e-10 | 1.94e-09 |
| ENSG00000136141 | 1.11e-12 | 1.48e-11 |
| ENSG00000136143 | 2.07e-01 | 2.79e-01 |
| ENSG00000136144 | 8.25e-20 | 5.12e-18 |
| ENSG00000136146 | 1.30e-02 | 2.57e-02 |
| ENSG00000136147 | 1.32e-04 | 4.20e-04 |
| ENSG00000136149 | 2.19e-01 | 2.92e-01 |
| ENSG00000136152 | 2.86e-03 | 6.77e-03 |
| ENSG00000136153 | 3.29e-02 | 5.76e-02 |
| ENSG00000136155 | 9.41e-02 | 1.43e-01 |
| ENSG00000136156 | 6.58e-10 | 5.35e-09 |
| ENSG00000136158 | 1.09e-02 | 2.19e-02 |
| ENSG00000136159 | 2.37e-02 | 4.33e-02 |
| ENSG00000136160 | 1.38e-12 | 1.83e-11 |
| ENSG00000136161 | 2.08e-03 | 5.09e-03 |
| ENSG00000136167 | 4.92e-05 | 1.71e-04 |
| ENSG00000136169 | 2.81e-02 | 5.03e-02 |
| ENSG00000136193 | 5.20e-03 | 1.15e-02 |
| ENSG00000136197 | 5.98e-04 | 1.66e-03 |
| ENSG00000136205 | 1.29e-01 | 1.87e-01 |
| ENSG00000136206 | 3.31e-01 | 4.13e-01 |
| ENSG00000136213 | 2.99e-01 | 3.80e-01 |
| ENSG00000136231 | 7.70e-05 | 2.56e-04 |
| ENSG00000136235 | 6.63e-06 | 2.70e-05 |
| ENSG00000136237 | 5.77e-01 | 6.53e-01 |
| ENSG00000136238 | 1.04e-01 | 1.55e-01 |
| ENSG00000136240 | 8.12e-04 | 2.19e-03 |
| ENSG00000136243 | 5.04e-12 | 5.96e-11 |
| ENSG00000136244 | 4.32e-06 | 1.82e-05 |
| ENSG00000136247 | 1.38e-09 | 1.07e-08 |
| ENSG00000136250 | 6.09e-01 | 6.81e-01 |
| ENSG00000136261 | 8.54e-13 | 1.17e-11 |
| ENSG00000136267 | 8.57e-07 | 4.11e-06 |
| ENSG00000136270 | 1.25e-12 | 1.66e-11 |
| ENSG00000136271 | 1.38e-20 | 1.07e-18 |
| ENSG00000136273 | 5.74e-01 | 6.49e-01 |
| ENSG00000136274 | 4.81e-15 | 9.99e-14 |
| ENSG00000136275 | 2.38e-12 | 3.00e-11 |
| ENSG00000136279 | 3.84e-03 | 8.77e-03 |
| ENSG00000136280 | 6.29e-02 | 1.01e-01 |
| ENSG00000136286 | 3.73e-02 | 6.43e-02 |
| ENSG00000136295 | 6.42e-24 | 1.47e-21 |
| ENSG00000136297 | 1.62e-07 | 8.78e-07 |
| ENSG00000136305 | 1.25e-08 | 8.19e-08 |
| ENSG00000136315 | 7.25e-01 | 7.83e-01 |
| ENSG00000136319 | 2.27e-05 | 8.40e-05 |
| ENSG00000136327 | 1.37e-03 | 3.50e-03 |
| ENSG00000136352 | 1.33e-05 | 5.15e-05 |
| ENSG00000136367 | 8.07e-02 | 1.25e-01 |
| ENSG00000136371 | 6.51e-01 | 7.19e-01 |
| ENSG00000136378 | 1.89e-09 | 1.43e-08 |
| ENSG00000136379 | 9.11e-05 | 2.99e-04 |
| ENSG00000136381 | 4.74e-02 | 7.91e-02 |
| ENSG00000136383 | 1.17e-04 | 3.77e-04 |
| ENSG00000136404 | 8.85e-04 | 2.37e-03 |
| ENSG00000136425 | 9.79e-06 | 3.88e-05 |
| ENSG00000136436 | 4.13e-15 | 8.73e-14 |
| ENSG00000136444 | 3.90e-01 | 4.73e-01 |
| ENSG00000136448 | 1.99e-07 | 1.07e-06 |
| ENSG00000136449 | 6.33e-01 | 7.03e-01 |
| ENSG00000136450 | 5.14e-07 | 2.55e-06 |
| ENSG00000136451 | 5.20e-01 | 5.99e-01 |
| ENSG00000136457 | 3.67e-15 | 7.87e-14 |
| ENSG00000136463 | 3.58e-05 | 1.28e-04 |
| ENSG00000136478 | 5.97e-01 | 6.70e-01 |
| ENSG00000136485 | 1.78e-02 | 3.37e-02 |
| ENSG00000136490 | 7.01e-03 | 1.49e-02 |
| ENSG00000136492 | 1.22e-09 | 9.49e-09 |
| ENSG00000136504 | 1.11e-01 | 1.65e-01 |
| ENSG00000136514 | 3.53e-02 | 6.13e-02 |
| ENSG00000136518 | 1.31e-16 | 3.81e-15 |
| ENSG00000136521 | 3.70e-04 | 1.07e-03 |
| ENSG00000136522 | 6.06e-02 | 9.78e-02 |
| ENSG00000136527 | 1.29e-06 | 5.98e-06 |
| ENSG00000136531 | 3.09e-01 | 3.90e-01 |
| ENSG00000136535 | 1.04e-01 | 1.55e-01 |
| ENSG00000136536 | 1.24e-01 | 1.81e-01 |
| ENSG00000136541 | 1.89e-01 | 2.58e-01 |
| ENSG00000136542 | 9.24e-01 | 9.44e-01 |
| ENSG00000136546 | 1.72e-20 | 1.29e-18 |
| ENSG00000136560 | 5.07e-02 | 8.40e-02 |
| ENSG00000136573 | 3.19e-11 | 3.26e-10 |
| ENSG00000136574 | 2.89e-01 | 3.69e-01 |
| ENSG00000136603 | 1.97e-04 | 6.06e-04 |
| ENSG00000136628 | 5.45e-07 | 2.69e-06 |
| ENSG00000136630 | 5.10e-03 | 1.13e-02 |
| ENSG00000136631 | 8.39e-04 | 2.26e-03 |
| ENSG00000136634 | 7.74e-07 | 3.73e-06 |
| ENSG00000136636 | 1.17e-01 | 1.71e-01 |
| ENSG00000136643 | 3.74e-01 | 4.57e-01 |
| ENSG00000136682 | 1.07e-04 | 3.46e-04 |
| ENSG00000136688 | 1.84e-03 | 4.57e-03 |
| ENSG00000136689 | 6.47e-09 | 4.46e-08 |
| ENSG00000136694 | 1.61e-01 | 2.26e-01 |
| ENSG00000136695 | 5.08e-08 | 3.02e-07 |
| ENSG00000136696 | 5.85e-03 | 1.27e-02 |
| ENSG00000136698 | 4.75e-04 | 1.35e-03 |
| ENSG00000136699 | 2.21e-11 | 2.32e-10 |
| ENSG00000136709 | 9.68e-03 | 1.98e-02 |
| ENSG00000136710 | 1.83e-03 | 4.54e-03 |
| ENSG00000136715 | 1.69e-01 | 2.35e-01 |
| ENSG00000136717 | 2.91e-02 | 5.18e-02 |
| ENSG00000136718 | 1.57e-11 | 1.68e-10 |
| ENSG00000136720 | 5.70e-03 | 1.24e-02 |
| ENSG00000136731 | 1.30e-07 | 7.22e-07 |
| ENSG00000136732 | 3.84e-13 | 5.61e-12 |
| ENSG00000136738 | 5.94e-09 | 4.12e-08 |
| ENSG00000136750 | 3.34e-01 | 4.16e-01 |
| ENSG00000136754 | 8.00e-01 | 8.46e-01 |
| ENSG00000136758 | 9.95e-01 | 9.96e-01 |
| ENSG00000136770 | 1.06e-03 | 2.80e-03 |
| ENSG00000136783 | 7.26e-07 | 3.52e-06 |
| ENSG00000136802 | 3.69e-06 | 1.57e-05 |
| ENSG00000136807 | 3.46e-02 | 6.03e-02 |
| ENSG00000136810 | 7.73e-01 | 8.23e-01 |
| ENSG00000136811 | 1.92e-15 | 4.30e-14 |
| ENSG00000136813 | 2.11e-01 | 2.83e-01 |
| ENSG00000136816 | 7.77e-03 | 1.63e-02 |
| ENSG00000136819 | 9.01e-01 | 9.27e-01 |
| ENSG00000136824 | 7.46e-07 | 3.61e-06 |
| ENSG00000136826 | 3.04e-23 | 5.40e-21 |
| ENSG00000136827 | 1.16e-02 | 2.31e-02 |
| ENSG00000136828 | 6.35e-03 | 1.37e-02 |
| ENSG00000136830 | 3.09e-01 | 3.90e-01 |
| ENSG00000136834 | 1.01e-03 | 2.67e-03 |
| ENSG00000136840 | 3.46e-01 | 4.29e-01 |
| ENSG00000136842 | 1.18e-13 | 1.88e-12 |
| ENSG00000136848 | 1.01e-08 | 6.73e-08 |
| ENSG00000136854 | 4.59e-12 | 5.47e-11 |
| ENSG00000136856 | 4.55e-07 | 2.28e-06 |
| ENSG00000136859 | 9.89e-02 | 1.49e-01 |
| ENSG00000136861 | 4.79e-03 | 1.07e-02 |
| ENSG00000136866 | 7.40e-03 | 1.56e-02 |
| ENSG00000136867 | 3.25e-07 | 1.68e-06 |
| ENSG00000136868 | 4.77e-01 | 5.59e-01 |
| ENSG00000136869 | 4.48e-01 | 5.31e-01 |
| ENSG00000136870 | 5.04e-02 | 8.34e-02 |
| ENSG00000136872 | 3.75e-03 | 8.61e-03 |
| ENSG00000136874 | 4.04e-01 | 4.87e-01 |
| ENSG00000136875 | 5.77e-21 | 5.00e-19 |
| ENSG00000136877 | 9.08e-13 | 1.24e-11 |
| ENSG00000136878 | 1.95e-01 | 2.65e-01 |
| ENSG00000136881 | 2.01e-11 | 2.12e-10 |
| ENSG00000136883 | 1.71e-07 | 9.25e-07 |
| ENSG00000136888 | 2.51e-04 | 7.55e-04 |
| ENSG00000136891 | 7.63e-19 | 3.73e-17 |
| ENSG00000136895 | 3.95e-01 | 4.78e-01 |
| ENSG00000136897 | 1.31e-08 | 8.55e-08 |
| ENSG00000136908 | 1.09e-16 | 3.20e-15 |
| ENSG00000136918 | 1.60e-02 | 3.07e-02 |
| ENSG00000136925 | 4.05e-04 | 1.17e-03 |
| ENSG00000136928 | 2.35e-02 | 4.30e-02 |
| ENSG00000136929 | 1.25e-08 | 8.22e-08 |
| ENSG00000136930 | 4.40e-08 | 2.64e-07 |
| ENSG00000136931 | 1.57e-03 | 3.96e-03 |
| ENSG00000136932 | 2.77e-01 | 3.56e-01 |
| ENSG00000136933 | 1.58e-14 | 2.99e-13 |
| ENSG00000136935 | 5.72e-05 | 1.96e-04 |
| ENSG00000136936 | 2.71e-01 | 3.49e-01 |
| ENSG00000136937 | 2.22e-04 | 6.76e-04 |
| ENSG00000136938 | 3.09e-09 | 2.25e-08 |
| ENSG00000136939 | 3.47e-01 | 4.29e-01 |
| ENSG00000136940 | 8.42e-02 | 1.30e-01 |
| ENSG00000136942 | 2.86e-08 | 1.77e-07 |
| ENSG00000136943 | 7.90e-08 | 4.53e-07 |
| ENSG00000136944 | 6.58e-02 | 1.05e-01 |
| ENSG00000136950 | 2.01e-08 | 1.28e-07 |
| ENSG00000136960 | 6.31e-15 | 1.28e-13 |
| ENSG00000136982 | 5.08e-20 | 3.29e-18 |
| ENSG00000136986 | 2.26e-01 | 2.99e-01 |
| ENSG00000136997 | 2.60e-18 | 1.10e-16 |
| ENSG00000136999 | 2.42e-06 | 1.06e-05 |
| ENSG00000137033 | 2.63e-01 | 3.40e-01 |
| ENSG00000137038 | 7.33e-03 | 1.55e-02 |
| ENSG00000137040 | 1.72e-04 | 5.35e-04 |
| ENSG00000137054 | 1.04e-17 | 3.80e-16 |
| ENSG00000137055 | 1.26e-05 | 4.90e-05 |
| ENSG00000137070 | 6.20e-09 | 4.29e-08 |
| ENSG00000137073 | 8.04e-16 | 1.96e-14 |
| ENSG00000137074 | 2.35e-10 | 2.07e-09 |
| ENSG00000137075 | 2.16e-01 | 2.89e-01 |
| ENSG00000137076 | 1.03e-03 | 2.71e-03 |
| ENSG00000137077 | 2.46e-11 | 2.56e-10 |
| ENSG00000137078 | 4.12e-04 | 1.18e-03 |
| ENSG00000137080 | 1.83e-01 | 2.51e-01 |
| ENSG00000137090 | 5.18e-06 | 2.15e-05 |
| ENSG00000137094 | 1.51e-07 | 8.25e-07 |
| ENSG00000137098 | 3.01e-01 | 3.82e-01 |
| ENSG00000137100 | 1.55e-01 | 2.18e-01 |
| ENSG00000137101 | 9.26e-01 | 9.45e-01 |
| ENSG00000137103 | 5.46e-01 | 6.24e-01 |
| ENSG00000137106 | 2.33e-02 | 4.27e-02 |
| ENSG00000137124 | 5.72e-09 | 3.98e-08 |
| ENSG00000137133 | 1.88e-04 | 5.82e-04 |
| ENSG00000137135 | 1.43e-16 | 4.12e-15 |
| ENSG00000137142 | 8.99e-01 | 9.25e-01 |
| ENSG00000137145 | 6.03e-01 | 6.75e-01 |
| ENSG00000137154 | 3.82e-06 | 1.63e-05 |
| ENSG00000137161 | 1.63e-09 | 1.24e-08 |
| ENSG00000137166 | 3.28e-17 | 1.08e-15 |
| ENSG00000137168 | 4.29e-21 | 3.87e-19 |
| ENSG00000137171 | 1.46e-10 | 1.32e-09 |
| ENSG00000137177 | 4.98e-03 | 1.10e-02 |
| ENSG00000137185 | 5.06e-02 | 8.38e-02 |
| ENSG00000137193 | 1.59e-02 | 3.06e-02 |
| ENSG00000137198 | 4.56e-05 | 1.59e-04 |
| ENSG00000137200 | 7.24e-07 | 3.51e-06 |
| ENSG00000137203 | 4.59e-14 | 7.90e-13 |
| ENSG00000137204 | 2.46e-01 | 3.22e-01 |
| ENSG00000137207 | 9.17e-01 | 9.38e-01 |
| ENSG00000137210 | 3.87e-01 | 4.70e-01 |
| ENSG00000137216 | 2.60e-06 | 1.14e-05 |
| ENSG00000137218 | 4.63e-05 | 1.62e-04 |
| ENSG00000137221 | 2.57e-11 | 2.66e-10 |
| ENSG00000137225 | 8.05e-01 | 8.51e-01 |
| ENSG00000137251 | 3.15e-10 | 2.71e-09 |
| ENSG00000137252 | 2.30e-01 | 3.04e-01 |
| ENSG00000137261 | 8.18e-01 | 8.61e-01 |
| ENSG00000137265 | 3.89e-16 | 1.02e-14 |
| ENSG00000137266 | 2.50e-15 | 5.51e-14 |
| ENSG00000137267 | 2.34e-05 | 8.65e-05 |
| ENSG00000137269 | 1.32e-15 | 3.07e-14 |
| ENSG00000137270 | 1.07e-06 | 5.03e-06 |
| ENSG00000137273 | 1.08e-16 | 3.19e-15 |
| ENSG00000137274 | 2.29e-03 | 5.54e-03 |
| ENSG00000137275 | 1.08e-10 | 1.00e-09 |
| ENSG00000137285 | 2.07e-07 | 1.10e-06 |
| ENSG00000137288 | 6.50e-08 | 3.78e-07 |
| ENSG00000137309 | 2.54e-17 | 8.60e-16 |
| ENSG00000137310 | 1.72e-10 | 1.55e-09 |
| ENSG00000137312 | 8.45e-01 | 8.83e-01 |
| ENSG00000137331 | 1.36e-08 | 8.85e-08 |
| ENSG00000137337 | 5.97e-08 | 3.50e-07 |
| ENSG00000137338 | 3.24e-01 | 4.05e-01 |
| ENSG00000137343 | 4.09e-14 | 7.13e-13 |
| ENSG00000137364 | 5.33e-08 | 3.16e-07 |
| ENSG00000137392 | 4.53e-01 | 5.36e-01 |
| ENSG00000137393 | 1.87e-02 | 3.53e-02 |
| ENSG00000137404 | 2.18e-07 | 1.16e-06 |
| ENSG00000137409 | 2.82e-01 | 3.61e-01 |
| ENSG00000137411 | 1.70e-06 | 7.66e-06 |
| ENSG00000137413 | 2.63e-06 | 1.15e-05 |
| ENSG00000137414 | 2.93e-08 | 1.81e-07 |
| ENSG00000137434 | 4.74e-01 | 5.56e-01 |
| ENSG00000137440 | 7.09e-08 | 4.10e-07 |
| ENSG00000137441 | 1.65e-16 | 4.68e-15 |
| ENSG00000137449 | 3.27e-10 | 2.80e-09 |
| ENSG00000137460 | 8.61e-01 | 8.95e-01 |
| ENSG00000137462 | 1.23e-03 | 3.19e-03 |
| ENSG00000137463 | 1.07e-04 | 3.48e-04 |
| ENSG00000137473 | 2.86e-02 | 5.09e-02 |
| ENSG00000137474 | 1.78e-09 | 1.35e-08 |
| ENSG00000137478 | 7.22e-01 | 7.81e-01 |
| ENSG00000137486 | 1.30e-15 | 3.03e-14 |
| ENSG00000137491 | 2.75e-03 | 6.54e-03 |
| ENSG00000137492 | 8.84e-01 | 9.14e-01 |
| ENSG00000137494 | 6.30e-02 | 1.01e-01 |
| ENSG00000137496 | 6.96e-01 | 7.59e-01 |
| ENSG00000137497 | 2.32e-01 | 3.06e-01 |
| ENSG00000137500 | 3.41e-01 | 4.23e-01 |
| ENSG00000137501 | 3.09e-11 | 3.16e-10 |
| ENSG00000137502 | 1.86e-09 | 1.40e-08 |
| ENSG00000137504 | 6.12e-13 | 8.57e-12 |
| ENSG00000137507 | 3.01e-05 | 1.09e-04 |
| ENSG00000137509 | 9.88e-01 | 9.92e-01 |
| ENSG00000137513 | 7.70e-09 | 5.24e-08 |
| ENSG00000137522 | 3.43e-01 | 4.25e-01 |
| ENSG00000137547 | 4.31e-03 | 9.71e-03 |
| ENSG00000137558 | 4.27e-06 | 1.80e-05 |
| ENSG00000137561 | 5.70e-02 | 9.27e-02 |
| ENSG00000137563 | 2.08e-04 | 6.36e-04 |
| ENSG00000137571 | 3.06e-09 | 2.23e-08 |
| ENSG00000137573 | 6.83e-14 | 1.14e-12 |
| ENSG00000137574 | 1.73e-15 | 3.91e-14 |
| ENSG00000137575 | 9.54e-02 | 1.44e-01 |
| ENSG00000137601 | 1.44e-05 | 5.54e-05 |
| ENSG00000137628 | 1.76e-09 | 1.33e-08 |
| ENSG00000137634 | 1.18e-13 | 1.89e-12 |
| ENSG00000137642 | 3.11e-02 | 5.49e-02 |
| ENSG00000137648 | 1.01e-01 | 1.52e-01 |
| ENSG00000137656 | 2.86e-02 | 5.11e-02 |
| ENSG00000137672 | 6.53e-06 | 2.66e-05 |
| ENSG00000137673 | 6.47e-25 | 2.03e-22 |
| ENSG00000137674 | 3.43e-13 | 5.04e-12 |
| ENSG00000137675 | 3.27e-19 | 1.74e-17 |
| ENSG00000137691 | 5.26e-01 | 6.05e-01 |
| ENSG00000137692 | 2.77e-20 | 1.95e-18 |
| ENSG00000137693 | 9.10e-08 | 5.17e-07 |
| ENSG00000137699 | 2.09e-22 | 2.78e-20 |
| ENSG00000137700 | 9.96e-01 | 9.97e-01 |
| ENSG00000137707 | 2.25e-01 | 2.99e-01 |
| ENSG00000137709 | 1.40e-02 | 2.73e-02 |
| ENSG00000137710 | 4.67e-05 | 1.63e-04 |
| ENSG00000137713 | 1.84e-01 | 2.52e-01 |
| ENSG00000137714 | 1.42e-05 | 5.47e-05 |
| ENSG00000137720 | 2.20e-11 | 2.30e-10 |
| ENSG00000137726 | 1.95e-11 | 2.06e-10 |
| ENSG00000137727 | 3.71e-18 | 1.52e-16 |
| ENSG00000137731 | 2.01e-01 | 2.72e-01 |
| ENSG00000137745 | 1.39e-18 | 6.30e-17 |
| ENSG00000137747 | 1.32e-13 | 2.09e-12 |
| ENSG00000137752 | 4.04e-03 | 9.18e-03 |
| ENSG00000137757 | 1.51e-13 | 2.37e-12 |
| ENSG00000137760 | 2.75e-01 | 3.54e-01 |
| ENSG00000137764 | 1.59e-01 | 2.23e-01 |
| ENSG00000137766 | 1.20e-06 | 5.60e-06 |
| ENSG00000137767 | 7.72e-20 | 4.81e-18 |
| ENSG00000137770 | 4.32e-01 | 5.15e-01 |
| ENSG00000137776 | 5.85e-01 | 6.59e-01 |
| ENSG00000137801 | 4.12e-01 | 4.94e-01 |
| ENSG00000137802 | 5.92e-01 | 6.66e-01 |
| ENSG00000137804 | 3.38e-11 | 3.44e-10 |
| ENSG00000137806 | 1.46e-02 | 2.83e-02 |
| ENSG00000137807 | 1.87e-15 | 4.20e-14 |
| ENSG00000137809 | 5.69e-14 | 9.64e-13 |
| ENSG00000137812 | 1.58e-07 | 8.60e-07 |
| ENSG00000137814 | 4.57e-08 | 2.74e-07 |
| ENSG00000137815 | 2.81e-02 | 5.03e-02 |
| ENSG00000137817 | 4.31e-04 | 1.23e-03 |
| ENSG00000137818 | 7.78e-03 | 1.64e-02 |
| ENSG00000137819 | 1.06e-18 | 4.96e-17 |
| ENSG00000137821 | 2.87e-02 | 5.11e-02 |
| ENSG00000137822 | 6.49e-08 | 3.78e-07 |
| ENSG00000137824 | 4.72e-01 | 5.54e-01 |
| ENSG00000137825 | 2.22e-10 | 1.96e-09 |
| ENSG00000137831 | 2.78e-01 | 3.57e-01 |
| ENSG00000137834 | 2.64e-12 | 3.30e-11 |
| ENSG00000137841 | 2.01e-01 | 2.72e-01 |
| ENSG00000137842 | 1.22e-03 | 3.16e-03 |
| ENSG00000137843 | 5.10e-03 | 1.13e-02 |
| ENSG00000137845 | 1.55e-01 | 2.18e-01 |
| ENSG00000137857 | 3.35e-03 | 7.77e-03 |
| ENSG00000137860 | 6.40e-08 | 3.73e-07 |
| ENSG00000137868 | 1.19e-23 | 2.49e-21 |
| ENSG00000137869 | 3.47e-07 | 1.78e-06 |
| ENSG00000137871 | 9.88e-01 | 9.92e-01 |
| ENSG00000137872 | 5.74e-21 | 4.99e-19 |
| ENSG00000137875 | 3.59e-07 | 1.84e-06 |
| ENSG00000137876 | 6.00e-03 | 1.30e-02 |
| ENSG00000137877 | 3.52e-14 | 6.19e-13 |
| ENSG00000137878 | 2.14e-10 | 1.89e-09 |
| ENSG00000137880 | 9.05e-01 | 9.29e-01 |
| ENSG00000137936 | 1.29e-21 | 1.43e-19 |
| ENSG00000137941 | 7.49e-12 | 8.56e-11 |
| ENSG00000137942 | 8.17e-02 | 1.26e-01 |
| ENSG00000137944 | 8.31e-01 | 8.71e-01 |
| ENSG00000137947 | 1.07e-03 | 2.81e-03 |
| ENSG00000137948 | 1.53e-04 | 4.81e-04 |
| ENSG00000137955 | 1.23e-07 | 6.84e-07 |
| ENSG00000137959 | 7.77e-01 | 8.27e-01 |
| ENSG00000137960 | 2.51e-06 | 1.10e-05 |
| ENSG00000137962 | 5.24e-01 | 6.03e-01 |
| ENSG00000137965 | 7.84e-04 | 2.12e-03 |
| ENSG00000137968 | 3.23e-01 | 4.04e-01 |
| ENSG00000137970 | 2.87e-01 | 3.67e-01 |
| ENSG00000137975 | 4.43e-10 | 3.72e-09 |
| ENSG00000137976 | 3.04e-04 | 8.98e-04 |
| ENSG00000137992 | 8.64e-07 | 4.14e-06 |
| ENSG00000137996 | 7.83e-01 | 8.33e-01 |
| ENSG00000138002 | 1.61e-02 | 3.08e-02 |
| ENSG00000138018 | 3.78e-06 | 1.61e-05 |
| ENSG00000138028 | 7.51e-27 | 5.42e-24 |
| ENSG00000138029 | 4.64e-21 | 4.12e-19 |
| ENSG00000138030 | 7.73e-05 | 2.57e-04 |
| ENSG00000138031 | 1.41e-14 | 2.70e-13 |
| ENSG00000138032 | 1.78e-07 | 9.62e-07 |
| ENSG00000138035 | 5.75e-16 | 1.45e-14 |
| ENSG00000138036 | 3.42e-01 | 4.24e-01 |
| ENSG00000138039 | 4.19e-01 | 5.02e-01 |
| ENSG00000138050 | 2.66e-17 | 8.95e-16 |
| ENSG00000138061 | 1.15e-03 | 3.00e-03 |
| ENSG00000138069 | 5.26e-01 | 6.05e-01 |
| ENSG00000138071 | 4.02e-02 | 6.86e-02 |
| ENSG00000138073 | 1.64e-11 | 1.75e-10 |
| ENSG00000138074 | 9.25e-20 | 5.64e-18 |
| ENSG00000138075 | 4.52e-03 | 1.01e-02 |
| ENSG00000138078 | 3.69e-01 | 4.51e-01 |
| ENSG00000138079 | 1.11e-06 | 5.19e-06 |
| ENSG00000138080 | 5.62e-02 | 9.16e-02 |
| ENSG00000138081 | 3.88e-01 | 4.71e-01 |
| ENSG00000138083 | 3.70e-03 | 8.50e-03 |
| ENSG00000138085 | 3.41e-02 | 5.95e-02 |
| ENSG00000138092 | 2.75e-15 | 6.01e-14 |
| ENSG00000138095 | 5.98e-04 | 1.67e-03 |
| ENSG00000138100 | 3.14e-02 | 5.53e-02 |
| ENSG00000138101 | 2.83e-15 | 6.18e-14 |
| ENSG00000138107 | 1.58e-06 | 7.20e-06 |
| ENSG00000138109 | 3.48e-02 | 6.06e-02 |
| ENSG00000138111 | 7.78e-07 | 3.75e-06 |
| ENSG00000138115 | 1.60e-03 | 4.03e-03 |
| ENSG00000138119 | 6.26e-01 | 6.96e-01 |
| ENSG00000138131 | 2.02e-07 | 1.08e-06 |
| ENSG00000138134 | 5.94e-12 | 6.93e-11 |
| ENSG00000138135 | 1.74e-01 | 2.41e-01 |
| ENSG00000138136 | 4.55e-03 | 1.02e-02 |
| ENSG00000138138 | 9.93e-01 | 9.95e-01 |
| ENSG00000138152 | 2.50e-20 | 1.79e-18 |
| ENSG00000138160 | 5.80e-08 | 3.41e-07 |
| ENSG00000138161 | 5.25e-01 | 6.04e-01 |
| ENSG00000138162 | 7.31e-05 | 2.45e-04 |
| ENSG00000138166 | 8.33e-05 | 2.76e-04 |
| ENSG00000138172 | 5.09e-03 | 1.12e-02 |
| ENSG00000138175 | 9.70e-05 | 3.17e-04 |
| ENSG00000138180 | 2.38e-15 | 5.26e-14 |
| ENSG00000138182 | 3.41e-07 | 1.75e-06 |
| ENSG00000138185 | 5.07e-02 | 8.40e-02 |
| ENSG00000138190 | 1.63e-01 | 2.27e-01 |
| ENSG00000138193 | 7.27e-23 | 1.15e-20 |
| ENSG00000138207 | 1.06e-02 | 2.14e-02 |
| ENSG00000138231 | 1.27e-10 | 1.17e-09 |
| ENSG00000138246 | 6.30e-05 | 2.13e-04 |
| ENSG00000138271 | 4.07e-04 | 1.17e-03 |
| ENSG00000138279 | 1.65e-02 | 3.15e-02 |
| ENSG00000138286 | 6.36e-01 | 7.06e-01 |
| ENSG00000138303 | 1.97e-08 | 1.25e-07 |
| ENSG00000138308 | 4.85e-02 | 8.07e-02 |
| ENSG00000138311 | 3.00e-01 | 3.80e-01 |
| ENSG00000138315 | 5.37e-01 | 6.16e-01 |
| ENSG00000138316 | 4.89e-10 | 4.07e-09 |
| ENSG00000138326 | 8.67e-02 | 1.33e-01 |
| ENSG00000138336 | 9.01e-01 | 9.26e-01 |
| ENSG00000138346 | 3.27e-13 | 4.83e-12 |
| ENSG00000138347 | 1.06e-13 | 1.71e-12 |
| ENSG00000138356 | 1.86e-10 | 1.67e-09 |
| ENSG00000138363 | 5.25e-20 | 3.39e-18 |
| ENSG00000138375 | 9.02e-05 | 2.96e-04 |
| ENSG00000138376 | 1.49e-09 | 1.14e-08 |
| ENSG00000138378 | 1.48e-05 | 5.68e-05 |
| ENSG00000138379 | 6.61e-10 | 5.37e-09 |
| ENSG00000138380 | 6.71e-01 | 7.37e-01 |
| ENSG00000138381 | 3.68e-06 | 1.57e-05 |
| ENSG00000138382 | 4.46e-14 | 7.72e-13 |
| ENSG00000138385 | 8.59e-09 | 5.79e-08 |
| ENSG00000138386 | 2.99e-05 | 1.08e-04 |
| ENSG00000138395 | 5.56e-08 | 3.28e-07 |
| ENSG00000138398 | 2.84e-01 | 3.63e-01 |
| ENSG00000138399 | 7.55e-04 | 2.05e-03 |
| ENSG00000138400 | 2.90e-01 | 3.70e-01 |
| ENSG00000138411 | 8.84e-15 | 1.75e-13 |
| ENSG00000138413 | 1.31e-02 | 2.59e-02 |
| ENSG00000138430 | 8.62e-15 | 1.71e-13 |
| ENSG00000138433 | 3.68e-01 | 4.50e-01 |
| ENSG00000138434 | 3.91e-09 | 2.80e-08 |
| ENSG00000138435 | 6.00e-10 | 4.91e-09 |
| ENSG00000138439 | 8.75e-01 | 9.07e-01 |
| ENSG00000138442 | 1.47e-20 | 1.13e-18 |
| ENSG00000138443 | 1.06e-03 | 2.79e-03 |
| ENSG00000138448 | 4.34e-02 | 7.33e-02 |
| ENSG00000138449 | 5.26e-05 | 1.81e-04 |
| ENSG00000138459 | 1.34e-04 | 4.26e-04 |
| ENSG00000138463 | 4.26e-10 | 3.59e-09 |
| ENSG00000138468 | 2.56e-03 | 6.14e-03 |
| ENSG00000138483 | 8.18e-01 | 8.61e-01 |
| ENSG00000138495 | 2.54e-02 | 4.61e-02 |
| ENSG00000138496 | 7.57e-02 | 1.18e-01 |
| ENSG00000138587 | 8.92e-02 | 1.36e-01 |
| ENSG00000138592 | 3.04e-03 | 7.14e-03 |
| ENSG00000138593 | 1.78e-09 | 1.34e-08 |
| ENSG00000138594 | 4.65e-05 | 1.62e-04 |
| ENSG00000138600 | 1.87e-20 | 1.38e-18 |
| ENSG00000138604 | 5.41e-06 | 2.24e-05 |
| ENSG00000138606 | 1.60e-03 | 4.04e-03 |
| ENSG00000138613 | 2.58e-01 | 3.35e-01 |
| ENSG00000138614 | 6.10e-07 | 2.99e-06 |
| ENSG00000138615 | 3.79e-14 | 6.64e-13 |
| ENSG00000138617 | 6.40e-01 | 7.09e-01 |
| ENSG00000138621 | 6.68e-02 | 1.06e-01 |
| ENSG00000138622 | 5.41e-08 | 3.20e-07 |
| ENSG00000138623 | 7.08e-03 | 1.50e-02 |
| ENSG00000138629 | 1.38e-01 | 1.98e-01 |
| ENSG00000138639 | 5.61e-04 | 1.57e-03 |
| ENSG00000138640 | 2.21e-02 | 4.09e-02 |
| ENSG00000138641 | 3.91e-11 | 3.93e-10 |
| ENSG00000138642 | 8.37e-01 | 8.77e-01 |
| ENSG00000138646 | 1.64e-04 | 5.13e-04 |
| ENSG00000138650 | 3.71e-15 | 7.95e-14 |
| ENSG00000138653 | 3.26e-06 | 1.40e-05 |
| ENSG00000138658 | 1.70e-07 | 9.20e-07 |
| ENSG00000138660 | 1.48e-01 | 2.10e-01 |
| ENSG00000138663 | 7.14e-02 | 1.13e-01 |
| ENSG00000138668 | 2.86e-11 | 2.95e-10 |
| ENSG00000138669 | 1.41e-22 | 2.01e-20 |
| ENSG00000138670 | 7.85e-01 | 8.33e-01 |
| ENSG00000138674 | 1.17e-01 | 1.72e-01 |
| ENSG00000138675 | 6.18e-01 | 6.89e-01 |
| ENSG00000138678 | 3.81e-26 | 1.95e-23 |
| ENSG00000138684 | 4.98e-01 | 5.78e-01 |
| ENSG00000138685 | 2.24e-08 | 1.41e-07 |
| ENSG00000138686 | 3.20e-01 | 4.01e-01 |
| ENSG00000138688 | 1.71e-06 | 7.70e-06 |
| ENSG00000138696 | 3.55e-01 | 4.38e-01 |
| ENSG00000138698 | 5.45e-06 | 2.25e-05 |
| ENSG00000138709 | 5.13e-01 | 5.93e-01 |
| ENSG00000138722 | 5.79e-19 | 2.91e-17 |
| ENSG00000138735 | 3.42e-16 | 9.07e-15 |
| ENSG00000138738 | 2.56e-01 | 3.32e-01 |
| ENSG00000138741 | 7.21e-01 | 7.80e-01 |
| ENSG00000138744 | 1.09e-23 | 2.31e-21 |
| ENSG00000138750 | 2.28e-03 | 5.54e-03 |
| ENSG00000138755 | 8.27e-05 | 2.74e-04 |
| ENSG00000138756 | 2.44e-05 | 8.97e-05 |
| ENSG00000138757 | 1.25e-01 | 1.82e-01 |
| ENSG00000138758 | 1.81e-01 | 2.49e-01 |
| ENSG00000138759 | 7.01e-03 | 1.49e-02 |
| ENSG00000138760 | 2.91e-08 | 1.80e-07 |
| ENSG00000138764 | 1.34e-10 | 1.22e-09 |
| ENSG00000138767 | 1.28e-01 | 1.85e-01 |
| ENSG00000138768 | 9.42e-01 | 9.57e-01 |
| ENSG00000138769 | 1.73e-13 | 2.68e-12 |
| ENSG00000138771 | 2.39e-09 | 1.77e-08 |
| ENSG00000138772 | 2.43e-14 | 4.43e-13 |
| ENSG00000138777 | 1.46e-02 | 2.83e-02 |
| ENSG00000138778 | 6.52e-08 | 3.80e-07 |
| ENSG00000138780 | 5.12e-09 | 3.59e-08 |
| ENSG00000138785 | 2.89e-01 | 3.69e-01 |
| ENSG00000138792 | 2.38e-03 | 5.74e-03 |
| ENSG00000138794 | 4.95e-03 | 1.10e-02 |
| ENSG00000138795 | 5.33e-18 | 2.09e-16 |
| ENSG00000138796 | 2.75e-17 | 9.21e-16 |
| ENSG00000138798 | 4.07e-07 | 2.06e-06 |
| ENSG00000138801 | 1.19e-08 | 7.83e-08 |
| ENSG00000138802 | 2.26e-01 | 3.00e-01 |
| ENSG00000138814 | 7.92e-01 | 8.39e-01 |
| ENSG00000138821 | 9.99e-04 | 2.64e-03 |
| ENSG00000138823 | 3.09e-02 | 5.46e-02 |
| ENSG00000138829 | 1.73e-02 | 3.29e-02 |
| ENSG00000138834 | 1.19e-06 | 5.55e-06 |
| ENSG00000138835 | 1.12e-01 | 1.65e-01 |
| ENSG00000138867 | 1.83e-07 | 9.83e-07 |
| ENSG00000138892 | 4.09e-02 | 6.96e-02 |
| ENSG00000138942 | 8.43e-16 | 2.04e-14 |
| ENSG00000138944 | 3.64e-10 | 3.10e-09 |
| ENSG00000138964 | 1.22e-03 | 3.17e-03 |
| ENSG00000139044 | 2.14e-06 | 9.48e-06 |
| ENSG00000139053 | 5.35e-01 | 6.14e-01 |
| ENSG00000139055 | 2.42e-08 | 1.52e-07 |
| ENSG00000139083 | 1.46e-01 | 2.07e-01 |
| ENSG00000139112 | 7.22e-08 | 4.17e-07 |
| ENSG00000139116 | 1.08e-01 | 1.61e-01 |
| ENSG00000139117 | 2.71e-19 | 1.46e-17 |
| ENSG00000139131 | 1.15e-12 | 1.53e-11 |
| ENSG00000139132 | 1.18e-07 | 6.56e-07 |
| ENSG00000139133 | 6.14e-03 | 1.33e-02 |
| ENSG00000139144 | 5.08e-09 | 3.57e-08 |
| ENSG00000139146 | 7.23e-15 | 1.45e-13 |
| ENSG00000139151 | 4.93e-01 | 5.74e-01 |
| ENSG00000139154 | 1.82e-07 | 9.80e-07 |
| ENSG00000139155 | 7.04e-08 | 4.07e-07 |
| ENSG00000139160 | 4.84e-08 | 2.88e-07 |
| ENSG00000139163 | 8.32e-03 | 1.74e-02 |
| ENSG00000139168 | 3.80e-01 | 4.63e-01 |
| ENSG00000139173 | 1.87e-03 | 4.64e-03 |
| ENSG00000139174 | 5.28e-05 | 1.82e-04 |
| ENSG00000139178 | 1.15e-01 | 1.70e-01 |
| ENSG00000139180 | 1.24e-01 | 1.80e-01 |
| ENSG00000139182 | 3.68e-10 | 3.13e-09 |
| ENSG00000139187 | 5.09e-06 | 2.12e-05 |
| ENSG00000139190 | 4.26e-03 | 9.61e-03 |
| ENSG00000139192 | 5.07e-04 | 1.43e-03 |
| ENSG00000139193 | 1.03e-14 | 2.02e-13 |
| ENSG00000139194 | 4.41e-06 | 1.86e-05 |
| ENSG00000139197 | 4.74e-11 | 4.71e-10 |
| ENSG00000139200 | 9.70e-20 | 5.88e-18 |
| ENSG00000139209 | 5.84e-09 | 4.06e-08 |
| ENSG00000139211 | 2.74e-02 | 4.92e-02 |
| ENSG00000139218 | 4.22e-01 | 5.05e-01 |
| ENSG00000139219 | 4.15e-09 | 2.95e-08 |
| ENSG00000139220 | 1.26e-02 | 2.50e-02 |
| ENSG00000139223 | 4.11e-03 | 9.32e-03 |
| ENSG00000139233 | 1.48e-01 | 2.10e-01 |
| ENSG00000139239 | 1.95e-07 | 1.05e-06 |
| ENSG00000139263 | 1.48e-01 | 2.10e-01 |
| ENSG00000139266 | 3.37e-15 | 7.26e-14 |
| ENSG00000139269 | 6.03e-07 | 2.96e-06 |
| ENSG00000139278 | 4.03e-01 | 4.86e-01 |
| ENSG00000139287 | 1.58e-01 | 2.22e-01 |
| ENSG00000139289 | 1.97e-22 | 2.64e-20 |
| ENSG00000139291 | 7.57e-07 | 3.66e-06 |
| ENSG00000139292 | 3.31e-12 | 4.06e-11 |
| ENSG00000139304 | 1.73e-05 | 6.52e-05 |
| ENSG00000139318 | 1.38e-01 | 1.98e-01 |
| ENSG00000139323 | 3.21e-08 | 1.97e-07 |
| ENSG00000139324 | 6.64e-01 | 7.30e-01 |
| ENSG00000139329 | 6.11e-02 | 9.86e-02 |
| ENSG00000139330 | 8.76e-01 | 9.07e-01 |
| ENSG00000139343 | 1.09e-16 | 3.20e-15 |
| ENSG00000139344 | 6.68e-02 | 1.06e-01 |
| ENSG00000139350 | 6.51e-05 | 2.20e-04 |
| ENSG00000139351 | 3.78e-01 | 4.61e-01 |
| ENSG00000139352 | 1.18e-09 | 9.24e-09 |
| ENSG00000139354 | 1.54e-02 | 2.97e-02 |
| ENSG00000139364 | 5.83e-11 | 5.69e-10 |
| ENSG00000139370 | 5.92e-12 | 6.91e-11 |
| ENSG00000139372 | 7.43e-02 | 1.17e-01 |
| ENSG00000139405 | 1.13e-01 | 1.66e-01 |
| ENSG00000139410 | 8.08e-05 | 2.68e-04 |
| ENSG00000139428 | 1.64e-05 | 6.22e-05 |
| ENSG00000139433 | 4.69e-28 | 5.17e-25 |
| ENSG00000139436 | 9.41e-02 | 1.43e-01 |
| ENSG00000139437 | 1.50e-03 | 3.79e-03 |
| ENSG00000139438 | 2.12e-10 | 1.88e-09 |
| ENSG00000139445 | 2.04e-01 | 2.76e-01 |
| ENSG00000139496 | 4.22e-14 | 7.34e-13 |
| ENSG00000139505 | 1.24e-01 | 1.81e-01 |
| ENSG00000139508 | 5.06e-14 | 8.66e-13 |
| ENSG00000139514 | 5.04e-17 | 1.60e-15 |
| ENSG00000139515 | 2.25e-24 | 5.78e-22 |
| ENSG00000139517 | 1.50e-01 | 2.12e-01 |
| ENSG00000139531 | 1.47e-02 | 2.84e-02 |
| ENSG00000139537 | 2.73e-01 | 3.52e-01 |
| ENSG00000139540 | 4.58e-07 | 2.30e-06 |
| ENSG00000139546 | 2.82e-10 | 2.44e-09 |
| ENSG00000139547 | 4.47e-12 | 5.33e-11 |
| ENSG00000139549 | 3.40e-02 | 5.94e-02 |
| ENSG00000139567 | 1.29e-15 | 3.01e-14 |
| ENSG00000139572 | 1.42e-10 | 1.30e-09 |
| ENSG00000139579 | 1.07e-08 | 7.12e-08 |
| ENSG00000139597 | 5.92e-03 | 1.29e-02 |
| ENSG00000139610 | 4.25e-05 | 1.49e-04 |
| ENSG00000139613 | 3.85e-02 | 6.61e-02 |
| ENSG00000139618 | 4.47e-17 | 1.44e-15 |
| ENSG00000139620 | 2.11e-09 | 1.58e-08 |
| ENSG00000139624 | 7.46e-10 | 6.01e-09 |
| ENSG00000139625 | 7.49e-01 | 8.04e-01 |
| ENSG00000139626 | 7.32e-10 | 5.91e-09 |
| ENSG00000139629 | 6.03e-18 | 2.33e-16 |
| ENSG00000139631 | 2.92e-04 | 8.65e-04 |
| ENSG00000139636 | 2.66e-02 | 4.79e-02 |
| ENSG00000139637 | 1.31e-05 | 5.06e-05 |
| ENSG00000139641 | 1.14e-05 | 4.45e-05 |
| ENSG00000139644 | 5.75e-05 | 1.97e-04 |
| ENSG00000139645 | 4.50e-03 | 1.01e-02 |
| ENSG00000139648 | 1.43e-02 | 2.79e-02 |
| ENSG00000139651 | 3.40e-06 | 1.46e-05 |
| ENSG00000139656 | 4.30e-08 | 2.58e-07 |
| ENSG00000139668 | 8.93e-08 | 5.08e-07 |
| ENSG00000139675 | 8.53e-11 | 8.10e-10 |
| ENSG00000139679 | 8.84e-02 | 1.35e-01 |
| ENSG00000139684 | 3.08e-01 | 3.88e-01 |
| ENSG00000139687 | 4.56e-03 | 1.02e-02 |
| ENSG00000139697 | 1.40e-04 | 4.43e-04 |
| ENSG00000139714 | 1.46e-03 | 3.71e-03 |
| ENSG00000139718 | 3.44e-01 | 4.26e-01 |
| ENSG00000139719 | 7.13e-01 | 7.73e-01 |
| ENSG00000139722 | 1.31e-07 | 7.26e-07 |
| ENSG00000139725 | 2.72e-12 | 3.40e-11 |
| ENSG00000139726 | 2.86e-05 | 1.04e-04 |
| ENSG00000139734 | 1.64e-16 | 4.65e-15 |
| ENSG00000139737 | 3.88e-04 | 1.12e-03 |
| ENSG00000139746 | 7.48e-14 | 1.24e-12 |
| ENSG00000139767 | 1.68e-03 | 4.20e-03 |
| ENSG00000139780 | 4.87e-02 | 8.10e-02 |
| ENSG00000139793 | 1.43e-02 | 2.79e-02 |
| ENSG00000139797 | 3.61e-05 | 1.29e-04 |
| ENSG00000139800 | 1.61e-23 | 3.20e-21 |
| ENSG00000139826 | 1.11e-01 | 1.64e-01 |
| ENSG00000139832 | 5.35e-02 | 8.78e-02 |
| ENSG00000139835 | 7.62e-01 | 8.15e-01 |
| ENSG00000139842 | 4.52e-15 | 9.44e-14 |
| ENSG00000139865 | 6.56e-05 | 2.22e-04 |
| ENSG00000139874 | 3.89e-10 | 3.30e-09 |
| ENSG00000139880 | 4.73e-14 | 8.13e-13 |
| ENSG00000139890 | 1.02e-11 | 1.14e-10 |
| ENSG00000139899 | 2.68e-01 | 3.46e-01 |
| ENSG00000139908 | 1.79e-01 | 2.46e-01 |
| ENSG00000139910 | 3.98e-18 | 1.61e-16 |
| ENSG00000139914 | 3.83e-06 | 1.63e-05 |
| ENSG00000139915 | 1.85e-06 | 8.29e-06 |
| ENSG00000139921 | 3.75e-02 | 6.47e-02 |
| ENSG00000139926 | 9.48e-04 | 2.52e-03 |
| ENSG00000139946 | 2.37e-10 | 2.09e-09 |
| ENSG00000139970 | 2.11e-15 | 4.70e-14 |
| ENSG00000139971 | 4.78e-02 | 7.97e-02 |
| ENSG00000139973 | 6.10e-01 | 6.82e-01 |
| ENSG00000139974 | 3.85e-01 | 4.68e-01 |
| ENSG00000139977 | 3.34e-01 | 4.16e-01 |
| ENSG00000139985 | 3.54e-01 | 4.37e-01 |
| ENSG00000139988 | 3.55e-06 | 1.52e-05 |
| ENSG00000139990 | 1.30e-07 | 7.21e-07 |
| ENSG00000139998 | 8.23e-16 | 2.00e-14 |
| ENSG00000140006 | 9.27e-03 | 1.91e-02 |
| ENSG00000140009 | 1.14e-08 | 7.54e-08 |
| ENSG00000140015 | 1.19e-06 | 5.55e-06 |
| ENSG00000140022 | 1.08e-04 | 3.50e-04 |
| ENSG00000140025 | 4.87e-07 | 2.43e-06 |
| ENSG00000140030 | 5.58e-03 | 1.22e-02 |
| ENSG00000140043 | 5.22e-04 | 1.47e-03 |
| ENSG00000140044 | 3.17e-09 | 2.30e-08 |
| ENSG00000140057 | 1.98e-03 | 4.87e-03 |
| ENSG00000140067 | 7.97e-02 | 1.24e-01 |
| ENSG00000140090 | 8.69e-10 | 6.93e-09 |
| ENSG00000140092 | 1.83e-08 | 1.17e-07 |
| ENSG00000140093 | 6.10e-03 | 1.32e-02 |
| ENSG00000140104 | 1.03e-17 | 3.77e-16 |
| ENSG00000140105 | 5.54e-05 | 1.90e-04 |
| ENSG00000140107 | 1.16e-01 | 1.71e-01 |
| ENSG00000140153 | 4.42e-09 | 3.13e-08 |
| ENSG00000140157 | 8.62e-03 | 1.79e-02 |
| ENSG00000140199 | 9.95e-04 | 2.63e-03 |
| ENSG00000140254 | 6.36e-02 | 1.02e-01 |
| ENSG00000140259 | 8.14e-02 | 1.26e-01 |
| ENSG00000140262 | 2.09e-04 | 6.40e-04 |
| ENSG00000140263 | 1.12e-19 | 6.72e-18 |
| ENSG00000140264 | 2.20e-01 | 2.93e-01 |
| ENSG00000140265 | 1.35e-01 | 1.95e-01 |
| ENSG00000140274 | 1.83e-04 | 5.67e-04 |
| ENSG00000140279 | 3.13e-01 | 3.94e-01 |
| ENSG00000140280 | 8.86e-02 | 1.36e-01 |
| ENSG00000140284 | 1.48e-04 | 4.66e-04 |
| ENSG00000140285 | 2.72e-04 | 8.11e-04 |
| ENSG00000140287 | 3.98e-08 | 2.40e-07 |
| ENSG00000140297 | 7.42e-14 | 1.23e-12 |
| ENSG00000140299 | 6.64e-02 | 1.06e-01 |
| ENSG00000140307 | 1.02e-02 | 2.08e-02 |
| ENSG00000140319 | 2.48e-04 | 7.46e-04 |
| ENSG00000140320 | 1.41e-07 | 7.73e-07 |
| ENSG00000140323 | 2.91e-20 | 2.04e-18 |
| ENSG00000140326 | 7.94e-03 | 1.67e-02 |
| ENSG00000140332 | 2.11e-01 | 2.83e-01 |
| ENSG00000140350 | 2.33e-01 | 3.08e-01 |
| ENSG00000140365 | 4.63e-01 | 5.45e-01 |
| ENSG00000140367 | 9.63e-01 | 9.72e-01 |
| ENSG00000140368 | 2.56e-01 | 3.33e-01 |
| ENSG00000140374 | 7.42e-11 | 7.11e-10 |
| ENSG00000140379 | 2.38e-03 | 5.75e-03 |
| ENSG00000140382 | 5.15e-01 | 5.95e-01 |
| ENSG00000140386 | 2.05e-05 | 7.66e-05 |
| ENSG00000140391 | 8.82e-11 | 8.35e-10 |
| ENSG00000140395 | 3.95e-03 | 9.00e-03 |
| ENSG00000140396 | 7.72e-03 | 1.62e-02 |
| ENSG00000140398 | 2.57e-01 | 3.34e-01 |
| ENSG00000140400 | 1.57e-01 | 2.21e-01 |
| ENSG00000140403 | 1.27e-02 | 2.51e-02 |
| ENSG00000140406 | 3.68e-04 | 1.07e-03 |
| ENSG00000140416 | 6.55e-06 | 2.67e-05 |
| ENSG00000140443 | 7.23e-04 | 1.97e-03 |
| ENSG00000140450 | 1.93e-18 | 8.42e-17 |
| ENSG00000140451 | 2.20e-12 | 2.80e-11 |
| ENSG00000140455 | 2.77e-01 | 3.56e-01 |
| ENSG00000140459 | 1.85e-11 | 1.97e-10 |
| ENSG00000140463 | 2.98e-01 | 3.78e-01 |
| ENSG00000140464 | 1.48e-12 | 1.94e-11 |
| ENSG00000140465 | 3.63e-01 | 4.45e-01 |
| ENSG00000140470 | 3.43e-02 | 5.97e-02 |
| ENSG00000140471 | 4.03e-02 | 6.87e-02 |
| ENSG00000140474 | 1.97e-06 | 8.81e-06 |
| ENSG00000140478 | 1.24e-01 | 1.81e-01 |
| ENSG00000140479 | 1.45e-10 | 1.32e-09 |
| ENSG00000140481 | 4.06e-05 | 1.43e-04 |
| ENSG00000140488 | 2.18e-01 | 2.91e-01 |
| ENSG00000140497 | 9.23e-14 | 1.51e-12 |
| ENSG00000140505 | 7.01e-01 | 7.63e-01 |
| ENSG00000140506 | 1.39e-01 | 2.00e-01 |
| ENSG00000140511 | 1.57e-18 | 6.98e-17 |
| ENSG00000140519 | 5.89e-08 | 3.46e-07 |
| ENSG00000140521 | 9.47e-04 | 2.52e-03 |
| ENSG00000140522 | 1.10e-02 | 2.22e-02 |
| ENSG00000140525 | 5.02e-16 | 1.29e-14 |
| ENSG00000140526 | 6.96e-08 | 4.03e-07 |
| ENSG00000140527 | 1.20e-01 | 1.75e-01 |
| ENSG00000140534 | 9.40e-15 | 1.85e-13 |
| ENSG00000140538 | 1.15e-17 | 4.15e-16 |
| ENSG00000140543 | 1.59e-01 | 2.23e-01 |
| ENSG00000140545 | 7.05e-03 | 1.50e-02 |
| ENSG00000140548 | 1.25e-02 | 2.49e-02 |
| ENSG00000140553 | 8.97e-01 | 9.24e-01 |
| ENSG00000140557 | 3.17e-01 | 3.98e-01 |
| ENSG00000140563 | 3.07e-05 | 1.11e-04 |
| ENSG00000140564 | 3.31e-01 | 4.13e-01 |
| ENSG00000140575 | 2.21e-02 | 4.08e-02 |
| ENSG00000140577 | 1.08e-02 | 2.18e-02 |
| ENSG00000140598 | 2.37e-04 | 7.17e-04 |
| ENSG00000140600 | 7.84e-08 | 4.50e-07 |
| ENSG00000140612 | 3.17e-02 | 5.58e-02 |
| ENSG00000140623 | 1.42e-05 | 5.47e-05 |
| ENSG00000140632 | 1.24e-03 | 3.20e-03 |
| ENSG00000140650 | 1.27e-07 | 7.06e-07 |
| ENSG00000140675 | 2.29e-09 | 1.70e-08 |
| ENSG00000140678 | 1.82e-07 | 9.81e-07 |
| ENSG00000140682 | 3.44e-03 | 7.97e-03 |
| ENSG00000140688 | 1.63e-04 | 5.08e-04 |
| ENSG00000140691 | 1.33e-03 | 3.43e-03 |
| ENSG00000140694 | 4.64e-08 | 2.77e-07 |
| ENSG00000140718 | 2.06e-02 | 3.83e-02 |
| ENSG00000140740 | 1.27e-08 | 8.32e-08 |
| ENSG00000140743 | 4.85e-16 | 1.25e-14 |
| ENSG00000140749 | 6.50e-02 | 1.04e-01 |
| ENSG00000140750 | 3.40e-08 | 2.08e-07 |
| ENSG00000140795 | 6.94e-02 | 1.10e-01 |
| ENSG00000140807 | 1.42e-12 | 1.86e-11 |
| ENSG00000140829 | 1.23e-01 | 1.80e-01 |
| ENSG00000140830 | 6.87e-07 | 3.34e-06 |
| ENSG00000140832 | 1.26e-11 | 1.38e-10 |
| ENSG00000140835 | 4.80e-10 | 4.00e-09 |
| ENSG00000140836 | 3.88e-01 | 4.71e-01 |
| ENSG00000140839 | 1.27e-01 | 1.85e-01 |
| ENSG00000140848 | 2.57e-06 | 1.13e-05 |
| ENSG00000140853 | 4.91e-06 | 2.05e-05 |
| ENSG00000140854 | 1.26e-06 | 5.84e-06 |
| ENSG00000140859 | 9.16e-13 | 1.25e-11 |
| ENSG00000140873 | 5.59e-05 | 1.91e-04 |
| ENSG00000140876 | 5.16e-10 | 4.28e-09 |
| ENSG00000140905 | 6.92e-07 | 3.37e-06 |
| ENSG00000140931 | 7.92e-03 | 1.66e-02 |
| ENSG00000140932 | 1.80e-04 | 5.58e-04 |
| ENSG00000140937 | 3.01e-18 | 1.26e-16 |
| ENSG00000140939 | 9.97e-16 | 2.38e-14 |
| ENSG00000140941 | 7.93e-02 | 1.23e-01 |
| ENSG00000140943 | 4.82e-01 | 5.64e-01 |
| ENSG00000140945 | 7.72e-07 | 3.73e-06 |
| ENSG00000140948 | 1.47e-01 | 2.08e-01 |
| ENSG00000140950 | 1.98e-02 | 3.71e-02 |
| ENSG00000140955 | 1.22e-03 | 3.16e-03 |
| ENSG00000140961 | 1.30e-06 | 6.01e-06 |
| ENSG00000140968 | 1.51e-01 | 2.13e-01 |
| ENSG00000140983 | 9.34e-04 | 2.49e-03 |
| ENSG00000140986 | 8.15e-03 | 1.71e-02 |
| ENSG00000140987 | 2.48e-01 | 3.25e-01 |
| ENSG00000140988 | 2.09e-11 | 2.20e-10 |
| ENSG00000140990 | 2.15e-02 | 3.98e-02 |
| ENSG00000140992 | 8.29e-01 | 8.70e-01 |
| ENSG00000140993 | 2.65e-05 | 9.70e-05 |
| ENSG00000140995 | 6.27e-08 | 3.66e-07 |
| ENSG00000141002 | 3.00e-01 | 3.80e-01 |
| ENSG00000141012 | 7.87e-01 | 8.36e-01 |
| ENSG00000141013 | 2.81e-13 | 4.21e-12 |
| ENSG00000141026 | 3.40e-11 | 3.45e-10 |
| ENSG00000141027 | 1.33e-07 | 7.36e-07 |
| ENSG00000141028 | 7.86e-01 | 8.35e-01 |
| ENSG00000141030 | 8.05e-01 | 8.50e-01 |
| ENSG00000141034 | 4.95e-01 | 5.76e-01 |
| ENSG00000141040 | 6.27e-08 | 3.66e-07 |
| ENSG00000141052 | 3.74e-12 | 4.54e-11 |
| ENSG00000141068 | 3.26e-01 | 4.07e-01 |
| ENSG00000141076 | 8.70e-24 | 1.92e-21 |
| ENSG00000141084 | 6.22e-06 | 2.54e-05 |
| ENSG00000141086 | 3.90e-03 | 8.91e-03 |
| ENSG00000141096 | 2.98e-12 | 3.69e-11 |
| ENSG00000141098 | 1.21e-02 | 2.41e-02 |
| ENSG00000141101 | 1.48e-18 | 6.65e-17 |
| ENSG00000141127 | 1.09e-02 | 2.20e-02 |
| ENSG00000141161 | 3.99e-02 | 6.82e-02 |
| ENSG00000141179 | 5.87e-04 | 1.64e-03 |
| ENSG00000141198 | 4.11e-01 | 4.94e-01 |
| ENSG00000141219 | 2.53e-03 | 6.07e-03 |
| ENSG00000141232 | 9.65e-01 | 9.74e-01 |
| ENSG00000141252 | 6.27e-02 | 1.01e-01 |
| ENSG00000141255 | 1.25e-06 | 5.81e-06 |
| ENSG00000141258 | 4.09e-01 | 4.92e-01 |
| ENSG00000141279 | 7.21e-05 | 2.42e-04 |
| ENSG00000141293 | 5.98e-05 | 2.04e-04 |
| ENSG00000141294 | 1.57e-10 | 1.42e-09 |
| ENSG00000141295 | 3.22e-01 | 4.04e-01 |
| ENSG00000141298 | 1.57e-01 | 2.20e-01 |
| ENSG00000141314 | 2.08e-05 | 7.74e-05 |
| ENSG00000141316 | 2.30e-04 | 6.97e-04 |
| ENSG00000141337 | 2.07e-01 | 2.78e-01 |
| ENSG00000141338 | 1.28e-25 | 5.57e-23 |
| ENSG00000141349 | 5.02e-13 | 7.17e-12 |
| ENSG00000141367 | 1.52e-02 | 2.94e-02 |
| ENSG00000141371 | 9.74e-01 | 9.81e-01 |
| ENSG00000141376 | 4.19e-01 | 5.02e-01 |
| ENSG00000141378 | 2.16e-14 | 4.00e-13 |
| ENSG00000141380 | 8.86e-02 | 1.36e-01 |
| ENSG00000141384 | 6.56e-06 | 2.68e-05 |
| ENSG00000141385 | 7.11e-17 | 2.17e-15 |
| ENSG00000141391 | 1.24e-19 | 7.33e-18 |
| ENSG00000141401 | 2.26e-11 | 2.37e-10 |
| ENSG00000141404 | 4.94e-11 | 4.88e-10 |
| ENSG00000141424 | 3.05e-14 | 5.44e-13 |
| ENSG00000141425 | 1.70e-01 | 2.36e-01 |
| ENSG00000141428 | 1.00e-01 | 1.51e-01 |
| ENSG00000141429 | 4.44e-07 | 2.23e-06 |
| ENSG00000141431 | 3.32e-17 | 1.10e-15 |
| ENSG00000141433 | 2.77e-04 | 8.24e-04 |
| ENSG00000141434 | 1.03e-15 | 2.45e-14 |
| ENSG00000141437 | 3.99e-01 | 4.82e-01 |
| ENSG00000141441 | 5.83e-10 | 4.79e-09 |
| ENSG00000141446 | 9.29e-01 | 9.47e-01 |
| ENSG00000141447 | 2.56e-16 | 7.00e-15 |
| ENSG00000141448 | 7.05e-01 | 7.66e-01 |
| ENSG00000141449 | 3.29e-01 | 4.10e-01 |
| ENSG00000141452 | 2.29e-06 | 1.01e-05 |
| ENSG00000141456 | 3.13e-03 | 7.33e-03 |
| ENSG00000141458 | 2.73e-04 | 8.15e-04 |
| ENSG00000141469 | 1.68e-03 | 4.20e-03 |
| ENSG00000141480 | 6.97e-02 | 1.10e-01 |
| ENSG00000141485 | 2.63e-01 | 3.41e-01 |
| ENSG00000141497 | 9.20e-08 | 5.22e-07 |
| ENSG00000141499 | 9.45e-04 | 2.51e-03 |
| ENSG00000141503 | 4.92e-06 | 2.05e-05 |
| ENSG00000141504 | 1.92e-04 | 5.93e-04 |
| ENSG00000141505 | 3.20e-12 | 3.94e-11 |
| ENSG00000141506 | 1.41e-06 | 6.47e-06 |
| ENSG00000141510 | 8.32e-03 | 1.74e-02 |
| ENSG00000141519 | 2.44e-10 | 2.14e-09 |
| ENSG00000141522 | 2.22e-01 | 2.95e-01 |
| ENSG00000141524 | 6.44e-04 | 1.78e-03 |
| ENSG00000141526 | 2.05e-01 | 2.76e-01 |
| ENSG00000141527 | 3.78e-18 | 1.54e-16 |
| ENSG00000141540 | 3.08e-05 | 1.11e-04 |
| ENSG00000141542 | 2.74e-02 | 4.92e-02 |
| ENSG00000141543 | 5.85e-08 | 3.44e-07 |
| ENSG00000141551 | 4.11e-01 | 4.94e-01 |
| ENSG00000141552 | 2.57e-04 | 7.72e-04 |
| ENSG00000141556 | 5.88e-13 | 8.27e-12 |
| ENSG00000141560 | 5.14e-01 | 5.94e-01 |
| ENSG00000141562 | 7.95e-08 | 4.56e-07 |
| ENSG00000141564 | 1.44e-02 | 2.80e-02 |
| ENSG00000141568 | 4.54e-13 | 6.55e-12 |
| ENSG00000141569 | 3.31e-14 | 5.86e-13 |
| ENSG00000141570 | 1.59e-23 | 3.17e-21 |
| ENSG00000141574 | 3.09e-20 | 2.14e-18 |
| ENSG00000141576 | 2.81e-01 | 3.60e-01 |
| ENSG00000141577 | 4.28e-07 | 2.15e-06 |
| ENSG00000141579 | 1.04e-05 | 4.11e-05 |
| ENSG00000141580 | 2.63e-10 | 2.30e-09 |
| ENSG00000141582 | 9.27e-25 | 2.68e-22 |
| ENSG00000141622 | 7.90e-06 | 3.19e-05 |
| ENSG00000141627 | 4.41e-01 | 5.24e-01 |
| ENSG00000141639 | 6.09e-15 | 1.24e-13 |
| ENSG00000141642 | 5.96e-10 | 4.89e-09 |
| ENSG00000141644 | 2.04e-06 | 9.10e-06 |
| ENSG00000141646 | 1.02e-06 | 4.79e-06 |
| ENSG00000141655 | 6.18e-11 | 6.01e-10 |
| ENSG00000141664 | 4.69e-04 | 1.33e-03 |
| ENSG00000141665 | 9.60e-01 | 9.71e-01 |
| ENSG00000141668 | 1.43e-20 | 1.11e-18 |
| ENSG00000141682 | 1.66e-21 | 1.75e-19 |
| ENSG00000141696 | 5.12e-11 | 5.05e-10 |
| ENSG00000141698 | 2.57e-09 | 1.89e-08 |
| ENSG00000141699 | 1.52e-01 | 2.15e-01 |
| ENSG00000141736 | 2.77e-02 | 4.97e-02 |
| ENSG00000141738 | 2.45e-12 | 3.08e-11 |
| ENSG00000141741 | 2.50e-01 | 3.27e-01 |
| ENSG00000141744 | 6.44e-01 | 7.13e-01 |
| ENSG00000141748 | 3.39e-03 | 7.86e-03 |
| ENSG00000141750 | 3.01e-09 | 2.19e-08 |
| ENSG00000141753 | 3.22e-01 | 4.03e-01 |
| ENSG00000141756 | 1.29e-16 | 3.74e-15 |
| ENSG00000141759 | 2.40e-01 | 3.16e-01 |
| ENSG00000141837 | 6.74e-12 | 7.77e-11 |
| ENSG00000141854 | 4.06e-01 | 4.89e-01 |
| ENSG00000141858 | 9.27e-09 | 6.21e-08 |
| ENSG00000141867 | 3.37e-09 | 2.43e-08 |
| ENSG00000141873 | 7.47e-05 | 2.49e-04 |
| ENSG00000141905 | 2.23e-02 | 4.12e-02 |
| ENSG00000141933 | 4.15e-05 | 1.46e-04 |
| ENSG00000141934 | 1.30e-07 | 7.22e-07 |
| ENSG00000141956 | 2.23e-06 | 9.84e-06 |
| ENSG00000141959 | 4.77e-01 | 5.59e-01 |
| ENSG00000141965 | 7.30e-01 | 7.87e-01 |
| ENSG00000141968 | 8.49e-09 | 5.73e-08 |
| ENSG00000141971 | 9.11e-01 | 9.34e-01 |
| ENSG00000141977 | 1.97e-01 | 2.67e-01 |
| ENSG00000141979 | 3.16e-02 | 5.57e-02 |
| ENSG00000141985 | 2.13e-09 | 1.59e-08 |
| ENSG00000141994 | 5.03e-11 | 4.96e-10 |
| ENSG00000142002 | 5.50e-03 | 1.20e-02 |
| ENSG00000142039 | 3.39e-01 | 4.21e-01 |
| ENSG00000142046 | 8.25e-01 | 8.67e-01 |
| ENSG00000142065 | 5.01e-01 | 5.81e-01 |
| ENSG00000142082 | 7.39e-03 | 1.56e-02 |
| ENSG00000142089 | 2.17e-19 | 1.21e-17 |
| ENSG00000142102 | 1.10e-13 | 1.78e-12 |
| ENSG00000142149 | 1.39e-03 | 3.57e-03 |
| ENSG00000142156 | 1.20e-01 | 1.76e-01 |
| ENSG00000142166 | 5.26e-03 | 1.16e-02 |
| ENSG00000142168 | 4.31e-01 | 5.14e-01 |
| ENSG00000142173 | 4.21e-01 | 5.04e-01 |
| ENSG00000142178 | 3.96e-01 | 4.79e-01 |
| ENSG00000142182 | 6.68e-03 | 1.43e-02 |
| ENSG00000142185 | 3.96e-11 | 3.99e-10 |
| ENSG00000142186 | 3.44e-01 | 4.26e-01 |
| ENSG00000142188 | 1.00e-03 | 2.65e-03 |
| ENSG00000142192 | 1.93e-01 | 2.62e-01 |
| ENSG00000142197 | 3.73e-05 | 1.32e-04 |
| ENSG00000142207 | 1.32e-20 | 1.04e-18 |
| ENSG00000142208 | 3.20e-01 | 4.01e-01 |
| ENSG00000142224 | 9.12e-01 | 9.35e-01 |
| ENSG00000142227 | 2.68e-05 | 9.81e-05 |
| ENSG00000142230 | 4.10e-06 | 1.74e-05 |
| ENSG00000142233 | 3.24e-02 | 5.69e-02 |
| ENSG00000142235 | 1.13e-17 | 4.10e-16 |
| ENSG00000142252 | 1.21e-02 | 2.41e-02 |
| ENSG00000142273 | 6.90e-01 | 7.53e-01 |
| ENSG00000142279 | 8.67e-01 | 9.00e-01 |
| ENSG00000142303 | 6.76e-01 | 7.41e-01 |
| ENSG00000142319 | 7.55e-05 | 2.52e-04 |
| ENSG00000142327 | 2.30e-01 | 3.05e-01 |
| ENSG00000142330 | 3.90e-16 | 1.02e-14 |
| ENSG00000142347 | 1.30e-01 | 1.88e-01 |
| ENSG00000142396 | 6.25e-02 | 1.01e-01 |
| ENSG00000142405 | 8.13e-02 | 1.26e-01 |
| ENSG00000142408 | 7.03e-19 | 3.45e-17 |
| ENSG00000142409 | 3.01e-01 | 3.82e-01 |
| ENSG00000142444 | 2.62e-06 | 1.14e-05 |
| ENSG00000142449 | 6.20e-02 | 9.98e-02 |
| ENSG00000142453 | 1.54e-09 | 1.18e-08 |
| ENSG00000142459 | 6.32e-03 | 1.36e-02 |
| ENSG00000142484 | 8.55e-01 | 8.91e-01 |
| ENSG00000142494 | 3.80e-10 | 3.22e-09 |
| ENSG00000142507 | 6.45e-01 | 7.14e-01 |
| ENSG00000142512 | 1.06e-03 | 2.79e-03 |
| ENSG00000142513 | 1.88e-04 | 5.81e-04 |
| ENSG00000142515 | 6.67e-07 | 3.25e-06 |
| ENSG00000142528 | 4.76e-18 | 1.89e-16 |
| ENSG00000142530 | 2.76e-14 | 4.98e-13 |
| ENSG00000142534 | 4.75e-04 | 1.35e-03 |
| ENSG00000142541 | 1.09e-01 | 1.62e-01 |
| ENSG00000142544 | 5.88e-12 | 6.86e-11 |
| ENSG00000142546 | 8.73e-02 | 1.34e-01 |
| ENSG00000142549 | 9.19e-02 | 1.40e-01 |
| ENSG00000142552 | 7.48e-06 | 3.03e-05 |
| ENSG00000142556 | 3.46e-01 | 4.29e-01 |
| ENSG00000142583 | 6.06e-10 | 4.96e-09 |
| ENSG00000142599 | 2.00e-06 | 8.91e-06 |
| ENSG00000142606 | 8.94e-01 | 9.21e-01 |
| ENSG00000142609 | 9.48e-10 | 7.51e-09 |
| ENSG00000142611 | 2.55e-04 | 7.66e-04 |
| ENSG00000142615 | 1.03e-01 | 1.54e-01 |
| ENSG00000142619 | 6.50e-05 | 2.20e-04 |
| ENSG00000142621 | 6.72e-05 | 2.27e-04 |
| ENSG00000142623 | 2.57e-05 | 9.42e-05 |
| ENSG00000142627 | 1.71e-06 | 7.72e-06 |
| ENSG00000142632 | 1.00e-12 | 1.35e-11 |
| ENSG00000142634 | 1.58e-01 | 2.21e-01 |
| ENSG00000142655 | 9.16e-01 | 9.37e-01 |
| ENSG00000142657 | 6.93e-07 | 3.37e-06 |
| ENSG00000142661 | 1.83e-10 | 1.64e-09 |
| ENSG00000142669 | 4.35e-03 | 9.79e-03 |
| ENSG00000142675 | 4.30e-02 | 7.28e-02 |
| ENSG00000142676 | 3.55e-03 | 8.19e-03 |
| ENSG00000142677 | 6.90e-01 | 7.54e-01 |
| ENSG00000142684 | 6.51e-10 | 5.30e-09 |
| ENSG00000142686 | 5.92e-04 | 1.65e-03 |
| ENSG00000142687 | 1.65e-01 | 2.30e-01 |
| ENSG00000142694 | 9.36e-04 | 2.49e-03 |
| ENSG00000142698 | 2.34e-06 | 1.03e-05 |
| ENSG00000142700 | 2.56e-08 | 1.59e-07 |
| ENSG00000142731 | 8.23e-09 | 5.56e-08 |
| ENSG00000142733 | 5.18e-01 | 5.98e-01 |
| ENSG00000142748 | 2.63e-10 | 2.30e-09 |
| ENSG00000142751 | 2.33e-01 | 3.08e-01 |
| ENSG00000142765 | 2.63e-03 | 6.29e-03 |
| ENSG00000142784 | 7.92e-08 | 4.54e-07 |
| ENSG00000142789 | 9.11e-06 | 3.63e-05 |
| ENSG00000142794 | 2.79e-01 | 3.59e-01 |
| ENSG00000142798 | 4.42e-01 | 5.25e-01 |
| ENSG00000142856 | 1.41e-05 | 5.45e-05 |
| ENSG00000142864 | 1.66e-04 | 5.18e-04 |
| ENSG00000142867 | 2.70e-10 | 2.36e-09 |
| ENSG00000142871 | 3.45e-01 | 4.27e-01 |
| ENSG00000142875 | 1.05e-17 | 3.84e-16 |
| ENSG00000142892 | 4.78e-02 | 7.97e-02 |
| ENSG00000142910 | 1.83e-03 | 4.54e-03 |
| ENSG00000142920 | 7.01e-06 | 2.85e-05 |
| ENSG00000142937 | 5.07e-03 | 1.12e-02 |
| ENSG00000142945 | 9.58e-17 | 2.86e-15 |
| ENSG00000142949 | 1.03e-09 | 8.13e-09 |
| ENSG00000142959 | 1.27e-36 | 1.33e-32 |
| ENSG00000142961 | 1.44e-03 | 3.67e-03 |
| ENSG00000142973 | 7.63e-11 | 7.30e-10 |
| ENSG00000143001 | 4.64e-11 | 4.62e-10 |
| ENSG00000143013 | 1.02e-03 | 2.68e-03 |
| ENSG00000143028 | 3.53e-08 | 2.15e-07 |
| ENSG00000143033 | 2.70e-01 | 3.49e-01 |
| ENSG00000143036 | 9.85e-02 | 1.48e-01 |
| ENSG00000143061 | 1.00e-12 | 1.35e-11 |
| ENSG00000143067 | 3.39e-15 | 7.29e-14 |
| ENSG00000143079 | 1.65e-02 | 3.15e-02 |
| ENSG00000143093 | 2.93e-01 | 3.74e-01 |
| ENSG00000143105 | 5.44e-05 | 1.87e-04 |
| ENSG00000143106 | 4.50e-06 | 1.89e-05 |
| ENSG00000143107 | 3.33e-01 | 4.14e-01 |
| ENSG00000143110 | 6.04e-04 | 1.68e-03 |
| ENSG00000143119 | 9.54e-05 | 3.12e-04 |
| ENSG00000143125 | 2.18e-02 | 4.03e-02 |
| ENSG00000143126 | 2.19e-09 | 1.63e-08 |
| ENSG00000143127 | 7.50e-01 | 8.04e-01 |
| ENSG00000143147 | 4.52e-01 | 5.35e-01 |
| ENSG00000143149 | 5.54e-07 | 2.74e-06 |
| ENSG00000143153 | 9.10e-03 | 1.88e-02 |
| ENSG00000143155 | 2.30e-02 | 4.22e-02 |
| ENSG00000143156 | 1.57e-05 | 5.97e-05 |
| ENSG00000143157 | 5.10e-11 | 5.03e-10 |
| ENSG00000143158 | 1.23e-05 | 4.79e-05 |
| ENSG00000143162 | 5.27e-03 | 1.16e-02 |
| ENSG00000143164 | 2.42e-06 | 1.06e-05 |
| ENSG00000143167 | 1.49e-15 | 3.41e-14 |
| ENSG00000143171 | 8.51e-25 | 2.53e-22 |
| ENSG00000143178 | 4.97e-03 | 1.10e-02 |
| ENSG00000143179 | 6.66e-04 | 1.83e-03 |
| ENSG00000143183 | 1.68e-08 | 1.08e-07 |
| ENSG00000143184 | 3.20e-07 | 1.65e-06 |
| ENSG00000143185 | 8.81e-13 | 1.20e-11 |
| ENSG00000143190 | 1.00e-04 | 3.27e-04 |
| ENSG00000143194 | 7.28e-02 | 1.15e-01 |
| ENSG00000143195 | 3.79e-02 | 6.52e-02 |
| ENSG00000143196 | 1.84e-21 | 1.90e-19 |
| ENSG00000143198 | 1.07e-14 | 2.09e-13 |
| ENSG00000143199 | 5.34e-01 | 6.13e-01 |
| ENSG00000143207 | 2.45e-01 | 3.21e-01 |
| ENSG00000143217 | 3.86e-13 | 5.63e-12 |
| ENSG00000143222 | 7.06e-02 | 1.12e-01 |
| ENSG00000143224 | 3.59e-07 | 1.84e-06 |
| ENSG00000143226 | 1.71e-03 | 4.26e-03 |
| ENSG00000143228 | 2.06e-16 | 5.75e-15 |
| ENSG00000143248 | 1.33e-02 | 2.61e-02 |
| ENSG00000143252 | 1.45e-02 | 2.82e-02 |
| ENSG00000143256 | 2.92e-14 | 5.23e-13 |
| ENSG00000143257 | 1.91e-03 | 4.72e-03 |
| ENSG00000143258 | 4.35e-05 | 1.52e-04 |
| ENSG00000143278 | 9.94e-01 | 9.95e-01 |
| ENSG00000143294 | 1.33e-01 | 1.91e-01 |
| ENSG00000143297 | 5.80e-09 | 4.03e-08 |
| ENSG00000143303 | 8.22e-01 | 8.65e-01 |
| ENSG00000143314 | 1.17e-02 | 2.34e-02 |
| ENSG00000143315 | 1.26e-07 | 6.99e-07 |
| ENSG00000143318 | 2.90e-05 | 1.05e-04 |
| ENSG00000143319 | 1.78e-11 | 1.89e-10 |
| ENSG00000143320 | 3.54e-04 | 1.03e-03 |
| ENSG00000143321 | 7.87e-08 | 4.51e-07 |
| ENSG00000143322 | 1.81e-08 | 1.16e-07 |
| ENSG00000143324 | 6.22e-02 | 1.00e-01 |
| ENSG00000143333 | 5.29e-19 | 2.69e-17 |
| ENSG00000143337 | 7.78e-04 | 2.11e-03 |
| ENSG00000143340 | 6.04e-14 | 1.02e-12 |
| ENSG00000143341 | 5.20e-01 | 6.00e-01 |
| ENSG00000143344 | 7.91e-14 | 1.30e-12 |
| ENSG00000143353 | 1.35e-03 | 3.47e-03 |
| ENSG00000143355 | 3.08e-02 | 5.44e-02 |
| ENSG00000143363 | 9.13e-02 | 1.39e-01 |
| ENSG00000143365 | 1.78e-05 | 6.71e-05 |
| ENSG00000143367 | 5.17e-03 | 1.14e-02 |
| ENSG00000143368 | 1.99e-06 | 8.90e-06 |
| ENSG00000143369 | 5.66e-07 | 2.79e-06 |
| ENSG00000143373 | 1.03e-01 | 1.54e-01 |
| ENSG00000143374 | 6.10e-10 | 4.99e-09 |
| ENSG00000143375 | 8.51e-10 | 6.80e-09 |
| ENSG00000143376 | 5.92e-02 | 9.59e-02 |
| ENSG00000143379 | 9.25e-10 | 7.35e-09 |
| ENSG00000143382 | 5.62e-11 | 5.50e-10 |
| ENSG00000143384 | 3.01e-03 | 7.09e-03 |
| ENSG00000143387 | 3.47e-03 | 8.03e-03 |
| ENSG00000143390 | 1.87e-05 | 7.04e-05 |
| ENSG00000143393 | 1.77e-01 | 2.44e-01 |
| ENSG00000143398 | 6.30e-04 | 1.74e-03 |
| ENSG00000143401 | 5.81e-03 | 1.26e-02 |
| ENSG00000143409 | 1.41e-11 | 1.53e-10 |
| ENSG00000143412 | 1.09e-02 | 2.20e-02 |
| ENSG00000143416 | 6.67e-16 | 1.66e-14 |
| ENSG00000143418 | 4.75e-04 | 1.35e-03 |
| ENSG00000143420 | 7.22e-04 | 1.97e-03 |
| ENSG00000143429 | 2.99e-04 | 8.84e-04 |
| ENSG00000143434 | 3.70e-04 | 1.07e-03 |
| ENSG00000143436 | 1.25e-08 | 8.18e-08 |
| ENSG00000143437 | 3.77e-05 | 1.34e-04 |
| ENSG00000143442 | 7.83e-01 | 8.32e-01 |
| ENSG00000143443 | 7.93e-05 | 2.63e-04 |
| ENSG00000143450 | 5.21e-01 | 6.00e-01 |
| ENSG00000143452 | 5.41e-01 | 6.19e-01 |
| ENSG00000143457 | 6.35e-06 | 2.60e-05 |
| ENSG00000143458 | 3.87e-04 | 1.12e-03 |
| ENSG00000143469 | 6.19e-01 | 6.90e-01 |
| ENSG00000143473 | 3.29e-07 | 1.69e-06 |
| ENSG00000143476 | 1.72e-14 | 3.24e-13 |
| ENSG00000143479 | 1.99e-08 | 1.26e-07 |
| ENSG00000143486 | 7.62e-02 | 1.19e-01 |
| ENSG00000143493 | 1.75e-19 | 9.99e-18 |
| ENSG00000143494 | 2.23e-10 | 1.97e-09 |
| ENSG00000143498 | 1.19e-16 | 3.47e-15 |
| ENSG00000143499 | 2.84e-17 | 9.47e-16 |
| ENSG00000143502 | 1.25e-03 | 3.22e-03 |
| ENSG00000143507 | 8.76e-14 | 1.44e-12 |
| ENSG00000143512 | 3.52e-01 | 4.35e-01 |
| ENSG00000143514 | 2.90e-02 | 5.16e-02 |
| ENSG00000143515 | 5.42e-08 | 3.21e-07 |
| ENSG00000143520 | 2.88e-02 | 5.13e-02 |
| ENSG00000143537 | 8.91e-06 | 3.56e-05 |
| ENSG00000143543 | 5.50e-02 | 9.00e-02 |
| ENSG00000143545 | 8.86e-01 | 9.15e-01 |
| ENSG00000143546 | 7.16e-02 | 1.13e-01 |
| ENSG00000143549 | 4.37e-01 | 5.20e-01 |
| ENSG00000143552 | 2.23e-01 | 2.96e-01 |
| ENSG00000143553 | 7.65e-01 | 8.17e-01 |
| ENSG00000143554 | 8.02e-01 | 8.48e-01 |
| ENSG00000143556 | 7.27e-05 | 2.44e-04 |
| ENSG00000143569 | 1.44e-18 | 6.52e-17 |
| ENSG00000143570 | 6.10e-07 | 2.99e-06 |
| ENSG00000143575 | 2.13e-01 | 2.85e-01 |
| ENSG00000143578 | 2.11e-02 | 3.92e-02 |
| ENSG00000143590 | 2.14e-14 | 3.96e-13 |
| ENSG00000143595 | 3.05e-01 | 3.86e-01 |
| ENSG00000143603 | 2.72e-13 | 4.08e-12 |
| ENSG00000143612 | 4.11e-03 | 9.34e-03 |
| ENSG00000143614 | 3.16e-03 | 7.38e-03 |
| ENSG00000143621 | 4.33e-11 | 4.32e-10 |
| ENSG00000143622 | 5.16e-01 | 5.95e-01 |
| ENSG00000143624 | 2.41e-07 | 1.27e-06 |
| ENSG00000143627 | 4.47e-01 | 5.30e-01 |
| ENSG00000143630 | 6.53e-03 | 1.40e-02 |
| ENSG00000143631 | 2.91e-01 | 3.71e-01 |
| ENSG00000143632 | 6.43e-01 | 7.12e-01 |
| ENSG00000143633 | 5.82e-04 | 1.62e-03 |
| ENSG00000143641 | 1.85e-07 | 9.94e-07 |
| ENSG00000143643 | 1.07e-01 | 1.59e-01 |
| ENSG00000143653 | 2.27e-01 | 3.00e-01 |
| ENSG00000143669 | 1.47e-06 | 6.73e-06 |
| ENSG00000143674 | 8.34e-05 | 2.76e-04 |
| ENSG00000143702 | 3.64e-02 | 6.31e-02 |
| ENSG00000143727 | 1.25e-14 | 2.41e-13 |
| ENSG00000143740 | 1.39e-04 | 4.41e-04 |
| ENSG00000143742 | 4.58e-01 | 5.40e-01 |
| ENSG00000143748 | 2.12e-03 | 5.18e-03 |
| ENSG00000143751 | 2.89e-04 | 8.57e-04 |
| ENSG00000143753 | 3.50e-03 | 8.10e-03 |
| ENSG00000143756 | 4.84e-01 | 5.66e-01 |
| ENSG00000143761 | 9.03e-03 | 1.87e-02 |
| ENSG00000143768 | 3.15e-01 | 3.96e-01 |
| ENSG00000143771 | 1.90e-03 | 4.68e-03 |
| ENSG00000143772 | 4.79e-05 | 1.66e-04 |
| ENSG00000143774 | 1.11e-05 | 4.35e-05 |
| ENSG00000143776 | 4.05e-08 | 2.44e-07 |
| ENSG00000143786 | 1.59e-13 | 2.48e-12 |
| ENSG00000143793 | 7.20e-09 | 4.92e-08 |
| ENSG00000143797 | 3.92e-02 | 6.71e-02 |
| ENSG00000143799 | 5.16e-07 | 2.56e-06 |
| ENSG00000143801 | 8.87e-01 | 9.16e-01 |
| ENSG00000143811 | 1.90e-11 | 2.01e-10 |
| ENSG00000143815 | 6.84e-04 | 1.88e-03 |
| ENSG00000143816 | 8.33e-09 | 5.63e-08 |
| ENSG00000143819 | 1.24e-11 | 1.35e-10 |
| ENSG00000143839 | 6.64e-02 | 1.06e-01 |
| ENSG00000143842 | 5.86e-01 | 6.61e-01 |
| ENSG00000143845 | 2.84e-01 | 3.63e-01 |
| ENSG00000143847 | 1.81e-05 | 6.81e-05 |
| ENSG00000143850 | 4.99e-05 | 1.73e-04 |
| ENSG00000143851 | 1.04e-02 | 2.12e-02 |
| ENSG00000143858 | 2.76e-07 | 1.44e-06 |
| ENSG00000143862 | 2.20e-10 | 1.95e-09 |
| ENSG00000143867 | 2.07e-16 | 5.76e-15 |
| ENSG00000143869 | 1.75e-07 | 9.47e-07 |
| ENSG00000143870 | 5.02e-07 | 2.50e-06 |
| ENSG00000143878 | 6.34e-01 | 7.04e-01 |
| ENSG00000143882 | 4.58e-20 | 3.03e-18 |
| ENSG00000143889 | 1.24e-03 | 3.21e-03 |
| ENSG00000143891 | 1.53e-15 | 3.51e-14 |
| ENSG00000143919 | 4.24e-12 | 5.09e-11 |
| ENSG00000143921 | 6.50e-01 | 7.18e-01 |
| ENSG00000143924 | 3.65e-03 | 8.40e-03 |
| ENSG00000143933 | 2.00e-06 | 8.94e-06 |
| ENSG00000143942 | 8.48e-01 | 8.85e-01 |
| ENSG00000143947 | 2.53e-03 | 6.06e-03 |
| ENSG00000143951 | 1.98e-02 | 3.71e-02 |
| ENSG00000143952 | 2.83e-02 | 5.05e-02 |
| ENSG00000143954 | 2.74e-04 | 8.16e-04 |
| ENSG00000143970 | 1.27e-01 | 1.84e-01 |
| ENSG00000143971 | 4.89e-02 | 8.14e-02 |
| ENSG00000143977 | 3.87e-08 | 2.34e-07 |
| ENSG00000143994 | 5.09e-06 | 2.12e-05 |
| ENSG00000143995 | 3.31e-10 | 2.84e-09 |
| ENSG00000144021 | 1.16e-09 | 9.09e-09 |
| ENSG00000144026 | 1.40e-11 | 1.51e-10 |
| ENSG00000144028 | 9.12e-09 | 6.12e-08 |
| ENSG00000144029 | 8.30e-03 | 1.73e-02 |
| ENSG00000144031 | 1.63e-07 | 8.88e-07 |
| ENSG00000144034 | 9.86e-01 | 9.90e-01 |
| ENSG00000144035 | 1.09e-06 | 5.10e-06 |
| ENSG00000144036 | 8.39e-09 | 5.66e-08 |
| ENSG00000144040 | 2.42e-06 | 1.06e-05 |
| ENSG00000144043 | 2.19e-01 | 2.92e-01 |
| ENSG00000144045 | 3.37e-06 | 1.44e-05 |
| ENSG00000144048 | 2.13e-01 | 2.85e-01 |
| ENSG00000144057 | 7.33e-02 | 1.15e-01 |
| ENSG00000144061 | 1.09e-02 | 2.20e-02 |
| ENSG00000144063 | 1.16e-22 | 1.69e-20 |
| ENSG00000144115 | 7.71e-01 | 8.22e-01 |
| ENSG00000144118 | 1.06e-07 | 5.97e-07 |
| ENSG00000144119 | 7.93e-01 | 8.40e-01 |
| ENSG00000144120 | 6.59e-02 | 1.05e-01 |
| ENSG00000144130 | 3.23e-12 | 3.97e-11 |
| ENSG00000144134 | 1.68e-01 | 2.33e-01 |
| ENSG00000144136 | 3.86e-07 | 1.96e-06 |
| ENSG00000144152 | 9.03e-06 | 3.60e-05 |
| ENSG00000144158 | 8.93e-07 | 4.27e-06 |
| ENSG00000144161 | 2.35e-14 | 4.31e-13 |
| ENSG00000144182 | 4.85e-01 | 5.67e-01 |
| ENSG00000144191 | 3.91e-15 | 8.33e-14 |
| ENSG00000144199 | 1.69e-01 | 2.35e-01 |
| ENSG00000144214 | 1.92e-04 | 5.93e-04 |
| ENSG00000144218 | 1.98e-19 | 1.12e-17 |
| ENSG00000144224 | 3.46e-03 | 8.01e-03 |
| ENSG00000144227 | 6.94e-03 | 1.48e-02 |
| ENSG00000144228 | 6.33e-03 | 1.36e-02 |
| ENSG00000144229 | 8.93e-03 | 1.85e-02 |
| ENSG00000144230 | 7.83e-06 | 3.16e-05 |
| ENSG00000144231 | 3.17e-21 | 3.01e-19 |
| ENSG00000144233 | 1.85e-11 | 1.96e-10 |
| ENSG00000144278 | 8.20e-10 | 6.57e-09 |
| ENSG00000144283 | 2.65e-16 | 7.23e-15 |
| ENSG00000144285 | 3.79e-02 | 6.53e-02 |
| ENSG00000144290 | 3.13e-20 | 2.17e-18 |
| ENSG00000144306 | 1.07e-02 | 2.16e-02 |
| ENSG00000144320 | 2.66e-02 | 4.80e-02 |
| ENSG00000144331 | 4.53e-10 | 3.79e-09 |
| ENSG00000144339 | 1.42e-23 | 2.90e-21 |
| ENSG00000144354 | 2.73e-17 | 9.15e-16 |
| ENSG00000144355 | 9.97e-03 | 2.03e-02 |
| ENSG00000144357 | 2.63e-01 | 3.41e-01 |
| ENSG00000144362 | 6.52e-01 | 7.20e-01 |
| ENSG00000144366 | 9.74e-06 | 3.86e-05 |
| ENSG00000144369 | 7.22e-05 | 2.42e-04 |
| ENSG00000144381 | 1.59e-15 | 3.64e-14 |
| ENSG00000144395 | 1.61e-14 | 3.04e-13 |
| ENSG00000144401 | 7.90e-03 | 1.66e-02 |
| ENSG00000144406 | 1.84e-14 | 3.45e-13 |
| ENSG00000144407 | 1.62e-02 | 3.11e-02 |
| ENSG00000144410 | 1.84e-01 | 2.52e-01 |
| ENSG00000144426 | 7.46e-14 | 1.23e-12 |
| ENSG00000144445 | 1.01e-04 | 3.28e-04 |
| ENSG00000144451 | 5.59e-06 | 2.31e-05 |
| ENSG00000144452 | 1.79e-04 | 5.57e-04 |
| ENSG00000144455 | 8.46e-05 | 2.79e-04 |
| ENSG00000144460 | 2.19e-01 | 2.92e-01 |
| ENSG00000144468 | 2.65e-02 | 4.79e-02 |
| ENSG00000144476 | 7.56e-02 | 1.18e-01 |
| ENSG00000144481 | 1.51e-01 | 2.14e-01 |
| ENSG00000144485 | 1.19e-12 | 1.59e-11 |
| ENSG00000144488 | 2.46e-01 | 3.23e-01 |
| ENSG00000144504 | 1.54e-11 | 1.65e-10 |
| ENSG00000144524 | 7.44e-16 | 1.83e-14 |
| ENSG00000144535 | 3.87e-09 | 2.77e-08 |
| ENSG00000144550 | 2.42e-07 | 1.27e-06 |
| ENSG00000144554 | 3.56e-13 | 5.21e-12 |
| ENSG00000144559 | 2.31e-18 | 9.90e-17 |
| ENSG00000144560 | 1.24e-09 | 9.67e-09 |
| ENSG00000144566 | 4.76e-01 | 5.58e-01 |
| ENSG00000144567 | 2.64e-07 | 1.38e-06 |
| ENSG00000144579 | 4.82e-07 | 2.41e-06 |
| ENSG00000144580 | 2.01e-17 | 7.00e-16 |
| ENSG00000144583 | 1.08e-05 | 4.26e-05 |
| ENSG00000144589 | 2.11e-01 | 2.83e-01 |
| ENSG00000144591 | 4.35e-03 | 9.80e-03 |
| ENSG00000144596 | 1.25e-02 | 2.49e-02 |
| ENSG00000144597 | 1.72e-04 | 5.36e-04 |
| ENSG00000144619 | 3.00e-20 | 2.09e-18 |
| ENSG00000144635 | 5.08e-05 | 1.75e-04 |
| ENSG00000144642 | 5.03e-10 | 4.18e-09 |
| ENSG00000144644 | 1.91e-03 | 4.73e-03 |
| ENSG00000144645 | 7.66e-12 | 8.73e-11 |
| ENSG00000144647 | 5.36e-03 | 1.18e-02 |
| ENSG00000144648 | 5.69e-20 | 3.63e-18 |
| ENSG00000144649 | 6.02e-08 | 3.53e-07 |
| ENSG00000144655 | 2.71e-02 | 4.88e-02 |
| ENSG00000144659 | 1.74e-01 | 2.41e-01 |
| ENSG00000144668 | 2.78e-01 | 3.57e-01 |
| ENSG00000144671 | 2.60e-01 | 3.37e-01 |
| ENSG00000144674 | 4.27e-02 | 7.23e-02 |
| ENSG00000144677 | 8.97e-05 | 2.95e-04 |
| ENSG00000144681 | 7.39e-03 | 1.56e-02 |
| ENSG00000144711 | 1.68e-02 | 3.20e-02 |
| ENSG00000144712 | 1.28e-15 | 3.00e-14 |
| ENSG00000144713 | 1.39e-05 | 5.35e-05 |
| ENSG00000144724 | 1.60e-04 | 5.00e-04 |
| ENSG00000144730 | 5.09e-03 | 1.13e-02 |
| ENSG00000144736 | 8.53e-07 | 4.09e-06 |
| ENSG00000144741 | 3.00e-07 | 1.56e-06 |
| ENSG00000144744 | 6.53e-02 | 1.04e-01 |
| ENSG00000144746 | 1.34e-07 | 7.40e-07 |
| ENSG00000144747 | 5.39e-02 | 8.84e-02 |
| ENSG00000144749 | 1.51e-01 | 2.14e-01 |
| ENSG00000144771 | 1.17e-02 | 2.34e-02 |
| ENSG00000144785 | 2.04e-03 | 4.99e-03 |
| ENSG00000144791 | 1.13e-02 | 2.28e-02 |
| ENSG00000144792 | 8.84e-05 | 2.91e-04 |
| ENSG00000144802 | 4.27e-01 | 5.10e-01 |
| ENSG00000144810 | 8.14e-12 | 9.25e-11 |
| ENSG00000144815 | 8.39e-10 | 6.71e-09 |
| ENSG00000144820 | 4.04e-09 | 2.88e-08 |
| ENSG00000144821 | 1.02e-14 | 2.00e-13 |
| ENSG00000144824 | 1.50e-05 | 5.73e-05 |
| ENSG00000144827 | 2.80e-03 | 6.64e-03 |
| ENSG00000144834 | 9.04e-14 | 1.48e-12 |
| ENSG00000144837 | 1.52e-06 | 6.93e-06 |
| ENSG00000144840 | 5.21e-04 | 1.47e-03 |
| ENSG00000144843 | 5.02e-01 | 5.82e-01 |
| ENSG00000144847 | 3.58e-15 | 7.69e-14 |
| ENSG00000144848 | 1.96e-03 | 4.83e-03 |
| ENSG00000144852 | 1.86e-01 | 2.54e-01 |
| ENSG00000144857 | 2.62e-06 | 1.15e-05 |
| ENSG00000144867 | 2.39e-14 | 4.37e-13 |
| ENSG00000144868 | 4.51e-14 | 7.78e-13 |
| ENSG00000144891 | 1.81e-14 | 3.39e-13 |
| ENSG00000144893 | 5.62e-08 | 3.31e-07 |
| ENSG00000144895 | 5.33e-06 | 2.21e-05 |
| ENSG00000144908 | 5.04e-03 | 1.12e-02 |
| ENSG00000144909 | 1.02e-08 | 6.78e-08 |
| ENSG00000144935 | 5.83e-06 | 2.40e-05 |
| ENSG00000144959 | 8.64e-01 | 8.97e-01 |
| ENSG00000145002 | 7.61e-02 | 1.19e-01 |
| ENSG00000145012 | 3.43e-08 | 2.09e-07 |
| ENSG00000145014 | 5.03e-02 | 8.34e-02 |
| ENSG00000145016 | 1.44e-01 | 2.05e-01 |
| ENSG00000145020 | 2.71e-06 | 1.18e-05 |
| ENSG00000145022 | 4.04e-08 | 2.44e-07 |
| ENSG00000145029 | 2.12e-01 | 2.84e-01 |
| ENSG00000145040 | 2.21e-21 | 2.23e-19 |
| ENSG00000145041 | 2.04e-08 | 1.29e-07 |
| ENSG00000145050 | 8.07e-08 | 4.62e-07 |
| ENSG00000145063 | 3.03e-02 | 5.36e-02 |
| ENSG00000145075 | 3.76e-02 | 6.48e-02 |
| ENSG00000145087 | 3.38e-09 | 2.44e-08 |
| ENSG00000145088 | 6.90e-11 | 6.66e-10 |
| ENSG00000145103 | 4.82e-05 | 1.67e-04 |
| ENSG00000145107 | 4.62e-12 | 5.50e-11 |
| ENSG00000145113 | 6.50e-07 | 3.17e-06 |
| ENSG00000145147 | 1.41e-10 | 1.29e-09 |
| ENSG00000145191 | 4.65e-08 | 2.78e-07 |
| ENSG00000145192 | 1.16e-04 | 3.72e-04 |
| ENSG00000145194 | 6.09e-14 | 1.03e-12 |
| ENSG00000145198 | 1.46e-06 | 6.67e-06 |
| ENSG00000145214 | 3.43e-02 | 5.98e-02 |
| ENSG00000145216 | 2.33e-02 | 4.27e-02 |
| ENSG00000145217 | 1.91e-03 | 4.72e-03 |
| ENSG00000145220 | 2.14e-17 | 7.41e-16 |
| ENSG00000145241 | 1.56e-02 | 3.01e-02 |
| ENSG00000145242 | 1.37e-13 | 2.17e-12 |
| ENSG00000145244 | 2.40e-20 | 1.73e-18 |
| ENSG00000145246 | 1.17e-01 | 1.71e-01 |
| ENSG00000145247 | 1.51e-06 | 6.89e-06 |
| ENSG00000145248 | 4.83e-14 | 8.30e-13 |
| ENSG00000145283 | 3.32e-02 | 5.81e-02 |
| ENSG00000145284 | 4.54e-02 | 7.63e-02 |
| ENSG00000145287 | 1.92e-16 | 5.40e-15 |
| ENSG00000145293 | 5.80e-19 | 2.91e-17 |
| ENSG00000145321 | 9.45e-02 | 1.43e-01 |
| ENSG00000145331 | 8.88e-01 | 9.16e-01 |
| ENSG00000145332 | 7.15e-03 | 1.52e-02 |
| ENSG00000145335 | 1.93e-09 | 1.45e-08 |
| ENSG00000145337 | 7.55e-02 | 1.18e-01 |
| ENSG00000145348 | 2.10e-01 | 2.82e-01 |
| ENSG00000145349 | 6.33e-13 | 8.84e-12 |
| ENSG00000145354 | 4.61e-02 | 7.73e-02 |
| ENSG00000145358 | 6.32e-04 | 1.75e-03 |
| ENSG00000145362 | 1.11e-21 | 1.23e-19 |
| ENSG00000145365 | 2.57e-01 | 3.34e-01 |
| ENSG00000145375 | 8.53e-05 | 2.82e-04 |
| ENSG00000145384 | 1.71e-15 | 3.88e-14 |
| ENSG00000145386 | 7.46e-12 | 8.54e-11 |
| ENSG00000145388 | 5.42e-03 | 1.19e-02 |
| ENSG00000145390 | 1.47e-06 | 6.74e-06 |
| ENSG00000145391 | 1.68e-03 | 4.21e-03 |
| ENSG00000145414 | 1.74e-04 | 5.40e-04 |
| ENSG00000145416 | 3.82e-03 | 8.75e-03 |
| ENSG00000145423 | 1.09e-04 | 3.54e-04 |
| ENSG00000145425 | 8.69e-01 | 9.02e-01 |
| ENSG00000145428 | 1.16e-02 | 2.33e-02 |
| ENSG00000145431 | 6.35e-01 | 7.05e-01 |
| ENSG00000145439 | 1.27e-03 | 3.29e-03 |
| ENSG00000145451 | 8.36e-02 | 1.29e-01 |
| ENSG00000145476 | 7.76e-10 | 6.24e-09 |
| ENSG00000145491 | 3.46e-01 | 4.28e-01 |
| ENSG00000145494 | 1.77e-01 | 2.44e-01 |
| ENSG00000145495 | 1.16e-01 | 1.71e-01 |
| ENSG00000145506 | 2.84e-17 | 9.48e-16 |
| ENSG00000145526 | 3.47e-07 | 1.78e-06 |
| ENSG00000145536 | 6.97e-01 | 7.59e-01 |
| ENSG00000145545 | 1.43e-08 | 9.31e-08 |
| ENSG00000145555 | 3.60e-05 | 1.29e-04 |
| ENSG00000145569 | 4.98e-01 | 5.79e-01 |
| ENSG00000145592 | 2.28e-11 | 2.38e-10 |
| ENSG00000145604 | 1.12e-13 | 1.80e-12 |
| ENSG00000145623 | 1.35e-01 | 1.94e-01 |
| ENSG00000145626 | 7.11e-01 | 7.71e-01 |
| ENSG00000145632 | 7.59e-03 | 1.60e-02 |
| ENSG00000145642 | 4.93e-04 | 1.40e-03 |
| ENSG00000145649 | 3.42e-08 | 2.09e-07 |
| ENSG00000145675 | 2.17e-03 | 5.30e-03 |
| ENSG00000145681 | 4.75e-20 | 3.13e-18 |
| ENSG00000145685 | 2.45e-06 | 1.08e-05 |
| ENSG00000145687 | 8.23e-17 | 2.49e-15 |
| ENSG00000145692 | 1.14e-01 | 1.68e-01 |
| ENSG00000145700 | 2.84e-06 | 1.24e-05 |
| ENSG00000145703 | 6.23e-16 | 1.56e-14 |
| ENSG00000145708 | 5.71e-18 | 2.22e-16 |
| ENSG00000145715 | 2.37e-04 | 7.17e-04 |
| ENSG00000145721 | 2.59e-05 | 9.48e-05 |
| ENSG00000145723 | 2.44e-04 | 7.37e-04 |
| ENSG00000145725 | 2.43e-03 | 5.84e-03 |
| ENSG00000145730 | 6.18e-01 | 6.90e-01 |
| ENSG00000145734 | 8.70e-02 | 1.34e-01 |
| ENSG00000145736 | 1.73e-02 | 3.29e-02 |
| ENSG00000145740 | 8.44e-03 | 1.76e-02 |
| ENSG00000145741 | 4.82e-03 | 1.07e-02 |
| ENSG00000145743 | 1.05e-07 | 5.88e-07 |
| ENSG00000145757 | 9.36e-01 | 9.52e-01 |
| ENSG00000145777 | 4.49e-09 | 3.18e-08 |
| ENSG00000145779 | 6.42e-03 | 1.38e-02 |
| ENSG00000145780 | 5.16e-08 | 3.06e-07 |
| ENSG00000145781 | 7.40e-02 | 1.16e-01 |
| ENSG00000145782 | 1.24e-01 | 1.81e-01 |
| ENSG00000145794 | 1.74e-09 | 1.32e-08 |
| ENSG00000145808 | 3.82e-04 | 1.11e-03 |
| ENSG00000145817 | 6.32e-04 | 1.75e-03 |
| ENSG00000145819 | 4.95e-01 | 5.76e-01 |
| ENSG00000145824 | 1.54e-03 | 3.89e-03 |
| ENSG00000145826 | 7.66e-02 | 1.20e-01 |
| ENSG00000145832 | 4.87e-05 | 1.69e-04 |
| ENSG00000145833 | 1.65e-03 | 4.14e-03 |
| ENSG00000145839 | 1.05e-01 | 1.57e-01 |
| ENSG00000145850 | 3.20e-06 | 1.38e-05 |
| ENSG00000145860 | 7.92e-01 | 8.39e-01 |
| ENSG00000145861 | 2.71e-15 | 5.94e-14 |
| ENSG00000145863 | 2.64e-01 | 3.41e-01 |
| ENSG00000145864 | 5.79e-08 | 3.41e-07 |
| ENSG00000145868 | 7.58e-02 | 1.19e-01 |
| ENSG00000145879 | 3.79e-02 | 6.52e-02 |
| ENSG00000145882 | 8.66e-06 | 3.46e-05 |
| ENSG00000145888 | 4.73e-02 | 7.90e-02 |
| ENSG00000145901 | 6.21e-09 | 4.29e-08 |
| ENSG00000145907 | 9.85e-11 | 9.23e-10 |
| ENSG00000145908 | 9.63e-02 | 1.46e-01 |
| ENSG00000145911 | 8.88e-01 | 9.17e-01 |
| ENSG00000145912 | 2.52e-09 | 1.86e-08 |
| ENSG00000145916 | 9.01e-01 | 9.27e-01 |
| ENSG00000145919 | 2.82e-14 | 5.06e-13 |
| ENSG00000145920 | 1.12e-06 | 5.23e-06 |
| ENSG00000145934 | 5.41e-07 | 2.68e-06 |
| ENSG00000145936 | 1.82e-09 | 1.37e-08 |
| ENSG00000145945 | 1.20e-05 | 4.67e-05 |
| ENSG00000145949 | 2.14e-02 | 3.97e-02 |
| ENSG00000145975 | 8.98e-01 | 9.24e-01 |
| ENSG00000145979 | 2.87e-10 | 2.49e-09 |
| ENSG00000145982 | 5.32e-01 | 6.11e-01 |
| ENSG00000145990 | 8.98e-04 | 2.40e-03 |
| ENSG00000145996 | 1.48e-06 | 6.76e-06 |
| ENSG00000146001 | 6.01e-01 | 6.74e-01 |
| ENSG00000146005 | 3.87e-02 | 6.63e-02 |
| ENSG00000146006 | 2.08e-05 | 7.74e-05 |
| ENSG00000146007 | 6.06e-04 | 1.68e-03 |
| ENSG00000146013 | 5.80e-15 | 1.18e-13 |
| ENSG00000146021 | 5.11e-03 | 1.13e-02 |
| ENSG00000146038 | 9.06e-01 | 9.30e-01 |
| ENSG00000146039 | 2.40e-20 | 1.73e-18 |
| ENSG00000146049 | 1.89e-02 | 3.56e-02 |
| ENSG00000146054 | 1.27e-01 | 1.84e-01 |
| ENSG00000146063 | 5.22e-03 | 1.15e-02 |
| ENSG00000146066 | 2.51e-05 | 9.23e-05 |
| ENSG00000146067 | 2.56e-04 | 7.68e-04 |
| ENSG00000146070 | 8.14e-01 | 8.58e-01 |
| ENSG00000146072 | 2.18e-03 | 5.30e-03 |
| ENSG00000146083 | 2.37e-08 | 1.49e-07 |
| ENSG00000146085 | 1.44e-14 | 2.75e-13 |
| ENSG00000146090 | 1.27e-17 | 4.55e-16 |
| ENSG00000146094 | 7.93e-02 | 1.23e-01 |
| ENSG00000146109 | 3.55e-08 | 2.16e-07 |
| ENSG00000146112 | 3.38e-01 | 4.20e-01 |
| ENSG00000146122 | 5.76e-19 | 2.90e-17 |
| ENSG00000146143 | 1.45e-14 | 2.78e-13 |
| ENSG00000146147 | 3.73e-02 | 6.43e-02 |
| ENSG00000146151 | 3.34e-20 | 2.30e-18 |
| ENSG00000146166 | 4.83e-03 | 1.07e-02 |
| ENSG00000146192 | 9.74e-07 | 4.62e-06 |
| ENSG00000146197 | 5.58e-05 | 1.91e-04 |
| ENSG00000146205 | 5.40e-06 | 2.23e-05 |
| ENSG00000146215 | 6.38e-01 | 7.08e-01 |
| ENSG00000146216 | 5.81e-01 | 6.56e-01 |
| ENSG00000146221 | 1.15e-02 | 2.30e-02 |
| ENSG00000146223 | 1.12e-17 | 4.07e-16 |
| ENSG00000146232 | 1.30e-04 | 4.14e-04 |
| ENSG00000146233 | 6.87e-09 | 4.71e-08 |
| ENSG00000146242 | 2.81e-02 | 5.03e-02 |
| ENSG00000146243 | 2.56e-09 | 1.88e-08 |
| ENSG00000146247 | 4.63e-01 | 5.45e-01 |
| ENSG00000146250 | 5.42e-03 | 1.19e-02 |
| ENSG00000146263 | 7.81e-12 | 8.89e-11 |
| ENSG00000146267 | 1.91e-04 | 5.90e-04 |
| ENSG00000146276 | 5.44e-01 | 6.22e-01 |
| ENSG00000146278 | 4.75e-03 | 1.06e-02 |
| ENSG00000146281 | 2.17e-03 | 5.28e-03 |
| ENSG00000146282 | 5.59e-02 | 9.13e-02 |
| ENSG00000146285 | 4.00e-02 | 6.83e-02 |
| ENSG00000146350 | 7.80e-01 | 8.30e-01 |
| ENSG00000146352 | 9.42e-15 | 1.85e-13 |
| ENSG00000146373 | 9.09e-01 | 9.33e-01 |
| ENSG00000146374 | 2.15e-07 | 1.14e-06 |
| ENSG00000146376 | 1.20e-02 | 2.40e-02 |
| ENSG00000146386 | 3.78e-03 | 8.67e-03 |
| ENSG00000146399 | 5.48e-02 | 8.96e-02 |
| ENSG00000146409 | 1.60e-02 | 3.07e-02 |
| ENSG00000146410 | 1.86e-15 | 4.19e-14 |
| ENSG00000146411 | 5.37e-09 | 3.75e-08 |
| ENSG00000146414 | 3.95e-01 | 4.78e-01 |
| ENSG00000146416 | 2.64e-07 | 1.39e-06 |
| ENSG00000146425 | 1.29e-01 | 1.87e-01 |
| ENSG00000146426 | 6.98e-02 | 1.10e-01 |
| ENSG00000146433 | 4.24e-02 | 7.19e-02 |
| ENSG00000146453 | 5.05e-01 | 5.85e-01 |
| ENSG00000146457 | 1.44e-01 | 2.05e-01 |
| ENSG00000146463 | 1.55e-02 | 2.99e-02 |
| ENSG00000146469 | 4.97e-17 | 1.58e-15 |
| ENSG00000146476 | 1.18e-01 | 1.73e-01 |
| ENSG00000146477 | 1.09e-15 | 2.58e-14 |
| ENSG00000146521 | 5.96e-07 | 2.93e-06 |
| ENSG00000146530 | 5.08e-05 | 1.76e-04 |
| ENSG00000146535 | 1.88e-03 | 4.64e-03 |
| ENSG00000146540 | 1.06e-07 | 5.97e-07 |
| ENSG00000146555 | 8.40e-07 | 4.03e-06 |
| ENSG00000146556 | 9.37e-01 | 9.53e-01 |
| ENSG00000146574 | 1.69e-04 | 5.28e-04 |
| ENSG00000146576 | 3.10e-05 | 1.12e-04 |
| ENSG00000146587 | 1.33e-05 | 5.15e-05 |
| ENSG00000146592 | 1.36e-01 | 1.95e-01 |
| ENSG00000146648 | 5.08e-08 | 3.02e-07 |
| ENSG00000146666 | 5.95e-10 | 4.88e-09 |
| ENSG00000146670 | 1.00e-17 | 3.68e-16 |
| ENSG00000146674 | 4.12e-09 | 2.94e-08 |
| ENSG00000146676 | 1.71e-01 | 2.38e-01 |
| ENSG00000146677 | 7.77e-08 | 4.46e-07 |
| ENSG00000146678 | 3.70e-04 | 1.07e-03 |
| ENSG00000146700 | 3.46e-16 | 9.18e-15 |
| ENSG00000146701 | 6.71e-05 | 2.26e-04 |
| ENSG00000146707 | 1.96e-01 | 2.66e-01 |
| ENSG00000146722 | 8.43e-01 | 8.81e-01 |
| ENSG00000146729 | 5.91e-01 | 6.65e-01 |
| ENSG00000146731 | 7.00e-22 | 8.32e-20 |
| ENSG00000146733 | 1.27e-19 | 7.49e-18 |
| ENSG00000146755 | 2.10e-07 | 1.12e-06 |
| ENSG00000146757 | 2.83e-04 | 8.40e-04 |
| ENSG00000146776 | 3.83e-02 | 6.58e-02 |
| ENSG00000146802 | 7.96e-01 | 8.42e-01 |
| ENSG00000146809 | 7.07e-01 | 7.68e-01 |
| ENSG00000146826 | 1.99e-01 | 2.69e-01 |
| ENSG00000146828 | 3.55e-12 | 4.33e-11 |
| ENSG00000146830 | 3.45e-09 | 2.49e-08 |
| ENSG00000146833 | 1.44e-06 | 6.61e-06 |
| ENSG00000146834 | 5.54e-06 | 2.29e-05 |
| ENSG00000146839 | 6.50e-03 | 1.40e-02 |
| ENSG00000146842 | 5.61e-05 | 1.92e-04 |
| ENSG00000146856 | 3.38e-02 | 5.90e-02 |
| ENSG00000146857 | 4.98e-01 | 5.79e-01 |
| ENSG00000146858 | 8.86e-25 | 2.58e-22 |
| ENSG00000146859 | 6.71e-14 | 1.12e-12 |
| ENSG00000146872 | 4.09e-03 | 9.29e-03 |
| ENSG00000146904 | 3.70e-11 | 3.74e-10 |
| ENSG00000146909 | 1.47e-19 | 8.61e-18 |
| ENSG00000146910 | 7.86e-03 | 1.65e-02 |
| ENSG00000146918 | 2.78e-18 | 1.17e-16 |
| ENSG00000146926 | 7.85e-01 | 8.34e-01 |
| ENSG00000146938 | 1.43e-15 | 3.30e-14 |
| ENSG00000146950 | 8.69e-01 | 9.02e-01 |
| ENSG00000146955 | 8.19e-01 | 8.62e-01 |
| ENSG00000146963 | 6.85e-08 | 3.97e-07 |
| ENSG00000146966 | 2.93e-16 | 7.90e-15 |
| ENSG00000147003 | 3.02e-01 | 3.83e-01 |
| ENSG00000147010 | 1.06e-08 | 7.00e-08 |
| ENSG00000147027 | 1.22e-08 | 8.02e-08 |
| ENSG00000147036 | 1.23e-15 | 2.88e-14 |
| ENSG00000147041 | 1.83e-04 | 5.66e-04 |
| ENSG00000147044 | 1.93e-04 | 5.95e-04 |
| ENSG00000147050 | 2.98e-03 | 7.01e-03 |
| ENSG00000147059 | 3.52e-02 | 6.12e-02 |
| ENSG00000147065 | 5.62e-02 | 9.16e-02 |
| ENSG00000147081 | 6.85e-10 | 5.55e-09 |
| ENSG00000147082 | 5.77e-01 | 6.52e-01 |
| ENSG00000147099 | 2.95e-14 | 5.27e-13 |
| ENSG00000147100 | 6.91e-04 | 1.89e-03 |
| ENSG00000147113 | 5.02e-01 | 5.83e-01 |
| ENSG00000147117 | 2.00e-06 | 8.94e-06 |
| ENSG00000147118 | 6.77e-09 | 4.65e-08 |
| ENSG00000147119 | 9.69e-03 | 1.98e-02 |
| ENSG00000147121 | 2.71e-02 | 4.88e-02 |
| ENSG00000147123 | 6.77e-02 | 1.08e-01 |
| ENSG00000147124 | 5.54e-01 | 6.31e-01 |
| ENSG00000147127 | 1.14e-01 | 1.68e-01 |
| ENSG00000147130 | 7.95e-11 | 7.59e-10 |
| ENSG00000147133 | 1.14e-05 | 4.45e-05 |
| ENSG00000147138 | 2.75e-08 | 1.70e-07 |
| ENSG00000147140 | 2.37e-22 | 3.11e-20 |
| ENSG00000147144 | 1.17e-10 | 1.08e-09 |
| ENSG00000147145 | 9.09e-02 | 1.39e-01 |
| ENSG00000147155 | 8.38e-06 | 3.37e-05 |
| ENSG00000147160 | 1.80e-02 | 3.41e-02 |
| ENSG00000147162 | 3.59e-10 | 3.06e-09 |
| ENSG00000147164 | 9.99e-01 | 9.99e-01 |
| ENSG00000147166 | 2.41e-01 | 3.17e-01 |
| ENSG00000147168 | 8.01e-04 | 2.17e-03 |
| ENSG00000147174 | 3.52e-04 | 1.03e-03 |
| ENSG00000147180 | 4.32e-01 | 5.15e-01 |
| ENSG00000147202 | 1.24e-03 | 3.22e-03 |
| ENSG00000147206 | 5.78e-01 | 6.53e-01 |
| ENSG00000147223 | 2.51e-05 | 9.24e-05 |
| ENSG00000147224 | 3.76e-16 | 9.90e-15 |
| ENSG00000147231 | 8.57e-01 | 8.92e-01 |
| ENSG00000147234 | 9.23e-01 | 9.43e-01 |
| ENSG00000147246 | 6.37e-03 | 1.37e-02 |
| ENSG00000147251 | 8.83e-03 | 1.83e-02 |
| ENSG00000147255 | 1.48e-02 | 2.88e-02 |
| ENSG00000147256 | 4.43e-13 | 6.41e-12 |
| ENSG00000147257 | 3.34e-05 | 1.20e-04 |
| ENSG00000147262 | 1.53e-15 | 3.50e-14 |
| ENSG00000147274 | 3.59e-18 | 1.47e-16 |
| ENSG00000147316 | 8.72e-01 | 9.04e-01 |
| ENSG00000147324 | 8.45e-01 | 8.83e-01 |
| ENSG00000147364 | 1.40e-04 | 4.43e-04 |
| ENSG00000147378 | 5.53e-02 | 9.03e-02 |
| ENSG00000147381 | 9.85e-03 | 2.01e-02 |
| ENSG00000147383 | 3.42e-14 | 6.03e-13 |
| ENSG00000147394 | 2.92e-05 | 1.06e-04 |
| ENSG00000147400 | 2.21e-07 | 1.17e-06 |
| ENSG00000147403 | 1.46e-05 | 5.59e-05 |
| ENSG00000147408 | 3.96e-04 | 1.14e-03 |
| ENSG00000147416 | 6.30e-01 | 7.01e-01 |
| ENSG00000147419 | 6.47e-05 | 2.19e-04 |
| ENSG00000147421 | 9.71e-01 | 9.78e-01 |
| ENSG00000147432 | 3.59e-02 | 6.22e-02 |
| ENSG00000147434 | 5.55e-08 | 3.28e-07 |
| ENSG00000147437 | 3.13e-02 | 5.53e-02 |
| ENSG00000147439 | 4.04e-01 | 4.87e-01 |
| ENSG00000147443 | 4.25e-06 | 1.79e-05 |
| ENSG00000147454 | 8.06e-03 | 1.69e-02 |
| ENSG00000147457 | 1.68e-06 | 7.60e-06 |
| ENSG00000147459 | 4.28e-04 | 1.22e-03 |
| ENSG00000147465 | 1.17e-06 | 5.47e-06 |
| ENSG00000147471 | 2.69e-10 | 2.35e-09 |
| ENSG00000147475 | 2.87e-02 | 5.12e-02 |
| ENSG00000147481 | 2.43e-04 | 7.33e-04 |
| ENSG00000147485 | 6.57e-01 | 7.24e-01 |
| ENSG00000147488 | 3.59e-04 | 1.05e-03 |
| ENSG00000147509 | 3.79e-04 | 1.10e-03 |
| ENSG00000147526 | 2.48e-02 | 4.51e-02 |
| ENSG00000147533 | 2.75e-01 | 3.53e-01 |
| ENSG00000147535 | 2.24e-02 | 4.13e-02 |
| ENSG00000147536 | 2.07e-13 | 3.17e-12 |
| ENSG00000147548 | 9.69e-01 | 9.77e-01 |
| ENSG00000147570 | 7.22e-01 | 7.81e-01 |
| ENSG00000147573 | 1.32e-10 | 1.21e-09 |
| ENSG00000147576 | 2.03e-23 | 3.81e-21 |
| ENSG00000147586 | 2.08e-01 | 2.79e-01 |
| ENSG00000147588 | 3.91e-19 | 2.05e-17 |
| ENSG00000147592 | 9.91e-08 | 5.59e-07 |
| ENSG00000147596 | 6.17e-02 | 9.93e-02 |
| ENSG00000147601 | 1.24e-02 | 2.47e-02 |
| ENSG00000147604 | 1.80e-05 | 6.80e-05 |
| ENSG00000147606 | 9.77e-07 | 4.63e-06 |
| ENSG00000147614 | 1.65e-12 | 2.15e-11 |
| ENSG00000147642 | 1.41e-10 | 1.29e-09 |
| ENSG00000147647 | 2.10e-01 | 2.82e-01 |
| ENSG00000147649 | 6.82e-08 | 3.96e-07 |
| ENSG00000147650 | 2.23e-02 | 4.11e-02 |
| ENSG00000147654 | 2.07e-01 | 2.78e-01 |
| ENSG00000147655 | 2.34e-25 | 8.73e-23 |
| ENSG00000147669 | 5.94e-05 | 2.03e-04 |
| ENSG00000147676 | 9.45e-01 | 9.60e-01 |
| ENSG00000147677 | 3.57e-12 | 4.35e-11 |
| ENSG00000147679 | 3.09e-09 | 2.25e-08 |
| ENSG00000147684 | 5.83e-03 | 1.27e-02 |
| ENSG00000147687 | 5.34e-08 | 3.16e-07 |
| ENSG00000147689 | 5.52e-05 | 1.89e-04 |
| ENSG00000147697 | 4.76e-14 | 8.19e-13 |
| ENSG00000147724 | 1.36e-26 | 8.37e-24 |
| ENSG00000147753 | 5.03e-02 | 8.33e-02 |
| ENSG00000147761 | 1.88e-01 | 2.58e-01 |
| ENSG00000147789 | 9.32e-15 | 1.84e-13 |
| ENSG00000147799 | 3.43e-13 | 5.04e-12 |
| ENSG00000147804 | 2.62e-04 | 7.85e-04 |
| ENSG00000147813 | 6.39e-01 | 7.08e-01 |
| ENSG00000147852 | 4.03e-08 | 2.43e-07 |
| ENSG00000147853 | 5.44e-06 | 2.25e-05 |
| ENSG00000147854 | 2.39e-07 | 1.26e-06 |
| ENSG00000147862 | 5.96e-01 | 6.69e-01 |
| ENSG00000147869 | 2.62e-02 | 4.74e-02 |
| ENSG00000147872 | 3.39e-02 | 5.91e-02 |
| ENSG00000147874 | 9.31e-13 | 1.26e-11 |
| ENSG00000147883 | 7.77e-23 | 1.22e-20 |
| ENSG00000147889 | 6.35e-14 | 1.07e-12 |
| ENSG00000147894 | 1.60e-03 | 4.02e-03 |
| ENSG00000147896 | 3.38e-01 | 4.20e-01 |
| ENSG00000147905 | 4.28e-12 | 5.12e-11 |
| ENSG00000147912 | 5.35e-01 | 6.13e-01 |
| ENSG00000147955 | 2.14e-19 | 1.20e-17 |
| ENSG00000147996 | 1.04e-12 | 1.39e-11 |
| ENSG00000148019 | 1.00e-08 | 6.67e-08 |
| ENSG00000148053 | 1.12e-07 | 6.24e-07 |
| ENSG00000148057 | 4.01e-04 | 1.15e-03 |
| ENSG00000148082 | 2.88e-01 | 3.68e-01 |
| ENSG00000148090 | 3.53e-11 | 3.58e-10 |
| ENSG00000148110 | 1.69e-05 | 6.40e-05 |
| ENSG00000148120 | 1.71e-02 | 3.26e-02 |
| ENSG00000148123 | 3.38e-01 | 4.20e-01 |
| ENSG00000148143 | 9.55e-02 | 1.45e-01 |
| ENSG00000148153 | 6.86e-01 | 7.50e-01 |
| ENSG00000148154 | 1.80e-03 | 4.47e-03 |
| ENSG00000148156 | 5.51e-02 | 9.00e-02 |
| ENSG00000148158 | 1.47e-03 | 3.73e-03 |
| ENSG00000148175 | 7.86e-06 | 3.17e-05 |
| ENSG00000148180 | 1.60e-21 | 1.70e-19 |
| ENSG00000148187 | 1.43e-05 | 5.50e-05 |
| ENSG00000148200 | 5.56e-04 | 1.56e-03 |
| ENSG00000148204 | 5.80e-01 | 6.55e-01 |
| ENSG00000148215 | 5.70e-01 | 6.46e-01 |
| ENSG00000148218 | 2.60e-12 | 3.26e-11 |
| ENSG00000148219 | 1.71e-03 | 4.28e-03 |
| ENSG00000148225 | 2.10e-08 | 1.33e-07 |
| ENSG00000148229 | 5.22e-11 | 5.14e-10 |
| ENSG00000148248 | 4.95e-06 | 2.07e-05 |
| ENSG00000148288 | 5.37e-01 | 6.16e-01 |
| ENSG00000148290 | 1.70e-03 | 4.25e-03 |
| ENSG00000148291 | 3.24e-05 | 1.16e-04 |
| ENSG00000148296 | 6.41e-07 | 3.14e-06 |
| ENSG00000148297 | 1.34e-03 | 3.43e-03 |
| ENSG00000148300 | 4.88e-10 | 4.06e-09 |
| ENSG00000148303 | 6.62e-08 | 3.85e-07 |
| ENSG00000148308 | 4.82e-07 | 2.41e-06 |
| ENSG00000148331 | 5.66e-06 | 2.33e-05 |
| ENSG00000148334 | 4.92e-06 | 2.05e-05 |
| ENSG00000148335 | 1.81e-21 | 1.89e-19 |
| ENSG00000148337 | 2.02e-04 | 6.20e-04 |
| ENSG00000148339 | 2.69e-01 | 3.47e-01 |
| ENSG00000148341 | 1.61e-01 | 2.26e-01 |
| ENSG00000148343 | 4.85e-10 | 4.04e-09 |
| ENSG00000148344 | 1.94e-06 | 8.70e-06 |
| ENSG00000148346 | 2.06e-04 | 6.29e-04 |
| ENSG00000148356 | 1.34e-08 | 8.76e-08 |
| ENSG00000148357 | 1.32e-14 | 2.54e-13 |
| ENSG00000148358 | 6.72e-01 | 7.37e-01 |
| ENSG00000148362 | 1.88e-09 | 1.42e-08 |
| ENSG00000148377 | 4.16e-02 | 7.07e-02 |
| ENSG00000148384 | 1.02e-08 | 6.80e-08 |
| ENSG00000148396 | 8.23e-03 | 1.72e-02 |
| ENSG00000148399 | 2.50e-14 | 4.55e-13 |
| ENSG00000148400 | 6.87e-06 | 2.79e-05 |
| ENSG00000148408 | 3.35e-08 | 2.05e-07 |
| ENSG00000148411 | 1.42e-02 | 2.77e-02 |
| ENSG00000148426 | 1.59e-01 | 2.23e-01 |
| ENSG00000148429 | 4.42e-12 | 5.27e-11 |
| ENSG00000148444 | 1.77e-01 | 2.45e-01 |
| ENSG00000148450 | 8.05e-01 | 8.51e-01 |
| ENSG00000148459 | 1.95e-02 | 3.66e-02 |
| ENSG00000148468 | 3.14e-03 | 7.34e-03 |
| ENSG00000148481 | 9.41e-02 | 1.43e-01 |
| ENSG00000148482 | 4.49e-11 | 4.48e-10 |
| ENSG00000148483 | 4.91e-20 | 3.21e-18 |
| ENSG00000148484 | 7.74e-05 | 2.58e-04 |
| ENSG00000148488 | 1.43e-11 | 1.55e-10 |
| ENSG00000148498 | 3.80e-03 | 8.70e-03 |
| ENSG00000148513 | 8.50e-04 | 2.28e-03 |
| ENSG00000148516 | 6.00e-05 | 2.04e-04 |
| ENSG00000148541 | 7.66e-10 | 6.17e-09 |
| ENSG00000148572 | 1.30e-03 | 3.34e-03 |
| ENSG00000148584 | 4.86e-08 | 2.90e-07 |
| ENSG00000148600 | 6.25e-02 | 1.00e-01 |
| ENSG00000148602 | 3.07e-01 | 3.88e-01 |
| ENSG00000148604 | 4.27e-05 | 1.50e-04 |
| ENSG00000148606 | 5.21e-14 | 8.89e-13 |
| ENSG00000148634 | 2.25e-01 | 2.98e-01 |
| ENSG00000148655 | 5.68e-01 | 6.44e-01 |
| ENSG00000148660 | 1.47e-08 | 9.53e-08 |
| ENSG00000148672 | 5.96e-03 | 1.29e-02 |
| ENSG00000148677 | 5.02e-04 | 1.42e-03 |
| ENSG00000148680 | 3.51e-18 | 1.44e-16 |
| ENSG00000148688 | 7.90e-02 | 1.23e-01 |
| ENSG00000148690 | 5.33e-01 | 6.12e-01 |
| ENSG00000148700 | 2.35e-01 | 3.10e-01 |
| ENSG00000148702 | 5.32e-11 | 5.22e-10 |
| ENSG00000148704 | 1.10e-01 | 1.64e-01 |
| ENSG00000148719 | 7.75e-01 | 8.25e-01 |
| ENSG00000148730 | 5.21e-09 | 3.65e-08 |
| ENSG00000148734 | 1.13e-18 | 5.25e-17 |
| ENSG00000148735 | 1.97e-14 | 3.67e-13 |
| ENSG00000148737 | 4.92e-07 | 2.45e-06 |
| ENSG00000148773 | 1.93e-08 | 1.23e-07 |
| ENSG00000148795 | 2.35e-01 | 3.10e-01 |
| ENSG00000148798 | 6.06e-18 | 2.34e-16 |
| ENSG00000148803 | 1.11e-01 | 1.64e-01 |
| ENSG00000148814 | 7.61e-04 | 2.07e-03 |
| ENSG00000148824 | 3.90e-07 | 1.98e-06 |
| ENSG00000148826 | 7.77e-01 | 8.27e-01 |
| ENSG00000148832 | 2.84e-06 | 1.23e-05 |
| ENSG00000148834 | 9.22e-03 | 1.90e-02 |
| ENSG00000148835 | 5.72e-01 | 6.48e-01 |
| ENSG00000148840 | 1.22e-20 | 9.78e-19 |
| ENSG00000148841 | 7.72e-12 | 8.80e-11 |
| ENSG00000148842 | 3.83e-21 | 3.54e-19 |
| ENSG00000148843 | 7.62e-15 | 1.53e-13 |
| ENSG00000148848 | 1.57e-21 | 1.69e-19 |
| ENSG00000148908 | 3.54e-08 | 2.15e-07 |
| ENSG00000148925 | 3.19e-01 | 4.01e-01 |
| ENSG00000148926 | 4.46e-02 | 7.51e-02 |
| ENSG00000148935 | 2.85e-13 | 4.25e-12 |
| ENSG00000148942 | 3.49e-05 | 1.25e-04 |
| ENSG00000148943 | 5.04e-01 | 5.85e-01 |
| ENSG00000148948 | 6.46e-07 | 3.16e-06 |
| ENSG00000148950 | 9.28e-02 | 1.41e-01 |
| ENSG00000148965 | 2.53e-05 | 9.30e-05 |
| ENSG00000148985 | 2.20e-06 | 9.75e-06 |
| ENSG00000149016 | 6.97e-11 | 6.72e-10 |
| ENSG00000149021 | 6.98e-02 | 1.10e-01 |
| ENSG00000149043 | 3.28e-05 | 1.18e-04 |
| ENSG00000149050 | 3.04e-02 | 5.38e-02 |
| ENSG00000149054 | 2.26e-04 | 6.87e-04 |
| ENSG00000149084 | 1.76e-05 | 6.66e-05 |
| ENSG00000149089 | 1.07e-02 | 2.16e-02 |
| ENSG00000149090 | 8.81e-14 | 1.44e-12 |
| ENSG00000149091 | 4.86e-11 | 4.81e-10 |
| ENSG00000149100 | 7.21e-10 | 5.83e-09 |
| ENSG00000149115 | 9.54e-01 | 9.66e-01 |
| ENSG00000149124 | 2.50e-04 | 7.53e-04 |
| ENSG00000149131 | 4.39e-04 | 1.25e-03 |
| ENSG00000149136 | 1.68e-09 | 1.28e-08 |
| ENSG00000149150 | 8.69e-11 | 8.24e-10 |
| ENSG00000149177 | 7.64e-02 | 1.19e-01 |
| ENSG00000149179 | 2.51e-08 | 1.57e-07 |
| ENSG00000149182 | 8.12e-02 | 1.26e-01 |
| ENSG00000149187 | 1.04e-02 | 2.12e-02 |
| ENSG00000149196 | 9.01e-10 | 7.17e-09 |
| ENSG00000149201 | 1.99e-01 | 2.70e-01 |
| ENSG00000149212 | 1.84e-01 | 2.52e-01 |
| ENSG00000149218 | 3.47e-23 | 5.99e-21 |
| ENSG00000149231 | 9.02e-01 | 9.27e-01 |
| ENSG00000149243 | 3.54e-24 | 8.58e-22 |
| ENSG00000149256 | 2.88e-06 | 1.25e-05 |
| ENSG00000149257 | 1.47e-17 | 5.22e-16 |
| ENSG00000149260 | 1.85e-13 | 2.85e-12 |
| ENSG00000149262 | 8.69e-04 | 2.33e-03 |
| ENSG00000149269 | 9.36e-02 | 1.42e-01 |
| ENSG00000149273 | 5.02e-08 | 2.99e-07 |
| ENSG00000149289 | 7.90e-10 | 6.35e-09 |
| ENSG00000149292 | 7.53e-09 | 5.14e-08 |
| ENSG00000149294 | 2.37e-19 | 1.30e-17 |
| ENSG00000149295 | 1.97e-03 | 4.85e-03 |
| ENSG00000149300 | 7.29e-01 | 7.86e-01 |
| ENSG00000149305 | 8.40e-13 | 1.15e-11 |
| ENSG00000149308 | 4.81e-01 | 5.64e-01 |
| ENSG00000149311 | 9.14e-01 | 9.36e-01 |
| ENSG00000149313 | 9.17e-09 | 6.15e-08 |
| ENSG00000149328 | 4.70e-02 | 7.85e-02 |
| ENSG00000149346 | 7.95e-06 | 3.20e-05 |
| ENSG00000149357 | 1.75e-01 | 2.41e-01 |
| ENSG00000149380 | 4.66e-14 | 8.02e-13 |
| ENSG00000149403 | 6.22e-06 | 2.54e-05 |
| ENSG00000149418 | 9.76e-03 | 2.00e-02 |
| ENSG00000149428 | 1.64e-05 | 6.24e-05 |
| ENSG00000149435 | 2.81e-01 | 3.60e-01 |
| ENSG00000149443 | 2.63e-01 | 3.40e-01 |
| ENSG00000149451 | 4.86e-13 | 6.97e-12 |
| ENSG00000149452 | 5.70e-01 | 6.46e-01 |
| ENSG00000149474 | 3.12e-02 | 5.51e-02 |
| ENSG00000149476 | 4.79e-06 | 2.00e-05 |
| ENSG00000149480 | 1.68e-06 | 7.61e-06 |
| ENSG00000149483 | 1.08e-02 | 2.18e-02 |
| ENSG00000149485 | 4.84e-09 | 3.41e-08 |
| ENSG00000149488 | 9.91e-01 | 9.94e-01 |
| ENSG00000149489 | 7.46e-03 | 1.58e-02 |
| ENSG00000149499 | 8.39e-04 | 2.26e-03 |
| ENSG00000149503 | 1.50e-11 | 1.62e-10 |
| ENSG00000149506 | 2.96e-02 | 5.26e-02 |
| ENSG00000149516 | 3.64e-01 | 4.46e-01 |
| ENSG00000149527 | 2.93e-01 | 3.73e-01 |
| ENSG00000149531 | 7.45e-05 | 2.49e-04 |
| ENSG00000149532 | 6.36e-02 | 1.02e-01 |
| ENSG00000149534 | 5.69e-13 | 8.02e-12 |
| ENSG00000149541 | 1.86e-03 | 4.61e-03 |
| ENSG00000149547 | 4.74e-03 | 1.06e-02 |
| ENSG00000149548 | 1.07e-02 | 2.16e-02 |
| ENSG00000149554 | 5.37e-16 | 1.37e-14 |
| ENSG00000149557 | 6.53e-10 | 5.31e-09 |
| ENSG00000149564 | 6.73e-02 | 1.07e-01 |
| ENSG00000149571 | 3.89e-09 | 2.79e-08 |
| ENSG00000149573 | 8.00e-02 | 1.24e-01 |
| ENSG00000149575 | 2.05e-18 | 8.89e-17 |
| ENSG00000149577 | 3.17e-09 | 2.30e-08 |
| ENSG00000149582 | 3.57e-09 | 2.57e-08 |
| ENSG00000149591 | 3.16e-05 | 1.14e-04 |
| ENSG00000149596 | 8.06e-09 | 5.46e-08 |
| ENSG00000149599 | 3.04e-06 | 1.31e-05 |
| ENSG00000149600 | 1.26e-03 | 3.26e-03 |
| ENSG00000149609 | 3.03e-13 | 4.49e-12 |
| ENSG00000149633 | 1.84e-05 | 6.93e-05 |
| ENSG00000149634 | 2.11e-05 | 7.87e-05 |
| ENSG00000149635 | 2.63e-01 | 3.41e-01 |
| ENSG00000149636 | 3.02e-17 | 1.00e-15 |
| ENSG00000149639 | 1.02e-05 | 4.03e-05 |
| ENSG00000149646 | 8.61e-04 | 2.31e-03 |
| ENSG00000149651 | 6.42e-04 | 1.77e-03 |
| ENSG00000149654 | 1.31e-08 | 8.57e-08 |
| ENSG00000149656 | 5.65e-01 | 6.42e-01 |
| ENSG00000149657 | 3.59e-12 | 4.37e-11 |
| ENSG00000149658 | 1.60e-20 | 1.21e-18 |
| ENSG00000149679 | 6.23e-09 | 4.31e-08 |
| ENSG00000149716 | 1.24e-06 | 5.76e-06 |
| ENSG00000149735 | 9.32e-01 | 9.49e-01 |
| ENSG00000149742 | 4.86e-01 | 5.68e-01 |
| ENSG00000149743 | 8.94e-01 | 9.21e-01 |
| ENSG00000149761 | 3.97e-04 | 1.15e-03 |
| ENSG00000149781 | 1.52e-06 | 6.92e-06 |
| ENSG00000149782 | 2.24e-01 | 2.97e-01 |
| ENSG00000149792 | 2.85e-01 | 3.65e-01 |
| ENSG00000149798 | 1.69e-05 | 6.38e-05 |
| ENSG00000149806 | 4.70e-01 | 5.53e-01 |
| ENSG00000149809 | 3.28e-07 | 1.69e-06 |
| ENSG00000149823 | 4.61e-01 | 5.44e-01 |
| ENSG00000149922 | 9.13e-09 | 6.13e-08 |
| ENSG00000149923 | 3.33e-05 | 1.19e-04 |
| ENSG00000149925 | 8.04e-01 | 8.49e-01 |
| ENSG00000149926 | 3.60e-02 | 6.24e-02 |
| ENSG00000149927 | 1.30e-01 | 1.89e-01 |
| ENSG00000149929 | 2.73e-01 | 3.51e-01 |
| ENSG00000149930 | 4.87e-01 | 5.69e-01 |
| ENSG00000149932 | 7.12e-03 | 1.51e-02 |
| ENSG00000149948 | 6.47e-05 | 2.19e-04 |
| ENSG00000149968 | 1.24e-19 | 7.32e-18 |
| ENSG00000149970 | 3.36e-13 | 4.95e-12 |
| ENSG00000149972 | 9.36e-07 | 4.45e-06 |
| ENSG00000150045 | 7.54e-13 | 1.04e-11 |
| ENSG00000150048 | 7.56e-04 | 2.05e-03 |
| ENSG00000150051 | 3.08e-02 | 5.44e-02 |
| ENSG00000150054 | 1.08e-09 | 8.47e-09 |
| ENSG00000150093 | 9.15e-01 | 9.37e-01 |
| ENSG00000150175 | 2.04e-01 | 2.75e-01 |
| ENSG00000150201 | 9.27e-08 | 5.26e-07 |
| ENSG00000150275 | 7.65e-04 | 2.08e-03 |
| ENSG00000150276 | 1.42e-02 | 2.77e-02 |
| ENSG00000150281 | 3.86e-03 | 8.83e-03 |
| ENSG00000150316 | 7.70e-01 | 8.21e-01 |
| ENSG00000150337 | 1.75e-08 | 1.12e-07 |
| ENSG00000150347 | 1.11e-03 | 2.90e-03 |
| ENSG00000150361 | 2.00e-09 | 1.50e-08 |
| ENSG00000150394 | 6.44e-01 | 7.13e-01 |
| ENSG00000150401 | 8.44e-13 | 1.16e-11 |
| ENSG00000150403 | 7.97e-05 | 2.65e-04 |
| ENSG00000150433 | 4.35e-01 | 5.18e-01 |
| ENSG00000150455 | 3.80e-02 | 6.54e-02 |
| ENSG00000150456 | 3.59e-14 | 6.31e-13 |
| ENSG00000150457 | 4.38e-01 | 5.21e-01 |
| ENSG00000150459 | 1.01e-07 | 5.70e-07 |
| ENSG00000150471 | 4.76e-19 | 2.45e-17 |
| ENSG00000150477 | 6.88e-03 | 1.47e-02 |
| ENSG00000150510 | 1.25e-06 | 5.79e-06 |
| ENSG00000150526 | 9.35e-05 | 3.06e-04 |
| ENSG00000150527 | 1.61e-07 | 8.78e-07 |
| ENSG00000150540 | 3.43e-03 | 7.95e-03 |
| ENSG00000150551 | 5.74e-02 | 9.33e-02 |
| ENSG00000150556 | 1.97e-04 | 6.04e-04 |
| ENSG00000150593 | 1.07e-22 | 1.62e-20 |
| ENSG00000150594 | 6.30e-10 | 5.13e-09 |
| ENSG00000150625 | 6.25e-15 | 1.27e-13 |
| ENSG00000150627 | 1.83e-16 | 5.18e-15 |
| ENSG00000150628 | 6.84e-09 | 4.70e-08 |
| ENSG00000150630 | 7.28e-01 | 7.85e-01 |
| ENSG00000150636 | 3.12e-01 | 3.93e-01 |
| ENSG00000150637 | 1.47e-05 | 5.63e-05 |
| ENSG00000150656 | 4.66e-01 | 5.49e-01 |
| ENSG00000150667 | 3.20e-14 | 5.68e-13 |
| ENSG00000150672 | 6.20e-12 | 7.21e-11 |
| ENSG00000150676 | 4.79e-05 | 1.67e-04 |
| ENSG00000150681 | 4.20e-06 | 1.77e-05 |
| ENSG00000150687 | 8.69e-01 | 9.01e-01 |
| ENSG00000150712 | 1.08e-02 | 2.18e-02 |
| ENSG00000150722 | 1.58e-01 | 2.21e-01 |
| ENSG00000150750 | 6.94e-04 | 1.90e-03 |
| ENSG00000150753 | 2.23e-14 | 4.11e-13 |
| ENSG00000150756 | 3.97e-04 | 1.14e-03 |
| ENSG00000150760 | 1.60e-06 | 7.28e-06 |
| ENSG00000150764 | 2.07e-08 | 1.31e-07 |
| ENSG00000150768 | 6.69e-04 | 1.84e-03 |
| ENSG00000150773 | 3.52e-05 | 1.26e-04 |
| ENSG00000150776 | 2.30e-04 | 6.98e-04 |
| ENSG00000150779 | 6.43e-02 | 1.03e-01 |
| ENSG00000150782 | 2.82e-09 | 2.06e-08 |
| ENSG00000150783 | 4.44e-01 | 5.27e-01 |
| ENSG00000150787 | 3.34e-06 | 1.43e-05 |
| ENSG00000150867 | 1.80e-02 | 3.40e-02 |
| ENSG00000150873 | 4.38e-02 | 7.39e-02 |
| ENSG00000150893 | 4.05e-01 | 4.88e-01 |
| ENSG00000150907 | 4.81e-08 | 2.87e-07 |
| ENSG00000150938 | 1.25e-01 | 1.82e-01 |
| ENSG00000150961 | 3.41e-02 | 5.95e-02 |
| ENSG00000150967 | 2.29e-01 | 3.03e-01 |
| ENSG00000150977 | 3.43e-04 | 1.00e-03 |
| ENSG00000150990 | 3.25e-13 | 4.80e-12 |
| ENSG00000150991 | 6.37e-02 | 1.02e-01 |
| ENSG00000150995 | 3.11e-10 | 2.68e-09 |
| ENSG00000151005 | 7.15e-01 | 7.75e-01 |
| ENSG00000151006 | 2.96e-07 | 1.54e-06 |
| ENSG00000151012 | 1.38e-15 | 3.21e-14 |
| ENSG00000151014 | 1.04e-01 | 1.56e-01 |
| ENSG00000151023 | 1.14e-02 | 2.29e-02 |
| ENSG00000151025 | 1.39e-04 | 4.41e-04 |
| ENSG00000151062 | 1.06e-01 | 1.58e-01 |
| ENSG00000151065 | 1.88e-02 | 3.55e-02 |
| ENSG00000151067 | 8.34e-01 | 8.74e-01 |
| ENSG00000151090 | 4.40e-19 | 2.27e-17 |
| ENSG00000151092 | 1.48e-01 | 2.10e-01 |
| ENSG00000151093 | 8.73e-01 | 9.05e-01 |
| ENSG00000151116 | 1.86e-05 | 6.98e-05 |
| ENSG00000151117 | 3.18e-01 | 3.99e-01 |
| ENSG00000151131 | 9.65e-14 | 1.57e-12 |
| ENSG00000151135 | 3.61e-02 | 6.25e-02 |
| ENSG00000151136 | 2.77e-03 | 6.57e-03 |
| ENSG00000151148 | 1.59e-06 | 7.24e-06 |
| ENSG00000151150 | 4.27e-19 | 2.21e-17 |
| ENSG00000151151 | 9.35e-05 | 3.06e-04 |
| ENSG00000151164 | 7.86e-02 | 1.22e-01 |
| ENSG00000151176 | 4.64e-08 | 2.77e-07 |
| ENSG00000151208 | 2.65e-06 | 1.16e-05 |
| ENSG00000151224 | 4.86e-06 | 2.03e-05 |
| ENSG00000151229 | 1.02e-15 | 2.45e-14 |
| ENSG00000151233 | 2.27e-03 | 5.52e-03 |
| ENSG00000151239 | 2.00e-01 | 2.71e-01 |
| ENSG00000151240 | 3.17e-04 | 9.33e-04 |
| ENSG00000151247 | 8.72e-01 | 9.04e-01 |
| ENSG00000151276 | 4.25e-08 | 2.56e-07 |
| ENSG00000151287 | 2.36e-09 | 1.75e-08 |
| ENSG00000151292 | 3.43e-04 | 1.00e-03 |
| ENSG00000151303 | 4.23e-03 | 9.56e-03 |
| ENSG00000151304 | 4.70e-05 | 1.64e-04 |
| ENSG00000151320 | 9.77e-08 | 5.51e-07 |
| ENSG00000151322 | 2.18e-10 | 1.93e-09 |
| ENSG00000151327 | 5.05e-09 | 3.55e-08 |
| ENSG00000151332 | 5.61e-01 | 6.37e-01 |
| ENSG00000151338 | 9.86e-12 | 1.10e-10 |
| ENSG00000151348 | 2.66e-03 | 6.35e-03 |
| ENSG00000151353 | 8.26e-05 | 2.74e-04 |
| ENSG00000151360 | 2.79e-02 | 5.00e-02 |
| ENSG00000151364 | 3.22e-14 | 5.71e-13 |
| ENSG00000151365 | 7.61e-03 | 1.60e-02 |
| ENSG00000151366 | 1.49e-02 | 2.88e-02 |
| ENSG00000151376 | 5.30e-02 | 8.71e-02 |
| ENSG00000151379 | 3.24e-02 | 5.69e-02 |
| ENSG00000151388 | 3.35e-24 | 8.15e-22 |
| ENSG00000151413 | 2.82e-01 | 3.62e-01 |
| ENSG00000151414 | 1.08e-05 | 4.24e-05 |
| ENSG00000151422 | 1.50e-02 | 2.91e-02 |
| ENSG00000151445 | 4.14e-03 | 9.38e-03 |
| ENSG00000151458 | 2.70e-01 | 3.48e-01 |
| ENSG00000151461 | 9.13e-01 | 9.36e-01 |
| ENSG00000151465 | 1.32e-12 | 1.75e-11 |
| ENSG00000151466 | 1.80e-02 | 3.41e-02 |
| ENSG00000151468 | 1.01e-05 | 3.99e-05 |
| ENSG00000151470 | 1.08e-08 | 7.18e-08 |
| ENSG00000151474 | 1.01e-01 | 1.52e-01 |
| ENSG00000151475 | 2.88e-01 | 3.68e-01 |
| ENSG00000151490 | 6.44e-04 | 1.78e-03 |
| ENSG00000151491 | 3.50e-07 | 1.79e-06 |
| ENSG00000151498 | 3.25e-09 | 2.35e-08 |
| ENSG00000151500 | 8.64e-01 | 8.98e-01 |
| ENSG00000151502 | 1.66e-01 | 2.32e-01 |
| ENSG00000151503 | 2.40e-19 | 1.32e-17 |
| ENSG00000151532 | 9.72e-01 | 9.79e-01 |
| ENSG00000151552 | 3.82e-04 | 1.11e-03 |
| ENSG00000151553 | 5.78e-01 | 6.53e-01 |
| ENSG00000151572 | 7.39e-05 | 2.47e-04 |
| ENSG00000151575 | 3.99e-01 | 4.82e-01 |
| ENSG00000151576 | 3.21e-12 | 3.95e-11 |
| ENSG00000151577 | 4.56e-01 | 5.39e-01 |
| ENSG00000151611 | 1.42e-04 | 4.48e-04 |
| ENSG00000151612 | 2.20e-01 | 2.94e-01 |
| ENSG00000151615 | 2.56e-01 | 3.32e-01 |
| ENSG00000151617 | 4.49e-15 | 9.40e-14 |
| ENSG00000151623 | 2.83e-21 | 2.74e-19 |
| ENSG00000151631 | 1.12e-04 | 3.60e-04 |
| ENSG00000151632 | 5.52e-15 | 1.13e-13 |
| ENSG00000151640 | 3.79e-01 | 4.62e-01 |
| ENSG00000151650 | 3.30e-09 | 2.38e-08 |
| ENSG00000151651 | 1.70e-07 | 9.23e-07 |
| ENSG00000151655 | 3.52e-01 | 4.34e-01 |
| ENSG00000151657 | 7.31e-01 | 7.88e-01 |
| ENSG00000151665 | 8.77e-03 | 1.82e-02 |
| ENSG00000151687 | 3.30e-01 | 4.12e-01 |
| ENSG00000151689 | 4.86e-01 | 5.68e-01 |
| ENSG00000151690 | 8.27e-01 | 8.68e-01 |
| ENSG00000151692 | 4.64e-07 | 2.32e-06 |
| ENSG00000151693 | 1.36e-07 | 7.51e-07 |
| ENSG00000151694 | 1.06e-09 | 8.36e-09 |
| ENSG00000151702 | 2.99e-11 | 3.07e-10 |
| ENSG00000151704 | 2.49e-01 | 3.25e-01 |
| ENSG00000151715 | 2.88e-09 | 2.11e-08 |
| ENSG00000151718 | 1.20e-01 | 1.76e-01 |
| ENSG00000151725 | 6.66e-08 | 3.88e-07 |
| ENSG00000151726 | 7.20e-03 | 1.53e-02 |
| ENSG00000151729 | 9.33e-07 | 4.44e-06 |
| ENSG00000151743 | 1.86e-01 | 2.54e-01 |
| ENSG00000151746 | 9.56e-15 | 1.88e-13 |
| ENSG00000151748 | 2.06e-01 | 2.77e-01 |
| ENSG00000151773 | 3.56e-03 | 8.21e-03 |
| ENSG00000151778 | 6.66e-01 | 7.32e-01 |
| ENSG00000151779 | 2.35e-03 | 5.68e-03 |
| ENSG00000151789 | 1.59e-05 | 6.05e-05 |
| ENSG00000151790 | 4.89e-08 | 2.92e-07 |
| ENSG00000151806 | 6.66e-03 | 1.43e-02 |
| ENSG00000151812 | 1.26e-01 | 1.83e-01 |
| ENSG00000151834 | 7.35e-07 | 3.56e-06 |
| ENSG00000151835 | 3.70e-03 | 8.51e-03 |
| ENSG00000151838 | 3.17e-02 | 5.58e-02 |
| ENSG00000151846 | 4.22e-15 | 8.88e-14 |
| ENSG00000151849 | 7.07e-13 | 9.83e-12 |
| ENSG00000151876 | 3.66e-07 | 1.87e-06 |
| ENSG00000151881 | 1.35e-09 | 1.04e-08 |
| ENSG00000151882 | 1.45e-14 | 2.78e-13 |
| ENSG00000151883 | 7.84e-07 | 3.78e-06 |
| ENSG00000151892 | 4.31e-19 | 2.23e-17 |
| ENSG00000151893 | 3.81e-02 | 6.56e-02 |
| ENSG00000151914 | 2.10e-08 | 1.33e-07 |
| ENSG00000151917 | 2.08e-05 | 7.77e-05 |
| ENSG00000151923 | 2.11e-03 | 5.15e-03 |
| ENSG00000151929 | 1.47e-01 | 2.09e-01 |
| ENSG00000151948 | 8.44e-01 | 8.82e-01 |
| ENSG00000151952 | 6.46e-13 | 9.02e-12 |
| ENSG00000151962 | 5.65e-02 | 9.21e-02 |
| ENSG00000151963 | 2.71e-01 | 3.49e-01 |
| ENSG00000151967 | 5.33e-03 | 1.17e-02 |
| ENSG00000152034 | 3.44e-11 | 3.49e-10 |
| ENSG00000152049 | 1.52e-02 | 2.94e-02 |
| ENSG00000152056 | 1.46e-14 | 2.79e-13 |
| ENSG00000152061 | 3.07e-02 | 5.42e-02 |
| ENSG00000152076 | 2.05e-02 | 3.82e-02 |
| ENSG00000152078 | 2.06e-12 | 2.63e-11 |
| ENSG00000152082 | 5.59e-01 | 6.36e-01 |
| ENSG00000152086 | 5.68e-01 | 6.45e-01 |
| ENSG00000152092 | 1.16e-22 | 1.69e-20 |
| ENSG00000152093 | 4.48e-02 | 7.53e-02 |
| ENSG00000152102 | 1.34e-02 | 2.64e-02 |
| ENSG00000152104 | 1.10e-02 | 2.21e-02 |
| ENSG00000152117 | 8.41e-14 | 1.38e-12 |
| ENSG00000152127 | 2.82e-11 | 2.91e-10 |
| ENSG00000152128 | 5.08e-02 | 8.40e-02 |
| ENSG00000152133 | 1.40e-02 | 2.74e-02 |
| ENSG00000152137 | 4.43e-10 | 3.71e-09 |
| ENSG00000152147 | 2.27e-07 | 1.20e-06 |
| ENSG00000152154 | 7.48e-01 | 8.03e-01 |
| ENSG00000152192 | 4.91e-06 | 2.05e-05 |
| ENSG00000152193 | 1.08e-13 | 1.74e-12 |
| ENSG00000152207 | 9.48e-09 | 6.34e-08 |
| ENSG00000152208 | 1.44e-05 | 5.52e-05 |
| ENSG00000152213 | 9.06e-01 | 9.30e-01 |
| ENSG00000152214 | 7.01e-12 | 8.06e-11 |
| ENSG00000152217 | 4.31e-17 | 1.39e-15 |
| ENSG00000152219 | 2.47e-02 | 4.49e-02 |
| ENSG00000152223 | 1.46e-02 | 2.83e-02 |
| ENSG00000152229 | 2.93e-06 | 1.27e-05 |
| ENSG00000152234 | 4.46e-16 | 1.16e-14 |
| ENSG00000152240 | 4.42e-02 | 7.45e-02 |
| ENSG00000152242 | 7.36e-04 | 2.00e-03 |
| ENSG00000152253 | 6.45e-14 | 1.08e-12 |
| ENSG00000152254 | 3.75e-04 | 1.09e-03 |
| ENSG00000152256 | 2.04e-02 | 3.80e-02 |
| ENSG00000152266 | 4.80e-02 | 7.99e-02 |
| ENSG00000152270 | 1.37e-05 | 5.30e-05 |
| ENSG00000152284 | 1.92e-11 | 2.03e-10 |
| ENSG00000152291 | 2.09e-02 | 3.88e-02 |
| ENSG00000152292 | 9.29e-18 | 3.46e-16 |
| ENSG00000152315 | 2.54e-02 | 4.60e-02 |
| ENSG00000152332 | 1.47e-01 | 2.09e-01 |
| ENSG00000152348 | 7.93e-01 | 8.40e-01 |
| ENSG00000152359 | 3.54e-11 | 3.59e-10 |
| ENSG00000152377 | 1.21e-02 | 2.40e-02 |
| ENSG00000152380 | 3.16e-04 | 9.29e-04 |
| ENSG00000152382 | 2.82e-07 | 1.47e-06 |
| ENSG00000152402 | 4.17e-01 | 5.00e-01 |
| ENSG00000152404 | 4.31e-02 | 7.29e-02 |
| ENSG00000152409 | 2.04e-01 | 2.75e-01 |
| ENSG00000152413 | 8.31e-18 | 3.12e-16 |
| ENSG00000152422 | 1.25e-01 | 1.82e-01 |
| ENSG00000152430 | 7.36e-02 | 1.16e-01 |
| ENSG00000152433 | 2.98e-01 | 3.78e-01 |
| ENSG00000152439 | 1.27e-03 | 3.28e-03 |
| ENSG00000152443 | 2.41e-02 | 4.40e-02 |
| ENSG00000152454 | 1.46e-06 | 6.69e-06 |
| ENSG00000152455 | 6.30e-18 | 2.42e-16 |
| ENSG00000152457 | 6.74e-04 | 1.85e-03 |
| ENSG00000152463 | 1.00e-03 | 2.65e-03 |
| ENSG00000152464 | 3.23e-04 | 9.50e-04 |
| ENSG00000152465 | 4.25e-11 | 4.25e-10 |
| ENSG00000152467 | 5.92e-04 | 1.65e-03 |
| ENSG00000152475 | 9.10e-02 | 1.39e-01 |
| ENSG00000152484 | 1.12e-01 | 1.66e-01 |
| ENSG00000152492 | 1.21e-04 | 3.87e-04 |
| ENSG00000152495 | 4.98e-14 | 8.54e-13 |
| ENSG00000152503 | 8.44e-11 | 8.02e-10 |
| ENSG00000152518 | 8.78e-01 | 9.09e-01 |
| ENSG00000152520 | 3.95e-11 | 3.97e-10 |
| ENSG00000152527 | 7.37e-09 | 5.03e-08 |
| ENSG00000152556 | 1.20e-07 | 6.70e-07 |
| ENSG00000152558 | 1.07e-07 | 5.98e-07 |
| ENSG00000152578 | 2.57e-17 | 8.67e-16 |
| ENSG00000152580 | 1.24e-14 | 2.39e-13 |
| ENSG00000152582 | 7.97e-02 | 1.24e-01 |
| ENSG00000152583 | 1.45e-13 | 2.28e-12 |
| ENSG00000152591 | 2.65e-02 | 4.78e-02 |
| ENSG00000152592 | 8.30e-08 | 4.74e-07 |
| ENSG00000152595 | 3.23e-04 | 9.48e-04 |
| ENSG00000152601 | 1.92e-10 | 1.71e-09 |
| ENSG00000152611 | 7.79e-02 | 1.21e-01 |
| ENSG00000152620 | 9.48e-02 | 1.44e-01 |
| ENSG00000152642 | 1.57e-21 | 1.69e-19 |
| ENSG00000152661 | 4.78e-01 | 5.61e-01 |
| ENSG00000152669 | 9.01e-20 | 5.55e-18 |
| ENSG00000152670 | 2.78e-01 | 3.57e-01 |
| ENSG00000152672 | 7.29e-09 | 4.98e-08 |
| ENSG00000152683 | 2.95e-04 | 8.72e-04 |
| ENSG00000152684 | 6.44e-13 | 8.99e-12 |
| ENSG00000152689 | 2.55e-02 | 4.62e-02 |
| ENSG00000152700 | 1.23e-08 | 8.05e-08 |
| ENSG00000152705 | 4.22e-03 | 9.55e-03 |
| ENSG00000152749 | 7.78e-16 | 1.90e-14 |
| ENSG00000152760 | 6.84e-02 | 1.09e-01 |
| ENSG00000152763 | 1.70e-18 | 7.52e-17 |
| ENSG00000152766 | 2.73e-06 | 1.19e-05 |
| ENSG00000152767 | 1.71e-09 | 1.29e-08 |
| ENSG00000152778 | 3.71e-01 | 4.53e-01 |
| ENSG00000152779 | 1.06e-14 | 2.07e-13 |
| ENSG00000152782 | 1.07e-02 | 2.17e-02 |
| ENSG00000152784 | 3.04e-03 | 7.14e-03 |
| ENSG00000152785 | 3.25e-23 | 5.72e-21 |
| ENSG00000152795 | 2.80e-08 | 1.74e-07 |
| ENSG00000152804 | 5.34e-09 | 3.73e-08 |
| ENSG00000152818 | 3.19e-03 | 7.46e-03 |
| ENSG00000152822 | 9.75e-01 | 9.81e-01 |
| ENSG00000152894 | 5.45e-01 | 6.23e-01 |
| ENSG00000152904 | 5.20e-03 | 1.15e-02 |
| ENSG00000152910 | 4.19e-06 | 1.77e-05 |
| ENSG00000152926 | 4.72e-05 | 1.64e-04 |
| ENSG00000152931 | 9.60e-01 | 9.70e-01 |
| ENSG00000152932 | 2.58e-12 | 3.24e-11 |
| ENSG00000152936 | 1.55e-03 | 3.92e-03 |
| ENSG00000152939 | 4.44e-01 | 5.27e-01 |
| ENSG00000152942 | 2.34e-02 | 4.29e-02 |
| ENSG00000152944 | 7.92e-02 | 1.23e-01 |
| ENSG00000152952 | 3.99e-07 | 2.02e-06 |
| ENSG00000152953 | 7.85e-08 | 4.50e-07 |
| ENSG00000152954 | 2.01e-12 | 2.57e-11 |
| ENSG00000152969 | 2.41e-03 | 5.80e-03 |
| ENSG00000152977 | 7.24e-11 | 6.95e-10 |
| ENSG00000152990 | 1.29e-11 | 1.40e-10 |
| ENSG00000153002 | 1.96e-09 | 1.47e-08 |
| ENSG00000153006 | 5.78e-01 | 6.53e-01 |
| ENSG00000153012 | 6.86e-02 | 1.09e-01 |
| ENSG00000153015 | 4.28e-01 | 5.11e-01 |
| ENSG00000153029 | 8.16e-02 | 1.26e-01 |
| ENSG00000153037 | 7.23e-01 | 7.81e-01 |
| ENSG00000153044 | 1.36e-16 | 3.94e-15 |
| ENSG00000153046 | 4.81e-01 | 5.63e-01 |
| ENSG00000153048 | 4.14e-09 | 2.95e-08 |
| ENSG00000153060 | 7.56e-01 | 8.09e-01 |
| ENSG00000153064 | 1.29e-06 | 5.97e-06 |
| ENSG00000153066 | 1.79e-11 | 1.91e-10 |
| ENSG00000153071 | 4.57e-01 | 5.40e-01 |
| ENSG00000153086 | 2.10e-01 | 2.81e-01 |
| ENSG00000153093 | 5.99e-04 | 1.67e-03 |
| ENSG00000153094 | 5.93e-10 | 4.86e-09 |
| ENSG00000153107 | 3.43e-09 | 2.47e-08 |
| ENSG00000153113 | 1.61e-08 | 1.04e-07 |
| ENSG00000153130 | 7.53e-03 | 1.59e-02 |
| ENSG00000153132 | 5.23e-09 | 3.66e-08 |
| ENSG00000153140 | 4.71e-01 | 5.54e-01 |
| ENSG00000153147 | 1.92e-01 | 2.62e-01 |
| ENSG00000153157 | 1.06e-02 | 2.16e-02 |
| ENSG00000153162 | 1.65e-18 | 7.30e-17 |
| ENSG00000153165 | 9.91e-01 | 9.93e-01 |
| ENSG00000153179 | 5.00e-05 | 1.73e-04 |
| ENSG00000153187 | 1.39e-08 | 9.06e-08 |
| ENSG00000153201 | 2.17e-02 | 4.02e-02 |
| ENSG00000153207 | 1.03e-03 | 2.71e-03 |
| ENSG00000153208 | 1.75e-02 | 3.32e-02 |
| ENSG00000153214 | 8.33e-04 | 2.24e-03 |
| ENSG00000153230 | 1.40e-01 | 2.00e-01 |
| ENSG00000153233 | 2.80e-13 | 4.20e-12 |
| ENSG00000153234 | 2.35e-02 | 4.30e-02 |
| ENSG00000153237 | 3.98e-10 | 3.37e-09 |
| ENSG00000153246 | 1.36e-01 | 1.95e-01 |
| ENSG00000153250 | 4.89e-01 | 5.71e-01 |
| ENSG00000153253 | 4.18e-15 | 8.82e-14 |
| ENSG00000153266 | 1.36e-04 | 4.32e-04 |
| ENSG00000153283 | 6.23e-12 | 7.23e-11 |
| ENSG00000153291 | 2.49e-08 | 1.56e-07 |
| ENSG00000153292 | 1.91e-03 | 4.72e-03 |
| ENSG00000153294 | 6.57e-01 | 7.24e-01 |
| ENSG00000153303 | 1.81e-08 | 1.16e-07 |
| ENSG00000153310 | 3.19e-06 | 1.37e-05 |
| ENSG00000153317 | 7.71e-09 | 5.24e-08 |
| ENSG00000153339 | 2.02e-09 | 1.51e-08 |
| ENSG00000153347 | 1.60e-01 | 2.24e-01 |
| ENSG00000153363 | 1.05e-03 | 2.75e-03 |
| ENSG00000153391 | 3.11e-05 | 1.12e-04 |
| ENSG00000153395 | 9.62e-13 | 1.30e-11 |
| ENSG00000153404 | 7.55e-05 | 2.52e-04 |
| ENSG00000153406 | 1.86e-08 | 1.19e-07 |
| ENSG00000153443 | 1.34e-09 | 1.04e-08 |
| ENSG00000153446 | 3.84e-21 | 3.54e-19 |
| ENSG00000153485 | 5.24e-08 | 3.11e-07 |
| ENSG00000153487 | 1.45e-11 | 1.57e-10 |
| ENSG00000153495 | 1.13e-03 | 2.95e-03 |
| ENSG00000153531 | 4.07e-03 | 9.24e-03 |
| ENSG00000153551 | 2.99e-13 | 4.45e-12 |
| ENSG00000153558 | 3.45e-03 | 7.99e-03 |
| ENSG00000153560 | 9.26e-01 | 9.45e-01 |
| ENSG00000153561 | 4.75e-06 | 1.99e-05 |
| ENSG00000153563 | 3.84e-09 | 2.75e-08 |
| ENSG00000153574 | 2.20e-14 | 4.06e-13 |
| ENSG00000153684 | 1.78e-03 | 4.43e-03 |
| ENSG00000153707 | 1.31e-02 | 2.57e-02 |
| ENSG00000153714 | 4.31e-03 | 9.72e-03 |
| ENSG00000153721 | 1.33e-05 | 5.14e-05 |
| ENSG00000153767 | 6.84e-06 | 2.78e-05 |
| ENSG00000153774 | 1.25e-05 | 4.86e-05 |
| ENSG00000153786 | 2.23e-14 | 4.11e-13 |
| ENSG00000153789 | 1.40e-02 | 2.73e-02 |
[truncated: 1,359,296 more chars]
