# Supplementary material for: OMICfpp: a fuzzy approach for paired RNA-Seq counts
Source: BMC Genomics. 2019 Apr 2;20:259. doi: 10.1186/s12864-019-5496-5 (PMC6444640; doi:10.1186/s12864-019-5496-5)

Supplementary Material

January 3, 2019

1 Download and preprocessing of the data

1.1 TCGA dataset

The RNA-seq (HTSeq - Counts) data of paired samples (solid tissue normal and primary tumor) from 50 patients with colorectal cancer were obtained from TCGA dataset. The dataset was downloaded from the Genomic Data Commons Data Portal (GDC: https://portal.gdc.cancer.gov/) using the following shell code:

```
./gdc-client download -m CRC_pairs_manifest.txt
```

The clinical data were downloaded too. The data were included in R:

```
setwd("../TCGA_CRC")
indir = getwd()
files = list.files(indir, pattern='\\counts.gz')
Metadata = read.csv(file= "ALL_data_TCGA_colonyrectum.csv",
                    header = TRUE, sep=",")
TCGA_CRC_Data = readDGE(files, path=indir, columns=c(1,2),
                        group=Metadata$Sample_Type)
save(TCGA_CRC, file = "TCGA_CRC.rda")
```

1.2 PRJNA218851 dataset

In addition, the raw RNA-seq data of paired samples (normal colon and primary tumor) from 18 patients with colorectal cancer were added, this were obtained from the PRJNA218851 BioProject. The dataset was downloaded in FASTQ format from the Sequence Read Archive (SRA: https://www.ncbi.nlm.nih.gov/sra) using the SRA toolkit following shell code:

```
fastq-dump --readids --split-files SRA_sample_code
```

The quality of the PRJNA218851 raw dataset was checked using the FASTQC tool and the first 10 low-quality bases, reads shorter than 30 nucleotides and global low quality reads were discarded using fastx-toolkit:

```
for i in *.fastq; do fastqc "$i";done &
for i in *.fastq; do fastx_trimmer -i "$i" -o "$i".trim -
    ↪ f 11 -Q 33 -v; done &
for i in *.trim;do fastx_clipper -i "$i" -o "$i".clip -l
    ↪ 30 -v; done &
for i in *.clip;do fastq_quality_filter -i "$i" -o "$i".
    ↪ filtered -q 20 -p 80 -Q 33 -v; done &
```

Later, the reads were mapped with STAR, first the GRCh38 reference human genome was indexed:

```
./STAR --runMode genomeGenerate --runThreadN 8 --
    ↪ genomeDir ./ --genomeFastaFiles /home4/GRCh38.fa
```

Then, the files were mapped, generating one SAM file per sample, using the following script:

```
#!/bin/bash
  A="_1.fastq"
  B="_2.fastq"
  for line in $(cat list.txt);
  do
  echo "$line"
  ./STAR --genomeDir ./ --runThreadN 8 starIndex --
      ↪ readFilesIn /home4/Data_SRA/$line$A /home4/
      ↪ Data_SRA/$line$B --outFileNamePrefix /home4/
      ↪ BAM_STAR/$line;
  done
```

After that, the ‘SAM‘ file were converted to sorted ‘BAM‘ file:

```
setwd("/home4/BAM_STAR/")
x = read.csv("files.txt",stringsAsFactors = FALSE)
foutput = "lanza_STAR"
file.create(foutput)
dir_aligned = "/home4/BAM_STAR/"
for(i in 1:nrow(x)){
    file_sam = paste0(dir_aligned,x[i,1])
    file_bam = paste0(dir_aligned,x[i,1],".bam")
    chunk3 = paste("samtools view -bS ",file_sam,
                   " | samtools sort - ",file_bam,"\n")
    cat(chunk3,file=foutput,append = TRUE)
}
```

Finally, the count matrix was generated.

```
library(Rsamtools)
library(GenomicFeatures)
library(GenomicAlignments)
gtfFile = "/home4/Data_SRA/GRCh38.gtf"
txdb = makeTxDbFromGFF(gtfFile, format="gtf")
genes = exonsBy(txdb, by="gene")
dirActualData =  paste(getwd(),"/",sep="")
sampleTable = read.table("file.txt")
fls = paste(dirActualData,sampleTable[,1],sep="")
bamLst = BamFileList(fls, index=character(),
                     yieldSize=100000,obeyQname=TRUE)
PRJNA218851_CRC =
    summarizeOverlaps(features = genes,read=bamLst,
                      mode="Union",
                      singleEnd=FALSE,
                      ignore.strand=TRUE,
                      fragments=FALSE)
Metadata = read.csv(file= "PRJNA218851_CRC.csv",
```

```
                    header = TRUE, sep=",")
SampleName = Metadata$name_file
Stage = Metadata$Stage
colData(PRJNA218851_CRC) = DataFrame(SampleName, Stage)
save(PRJNA218851_CRC,file="PRJNA218851_CRC.rda")
```

1.3 Creating the SummarizedExperiment

In order to manage the count matrix and the metadata jointly it is convenient to use a `SummarizedExperiment`.

```
pacman::p_load(SummarizedExperiment,edgeR)
load("PRJNA218851_CRC.rda")
dim(PRJNA218851_CRC)
colData(PRJNA218851_CRC)[,"Stage"]
x1 = PRJNA218851_CRC[,1:36]
dim(x1)
colnames_x1 = colnames(assay(x1))
colData(x1)[,"Stage"] = factor(colData(x1)[,"Stage"])
levels(colData(x1)[,"Stage"]) = c("case","control")
colData(x1) = DataFrame(tissue = colData(x1)[,"Stage"],
                         pair = c(51:68,51:68))
levels(colData(x1)[,"tissue"])
load("TCGA_CRC.rda")
dim(TCGA_CRC)
meta = read.csv("metadata.csv",header=TRUE,sep=";")
levels(meta$Group) = c("control","case")
match(TCGA_CRC$samples$files,meta[,"File_name"])
x2 = SummarizedExperiment(assays=TCGA_CRC$counts,
                           rowData=rownames(TCGA_CRC$counts))
colData(x2) = DataFrame(tissue=meta$Group,pair = meta$pair)
x2 = x2[1:60482]
dim(x2)
rowData(x2) = unlist(lapply(rowData(x2)$X,function(x0)
    unlist(strsplit(x0, "[.]"))[[1]]))

cc = match(rowData(x1)[,"ENSEMBL"],rowData(x2)[,"X"])
x11 = x1[!is.na(cc),]
cc = match(rowData(x2)[,"X"],rowData(x1)[,"ENSEMBL"])
x22 = x2[!is.na(cc),]

cc = match(rowData(x11)[,"ENSEMBL"],rowData(x22)[,"X"])
x3 = cbind(assay(x11[cc,]),assay(x22))
colnames(x3) = c(colnames_x1,as.character(meta$name_sample))

se = SummarizedExperiment(assays=x3,
                          rowData=rownames(x3))
colData(se) = rbind(colData(x1),colData(x2))
temp = rep(0,nrow(colData(se)))
temp[which(colData(se)[,"tissue"] == "case")] = 1
colData(se) = DataFrame(pair = colData(se)[,"pair"],tissue = temp)
save(se,file="se.rda")
```

2 Generating data

First, we load the packages.

```
pacman::p_load(SummarizedExperiment,org.Hs.eg.db,tami,ggplot2,latex2exp,
               OMICfpp,ReportingTools,DESeq2,pROC,gridExtra)
```

Second, we load the data.

```
load("../data/se.rda")
```

The just loaded `RangedSummarizedExperiment` `se` will contain all the needed data. We filter genes with low counts.

```
df = matrixStats::rowSums2(assay(se))
se = se[df>=20,]
```

3 Differential expression analysis

This section contains two different differential expression analysis using the R packages `edgeR` and `DESeq2` methods.

3.1 edgeR

We have paired data. We will begin with an analysis using **edgeR** by taking into account the paired design. It has been implemented in the OMICfpp package in the function OMICfpp::edgeRpaired.

```
OMICfpp::edgeRpaired

## function (x, y)
## {
##     subject = factor(y[, 1])
##     treatment = factor(y[, 2])
##     x1 = edgeR::DGEList(x)
##     design = model.matrix(~subject + treatment)
##     x1 = edgeR::estimateDisp(x1, design)
##     fit = edgeR::glmQLFit(x1, design)
##     qlf = edgeR::glmQLFTest(fit)
##     qlf1 = edgeR::topTags(qlf, sort.by = "none", n = nrow(x1))
##     qlf1$table[, "PValue"]
## }
## <bytecode: 0x55bdba7cf578>
## <environment: namespace:OMICfpp>
```

The p-values can be obtained with the following code.

```
p0.er = OMICfpp::edgeRpaired(x=assay(se),y=colData(se))
names(p0.er) = rownames(se)
save(p0.er,file="p0.er.rda")
```

The html report for the edgeR analysis.

```r
load("p0.er.rda")
p0.adj.er = p.adjust(p0.er,method="BH")
df = data.frame(gene=tami::ensembl2url(rownames(se)),edgeR = p0.er,
                edgeR_adj = p0.adj.er)
foutput = "edgeR"
htmlRep1 = HTMLReport(shortName = foutput,title = foutput,
reportDirectory = "./reports")
publish(df,htmlRep1)
finish(htmlRep1)

## [1] "./reports/edgeR.html"
```

3.2 DESeq2

Now, we use the package **DESeq2** by taking into account the paired design. It has been implemented in the OMICfpp package in the function OMICfpp::edgeRpaired.

```r
x=assay(se);y=colData(se)
y1 = S4Vectors::DataFrame(subject = factor(y[, 1]), treatment = factor(y[,2]))
dds <- DESeq2::DESeqDataSetFromMatrix(countData = x, colData = y1,
                                      design = ~subject + treatment)
dds = DESeq2::DESeq(dds)
##save(dds,file="dds.rda")
load("dds.rda")
resLFC69 = DESeq2::lfcShrink(dds, coef = 69)
##save(resLFC69,file="resLFC69.rda")
```

```r
load("resLFC69.rda")
p0.de = resLFC69[,"pvalue"]
save(p0.de,file="p0.de.rda")
```

```r
load("p0.er.rda")
load("p0.de.rda")
df0 = data.frame(er = p0.er, de = p0.de)
df1 = reshape2::melt(df0,id="er")
ggplot(df1,aes(x=er,y=value,color=variable)) + geom_smooth()

## `geom_smooth()` using method = 'gam' and formula 'y ~ s(x,
bs = "cs")'
```

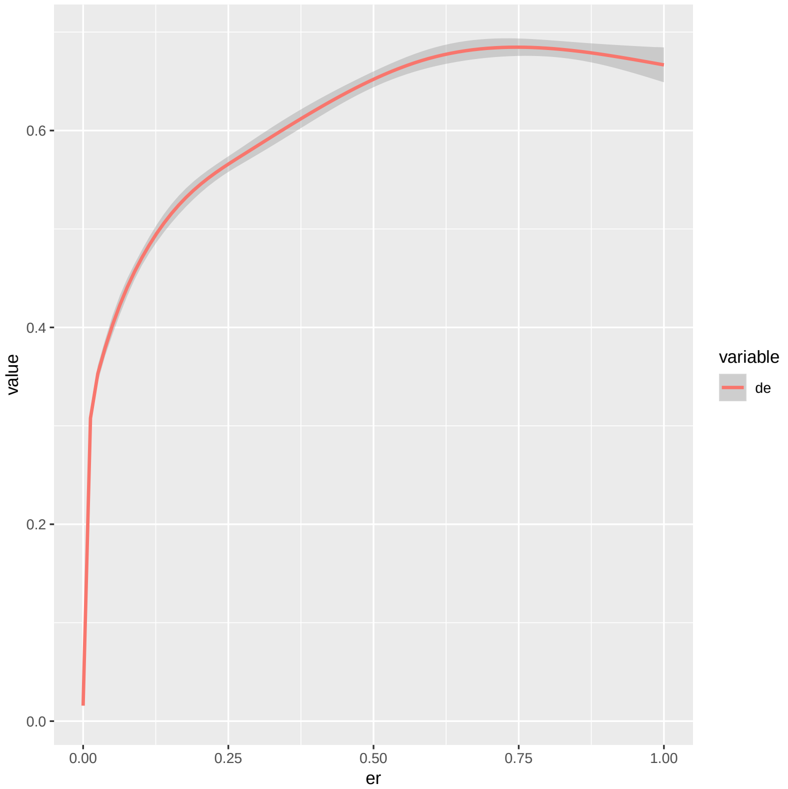

The html report for the DESeq2 analysis.

```
load("p0.de.rda")
p0.adj.de = p.adjust(p0.de,method="BH")
df = data.frame(gene=tami::ensembl2url(rownames(se)),DESeq2 = p0.de,
                DESeq2_adj = p0.adj.de)
foutput = "DESeq2"
htmlRep1 = HTMLReport(shortName = foutput,title = foutput,
reportDirectory = "./reports")
publish(df,htmlRep1)
finish(htmlRep1)

## [1] "./reports/DESeq2.html"
```

4 Marginal differential expression analysis

First, we generate and save the original matrix of p-values.

```
p0 = proportion.p(x=assay(se),y=colData(se))
save(p0,file="p0.rda")
```

Set the orness values.

```
norness0 = 50
orness0 = seq(.01,.99,length.out=norness0)
```

4.1 Test for proportion with 100 simulations

We use p-values corresponding to the test for the proportion using the **between-pair** randomization distribution.

```
dirData = "~/puentegenil17-tmp/"
nsim0 = 100
test0 = proportion.p
method0 = "dbinom"
type0 = "between-pair"
foutput0 = paste("proportion.p",norness0,nsim0,
                 method0,type0,sep="_")
foutput0 = paste0(dirData,foutput0,".rda")
Sys.time()
p1.b = OMICfpp::marginal.analysis(df=assay(se),y=colData(se),
                  test=test0,orness= orness0,nsim = nsim0,
                  method=method0,type=type0,is.decreasing=FALSE,
                  to.save=TRUE,
                  foutput=foutput0)
Sys.time()
```

We use p-values corresponding to the proportion test using the **complete** randomization distribution.

```
dirData = "~/Nextcloud/puentegenil17-b/"
test0 = proportion.p
method0 = "dbinom"
type0 = "complete"
foutput0 = paste("proportion.p",norness0,nsim0,
                 method0,type0,sep="_")
foutput0 = paste0(dirData,foutput0,".rda")
Sys.time()
p1.c = OMICfpp::marginal.analysis(df=assay(se),y=colData(se),test=test0,
                           orness= orness0,nsim = nsim0,method=method0,
                           type=type0,is.decreasing=FALSE,
                           to.save=TRUE,
                           foutput=foutput0)
Sys.time()
```

Evaluating randomization p-values.

```
dirData = "~/Nextcloud/puentegenil17-b/"
foutput0 = "proportion.p_50_100_dbinom_between-pair.rda"
load(paste0(dirData,foutput0))
p1 = sapply(1:norness0,
            function(j){df0 = df1[,(0:(nsim0-1))*50 + j]
                gen = df0[,1]
                ecdf(gen[-1])(gen[1])
                apply(df0,1,function(x) ecdf(x[-1])(x[1]))
            })
foutput1 = "proportion.p_50_100_dbinom_between-pair_p1.rda"
save(p1,file = foutput1)

foutput0 = "proportion.p_50_100_dbinom_complete.rda"
load(paste0(dirData,foutput0))
```

```
p1 = sapply(1:norness0,
            function(j){df0 = df1[,(0:(nsim0-1))*50 + j]
                gen = df0[,1]
                ecdf(gen[-1])(gen[1])
                apply(df0,1,function(x) ecdf(x[-1])(x[1]))
            })
foutput1 = "proportion.p_50_100_dbinom_complete_p1.rda"
save(p1,file = foutput1)
```

4.2 Test for proportion with 1000 simulations

The following code estimate the randomization p-values with 1000 simulations. First the between-pair distribution.

```
x0 = assay(se)
y0 = colData(se)
nsim0 = 1000
test0 = proportion.p
method0 = "dbinom"
type0 = "between-pair"
foutput0 = paste("proportion.p",norness0,nsim0,method0,type0,sep="_")
Sys.time()
cat("Permutation 0 \n")
pval0 = pairedCounts(x = x0, y = y0, test = test0, orness = orness0,
                     method = method0, is.decreasing = FALSE)
Sys.time()
pval1 = matrix(0, nrow = nrow(pval0), ncol = ncol(pval0))
for (j in 1:nsim0) {
    cat("Permutation ", j, "\n")
    pval1.temp = pairedCounts(x = x0, y = null.paired(y0, type0),
                              test = test0, orness = orness0, method = method0,
                              is.decreasing = FALSE)
    pval1 = pval1 + (pval1.temp < pval0) * 1
    save(pval1,file="pval1_b.rda")
    Sys.time()
}
p1.b = pval1/nsim0
save(p1.b,file=paste0(foutput0,"_p1.rda"))
Sys.time()
```

Second, the complete distribution.

```
x0 = assay(se)
y0 = colData(se)
nsim0 = 1000
test0 = proportion.p
method0 = "dbinom"
type0 = "complete"
foutput0 = paste("proportion.p",norness0,nsim0,method0,type0,sep="_")
Sys.time()
cat("Permutation 0 \n")
pval0 = pairedCounts(x = x0, y = y0, test = test0, orness = orness0,
                     method = method0, is.decreasing = FALSE)
```

```
Sys.time()
pval1 = matrix(0, nrow = nrow(pval0), ncol = ncol(pval0))
for (j in 1:nsim0) {
    cat("Permutation ", j, "\n")
    pval1.temp = pairedCounts(x = x0, y = null.paired(y0, type0),
                             test = test0, orness = orness0, method = method0,
                             is.decreasing = FALSE)
    pval1 = pval1 + (pval1.temp < pval0) * 1
    save(pval1,file="pval1_b.rda")
    Sys.time()
}
p1.c = pval1/nsim0
save(p1.c,file=paste0(foutput0,"_p1.rda"))
Sys.time()
```

Now we repeat the analysis with a few samples, in fact, a random sample of 20 samples.

```
n0 = 20
##sel0 = sample(1:68,n0)
##  c(28,67,68,50,31,47,38, 9, 16, 20)
##  9 67 22 33 35 14 20 38 63 28 34 62 40 24 52 16 13 15 29 31
se0 = se[,is.element(colData(se)[,"pair"],sel0)]
x0 = assay(se0)
y0 = colData(se0)

nsim0 = 1000
test0 = proportion.p
method0 = "dbinom"
type0 = "complete"
foutput0 = paste("proportion.p",norness0,nsim0,method0,type0,n0,sep="_")
Sys.time()
cat("Permutation 0 \n")
pval0 = pairedCounts(x = x0, y = y0, test = test0, orness = orness0,
                     method = method0, is.decreasing = FALSE)
Sys.time()
pval1 = matrix(0, nrow = nrow(pval0), ncol = ncol(pval0))
for (j in 1:nsim0) {
    cat("Permutation ", j, "\n")
    pval1.temp = pairedCounts(x = x0, y = null.paired(y0, type0),
                             test = test0, orness = orness0, method = method0,
                             is.decreasing = FALSE)
    pval1 = pval1 + (pval1.temp < pval0) * 1
    save(pval1,file="pval1_b.rda")
    Sys.time()
}
p1.c = pval1/nsim0
save(p1.c,file=paste0(foutput0,"_p1.rda"))
Sys.time()
```

```
load("proportion.p_50_1000_dbinom_complete_p1.rda")

p1.c.68 = p1.c
```

```r
load("proportion.p_50_45_dbinom_complete_p1.rda")
p1.c.10 = p1.c
load("proportion.p_50_1000_dbinom_complete_20_p1.rda")
p1.c.20 = p1.c
```

```r
small.sample.n = function(alpha,p1.c.68,p1.c.20,p1.c.10){
    sig68 = p1.c.68 <alpha
    sig20 = p1.c.20 <alpha
    sig10 = p1.c.10 <alpha
    n68 = apply(sig68,2,sum)
    n68.n20= apply(sig68 * sig20,2,sum)
    n68.n10 = apply(sig68 * sig10,2,sum)
    n20.n10 = apply(sig20 * sig10,2,sum)
    df0 = data.frame(orness = orness0,n68.n20,n68.n10,n20.n10)
    df1 = reshape2::melt(df0,id="orness")
    ggplot(df1,aes(x=orness,y=value,color=variable)) +
        geom_line(aes(linetype=variable)) + ylab("Number of genes")
}

small.sample.p = function(alpha,p1.c.68,p1.c.20,p1.c.10){
    sig68 = p1.c.68 <alpha
    sig20 = p1.c.20 <alpha
    sig10 = p1.c.10 <alpha
    n68 = apply(sig68,2,sum)
    n68.n20= apply(sig68 * sig20,2,sum)/n68
    n68.n10 = apply(sig68 * sig10,2,sum)/n68
    n20.n10 = apply(sig20 * sig10,2,sum)/n68
    df0 = data.frame(orness = orness0,n68.n20,n68.n10,n20.n10)
    df1 = reshape2::melt(df0,id="orness")
    ggplot(df1,aes(x=orness,y=value,color=variable)) +
        geom_line(aes(linetype=variable)) + ylab("Number of genes")
}
```

Now, the plots comparing the performance using different numbers of samples.

```r
png("figures/small_samples_001_n.png")
small.sample.n(alpha=.001,p1.c.68=p1.c.68,p1.c.20=p1.c.20,
             p1.c.10=p1.c.10)
dev.off()

## pdf
##   2

small.sample.n(alpha=.001,p1.c.68=p1.c.68,p1.c.20=p1.c.20,
             p1.c.10=p1.c.10)
```

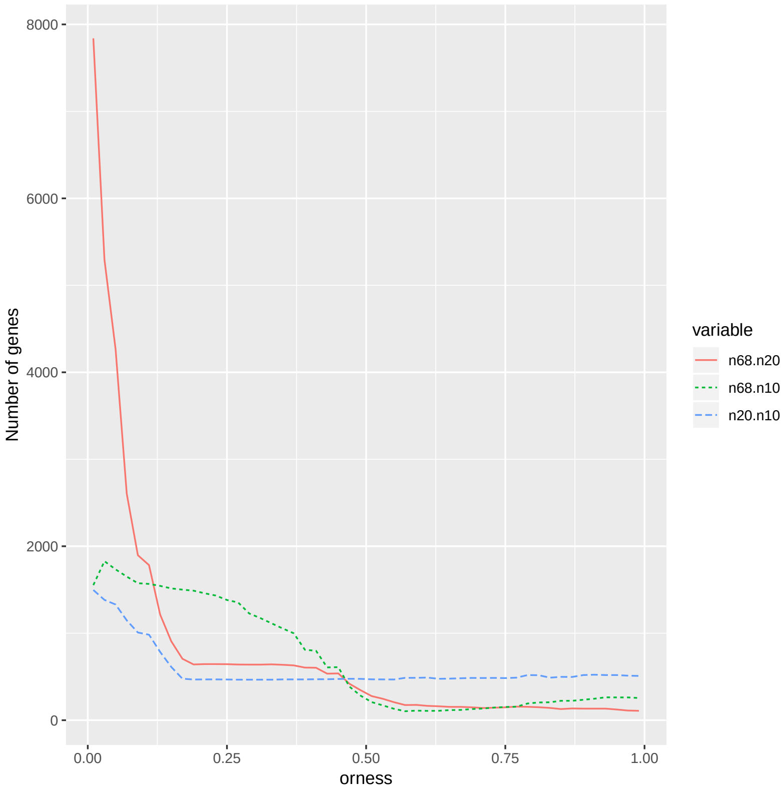


```
png("figures/small_samples_001_p.png")
small.sample.p(alpha=.001,p1.c.68=p1.c.68,p1.c.20=p1.c.20,
             p1.c.10=p1.c.10)
dev.off()

## pdf
##   2

small.sample.p(alpha=.001,p1.c.68=p1.c.68,p1.c.20=p1.c.20,
             p1.c.10=p1.c.10)
```

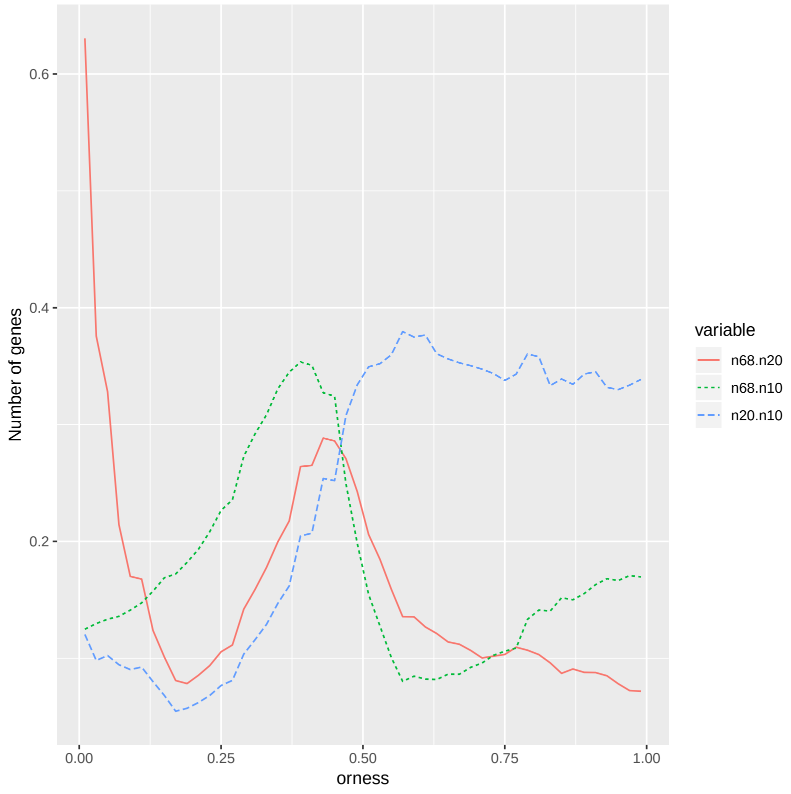


```
png("figures/small_samples_03_n.png")
small.sample.n(alpha=.03,p1.c.68=p1.c.68,p1.c.20=p1.c.20,
             p1.c.10=p1.c.10)
dev.off()

## pdf
##   2

small.sample.n(alpha=.03,p1.c.68=p1.c.68,p1.c.20=p1.c.20,
             p1.c.10=p1.c.10)
```

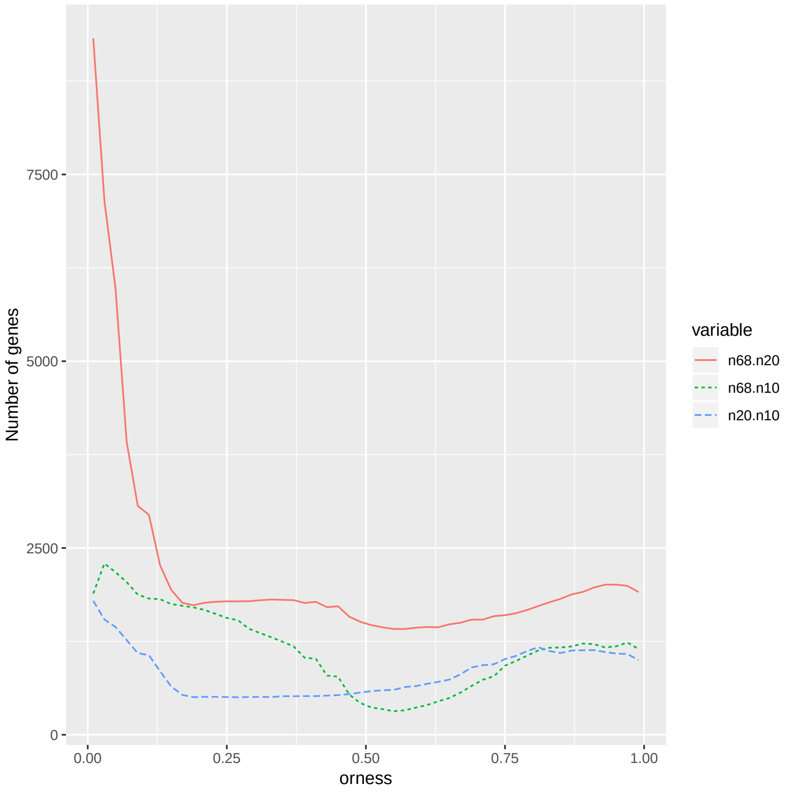


```
png("figures/small_samples_03_p.png")
small.sample.p(alpha=.03,p1.c.68=p1.c.68,p1.c.20=p1.c.20,
             p1.c.10=p1.c.10)
dev.off()

## pdf
##   2

small.sample.p(alpha=.03,p1.c.68=p1.c.68,p1.c.20=p1.c.20,
             p1.c.10=p1.c.10)
```

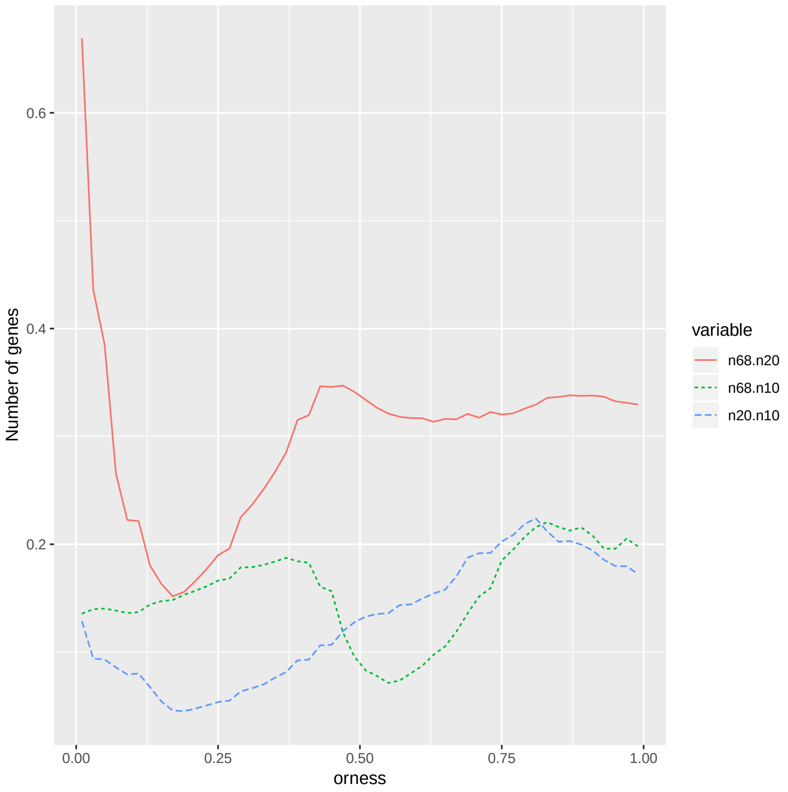

4.3 Analysis of selected genes

Loading the p-values.

```
load("p0.rda")
load("p0.er.rda")
load("p0.de.rda")
load("proportion.p_50_1000_dbinom_between-pair_p1.rda")
load("proportion.p_50_1000_dbinom_complete_p1.rda")
```

4.3.1 Selecting genes using the observed p-values

We choose the most significant gene using edgeR.

```
png("figures/edgeR_most_significant_evalOrness.png")
evalOrness(p0=p0,p.b=p1.b,p.c=p1.c,orness=orness0,row = which.min(p0.er))
dev.off()

## pdf
##   2

evalOrness(p0=p0,p.b=p1.b,p.c=p1.c,orness=orness0,row = which.min(p0.er))
```

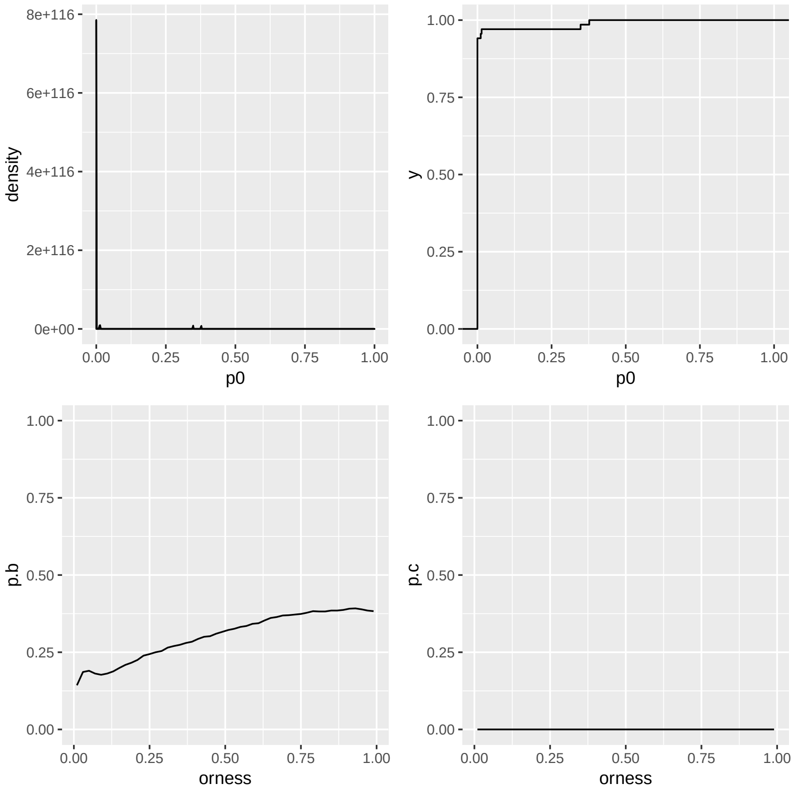

Now, the less significant gene using edgeR.

```
png("figures/edgeR_lowest_significant_evalOrness.png")
evalOrness(p0=p0,p.b=p1.b,p.c=p1.c,orness=orness0,row = which.max(p0.er))

## Warning: Removed 1 rows containing non-finite values (stat_density).
## Warning: Removed 1 rows containing non-finite values (stat_ecdf).

dev.off()

## pdf
##   2

evalOrness(p0=p0,p.b=p1.b,p.c=p1.c,orness=orness0,row = which.max(p0.er))

## Warning: Removed 1 rows containing non-finite values (stat_density).
## Warning: Removed 1 rows containing non-finite values (stat_ecdf).
```

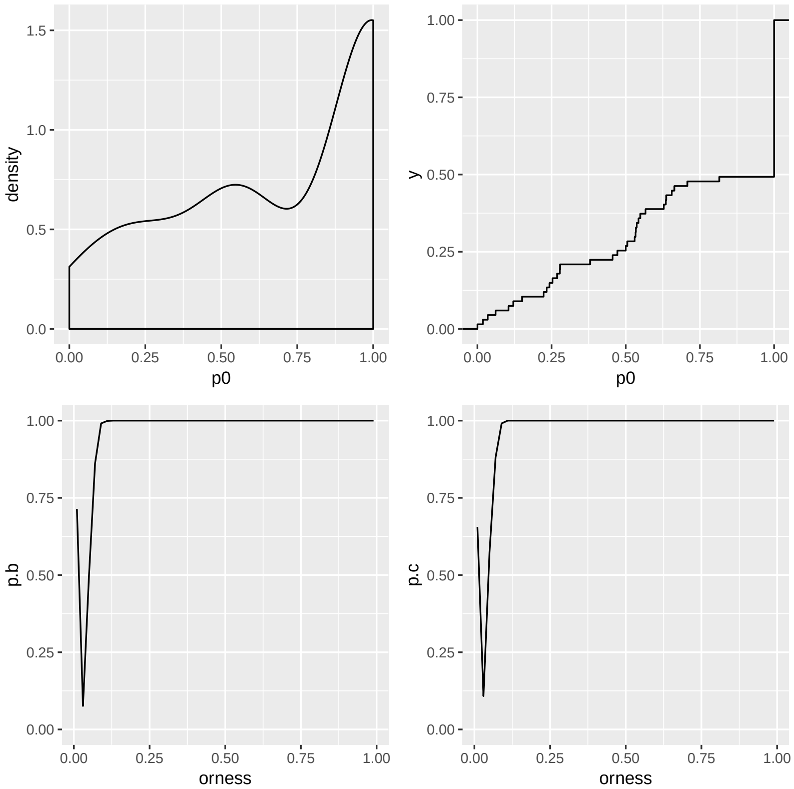

The genes can be ordered using the area under the empirical cumulative distribution function.

```
p0.area = apply(p0,1,function(x){
    ff = ecdf(x)
    integrate(ff,lower=0,upper=1,stop.on.error=FALSE)$value
})
rownames(p0.area) = rownames(p0)
save(p0.area,file="p0.area.rda")
```

```
load("p0.area.rda")
```

We can see the genes with the lesser area under the empirical cumulative distribution function.

```
png("figures/ecdf_lowest_1.png")
evalOrness(p0=p0,p.b=p1.b,p.c=p1.c,orness=orness0,
           row =sort(p0.area,index.return=TRUE)$ix[1])
dev.off()

## pdf
##   2

evalOrness(p0=p0,p.b=p1.b,p.c=p1.c,orness=orness0,
           row =sort(p0.area,index.return=TRUE)$ix[1])
```

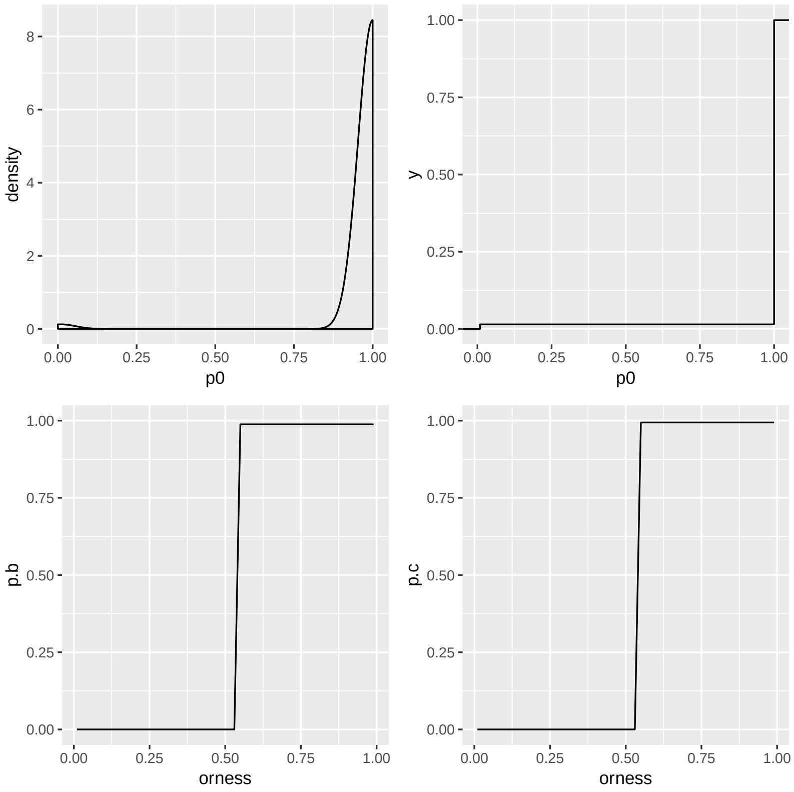


```
## The gene corresponds to the row 34254
```

```
png("figures/ecdf_lowest_10.png")
evalOrness(p0=p0,p.b=p1.b,p.c=p1.c,orness=orness0,
           row =sort(p0.area,index.return=TRUE)$ix[10])
dev.off()

## pdf
##   2

evalOrness(p0=p0,p.b=p1.b,p.c=p1.c,orness=orness0,
           row =sort(p0.area,index.return=TRUE)$ix[10])
```

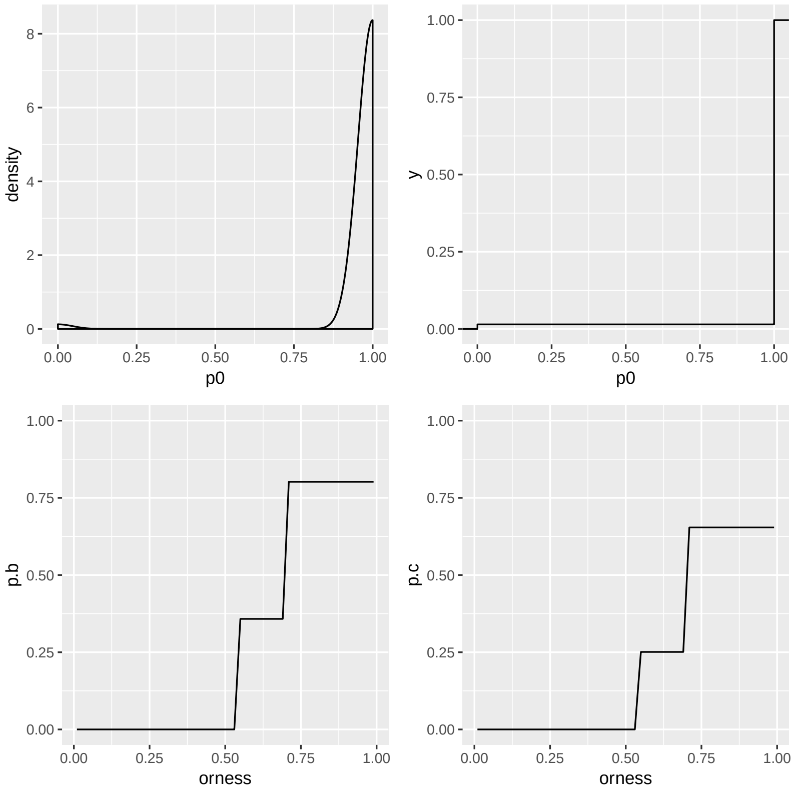


```
png("figures/ecdf_lowest_100.png")
evalOrness(p0=p0,p.b=p1.b,p.c=p1.c,orness=orness0,
           row =sort(p0.area,index.return=TRUE)$ix[100])
dev.off()

## pdf
##   2

evalOrness(p0=p0,p.b=p1.b,p.c=p1.c,orness=orness0,
           row =sort(p0.area,index.return=TRUE)$ix[100])
```

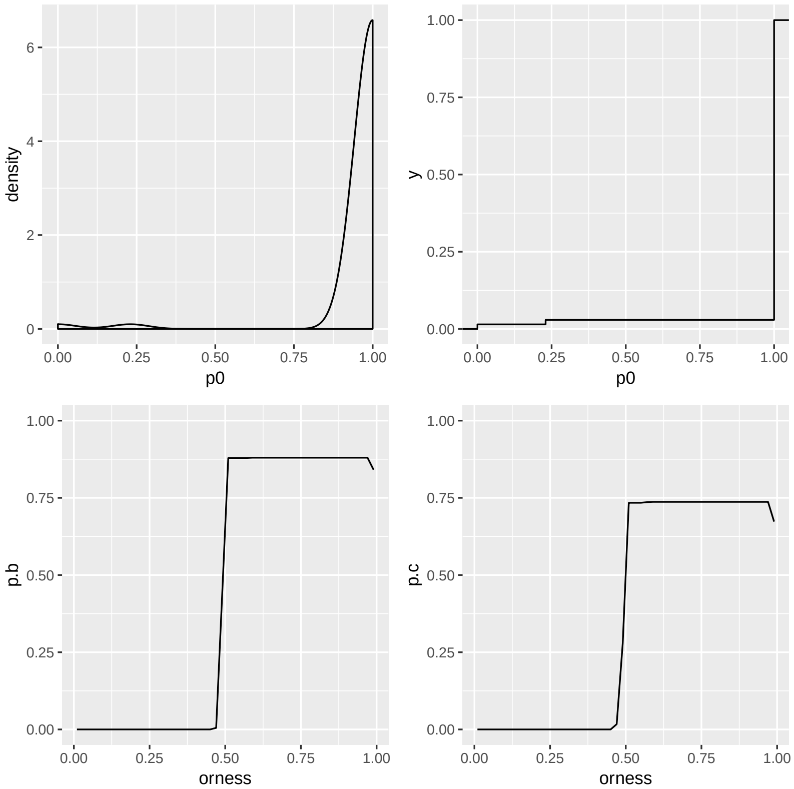

Now we can evaluate the genes with the highest area under the empirical cumulative distribution function.

```
png("figures/ecdf_highest_1.png")
evalOrness(p0=p0,p.b=p1.b,p.c=p1.c,orness=orness0,
           row =sort(p0.area,decreasing=TRUE,index.return=TRUE)$ix[1])
dev.off()

## pdf
##   2

evalOrness(p0=p0,p.b=p1.b,p.c=p1.c,orness=orness0,
           row =sort(p0.area,decreasing=TRUE,index.return=TRUE)$ix[1])
```

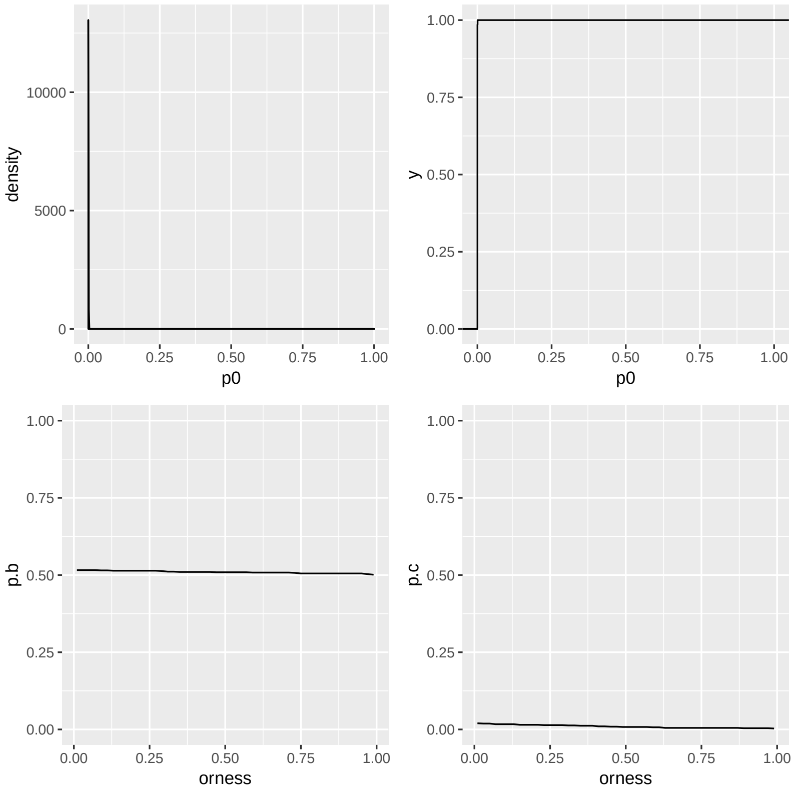


```
png("figures/ecdf_highest_10.png")
evalOrness(p0=p0,p.b=p1.b,p.c=p1.c,orness=orness0,
           row =sort(p0.area,decreasing=TRUE,index.return=TRUE)$ix[10])
dev.off()

## pdf
##   2

evalOrness(p0=p0,p.b=p1.b,p.c=p1.c,orness=orness0,
           row =sort(p0.area,decreasing=TRUE,index.return=TRUE)$ix[10])
```

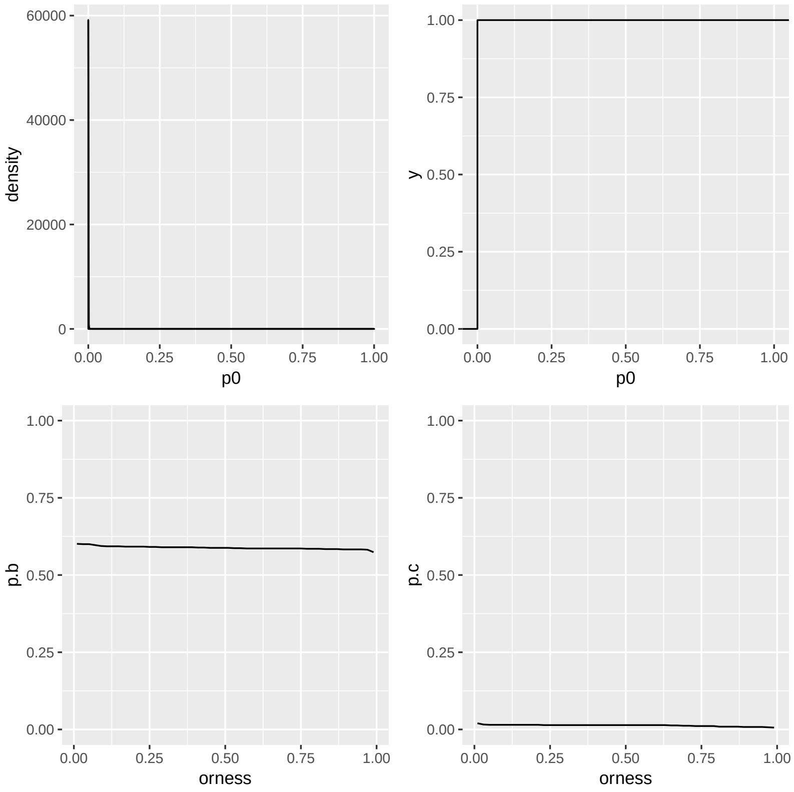


```
png("figures/ecdf_highest_100.png")
evalOrness(p0=p0,p.b=p1.b,p.c=p1.c,orness=orness0,
           row =sort(p0.area,decreasing=TRUE,index.return=TRUE)$ix[100])
dev.off()

## pdf
##   2

evalOrness(p0=p0,p.b=p1.b,p.c=p1.c,orness=orness0,
           row =sort(p0.area,decreasing=TRUE,index.return=TRUE)$ix[100])
```

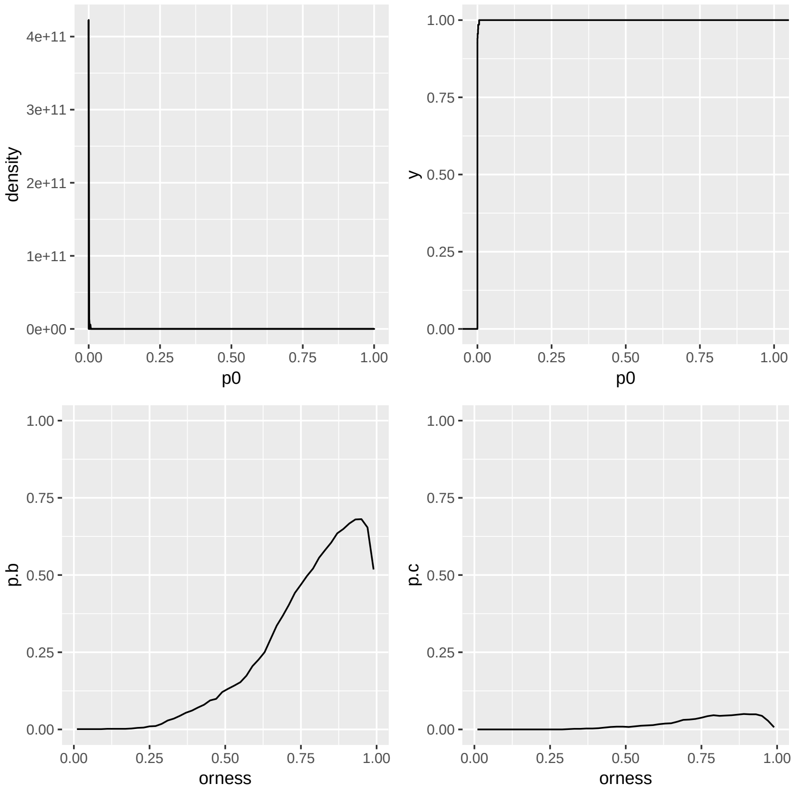

The following corresponds to the genes used in figure 1(b), 1(c) and 1(d) of the paper.

```
png("figures/ecdf_ENSG00000095752.png")
evalOrness(p0=p0,p.b=p1.b,p.c=p1.c,orness=orness0,
           row = which(rownames(p1.c) == "ENSG00000095752"))
dev.off()

## pdf
##   2

evalOrness(p0=p0,p.b=p1.b,p.c=p1.c,orness=orness0,
           row =which(rownames(p1.c) == "ENSG00000095752"))
```

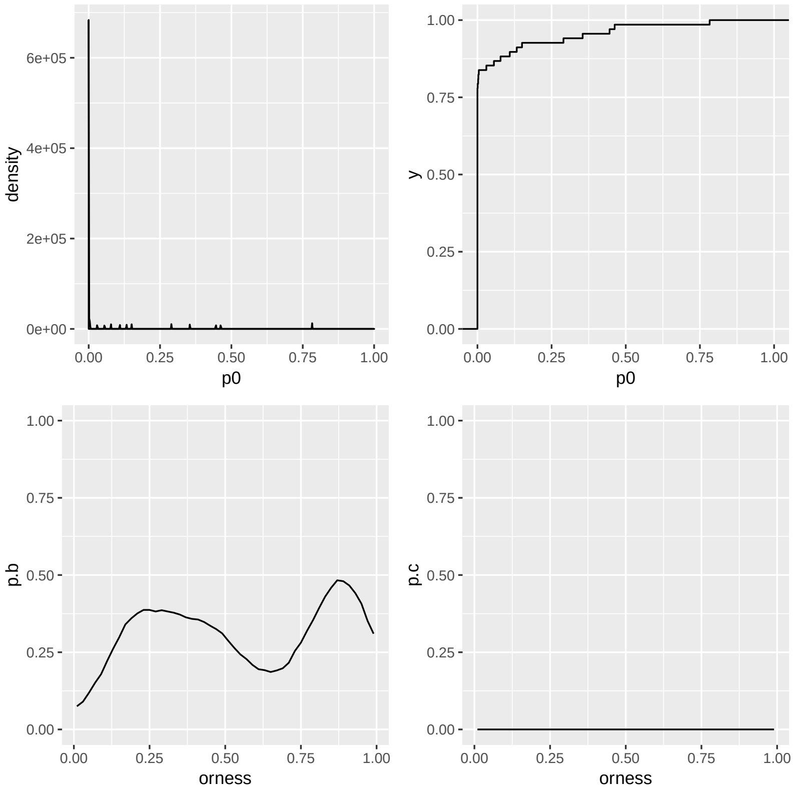


```
png("figures/ecdf_ENSG00000203811.png")
evalOrness(p0=p0,p.b=p1.b,p.c=p1.c,orness=orness0,
           row = which(rownames(p1.c) == "ENSG00000203811"))

## Warning: Removed 1 rows containing non-finite values (stat_density).
## Warning: Removed 1 rows containing non-finite values (stat_ecdf).

dev.off()

## pdf
##   2

evalOrness(p0=p0,p.b=p1.b,p.c=p1.c,orness=orness0,
           row =which(rownames(p1.c) == "ENSG00000095752"))
```

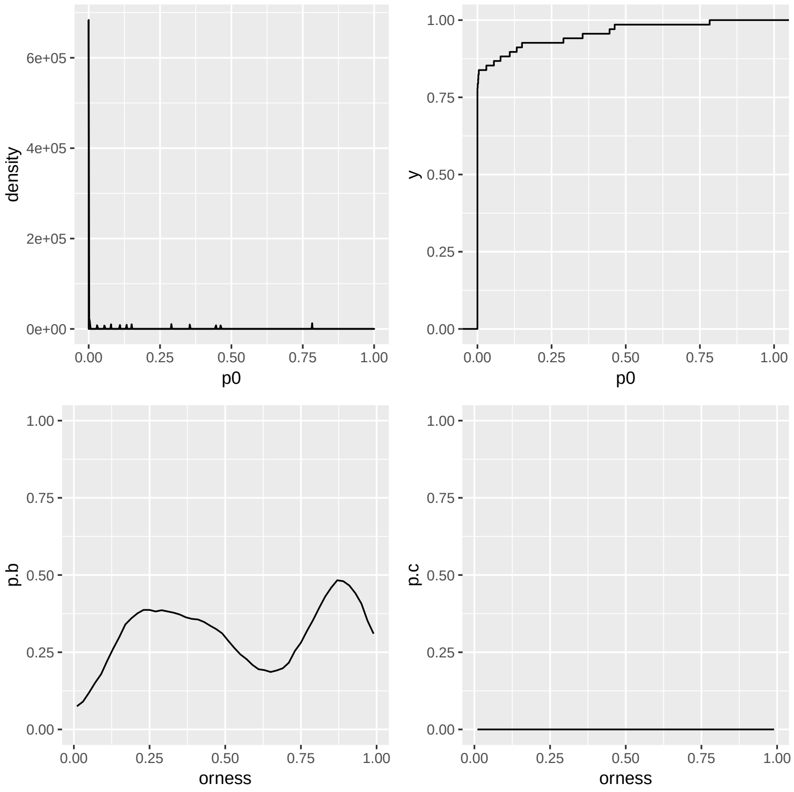


```
rownames(p1.c)[34254]

## [1] "ENSG00000259383"

png("figures/ecdf_ENSG00000259383.png")
evalOrness(p0=p0,p.b=p1.b,p.c=p1.c,orness=orness0,
           row = which(rownames(p1.c) == "ENSG00000259383"))
dev.off()

## pdf
##   2

evalOrness(p0=p0,p.b=p1.b,p.c=p1.c,orness=orness0,
           row =which(rownames(p1.c) == "ENSG00000259383"))
```

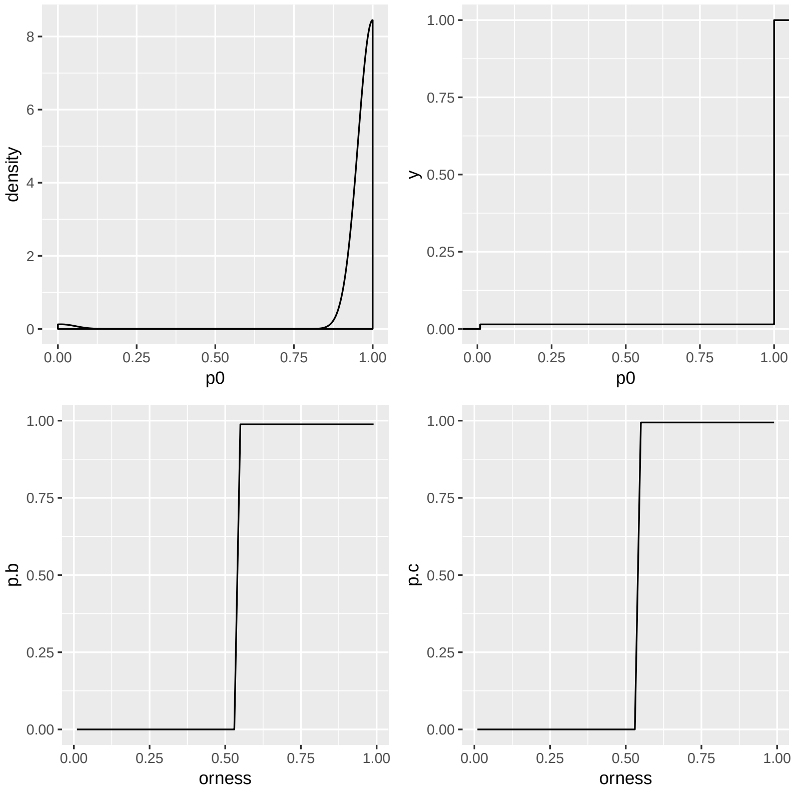

4.3.2 Selected genes using bibliography

We have selected genes in the previous plots using the observed p-values. Now two different gene sets are going to be proposed. The first one are genes that it is known are related with the disease. The second gene set is composed by housekeeping genes not related with the phenotype.

```
significant_names= c("DPEP1","CEACAM6","S100P","CLDN2","CDH3","CXCL8",
                     "REG4","MUC2","AGR2","KRT20")
significant_genes =
    c("ENSG00000015413","ENSG00000086548","ENSG00000163993",
      "ENSG00000165376","ENSG00000062038","ENSG00000169429",
      "ENSG00000134193", "ENSG00000198788","ENSG00000106541",
      "ENSG00000171431")
significant_row = NULL
for(i in significant_genes)
    significant_row = c(significant_row,grep(i,rownames(p1.c)))

housekeeping_names =c("HIST2H3C","TAF5","EDC3","KATNA1","BRAP","NUP188",
                      "CNTRL","ODF2","ACTB","RPS23")
housekeeping_genes =
    c("ENSG00000203811","ENSG00000148835","ENSG00000179151",
      "ENSG00000186625","ENSG00000089234","ENSG00000095319",
      "ENSG00000119397","ENSG00000136811","ENSG00000075624",
      "ENSG00000186468")

housekeeping_row = NULL
for(i in housekeeping_genes)
```

```
    housekeeping_row = c(housekeeping_row,grep(i,rownames(p1.c)))

important = data.frame(
    name = c(significant_names,housekeeping_names),
    gene = c(significant_genes,housekeeping_genes),
    row = c(significant_row,housekeeping_row),
    type = factor(rep(1:2,each=10),
                  levels=1:2,labels=c("significant","housekeeping")))
```

```
## i=1,9
for(i in c(2:8,10:20)){
    filepng = paste0("figures/",important[i,"name"],"_evalOrness.png")
    png(filepng)
    evalOrness(p0 = p0,p.b = p1.b,p.c = p1.c,orness=orness0,
               row =important[i,"row"]  )
    dev.off()
}

## Warning: Removed 1 rows containing non-finite values (stat_density).
## Warning: Removed 1 rows containing non-finite values (stat_ecdf).
## Warning: Removed 1 rows containing non-finite values (stat_density).
## Warning: Removed 1 rows containing non-finite values (stat_ecdf).
```

The names of the genes and the rows in the corresponding matrices of p-values.

```
png("figures/p1_complete.png")
comp.profiles(genes = important[,"name"] ,rowgenes = important[,"row"],
              p1=p1.c,orness=orness0)

## `geom_smooth()` using method = 'loess' and formula 'y ~
x'

dev.off()
comp.profiles(genes = important[,"name"] ,rowgenes = important[,"row"],
              p1=p1.c,orness=orness0)

## `geom_smooth()` using method = 'loess' and formula 'y ~
x'
```

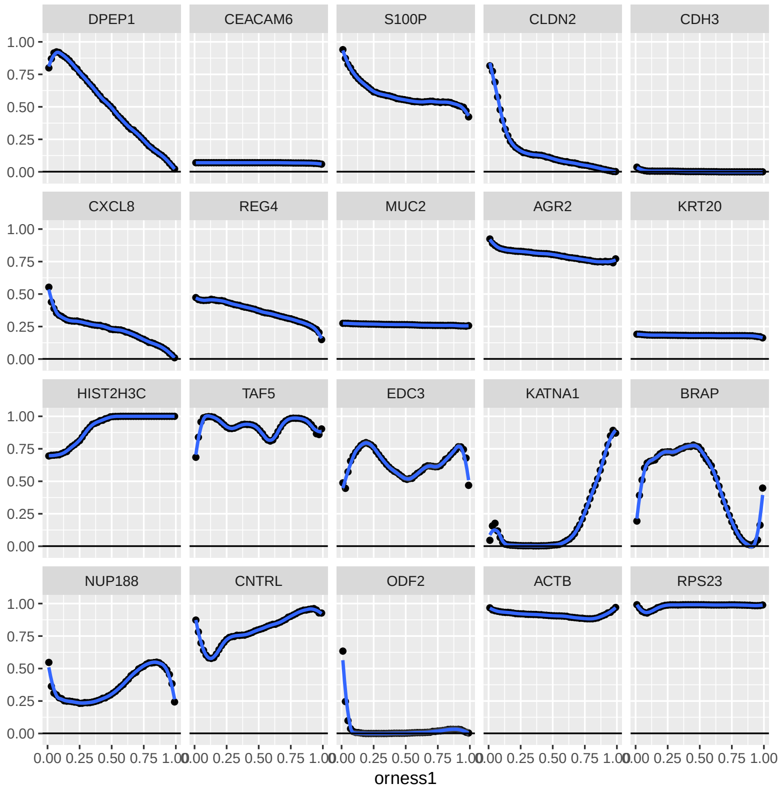


```
png("figures/p1_between-pair.png")
comp.profiles(genes = important[,"name"] ,rowgenes = important[,"row"],
              p1=p1.b,orness=orness0)

## `geom_smooth()` using method = 'loess' and formula 'y ~
x'

dev.off()
comp.profiles(genes = important[,"name"] ,rowgenes = important[,"row"],
              p1=p1.b,orness=orness0)

## `geom_smooth()` using method = 'loess' and formula 'y ~
x'
```

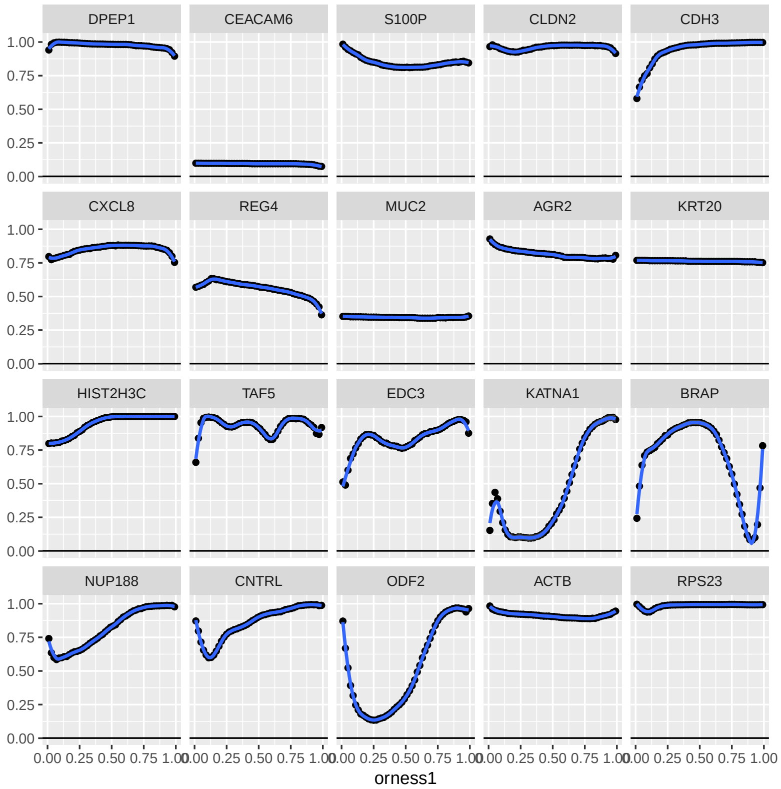

5 Global analysis

In this section we analyze the global results when the orness is modified. First, we load the randomization p-values.

```
load("proportion.p_50_1000_dbinom_between-pair_p1.rda")
load("proportion.p_50_1000_dbinom_complete_p1.rda")
```

Let us evaluate the number of significant genes for different orness.

```
p1 = p1.b
df0 = data.frame(p1,1:nrow(p1))
names(df0) = c(paste("o_",orness0),"gene")
df1 = reshape2::melt(df0,id = "gene")
alphaValues = seq(.01,.1,length.out=10)
aa = sapply(alphaValues,function(alpha) apply(p1<alpha,2,sum))/nrow(p1)

df0 = data.frame(aa,orness0)
df = reshape2::melt(df0,id="orness0")
names(df) = c("orness","alpha","proportion")
levels(df[,"alpha"]) = alphaValues


png("figures/proportionsignificant_between-pair.png")
labels0 = lapply(sprintf('$\\alpha = %4.2f$',alphaValues), TeX)
ggplot(df,aes(x=orness,y=proportion,color=alpha)) +
    geom_line() +
```

```
    xlab("Orness") + ylab(TeX('Proportion of $p \\leq \\alpha$')) +
    guides(color=guide_legend(title=NULL)) +
    scale_color_discrete(labels=labels0)
dev.off()

ggplot(df,aes(x=orness,y=proportion,color=alpha)) +
    geom_line() +
    xlab("Orness") + ylab(TeX('Proportion of $p \\leq \\alpha$'))+
    guides(color=guide_legend(title=NULL)) +
    scale_color_discrete(labels=labels0)
```

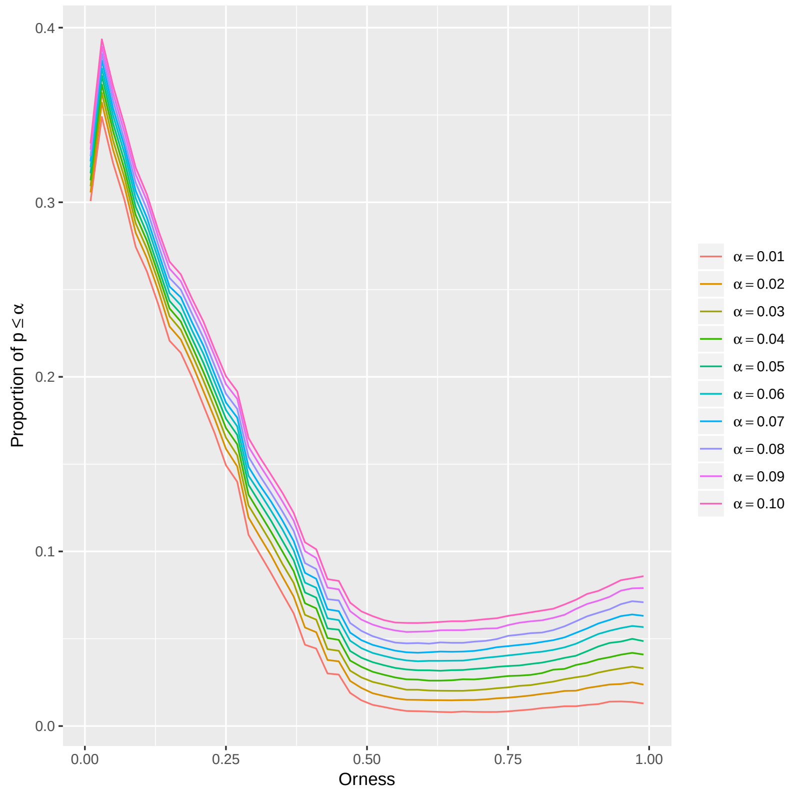


```
p1 = p1.c
df0 = data.frame(p1,1:nrow(p1))
names(df0) = c(paste("o_",orness0),"gene")
df1 = reshape2::melt(df0,id = "gene")

alphaValues = seq(.01,.1,length.out=10)
aa = sapply(alphaValues,function(alpha) apply(p1<alpha,2,sum))/nrow(p1)

df0 = data.frame(aa,orness0)
df = reshape2::melt(df0,id="orness0")
names(df) = c("orness","alpha","proportion")
levels(df[,"alpha"]) = alphaValues

png("figures/proportionsignificant_complete.png")
labels0 = lapply(sprintf('$\\alpha = %4.2f$',alphaValues), TeX)
ggplot(df,aes(x=orness,y=proportion,color=alpha)) +
    geom_line() +
```

```
    xlab("Orness") + ylab(TeX('Proportion of $p \\leq \\alpha$'))+
    guides(color=guide_legend(title=NULL)) +
    scale_color_discrete(labels=labels0)
dev.off()

ggplot(df,aes(x=orness,y=proportion,color=alpha)) +
    geom_line() +
    xlab(TeX('$\\alpha$')) + ylab(TeX('Proportion of $p \\leq \\alpha$'))+
    guides(color=guide_legend(title=NULL)) +
    scale_color_discrete(labels=labels0)
```

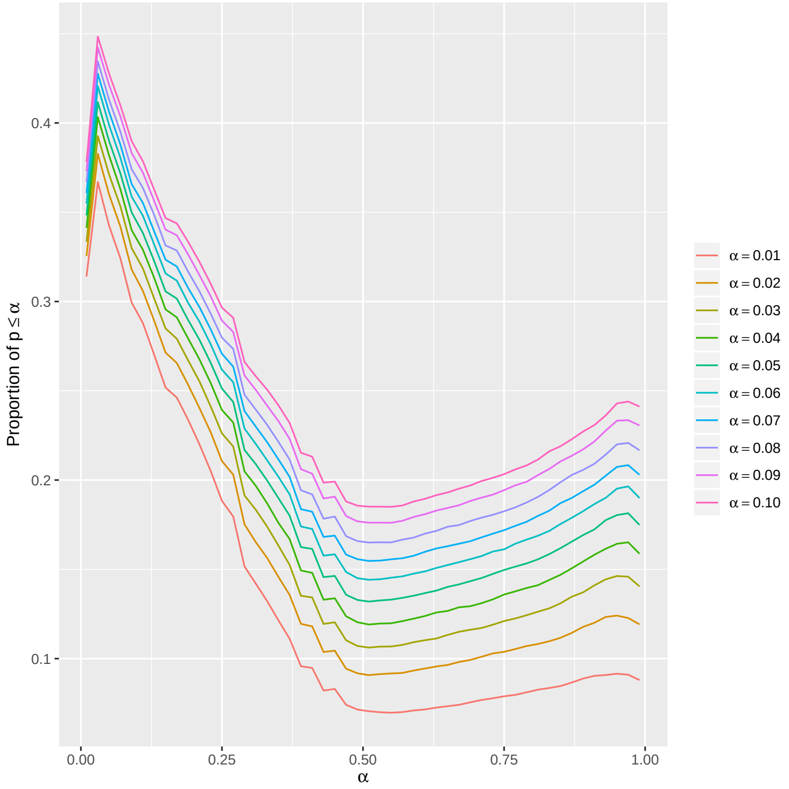

6 Marginal differential expression with a given orness

First, we estimate the number of non significant genes using the package **qvalue**. We use the p-values provided by the method edgeR previously used.

```
library(qvalue)
load("p0.er.rda")
q0 = qvalue(p0.er)
floor(nrow(se)*(q0$pi0))

## [1] 11307
```

We estimate the number of non significant genes around 11000. We will explore the values for orness comparing the lowest and highest p-values following the method proposed in the paper.

```
borness = NULL
n.values = seq(100,10000,10)
for(i in n.values)
    borness = c(borness,chooseOrness(pval = p1.c,orness=orness0,
                                     nmin = i, nmax = i))
save(borness,file="borness.rda")
```

Let us see the results.

```
n.values = seq(100,10000,10)
load("borness.rda")
df = data.frame(n=n.values,orness = borness)
png("choosingOrness.png")
ggplot(df,aes(x=n,y=borness)) + geom_point()
dev.off()

## pdf
##   2

png("choosingOrnessSmooth.png")
library(locfit)
ggplot(df,aes(x=n,y=borness)) +  geom_smooth(method="locfit")
dev.off()

## pdf
##   2
```

We can see two different values for orness to explore. The first one is 0.37 and the second one is 0.93

```
orness0[19]

## [1] 0.37

orness0[47]

## [1] 0.93
```

```
chosen = 19
load("proportion.p_50_1000_dbinom_between-pair_p1.rda")
p1.b = p1[,chosen]
load("proportion.p_50_1000_dbinom_complete_p1.rda")
p1.c = p1[,chosen]
df = data.frame(ENSEMBL = ensembl2url(names(p1.b)),
                p.between=p1.b,
                p.complete=p1.c)
foutput = paste0("justonce",orness0[chosen])
htmlRep1 = HTMLReport(shortName = foutput,title = foutput,
reportDirectory = "./reports")
publish(df,htmlRep1)
finish(htmlRep1)

## [1] "./reports/justonce0.37.html"
```

```
chosen = 47
load("proportion.p_50_1000_dbinom_between-pair_p1.rda")
p1.b = p1[,chosen]
load("proportion.p_50_1000_dbinom_complete_p1.rda")
p1.c = p1[,chosen]
df = data.frame(ENSEMBL = ensembl2url(names(p1.b)),
                p.between=p1.b,
                p.complete=p1.c)
foutput = paste0("justonce",orness0[chosen])
htmlRep1 = HTMLReport(shortName = foutput,title = foutput,
reportDirectory = "./reports")
publish(df,htmlRep1)
finish(htmlRep1)

## [1] "./reports/justonce0.93.html"
```

6.1 Ordering genes

We pretend to sort the genes using the randomization p-values corresponding to all orness values.

What are significant genes? We are going to calculate the area under the function giving the p-values in (for instance) the interval $[0.37, .93]$ suggested by the analysis in the previous section. Details in the paper.

```
ff = function(x)
    (pbeta(.37,shape1=x[1],shape2=x[2])-.05)^2 +
        (pbeta(.93,shape1=x[1],shape2=x[2])-.95)^2
nlm(ff,p=c(.5,.5))

## $minimum
## [1] 2.670996e-12
##
## $estimate
## [1] 4.310396 1.977092
##
## $gradient
## [1]  1.433527e-07 -1.366894e-07
##
## $code
## [1] 1
##
## $iterations
## [1] 22

estimate = nlm(ff,p=c(.5,.5))$estimate
pbeta(.37,shape1 = estimate[1],shape2=estimate[2])

## [1] 0.04999837

pbeta(.93,shape1 = estimate[1],shape2=estimate[2])

## [1] 0.95
```

```
ww = dbeta(orness0,shape1 = estimate[1],shape2=estimate[2])/
    sum(dbeta(orness0,shape1 = estimate[1],shape2=estimate[2]))
```

These weights are plotted with the following code.

```
dbeta0 = function(x)
    dbeta(x,shape1 =  estimate[1],shape2=estimate[2])
png("figures/dbeta_weigth.png")
ggplot(data.frame(x=c(0, 1)), aes(x)) + stat_function(fun=dbeta0)+
    xlab("orness") + ylab("weight")
dev.off()

## pdf
##   2

ggplot(data.frame(x=c(0, 1)), aes(x)) + stat_function(fun=dbeta0)+
    xlab("orness") + ylab("weight")
```

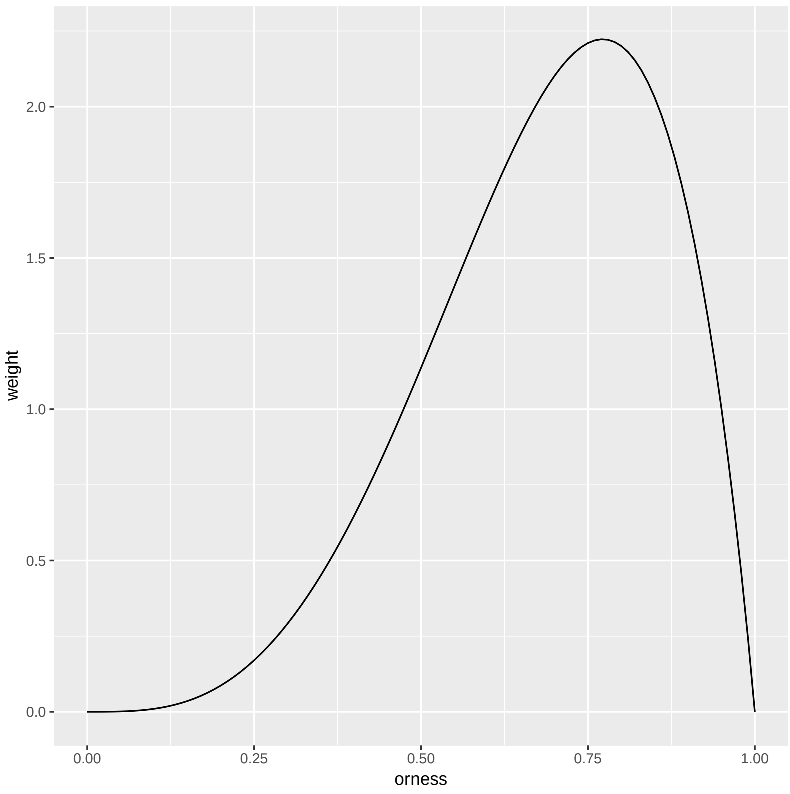


```
load("proportion.p_50_1000_dbinom_between-pair_p1.rda")
p1 = p1.b
A = matrix(rep(ww,nrow(p1)),byrow=TRUE,ncol = length(ww))
p2 = p1 * A
score.between  =
    0.02*(p2[,1]/2 + apply(p2[,-c(1,ncol(p2))],1,sum) + p2[,ncol(p2)]/2)
write.csv(sort(score.between),file="score_between.csv")
df = data.frame(ENSEMBL = ensembl2url(rownames(p1)),score.between)
foutput = "score_between"
htmlRep1 = HTMLReport(shortName = foutput,title = foutput,
```

```
reportDirectory = "./reports")
publish(df,htmlRep1)
finish(htmlRep1)

## [1] "./reports/score_between.html"
```

```
load("proportion.p_50_1000_dbinom_complete_p1.rda")
p1 = p1.c
A = matrix(rep(ww,nrow(p1)),byrow=TRUE,ncol = length(ww))
p2 = p1 * A
score.complete=
    0.02*(p2[,1]/2 + apply(p2[,-c(1,ncol(p2))],1,sum) + p2[,ncol(p2)]/2)
write.csv(sort(score.complete),file="score_complete.csv")
df = data.frame(ENSEMBL = ensembl2url(rownames(p1)),score.complete)
foutput = "score_complete"
htmlRep1 = HTMLReport(shortName = foutput,title = foutput,
reportDirectory = "./reports")
publish(df,htmlRep1)
finish(htmlRep1)

## [1] "./reports/score_complete.html"
```

Some plots using the ordered genes. We consider two gene sets. First, the genes with the lowest scores.

```
score.ix = sort(score.complete,decreasing=FALSE,index.return=TRUE)$ix
lower.score = score.ix[1:50]
score.ix = sort(score.complete,decreasing=TRUE,index.return=TRUE)$ix
upper.score = score.ix[1:50]
```

```
png("figures/lower.score.png")
comp.profiles(genes=rownames(se)[lower.score[1:30]],
              rowgenes=lower.score[1:30],p1=p1.c,orness=orness0)

## `geom_smooth()` using method = 'loess' and formula 'y ~
x'

dev.off()

## pdf
##   2

comp.profiles(genes=rownames(se)[lower.score[1:30]],
              rowgenes=lower.score[1:30],p1=p1.c,orness=orness0)

## `geom_smooth()` using method = 'loess' and formula 'y ~
x'
```

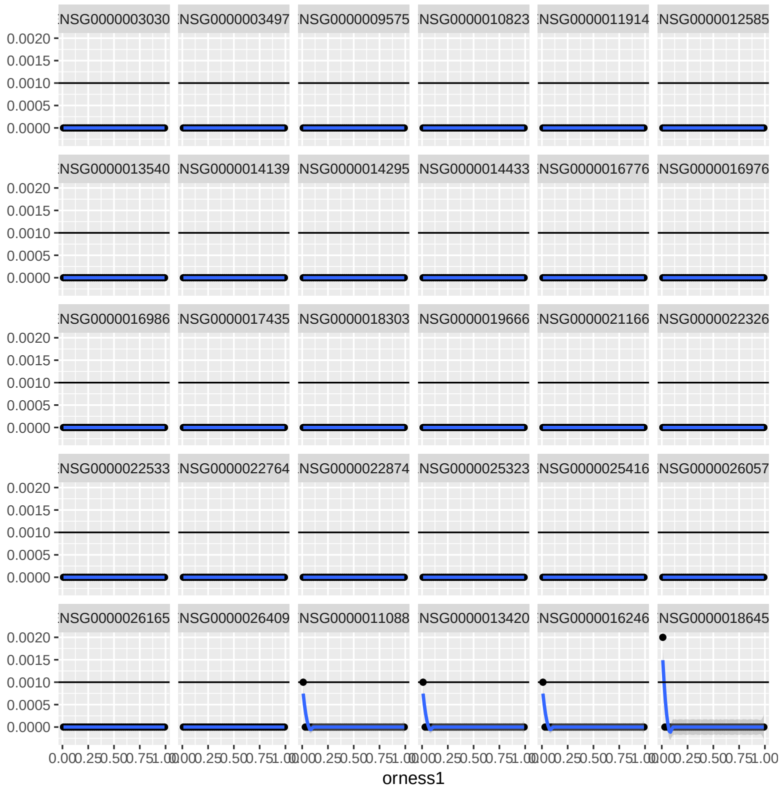


```
png("figures/lower.score.png")
comp.profiles(genes=rownames(se)[upper.score[1:50]],
              rowgenes=upper.score[1:50],p1=p1.c,orness=orness0)

## `geom_smooth()` using method = 'loess' and formula 'y ~
x'

dev.off()

## pdf
##   2

comp.profiles(genes=rownames(se)[upper.score[1:50]],
              rowgenes=upper.score[1:50],p1=p1.c,orness=orness0)

## `geom_smooth()` using method = 'loess' and formula 'y ~
x'
```

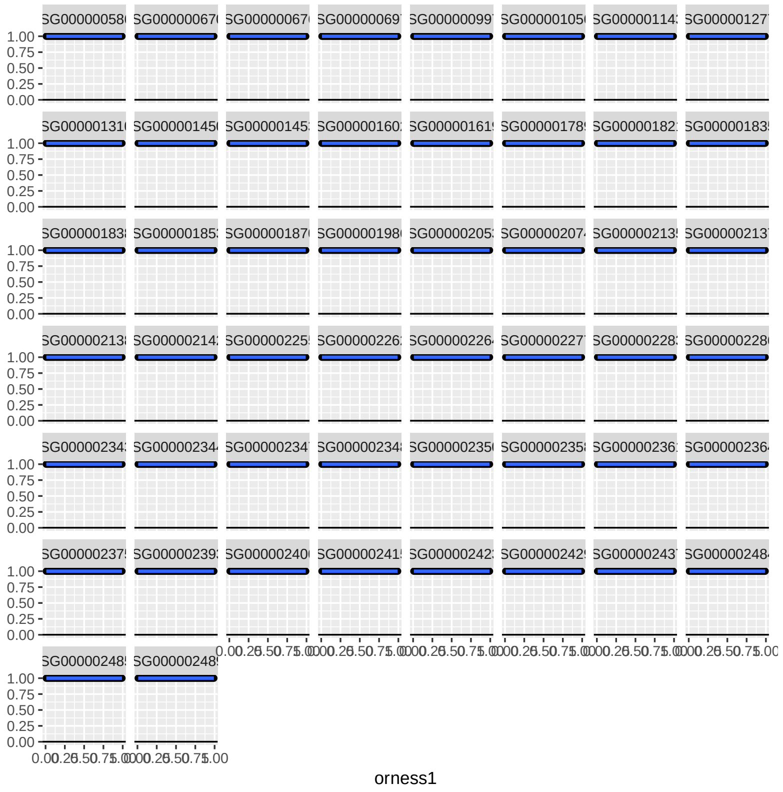

7 Comparative study

We made a unique `data.frame` with the (between and complete) randomization p-values and the p-values obtained using `edgeR`.

```
load("p0.er.rda")
load("p0.de.rda")
load("proportion.p_50_1000_dbinom_complete_p1.rda")
df = data.frame(ensembl = tami::ensembl2url(rownames(se)),
                complete.37 = p1.c[,19],
                complete.93 = p1.c[,47],
                p_edger = p0.er,
                p_deseq2 = p0.de)
foutput = "ComparativeStudy"
htmlRep1 = HTMLReport(shortName = foutput,title = foutput,
                      reportDirectory = "./reports")
publish(df,htmlRep1)
finish(htmlRep1)
```

```
pacman::p_load("VennDiagram")
load("p0.er.rda")
load("p0.de.rda")
load("proportion.p_50_1000_dbinom_complete_p1.rda")
df = data.frame(ensembl = tami::ensembl2url(rownames(se)),
                complete.37 = p1.c[,19],
                complete.93 = p1.c[,47],
```

```
                 p_edger = p0.er,
                 p_deseq2 = p0.de)

alpha = .001
set1 = df[,"complete.37"]<alpha
set2 = df[,"complete.93"]<alpha
set3 = df[,"p_edger"]<alpha
set4 = df[,"p_deseq2"]<alpha

table(set1&set2&set3&set4)

##
## FALSE  TRUE
## 41391   491

df0 = df[set1&set2&set3&set4,]
foutput = "commonVenn"
htmlRep1 = HTMLReport(shortName = foutput,title = foutput,
                      reportDirectory = "./reports")
publish(df0,htmlRep1)
finish(htmlRep1)

## [1] "./reports/commonVenn.html"

png("figures/VennDiagram_000001.png")
grid.newpage()
draw.quad.venn(area1 = sum(set1),
                  area2 = sum(set2),
                  area3 = sum(set3),
                  area4=sum(set4),
                  n12 = sum(set1 & set2),
                  n13 = sum(set1 & set3),
                  n14 = sum(set1 & set4),
                  n23 = sum(set2 & set3),
                  n24 = sum(set2 & set4),
                  n34 = sum(set3 & set4),
                  n123 = sum(set1&set2 & set3),
                  n124 = sum(set1 & set2 & set4),
                  n134 = sum(set1 & set3 & set4),
                  n234 = sum(set2 & set3 & set4),
                  n1234 = sum(set1&set2 & set3 & set4),
                  category = c("complete.37", "complete.93", "edgeR","DESeq2"),
                  lty = "blank",
                  fill = c("orange", "red", "green", "blue"))

## (polygon[GRID.polygon.66529], polygon[GRID.polygon.66530], polygon[GRID.polygon.6653

dev.off()

## pdf
##   2

grid.newpage()
draw.quad.venn(area1 = sum(set1),
                  area2 = sum(set2),
```

```
                  area3 = sum(set3),
                  area4=sum(set4),
                  n12 = sum(set1 & set2),
                  n13 = sum(set1 & set3),
                  n14 = sum(set1 & set4),
                  n23 = sum(set2 & set3),
                  n24 = sum(set2 & set4),
                  n34 = sum(set3 & set4),
                  n123 = sum(set1&set2 & set3),
                  n124 = sum(set1 & set2 & set4),
                  n134 = sum(set1 & set3 & set4),
                  n234 = sum(set2 & set3 & set4),
                  n1234 = sum(set1&set2 & set3 & set4),
                  category = c("complete.37", "complete.93", "edgeR","DESeq2"),
                  lty = "blank",
                  fill = c("orange", "red", "green", "blue"))
```

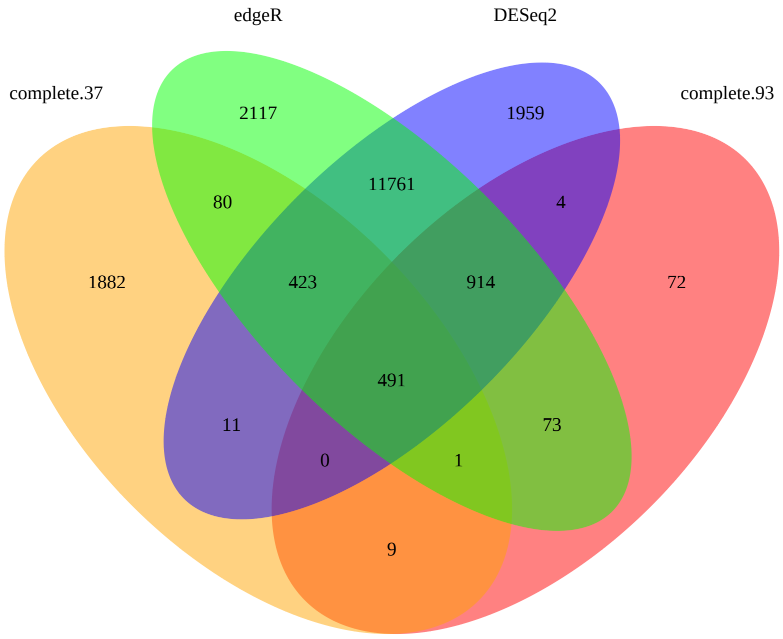


```
## (polygon[GRID.polygon.66556], polygon[GRID.polygon.66557], polygon[GRID.polygon.6655
```

8 Simulation study

A function needed later.

```
do.pci = function(u,foutlier,level = "Out"){

    if(ncol(u$p.value)==3){
```

```
        result =
            c(t.test(u$p.value[foutlier == level,1],
                     u$p.value[foutlier == level,2],
                     paired = TRUE)$conf.int,
              t.test(u$p.value[foutlier == level,1],
                     u$p.value[foutlier == level,3],
                     paired = TRUE)$conf.int,
              t.test(u$p.value[foutlier == level,2],
                     u$p.value[foutlier == level,3],
                     paired = TRUE)$conf.int)
    }

    if(ncol(u$p.value)==4){
        result =
            c(t.test(u$p.value[foutlier == level,1],
                     u$p.value[foutlier == level,2],
                     paired = TRUE)$conf.int,
              t.test(u$p.value[foutlier == level,1],
                     u$p.value[foutlier == level,3],
                     paired = TRUE)$conf.int,
              t.test(u$p.value[foutlier == level,1],
                     u$p.value[foutlier == level,4],
                     paired = TRUE)$conf.int,
              t.test(u$p.value[foutlier == level,2],
                     u$p.value[foutlier == level,3],
                     paired = TRUE)$conf.int,
              t.test(u$p.value[foutlier == level,2],
                     u$p.value[foutlier == level,4],
                     paired = TRUE)$conf.int,
              t.test(u$p.value[foutlier == level,3],
                     u$p.value[foutlier == level,4],
                     paired = TRUE)$conf.int)
        }
    result
}
```

8.1 Poisson distributions

First, we set the parameters of the simulation.

```
test=proportion.p
norness=50
nsim=100
method="dbinom"
is.decreasing=FALSE
proc = c("owb","owc","er")
do.auc=TRUE

N = 500
n = 50
lambda1 = 10
lambda2.values = seq(6,18,4)
lambda3 = 2
```

```
lambda4.values = seq(30,60,2)
nsig = 50
noutlier = rep(0,2)
noutlier[1] = 50
nfilas = length(lambda2.values)*length(lambda4.values)*10
foutlier.head = c(rep(1,noutlier[1]),rep(2,nsig))
foutlier0 = factor(c(foutlier.head,rep(3,N-length(foutlier.head))),
                   levels=1:3,labels=c("Out","Sig","NonSig"))

pci = vector("list",5)
names(pci) = c("parameters",levels(foutlier0),"auc")

pci[["Parameters"]] =  matrix(NA,nrow=nfilas,ncol=4)
pci[["Out"]] = matrix(NA,nrow=nfilas,ncol=6)
pci[["Sig"]] = matrix(NA,nrow=nfilas,ncol=6)
pci[["NonSig"]] = matrix(NA,nrow=nfilas,ncol=6)
pci[["auc"]] = matrix(NA,nrow=nfilas,ncol=3)

i= 0
for(lambda2 in lambda2.values){
    for(lambda4 in lambda4.values){
        lambda = c(lambda1,lambda2,lambda3,lambda4)
        for(outlier2 in 1:10){
            i = i+1
            noutlier[2] = outlier2
            cat("n lambda2 lambda4 outlier[2] ",c(n,lambda2,lambda4,
                                                noutlier[2]),"\n")
            u1 = justDo2(n=n,N=N,nsig=nsig,noutlier=noutlier,
                         theta=lambda,model="Poisson",
               test=proportion.p,norness=50,nsim=100,
               method="dbinom",is.decreasing=FALSE,
               proc = c("owb","owc","er"),do.auc=FALSE)
            pci[["Parameters"]][i,] = c(n,lambda2,lambda4,noutlier[2])
            pci[["Out"]][i,]=do.pci(u=u1,foutlier=foutlier0,level = "Out")
            pci[["Sig"]][i,]=do.pci(u=u1,foutlier=foutlier0,level = "Sig")
            pci[["NonSig"]][i,]=do.pci(u=u1,foutlier=foutlier0,level = "NonSig")
            ##pci[["auc"]][i,] = u1$auc
        }
    }
}
save(pci,file="pci_Poisson_500_50.rda")
```

Now we can plot the results.

```
load("pci_pois_with_auc.rda")
parameters = data.frame(pci$Parameters)
names(parameters) = c("n","lambda2","lambda4","outliers")
Out = data.frame(pci$Out)
names(Out) = c("low.bc.out","up.bc.out","low.be.out","up.be.out",
               "low.ce.out","up.ce.out")

center.bc.out = (Out$low.bc.out+ Out$up.bc.out)/2
center.be.out = (Out$low.be.out+ Out$up.be.out)/2
center.ce.out = (Out$low.ce.out+ Out$up.ce.out)/2
```

```
Out = data.frame(Out,center.bc.out = center.bc.out,
                 center.be.out = center.be.out,
                 center.ce.out = center.ce.out)
Sig = data.frame(pci$Sig)
names(Sig) = c("low.bc.Sig","up.bc.Sig","low.be.Sig","up.be.Sig",
               "low.ce.Sig","up.ce.Sig")
center.bc.Sig = (Sig$low.bc.Sig+ Sig$up.bc.Sig)/2
center.be.Sig = (Sig$low.be.Sig+ Sig$up.be.Sig)/2
center.ce.Sig = (Sig$low.ce.Sig+ Sig$up.ce.Sig)/2
Sig = data.frame(Sig,center.bc.Sig = center.bc.Sig,
                 center.be.Sig = center.be.Sig,
                 center.ce.Sig = center.ce.Sig)

NonSig = data.frame(pci$NonSig)
names(NonSig) = c("low.bc.NonSig","up.bc.NonSig","low.be.NonSig",
                  "up.be.NonSig","low.ce.NonSig","up.ce.NonSig")

center.bc.NonSig = (NonSig$low.bc.NonSig+ NonSig$up.bc.NonSig)/2
center.be.NonSig = (NonSig$low.be.NonSig+ NonSig$up.be.NonSig)/2
center.ce.NonSig = (NonSig$low.ce.NonSig+ NonSig$up.ce.NonSig)/2
NonSig = data.frame(NonSig,center.bc.NonSig = center.bc.NonSig,
                 center.be.NonSig = center.be.NonSig,
                 center.ce.NonSig = center.ce.NonSig)
df = data.frame(parameters,Out,Sig,NonSig)
df[df<0] = 0
names(df) = c(names(parameters),names(Out),names(Sig),names(NonSig))
df0 = df[,c("outliers","lambda2","lambda4",
                "center.bc.out","center.be.out","center.ce.out",
                "center.bc.Sig","center.be.Sig","center.ce.Sig",
                "center.bc.NonSig","center.be.NonSig","center.ce.NonSig")]
df0[df0<0] = 0
df1 = reshape2::melt(df0,id=c("outliers","lambda2","lambda4"))
gene.type = factor(rep(1:3,each=640*3),levels=1:3,
                    labels=c("Outlier","Significant","Non Significant"))
comparison = factor(rep(rep(1:3,each=640),3),levels=1:3,
                    labels=c("between-pair vs complete",
                             "between-pair vs edgeR",
                             "complete vs edgeR"))
df1[,"lambda2"] = factor(df1[,"lambda2"])
df2 = data.frame(df1,comparison,gene.type)
labels0 = lapply(sprintf('$\\lambda_2 = %d$',unique(parameters$lambda2)), TeX)
png("figures/Simulation_Poisson.png")
ggplot(df2[df2$outliers==1,],aes(x=lambda4,y=value,color=lambda2)) +
    geom_line() + ylim(0,1) +
    facet_grid(rows = vars(comparison),cols=vars(gene.type)) +
    xlab(TeX('$\\lambda_4$')) +
    ylab(TeX('Difference of p-values')) +
    guides(color=guide_legend(title=NULL)) +
    scale_color_discrete(labels=labels0)
 dev.off()


## pdf
##   2
```

```
ggplot(df2[df2$outliers==1,],aes(x=lambda4,y=value,color=lambda2)) +
    geom_line() + ylim(0,1) +
    facet_grid(rows = vars(comparison),cols=vars(gene.type)) +
    xlab(TeX('$\\lambda_4$')) +
    ylab(TeX('Difference of p-values')) +
    guides(color=guide_legend(title=NULL)) +
    scale_color_discrete(labels=labels0)
```

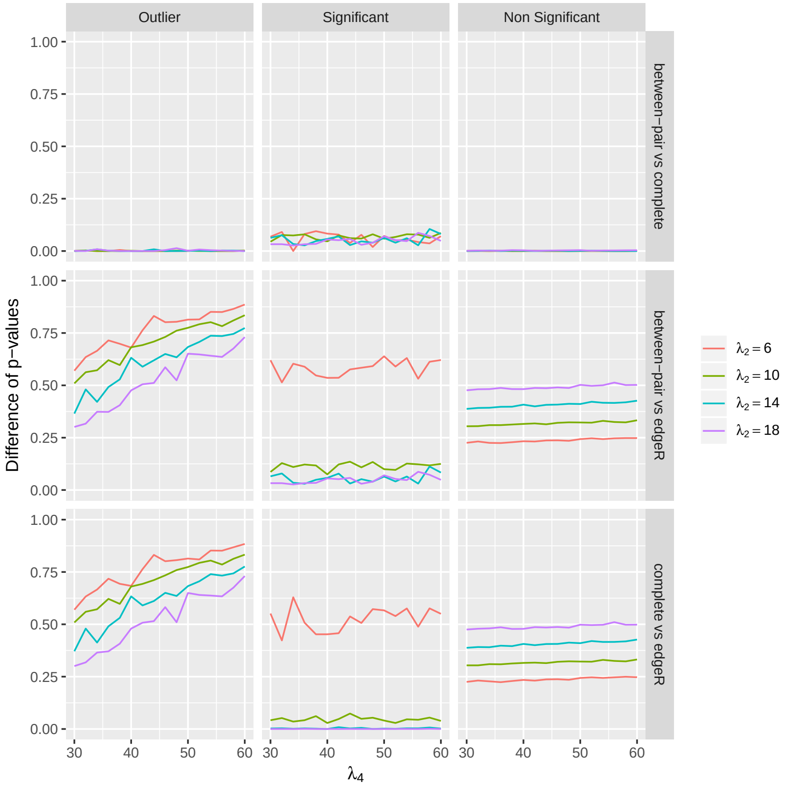

8.2 Negative binomial distributions

First, we set the parameters of the simulation.

```
test=proportion.p
norness=50
nsim0=100
method="dbinom"
is.decreasing=FALSE
proc = c("owb","owc","er")
do.auc=FALSE

N = 500
n = 50
mu1 = 10
mu2.values = seq(20,50,10)
mu3 = 2
mu4.values = seq(60,100,5)
nsig = 10
noutlier = rep(0,2)
```

```r
noutlier[1] = 10
nfilas = length(mu2.values)*length(mu4.values)*10
foutlier.head = c(rep(1,noutlier[1]),rep(2,nsig))
foutlier0 = factor(c(foutlier.head,rep(3,N-length(foutlier.head))),
                   levels=1:3,labels=c("Out","Sig","NonSig"))

pci = vector("list",5)
names(pci) = c("parameters",levels(foutlier0),"auc")

pci[["Parameters"]] =  matrix(NA,nrow=nfilas,ncol=4)
pci[["Out"]] = matrix(NA,nrow=nfilas,ncol=12)
pci[["Sig"]] = matrix(NA,nrow=nfilas,ncol=12)
pci[["NonSig"]] = matrix(NA,nrow=nfilas,ncol=12)
pci[["auc"]] = matrix(NA,nrow=nfilas,ncol=3)

i=0
for(mu2 in mu2.values){
    for(mu4 in mu4.values){
        theta = matrix(NA,nrow=4,ncol=2)
        theta[,1] = c(mu1,mu2,mu3,mu4)
        theta[,2] = rep(10,4)
        for(outlier2 in 1:10){
            i = i+1
            noutlier[2] = outlier2
            cat("n mu2 mu4 outlier[2] ",c(n,mu2,mu4,noutlier[2]),"\n")
            u1 = justDo2(n=n,N=N,nsig=nsig,noutlier=noutlier,
                         theta=theta,model="NB",
               test=proportion.p,norness=50,nsim=nsim0,
               method="dbinom",is.decreasing=FALSE,
               proc = c("owb","owc","er","de"),do.auc=TRUE)
            pci[["Parameters"]][i,] = c(n,mu2,mu4,noutlier[2])
            pci[["Out"]][i,]=do.pci(u=u1,foutlier=foutlier0,level = "Out")
            pci[["Sig"]][i,]=do.pci(u=u1,foutlier=foutlier0,level = "Sig")
            pci[["NonSig"]][i,]=do.pci(u=u1,foutlier=foutlier0,level = "NonSig")
            pci[["auc"]][i,] = u1$auc
        }
    }
}
foutput0 = paste("pci_NB","N",N,"n",n,"nsim",nsim0,"size",10,sep="-")
save(pci,file=paste0(foutput0,".rda"))
```

Now we can plot the results.

```r
load("pci_NB-N-500-n-50-nsim-500-size-10.rda")
##load("pci_NB-N-500-n-50-nsim-100-size-10.rda")

dim(pci[["Out"]])

## [1] 360   6

parameters = data.frame(pci$Parameters)
names(parameters) = c("n","mu2","mu4","outliers")
Out = data.frame(pci$Out)
names(Out) = c("low.bc.out","up.bc.out","low.be.out","up.be.out",
```

```
               "low.ce.out","up.ce.out")

center.bc.out = (Out$low.bc.out+ Out$up.bc.out)/2
center.be.out = (Out$low.be.out+ Out$up.be.out)/2
center.ce.out = (Out$low.ce.out+ Out$up.ce.out)/2


Out = data.frame(Out,center.bc.out = center.bc.out,
                 center.be.out = center.be.out,
                 center.ce.out = center.ce.out)
Sig = data.frame(pci$Sig)
names(Sig) = c("low.bc.Sig","up.bc.Sig","low.be.Sig","up.be.Sig",
               "low.ce.Sig","up.ce.Sig")
center.bc.Sig = (Sig$low.bc.Sig+ Sig$up.bc.Sig)/2
center.be.Sig = (Sig$low.be.Sig+ Sig$up.be.Sig)/2
center.ce.Sig = (Sig$low.ce.Sig+ Sig$up.ce.Sig)/2
Sig = data.frame(Sig,center.bc.Sig = center.bc.Sig,
                 center.be.Sig = center.be.Sig,
                 center.ce.Sig = center.ce.Sig)

NonSig = data.frame(pci$NonSig)
names(NonSig) = c("low.bc.NonSig","up.bc.NonSig","low.be.NonSig",
                  "up.be.NonSig","low.ce.NonSig","up.ce.NonSig")

center.bc.NonSig = (NonSig$low.bc.NonSig+ NonSig$up.bc.NonSig)/2
center.be.NonSig = (NonSig$low.be.NonSig+ NonSig$up.be.NonSig)/2
center.ce.NonSig = (NonSig$low.ce.NonSig+ NonSig$up.ce.NonSig)/2
NonSig = data.frame(NonSig,center.bc.NonSig = center.bc.NonSig,
                 center.be.NonSig = center.be.NonSig,
                 center.ce.NonSig = center.ce.NonSig)

df = data.frame(parameters,Out,Sig,NonSig)
df[df<0] = 0
names(df) = c(names(parameters),names(Out),names(Sig),names(NonSig))
df0 = df[,c("outliers","mu2","mu4",
                "center.bc.out","center.be.out","center.ce.out",
                "center.bc.Sig","center.be.Sig","center.ce.Sig",
                "center.bc.NonSig","center.be.NonSig","center.ce.NonSig")]
df0[df0<0] = 0
df1 = reshape2::melt(df0,id=c("outliers","mu2","mu4"))
gene.type = factor(rep(1:3,each=360*3),levels=1:3,
                   labels=c("Outlier","Significant","Non Significant"))
comparison = factor(rep(rep(1:3,each=360),3),levels=1:3,
                   labels=c("between-pair vs complete",
                            "between-pair vs edgeR",
                            "complete vs edgeR"))
df1[,"mu2"] = factor(df1[,"mu2"])
df2 = data.frame(df1,comparison,gene.type)

labels0 = lapply(sprintf('$\\mu_2 = %d$',unique(parameters$mu2)), TeX)
png("figures/Simulation_NB.png")
ggplot(df2[df2$outliers==1,],aes(x=mu4,y=value,color=mu2)) +
    geom_line() + ylim(0,1) +
```

```
    facet_grid(rows = vars(comparison),cols=vars(gene.type)) +
    xlab(TeX('$\\mu_4$')) +
    ylab(TeX('Difference of p-values')) +
    guides(color=guide_legend(title=NULL)) +
    scale_color_discrete(labels=labels0)

## Warning: Removed 14 rows containing missing values (geom_path).

dev.off()

## pdf
##   2

ggplot(df2[df2$outliers==1,],aes(x=mu4,y=value,color=mu2)) +
    geom_line() + ylim(0,1) +
    facet_grid(rows = vars(comparison),cols=vars(gene.type)) +
    xlab(TeX('$\\mu_4$')) +
    ylab(TeX('Difference of p-values')) +
    guides(color=guide_legend(title=NULL)) +
    scale_color_discrete(labels=labels0)

## Warning: Removed 14 rows containing missing values (geom_path).
```

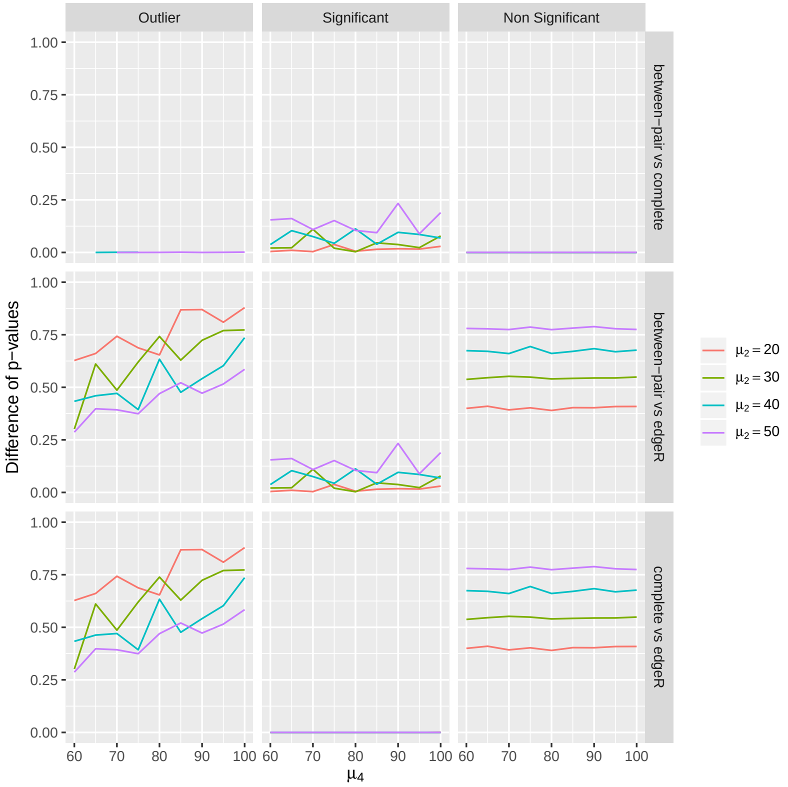

Supplement: Supplementary file 1 — All procedures and data needed to reproduce the whole study have been included in the file SupplementaryMaterial.tar.gz. Once decompressed the file SupplementaryMaterialMethods.pdf contains a detailed description of the methods used and the results obtained. The whole paper can be reproduced reading this file. Other data files generated during the analysis are included in the folder Methods. The detailed html reports with the results can be found in the folder Results. (GZ 118,244 kb) [file 12864_2019_5496_MOESM1_ESM.gz › SupplementaryMaterial/SupplementaryMaterialMethods.pdf]
